# EffectS of Lifestyle Interventions in Older PEople With Obesity (Effective SLOPE): a Systematic Review With Network Meta‐Analyses

**DOI:** 10.1111/obr.70123

**Published:** 2026-04-03

**Authors:** Gabriel Torbahn, Daniel Schoene, Isabel Galicia Ernst, Lukas Schwingshackl, Gerta Rücker, Helge Knüttel, Wolfgang Kemmler, Cornel C. Sieber, John A. Batsis, Dennis T. Villareal, Nanette Stroebele‐Benschop, Dorothee Volkert, Eva Kiesswetter

**Affiliations:** ^1^ Institute for Biomedicine of Aging Friedrich‐Alexander‐Universität Erlangen‐Nürnberg Nuremberg Germany; ^2^ Department of Pediatrics Paracelsus Medical University Nuremberg Germany; ^3^ Department of Pediatrics Paracelsus Medical University Salzburg Austria; ^4^ Obesity Research Unit Paracelsus Medical University Salzburg Austria; ^5^ Institute of Radiology University Hospital Erlangen Erlangen Germany; ^6^ Department of Clinical Gerontology, Robert Bosch Hospital Stuttgart Germany; ^7^ Institute of Epidemiology and Medical Biometry Ulm University Ulm Germany; ^8^ Institute for Evidence in Medicine, Faculty of Medicine Medical Center ‐ University of Freiburg Freiburg Germany; ^9^ Institute of Medical Biometry and Statistics, Faculty of Medicine Medical Center ‐ University of Freiburg Freiburg Germany; ^10^ University Library University of Regensburg Regensburg Germany; ^11^ Department of Medicine Kantonsspital Winterthur, Winterthur Zurich Switzerland; ^12^ Division of Geriatric Medicine, School of Medicine University of North Carolina at Chapel Hill Chapel Hill North Carolina USA; ^13^ Department of Nutrition, Gillings School of Global Public Health University of North Carolina at Chapel Hill Chapel Hill North Carolina USA; ^14^ Division of Endocrinology, Diabetes and Metabolism Baylor College of Medicine Houston Texas USA; ^15^ Department of Nutritional Psychology, Institute of Nutritional Medicine University of Hohenheim Stuttgart Germany

**Keywords:** aged, lifestyle interventions, network meta‐analysis, obesity, sarcopenic obesity

## Abstract

**Background/Aim:**

We conducted a systematic review with network meta‐analyses (NMA) summarizing the effects and safety of lifestyle interventions containing nutrition (NUT; e.g., calorie restriction), exercise (EX; e.g., aerobic/resistance exercise) and behavior change interventions (BCI; e.g., behavioral therapy) on physical function, body composition, quality of life, psychosocial outcomes, health and adverse events in community‐dwelling older adults with obesity.

**Methods:**

We used the methodology proposed by Cochrane and searched six databases and one trial registry for eligible randomized controlled trials (RCTs; intervention duration ≥ 12 weeks) up to May 2022 with a full new search in MEDLINE and a re‐assessment of previously identified eligible trial registry entries in October 2025. Random‐effects NMA ((standardized) mean difference ((S)MD), 95% confidence intervals) were conducted if possible.

**Results:**

We included 72 RCTs (*n* = 6716) for descriptive summaries and 54 RCTs (*n* = 4249) for NMA. NUT+EX+BCI improved physical function (performance batteries) compared to control (SMD 3.37 [1.76;4.97]; high certainty of evidence). NUT+EX+BCI may reduce body (MD −8.69 [−13.14;−4.25]) and fat mass (MD −6.58 [−10.44;−2.73]) while not negatively affecting fat‐free mass (MD −1.38 [−3.52;0.76]) or bone mineral density (MD −0.01 [−0.05;0.02]) (evidence very uncertain). Other interventions (single/combined) may also be effective; however, effects were often imprecise. For psychosocial outcomes, quality of life, and health events, data were insufficient or too heterogeneous to derive clear results.

**Conclusion:**

The evidence suggests that NUT+EX+BCI interventions are most suitable for the management of obesity in older adults. Nevertheless, further RCTs—especially in frail populations and on patient‐relevant outcomes—are needed.

**Abbreviations**
AE: Aerobic exercise
BCI: Behavioral change intervention
BMD: Bone mineral density
CI: Confidence interval
CoE: Certainty of evidence
CR: Calorie restriction
EX: Exercise
FFM: Fat‐free mass
FM: Fat mass
GLP‐1RA: Glucagon‐like peptide‐1 receptor agonists
GRADE: Grading of Recommendations, Assessment, Development and Evaluation
IRR: Incidence rate ratio
HP: High protein diet
IBT: Incretin‐based therapies
NUT: Nutrition
RE: Resistance exercise
RCT: Randomized controlled trial
RoB: Risk of bias
SAT: Subcutaneous adipose tissue
VAT: Visceral adipose tissue
VFA: Visceral fat area
VO2max: Peak oxygen uptake
WL: Weight loss
(C)NMA: (Component) Network meta‐analysis
(S)MD: (Standardized) Mean difference

## 1 Background

Obesity in older people is a growing worldwide public health concern [1]. This is driven by an aging population and the increasing prevalence of obesity in older age groups, with advances in medical care enabling longer lifespans but leaving a population living with conditions that contribute to morbidity [2]. In the United States, simulations show a further growing prevalence of obesity and severe obesity at least until 2030 [3]. Apart from well‐known negative health outcomes associated with obesity across the lifespan, such as diabetes and cardiovascular disease [4], obesity is particularly problematic for older people due to its influence on outcomes affecting daily life, e.g., risk of falls, multimorbidity, chronic pain, frailty, and functional impairments [5, 6, 7, 8, 9, 10]. Sarcopenia, a geriatric syndrome, which is defined as the loss of muscle mass, strength and function [11], also exists in those living with obesity [12]. In older people with obesity and sarcopenia (*sarcopenic obesity*), negative health consequences, for example a higher cardiovascular risk, are considerably more pronounced [13]. Pathophysiological alterations in muscle structure, e.g., fat infiltration, may negatively impact muscle function and subsequently raise the risk for further functional decline [14, 15, 16].

Currently, very few specific evidence‐based recommendations exist for the management of obesity in older people [17]. Similar to younger age groups, clinical practice guidelines recommend non‐pharmacological and non‐surgical multicomponent lifestyle interventions (e.g., calorie restriction [CR] and exercise [EX]) as a first‐line strategy [18, 20]. However, in older adults, age‐related declines in muscle and bone mass, coupled with reduced functional reserves, make the adverse effects of weight loss (WL) on these structures and their associated functions a significant concern. Such losses may increase the risk of sarcopenia, reduced physical functioning and fall‐related fractures, ultimately jeopardizing long‐term independence [15, 21].

Published systematic reviews on the efficacy and safety of lifestyle interventions for the management of obesity in older people have focused on nutrition (NUT) and exercise (EX), but few have considered behavior change interventions (BCIs) as an individual component [22, 23, 24, 25]. This is an important gap as evidence from younger populations highlights the importance of this component for sustained effects [25]. Due to different intervention types (i.e., NUT, EX, or BCI), modalities (e.g., CR, aerobic exercise [AE], or resistance exercise [RE]) and doses potentially leading to high heterogeneity, pairwise meta‐analysis may not be preferred to summarize available evidence [26]. On the contrary, network meta‐analysis (NMA) allows comparing available results of intervention arms directly and indirectly, regardless of whether they have been directly compared in published RCTs [27]. To the best of our knowledge, no systematic review with NMA exists comparing single or combined lifestyle intervention components in older adults aged 65 years and over with obesity.

Therefore, we aimed at summarizing the current evidence on the effects and safety of lifestyle interventions to manage obesity in older people by conducting a comprehensive systematic review.

## 2 Methods

The detailed methods are described in the published protocol and the Supporting information 1 (supplemental methods) [28]. We prospectively registered the work on the Prospective Register of Systematic Reviews (CRD42019147286) and adhered to the reporting guidelines for systematic reviews and its extensions (PRISMA‐statement 2020) as well as for NMA [30, 31, 32].

### 2.1 Search, Study Selection, and Data Extraction

We included RCTs enrolling older adults (age at inclusion ≥ 60 years *and* mean age of ≥ 65 years) with obesity (BMI ≥ 30 kg/m^2^ or waist circumference of ≥ 88 cm for women [w] and ≥ 102 cm for men [m] or percentage body fat of ≥ 35% [w] and ≥ 25% [m] or ethnic‐specific cutoff values) or sarcopenic obesity (sarcopenia was defined based on the criteria used by the primary RCTs) that evaluated the effects of lifestyle interventions consisting of NUT (e.g., CR or high protein [HP]), EX (e.g., AE or RE), or BCI (e.g., motivational interviewing)—or their combinations with a duration of ≥ 12 weeks compared to each other or control—on physical function (primary outcome), body composition, quality of life, psychosocial outcomes, and health as well as therapy‐related adverse events. We searched Medline, Embase, CENTRAL, CINAHL, PsycINFO, Science Citation Index Expanded, and ClinicalTrials.gov from inception until May 25, 2022, without language restrictions (Supporting information 1 Table S1A–G). The search strategies contained a combination of keywords and database‐specific thesaurus vocabulary. To capture the latest evidence, we undertook a full search in MEDLINE on October 14, 2025 and re‐checked the status of potential relevant ongoing studies brought up by the search in ClinicalTrials.gov. Two reviewers (GT, DS, EK, IGE) independently conducted the pre‐piloted review steps: screening of titles/abstracts, full‐texts, data extraction, risk of bias assessment (Cochrane Risk of Bias 2; Supporting information Table S2), and evaluation of the certainty of evidence (CoE) according to the framework proposed by the Grading of Recommendations, Assessment, Development and Evaluation (GRADE) working group (Supporting information 1 Table S3) [33, 34]. Conflicts were solved by discussion or by a third reviewer.

### 2.2 Evidence Synthesis and GRADE Assessment

If possible, we conducted random effects NMA using a frequentist approach and component NMA (CNMA) to disentangle the effects of single components in multicomponent treatment arms and to reconnect disconnected networks using R studio software and R packages [35, 36, 37]. We pooled continuous effects for performance batteries, chair rise, gait speed, handgrip strength, cardiovascular fitness (VO_2_max), body mass, fat‐free mass (FFM), fat mass (FM), waist circumference, BMD of the total hip using the mean difference (MD), or the standardized mean difference (SMD). For the chair rise test, we pooled effects as a so‐called “chair rise rate ratio” (see Supporting information 1 Supplemental methods). We defined network nodes on the level of intervention types (NUT, EX, BCI, or any combinations; main analysis) and more specifically on the level of treatment modalities by subdividing NUT interventions in CR and HP, EX interventions in AE and RE. Forest plots show results of any intervention compared to control. League tables provide additional results between the different interventions. When NMA was not possible, we synthesized the results descriptively. We evaluated the CoE for the six prioritized outcomes: (1) performance batteries as composite measure of functional status, (2) cardiovascular fitness (VO_2_max), (3) FFM, (4) FM, (5) body mass, and (6) BMD of the total hip.

## 3 Results

### 3.1 Literature Search and Screening

The search resulted in 68,928 records from databases, 8660 records from the trial register and three additional records from citation searching and organizations. After removing duplicates, we screened 59,362 titles/abstracts and 1604 full texts for eligibility. Finally, we included 137 records from 72 RCTs (Supporting information 1 Figure S1, Supporting information 1 Table S5) [38, 39, 40, 41, 42, 43, 44, 45, 46, 47, 48, 49, 50, 51, 52, 53, 54, 55, 56, 57, 58, 59, 60, 61, 62, 63, 64, 65, 66, 67, 68, 69, 70, 71, 72, 73, 74, 75, 76, 77, 78, 79, 80, 81, 82, 83, 84, 85, 86, 87, 88, 89, 90, 91, 92, 93, 94, 95, 96, 97, 98, 99, 100, 101, 102, 103, 104, 105, 106, 107, 108, 109]. We did not include 18 RCTs in any NMA but summarized the results descriptively. Ten were not eligible as node definitions for the study arms did not differ [41, 42, 45, 62, 71, 77, 82, 93, 105, 107], six, as given units and effect measures did not match with those of the NMA [38, 50, 56, 59, 75, 97], one RCT due to an insufficient description of the intervention to correctly assign study arms to our node definitions [64] and one RCT as results were not reported per study arm [94].

### 3.2 Study and Participants' Characteristics

All included RCTs (*n* = 6716 participants) were parallel RCTs with the exception of two cluster RCTs (Table 1 and Supporting information 2 Table S6) [89, 106].

**TABLE 1:** Study characteristics of included randomized controlled trials (*n* = 72).

Characteristics		*n*	%
**Country**	North America	37	51.4
	Asia	17	23.6
	Europe	10	13.9
	South America	3	4.2
	Africa	3	4.2
	Australia	2	2.8
**Sample size**	< 50	33	45.8
	50 to < 100	20	27.8
	100 to < 200	13	18.1
	≥ 200	6	8.3
**Mean age of participants** ^**a**^	65 to < 70 years	45	63.4
	70 to < 75 years	20	28.2
	≥ 75 years	6	8.5
**Sex**	Female	18	25.0
	Male	5	6.9
	Both	46	63.9
	Not reported	3	4.2
**Participants with functional limitations** (objectively measured)	Yes	12	16.7
	No	60	83.3
**Phenotype**	Obesity	62	86.1
	Sarcopenic obesity	10	13.9
**Obesity criterion**	Body mass index	57	79.2
	Waist circumference	5	6.9
	% Body fat	6	8.3
	Combination	4	5.6
**Intervention duration**	12–19 weeks	38	52.8
	20–26 weeks	25	34.7
	27–51 weeks	1	1.4
	≥ 52 weeks	8	11.1
**Intervention arms**	Control	51	31.1
**(*n* = 164)**	BCI	4	2.4
	Nut^b^	13	7.9
	Ex	49	29.9
	Nut + BCI	9	5.5
	Ex + BCI	2	1.2
	Nut + Ex	24	14.6
	Nut + Ex + BCI	12	7.3

Abbreviations: BCI, behavior change intervention; Ex, exercise; Nut, nutrition.

^a^ Travieso et al. [108]; mean age not reported (age range 60–75).

^b^ Muscariello et al. [64]; not included here.

For RCTs reporting age at baseline, this ranged from 65.0 to 77.4 years with no studies having a mean age of ≥ 80 or a minimum age of ≥ 75 years. Twelve RCTs used objectively measured functional limitations as an inclusion criterion [59, 60, 61, 63, 74, 85, 86, 87, 95, 96, 100, 106]. Ten RCTs were conducted in older people with sarcopenic obesity [41, 53, 54, 61, 65, 66, 72, 96, 100, 106].

### 3.3 Intervention Characteristics

Forty‐seven RCTs conducted multicomponent interventions, with study arms most often combining NUT+EX (*n* = 24 RCTs) (Table 1, Supporting information 2 Table S7).

The combination of NUT+EX+BCI (*n* = 12 RCTs) and NUT+BCI (*n* = 9 RCTs) was used less frequently, while there were only two RCTs combining EX+BCI. The most often delivered single intervention was EX (*n* = 49 RCTs), followed by NUT (*n* = 13 RCTs) and BCI (*n* = 4 RCTs).

Control conditions comprised no intervention or usual care, educational materials and lectures, or written recommendations on healthy aging, or physical activity. Furthermore, unsupervised home‐based exercise recommendations were also considered as a control condition. Interventions lasted mainly between 12 and 19 weeks (38 RCTs) or 20–26 weeks (25 RCTs). A follow‐up after intervention completion was done in five RCTs lasting 4–78 weeks [61, 66, 68, 80, 89]. Detailed intervention characteristics are displayed in Supporting information 2 Table S7.

### 3.4 Risk of Bias

Across all objectively measured outcomes, we rated the RoB as high in 23, some concerns in 48, and low in 10 RCTs, while for subjectively measured outcomes we rated RoB as high in four, and some concerns in 13 RCTs (see Supporting information 1 Figures S2 and S3). For objective outcomes, most concerns arose from insufficient reporting of the randomization process (64.3%), deviations from intended interventions (58.9%), and selection of the reported results (64.4%). We also could not find evidence for publication bias based on the funnel plots (Supporting information 1 Table S15–S24) or the results of the search in the trial registry.

### 3.5 NMA Characteristics

Information on characteristics of NMA, such as the number of included RCTs and participants, the number of pairwise comparisons, or measures of inconsistency are summarized in Table 2. In all NMA with connected networks, we could not find evidence for statistically significant inconsistency based on the global test for inconsistency (i.e., design‐by‐treatment interaction in the random effects model). We could not evaluate the inconsistency for disconnected networks.

**TABLE 2:** Characteristics of network meta‐analyses.

Outcome category	Outcome	Treatment level	*N* RCTs	*N* participants	*N* treatments	*N* pairwise comparisons	Inconsistency (Q statistic and *p*)
**Physical function**	Performance batteries	Type	16	1442	8	23	1.84; 0.93
		Modality^a^	18	1586	17	37	n.a.
	Gait speed	Type	20	1548	6	29	0.57; 1.00
		Modality	20	1548	15	36	0.91; 1.00
**Strength measures**	Chair rise test	Type	9	441	5	8	0.00; n.a.
		Modality^b^	9	441	8	9	n.a.
	Handgrip strength	Type	13	771	7	17	0.23; 0.97
		Modality^c^	14	838	12	20	n.a.
**Endurance and aerobic capacity**	VO_2_max	Type	13	844	6	23	0.63; 0.96
		Modality	14	921	12	34	1.51; 0.98
**Body composition**	Fat‐free mass	Type	26	1565	7	38	1.48; 1.00
		Modality	27	1632	18	44	0.49; 1.00
	Fat mass	Type	28	1776	7	42	1.93; 1.00
		Modality	30	1920	20	54	3.79; 0.99
	Waist circumference	Type	24	2146	8	31	0.60; 1.00
		Modality^d^	25	2213	16	39	n.a.
	Body weight	Type	43	3198	8	55	1.53; 1.00
		Modality	45	3344	19	69	2.14; 1.00
	Bone mineral density (total hip)	Type^e^	4	420	6	9	n.a.
		Modality^f^	4	420	8	14	n.a.

Abbreviation: n.a., not applicable.

^a^ Subnetwork 1: 17 RCTs, 36 pairwise comparisons, 15 treatments; subnetwork 2: 1 RCT, 1 pairwise comparison, 2 treatments.

^b^ Subnetwork 1: 7 RCTs, 7 pairwise comparisons, 5 treatments; subnetwork 2: 2 RCTs, 2 pairwise comparisons, 3 treatments.

^c^ Subnetwork 1: 12 RCTs, 18 pairwise comparisons, 8 treatments; subnetwork 2: 1 RCT, 1 pairwise comparison, 2 treatments; subnetwork 3: 1 RCT, 1 pairwise comparison, 2 treatments.

^d^ Subnetwork 1: 23 RCTs, 39 pairwise comparisons, 16 treatments; subnetwork 2: 1 RCT, 1 pairwise comparison, 2 treatments; subnetwork 3: 1 RCT, 1 pairwise comparison; 2 treatments.

^e^ Subnetwork 1: 3 RCTs, 8 pairwise comparisons, 4 treatments; subnetwork 2: 1 RCT, 1 pairwise comparison, 2 treatments.

^f^ Subnetwork 1: 3 RCTs, 13 pairwise comparisons, 6 treatments; subnetwork 2: 1 RCT, 1 pairwise comparison, 2 treatments.

RCTs that have been considered for statistical syntheses by NMA for respective outcomes are presented in Supporting information 2 Table S6.

### 3.6 Outcomes

#### 3.6.1 Physical Function: Performance Batteries

**FIGURE 1:**
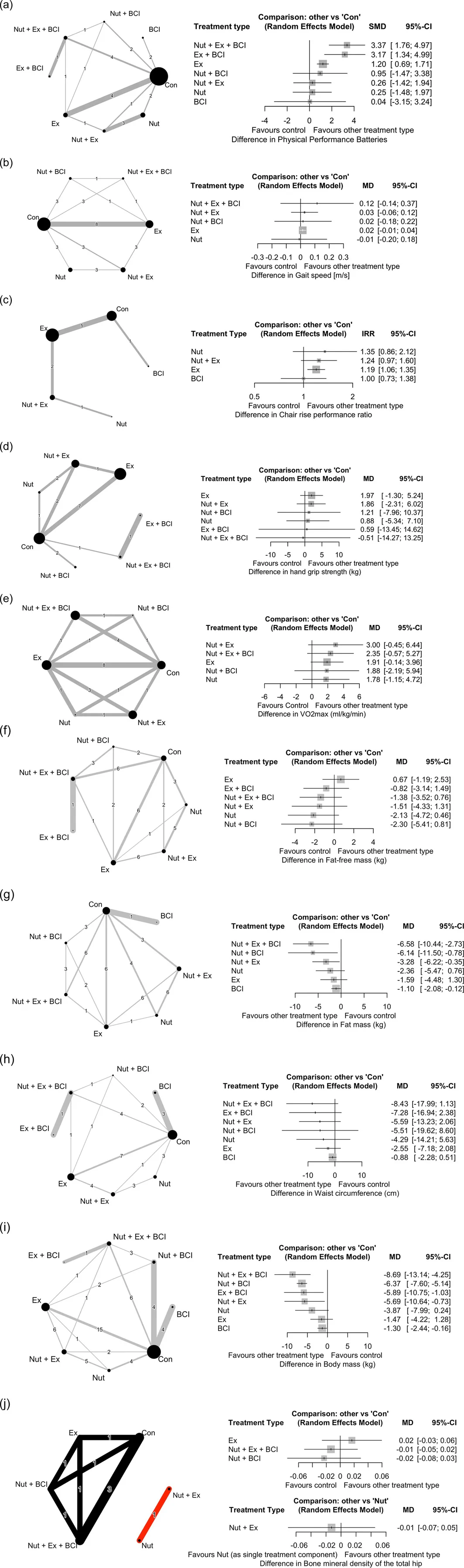
(A) Forest plot and network graph of network meta‐analysis of different intervention types on physical function measured by performance batteries. (B) Forest plot and network graph of network meta‐analysis of different intervention types on physical function measured by gait speed (m/s). (C) Forest plot and network graph of network meta‐analysis of different intervention types on physical function measured by chair rise test. (D) Forest plot and network graph of network meta‐analysis of different intervention types on functional status measured by handgrip strength (kg). (E) Forest plot and network graph of network meta‐analysis of different intervention types on functional status measured by VO2max (mL/kg/min). (F) Forest plot and network graph of network meta‐analysis of different intervention types on fat‐free mass (kg). (G) Forest plot and network graph of network meta‐analysis of different intervention types on fat mass (kg). (H) Forest plot and network graph of network meta‐analyses of different intervention types on waist‐circumference (cm). (I) Forest plot and network graph of network meta‐analysis of different intervention types on body weight (kg). (J) Forest plot and network graph of network meta‐analyses of different intervention types on bone mineral density of the total hip (g/cm^2^). BCI, behavior change intervention; Con, control; Ex, exercise; MD, mean difference; Nut, nutrition.

The NMA revealed that, compared to control, the most effective intervention types were NUT+EX+BCI (SMD 3.37 [95%CI 1.76; 4.97]; high CoE) and EX+BCI (SMD 3.17 [95%CI 1.34; 4.99]; high CoE). EX alone may also present a large effect (SMD 1.20 [95%CI 0.69; 1.71]; very low CoE). All other intervention types may not show effects compared to control (very low—low CoE) (Figure 1A, Supporting information 1 Table S8A). NUT+EX+BCI may be more beneficial for physical performance than NUT+EX, solely EX or NUT (all very low CoE) (Supporting information 1 Table S9A). EX+BCI may show higher effects compared to NUT alone (very low CoE) or NUT+EX (very low CoE).

Regarding treatment modality, most interventions that included AE, RE, or the combination of both were more effective compared to control (Supporting information 1 Figure S4A and Supporting information 1 Table S10A). The combination of AE+RE showed a larger effect when compared to solely AE or RE. CNMA demonstrated results with effects not changing the direction but with narrower 95%CIs for both analyses (Supporting information 1 Figures S5A and B), with the exception for BCI showing an effect on type and modality level. For the nine RCTs that have not been included in NMA, no differences between intervention types and modalities were reported (Supporting information 2 Table S11A).

#### 3.6.2 Physical Function: Mobility

Neither in the NMA on treatment type (Figure 1B, Supporting information 1 Table S9B) nor on modality (Supporting information Figure S4B, Supporting information Table S10B) did we find any group differences in gait speed.

Results for CNMA were similar but with narrower 95%CIs (Supporting information 1 Figures S6A and B). Results on RCTs not included in NMA are shown in Supporting information 2 Table S11A.

Based on a descriptive synthesis, seven RCTs reporting results on the timed‐up‐and‐go test showed improvements by AE, RE, or AE+RE when compared to control (Supporting information 2 Table S11A). Mobility assessed by the 400 m walk (*n* = 5) improved only in participants with class 2 obesity following RE+AE [58].

#### 3.6.3 Physical Function: Strength Measures

For functional strength chair rise performance was used, EX (IRR 1.19 [95%CI 1.06; 1.35]) and NUT+EX (IRR 1.24 [95%CI 0.97; 1.60]) were more favorable than control (Figure 1C), although the effect for NUT+EX was imprecise (Supporting information 1 Table S9C).

The NMA on treatment modalities showed a better chair rise performance only for RE compared to control, while other comparisons did not reveal any differences (Supporting information 1 Figure S4C, Supporting information 1 Table S10C). Results for CNMA were similar but with narrower 95% CIs (Supporting information 1 Figure S7A and B). Ten RCTs could not be included in NMA. In three RCTs, EX (AE, RE, or AE+RE) showed higher effects than control. Another RCT demonstrated better results for hypoxic vs. normoxic training (Supporting information 2 Table S11A) [70].

Instrumented leg strength was measured as leg power in five RCTs, and as maximum strength in 16 trials (*n* = 6 leg press, *n* = 11 knee extension). Lower leg strength following intervention was only shown for three comparisons (CR vs. Con (leg press), CR+BCI vs. AE+RE and CR+AE+BCI vs. CR+RE+BCI (knee extension) (Supporting information 2 Table S11A).

In NMA and CNMA, for handgrip strength, no intervention type or modality showed effects when compared to control (Figures 1D and S4D) or to another intervention (Supporting information 1 Tables S9D and S10D) (Supporting information 1 Figures S8A and B).

Results were similar for RCTs not included in NMA (Supporting information 2 Table S11A).

#### 3.6.4 Physical Function: Endurance and Aerobic Capacity

At the type level, NUT+EX (moderate CoE), NUT+EX+BCI (low CoE), and EX (very low CoE) may increase VO_2_max (mL/kg/min) compared to control; however, the 95% CI included the null effect (Figure 1E, Supporting information 1 Tables S8B and S9E). At the modality level, similar results were seen for AE, AE+RE, and CR+AE+RE+BCI relative to control (Supporting information 1 Figure S4E, Supporting information 1 Table S10E). For all other comparisons, results were very imprecise.

CNMA on treatment modality showed increases for AE, CR+AE+RE+BCI, and CR+AE+BCI compared to control (Supporting information 1 Figures S9A and B). Results for five RCTs not included in NMA largely support these findings (Supporting information 2 Table S1). In nine RCTs, lifestyle interventions, especially those including solely AE or CR+AE, showed improvements in the 6‐min walk test (Supporting information 2 Table S11A).

#### 3.6.5 Physical Function: Physical Function Measured by Other Tests

No NMA was conducted for few RCTs reporting on other functional tests. Mixed results were demonstrated for tests of balance and coordination with indications for better results in interventions with EX components, especially when RE and AE were combined (Supporting information 2 Table S11A). No effects were found in the few studies investigating flexibility as an outcome (Supporting information 2 Table S11A). Self‐reported function was improved by interventions including EX, more so when RE+AE were combined (Supporting information 2 Table S11A).

#### 3.6.6 Body Composition: Fat‐Free Mass (FFM)

NMA on treatment type (very low–moderate CoE) and modality identified that no intervention may reduce FFM (Figure 1F, Supporting information 1 Tables S8C and S9F, Supporting information 1 Figure S4F, Supporting information 1 Table S10F).

Results of CNMA showed similar but more precise effects (Supporting information 1 Figures S10A and B). Further results of included RCTs on other muscle measurements (e.g., muscle quality) not covered by our NMA are displayed in Supporting information 2 Table S11B.

#### 3.6.7 Body Composition: Fat Mass

NMA on absolute FM showed that all intervention types except NUT only and EX only may reduce FM compared to control, with the largest effects for NUT+EX+BCI (MD −6.58 kg [95%CI −10.44; −2.73]) and NUT+BCI (MD −6.14 kg [95%CI −11.50; −0.78]), although the evidence remains mainly very uncertain (CoE very low–low, Figure 1G, Supporting information 1 Table S9G).

With the exception for the comparison of NUT+EX+BCI vs. BCI alone and EX alone with higher effects for NUT+EX+BCI, there may be no differences between intervention types (very low–low CoE; Supporting information 1 Table S8D). In NMA on treatment modality, FM was reduced by CR+AE+RE+BCI and BCI compared to control (Supporting information 1 Figure S4G). We did not find differences between other intervention modalities, except for a greater reduction of FM by CR+AE+RE+BCI compared to BCI alone, AE alone or HP alone (Supporting information 1 Table S10G). CNMA on treatment type demonstrated reductions for all but EX only interventions (Supporting information 1 Figure S11A), CNMA on treatment modality revealed reductions only for those interventions containing CR or BCI (Supporting information 1 Figure S11B). RCTs not included showed results that are in line with those from NMA (Supporting information 2 Table S11C).

#### 3.6.8 Body Composition: Waist‐Circumference/Fat Tissue

Neither NMA on treatment type nor modality showed differences between interventions or control (Figure 1H, Supporting information 1 Figure S4H, Supporting information 1 Tables S9H, S10H).

Results of the RCTs not included in NMA are reported in Supporting information 2 Table S11C. CNMA revealed small reductions of waist circumference for NUT+EX+BCI, NUT+EX, or NUT+BCI (type) and for CR+AE+RE and CR+BCI (modality) compared to control (Supporting information 1 Figure S12).

We included 18 trials on visceral adipose tissue (VAT) or visceral fat area (VFA) and 12 trials for subcutaneous adipose tissue (SAT). Due to different assessment methods and units, we did not run NMA. Multimodal interventions including a CR component found improvements in both measures, while NUT and EX alone brought up mixed results compared to control (Supporting information 2 Table S11C).

#### 3.6.9 Body Composition: Body Mass

For treatment type, body mass may be reduced by all intervention types except solely NUT (MD −3.87 kg [95%CI −7.99; 0.24]) or EX (MD −1.47 kg [95%CI −4.22; 1.28]) compared to control (both very low CoE) (Figure 1I, Supporting information 1 Table S8E and S9I).

Reductions may be highest for NUT+EX+BCI (MD −8.69 [95%CI −13.14; −4.25], very low CoE), NUT+BCI (MD −6.37 [95%CI −7.60; −5.14], moderate CoE), EX+BCI (MD −5.89 [95%CI −10.75; −1.03], very low CoE) and NUT+EX (MD −5.69 [95%CI −10.64; −0.73], very low CoE) compared to control. A difference between intervention types was also demonstrated for NUT+EX+BCI versus EX+BCI, EX, and BCI as well as for NUT+BCI versus EX and BCI. Differences are shown in Supporting information 1 Tables S8E and S9I. NMA on treatment modality showed lower body mass for CR+AE+BCI, CR+AE+RE+BCI, CR+RE+BCI, CR+HP+BCI, and BCI when compared to control (Supporting information 1 Figure S4I, Supporting information 1 Table S10I). Differences between intervention modalities are displayed in Supporting information 1 Table S10l. CNMA on treatment type found similar effects but with narrower 95%CIs and weight reductions for all intervention types except EX only (Supporting information Figure S13A). Due to narrower 95%CIs, CNMA revealed effects for CR, CR+AE, CR+AE+RE, CR+BCI, CR+HP, CR+HP+AE, CR+HP+AE+RE, and CR+HP+RE when compared to control (Supporting information 1 Figure S13B).

#### 3.6.10 Body Composition: Bone Mineral Density

NMA and CNMA of total hip BMD may show no differences—neither on treatment type (low to very low CoE) (Figure 1J and Supporting information 1 S14A, Supporting information 1 Tables S8F and S9J) nor treatment modality (Supporting information 1 Figures S4J and S14B, Supporting information 1 Table S10J).

Regarding results on other BMD sites, most comparisons revealed no differences (Supporting information 2 Table S11D).

#### 3.6.11 Other Outcomes: Quality of Life, Psychological Outcomes, and Social Participation

Sixteen RCTs reported outcome data for quality of life. Due to the use of various instruments, we did not run NMA. Descriptive results show that lifestyle interventions did not negatively affect any measurement or domain of (health‐related) quality of life (Supporting information 2 Table S11E) with most interventions demonstrating no between‐group differences.

Very few RCTs reported results on psychological health (e.g., depression), indicating no differences between groups (Supporting information 2 Table S11F). No RCT reported results on social participation.

#### 3.6.12 Other Outcomes: Health‐Related Event Data

Six RCTs reported on risks for health‐related events (Supporting information 2 Table S11G). Three trials used the metabolic syndrome risk, with CR+AE+RE+BCI demonstrating a reduced relative risk compared to the control group, while no differences were found comparing a high glycemic index diet + AE vs. a low glycemic index diet + AE. The risk for mobility disability was reduced by AE+RE versus control [58]. No differences were identified between CR+HP+AE+RE compared to control on frailty and fall risk [79]. One RCT did not find differences in the post‐intervention risk for sarcopenia, obesity, and sarcopenic obesity when comparing HP+RE versus RE in people with sarcopenic obesity [65].

#### 3.6.13 Adverse Events

Forty‐two of 72 included RCTs did not inform about adverse events (Supporting information 1 Table S12). Most of the reported adverse events were classified as (1) non‐severe (e.g., pain or constipation) or (2) unrelated to the intervention, but definitions of severity varied. When reported, serious intervention‐related adverse events were rare (e.g., musculoskeletal injuries).

## 4 Discussion

This work investigated the effects of lifestyle interventions in older people with obesity based on 72 RCTs with 6617 participants. We found high certainty evidence that NUT+EX+BCI interventions improve physical functioning, as measured by performance batteries, compared to control. The combination of NUT+EX+BCI may also reduce body mass and FM and may not negatively affect FFM or BMD; however, the CoE is very low. Other intervention types (single or two‐component combinations), such as NUT+EX, may also be effective, although their estimated effects tended to be smaller. For psychosocial outcomes, quality of life, and health events, the available data were insufficient or too heterogeneous to derive clear results. When interpreting these findings, it should be noted that imprecision and high risk of bias in several RCTs reduced the CoE. Importantly, few RCTs included participants aged 75 years and older and/or with existing functional limitations. Taken together, these findings provide the basis for evaluating how individual intervention components contribute to the observed effects.

### 4.1 Relevance of Intervention Components

Our results indicate that NUT+EX+BCI may be the most favorable approach for older adults to improve health‐related outcomes, potentially due to its synergistic effects [87, 110]. For functional outcomes, EX appears to be the most relevant intervention component for older people with obesity. At the treatment type level, our NMA indicated improvements in physical performance batteries for all interventions that included an EX component (CoE: high–low) except for NUT+EX (CoE: very low) when compared to control. The NUT+EX node, however, consisted of only three RCTs with different EX modalities, i.e., CR+AE and CR+RE, and may have been affected by ceiling effects, as two of these RCTs reported high baseline SPPB scores [111, 112]. Potential ceiling effects, which are common in geriatric research [112], might also be relevant for other outcomes assessing physical function, as only 12 RCTs recruited participants with objectively measured functional impairments [59, 60, 61, 63, 74, 85, 86, 87, 95, 96, 100, 106]. For interventions focusing only on EX components without CR, modality level findings demonstrated clinically important effects for AE+RE, as well as RE or AE alone. This pattern aligns with the principle of diminishing returns, whereby AE may be sufficient to induce functional improvements in untrained and unfit older individuals [113]. Over time, however, the principle of task specificity may become more relevant, where effects are mainly driven by RE, increasing maximum strength, power, and balance, and hence improve functional abilities, such as standing up quickly [114]. This is supported by our findings for chair rise performance, which improved following EX‐only interventions at the type level and RE‐only interventions at the modality level. Our NMA on gait speed did not identify differences between intervention groups—potentially caused by low power and the application of different gait protocols [115]. An individual participant data meta‐analysis of eight RCTs from a single center also found the greatest improvements in gait speed with CR+EX compared with CR or EX alone [116].

Our NMA showed no improvements in handgrip strength, and all estimated effects were below the threshold of clinical relevance (6 kg) [112]. Apart from most studies recruiting participants without frailty, these findings might be explained by the fact that RE interventions primarily targeted lower extremity strength and function.

The measurement of aerobic capacity by the 6‐min‐walk‐test represents an important marker for assessing functional endurance in daily life and cardiovascular health. Our descriptive synthesis suggests that interventions including an aerobic component (e.g., AE alone or CR+AE) may improve 6‐min walk performance. Maximal (VO_2_max) and submaximal (VO_2_peak) oxygen uptake—commonly used indicators of cardiovascular fitness—decline with age and may modify the association between obesity and mortality [118, 119]. Previous findings suggest that improvements in EX capacity of 8–10% are associated with a 12% decrease in mortality [119]. Our NMA indicated that interventions including AE may improve VO_2_max meaningfully (> 10%) relative to control; however, the (very) low certainty of evidence in our analyses precludes firm conclusions of these findings.

It is well known that CR is particularly effective for loss of body mass [120]. Consistent with this, our NMAs at the type and modality level generally support CR‐induced weight loss in older adults. Interestingly, interventions involving NUT alone did not result in reductions in body mass at the type level (CoE: very low), although all of them applied CR. A more thorough examination of these four RCTs reveals that one large RCT implemented only a slight CR [90], in contrast to the moderate CR implemented in other trials. In two of the four RCTs, the control groups also lost more than 2.5 kg; the overall weight reductions in the intervention groups were modest (approximately 4–7 kg over 13–52 weeks) [44, 51], and adherence was not reported in three of the four trials, which may partly explain why CR alone did not yield clearer effects in these studies [44, 51, 91].

Our results further suggest that interventions incorporating CR reduce FM, whereas their effects on WC did not differ from control. Apart from low statistical power, also indicated by the positive findings of the CNMA, the lack of effect on WC may be related to larger measurement error in individuals with higher BMI [122, 123, 124]. Regarding reductions in VAT, several studies including at least one EX modality, CR or their combination, reported positive effects [44, 46, 54, 56, 58, 85, 87], consistent with findings in younger adults [124]. A dose–response relationship between EX and VAT observed in younger individuals suggests that the EX dose in some trials may have been insufficient to elicit meaningful changes [125, 126]. Furthermore, three studies demonstrated superior effects of CR+EX compared to EX alone, underscoring the advantages of a multimodal approach [58, 67, 87].

As WL interventions have been described to come at the expenses of muscle mass and BMD, adding EX is considered an important strategy to counteract these losses [86]. According to our NMA, no intervention type or modality appeared to reduce FFM compared to control, but evidence is mainly very uncertain. Previous research in adults indicates that AE leads to reduced FM while maintaining FFM, whereas RE increases FFM [126]. With regard to weight regain following weight management interventions, evidence in postmenopausal women suggests that gains in FM exceed gains in muscle mass [127]. This highlights the need for improving adherence to a healthy lifestyle, also to prevent sarcopenia. A further component of interest is protein intake, which has been proposed to mitigate loss of lean mass during CR. Our NMA findings suggest that interventions with a HP component, either as the only dietary component or combined with CR, did not affect FFM, and moreover physical function and strength compared to controls. These findings partially contrast results from two RCTs in older people with obesity that compared WL with or without a HP diet and reported improvements in FFM or physical function [66, 74]. Both studies were only included in our descriptive analyses due to missing network connectivity. For our work, a HP diet was defined as ≥ 1.2 g/kg BW/day. A meta‐analysis in adults aged ≥ 50 years found that adding HP (≥ 25% of energy intake or ≥ 1 g/kg BW/day) to CR increased lean mass retention and enhanced FM loss [126]. There is an ongoing debate about the optimal amount of protein as well as the reference measure, e.g., ideal, adjusted, or current body weight, during weight reduction, which might explain the partially conflicting results [128].

Our results do not indicate negative effects on BMD (CoE: low to very low). Due to heterogeneous assessment methods and units, we could only include four trials with 420 participants in the NMA of total hip BMD, which resulted in two separate networks. Interestingly, two trials included in the NMA reported improvements compared to control, whereas the pooled estimates did not [85, 87]. This discrepancy may reflect differences in the direction of change observed in the respective control groups (increase vs. decrease). One trial reported an increase in total hip BMD with AE+RE compared to control, and an attenuated decrease when AE+RE was added to CR+BCI compared to CR+BCI alone. The same two RCTs also examined whole body or lumbar spine BMD and found no intervention effects. These findings are consistent with evidence that WL, especially if diet‐induced, affects total hip BMD before other skeletal sites [129]. Nevertheless, potential negative effects at other bone regions should not be neglected. For example, lumbar spine BMD may decrease in postmenopausal women with obesity undergoing weight‐loss interventions without exercise [130]. Meta‐analyses in other populations, e.g., postmenopausal community‐dwelling women, suggest that EX can improve BMD [131], which has yet to be confirmed in older adults with obesity participating in WL interventions.

Our findings also align with and extend previous evidence syntheses in adjacent populations. A NMA in adults of retirement age with overweight and obesity evaluated lifestyle interventions (NUT, EX and combinations) and summarized effects on BMI, waist circumference, FM and FFM [23]. Although that review applied different inclusion criteria, such as age range (55–70 years), weight status (overweight and obesity), and intervention duration (6 weeks–6 months), and did not consider BCI as a distinct intervention component in their NMA, its results for body composition were broadly similar. However, that work did not evaluate the CoE and did not examine additional outcomes relevant to older adults, such as different measures of FM, functional status, or quality of life.

Given the central role of behavior in sustaining long‐term lifestyle changes, the contribution of BCI deserves particular attention. In our review, BCI was considered as a distinct intervention component that may facilitate the implementation of successful WL and EX strategies during the study period and moreover, might support the long‐term maintenance, as shown previously in younger adults [25]. Our analyses provide some support for the relevance of BCI in older people, as interventions including NUT+EX+BCI tended to show the largest effects, although not always statistically different from other approaches. For functional status measured by performance batteries, we found moderate CoE that the addition of BCI may be more effective than NUT+EX only. Unfortunately, there is no standardized definition of BCI, and corresponding interventions are often poorly described [133, 134], as was the case for most RCTs in our review. As a consequence, we were unable to evaluate the influence of BCI duration or intensity. Although we contacted authors of all included RCTs for additional information when reporting was incomplete, details on BCI components often remained too limited, allowing inclusion of this component in only 27 of 72 RCTs (37.5%). Among these RCTs, we were not able to identify any specific BCI technique or modality that might be superior to others. Evidence from younger populations suggests that BCI components in obesity management interventions may enhance adherence [134]. Our observation that interventions in RCTs are often inadequately reported is in line with previous research [135].

For other predefined outcomes, such as health events, quality of life, or psychological outcomes, conflicting findings or the scarcity of data does not allow any conclusions. Social participation was not reported in any of the included studies. Future RCTs should investigate effects on these outcomes as well as on others, such as pain and medication use, given their importance for older people with obesity [136].

Our synthesis also reveals poor and highly heterogeneous reporting of adverse events, highlighting the need for methodological improvements [137]. Future trials should report adverse events according to structured reporting standards such as CONSORT to allow more robust conclusions regarding potential harms of lifestyle interventions in older people with obesity [137].

### 4.2 Limitations

We acknowledge several limitations, making it necessary to interpret our results with caution. First, as for obesity (BMI, percent body fat, and WC), the use of different diagnostic criteria for sarcopenic obesity might have contributed to heterogeneity in the included patient populations [12, 139, 140]. Although a first consensus definition for sarcopenic obesity was published in 2022, we relied on the criteria reported in the primary studies [140]. Second, even though we conducted NMAs for intervention type and modality, heterogeneous intervention characteristics, e.g., differences in length of intervention, intensity, mode of administration may have introduced residual confounding. For the node definitions of the control groups, we considered passive controls as well as minimal interventions. A previous systematic review showed that digital self‐monitoring of diet and/or physical activity can affect WL [141], suggesting that differences between intervention and control groups may have been underestimated in some networks. Third, our results likely often lack power to detect differences in effects between various interventions and controls due to nodes with small numbers of RCTs and participants. This may have resulted in imprecise effect estimates or even implausible results. A more granular analysis of relevant intervention characteristics—such as specific dietary regimens, macronutrient compositions, or EX intensities—was not feasible based on available data. Fourth, high heterogeneity in outcome assessments and measurement units [135] further reduced the number of RCTs included in NMA. Finally, our review exclusively focused on lifestyle‐based interventions without additional co‐interventions, such as pharmacological therapy.

### 4.3 Implications for Practice

Our results may be used to inform evidence‐based clinical practice guidelines. Based on our findings, combined NUT+EX+BCI interventions appear most promising to manage obesity in community‐dwelling older people. Evidence remains limited for individuals aged > 75 years or those at high risk of mobility disability and loss of independence, which needs to be regarded when making treatment decisions. It is also important to consider perceptions, motives, and barriers of participants to enhance motivation and adherence to offer a patient‐centered care [143, 144, 145]. People who lack the motivation to change their dietary behavior, isolated EX interventions, or EX interventions combined with BCI may serve as alternatives to improve physical function and cardio‐metabolic health.

### 4.4 Implications for Research

Future trials should be conducted in geriatric samples (aged ≥ 75 years, pre‐existing functional limitations, with comorbidities) to investigate the efficacy of lifestyle interventions in this growing but underrepresented group. For adults aged ≥ 65 years, the evidence from the current analysis is insufficient to derive firm conclusions regarding the additional effect of a HP diet component or BCI modalities in WL interventions, highlighting the need for further investigation. Given the substantial heterogeneity in outcome variables currently used, standardization of outcomes and their assessment procedures is urgently needed [136]. Greater emphasis should also be placed on patient‐reported outcomes, such as quality of life or social participation due to their importance to those affected. Finally, trials with long‐term follow‐up are needed to evaluate the sustained efficacy and effectiveness of lifestyle interventions.

## 5 Conclusions

This systematic review with NMA suggests that lifestyle interventions combining nutrition, i.e., calorie restriction, with exercise, i.e., aerobic and/or resistance exercise, and behavior change intervention components improve physical functioning in older people with obesity, an outcome of particular relevance for maintaining independence in this age group. The combination of these interventions may also reduce body mass and fat mass without negatively affecting fat‐free mass or bone mineral density, while demonstrating low rates of mostly mild adverse events. However, many of the estimated treatment effects were based on low or very low certainty evidence due to risk of bias and imprecision. We also identified important evidence gaps, including scarcity of data on (1) patient‐relevant outcomes (quality of life, social participation), (2) specific intervention components (nutrition with high protein, behavior change intervention), (3) people with obesity aged ≥ 75 years or those with existing functional limitations, and (4) long‐term follow‐up. These findings highlight the need for further high‐quality RCTs to strengthen the evidence base for managing obesity in older adults.

## Author Contributions

The authors confirm contribution to the paper as follows: G.T., D.S., and E.K. planned and designed the project. G.T. and H.K. designed the search strategy. G.T., D.S., I.G.E., and E.K. screened titles/abstracts and/or full texts. G.T., D.S., I.G.E., and E.K. extracted the data. G.T. and I.G.E. assessed the risk of bias and G.T., D.S., and E.K. the certainty of evidence (GRADE). G.T. undertook network meta‐analyses. G.R. supervised conducting network meta‐analyses. G.T. wrote the first draft of the manuscript. D.S. and E.K. revised the manuscript. L.S., G.R., H.K., W.K., C.C.S., J.A.B., D.T.V., N.S.B., and D.V. were involved in the planning and design process of this project, providing critical feedback for the manuscript. All authors approved the final version.

## Funding

This work was funded by the German Federal Ministry of Education and Research (BMBF) grant number 01KG1903.

## Conflicts of Interest

GT, DS, IGE, LS, GR, HK, WK, CCS, NSB, DV, and EK have no conflicts of interest to declare. JAB has no conflicts of interest related to the submitted manuscript. Outside the submitted work, JB has received support: R01‐AG‐077163, NIH‐K23‐AG‐051681, grants: K23‐AG‐051681, R01‐AG‐067416, R41‐AG‐071290, R01‐AG‐058615, U01‐AG‐071450, 1UG1HD107692, R56 AG089080‐01, P30 DK056350, payment or honoraria for lectures or presentations: Obesity Medicine Association, Wake Forest Baptist Health, Hong Kong Research Grants Council, NIH Reviewer Payment, Columbia University. Patents planned, issued, or pending: No. 62/672,827, 17/335,986 (#11623114). Participation on a Data Safety Monitoring Board or Advisory Board: Nutrition for precision health, R01DK133509 and for multiple NIA clinical trials for his participation on a data safety monitoring board. No other financial or non‐financial interest: Equity in SynchroHealth LLC, remote monitoring company. DTV has no conflicts of interest related to the submitted manuscript. Outside the submitted work, DTV has received grants RO1‐DK109950 and CX002161 and payments from the Clinical Trials Advisory Panel to the NIH‐National Institute on Aging and the DSMB for multiple NIA clinical trials for his participation on a data safety monitoring board or advisory board. He sits on the scientific advisory board for Biophytis SA.

## Supporting information


**Figure S1:** PRISMA 2020 flow diagram for new systematic reviews which included searches of databases, registers, and other sources.
**Figure S2:** Risk of bias assessment for objective outcomes.
**Figure S3:** Risk of bias assessment for subjective outcomes.
**Figure S4:** Forest plots and network graphs of network meta‐analyses of different intervention modalities.
**Figure S5:** Forest plot of component and standard network meta‐analyses of different intervention types (A) and modalities (B) on functional status measured by performance batteries.
**Figure S6:** Forest plots of component and standard network meta‐analyses of different intervention types (A) and modalities (B) on functional status measured by gait speed.
**Figure S7:** Forest plot of component and standard network meta‐analyses of different intervention types (A) and modalities (B) on functional status measured by chair rise test.
**Figure S8:** Forest plot of component and standard network meta‐analyses of different intervention types (A) and modalities (B) on functional status measured by hand‐grip strength.
**Figure S9:** Forest plot of component and standard network meta‐analyses of different intervention types (A) and modalities (B) on functional status measured by VO2‐max.
**Figure S10:** Forest plot of component and standard network meta‐analyses of different intervention types (A) and modalities (B) on fat‐free mass (kg).
**Figure S11:** Forest plot of component and standard network meta‐analyses of different intervention types (A) and modalities (B) on fat mass (kg).
**Figure S12:** Forest plot of component and standard network meta‐analyses of different intervention types (A) and modalities (B) on waist circumference (cm).
**Figure S13:** Forest plot of component and standard network meta‐analyses of different intervention types (A) and modalities (B) on body mass (kg).
**Figure S14:** Forest plot of component and standard network meta‐analyses of different intervention types (A) and modalities (B) on bone mineral density of the total hip.
**Figure S15:** Funnel plot for physical performance batteries (NMA on intervention type).
**Figure S16:** Funnel plot for gait speed (NMA on intervention type).
**Figure S17:** Funnel plot for chair rise ratio (NMA on intervention type).
**Figure S18:** Funnel plot for hand‐grip strength (NMA on intervention type).
**Figure S19:** Funnel plot for VO2‐max (NMA on intervention type).
**Figure S20:** Funnel plot for fat‐free mass (NMA on intervention type).
**Figure S21:** Funnel plot for fat mass (NMA on intervention type).
**Figure S22:** Funnel plot for waist‐circumference (NMA on intervention type).
**Figure S23:** Funnel plot for body weight (NMA on intervention type).
**Figure S24:** Funnel plot for bone mineral density of the total hip (NMA on intervention type).
**Table S1:** (A–F) Search strategies.
**Table S2:** Special rules for the assessment of risk of bias.
**Table S3:** GRADE used thresholds for small, medium and large effects.
**Table S4:** Excluded references from full‐text screening.
**Table S5:** Included studies and reports.
**Table S6:** Detailed characteristics of included studies (design, participants, interventions, outcomes, funding/COI).
**Table S7:** Detailed description of intervention characteristics.
**Table S8:** GRADE evaluation for network meta‐analyses.
**Table S9:** League tables—results of pairwise and network meta‐analyses of different intervention types (mean or standardized mean differences with 95% confidence intervals).
**Table S10:** League tables—results of pairwise and network meta‐analyses of different intervention modalities (mean or standardized mean differences with 95% confidence intervals).
**Table S11:** (A–H) Descriptive results on the effects of lifestyle interventions on physical function (A), body composition and anthropometry measures related to muscle mass (B), body fat (C), and bone (D), quality of life (E), psychological outcomes (F), health events (G), and social participation (H).
**Table S12:** Adverse events reported in included randomized controlled trials (n = 54).
**Table S13:** References of reports for which requested information was requested.

## Data Availability

The data that support the findings of this study are available from the corresponding author upon reasonable request.
