# Supplementary material for: EffectS of Lifestyle Interventions in Older PEople With Obesity (Effective SLOPE): a Systematic Review With Network Meta‐Analyses
Source: Obes Rev. 2026 Apr 3;27(10):e70123. doi: 10.1111/obr.70123 (PMC13551408; doi:10.1111/obr.70123)
Supplement: Supplementary file 1 [file OBR-27-e70123-s001.pdf]

Supporting information 1 + 2

EffectS of Lifestyle interventions in Older PEople with obesity (Effective SLOPE): a systematic review with network meta-analyses

Gabriel Torbahn[1,2,3,4], Daniel Schoene[5,6,7], Isabel Galicia Ernst[1], Lukas Schwingshackl[8], Gerta Rücker[9], Helge Knüttel[10], Wolfgang Kemmler[5], Cornel C Sieber[1,11], John A Batsis[12,13], Dennis T Villareal[14], Nanette Stroebele-Benschop[15], Dorothee Volkert[1], Eva Kiesswetter[1,8]

[1]Institute for Biomedicine of Aging, Friedrich-Alexander-Universität Erlangen-Nürnberg, Nürnberg, Bayern, Germany
[2]Department of Pediatrics, Paracelsus Medical University, Nuremberg, Germany
[3]Department of Pediatrics, Paracelsus Medical University, Salzburg, Austria
[4]Obesity Research Unit, Paracelsus Medical University, Salzburg, Austria
[5]Institute of Radiology, University Hospital Erlangen, Erlangen, Germany
[6]Department of Clinical Gerontology, Robert Bosch Hospital, Stuttgart, Germany
[7]Institute of Epidemiology and Medical Biometry, Ulm University, Ulm, Germany
[8]Institute for Evidence in Medicine, Faculty of Medicine, Medical Center - University of Freiburg, Freiburg, Germany
[9]Institute of Medical Biometry and Statistics, Faculty of Medicine, Medical Center - University of Freiburg, Freiburg, Germany
[10]University Library, University of Regensburg, Regensburg, Germany
[11]Department of Medicine, Kantonsspital Winterthur, Winterthur, Zurich, Switzerland
[12]Division of Geriatric Medicine, School of Medicine, University of North Carolina at Chapel Hill, Chapel Hill, North Carolina, USA
[13]Department of Nutrition, Gillings School of Global Public Health, University of North Carolina at Chapel Hill, Chapel Hill, North Carolina, USA
[14]Division of Endocrinology, Diabetes and Metabolism, Baylor College of Medicine, Houston, Texas, USA
[15]Department of Nutritional Psychology, Institute of Nutritional Medicine, University of Hohenheim, Stuttgart, Germany

Address of corresponding author and e-mail address:
Gabriel Torbahn, MPH
Institute of Biomedicine of Aging
Friedrich-Alexander-Universität Erlangen-Nürnberg
Kobergerstr. 60
90408 Nuremberg, Germany
gabriel.torbahn@fau.de

Table of contents

Supporting information 1

Supplemental methods 4
References supplemental methods..12
Supplemental figures...14
Figure S1: PRISMA 2020 flow diagram for new systematic reviews which included searches of databases, registers and other sources 14
Figure S2: Risk of Bias assessment for objective outcomes 16
Figure S3: Risk of Bias assessment for subjective outcomes 17
Figure S4: Forest plots and network graphs of network meta-analyses of different intervention modalities 18
Figures S5-S14: Forest plots of component and standard network meta-analyses 28
Figures S15-S24: Funnel plots..39
Supplemental tables...44
Table S1 A-F: Search Strategies 44
Table S2: Special rules for the assessment of risk of bias 78
Table S3: GRADE used thresholds for small, medium and large effects 80
Table S4: Excluded references from full-text screening 81
Table S5: Included studies and reports 82
Table S6: Detailed Characteristics of included studies (design, participants, interventions, outcomes, funding/COI) 96
Table S7: Detailed Description of intervention characteristics 97
Tables S8: GRADE evaluation for network meta-analyses 98
Tables S9: League tables - results of pairwise and network meta-analyses of different intervention types (mean or standardized mean differences with 95% confidence intervals)..104
Tables S9: League tables - results of pairwise and network meta-analyses of different intervention modalities (mean or standardized mean differences with 95% confidence intervals) 114
Table S11 A-H: Descriptive results on the effects of lifestyle interventions on physical function (A), body composition and anthropometry measures related to muscle mass (B), body fat (C), and bone (D), quality of life (E), psychological outcomes (F), health events (G), and social participation (H) 124
Table S12: Adverse events reported in included randomized controlled trials (n=54)...125
Table S13: References of reports for which requested information was requested 128
Excluded References 129

Supporting information 2

To view supporting information 2, please download the file on OSF:
https://osf.io/ayhq3?view_only=6e987edd80924f35b689f2b50ee3749b

Supplemental methods

Systematic literature search

We searched six databases (Medline (via Ovid), Embase (via Ovid), Cochrane Register of Clinical Trials (CENTRAL, via Cochrane Library), Cumulated Index to Nursing and Allied Health Literature (CINAHL, via EBSCOhost), PsycINFO (via EBSCOhost), and Science Citation Index Expanded [SCI-EXPANDED] (via Web of Science Core Collection/Clarivate)) and one trial registry (ClinicalTrials.gov) from inception until May 2022 without language restrictions. As we searched ClinicalTrials.gov for ongoing trials and were limited in time resources, we did not consider trial entries from CENTRAL (table S1). We updated the search in May 2021 and May 2022 by re-running the full searches. In October 2025, we undertook a Medline-only search to capture the include evidence published since the last search date. We removed previous records based on their database accession numbers. We identified duplicates using the technique for Endnote by Bramer et al. followed by a duplicate check in Covidence [1]. We exported search results from ClinicalTrials.gov as CSV-file and screened according to our prespecified eligibility criteria. The search strategy contained a combination of keywords and database-specific thesaurus vocabulary. In October 2025, we checked the status of the ongoing studies captured by the primary search that have been identified in ClinicalTrials.gov. In addition, we used the Cochrane sensitivity-precision maximizing search filter for RCTs [2]. We adapted search engine-specific vocabulary to all searched databases. Based on a search strategy of a Cochrane review on obesity therapy in adolescents, a reviewer (GT) developed the search strategies together with a search specialist (HK) [3]. In addition, we sent the search strategies and the planned search procedure to an external information specialist (EM, University of Freiburg, Germany) who was not further involved in the project for peer-reviewing purposes.

Eligibility criteria

According to the PICOS-frame, we included studies based on the following criteria [4]:

Patients: Older adults (age at inclusion ≥60 years and mean age of ≥65 years) with obesity or sarcopenic obesity. As a body mass index (BMI) of ≥30 kg/m^2 is widely used in clinical practice but serves only as rough criterion for obesity especially in the older population, we additionally included RCTs that used percentage body fat (PBF) of ≥35% for women and ≥25% men, or waist circumference of ≥88 cm for women and ≥102 cm for men as obesity criterion [5-7]. Due to differences in body composition between Caucasians and Asians, we used cut-offs proposed by the WHO for RCTs conducted in the Asian-Pacific region [8, 9]. Sarcopenia was defined based on the criteria used by the primary RCTs.

Interventions: The respective intervention must have lasted for a period of ≥12 weeks with regular meetings. We pre-defined three major intervention types: NUT, EX and behavior change intervention (BCI), which are further described by their treatment modalities. Nutritional interventions needed to affect energy balance. Therefore, we did not consider interventions which might affect weight-loss via other mechanisms, such as extracts, micronutrients, or components of macronutrients. We included EX interventions that intended a planned, structured, and repetitive movement, as well as electromyostimulation (EMS) training if this was used as a supportive element during EX classes [10]. We included BCIs which followed the classical approach to behavioral therapy (e.g., social cognitive theory, trans-theoretical model), including motivational interviewing, goal setting, self-monitoring, problem-solving strategies, etc., under supervision by a professional particularly trained in this treatment approach. We did not

consider interventions that only used self-monitoring within counselling sessions with study personnel (e.g., dietician).

Control conditions: As we undertook NMA, all interventions were compared to each other, and to active (health education (e.g., minimal interventions providing written material or group sessions) and passive control conditions (e.g., waiting list, care as usual).

Outcomes: We included effects on functional status (primary outcome, measured by performance batteries or single tests), body weight, body composition, quality of life, psycho-social outcomes, and therapy-related adverse events.

Study type: We only included randomized controlled trials (RCT).

We did not include conference abstracts, publications that have not been peer-reviewed or doctoral theses that have not been published as peer-reviewed journal articles.

Screening process

After piloting the screening process, we did the eligibility screening of titles/abstracts and full texts in duplicate and independently (IGE, EK, DS, or GT). If agreements could not be reached, we consulted a third reviewer not involved in the screening of the respective record (EK, DS, or GT).

Assessment of risk of bias

For individually randomized, parallel-group clinical trials, we used the current version of the Cochrane Risk of Bias tool 2.0 (RoB 2.0) and for cluster-randomized clinical trials the updated test version for cluster RCTs (https://www.riskofbias.info/welcome/rob-2-0-tool) [11]. After piloting the use of the tool, rules for the assessment of RoB were defined in strong reference to the guidance of the Cochrane RoB 2.0 tool (Additional file 1 Table S2).

Two reviewers (GT and IGE) independently rated the RoB and judged each domain as well as the overall RoB as low RoB, some concerns or high RoB. We were mainly interested in the effects of assignment to intervention. To further judge the RoB due to adherence to an intervention, we added an extra signalling question to the second domain. We assessed publication bias by a search for non-published RCTs in clinicaltrials.gov, by visual inspection of comparison-adjusted funnel plots and by Eggers' linear regression tests [12].

Data extraction

Data was extracted based on a piloted form by two reviewers (GT, DS, IGE, or EK), independently. Conflicts were solved by discussion or a third reviewer (GT, DS, or EK). We extracted study (e.g., year, country), intervention (e.g., duration, type) and participants` baseline (e.g., age, sex) characteristics of all included studies. For all outcomes analysed by NMA, we further extracted post- or change scores as well as measures of dispersion (e.g., standard deviation (SD), standard error (SE), 95-% confidence intervals (CI)). If other measures of dispersion and no SD were reported, we calculated SDs according to the formulas in the Cochrane handbook.[13] If 95%-CI, SE or SD were reported as 0 without decimal points, we assumed the most conservative value that corresponds rounded to the value 0. If relevant but not reported, change scores were calculated by the formula in the Cochrane handbook.[13] For the imputation of the SD, we used a correlation coefficient (corr) of corr=0.5. For trials reporting respective data only in figures, we extracted relevant data via the online application 'Web plot digitizer' (https://automeris.io/WebPlotDigitizer/). In this case, data was extracted by one reviewer (GT, DS, or IGE) and checked by second reviewer (GT, DS, or IGE).

If not reported, we contacted authors of primary studies to clarify whether included references reported data on the same RCT, to obtain information on data for obesity subgroups of those trials enrolling older people with overweight and obesity and for relevant missing data (see supplement table S13). If authors did not respond on our first request, we resent our e-mail after two weeks. If authors did not respond for the second time or declined the request, we excluded the respective record.

Statistical analyses

After discussion with the advisory board, we assumed that the intransitivity assumption was fulfilled [14]. We conducted random effects NMA using a frequentist approach [15]. For the majority of outcomes, we used the mean difference (MD) or standardized mean difference (SMD; see manuscript). For FFM, we considered data for lean (body) mass, total non-bone/ mineral-free lean mass, lean soft tissue, and FFM. For the chair rise test, we pooled effects as a so called “chair rise rate ratio”. This test is available in two variants:

Variant 1:

The number of times a person stands up in a given time interval T is counted. This number (k), is Poisson distributed with expected value k and variance also k. The "Rise Rate" (number per time) is k/T with variance k/T^2. Here, large values are good. Unit is 1/sec.

Variant 2:

We wait until a person has stood up k times and document the time needed (T). Here, small values are good. Unit is sec.

Results of variant 2 were converted into those of variant 1. Let the number k be fixed. If we know the mean time (T_{mean}) that the subjects of a group needed for k attempts. Then

the mean "rise rate" is k/T_{mean} with variance $k/(n*T_{mean}^2)$. Thus, we can express the data from the study arms of both types in terms of "rise rates" and compare these rise rates as pooled rise rate ratios between groups. For pairwise meta-analysis, this is done using metainc (sm = "IRD" or "IRR") and for NMA, it is done using pairwise().

Given:

Variant 1: event = $n*k_{mean}$ (observed), time = n*T (fixed)

Variant 2: event = n*k (fixed), time = $T_{mean}*n$ (observed)

Example:

Variant 1: T = 30sec, k = 15 -> rate = 15/30sec = 0.5/sec

Variant 2: k = 5, T = 10sec -> rate = 5/10sec = 0.5/sec

We defined network nodes on two different levels: on the first level, we selected the control and intervention types NUT, EX, BCI or any combination of these interventions as nodes. To consider further information on treatment modality, we subdivided NUT interventions in CR and high protein (HP), EX interventions in aerobic exercise (AE) and RE.

Additionally, we ran component NMA (CMNA) which allow to disentangle the effects of single components in multicomponent treatment arms and to reconnect disconnected networks.[16] For all analyses we used R (R Core Team (2020). R: A language and environment for statistical computing. R Foundation for Statistical Computing, Vienna, Austria. URL https://www.R-project.org/) in R Studio (RStudio Team (2020). RStudio: Integrated Development Environment for R. RStudio, PBC, Boston, MA URL http://www.rstudio.com/). For NMA, we used the R package netmeta [17].

Assessment of the certainty of evidence

We assessed the certainty of evidence for performance batteries, aerobic capacity, body weight, FFM, FM as well as BMD of the total hip and followed the guidance of the Grading of Recommendations Assessment, Development and Evaluation (GRADE) approach for NMA, as proposed by the GRADE working group [18-21]. According to this approach, we firstly rated the direct evidence and – if necessary – rated down for RoB, inconsistency, or indirectness. We did not rate down for publication bias when assessing the certainty of evidence of the direct evidence as there have not been any direct comparisons with ≥10 studies [22]. Instead, we rated the overall publication bias as described above. If high certainty direct evidence was available and its contribution was >50%, we did not rate the indirect evidence. If this was not the case, we considered the lowest of two ratings of the direct evidence contributing to the first-order loop to rate the indirect evidence. To judge the evidence of the network estimate, we selected the rating of that evidence (direct or indirect) that contributed the most to the network estimate and – if necessary – rated further down for imprecision or incoherence. For the assessment of imprecision, we defined no, small, medium, and large effects for the respective outcomes (Table S3). For incoherence, we used the node splitting approach (p-value of the z-test of $p<0.05$) [23, 24]. The overall rating ranges from very low to high certainty.

Deviations from protocol

Contrary to the protocol, we only searched 1 trial registry as WHO ICTRP was not available during the COVID-19 pandemic.

As we only identified nine RCTs that specifically recruited participants with sarcopenic obesity and to the overall low number of RCTs for most of the outcomes we did not run subgroup analyses.

Due to disconnected networks for some outcomes, we selected the level of treatment type as primary and treatment modality as secondary analysis. Based on discussions with our clinical advisory board (JAB, DTV, WK, CCS, DV, NSB), we chose an additive model for CNMA. In addition to our extensive searches in six electronic databases in May 2022, our latest search update in July 2024 was in Medline only.

References supplemental methods

1 Bramer WM, Giustini D, de Jonge GB, Holland L, Bekhuis T. De-duplication of database search results for systematic reviews in EndNote. *J Med Libr Assoc*. 2016; 104: 240-3.
2 Lefebvre C, Glanville J, Briscoe S, Littlewood A, Marshall C, Metzendorf M-I*, et al.* Technical Supplement to Chapter 4: Searching for and selecting studies. In: Higgins JPT, Thomas J, Chandler J, Cumpston MS, Li T, Page MJ*, et al.* (eds.). *Cochrane Handbook for Systematic Reviews of Interventions Version 62 (updated February 2021)*. Cochrane 2021.
3 Mead E, Brown T, Rees K, Azevedo LB, Whittaker V, Jones D*, et al.* Diet, physical activity and behavioural interventions for the treatment of overweight or obese children from the age of 6 to 11 years. *Cochrane Database Syst Rev*. 2017; 6: Cd012651.
4 Akers J, Aguiar-Ibáñez R, Baba-Akbari A. Systematic reviews: CRD's guidance for undertaking reviews in health care: York, UK: Centre for Reviews and Dissemination, University of York 2009.
5 WHO consultation. Obesity: preventing and managing the global epidemic. Report of a WHO consultation. *World Health Organ Tech Rep Ser*. 2000; 894: i-xii, 1-253.
6 Durrer Schutz D, Busetto L, Dicker D, Farpour-Lambert N, Pryke R, Toplak H*, et al.* European Practical and Patient-Centred Guidelines for Adult Obesity Management in Primary Care. *Obes Facts*. 2019; 12: 40-66.
7 Gonzalez MC, Correia M, Heymsfield SB. A requiem for BMI in the clinical setting. *Curr Opin Clin Nutr Metab Care*. 2017; 20: 314-21.
8 Deurenberg P, Deurenberg-Yap M, Guricci S. Asians are different from Caucasians and from each other in their body mass index/body fat per cent relationship. *Obes Rev*. 2002; 3: 141-6.
9 World Health Organization. Regional Office for the Western P. The Asia-Pacific perspective : redefining obesity and its treatment: Sydney : Health Communications Australia 2000.
10 Chodzko-Zajko WJ, Proctor DN, Fiatarone Singh MA, Minson CT, Nigg CR, Salem GJ*, et al.* American College of Sports Medicine position stand. Exercise and physical activity for older adults. *Med Sci Sports Exerc*. 2009; 41: 1510-30.
11 Sterne JAC, Savović J, Page MJ, Elbers RG, Blencowe NS, Boutron I*, et al.* RoB 2: a revised tool for assessing risk of bias in randomised trials. *Bmj*. 2019; 366: l4898.
12 Chaimani A, Higgins JP, Mavridis D, Spyridonos P, Salanti G. Graphical tools for network meta-analysis in STATA. *PLoS One*. 2013; 8: e76654.
13 Higgins JPT, Li T, Deeks JJ (editors). Chapter 6: Choosing effect measures and computing estimates of effect. In: Higgins JPT, Thomas J, Chandler J, Cumpston M, Li T, Page MJ, Welch VA (editors). Cochrane Handbook for Systematic Reviews of Interventions version 6.2 (updated February 2021). Cochrane, 2021. Available from www.training.cochrane.org/handbook.
14 Salanti G. Indirect and mixed-treatment comparison, network, or multiple-treatments meta-analysis: many names, many benefits, many concerns for the next generation evidence synthesis tool. *Res Synth Methods*. 2012; 3: 80-97.
15 Rücker G. Network meta-analysis, electrical networks and graph theory. *Res Synth Methods*. 2012; 3: 312-24.
16 Rücker G, Petropoulou M, Schwarzer G. Network meta-analysis of multicomponent interventions. *Biom J*. 2020; 62: 808-21.

17 Rücker G, Krahn U, König J, Efthimiou O, Davies A, Papakonstantinou T*, et al.* netmeta: Network Meta-Analysis using Frequentist Methods. R package version 2.1-1.: 2022.
18 Brignardello-Petersen R, Bonner A, Alexander PE, Siemieniuk RA, Furukawa TA, Rochwerg B*, et al.* Advances in the GRADE approach to rate the certainty in estimates from a network meta-analysis. *J Clin Epidemiol*. 2018; 93: 36-44.
19 Brignardello-Petersen R, Guyatt GH, Mustafa RA, Chu DK, Hultcrantz M, Schünemann HJ*, et al.* GRADE guidelines 33: Addressing imprecision in a network meta-analysis. *J Clin Epidemiol*. 2021; 139: 49-56.
20 Brignardello-Petersen R, Mustafa RA, Siemieniuk RAC, Murad MH, Agoritsas T, Izcovich A*, et al.* GRADE approach to rate the certainty from a network meta-analysis: addressing incoherence. *J Clin Epidemiol*. 2019; 108: 77-85.
21 Puhan MA, Schünemann HJ, Murad MH, Li T, Brignardello-Petersen R, Singh JA*, et al.* A GRADE Working Group approach for rating the quality of treatment effect estimates from network meta-analysis. *Bmj*. 2014; 349: g5630.
22 Sterne JA, Sutton AJ, Ioannidis JP, Terrin N, Jones DR, Lau J*, et al.* Recommendations for examining and interpreting funnel plot asymmetry in meta-analyses of randomised controlled trials. *Bmj*. 2011; 343: d4002.
23 Dias S, Welton NJ, Caldwell DM, Ades AE. Checking consistency in mixed treatment comparison meta-analysis. *Stat Med*. 2010; 29: 932-44.
24 König J, Krahn U, Binder H. Visualizing the flow of evidence in network meta-analysis and characterizing mixed treatment comparisons. *Stat Med*. 2013; 32: 5414-29.

Supplemental figures

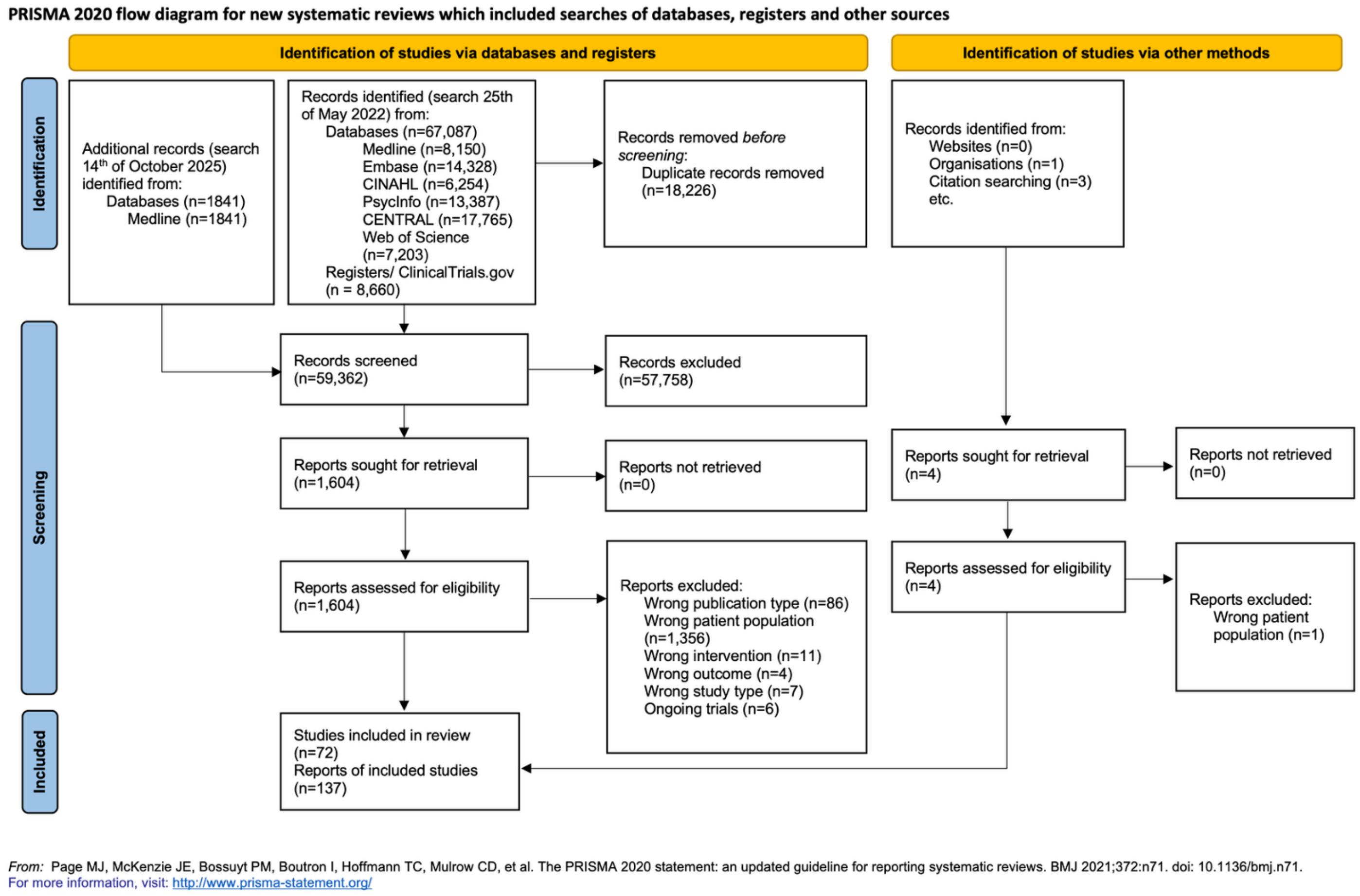

Figure S1: PRISMA 2020 flow diagram for new systematic reviews which included searches of databases, registers and other sources

Study ID	Experimental	Comparator	Outcome	D1	D2	D3	D4	D5	Overall
Abdelbasset 2020	Ex	Control	PF, BC	+	!	+	!	!	!
Amamou 2015	Nut + Ex	Nut	BC	!	!	+	+	!	!
Andonian 2023	Nut + Ex + BCI	Control	BC (BM)	!	+	+	+	+	!
Andonian 2023	Nut + Ex + BCI	Control	BF, BC	+	+	+	+	+	+
Ard 2017	Nut + Ex + BCI	Ex + BCI vs Ex + BCI	PF, BC	+	+	+	+	+	+
Balachandran 2014	Ex	Ex	PF, BC	-	-	+	+	!	-
Beavers 2015	Nut	Nut	PF, BC	!	!	+	+	!	!
Beavers 2019	Nut + BCI	Control	PF, BC	!	+	+	+	+	!
Bowen 2022	Control	Control	BC	!	+	+	+	!	!
Brennan 2020a	Nut + Ex	Nut vs Control	PF (VO2max), BC	!	+	-	+	+	-
Brennan 2020b	Nut + Ex	Nut vs Control	BC (DXA)	!	!	-	+	+	-
Brennan 2020c	Nut + Ex	Nut vs Control	BC (MRI)	!	!	-	+	+	-
Brubaker 2023	Nut + Ex	Nut + Ex	PF	+	+	+	+	!	!
Cai 2019	Control	Control	BC	!	!	+	+	!	!
Coppock 2023	Nut + Ex	Nut	*no results available per study arm	!	!	-	+	!	-
Davidson 2009	Ex vs Ex	Ex vs Control	PF, BC	-	+	+	+	!	-
Elsayed 2022	Nut + Ex	Nut	BC	+	!	+	+	!	!
Elsayed 2022	Nut + Ex	Nut	PF	+	!	+	!	!	!
Fanning 2020	BCI	Control	PF, BC	+	!	+	+	+	!
Fanning 2021	Nut + Ex + BCI	Nut + Ex + BCI vs Nut + BCI	BC	!	-	+	+	!	-
Ferhi 2023	Ex	Control	PF, BC	+	-	!	+	-	-
Furlano 2023	Ex	Control	PF, BC	+	+	+	+	!	!
Gregori 2025	Nut + Ex + BCI	Control	PF, BC	+	!	+	+	!	!
Haywood 2017	Nut + Ex	Nut + Ex vs Ex	PF (DEMMI)	!	+	+	+	+	!
Haywood 2017	Nut + Ex	Nut + Ex vs Ex	PF, BC	+	+	+	+	+	+
Horie 2016	Nut	Control	PF, BC	!	!	+	+	+	!
Ismail 2023	Nut + Ex	Ex	BC	+	+	+	+	!	!
Kallings 2009	BCI	Control	BC	!	!	+	+	!	!
Kemmler 2016	Nut + Ex	Ex vs Control	PF, BC	+	+	+	+	+	+
Kemmler 2017	Nut + Ex	Ex vs Control	PF, BC	+	+	+	+	+	+
Kim 2018	Ex	Control	BC	!	!	+	+	!	!
Kim 2019	Ex	Control	PF, BC	!	-	+	+	!	-
Kim 2020	Ex	Control	PF, BC	!	!	+	+	!	!
Kitzman 2016	Nut + Ex	Nut vs Ex	PF, BC	+	+	+	+	+	+
Koh 2025	Ex	Control	BC	+	!	+	+	!	!
Kritchevsky 2017	Ex	Control	PF	!	!	+	+	+	!
Lambert 2008	Nut + BCI	Ex	PF, BC	+	+	+	+	!	!
Lee 2021	Ex	Control	PF, BC	+	+	+	+	!	!
Maillard 2016	Ex	Ex	BC	!	!	+	+	!	!
Manini 2010	Ex	Control	BC	!	-	-	+	!	-
Manini 2010	Ex	Control	PF	!	-	-	+	!	-
Mesinovic 2025	Nut + Ex	Nut	PF, BC	!	-	+	+	!	-
Miller 2006	Nut + Ex + BCI	Control	PF, BC	!	-	-	+	!	-
Muscariello 2016	NA	NA	PF, BC	!	+	+	+	!	!
Nabuco 2019	Nut + Ex	Ex	PF, BC	!	!	+	+	!	!
Nicklas 2014	Nut + Ex + BCI	Nut + Ex	BC	!	!	+	+	!	!

Normandin 2019	Nut + Ex	Nut	PF, BC	!	!	+	!	+	!
Normandin 2019	Nut + Ex	Nut	PF (ExSPPB)	!	!	+	-	+	-
O'Leary 2007	Nut + Ex	Ex	PF, BC	!	!	+	+	!	!
Park 2017	Ex	Control	PF, BC	!	+	+	+	!	!
Park 2019	Ex	Ex	PF, BC	!	!	+	+	!	!
Park 2020	Ex	Control	PF, BC	!	-	+	+	!	-
Park 2024	Ex	Ex	BC	+	+	+	+	!	!
Polo-Ferrero 2025	Ex	Control vs Ex	PF, BC	+	!	+	+	!	!
Porter Starr 2016	Nut + BCI	Nut + BCI	PF, BC	!	-	!	-	+	-
Prieto 2014	Ex	Control vs Control vs Control	PF, BC	!	!	+	+	!	!
Prieto 2015	Ex	Control	PF	!	-	-	+	!	-
Rezaeipour 2021	Ex	Ex	BC	!	!	+	+	!	!
Rosenberg 2020	BCI	Control	PF, BC	+	+	+	+	+	+
Rosenberg 2024	BCI	Control	PF, BC	+	!	+	-	+	-
Rosety 2015	Ex	Control	PF, BC	!	+	+	+	!	!
Serra-Prat 2021a	Nut + Ex	Control	BC	!	-	-	+	!	-
Serra-Prat 2021b	Nut + Ex	Control	PF	!	-	-	-	!	-
Shah 2009	Nut + Ex + BCI	Nut + BCI	PF, BC	+	+	+	+	!	!
Solomon 2009	Nut + Ex	Ex	PF, BC	!	+	+	+	!	!
Solomon 2010	Nut	Nut	PF, BC	!	!	+	+	!	!
Son 2024a	Control	Control	BC	!	+	+	!	!	!
Son 2024b	Ex	Control	BC	!	!	+	!	!	!
Stillman 2018	Ex	Control	PF, BC	!	-	!	-	!	-
Travieso 2025	Ex	Control	PF, BC	!	+	!	+	!	!
Villareal 2006	Nut + Ex + BCI	Control	PF, BC	+	+	+	+	+	+
Villareal 2011	Nut + Ex + BCI	Nut + BCI vs Ex vs Control	PF, BC	+	+	+	+	+	+
Villareal 2017	Nut + Ex + BCI	Nut + Ex + BCI vs Nut + Ex + BCI vs Control	PF, BC	+	+	+	+	+	+
Vincent 2014	Ex	Control	PF, BC	+	!	+	+	+	!
Wang 2023	Ex	Control	PF, BC	!	!	+	+	!	!
West 2011	Nut + BCI	Control	BC	!	-	+	+	!	-
Yassine 2009	Nut + BCI	Ex	PF, BC	!	+	+	+	!	!
Yin 2023	Nut + BCI	Control	PF, BC	+	+	+	+	!	!
Yin 2023	Nut + BCI	Control	BC (WC)	!	+	+	+	!	!
Zhou 2021	Nut	Control vs Control	BC	+	-	-	+	+	-

+ Low risk
! Some concerns
- High risk

D1 Randomisation process
D2 Deviations from the intended interventions
D3 Missing outcome data
D4 Measurement of the outcome
D5 Selection of the reported result

Figure S2: Risk of Bias assessment for objective outcomes

Abbreviations: BC Body composition, BCI Behavior Change Intervention, Ex Exercise, Nut Nutrition, PF Physical function

Study ID	Experimental	Comparator	Outcome	D1	D2	D3	D4	D5	Overall
Andonian 2023	Nut + Ex + BCI	Control	QoL	+	+	+	!	+	!
Ard 2017	Nut + Ex + BCI	Ex + BCI vs Ex + BCI	QoL	+	+	+	!	+	!
Balachandran 2014	Ex	Ex	PF (IADL)	-	-	+	!	!	-
Brubaker 2023	Nut + Ex	Nut + Ex	QoL	+	+	+	!	!	!
Fanning 2020	BCI	Control	QoL	+	!	+	!	+	!
Gregori 2025	Nut + Ex + BCI	Control	QoL	+	!	+	!	!	!
Kallings 2009	BCI	Control	QoL	!	!	+	!	!	!
Kim 2020	Ex	Control	QoL	!	!	+	!	!	!
Kitzman 2016	Nut + Ex	Nut vs Ex	QoL	+	+	+	!	+	!
Nicklas 2019	Nut + Ex	Nut + Ex vs Ex	QoL	!	+	+	!	!	!
Prieto 2015	Ex	Control	QoL	!	-	-	!	!	-
Rosenberg 2020	BCI	Control	QoL, PS	+	+	+	!	+	!
Serra-Prat 2021b	Nut + Ex	Control	PF (Barthel), QoL	!	-	-	!	!	-
Villareal 2006	Nut + Ex + BCI	Control	QoL	+	+	+	!	+	!
Villareal 2011	Nut + Ex + BCI	Nut + BCI vs Ex vs Control	QoL, PS	+	-	+	!	+	-
Vincent 2014	Ex	Control	QoL, PS	+	!	+	!	+	!
Yin 2023	Nut + BCI	Control	QoL	+	+	+	!	!	!

+ Low risk
! Some concerns
- High risk

D1 Randomisation process
D2 Deviations from the intended interventions
D3 Missing outcome data
D4 Measurement of the outcome
D5 Selection of the reported result

Figure S3: Risk of Bias assessment for subjective outcomes

Abbreviations: BCI Behavior Change Intervention, Ex Exercise, Nut Nutrition, PF Physical function, QoL Quality of life, PS Psychological

Figure S4: Forest plots and network graphs of network meta-analyses of different intervention modalities

Abbreviations: AE aerobic exercise; BCI behavior change intervention; Con control; CI confidence interval; CR calorie restriction; HP high protein; MD mean difference; RE resistance exercise; SMD standardized mean difference

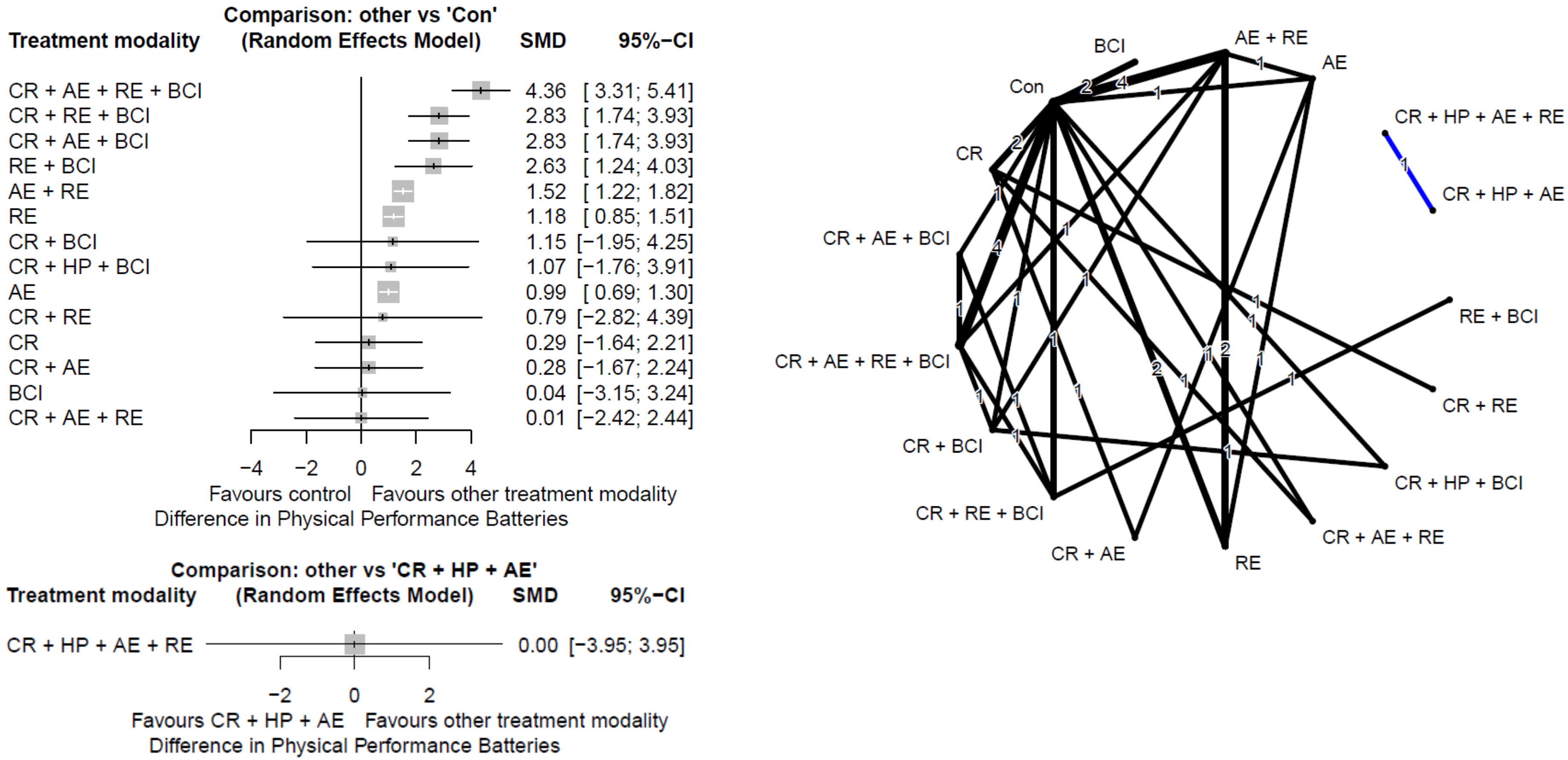

Figure S4A: Forest plot and network graph of network meta-analyses of different intervention modalities on functional status measured by performance batteries

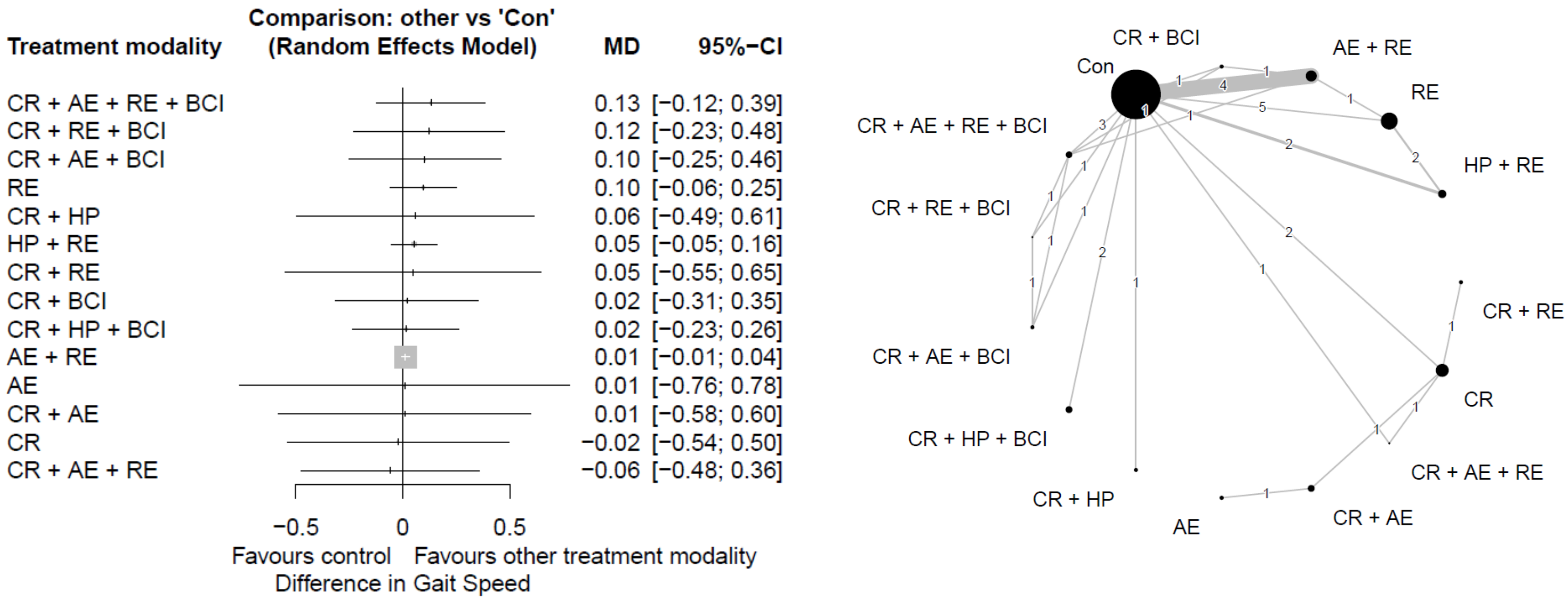

Figure S4B: Forest plot and network graph of network meta-analyses of different intervention modalities on functional status measured by gait speed

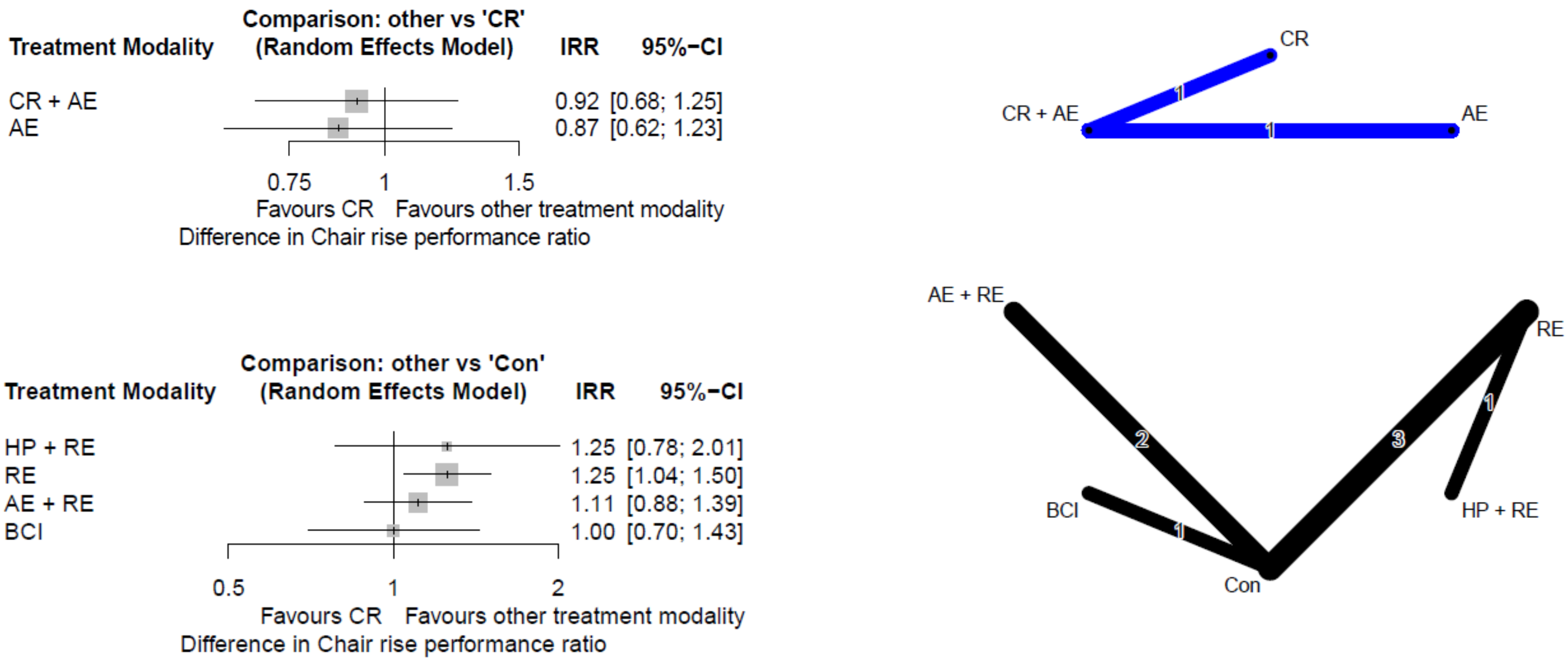

Figure S4C: Forest plots and network graph of network meta-analyses of different intervention modalities on functional status measured by chair rise test

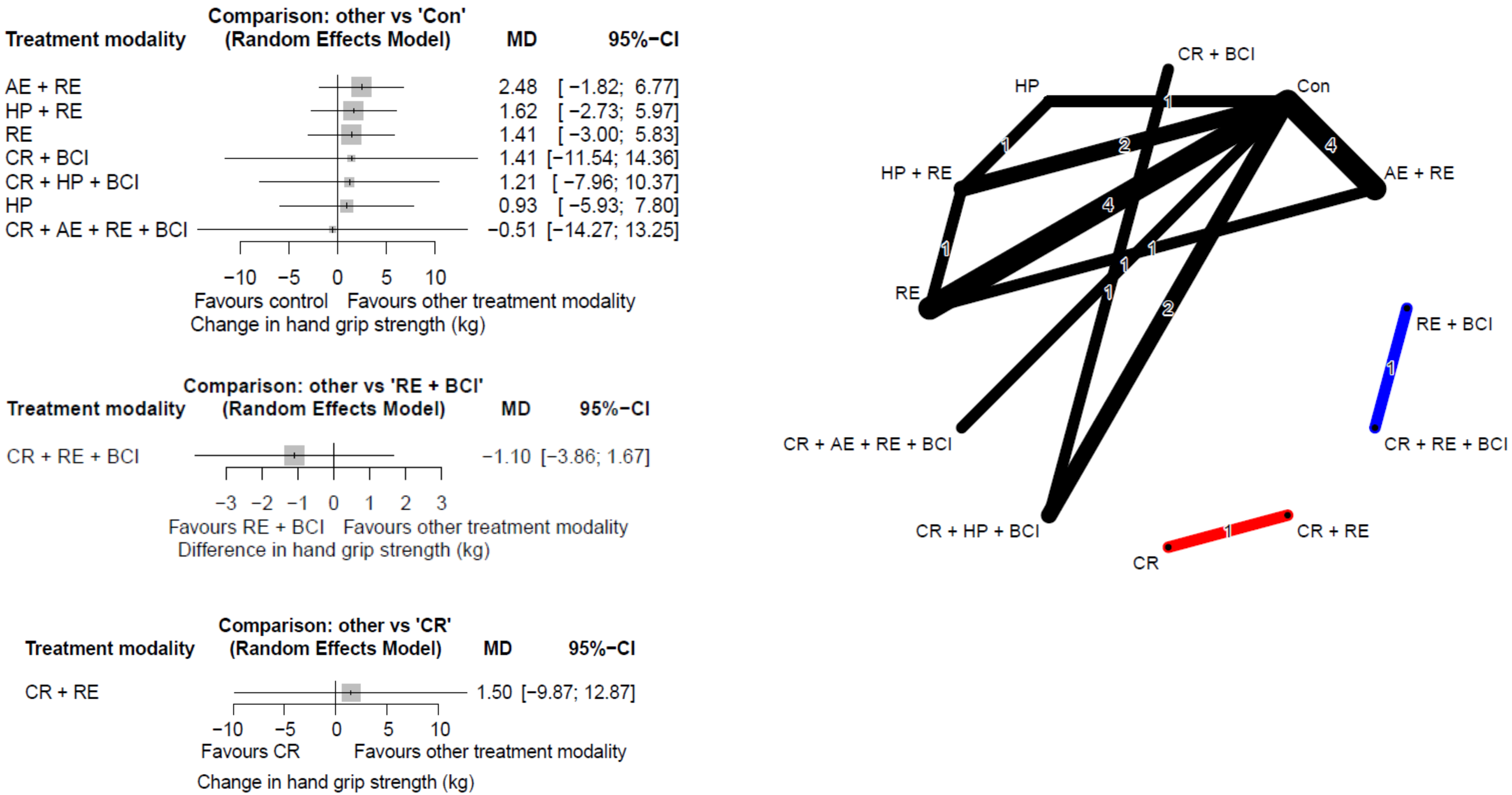

Figure S4D: Forest plots and network graph of network meta-analyses of different intervention modalities on functional status measured by hand grip strength

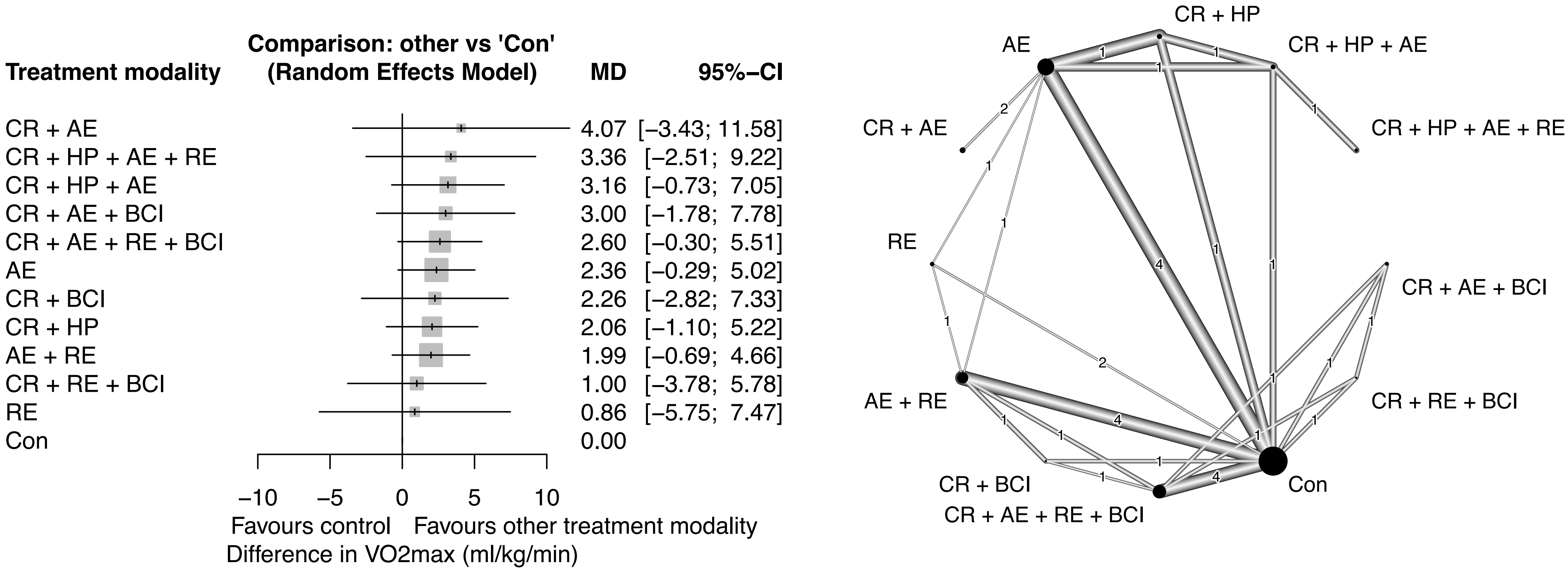

Figure S4E: Forest plot and network graph of network meta-analysis of different intervention modalities on functional status measured by VO_2max

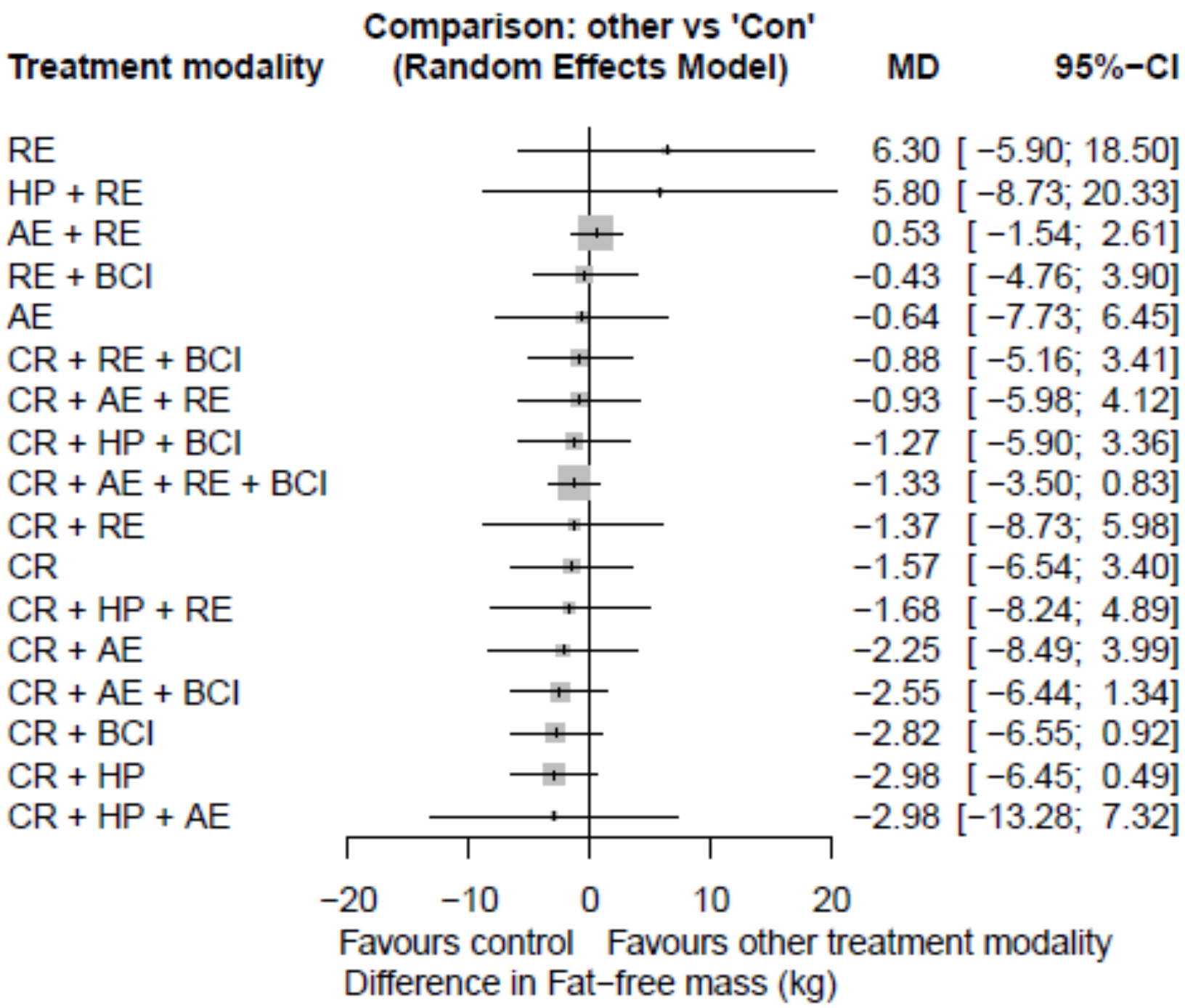

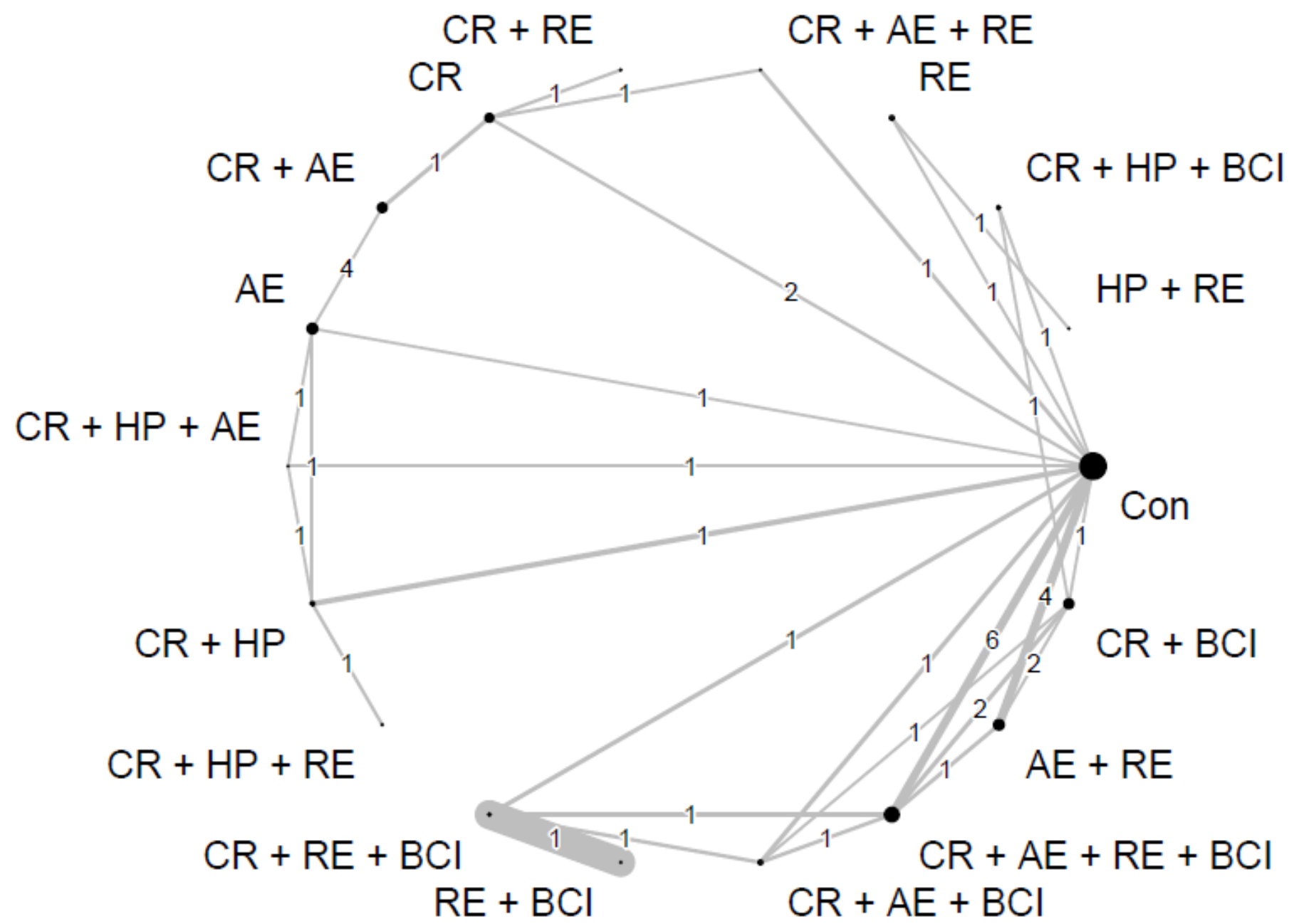

Figure S4F: Forest plots and network graph of network meta-analyses of different intervention modalities on fat-free mass

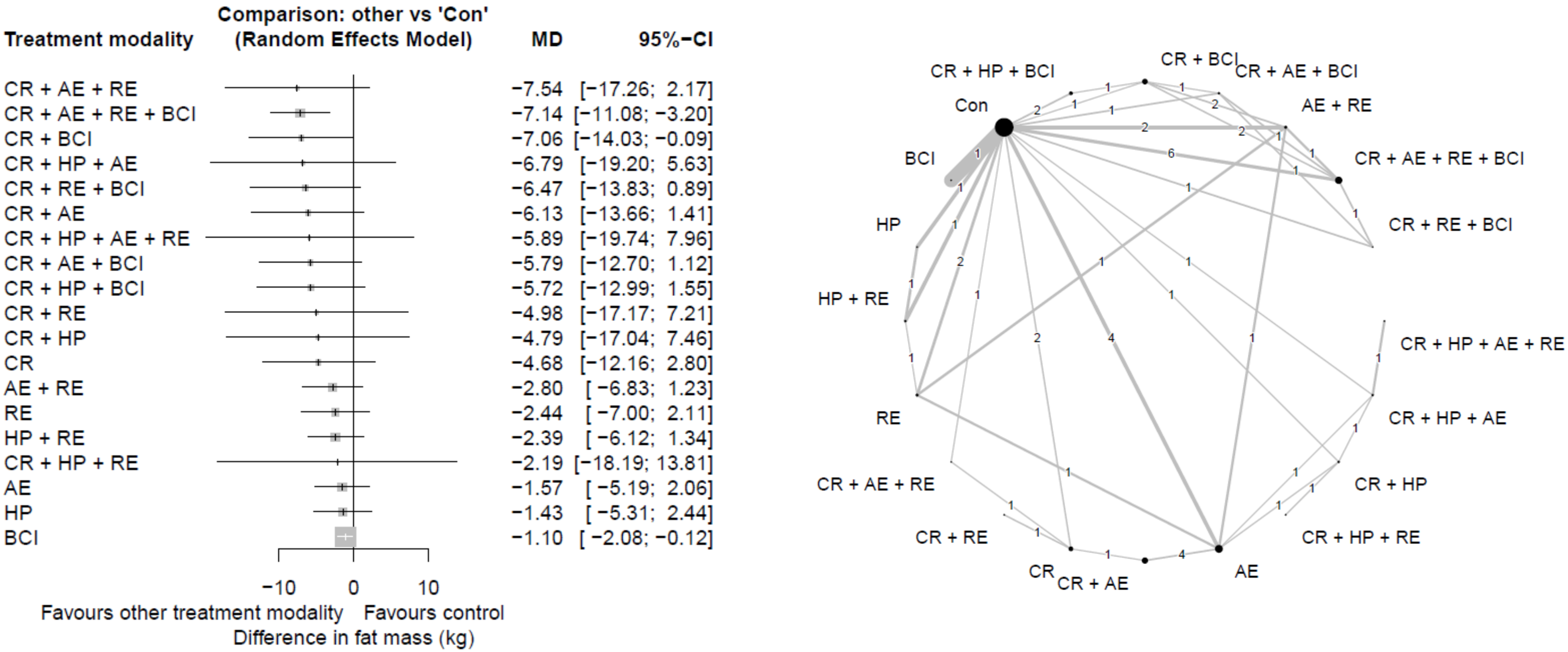

Figure S4G: Forest plot and network graph of network meta-analysis of different intervention modalities on fat mass

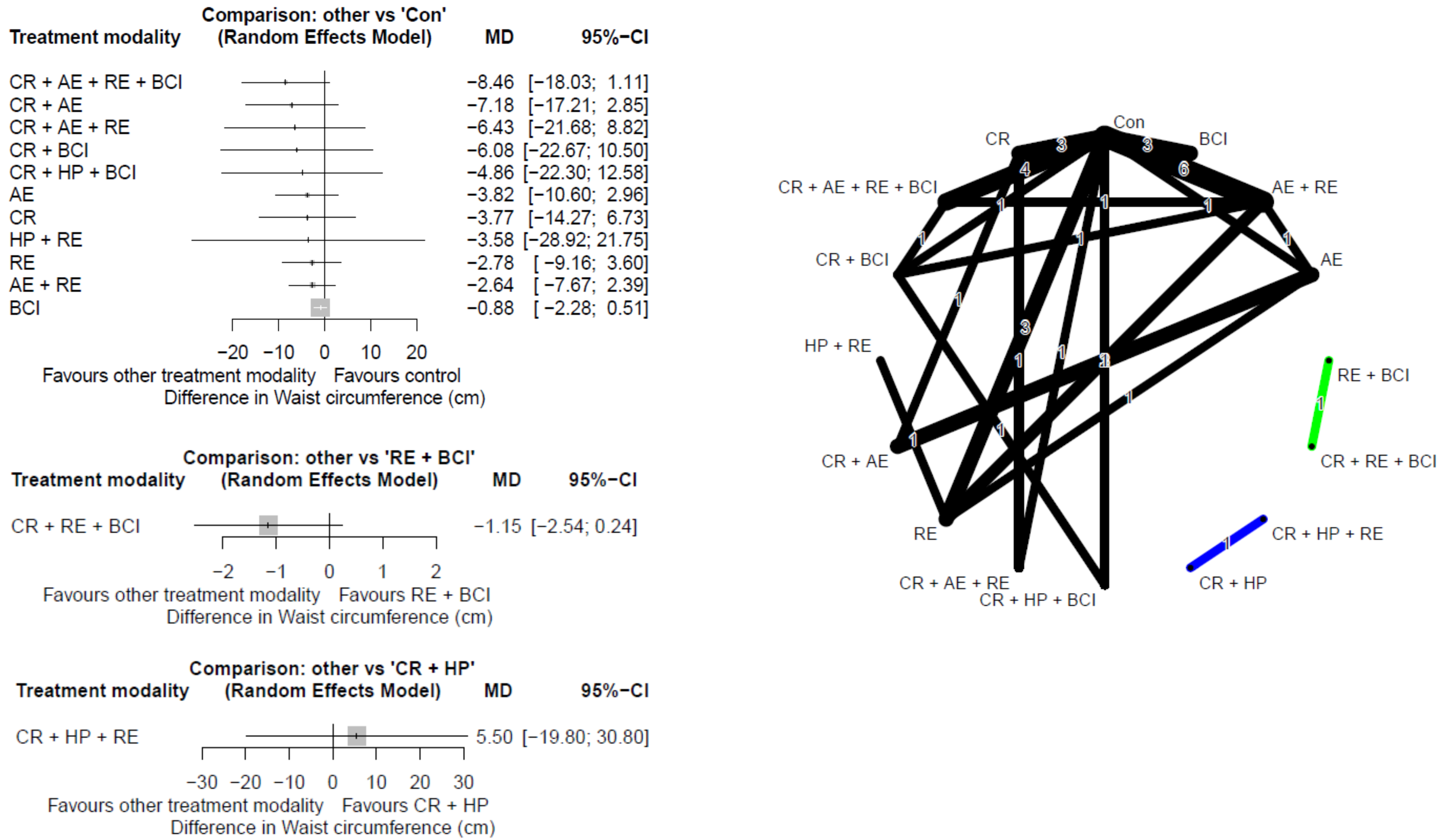

Figure S4H: Forest plots and network graph of network meta-analyses of different intervention modalities on waist-circumference

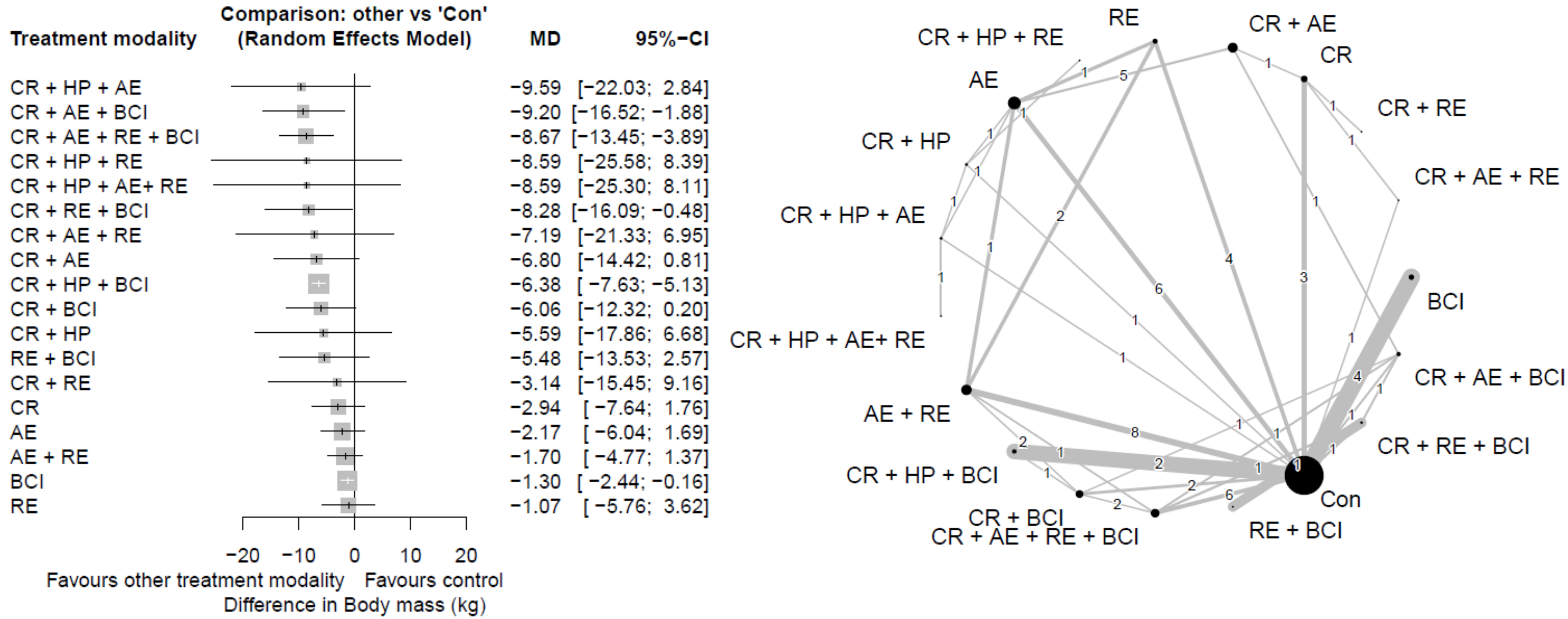

Figure S4I: Forest plot and network graph of network meta-analyses of different intervention modalities on body weight

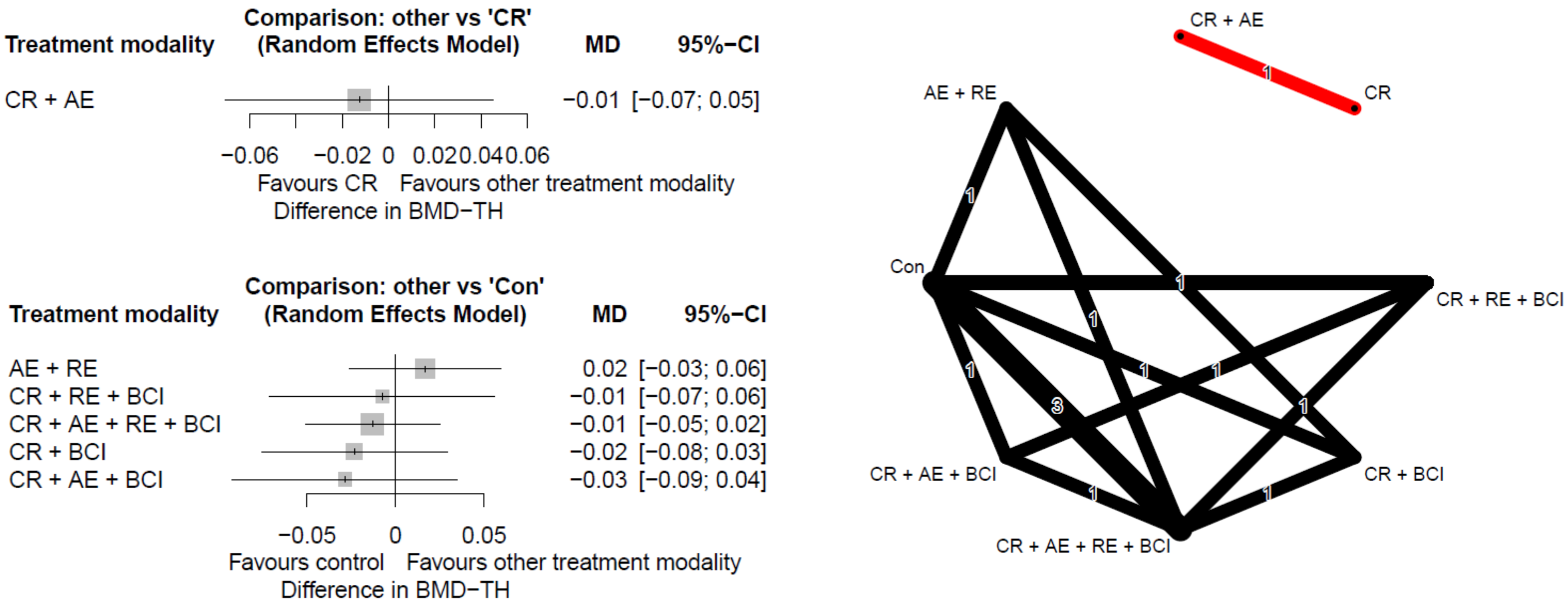

Figure S4J: Forest plots and network graph of network meta-analyses of different intervention modalities on bone mineral density of the total hip

Figures S5-S14: Forest plots of component and standard network meta-analyses

Abbreviations: AE aerobic exercise; BCI behavior change intervention; Con control; CI confidence interval; CR calorie restriction; EX exercise; HP high protein; MD mean difference; NUT nutrition; RE resistance exercise; SMD standardized mean difference

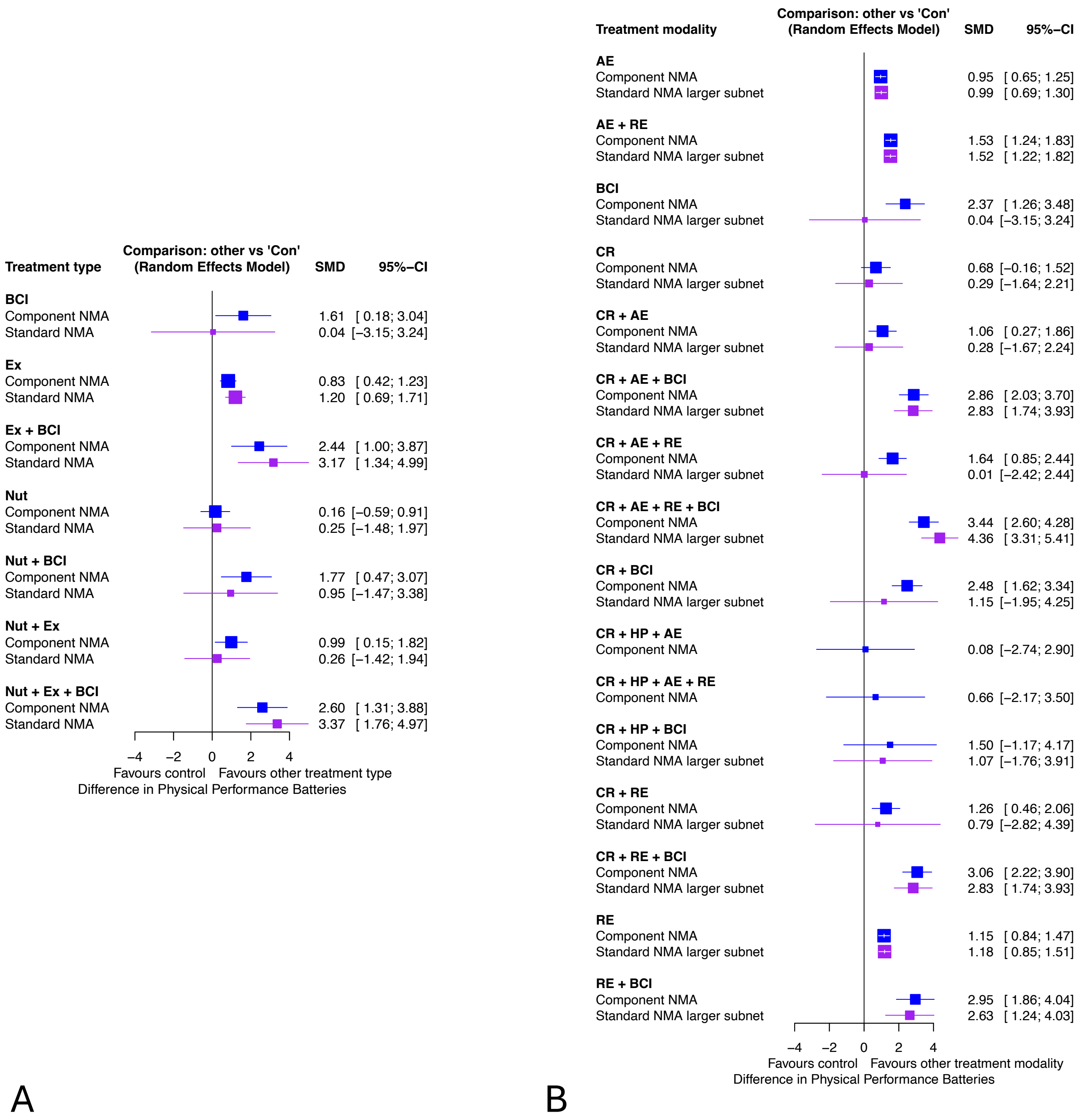

Figure S5: Forest plot of component and standard network meta-analyses of different intervention types (A) and modalities (B) on functional status measured by performance batteries

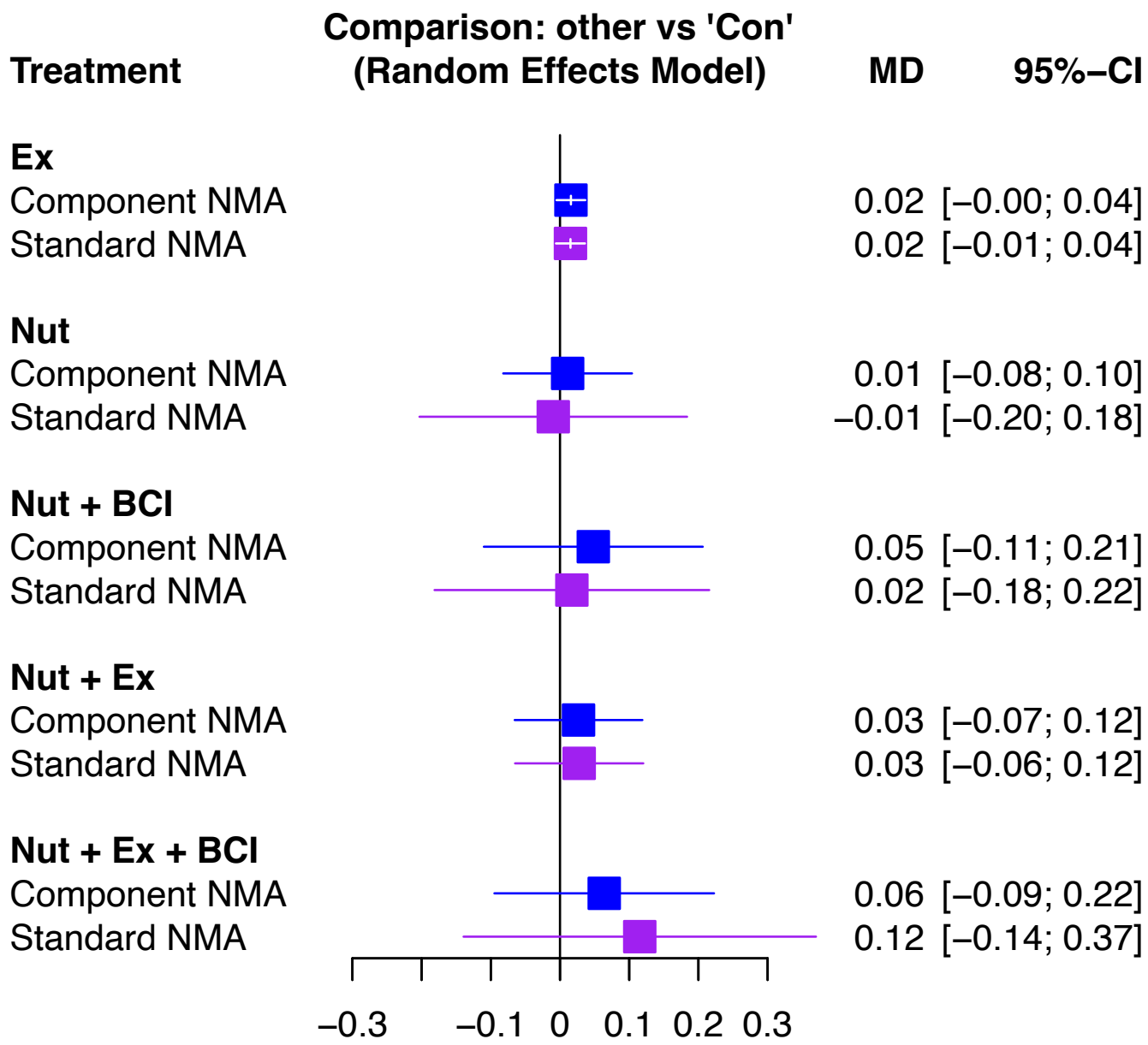

A

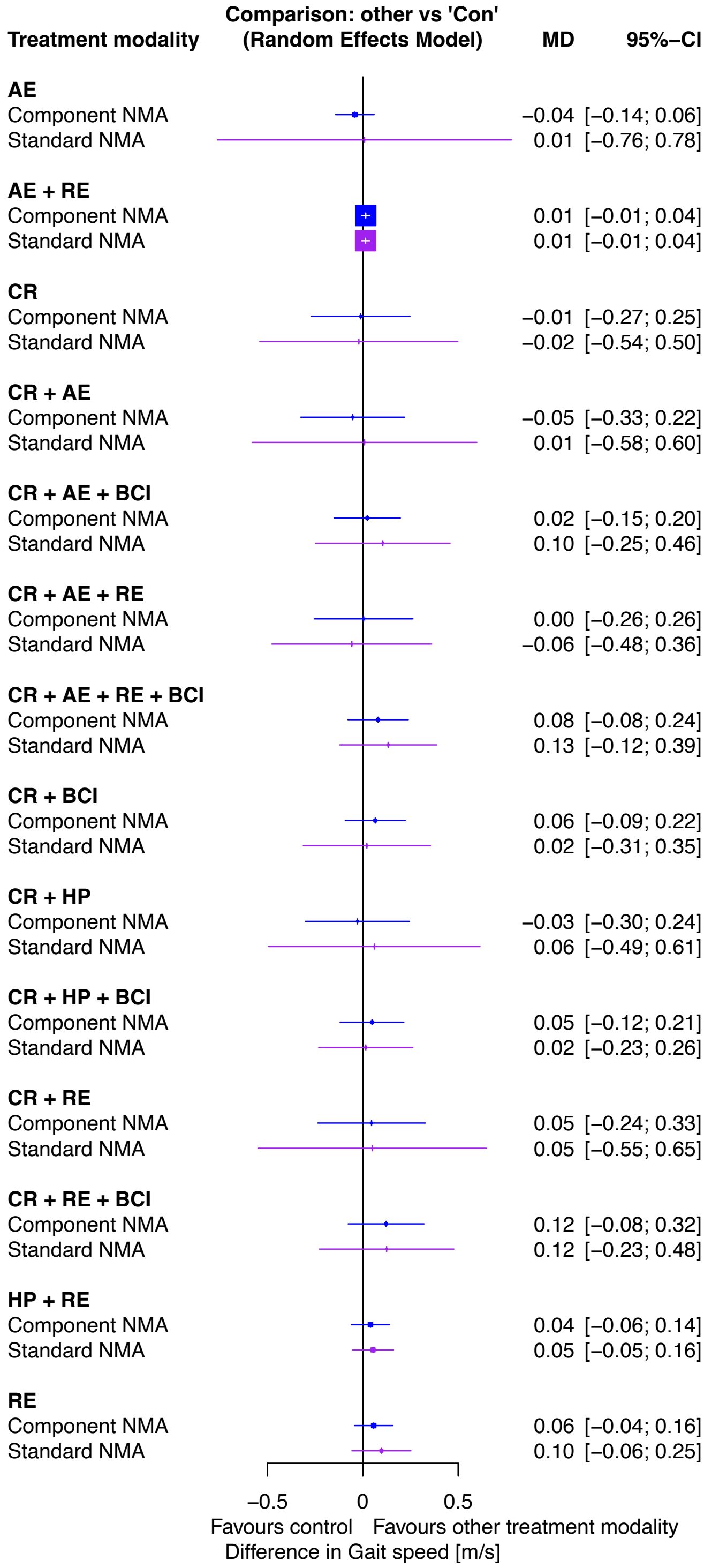

B

Figure S6: Forest plots of component and standard network meta-analyses of different intervention types (A) and modalities (B) on functional status measured by gait speed

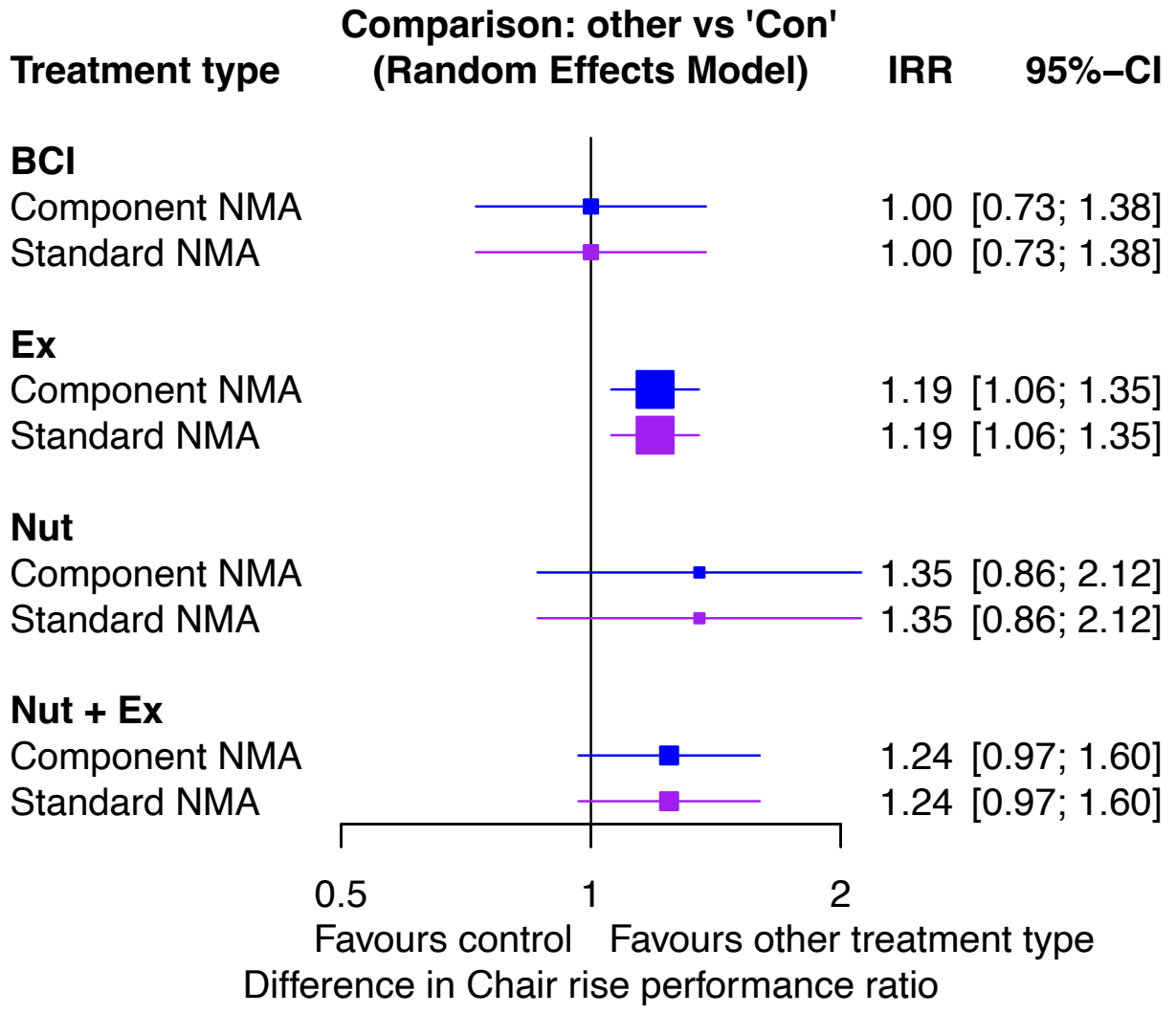

A

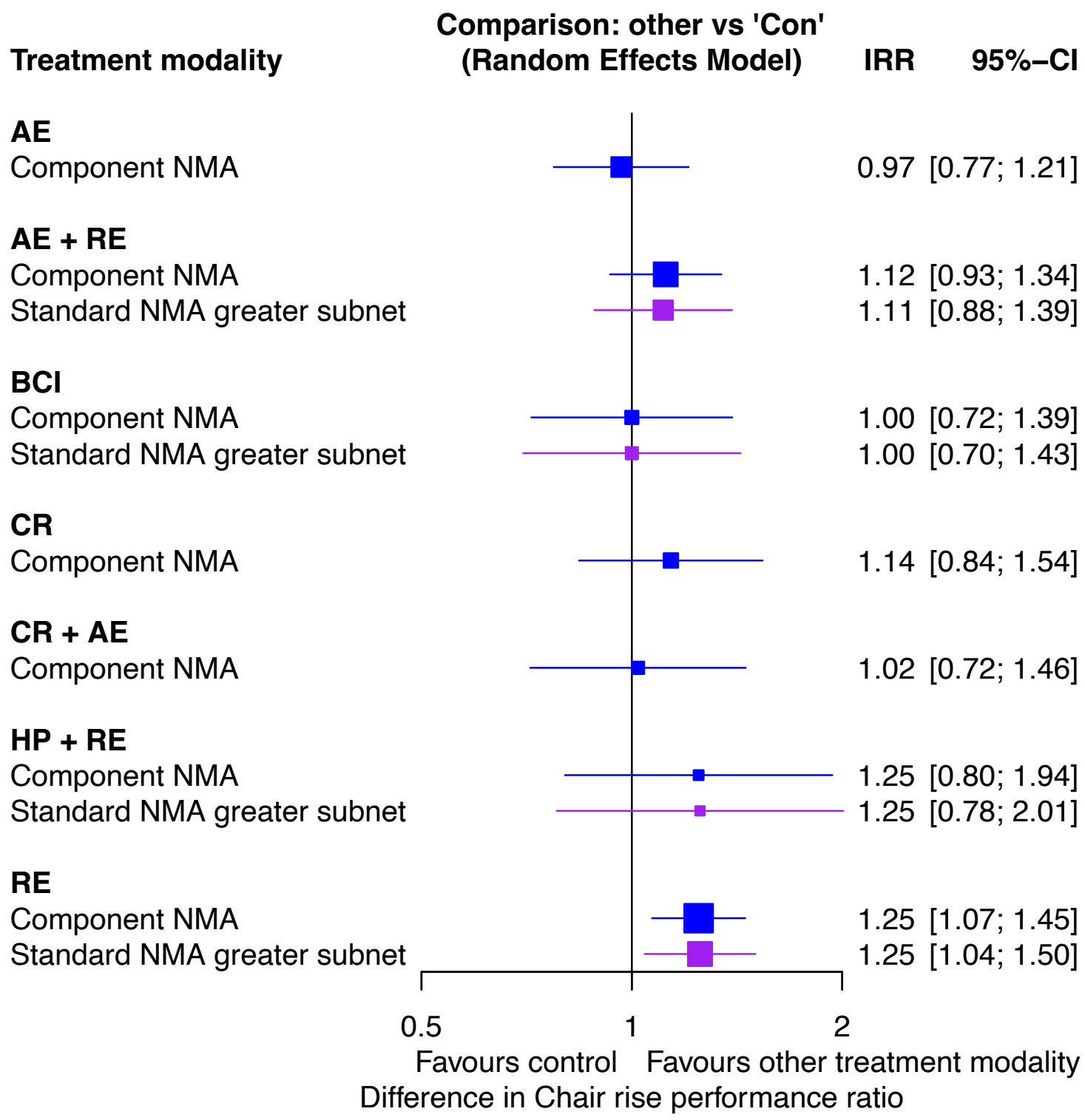

B

Figure S7: Forest plot of component and standard network meta-analyses of different intervention types (A) and modalities (B) on functional status measured by chair rise test

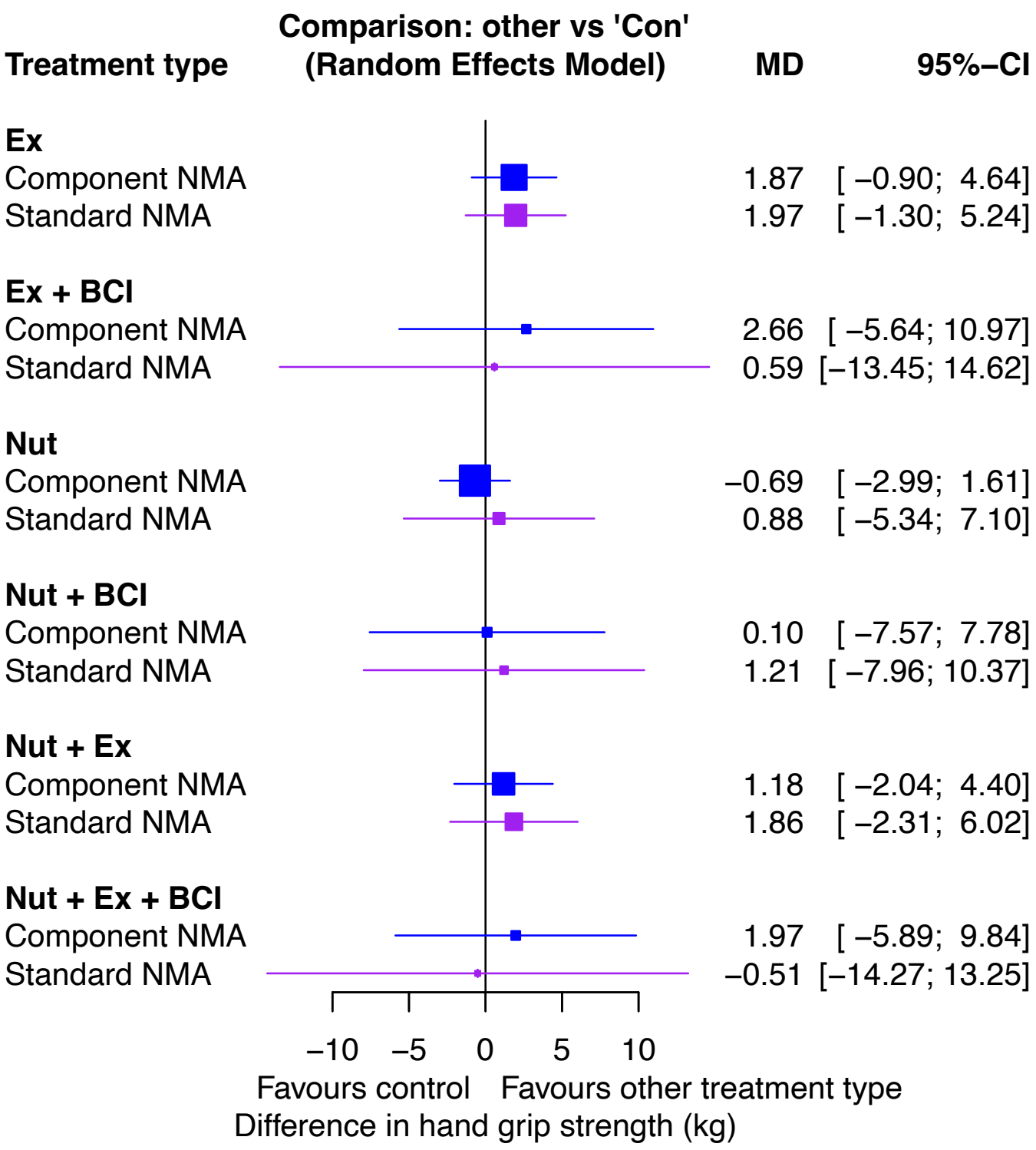

A

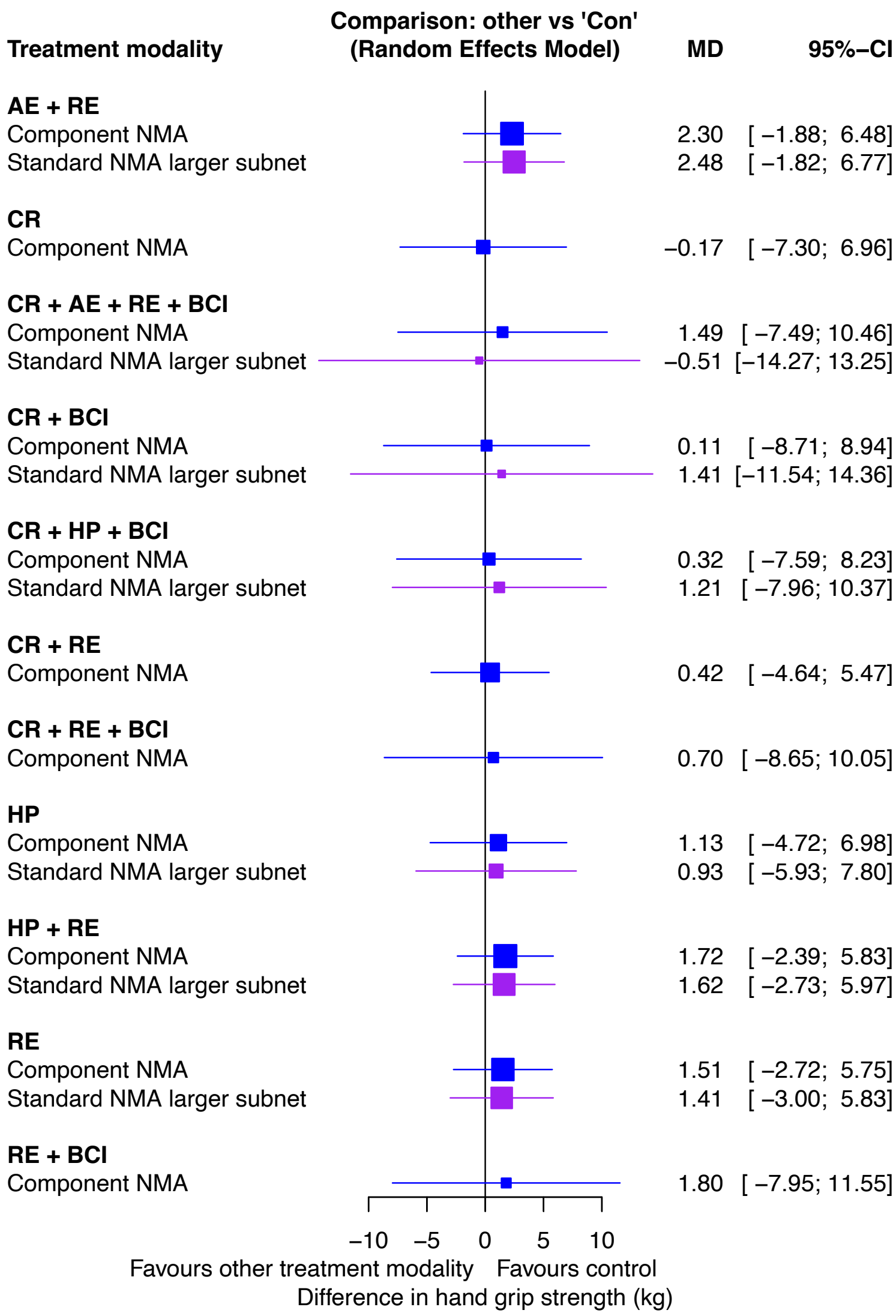

B

Figure S8: Forest plot of component and standard network meta-analyses of different intervention types (A) and modalities (B) on functional status measured by hand-grip strength

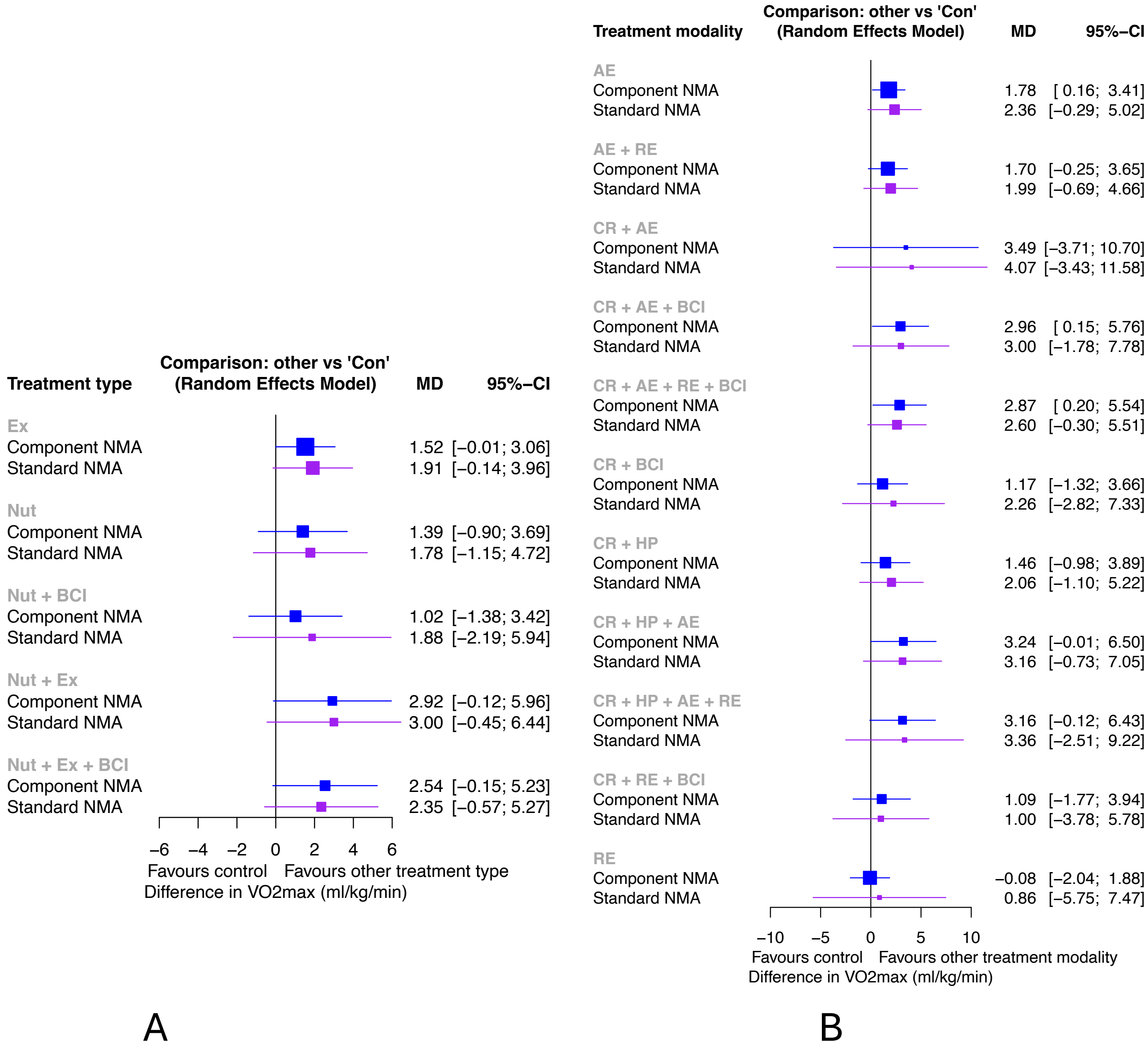

Figure S9: Forest plot of component and standard network meta-analyses of different intervention types (A) and modalities (B) on functional status measured by VO_2-max

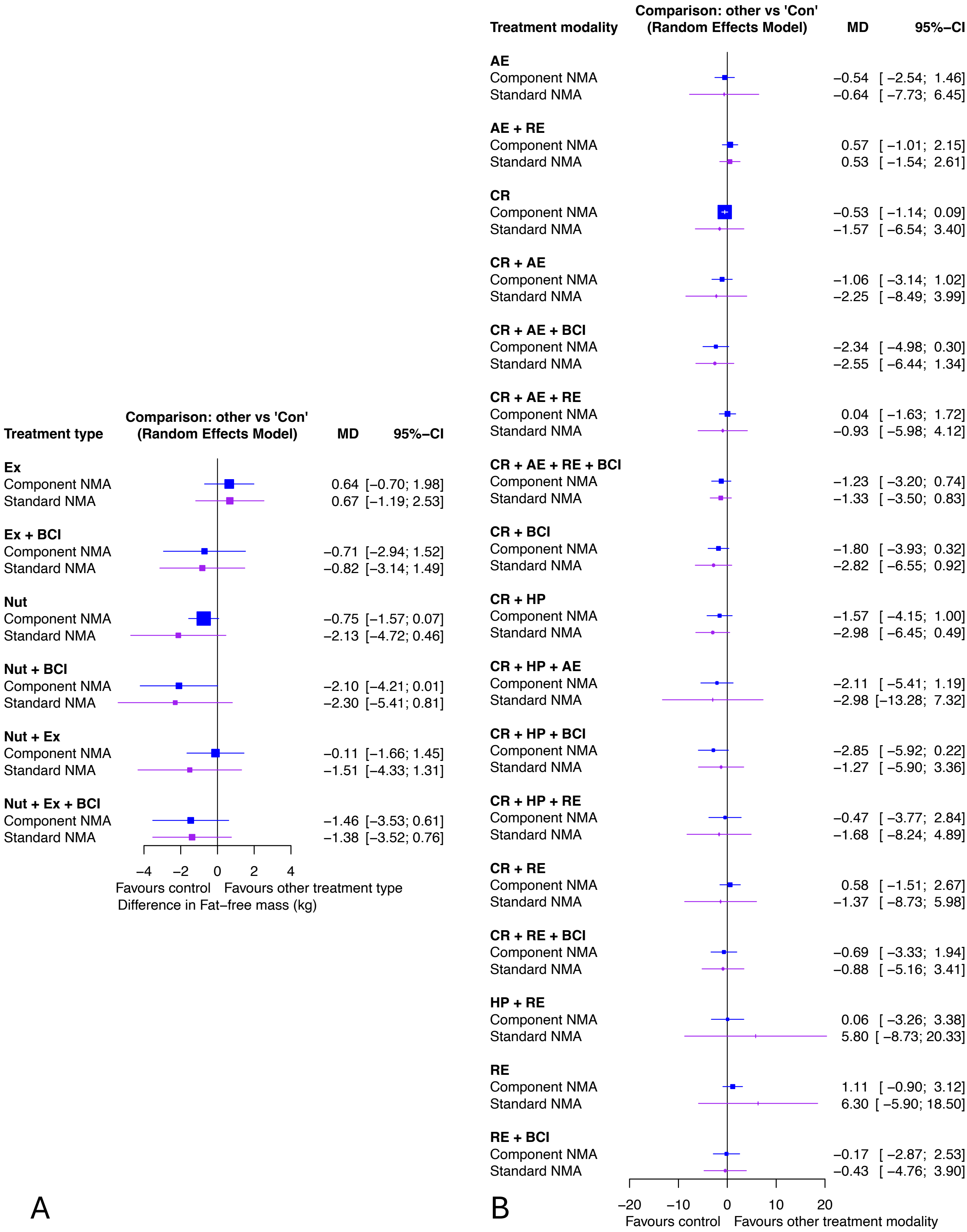

Figure S10: Forest plot of component and standard network meta-analyses of different intervention types (A) and modalities (B) on fat-free mass (kg)

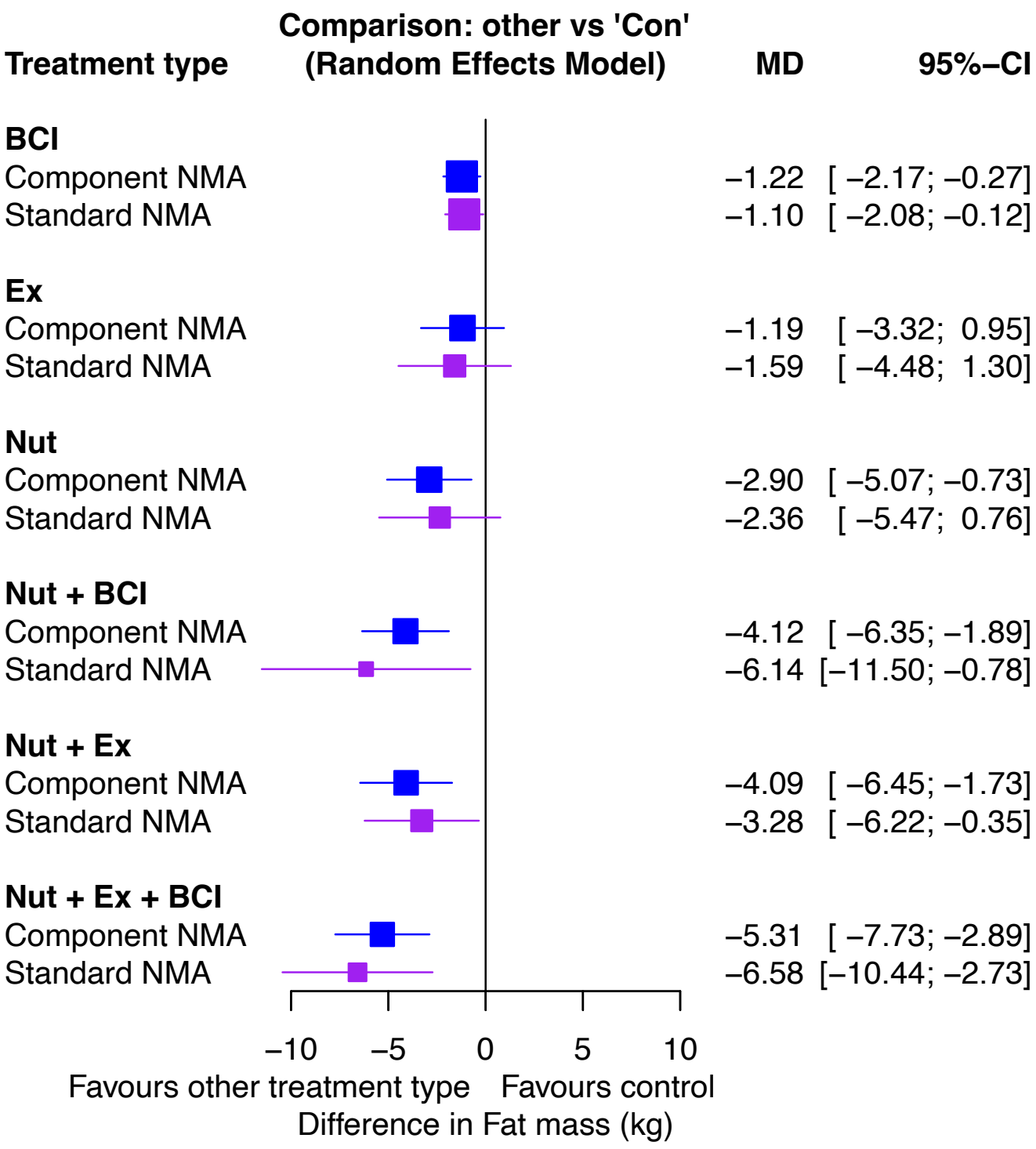

A

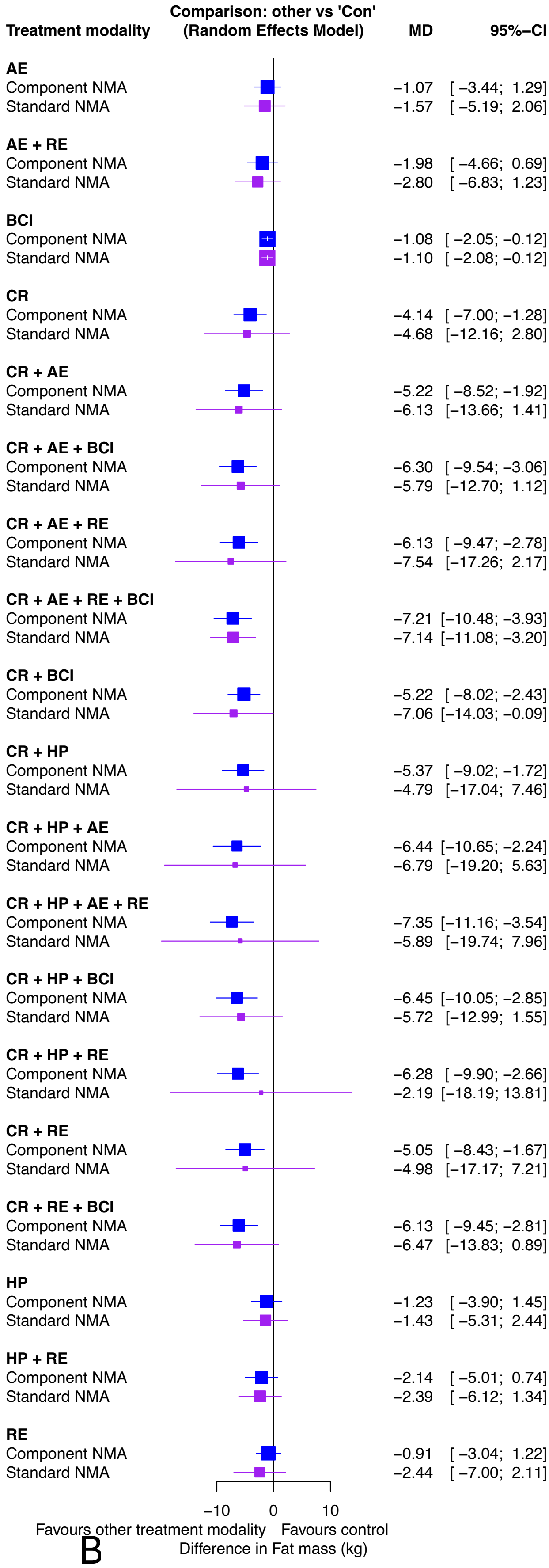

B

Figure S11: Forest plot of component and standard network meta-analyses of different intervention types (A) and modalities (B) on fat mass (kg)

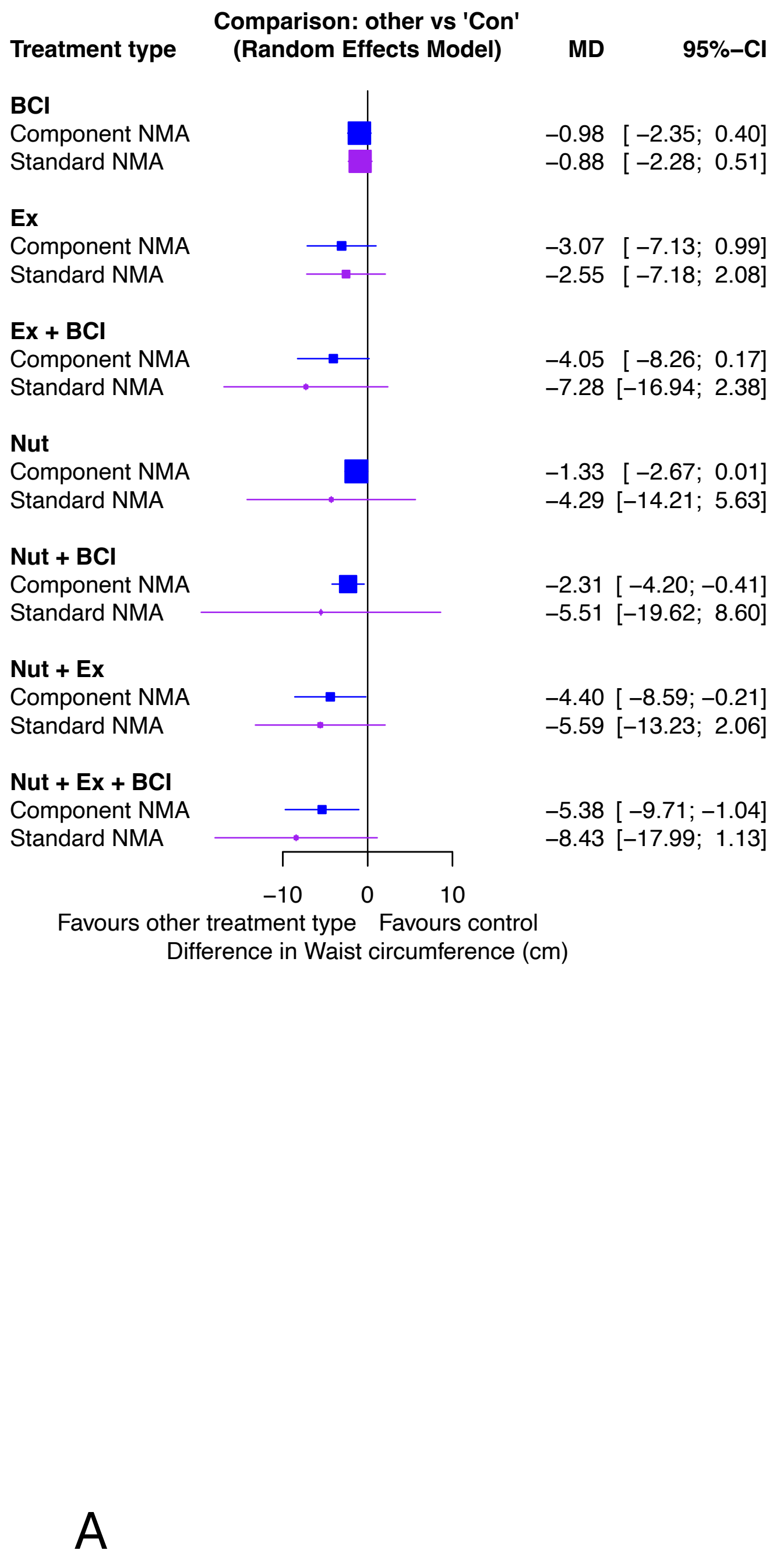

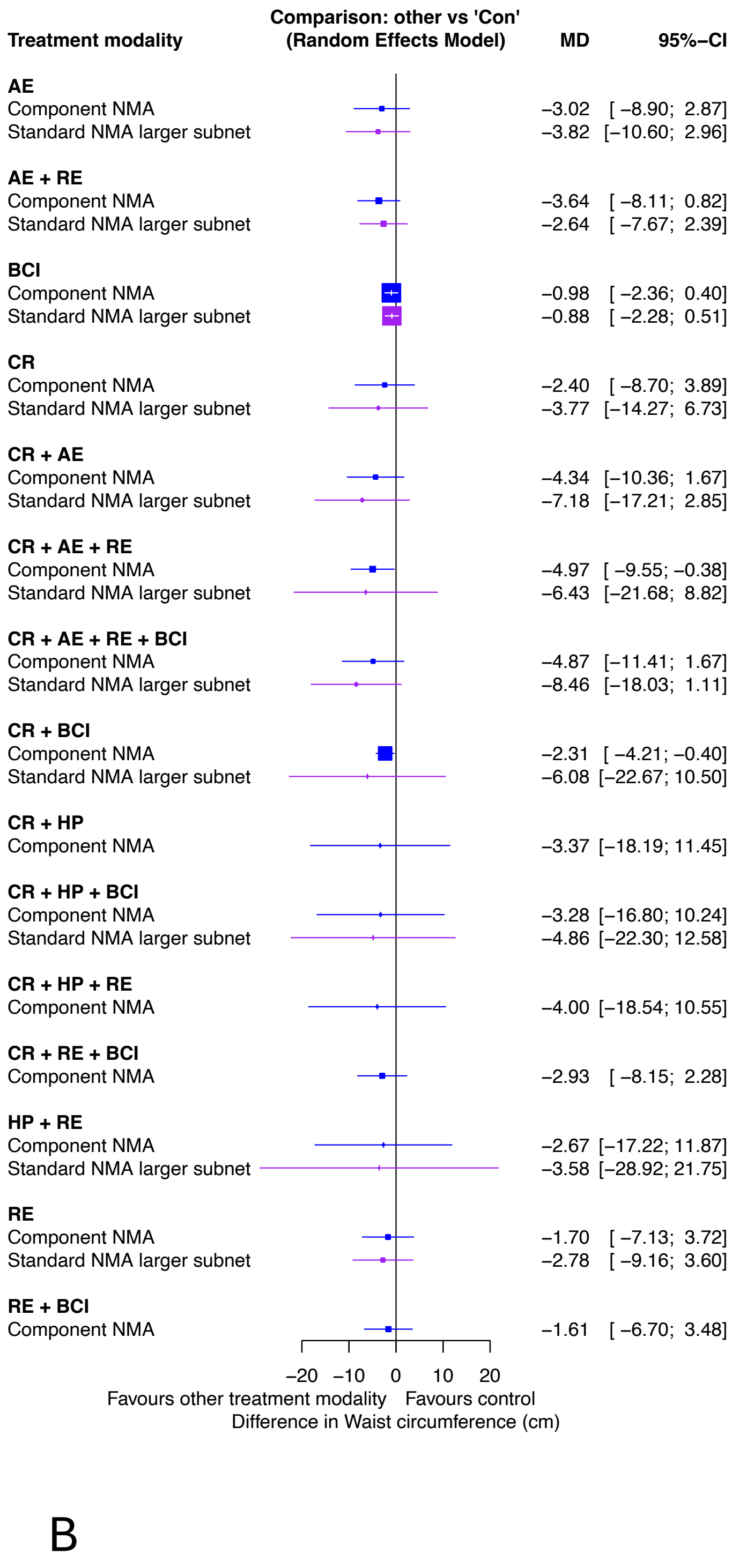

Figure S12: Forest plot of component and standard network meta-analyses of different intervention types (A) and modalities (B) on waist circumference (cm)

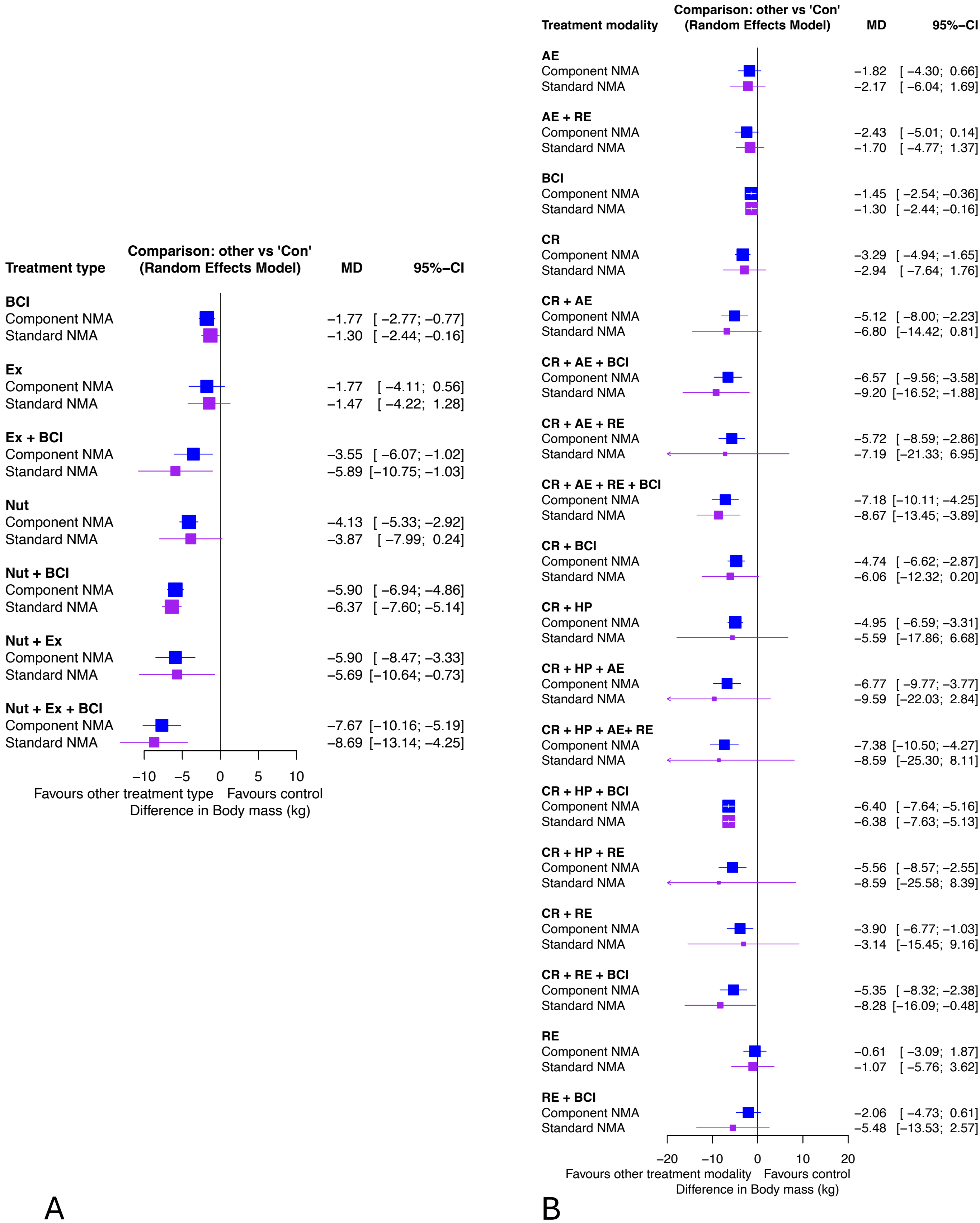

Figure S13: Forest plot of component and standard network meta-analyses of different intervention types (A) and modalities (B) on body mass (kg)

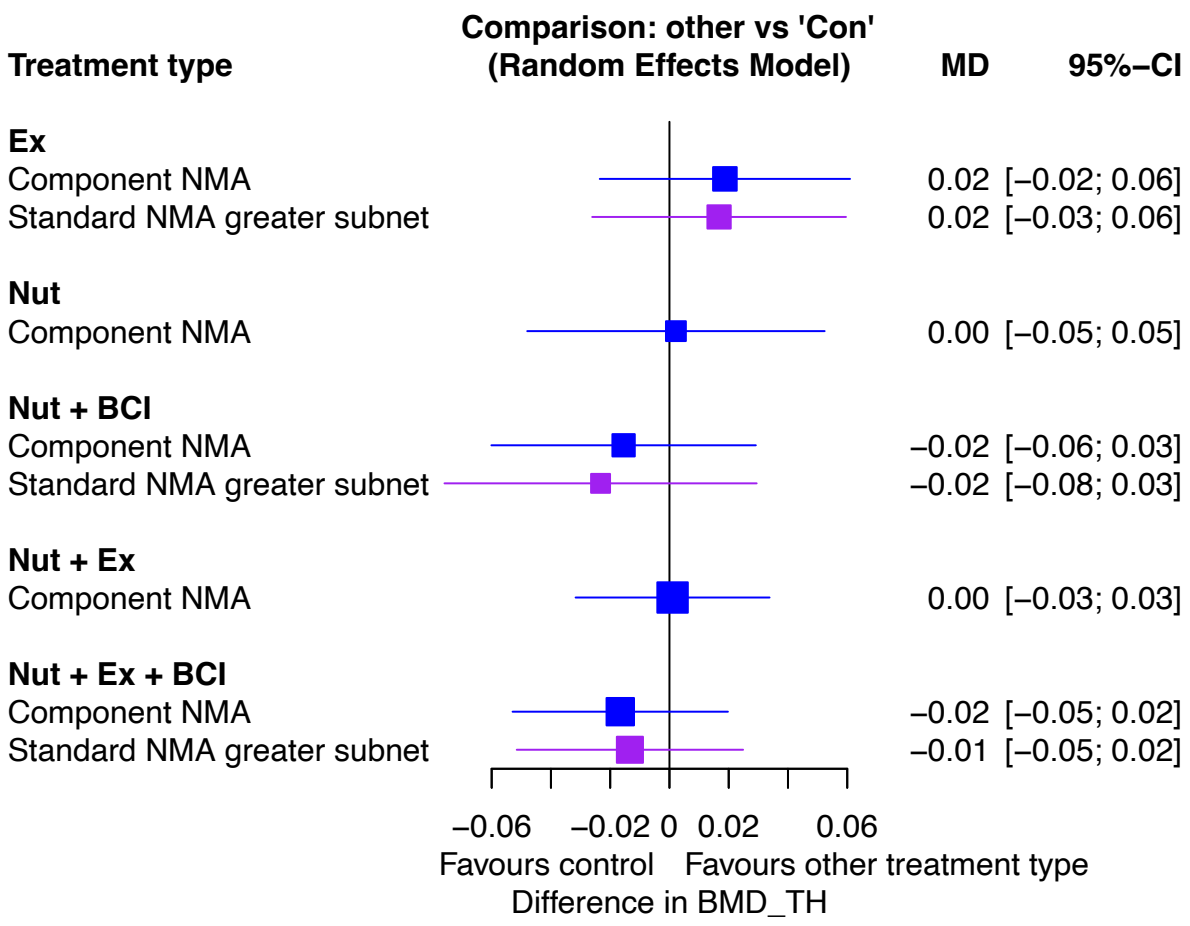

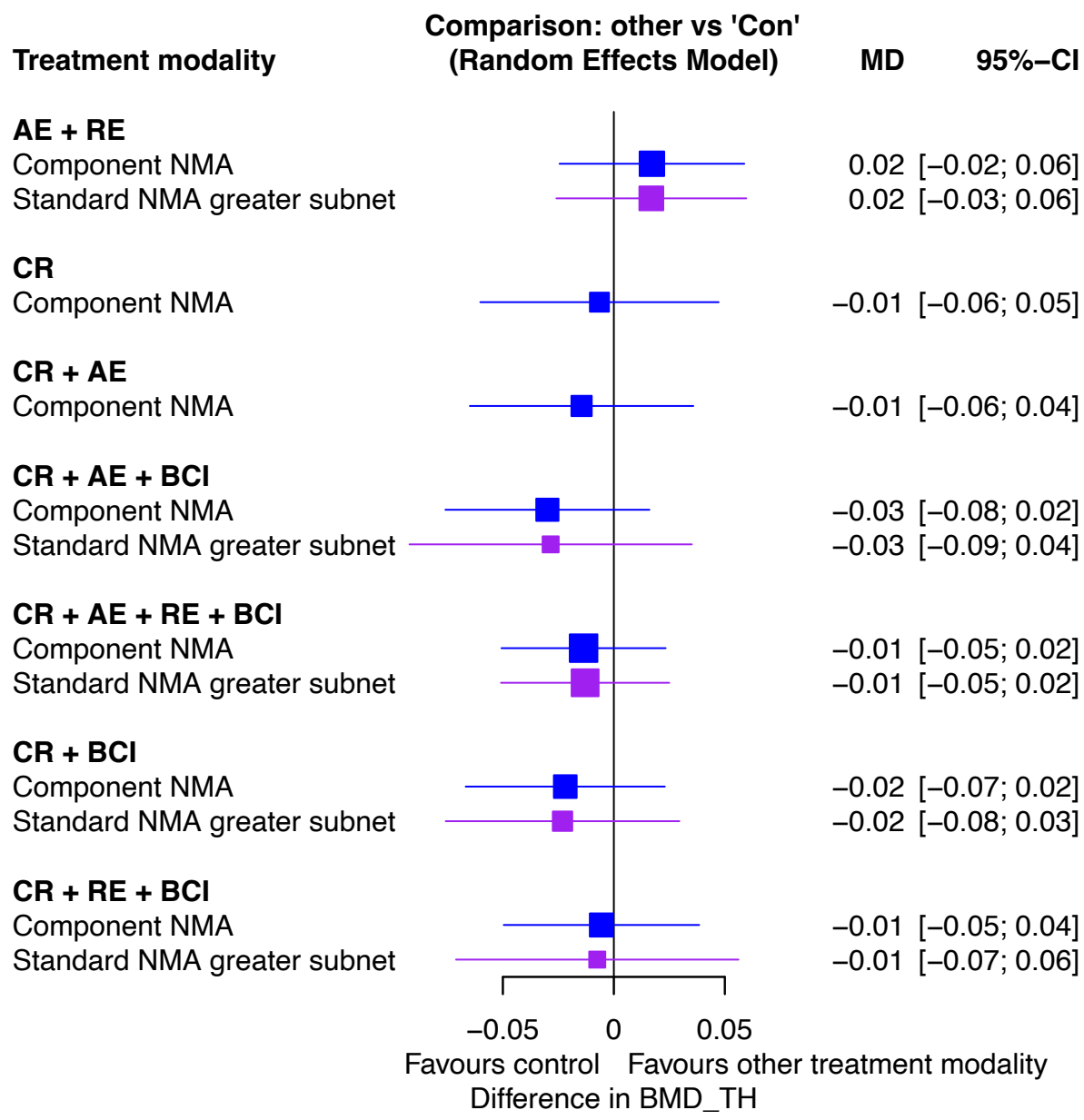

A

B

Figure S14: Forest plot of component and standard network meta-analyses of different intervention types (A) and modalities (B) on bone mineral density of the total hip

Figures S15-S24: Funnel plots

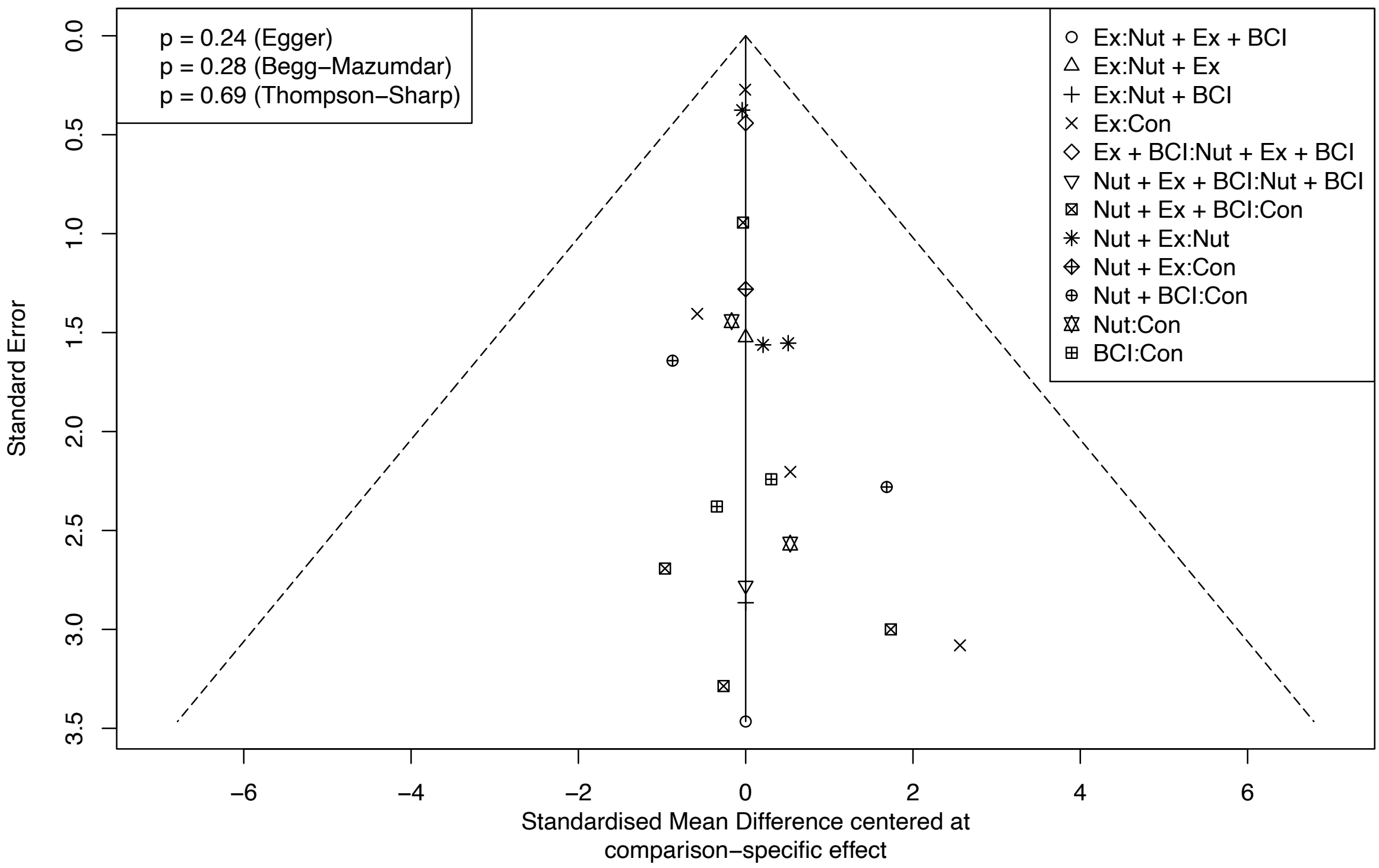

Figure S15: Funnel plot for physical performance batteries (NMA on intervention type)

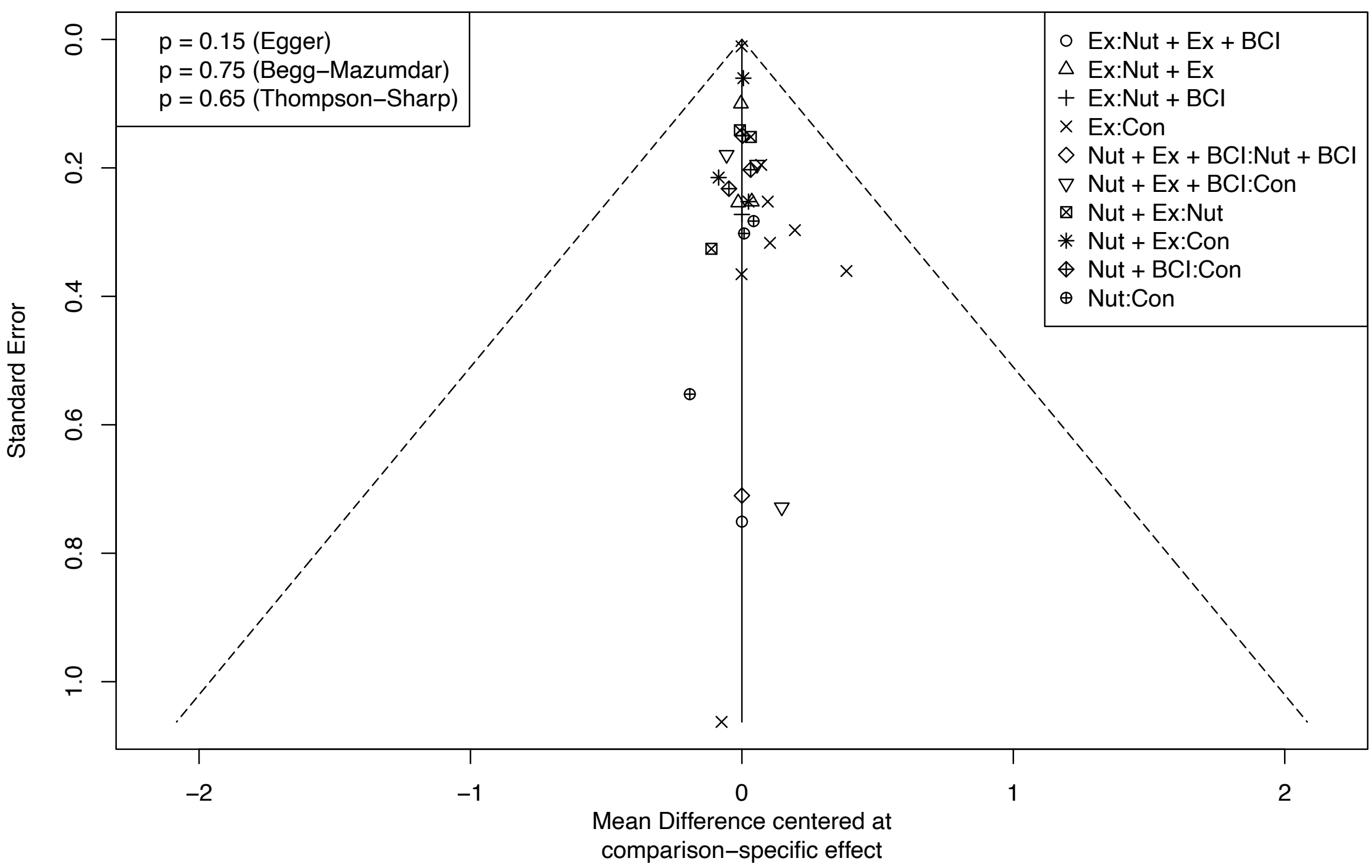

Figure S16: Funnel plot for gait speed (NMA on intervention type)

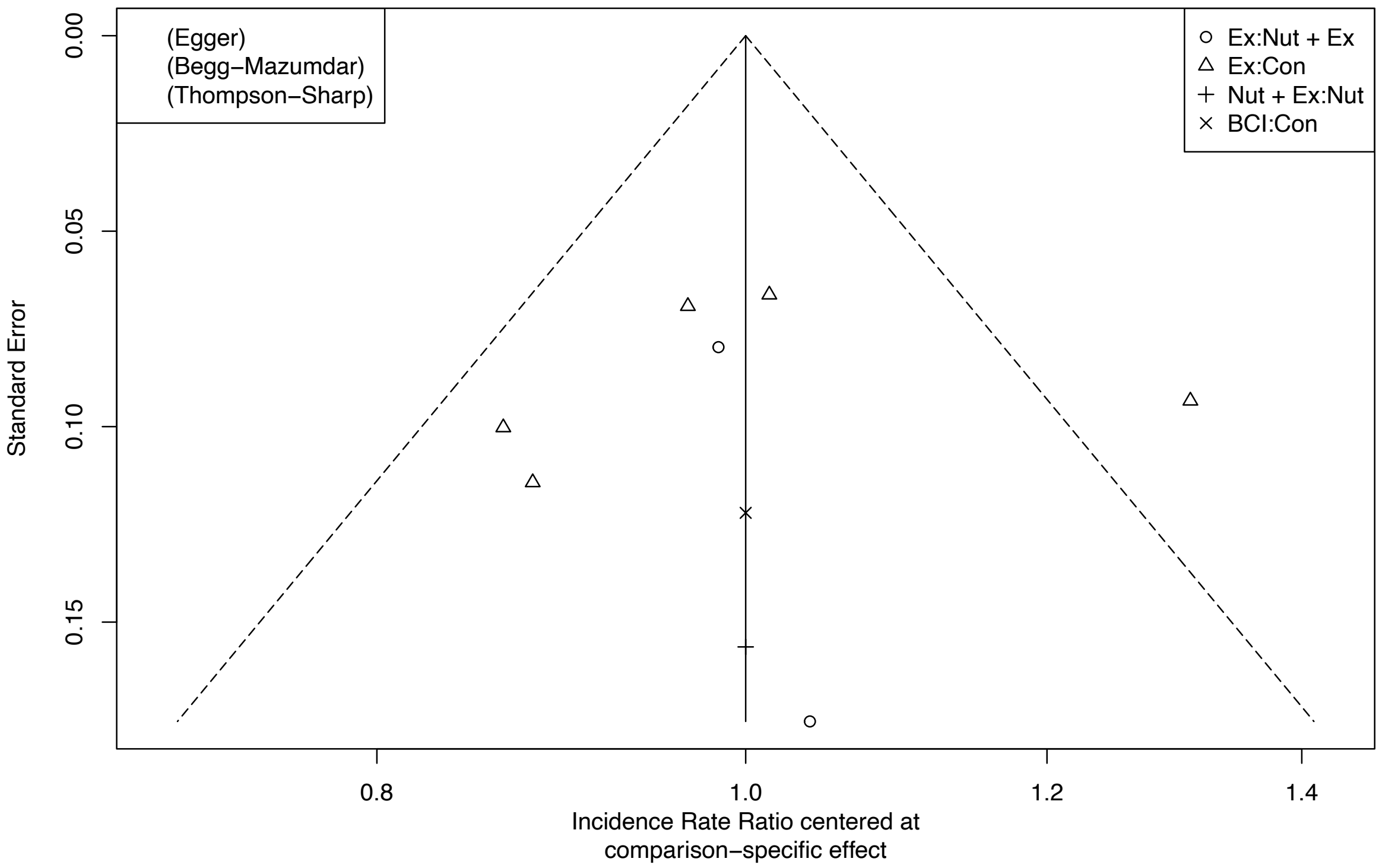

Figure S17: Funnel plot for chair rise ratio (NMA on intervention type)

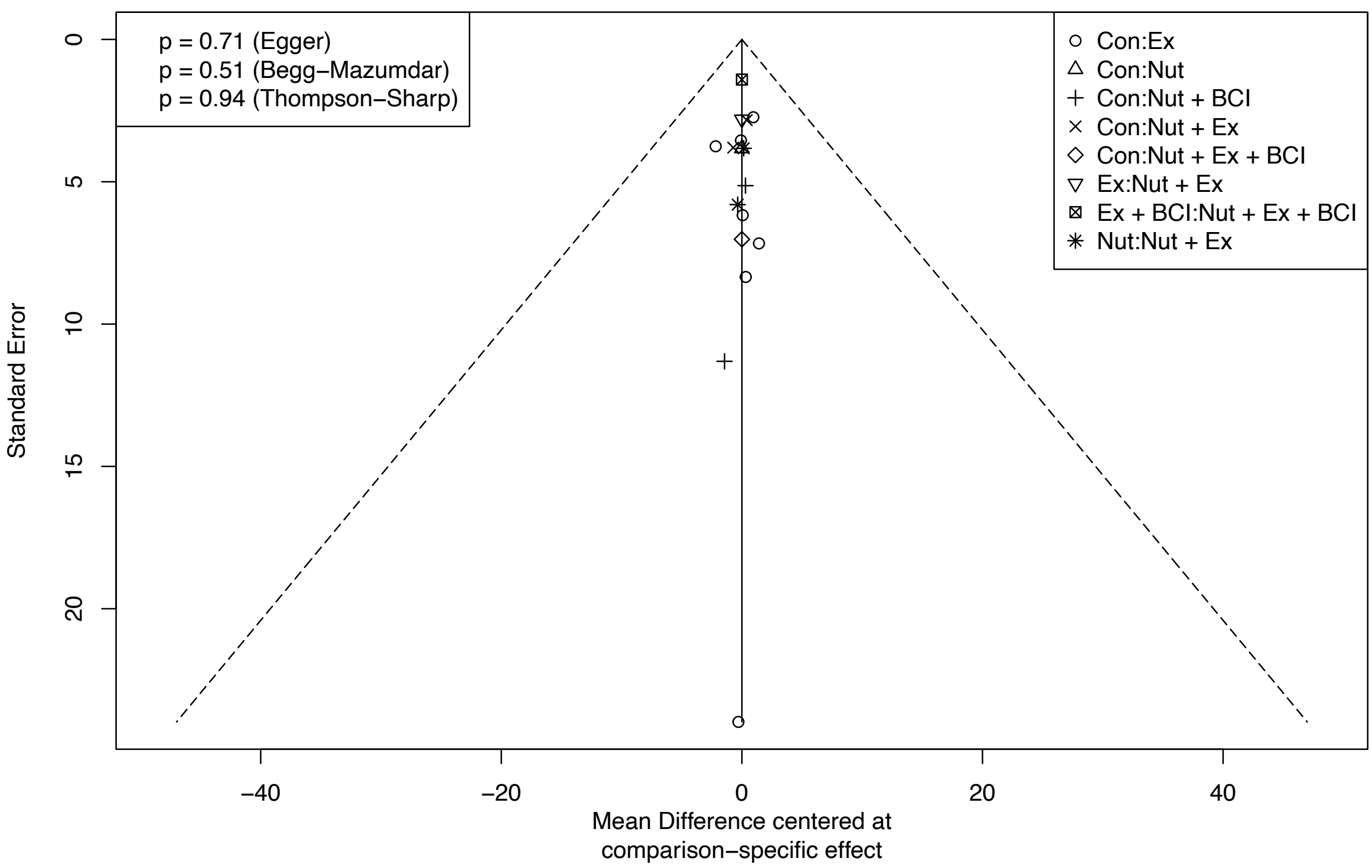

Figure S18: Funnel plot for hand-grip strength (NMA on intervention type)

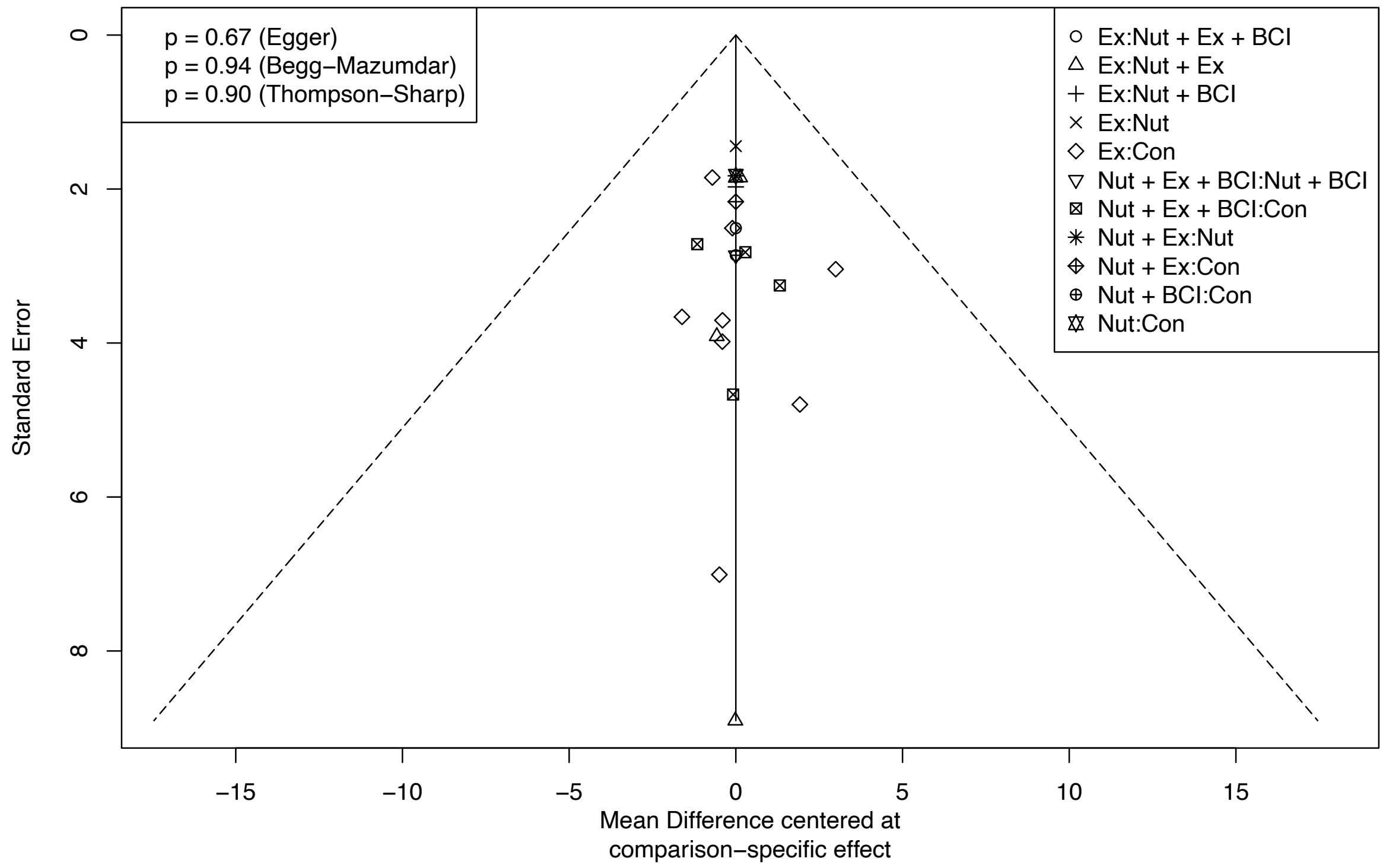

Figure S19: Funnel plot for VO_2-max (NMA on intervention type)

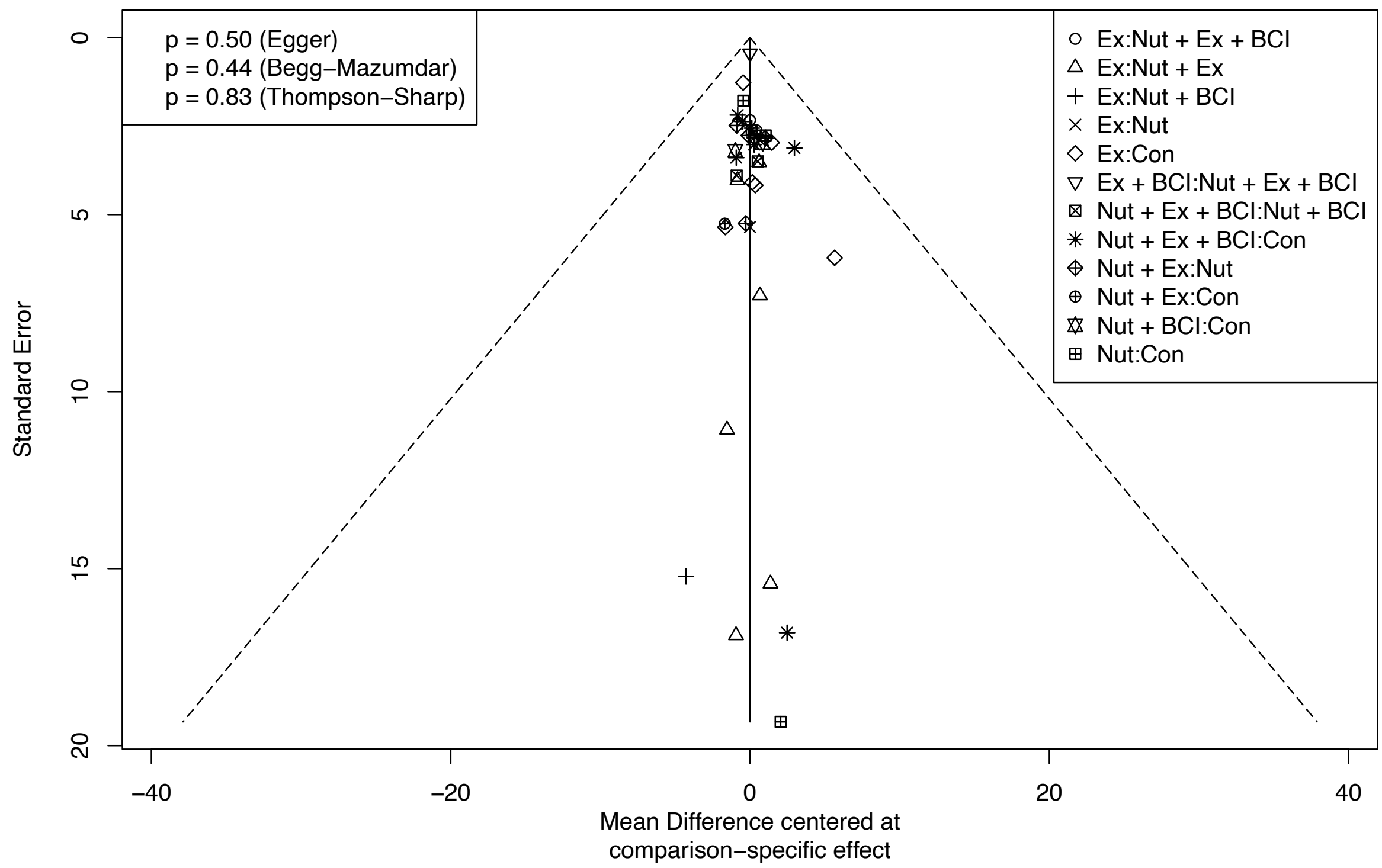

Figure S20: Funnel plot for fat-free mass (NMA on intervention type)

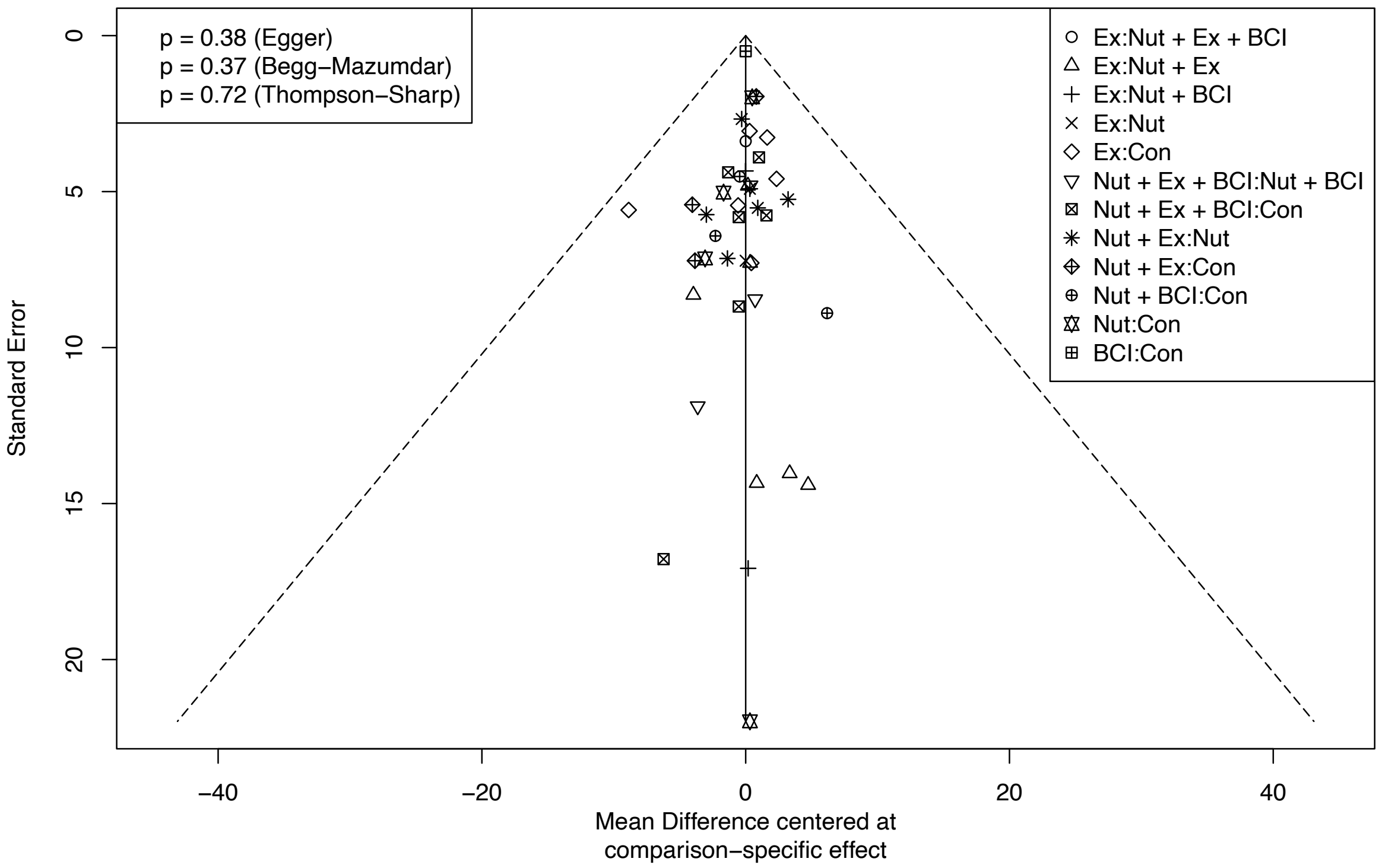

Figure S21: Funnel plot for fat mass (NMA on intervention type)

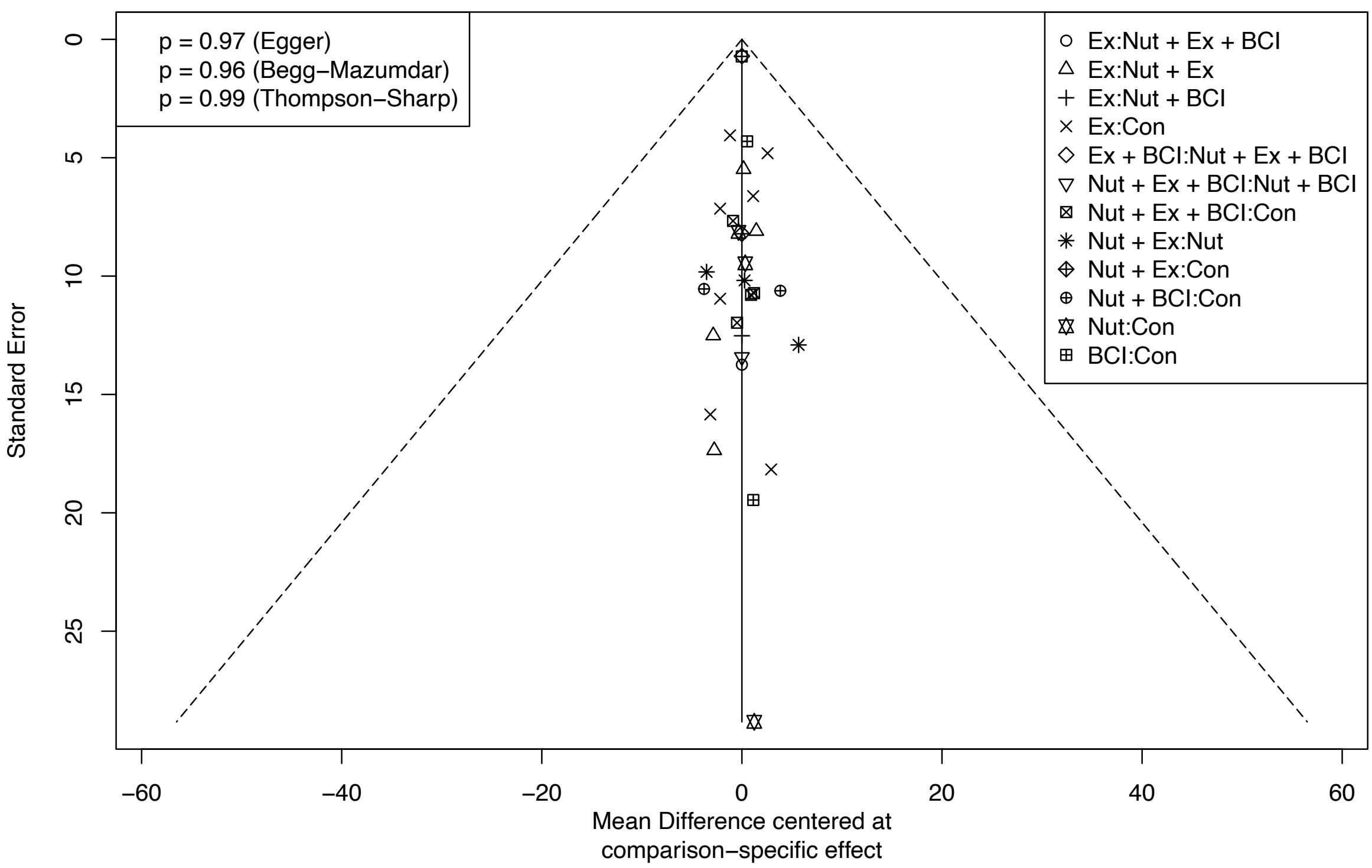

Figure S22: Funnel plot for waist-circumference (NMA on intervention type)

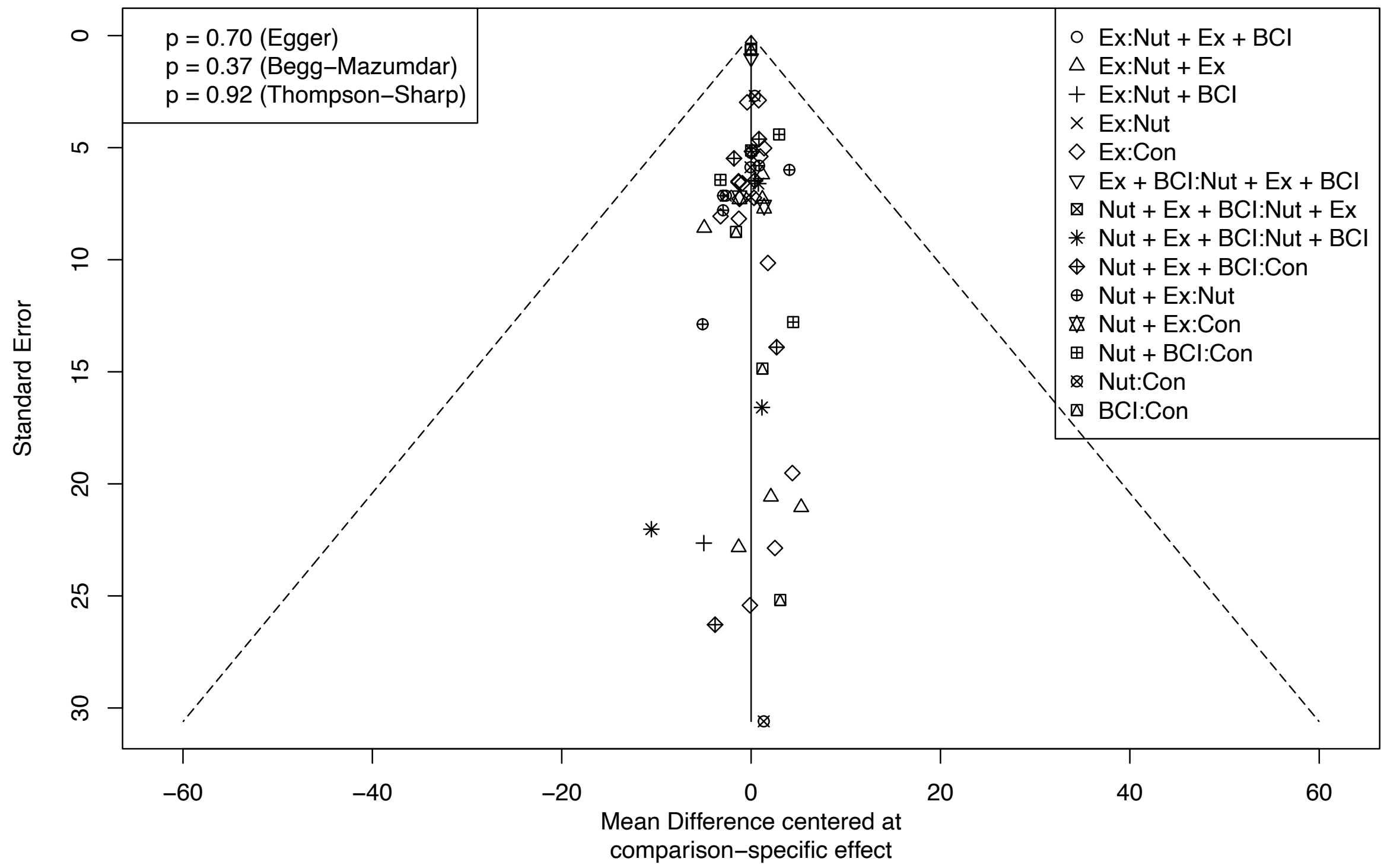

Figure S23: Funnel plot for body weight (NMA on intervention type)

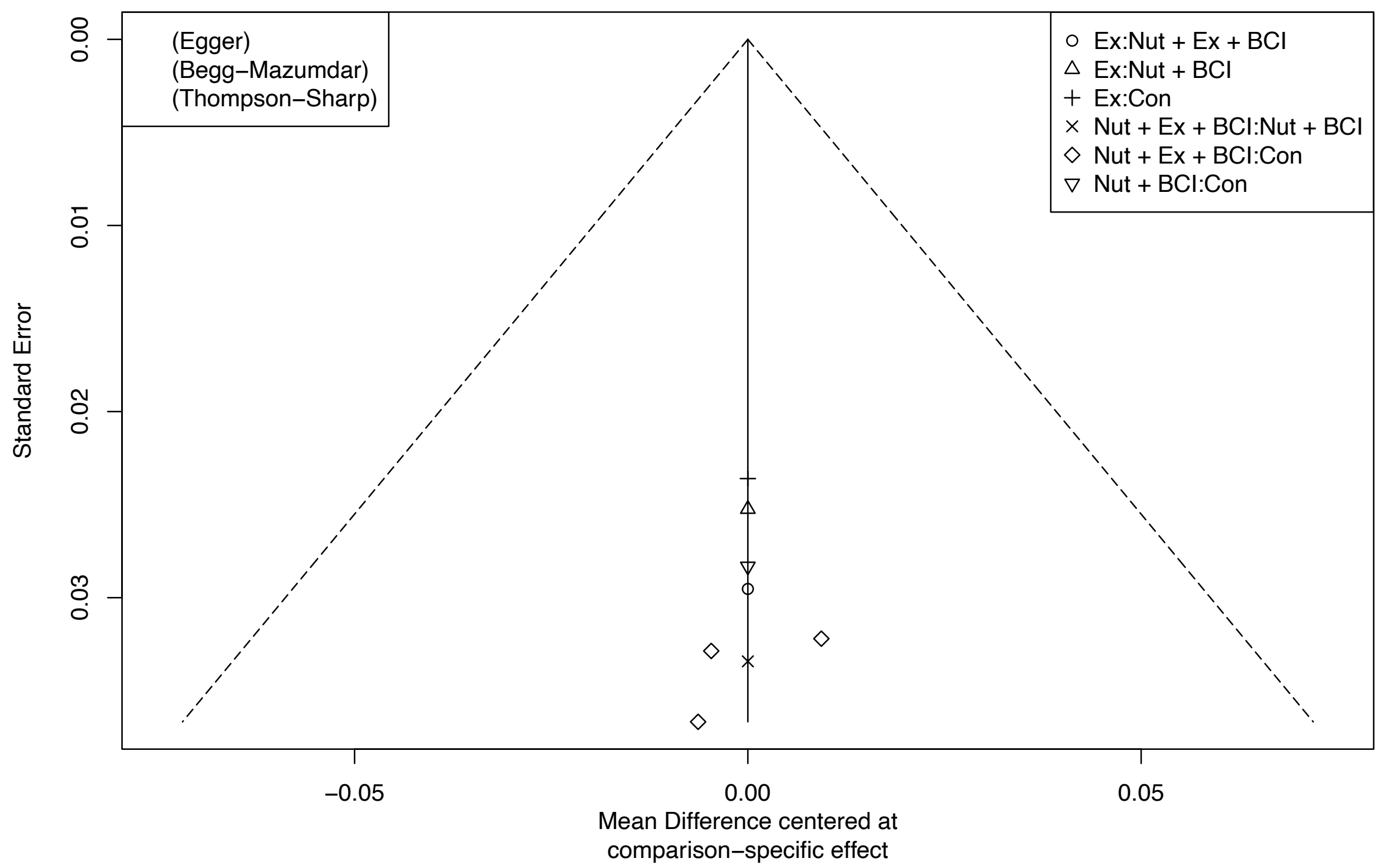

Figure S24: Funnel plot for bone mineral density of the total hip (NMA on intervention type)

Supplemental tables

Table S1 A-F: Search Strategies

A. Database(s): Ovid MEDLINE(R) ALL 1946 to October 13, 2025 (medall)
Search date: 2025-10-14

#	Search
1	obesity/
2	obesity, morbid/
3	obesity, abdominal/
4	obesity, metabolically benign/
5	overweight/
6	(adipos* or obes* or overweight* or over weight*).ti,ab,kf.
7	or/1-6 [Obesity]
8	exp behavior therapy/
9	exp counseling/
10	social support/
11	program evaluation/
12	exp exercise/
13	exp exercise therapy/
14	exp "physical education and training"/
15	exp exercise movement techniques/
16	exp physical fitness/
17	accidental falls/
18	postural balance/
19	motor activity/
20	gait/
21	exp muscle strength/
22	exp diet/
23	exp diet therapy/
24	fasting/
25	food preferences/
26	patient education as topic/
27	health education/
28	exp health behavior/
29	exp health promotion/
30	life style/
31	((obesity adj3 intervention) or program or programme).ti,ab,kf.
32	(lifestyle or life style).ti,ab,kf.

33	exercis*.ti,ab,kf.
34	(physic* adj (activ* or fit*)).ti,ab,kf.
35	(walk* or swim* or aqua or cycl* or weight lift* or danc* or aerobics or balance or electric* muscle stimulation or neuromuscular electric* stimulation or electromyostimulation or ems).ti,ab,kf.
36	((physic* or strength* or resist* or circuit or weight or aerob* or cross or endurance or vibration*) adj3 train*).ti,ab,kf.
37	(behavio?ral or behavio?r modification* or psychoth* or psychosocial).ti,ab,kf.
38	((group or cognit* or behav*) adj therap*).ti,ab,kf.
39	counsel?ing.ti,ab,kf.
40	educat*.ti,ab,kf.
41	(diet* or healthy nutrition or (nutrition* adj (knowledge or educat* or therap* or program* or intervention*))).ti,ab,kf.
42	(low-calori* or calori* restrict* or low-energ* or energ* restrict* or low-carbohydrate or high-carbohydrate or low-fat or high-fat or low-protein or high-protein or vegetarian or vegan or mediterranean or dash).ti,ab,kf.
43	weight loss/
44	(weight adj1 (reduc* or los* or control* or manag*)).ti,ab,kf.
45	or/8-44 [Interventions]
46	obesity/ or obesity, abdominal/ or obesity, morbid/ or overweight/ or obesity, metabolically benign/
47	(diet therapy or prevention & control or rehabilitation or therapy or psychology).fs.
48	46 and 47 [MeSH/subheading combination]
49	exp weight reduction programs/
50	or/48-49 [Obesity-specific interventions]
51	7 and 45 [Obesity AND Interventions]
52	or/50-51 [Obesity interventions complete: (Obesity AND Interventions) OR Obesity-specific interventions]
53	exp aged/
54	geriatrics/
55	frailty/
56	sarcopenia/
57	Independent Living/
58	((old* or senior or age*) adj2 (adult* or person* or subject* or individual* or people or group* or m?n or wom?n or patient* or client* or outpatient* or citizen* or "60" or "65" or age*)).ti,ab,kf.
59	(elder* or eldes* or geriat* or geronto* or frail* or sarcopen* or senior* or agedly or ag?ing).ti,ab,kf.
60	community-dwell*.ti,ab,kf.
61	((commun* or indepen*) adj1 (dwell* or livi*)).ti,ab,kf.
62	(("60" or "65") adj1 year*).ti,ab,kf.

63	((advanc* or high*) adj1 age*).ti,ab,kf.
64	(super adj1 age*).ti,ab,kf.
65	(superage* or super-age* or centen* or centa* or nonagen* or octagen* or sept?agen* or supercenten* or senium*).ti,ab,kf.
66	or/53-65 [Age group]
67	randomized controlled trial.pt.
68	controlled clinical trial.pt.
69	randomi?ed.ab.
70	placebo.ab.
71	clinical trials as topic.sh.
72	randomly.ab.
73	trial.ti.
74	or/67-73
75	exp animals/ not humans.sh.
76	74 not 75 [Study filter (Cochrane Handbook v5.0.2 2008 RCT filter - sensitivity and precision maximizing version)]
77	52 and 66 [Obesity interventions complete AND Age group]
78	77 and 76 [Obesity interventions complete AND Age group AND Study filter]
79	(exp infant/ or exp child/ or adolescent/ or young adult/ or adult/ or middle aged/) not exp aged/ [Child/adolescent/middle aged adults-only studies]
80	78 not 79 [Remove Child/adolescent/middle aged adults-only studies]

B. Database(s): Embase <1974 to 2022 May 13> (oemezd) via Ovid
Search date: 2022-05-16

1	obesity/
2	morbid obesity/
3	abdominal obesity/
4	metabolically benign obesity/
5	(adipos* or obes* or overweight* or over weight*).ti,ab,kw.
6	or/1-5 [Obesity]
7	behavior therapy/
8	cognitive therapy/
9	exp counseling/
10	social support/
11	exp program evaluation/
12	exp exercise/
13	exp physical education/
14	exp physical activity/
15	exp motor activity/
16	training/
17	fitness/
18	falling/
19	body equilibrium/
20	gait/
21	muscle strength/
22	exp diet/
23	exp diet therapy/
24	nutritional health/
25	feeding behavior/
26	patient education/
27	health promotion/
28	health literacy/
29	nutrition education/
30	health education/
31	lifestyle/
32	lifestyle modification/
33	((obesity adj3 intervention) or program or programme).ti,ab,kw.
34	(lifestyle or life style).ti,ab,kw.
35	exercis*.ti,ab,kw.
36	(physic* adj (activ* or fit*)).ti,ab,kw.
37	(walk* or swim* or aqua or cycl* or weight lift* or danc* or aerobics or balance or electric* muscle stimulation or neuromuscular electric* stimulation or electromyostimulation or ems).ti,ab,kw.
38	((physic* or strength* or resist* or circuit or weight or aerob* or cross or endurance or vibration*) adj3 train*).ti,ab,kw.
39	(behavio?ral or behavio?r modification or psychoth* or psychosocial).ti,ab,kw.
40	((group or cognit* or behav*) adj therap*).ti,ab,kw.
41	counsel?ing.ti,ab,kw.
42	educat*.ti,ab,kw.
43	(diet* or healthy nutrition or (nutrition* adj (knowledge or educat* or therap* or program* or intervention*))).ti,ab,kw.

44	(low-calori* or calori* restrict* or low-energ* or energ* restrict* or low-carbohydrate or high-carbohydrate or low-fat or high-fat or low-protein or high-protein or vegetarian or vegan or mediterranean or dash).ti,ab,kw.
45	weight reduction/
46	weight control/
47	(weight adj1 (reduc* or los* or control* or manag*)).ti,ab,kw.
48	or/7-47 [Interventions]
49	obesity/ or morbid obesity/ or abdominal obesity/ or metabolically benign obesity/
50	(pc or rh or th).fs. [prevention.fs. or rehabilitation.fs. or therapy.fs.]
51	49 and 50 [EMTREE/subheading combination]
52	exp body weight management/
53	or/51-52 [Obesity-specific interventions]
54	6 and 48 [Obesity AND Interventions]
55	or/53-54 [Obesity interventions complete: (Obesity AND Interventions) OR Obesity-specific interventions]
56	aged/
57	very elderly/
58	frail elderly/
59	geriatrics/
60	frailty/
61	sarcopenia/
62	sarcopenic obesity/
63	independent living/
64	((old* or senior or age*) adj2 (adult* or person* or subject* or individual* or people or group* or m?n or wom?n or patient* or client* or outpatient* or citizen* or "60" or "65" or age*)).ti,ab,kw.
65	(elder* or eldes* or geriat* or geronto* or frail* or sarcopen* or senior* or agedly or ag?ing).ti,ab,kw.
66	community-dwell*.ti,ab,kw.
67	((commun* or indepen*) adj1 (dwell* or livi*)).ti,ab,kw.
68	(("60" or "65") adj1 year*).ti,ab,kw.
69	((advanc* or high*) adj1 age*).ti,ab,kw.
70	(super adj1 age*).ti,ab,kw.
71	(superage* or super-age* or centen* or centa* or nonagen* or octagen* or sept?agen* or supercenten* or senium*).ti,ab,kw.
72	or/56-71 [Age group]
73	random*.tw. or clinical trial*.mp. or exp treatment outcome/
74	55 and 72 [Obesity interventions complete AND Age group]
75	74 and 73 [Obesity interventions complete AND Age group AND Study filter]
76	(exp juvenile/ or young adult/ or adult/ or middle aged/) not (aged/ or very elderly/ or frail elderly/)
77	75 not 76
78	limit 77 to embase

C. Database(s): CINAHL via EBSCOhost
Search date: 2022-05-21

#	Query	Limiters/ Expanders	Last Run Via
S1	MH obesity	Expanders - Apply equivalent subjects<br>Search modes - Boolean/Phrase	Interface - EBSCOhost Research Databases<br>Search Screen - Advanced Search<br>Database - CINAHL
S2	MH "obesity, morbid"	Expanders - Apply equivalent subjects<br>Search modes - Boolean/Phrase	Interface - EBSCOhost Research Databases<br>Search Screen - Advanced Search<br>Database – CINAHL
S3	TX (adipos* OR obes* OR overweight* OR "over weight*")	Expanders - Apply equivalent subjects<br>Search modes - Boolean/Phrase	Interface - EBSCOhost Research Databases<br>Search Screen - Advanced Search<br>Database – CINAHL
S4	S1 OR S2 OR S3	Expanders - Apply equivalent subjects<br>Search modes - Boolean/Phrase	Interface - EBSCOhost Research Databases<br>Search Screen - Advanced Search<br>Database – CINAHL
S5	MH "weight loss"	Expanders - Apply equivalent subjects<br>Search modes - Boolean/Phrase	Interface - EBSCOhost Research Databases<br>Search Screen - Advanced Search<br>Database – CINAHL
S6	(MH "Behavior Modification+")	Expanders - Apply equivalent subjects<br>Search modes - Boolean/Phrase	Interface - EBSCOhost Research Databases<br>Search Screen - Advanced Search<br>Database – CINAHL
S7	MH Counseling	Expanders - Apply equivalent subjects<br>Search modes - Boolean/Phrase	Interface - EBSCOhost Research Databases<br>Search Screen - Advanced Search<br>Database – CINAHL
S8	MH "Family Therapy"	Expanders - Apply equivalent subjects<br>Search modes - Boolean/Phrase	Interface - EBSCOhost Research Databases<br>Search Screen - Advanced Search<br>Database – CINAHL
S9	(MH "Support, Psychosocial")	Expanders - Apply equivalent subjects	Interface - EBSCOhost Research Databases

		Search modes - Boolean/Phrase	Search Screen - Advanced Search<br>Database – CINAHL
S10	MH "support groups"	Expanders - Apply equivalent subjects<br>Search modes - Boolean/Phrase	Interface - EBSCOhost Research Databases<br>Search Screen - Advanced Search<br>Database – CINAHL
S11	(MH "Program Evaluation")	Expanders - Apply equivalent subjects<br>Search modes - Boolean/Phrase	Interface - EBSCOhost Research Databases<br>Search Screen - Advanced Search<br>Database – CINAHL
S12	(MH "Program Implementation")	Expanders - Apply equivalent subjects<br>Search modes - Boolean/Phrase	Interface - EBSCOhost Research Databases<br>Search Screen - Advanced Search<br>Database – CINAHL
S13	(MH "Exercise+")	Expanders - Apply equivalent subjects<br>Search modes - Boolean/Phrase	Interface - EBSCOhost Research Databases<br>Search Screen - Advanced Search<br>Database – CINAHL
S14	(MH "Sports+")	Expanders - Apply equivalent subjects<br>Search modes - Boolean/Phrase	Interface - EBSCOhost Research Databases<br>Search Screen - Advanced Search<br>Database – CINAHL
S15	(MH "Therapeutic Exercise+")	Expanders - Apply equivalent subjects<br>Search modes - Boolean/Phrase	Interface - EBSCOhost Research Databases<br>Search Screen - Advanced Search<br>Database – CINAHL
S16	(MH "Physical Fitness")	Expanders - Apply equivalent subjects<br>Search modes - Boolean/Phrase	Interface - EBSCOhost Research Databases<br>Search Screen - Advanced Search<br>Database – CINAHL
S17	(MH "Physical Education and Training+")	Expanders - Apply equivalent subjects<br>Search modes - Boolean/Phrase	Interface - EBSCOhost Research Databases<br>Search Screen - Advanced Search<br>Database – CINAHL
S18	(MH "Accidental Falls")	Expanders - Apply equivalent subjects	Interface - EBSCOhost Research Databases

		Search modes - Boolean/Phrase	Search Screen - Advanced Search<br>Database – CINAHL
S19	(MH "Balance, Postural")	Expanders - Apply equivalent subjects<br>Search modes - Boolean/Phrase	Interface - EBSCOhost Research Databases<br>Search Screen - Advanced Search<br>Database - CINAHL
S20	(MH "Motor Activity")	Expanders - Apply equivalent subjects<br>Search modes - Boolean/Phrase	Interface - EBSCOhost Research Databases<br>Search Screen - Advanced Search<br>Database – CINAHL
S21	(MH "Gait+")	Expanders - Apply equivalent subjects<br>Search modes - Boolean/Phrase	Interface - EBSCOhost Research Databases<br>Search Screen - Advanced Search<br>Database – CINAHL
S22	(MH "Muscle Strength+")	Expanders - Apply equivalent subjects<br>Search modes - Boolean/Phrase	Interface - EBSCOhost Research Databases<br>Search Screen - Advanced Search<br>Database – CINAHL
S23	(MH "Diet+")	Expanders - Apply equivalent subjects<br>Search modes - Boolean/Phrase	Interface - EBSCOhost Research Databases<br>Search Screen - Advanced Search<br>Database – CINAHL
S24	(MH "Diet Therapy+")	Expanders - Apply equivalent subjects<br>Search modes - Boolean/Phrase	Interface - EBSCOhost Research Databases<br>Search Screen - Advanced Search<br>Database – CINAHL
S25	(MH "Health Behavior")	Expanders - Apply equivalent subjects<br>Search modes - Boolean/Phrase	Interface - EBSCOhost Research Databases<br>Search Screen - Advanced Search<br>Database – CINAHL
S26	(MH "Eating Behavior")	Expanders - Apply equivalent subjects<br>Search modes - Boolean/Phrase	Interface - EBSCOhost Research Databases<br>Search Screen - Advanced Search<br>Database – CINAHL
S27	(MH "Health Promotion")	Expanders - Apply equivalent subjects	Interface - EBSCOhost Research Databases

		Search modes - Boolean/Phrase	Search Screen - Advanced Search<br>Database – CINAHL
S28	(MH "Patient Education")	Expanders - Apply equivalent subjects<br>Search modes - Boolean/Phrase	Interface - EBSCOhost Research Databases<br>Search Screen - Advanced Search<br>Database – CINAHL
S29	(MH "Life style changes")	Expanders - Apply equivalent subjects<br>Search modes - Boolean/Phrase	Interface - EBSCOhost Research Databases<br>Search Screen - Advanced Search<br>Database – CINAHL
S30	(MH "Life style")	Expanders - Apply equivalent subjects<br>Search modes - Boolean/Phrase	Interface - EBSCOhost Research Databases<br>Search Screen - Advanced Search<br>Database – CINAHL
S31	TX (weight N1 (reduc* OR los* OR control* OR manag*))	Expanders - Apply equivalent subjects<br>Search modes - Boolean/Phrase	Interface - EBSCOhost Research Databases<br>Search Screen - Advanced Search<br>Database – CINAHL
S32	TX ((obesity N3 intervention) OR program OR programme)	Expanders - Apply equivalent subjects<br>Search modes - Boolean/Phrase	Interface - EBSCOhost Research Databases<br>Search Screen - Advanced Search<br>Database – CINAHL
S33	TX (lifestyle OR "life style")	Expanders - Apply equivalent subjects<br>Search modes - Boolean/Phrase	Interface - EBSCOhost Research Databases<br>Search Screen - Advanced Search<br>Database – CINAHL
S34	TX (exercis*)	Expanders - Apply equivalent subjects<br>Search modes - Boolean/Phrase	Interface - EBSCOhost Research Databases<br>Search Screen - Advanced Search<br>Database – CINAHL
S35	TX (physic* N1 (activ* OR fit*))	Expanders - Apply equivalent subjects<br>Search modes - Boolean/Phrase	Interface - EBSCOhost Research Databases<br>Search Screen - Advanced Search<br>Database – CINAHL
S36	TX (walk* OR swim* OR aqua OR cycl* OR "weight lift*" OR danc*	Expanders - Apply equivalent subjects	Interface - EBSCOhost Research Databases

	OR aerobics OR balance OR "electric* muscle stimulation" OR "neuromuscular electric* stimulation" OR electromyostimulation OR ems)	Search modes - Boolean/Phrase	Search Screen - Advanced Search<br>Database – CINAHL
S37	TX ((physic* OR strength* OR resist* OR circuit OR weight OR aerob* OR cross OR endurance OR vibration*) N3 train*)	Expanders - Apply equivalent subjects<br>Search modes - Boolean/Phrase	Interface - EBSCOhost Research Databases<br>Search Screen - Advanced Search<br>Database – CINAHL
S38	TX (behavioral OR behavioural OR "behavior modification*" OR "behaviour modification*" OR psychoth* OR psychosocial)	Expanders - Apply equivalent subjects<br>Search modes - Boolean/Phrase	Interface - EBSCOhost Research Databases<br>Search Screen - Advanced Search<br>Database – CINAHL
S39	TX ((group OR cognit* OR behav*) N1 therap*)	Expanders - Apply equivalent subjects<br>Search modes - Boolean/Phrase	Interface - EBSCOhost Research Databases<br>Search Screen - Advanced Search<br>Database – CINAHL
S40	TX (counseling OR counselling)	Expanders - Apply equivalent subjects<br>Search modes - Boolean/Phrase	Interface - EBSCOhost Research Databases<br>Search Screen - Advanced Search<br>Database – CINAHL
S41	TX (educat*)	Expanders - Apply equivalent subjects<br>Search modes - Boolean/Phrase	Interface - EBSCOhost Research Databases<br>Search Screen - Advanced Search<br>Database – CINAHL
S42	TX (diet* OR "healthy nutrition" OR (nutrition* N1 (knowledge OR educat* OR therap* OR program* OR intervention*)))	Expanders - Apply equivalent subjects<br>Search modes - Boolean/Phrase	Interface - EBSCOhost Research Databases<br>Search Screen - Advanced Search<br>Database – CINAHL
S43	S5 OR S6 OR S7 OR S8 OR S9 OR S10 OR S11 OR S12 OR S13 OR S14 OR S15 OR S16 OR S17 OR S18 OR S19 OR S20 OR S21 OR S22 OR S23 OR S24 OR S25 OR S26 OR S27 OR S28 OR S29 OR S30 OR S31 OR S32 OR S33 OR S34 OR S35 OR S36 OR S37 OR S38 OR S39 OR S40 OR S41 OR S42	Expanders - Apply equivalent subjects<br>Search modes - Boolean/Phrase	Interface - EBSCOhost Research Databases<br>Search Screen - Advanced Search<br>Database – CINAHL

S44	S4 AND S43	Expanders - Apply equivalent subjects<br>Search modes - Boolean/Phrase	Interface - EBSCOhost Research Databases<br>Search Screen - Advanced Search<br>Database – CINAHL
S45	(MH "Obesity+/DH/ED/PC/PF/RH/TH") [diet therapy or education or prevention & control or psychosocial factors or rehabilitation or therapy]	Expanders - Apply equivalent subjects<br>Search modes - Boolean/Phrase	Interface - EBSCOhost Research Databases<br>Search Screen - Advanced Search<br>Database – CINAHL
S46	S44 OR S45	Expanders - Apply equivalent subjects<br>Search modes - Boolean/Phrase	Interface - EBSCOhost Research Databases<br>Search Screen - Advanced Search<br>Database – CINAHL
S47	(MH "Aged+")	Expanders - Apply equivalent subjects<br>Search modes - Boolean/Phrase	Interface - EBSCOhost Research Databases<br>Search Screen - Advanced Search<br>Database – CINAHL
S48	(MH "Geriatrics")	Expanders - Apply equivalent subjects<br>Search modes - Boolean/Phrase	Interface - EBSCOhost Research Databases<br>Search Screen - Advanced Search<br>Database - CINAHL
S49	(MH "Frailty Syndrome")	Expanders - Apply equivalent subjects<br>Search modes - Boolean/Phrase	Interface - EBSCOhost Research Databases<br>Search Screen - Advanced Search<br>Database – CINAHL
S50	(MH "Sarcopenia")	Expanders - Apply equivalent subjects<br>Search modes - Boolean/Phrase	Interface - EBSCOhost Research Databases<br>Search Screen - Advanced Search<br>Database – CINAHL
S51	(MH "Community Living")	Expanders - Apply equivalent subjects<br>Search modes - Boolean/Phrase	Interface - EBSCOhost Research Databases<br>Search Screen - Advanced Search<br>Database – CINAHL
S52	(MH "Assisted Living")	Expanders - Apply equivalent subjects<br>Search modes - Boolean/Phrase	Interface - EBSCOhost Research Databases<br>Search Screen - Advanced Search

			Database - CINAHL
S53	TX ((old* OR senior OR age*) N2 (adult* OR person* OR subject* OR individual* OR people OR group* OR man OR men OR woman OR women OR patient* OR client* OR outpatient* OR citizen* OR age* OR 60 OR 65)))	Expanders - Apply equivalent subjects<br>Search modes - Boolean/Phrase	Interface - EBSCOhost Research Databases<br>Search Screen - Advanced Search<br>Database – CINAHL
S54	TX (elder* OR eldes* OR geriat* OR geronto* OR frail* OR sarcopen* OR senior* OR agedly OR aging OR ageing)	Expanders - Apply equivalent subjects<br>Search modes - Boolean/Phrase	Interface - EBSCOhost Research Databases<br>Search Screen - Advanced Search<br>Database – CINAHL
S55	TX (community-dwell*)	Expanders - Apply equivalent subjects<br>Search modes - Boolean/Phrase	Interface - EBSCOhost Research Databases<br>Search Screen - Advanced Search<br>Database – CINAHL
S56	TX ((commun* OR indepen*) N1 (dwell* OR livi*)))	Expanders - Apply equivalent subjects<br>Search modes - Boolean/Phrase	Interface - EBSCOhost Research Databases<br>Search Screen - Advanced Search<br>Database – CINAHL
S57	TX ((60 OR 65) N1 year*))	Expanders - Apply equivalent subjects<br>Search modes - Boolean/Phrase	Interface - EBSCOhost Research Databases<br>Search Screen - Advanced Search<br>Database - CINAHL
S58	TX ((advanc* OR high*) N1 age*))	Expanders - Apply equivalent subjects<br>Search modes - Boolean/Phrase	Interface - EBSCOhost Research Databases<br>Search Screen - Advanced Search<br>Database – CINAHL
S59	TX (super N1 age*)	Expanders - Apply equivalent subjects<br>Search modes - Boolean/Phrase	Interface - EBSCOhost Research Databases<br>Search Screen - Advanced Search<br>Database – CINAHL
S60	TX (superage* OR super-age* OR centen* OR centa* OR nonagen* OR octagen* OR septuagen* OR septagen* OR supercenten* OR senium*)	Expanders - Apply equivalent subjects<br>Search modes - Boolean/Phrase	Interface - EBSCOhost Research Databases<br>Search Screen - Advanced Search<br>Database – CINAHL
S61	S47 OR S48 OR S49 OR S50 OR S51 OR S52 OR S53 OR S54 OR	Expanders - Apply equivalent subjects	Interface - EBSCOhost Research Databases

	S55 OR S56 OR S57 OR S58 OR S59 OR S60	Search modes - Boolean/Phrase	Search Screen - Advanced Search<br>Database – CINAHL
S62	MH randomized controlled trials	Expanders - Apply equivalent subjects<br>Search modes - Boolean/Phrase	Interface - EBSCOhost Research Databases<br>Search Screen - Advanced Search<br>Database – CINAHL
S63	MH double-blind studies	Expanders - Apply equivalent subjects<br>Search modes - Boolean/Phrase	Interface - EBSCOhost Research Databases<br>Search Screen - Advanced Search<br>Database – CINAHL
S64	MH single-blind studies	Expanders - Apply equivalent subjects<br>Search modes - Boolean/Phrase	Interface - EBSCOhost Research Databases<br>Search Screen - Advanced Search<br>Database – CINAHL
S65	MH random assignment	Expanders - Apply equivalent subjects<br>Search modes - Boolean/Phrase	Interface - EBSCOhost Research Databases<br>Search Screen - Advanced Search<br>Database – CINAHL
S66	MH pretest-posttest design	Expanders - Apply equivalent subjects<br>Search modes - Boolean/Phrase	Interface - EBSCOhost Research Databases<br>Search Screen - Advanced Search<br>Database – CINAHL
S67	MH cluster sample	Expanders - Apply equivalent subjects<br>Search modes - Boolean/Phrase	Interface - EBSCOhost Research Databases<br>Search Screen - Advanced Search<br>Database – CINAHL
S68	TI (randomised OR randomized)	Expanders - Apply equivalent subjects<br>Search modes - Boolean/Phrase	Interface - EBSCOhost Research Databases<br>Search Screen - Advanced Search<br>Database – CINAHL
S69	AB (random*)	Expanders - Apply equivalent subjects<br>Search modes - Boolean/Phrase	Interface - EBSCOhost Research Databases<br>Search Screen - Advanced Search<br>Database – CINAHL
S70	TI (trial)	Expanders - Apply equivalent subjects	Interface - EBSCOhost Research Databases

		Search modes - Boolean/Phrase	Search Screen - Advanced Search<br>Database – CINAHL
S71	MH (sample size) AND AB (assigned OR allocated OR control)	Expanders - Apply equivalent subjects<br>Search modes - Boolean/Phrase	Interface - EBSCOhost Research Databases<br>Search Screen - Advanced Search<br>Database - CINAHL
S72	MH (placebos)	Expanders - Apply equivalent subjects<br>Search modes - Boolean/Phrase	Interface - EBSCOhost Research Databases<br>Search Screen - Advanced Search<br>Database – CINAHL
S73	PT (randomized controlled trial)	Expanders - Apply equivalent subjects<br>Search modes - Boolean/Phrase	Interface - EBSCOhost Research Databases<br>Search Screen - Advanced Search<br>Database – CINAHL
S74	AB (control W5 group)	Expanders - Apply equivalent subjects<br>Search modes - Boolean/Phrase	Interface - EBSCOhost Research Databases<br>Search Screen - Advanced Search<br>Database – CINAHL
S75	MH (crossover design) OR MH (comparative studies)	Expanders - Apply equivalent subjects<br>Search modes - Boolean/Phrase	Interface - EBSCOhost Research Databases<br>Search Screen - Advanced Search<br>Database – CINAHL
S76	AB (cluster W3 RCT)	Expanders - Apply equivalent subjects<br>Search modes - Boolean/Phrase	Interface - EBSCOhost Research Databases<br>Search Screen - Advanced Search<br>Database – CINAHL
S77	MH "Animals+"	Expanders - Apply equivalent subjects<br>Search modes - Boolean/Phrase	Interface - EBSCOhost Research Databases<br>Search Screen - Advanced Search<br>Database – CINAHL
S78	MH (animal studies)	Expanders - Apply equivalent subjects<br>Search modes - Boolean/Phrase	Interface - EBSCOhost Research Databases<br>Search Screen - Advanced Search<br>Database – CINAHL
S79	TI (animal model*)	Expanders - Apply equivalent subjects	Interface - EBSCOhost Research Databases

		Search modes - Boolean/Phrase	Search Screen - Advanced Search<br>Database – CINAHL
S80	S77 OR S78 OR S79	Expanders - Apply equivalent subjects<br>Search modes - Boolean/Phrase	Interface - EBSCOhost Research Databases<br>Search Screen - Advanced Search<br>Database – CINAHL
S81	MH (human)	Expanders - Apply equivalent subjects<br>Search modes - Boolean/Phrase	Interface - EBSCOhost Research Databases<br>Search Screen - Advanced Search<br>Database – CINAHL
S82	S80 NOT S81	Expanders - Apply equivalent subjects<br>Search modes - Boolean/Phrase	Interface - EBSCOhost Research Databases<br>Search Screen - Advanced Search<br>Database – CINAHL
S83	S62 OR S63 OR S64 OR S65 OR S66 OR S67 OR S68 OR S69 OR S70 OR S71 OR S72 OR S73 OR S74 OR S75 OR S76	Expanders - Apply equivalent subjects<br>Search modes - Boolean/Phrase	Interface - EBSCOhost Research Databases<br>Search Screen - Advanced Search<br>Database – CINAHL
S84	S83 NOT S82	Expanders - Apply equivalent subjects<br>Search modes - Boolean/Phrase	Interface - EBSCOhost Research Databases<br>Search Screen - Advanced Search<br>Database – CINAHL
S85	S4 AND S46 AND S61 AND S84	Expanders - Apply equivalent subjects<br>Search modes - Boolean/Phrase	Interface - EBSCOhost Research Databases<br>Search Screen - Advanced Search<br>Database – CINAHL
S86	((MH "Child+") OR (MH "Adolescence+") OR (MH "Minors (Legal)") OR (MH "Young Adult") OR (MH "Adult") OR (MH "Middle Age")) NOT (MH "Aged+")	Expanders - Apply equivalent subjects<br>Search modes - Boolean/Phrase	Interface - EBSCOhost Research Databases<br>Search Screen - Advanced Search<br>Database - CINAHL
S87	S85 NOT S86	Expanders - Apply equivalent subjects<br>Search modes - Boolean/Phrase	Interface - EBSCOhost Research Databases<br>Search Screen - Advanced Search<br>Database - CINAHL

D. Database(s): APA PsycInfo (psyh) via EBSCOhost
Search date: 2022-05-23

#	Query	Limiters/ Expanders	Last Run Via
S1	DE "Overweight"	Expanders - Apply equivalent subjects Search modes - Boolean/Phrase	Interface - EBSCOhost Research Databases Search Screen - Advanced Search Database - APA PsycInfo
S2	DE "Obesity"	Expanders - Apply equivalent subjects Search modes - Boolean/Phrase	Interface - EBSCOhost Research Databases Search Screen - Advanced Search Database - APA PsycInfo
S3	TX (adipos* OR obes* OR overweight* OR "over weight*")	Expanders - Apply equivalent subjects Search modes - Boolean/Phrase	Interface - EBSCOhost Research Databases Search Screen - Advanced Search Database - APA PsycInfo
S4	S1 OR S2 OR S3	Expanders - Apply equivalent subjects Search modes - Boolean/Phrase	Interface - EBSCOhost Research Databases Search Screen - Advanced Search Database - APA PsycInfo
S5	DE "Weight Control"	Expanders - Apply equivalent subjects Search modes - Boolean/Phrase	Interface - EBSCOhost Research Databases Search Screen - Advanced Search Database - APA PsycInfo
S6	DE "Weight Loss"	Expanders - Apply equivalent subjects Search modes - Boolean/Phrase	Interface - EBSCOhost Research Databases Search Screen - Advanced Search Database - APA PsycInfo
S7	DE "Diets"	Expanders - Apply equivalent subjects Search modes - Boolean/Phrase	Interface - EBSCOhost Research Databases Search Screen - Advanced Search Database - APA PsycInfo
S8	DE "Movement Therapy"	Expanders - Apply equivalent subjects Search modes - Boolean/Phrase	Interface - EBSCOhost Research Databases Search Screen - Advanced Search Database - APA PsycInfo
S9	DE "Dance Therapy"	Expanders - Apply equivalent subjects Search modes - Boolean/Phrase	Interface - EBSCOhost Research Databases Search Screen - Advanced Search Database - APA PsycInfo
S10	DE "Physical Activity" OR DE "Actigraphy" OR DE "Exercise" OR DE "Aerobic Exercise" OR DE "Weightlifting" OR DE "Yoga"	Expanders - Apply equivalent subjects Search modes - Boolean/Phrase	Interface - EBSCOhost Research Databases Search Screen - Advanced Search Database - APA PsycInfo

S11	DE "Physical Fitness"	Expanders - Apply equivalent subjects<br>Search modes - Boolean/Phrase	Interface - EBSCOhost Research Databases<br>Search Screen - Advanced Search<br>Database - APA PsycInfo
S12	DE "Health Behavior"	Expanders - Apply equivalent subjects<br>Search modes - Boolean/Phrase	Interface - EBSCOhost Research Databases<br>Search Screen - Advanced Search<br>Database - APA PsycInfo
S13	DE "Health Promotion"	Expanders - Apply equivalent subjects<br>Search modes - Boolean/Phrase	Interface - EBSCOhost Research Databases<br>Search Screen - Advanced Search<br>Database - APA PsycInfo
S14	DE "Health Knowledge"	Expanders - Apply equivalent subjects<br>Search modes - Boolean/Phrase	Interface - EBSCOhost Research Databases<br>Search Screen - Advanced Search<br>Database - APA PsycInfo
S15	DE "Health Literacy"	Expanders - Apply equivalent subjects<br>Search modes - Boolean/Phrase	Interface - EBSCOhost Research Databases<br>Search Screen - Advanced Search<br>Database - APA PsycInfo
S16	DE "Health Education"	Expanders - Apply equivalent subjects<br>Search modes - Boolean/Phrase	Interface - EBSCOhost Research Databases<br>Search Screen - Advanced Search<br>Database - APA PsycInfo
S17	DE "Client Education"	Expanders - Apply equivalent subjects<br>Search modes - Boolean/Phrase	Interface - EBSCOhost Research Databases<br>Search Screen - Advanced Search<br>Database - APA PsycInfo
S18	DE "Lifestyle"	Expanders - Apply equivalent subjects<br>Search modes - Boolean/Phrase	Interface - EBSCOhost Research Databases<br>Search Screen - Advanced Search<br>Database - APA PsycInfo
S19	DE "Physical Education"	Expanders - Apply equivalent subjects<br>Search modes - Boolean/Phrase	Interface - EBSCOhost Research Databases<br>Search Screen - Advanced Search<br>Database - APA PsycInfo
S20	DE "Program Evaluation" OR DE "Educational Program Evaluation" OR DE "Mental Health Program Evaluation"	Expanders - Apply equivalent subjects<br>Search modes - Boolean/Phrase	Interface - EBSCOhost Research Databases<br>Search Screen - Advanced Search<br>Database - APA PsycInfo
S21	DE "Educational Programs"	Expanders - Apply equivalent subjects<br>Search modes - Boolean/Phrase	Interface - EBSCOhost Research Databases<br>Search Screen - Advanced Search<br>Database - APA PsycInfo

S22	DE "Educational Therapy"	Expanders - Apply equivalent subjects<br>Search modes - Boolean/Phrase	Interface - EBSCOhost Research Databases<br>Search Screen - Advanced Search<br>Database - APA PsycInfo
S23	DE "Program Development" OR DE "Educational Program Planning"	Expanders - Apply equivalent subjects<br>Search modes - Boolean/Phrase	Interface - EBSCOhost Research Databases<br>Search Screen - Advanced Search<br>Database - APA PsycInfo
S24	DE "Counseling" OR DE "Community Counseling" OR DE "Educational Counseling" OR DE "Gerontological Counseling" OR DE "Group Counseling" OR DE "Occupational Guidance" OR DE "Peer Counseling" OR DE "Psychotherapeutic Counseling" OR DE "Rehabilitation Counseling"	Expanders - Apply equivalent subjects<br>Search modes - Boolean/Phrase	Interface - EBSCOhost Research Databases<br>Search Screen - Advanced Search<br>Database - APA PsycInfo
S25	DE "Family Therapy"	Expanders - Apply equivalent subjects<br>Search modes - Boolean/Phrase	Interface - EBSCOhost Research Databases<br>Search Screen - Advanced Search<br>Database - APA PsycInfo
S26	DE "Support Groups"	Expanders - Apply equivalent subjects<br>Search modes - Boolean/Phrase	Interface - EBSCOhost Research Databases<br>Search Screen - Advanced Search<br>Database - APA PsycInfo
S27	DE "Social Support"	Expanders - Apply equivalent subjects<br>Search modes - Boolean/Phrase	Interface - EBSCOhost Research Databases<br>Search Screen - Advanced Search<br>Database - APA PsycInfo
S28	DE "Behavior Modification" OR DE "Behavior Contracting" OR DE "Behavior Therapy" OR DE "Biofeedback Training" OR DE "Contingency Management" OR DE "Fading (Conditioning)" OR DE "Omission Training" OR DE "Overcorrection" OR DE "Self-Management" OR DE "Self-Instructional Training" OR DE "Time Out"	Expanders - Apply equivalent subjects<br>Search modes - Boolean/Phrase	Interface - EBSCOhost Research Databases<br>Search Screen - Advanced Search<br>Database - APA PsycInfo
S29	DE "Cognitive Behavior Therapy"	Expanders - Apply equivalent subjects	Interface - EBSCOhost Research Databases<br>Search Screen - Advanced Search

		Search modes - Boolean/Phrase	Database - APA PsycInfo
S30	DE "Cognitive Therapy"	Expanders - Apply equivalent subjects<br>Search modes - Boolean/Phrase	Interface - EBSCOhost Research Databases<br>Search Screen - Advanced Search<br>Database - APA PsycInfo
S31	DE "Falls"	Expanders - Apply equivalent subjects<br>Search modes - Boolean/Phrase	Interface - EBSCOhost Research Databases<br>Search Screen - Advanced Search<br>Database - APA PsycInfo
S32	DE "Dietary Restraint"	Expanders - Apply equivalent subjects<br>Search modes - Boolean/Phrase	Interface - EBSCOhost Research Databases<br>Search Screen - Advanced Search<br>Database - APA PsycInfo
S33	DE "Client Education"	Expanders - Apply equivalent subjects<br>Search modes - Boolean/Phrase	Interface - EBSCOhost Research Databases<br>Search Screen - Advanced Search<br>Database - APA PsycInfo
S34	DE "Motor Processes" OR DE "Activity Level" OR DE "Animal Locomotion" OR DE "Gait" OR DE "Grasping" OR DE "Jumping" OR DE "Licking" OR DE "Locomotion" OR DE "Motor Control" OR DE "Motor Coordination" OR DE "Motor Skills" OR DE "Physical Activity" OR DE "Physical Agility" OR DE "Physical Mobility" OR DE "Posture" OR DE "Range of Motion" OR DE "Rotational Behavior" OR DE "Swimming" OR DE "Tonic Immobility" OR DE "Tool Use" OR DE "Wandering Behavior" OR DE "Motor Performance" OR DE "Finger Tapping" OR DE "Jumping" OR DE "Running" OR DE "Walking" OR DE "Physical Activity" OR DE "Actigraphy" OR DE "Exercise" OR DE "Physical Agility" OR DE "Physical Dexterity" OR DE "Posture" OR DE "Postural Sway"	Expanders - Apply equivalent subjects<br>Search modes - Boolean/Phrase	Interface - EBSCOhost Research Databases<br>Search Screen - Advanced Search<br>Database - APA PsycInfo

S35	TX ((obesity N3 intervention) OR program OR programme)	Expanders - Apply equivalent subjects<br>Search modes - Boolean/Phrase	Interface - EBSCOhost Research Databases<br>Search Screen - Advanced Search<br>Database - APA PsycInfo
S36	TX (lifestyle OR "life style")	Expanders - Apply equivalent subjects<br>Search modes - Boolean/Phrase	Interface - EBSCOhost Research Databases<br>Search Screen - Advanced Search<br>Database - APA PsycInfo
S37	TX (exercis*)	Expanders - Apply equivalent subjects<br>Search modes - Boolean/Phrase	Interface - EBSCOhost Research Databases<br>Search Screen - Advanced Search<br>Database - APA PsycInfo
S38	TX (physic* N1 (activ* OR fit*))	Expanders - Apply equivalent subjects<br>Search modes - Boolean/Phrase	Interface - EBSCOhost Research Databases<br>Search Screen - Advanced Search<br>Database - APA PsycInfo
S39	TX (walk* OR swim* OR aqua OR cycl* OR "weight lift*" OR danc* OR aerobics OR balance OR "electric* muscle stimulation" OR "neuromuscular electric* stimulation" OR electromyostimulation OR ems)	Expanders - Apply equivalent subjects<br>Search modes - Boolean/Phrase	Interface - EBSCOhost Research Databases<br>Search Screen - Advanced Search<br>Database - APA PsycInfo
S40	TX ((physic* OR strength* OR resist* OR circuit OR weight OR aerob* OR cross OR endurance OR struct* OR vibration*) N3 train*)	Expanders - Apply equivalent subjects<br>Search modes - Boolean/Phrase	Interface - EBSCOhost Research Databases<br>Search Screen - Advanced Search<br>Database - APA PsycInfo
S41	TX (behavioral OR behavioural OR "behavior modification *" OR "behavioural modification*" OR psychoth* OR psychosocial)	Expanders - Apply equivalent subjects<br>Search modes - Boolean/Phrase	Interface - EBSCOhost Research Databases<br>Search Screen - Advanced Search<br>Database - APA PsycInfo
S42	TX ((group OR cognit* OR behav*) N1 therap*)	Expanders - Apply equivalent subjects<br>Search modes - Boolean/Phrase	Interface - EBSCOhost Research Databases<br>Search Screen - Advanced Search<br>Database - APA PsycInfo
S43	TX (counseling OR counselling)	Expanders - Apply equivalent subjects<br>Search modes - Boolean/Phrase	Interface - EBSCOhost Research Databases<br>Search Screen - Advanced Search<br>Database - APA PsycInfo
S44	TX (educat*)	Expanders - Apply equivalent subjects	Interface - EBSCOhost Research Databases

		Search modes - Boolean/Phrase	Search Screen - Advanced Search Database - APA PsycInfo
S45	TX (diet* OR "healthy nutrition" OR (nutrition* N1 (knowledge OR educat* OR therap* OR program* OR intervention*)))	Expanders - Apply equivalent subjects Search modes - Boolean/Phrase	Interface - EBSCOhost Research Databases Search Screen - Advanced Search Database - APA PsycInfo
S46	TX (weight N1 (reduc* OR los* OR control* OR manag*))	Expanders - Apply equivalent subjects Search modes - Boolean/Phrase	Interface - EBSCOhost Research Databases Search Screen - Advanced Search Database - APA PsycInfo
S47	S5 OR S6 OR S7 OR S8 OR S9 OR S10 OR S11 OR S12 OR S13 OR S14 OR S15 OR S16 OR S17 OR S18 OR S19 OR S20 OR S21 OR S22 OR S23 OR S24 OR S25 OR S26 OR S27 OR S28 OR S29 OR S30 OR S31 OR S32 OR S33 OR S34 OR S35 OR S36 OR S37 OR S38 OR S39 OR S40 OR S41 OR S42 OR S43 OR S44 OR S45 OR S46	Expanders - Apply equivalent subjects Search modes - Boolean/Phrase	Interface - EBSCOhost Research Databases Search Screen - Advanced Search Database - APA PsycInfo
S48	DE "Aging" OR DE "Aging in Place" OR DE "Cognitive Aging" OR DE "Physiological Aging"	Expanders - Apply equivalent subjects Search modes - Boolean/Phrase	Interface - EBSCOhost Research Databases Search Screen - Advanced Search Database - APA PsycInfo
S49	DE "Geriatrics" OR DE "Geriatric Assessment" OR DE "Geriatric Psychiatry" OR DE "Geriatric Psychotherapy"	Expanders - Apply equivalent subjects Search modes - Boolean/Phrase	Interface - EBSCOhost Research Databases Search Screen - Advanced Search Database - APA PsycInfo
S50	DE "Gerontology"	Expanders - Apply equivalent subjects Search modes - Boolean/Phrase	Interface - EBSCOhost Research Databases Search Screen - Advanced Search Database - APA PsycInfo
S51	DE "Assisted Living"	Expanders - Apply equivalent subjects Search modes - Boolean/Phrase	Interface - EBSCOhost Research Databases Search Screen - Advanced Search Database - APA PsycInfo
S52	TX ((old* OR senior OR age*) N2 (adult* OR person* OR subject* OR individual* OR people OR group* OR man OR men OR woman OR women OR patient* OR client* OR outpatient* OR citizen* OR age* OR 60 OR 65)))	Expanders - Apply equivalent subjects Search modes - Boolean/Phrase	Interface - EBSCOhost Research Databases Search Screen - Advanced Search Database - APA PsycInfo

S53	TX (elder* OR eldes* OR geriat* OR geronto* OR frail* OR sarcopen* OR senior* OR agedly OR aging OR ageing)	Expanders - Apply equivalent subjects<br>Search modes - Boolean/Phrase	Interface - EBSCOhost Research Databases<br>Search Screen - Advanced Search<br>Database - APA PsycInfo
S54	TX (community-dwell*)	Expanders - Apply equivalent subjects<br>Search modes - Boolean/Phrase	Interface - EBSCOhost Research Databases<br>Search Screen - Advanced Search<br>Database - APA PsycInfo
S55	TX ((commun* OR indepen*) N1 (dwell* OR livi*)))	Expanders - Apply equivalent subjects<br>Search modes - Boolean/Phrase	Interface - EBSCOhost Research Databases<br>Search Screen - Advanced Search<br>Database - APA PsycInfo
S56	TX ((60 OR 65) N1 year*))	Expanders - Apply equivalent subjects<br>Search modes - Boolean/Phrase	Interface - EBSCOhost Research Databases<br>Search Screen - Advanced Search<br>Database - APA PsycInfo
S57	TX ((advanc* OR high*) N1 age*))	Expanders - Apply equivalent subjects<br>Search modes - Boolean/Phrase	Interface - EBSCOhost Research Databases<br>Search Screen - Advanced Search<br>Database - APA PsycInfo
S58	TX (super N1 age*)	Expanders - Apply equivalent subjects<br>Search modes - Boolean/Phrase	Interface - EBSCOhost Research Databases<br>Search Screen - Advanced Search<br>Database - APA PsycInfo
S59	TX (superage* OR super-age* OR centen* OR centa* OR nonagen* OR octagen* OR septuagen* OR septagen* OR supercenten* OR senium*)	Expanders - Apply equivalent subjects<br>Search modes - Boolean/Phrase	Interface - EBSCOhost Research Databases<br>Search Screen - Advanced Search<br>Database - APA PsycInfo
S60	S48 OR S49 OR S50 OR S51 OR S52 OR S53 OR S54 OR S55 OR S56 OR S57 OR S58 OR S59	Expanders - Apply equivalent subjects<br>Search modes - Boolean/Phrase	Interface - EBSCOhost Research Databases<br>Search Screen - Advanced Search<br>Database - APA PsycInfo
S61	S4 AND S47 AND S60	Expanders - Apply equivalent subjects<br>Search modes - Boolean/Phrase	Interface - EBSCOhost Research Databases<br>Search Screen - Advanced Search<br>Database - APA PsycInfo
S62	TX (control*) OR TX (random*) OR DE "Treatment" OR DE "Client Transfer" OR DE "Client Treatment Matching" OR DE "Habilitation" OR DE "Horticulture Therapy" OR DE "Hospice" OR DE "Human Potential Movement" OR DE	Expanders - Apply equivalent subjects<br>Search modes - Boolean/Phrase	Interface - EBSCOhost Research Databases<br>Search Screen - Advanced Search<br>Database - APA PsycInfo

	"Human Services" OR DE "Institutionalization" OR DE "Integrated Services" OR DE "Intervention" OR DE "Maintenance Therapy" OR DE "Mental Health Programs" OR DE "Private Practice" OR DE "Psychoeducation" OR DE "Respite Care" OR DE "Self-Help Techniques" OR DE "Treatment Outcomes" OR DE "Treatment Planning" OR DE "Addiction Treatment" OR DE "Adjunctive Treatment" OR DE "Adventure Therapy" OR DE "Aftercare" OR DE "Alternative Medicine" OR DE "Anxiety Management" OR DE "Behavior Modification" OR DE "Bibliotherapy" OR DE "Cognitive Behavior Therapy" OR DE "Cognitive Techniques" OR DE "Computer Assisted Therapy" OR DE "Counseling" OR DE "Creative Arts Therapy" OR DE "Cross Cultural Treatment" OR DE "Disease Management" OR DE "Health Care Services" OR DE "Hydrotherapy" OR DE "Interdisciplinary Treatment Approach" OR DE "Involuntary Treatment" OR DE "Language Therapy" OR DE "Life Sustaining Treatment" OR DE "Medical Treatment (General)" OR DE "Milieu Therapy" OR DE "Mind Body Therapy" OR DE "Mindfulness-Based Interventions" OR DE "Movement Therapy" OR DE "Multimodal Treatment Approach" OR DE "Multisystemic Therapy" OR DE		

	"Outpatient Treatment" OR DE "Pain Management" OR DE "Partial Hospitalization" OR DE "Personal Therapy" OR DE "Physical Treatment Methods" OR DE "Preventive Medicine" OR DE "Psychotherapy" OR DE "Rehabilitation" OR DE "Relaxation Therapy" OR DE "Sex Therapy" OR DE "Social Casework" OR DE "Sociotherapy" OR DE "Speech Therapy" OR DE "Spiritual Care" OR DE "Symptoms Based Treatment" OR DE "Therapeutic Processes" OR DE "Treatment Guidelines" OR DE "Video-Based Interventions" OR DE "Human Potential Movement" OR DE "Assertiveness Training" OR DE "Consciousness Raising Groups" OR DE "Encounter Group Therapy" OR DE "Gestalt Therapy" OR DE "Human Relations Training" OR DE "Sensitivity Training" OR DE "Transactional Analysis" OR DE "Institutionalization" OR DE "Hospitalization" OR DE "Incarceration" OR DE "Institution Visitation" OR DE "Institutional Release" OR DE "Intervention" OR DE "Crisis Intervention" OR DE "Early Intervention" OR DE "Family Intervention" OR DE "Group Intervention" OR DE "School Based Intervention" OR DE "Workplace Intervention" OR DE "Maintenance Therapy" OR DE "Methadone Maintenance" OR DE "Mental Health Programs" OR DE "Crisis Intervention Services" OR DE		

	"Deinstitutionalization" OR DE "Home Visiting Programs" OR DE "Hot Line Services" OR DE "Suicide Prevention Centers" OR DE "Self-Help Techniques" OR DE "Self-Management" OR DE "Treatment Outcomes" OR DE "Psychotherapeutic Outcomes" OR DE "Side Effects (Treatment)" OR DE "Treatment Compliance" OR DE "Treatment Duration" OR DE "Treatment Refusal" OR DE "Treatment Termination" OR DE "Treatment Withholding" OR DE "Treatment Planning" OR DE "Caring Behaviors" OR DE "Discharge Planning" OR DE "Posttreatment Followup" OR DE "Addiction Treatment" OR DE "Detoxification" OR DE "Medication-Assisted Treatment" OR DE "Substance Use Treatment" OR DE "Twelve Step Programs" OR DE "Adventure Therapy" OR DE "Wilderness Experience" OR DE "Alternative Medicine" OR DE "Acupuncture" OR DE "Aromatherapy" OR DE "Faith Healing" OR DE "Folk Medicine" OR DE "Shamanism" OR DE "Behavior Modification" OR DE "Behavior Contracting" OR DE "Behavior Therapy" OR DE "Biofeedback Training" OR DE "Classroom Behavior Modification" OR DE "Contingency Management" OR DE "Fading (Conditioning)" OR DE "Omission Training" OR DE "Overcorrection" OR DE "Self-Management" OR DE "Time Out" OR DE "Cognitive Behavior		

	Therapy" OR DE "Acceptance and Commitment Therapy" OR DE "Cognitive Processing Therapy" OR DE "Prolonged Exposure Therapy" OR DE "Cognitive Techniques" OR DE "Cognitive Restructuring" OR DE "Cognitive Therapy" OR DE "Self-Instructional Training" OR DE "Computer Assisted Therapy" OR DE "Telemedicine" OR DE "Counseling" OR DE "Community Counseling" OR DE "Cross Cultural Counseling" OR DE "Educational Counseling" OR DE "Genetic Counseling" OR DE "Gerontological Counseling" OR DE "Grief Counseling" OR DE "Group Counseling" OR DE "Marriage Counseling" OR DE "Microcounseling" OR DE "Multicultural Counseling" OR DE "Occupational Guidance" OR DE "Pastoral Counseling" OR DE "Peer Counseling" OR DE "Premarital Counseling" OR DE "Psychotherapeutic Counseling" OR DE "Rehabilitation Counseling" OR DE "School Counseling" OR DE "Creative Arts Therapy" OR DE "Art Therapy" OR DE "Dance Therapy" OR DE "Music Therapy" OR DE "Poetry Therapy" OR DE "Recreation Therapy" OR DE "Cross Cultural Treatment" OR DE "Cross Cultural Counseling" OR DE "Multicultural Counseling" OR DE "Health Care Services" OR DE "Continuum of Care" OR DE "Electronic Health Services"		

	OR DE "Health Care Delivery" OR DE "Hospital Programs" OR DE "Long Term Care" OR DE "Mental Health Services" OR DE "Palliative Care" OR DE "Prenatal Care" OR DE "Primary Health Care" OR DE "Life Sustaining Treatment" OR DE "CPR" OR DE "Medical Treatment (General)" OR DE "Gene Therapy" OR DE "Oncology" OR DE "Outpatient Treatment" OR DE "Outpatient Commitment" OR DE "Physical Treatment Methods" OR DE "Acupuncture" OR DE "Artificial Respiration" OR DE "Blood Transfusion" OR DE "Catheterization" OR DE "Cosmetic Techniques" OR DE "Deep Brain Stimulation" OR DE "Dental Treatment" OR DE "Dialysis" OR DE "Drug Therapy" OR DE "Electrosleep Treatment" OR DE "Gene Therapy" OR DE "Immunization" OR DE "Immunotherapy" OR DE "Narcoanalysis" OR DE "Phototherapy" OR DE "Physical Restraint" OR DE "Psychosurgery" OR DE "Radiation Therapy" OR DE "Shock Therapy" OR DE "Surgery" OR DE "Transcranial Magnetic Stimulation" OR DE "Psychotherapy" OR DE "Adlerian Psychotherapy" OR DE "Adolescent Psychotherapy" OR DE "Affirmative Therapy" OR DE "Analytical Psychotherapy" OR DE "Autogenic Training" OR DE "Brief Psychotherapy" OR DE		

	"Brief Relational Therapy" OR DE "Child Psychotherapy" OR DE "Client Centered Therapy" OR DE "Conversion Therapy" OR DE "Couples Therapy" OR DE "Eclectic Psychotherapy" OR DE "Emotion Focused Therapy" OR DE "Existential Therapy" OR DE "Experiential Psychotherapy" OR DE "Expressive Psychotherapy" OR DE "Eye Movement Desensitization Therapy" OR DE "Feminist Therapy" OR DE "Geriatric Psychotherapy" OR DE "Gestalt Therapy" OR DE "Group Psychotherapy" OR DE "Guided Imagery" OR DE "Humanistic Psychotherapy" OR DE "Hypnotherapy" OR DE "Individual Psychotherapy" OR DE "Insight Therapy" OR DE "Integrative Psychotherapy" OR DE "Interpersonal Psychotherapy" OR DE "Logotherapy" OR DE "Narrative Therapy" OR DE "Network Therapy" OR DE "Persuasion Therapy" OR DE "Primal Therapy" OR DE "Psychoanalysis" OR DE "Psychodrama" OR DE "Psychodynamic Psychotherapy" OR DE "Psychotherapeutic Counseling" OR DE "Psychotherapeutic Techniques" OR DE "Rational Emotive Behavior Therapy" OR DE "Reality Therapy" OR DE "Relationship Therapy" OR DE "Solution Focused Therapy" OR DE "Strategic Therapy" OR DE "Supportive Psychotherapy" OR		

	DE "Transactional Analysis" OR DE "Rehabilitation" OR DE "Cognitive Rehabilitation" OR DE "Criminal Rehabilitation" OR DE "Neuropsychological Rehabilitation" OR DE "Neurorehabilitation" OR DE "Occupational Therapy" OR DE "Physical Therapy" OR DE "Psychosocial Rehabilitation" OR DE "Rehabilitation Centers" OR DE "Telerehabilitation" OR DE "Relaxation Therapy" OR DE "Progressive Relaxation Therapy" OR DE "Social Casework" OR DE "Social Group Work" OR DE "Therapeutic Processes" OR DE "Dual Relationships" OR DE "Patient Selection" OR DE "Psychotherapeutic Processes" OR DE "Therapist Selection"		
S63	S61 AND S62	Expanders - Apply equivalent subjects<br>Search modes - Boolean/Phrase	Interface - EBSCOhost Research Databases<br>Search Screen - Advanced Search<br>Database - APA PsycInfo
S64	TX ((infant* OR child* OR adolescen* OR youth*) NOT ((old* OR senior OR age*) N2 (adult* OR person* OR subject* OR individual* OR people OR group* OR man OR men OR woman OR women OR patient* OR client* OR outpatient* OR citizen* OR age* OR 60 OR 65)))	Expanders - Apply equivalent subjects<br>Search modes - Boolean/Phrase	Interface - EBSCOhost Research Databases<br>Search Screen - Advanced Search<br>Database - APA PsycInfo
S65	S63 NOT S64	Expanders - Apply equivalent subjects<br>Search modes - Boolean/Phrase	Interface - EBSCOhost Research Databases<br>Search Screen - Advanced Search<br>Database - APA PsycInfo

E. Database(s): Cochrane Central Register of Controlled Trials (CENTRAL) via Cochrane Library (Wiley)
Search date: 2022-05-25

ID	Search
#1	MeSH descriptor: [Obesity] this term only
#2	MeSH descriptor: [Obesity, Morbid] this term only
#3	MeSH descriptor: [Obesity, Abdominal] this term only
#4	MeSH descriptor: [Obesity, Metabolically Benign] this term only
#5	MeSH descriptor: [Overweight] this term only
#6	(adipos* or obes* or overweight* or ("over" next weight*)):ti,ab,kw
#7	#1 or #2 or #3 or #4 or #5 or #6
#8	MeSH descriptor: [Behavior Therapy] explode all trees
#9	MeSH descriptor: [Counseling] explode all trees
#10	MeSH descriptor: [Social Support] this term only
#11	MeSH descriptor: [Program Evaluation] this term only
#12	MeSH descriptor: [Exercise] explode all trees
#13	MeSH descriptor: [Exercise Therapy] explode all trees
#14	MeSH descriptor: [Physical Education and Training] explode all trees
#15	MeSH descriptor: [Exercise Movement Techniques] explode all trees
#16	MeSH descriptor: [Physical Fitness] explode all trees
#17	MeSH descriptor: [Accidental Falls] this term only
#18	MeSH descriptor: [Postural Balance] this term only
#19	MeSH descriptor: [Motor Activity] this term only
#20	MeSH descriptor: [Gait] this term only
#21	MeSH descriptor: [Muscle Strength] explode all trees
#22	MeSH descriptor: [Diet] explode all trees
#23	MeSH descriptor: [Diet Therapy] explode all trees
#24	MeSH descriptor: [Fasting] this term only
#25	MeSH descriptor: [Food Preferences] this term only
#26	MeSH descriptor: [Patient Education as Topic] this term only
#27	MeSH descriptor: [Health Education] explode all trees
#28	MeSH descriptor: [Health Behavior] explode all trees
#29	MeSH descriptor: [Health Promotion] explode all trees
#30	MeSH descriptor: [Life Style] this term only
#31	((obesity near/3 intervention) or program or programme):ti,ab,kw
#32	(lifestyle or ("life" next "style")):ti,ab,kw
#33	(exercis*):ti,ab,kw
#34	(physic* next (activ* or fit*)):ti,ab,kw
#35	(walk* or swim* or aqua or cycl* or ("weight" next lift*) or danc* or aerobics or balance or (electric* next "muscle stimulation") or ("neuromuscular" next (electric* next "stimulation")) or electromyostimulation or ems):ti,ab,kw
#36	((physic* or strength* or resist* or circuit or weight or aerob* or cross or endurance or vibration*) near/3 train*):ti,ab,kw

#37	(behavioral or behavioural or behavior or behaviour or modification* or psychoth* or psychosocial):ti,ab,kw
#38	((group or cognit* or behav*) next therap*):ti,ab,kw
#39	(counseling or counselling):ti,ab,kw
#40	(educat*):ti,ab,kw
#41	(diet* or ("healthy" next "nutrition") or (nutrition* next (knowledge or educat* or therap* or program* or intervention*))):ti,ab,kw
#42	(low-calori* or (calori* next restrict*) or low-energ* or (energ* next restrict*) or low-carbohydrate or high-carbohydrate or low-fat or high-fat or low-protein or high-protein or vegetarian or vegan or mediterranean or dash):ti,ab,kw
#43	MeSH descriptor: [Weight Loss] this term only
#44	(weight near/1 (reduc* or los* or control* or manag*)):ti,ab,kw
#45	#8 or #9 or #10 or #11 or #12 or #13 or #14 or #15 or #16 or #17 or #18 or #19 or #20 or #21 or #22 or #23 or #24 or #25 or #26 or #27 or #28 or #29 or #30 or #31 or #32 or #33 or #34 or #35 or #36 or #37 or #38 or #39 or #40 or #41 or #42 or #43 or #44
#46	#1 or #2 or #3 or #4 or #5
#47	MeSH descriptor: [] explode all trees and with qualifier(s): [diet therapy - DH, prevention & control - PC, rehabilitation - RH, therapy - TH, psychology - PX]
#48	#46 and #47
#49	MeSH descriptor: [Weight Reduction Programs] explode all trees
#50	#48 or #49
#51	#7 and #45
#52	#50 or #51
#53	MeSH descriptor: [Aged] explode all trees
#54	MeSH descriptor: [Geriatrics] this term only
#55	MeSH descriptor: [Frailty] this term only
#56	MeSH descriptor: [Sarcopenia] this term only
#57	MeSH descriptor: [Independent Living] this term only
#58	((old* or senior or age*) near/2 (adult* or person* or subject* or individual* or people or group* or man or men or woman or women or patient* or client* or outpatient* or citizen* or 60 or 65 or age*)):ti,ab,kw
#59	(elder* or eldes* or geriat* or geronto* or frail* or sarcopen* or senior* or agedly or ageing or aging):ti,ab,kw
#60	(community-dwell*):ti,ab,kw
#61	((commun* or indepen*) next (dwell* or livi*)):ti,ab,kw
#62	((60 or 65) next year*):ti,ab,kw
#63	((advanc* or high*) next age*):ti,ab,kw
#64	(super next age*):ti,ab,kw
#65	(superage* or super-age* or centen* or centa* or nonagen* or octagen* or septuagen* or septagen* or supercenten* or senium*):ti,ab,kw
#66	#53 or #54 or #55 or #56 or #57 or #58 or #59 or #60 or #61 or #62 or #63 or #64 or #65
#67	#52 and #66
#68	MeSH descriptor: [Infant] explode all trees
#69	MeSH descriptor: [Child] explode all trees
#70	MeSH descriptor: [Adolescent] explode all trees
#71	#68 or #69 or #70

#72	#71 not #53
#73	#67 not #72

F. Database(s): Science Citation Index Expanded (SCI-EXPANDED) via Web of Science Core Collection/Clarivate (1970 - current)
Search date: 2022-05-24

# 27	#25 NOT #26<br>*Indexes=SCI-EXPANDED Timespan=All years*
# 26	TS=((infant* OR child* OR adolescen* OR youth*) NOT ((old* OR "senior" OR age*) NEAR/0 (adult* OR person* OR subject* OR individual* OR "people" OR group* OR m?n OR wom?n OR patient* OR client* OR outpatient* OR citizen* OR 60 OR 65 OR age*)))<br>*Indexes=SCI-EXPANDED Timespan=All years*
# 25	#1 AND #14 AND #23 AND #24<br>*Indexes=SCI-EXPANDED Timespan=All years*
# 24	TS=("clinical trial*" OR "research design" OR "comparative stud*" OR "evaluation stud*" OR "controlled trial*" OR "follow-up stud*" OR "prospective stud*" OR random* OR placebo* OR "single blind*" OR "double blind*")<br>*Indexes=SCI-EXPANDED Timespan=All years*
# 23	#15 OR #16 OR #17 OR #18 OR #19 OR #20 OR #21 OR #22<br>*Indexes=SCI-EXPANDED Timespan=All years*
# 22	TS=(superage* OR "super-age*" OR centen* OR centa* OR nonagen* OR octagen* OR sept?agen* OR supercenten* OR senium*)<br>*Indexes=SCI-EXPANDED Timespan=All years*
# 21	TS=("super" NEAR/0 age*)<br>*Indexes=SCI-EXPANDED Timespan=All years*
# 20	TS=((advanc* OR high*) NEAR/0 age*)<br>*Indexes=SCI-EXPANDED Timespan=All years*
# 19	TS=((60 OR 65) NEAR/0 year*)<br>*Indexes=SCI-EXPANDED Timespan=All years*
# 18	TS=((commun* OR indepen*) NEAR/0 (dwell* OR livi*))<br>*Indexes=SCI-EXPANDED Timespan=All years*
# 17	TS=("community-dwell*")<br>*Indexes=SCI-EXPANDED Timespan=All years*
# 16	TS=(elder* OR eldes* OR geriat* OR geronto* OR frail* OR sarcopen* OR senior* OR "agedly" OR ag?ing)<br>*Indexes=SCI-EXPANDED Timespan=All years*
# 15	TS=((old* OR "senior" OR age*) NEAR/0 (adult* OR person* OR subject* OR individual* OR "people" OR group* OR m?n OR wom?n OR patient* OR client* OR outpatient* OR citizen* OR 60 OR 65 OR age*))<br>*Indexes=SCI-EXPANDED Timespan=All years*
# 14	#2 OR #3 OR #4 OR #5 OR #6 OR #7 OR #8 OR #9 OR #10 OR #11 OR #12 OR #13<br>*Indexes=SCI-EXPANDED Timespan=All years*
# 13	TS=("weight" NEAR/0 (reduc* OR los* OR control* OR manag*))<br>*Indexes=SCI-EXPANDED Timespan=All years*

# 12	TS=("low-calori*" OR "calori* restrict*" OR "low-energ*" OR "energ* restrict*" OR "low-carbohydrate" OR "high-carbohydrate" OR "low-fat" OR "high-fat" OR "low-protein" OR "high-protein" OR "vegetarian" OR "vegan" OR "mediterranean" OR "dash")<br>*Indexes=SCI-EXPANDED Timespan=All years*
# 11	TS=(diet* OR "healthy nutrition" OR (nutrition* NEAR/0 ("knowledge" OR educat* OR therap* OR program* OR intervention*)))<br>*Indexes=SCI-EXPANDED Timespan=All years*
# 10	TS=(educat*)<br>*Indexes=SCI-EXPANDED Timespan=All years*
# 9	TS=(counsel?ing)<br>*Indexes=SCI-EXPANDED Timespan=All years*
# 8	TS=(("group" OR cognit* OR behav*) NEAR/0 therap*)<br>*Indexes=SCI-EXPANDED Timespan=All years*
# 7	TS=(behavio?ral OR "behavio?r modification*" OR psychoth* OR "psychosocial")<br>*Indexes=SCI-EXPANDED Timespan=All years*
# 6	TS=((physic* OR strength* OR resist* OR "circuit" OR "weight" OR aerob* OR "cross" OR "endurance" OR vibration*) NEAR/2 train*)<br>*Indexes=SCI-EXPANDED Timespan=All years*
# 5	TS=(walk* OR swim* OR "aqua" OR cycl* OR "weight lift*" OR danc* OR "aerobics" OR "balance" OR "electric* muscle stimulation" OR "neuromuscular electric* stimulation" OR "electromyostimulation" OR "ems")<br>*Indexes=SCI-EXPANDED Timespan=All years*
# 4	TS=(physic* NEAR/0 (activ* OR fit*))<br>*Indexes=SCI-EXPANDED Timespan=All years*
# 3	TS=("lifestyle" OR "life style")<br>*Indexes=SCI-EXPANDED Timespan=All years*
# 2	TS=(("obesity" NEAR/2 "intervention") OR "program" OR "programme")<br>*Indexes=SCI-EXPANDED Timespan=All years*
# 1	TS=(adipos* OR obes* OR overweight* OR "over weight*")<br>*Indexes=SCI-EXPANDED Timespan=All years*

Table S2: Special rules for the assessment of risk of bias

Domain	Signaling question	Special rule(s)
Domain 1: Risk of bias arising from the randomization process	1.1 Was the allocation sequence random?	Vote yes: if authors state that allocation was randomized
	1.2 Was the allocation sequence concealed until participants were enrolled and assigned to interventions?	Vote probably yes: If done at other center or by person not otherwise involved with study<br>Vote no information: if stated information for concealed allocation sequence is insufficient
	1.3 Did baseline differences between intervention groups suggest a problem with the randomization process?	Prognostic factors for consideration: sex, age, body composition/BMI/weight, physical function, comorbidities (e.g., metabolic syndrome, type 2 diabetes mellitus)
Domain 2: Risk of bias due to deviations from the intended interventions (effect of assignment to intervention)	Additional item for adherence: Was there non-adherence to the assigned intervention regimen that could have affected participants' outcomes?	If adherence:<br>- ≥85%: answer 'probably no'<br>- <85%: answer 'probably yes'<br>- If not reported: answer 'no information'
Domain 3: Bias due to missing outcome data	3.1 Were data for this outcome available for all, or nearly all, participants randomized?	Cut-off from dropouts: 20%
	3.2 Is there evidence that the result was not biased by missing outcome data?	If methods for statistical imputation methods were not described: answer 'probably no'
Domain 5: Bias in selection of the reported result	5.1 Were the data that produced this result analysed in accordance with a pre-specified analysis plan that was finalized before unblinded outcome data were available for analysis?	- if no protocol was available: answer 'no information'<br>- if protocol was available but information was not sufficiently available for evaluation of potential deviations: answer 'no information'<br>- if protocol was available and sufficient information was available for evaluation of potential deviations: answer 'probably no' or 'no'<br>- if registry entry was available and no deviations were present: answer 'probably yes'

If a domain, signaling question or answer option is not covered in the table, the rules of the full guidance document were followed:
https://drive.google.com/file/d/19R9savfPdCHC8XLz2iiMvL_71lPJERWK/view

Table S3: GRADE used thresholds for small, medium and large effects

Outcome	Small	Medium	Large
Performance batteries	± ≥0.2	± ≥0.5	± ≥0.8
VO_2max*	± ≥1.646 ml/min/kg		
Lean Tissue/Fat Mass	± ≥0.5 kg	± ≥3.5 kg	± ≥7 kg
Body Weight	± ≥1 kg	± ≥5 kg	± ≥10 kg
Bone Mineral Density Total Hip	± ≥0.01 g/m^2	± ≥0.03 g/m^2	

*The weighted mean of baseline values across studies was calculated and a minimal important difference of 10% was applied (Asikainen TM et al. Randomised, controlled walking trials in postmenopausal women: the minimum dose to improve aerobic fitness?. Br J Sports Med. 2002;36(3):189-94.)

Table S4: Excluded references from full-text screening

**References** (see page 122-226)	**Exclusion reason**
1-82; 1446-1451	Wrong publication type
83-1421; 1452-1500	Wrong patient population
1422-1429; 1501-1505	Wrong intervention
1430-1433	Wrong outcome
1434-1439; 1506	Wrong study type
1440-1445	Ongoing studies

Table S5: Included studies and reports

Study ID	Included References
Abdelbasset 2020	Abdelbasset WK, Alrawaili SM, Moawd SA, Elsayed SH. Effect of 12-week endurance exercise on obese elderly patients with COPD: a randomized trial. Journal of Advanced Pharmacy Education & Research. 2020;10(1):101-6.
Amamou 2017	Amamou T, Normandin E, Pouliot J, Dionne IJ, Brochu M, Riesco E. Effect of a High-Protein Energy-Restricted Diet Combined with Resistance Training on Metabolic Profile in Older Individuals with Metabolic Impairments. J Nutr Health Aging. 2017;21(1):67-74. doi: 10.1007/s12603-016-0760-8. PMID: 27999852.
Andonian 2024	Andonian BJ, Ross LM, Sudnick AM, Johnson JL, Pieper CF, Belski KB, Counts JD, King AP, Wallis JT, Bennett WC, Gillespie JC, Moertl KM, Richard D, Huebner JL, Connelly MA, Siegler IC, Kraus WE, Bales CW, Porter Starr KN, Huffman KM. Effect of Remotely Supervised Weight Loss and Exercise Training Versus Lifestyle Counseling on Cardiovascular Risk and Clinical Outcomes in Older Adults With Rheumatoid Arthritis: A Randomized Controlled Trial. ACR Open Rheumatol. 2024 Mar;6(3):124-136. doi: 10.1002/acr2.11639. Epub 2023 Dec 21. PMID: 38126260.
	Kim G, Ross LM, Sudnick AM, Johnson JL, Pieper CF, Connelly MA, Ilkayeva O, Muehlbauer MJ, Bales CW, Porter Starr KN, Kraus WE, Andonian BJ, Huffman KM. The impact of lifestyle modification on metabolic pathways in older adults with overweight/obesity and rheumatoid arthritis: a secondary exploratory analysis of the SWET-RA study. BMC Rheumatol. 2025 Jun 9;9(1):68. doi: 10.1186/s41927-025-00525-8.
Ard 2017	Ard JD, Gower B, Hunter G, Ritchie CS, Roth DL, Goss A, Wingo BC, Bodner EV, Brown CJ, Bryan D, Buys DR, Haas MC, Keita AD, Flagg LA, Williams CP, Locher JL. Effects of Calorie Restriction in Obese Older Adults: The CROSSROADS Randomized Controlled Trial. J Gerontol A Biol Sci Med Sci. 2017 Dec 12;73(1):73-80. doi: 10.1093/gerona/glw237. PMID: 28003374.
	Bragg AE, Crowe-White KM, Ellis AC, Studer M, Phillips F, Samsel S, Parton J, Locher JL, Ard JD. Changes in Cardiometabolic Risk Among Older Adults with Obesity: An Ancillary Analysis of a Randomized Controlled Trial Investigating Exercise Plus Weight Maintenance and Exercise Plus Intentional Weight Loss by Caloric Restriction. J Acad Nutr Diet. 2022 Feb;122(2):354-362. doi: 10.1016/j.jand.2021.07.009. Epub 2021 Sep 1. PMID: 34486528.
	Senkus KE, Crowe-White KM, Locher JL, Ard JD. Relative fat mass assessment estimates changes in adiposity among female older adults with obesity after a 12-month exercise and diet intervention. Ann Med. 2022 Dec;54(1):1160-1166. doi: 10.1080/07853890.2022.2067352. PMID: 35471192.
	Senkus KE, Crowe-White KM, Bolland AC, Locher JL, Ard JD. Changes in adiponectin:leptin ratio among older adults with obesity following a 12-month exercise and diet intervention. Nutr Diabetes. 2022 Jun 2;12(1):30. doi: 10.1038/s41387-022-00207-1. PMID: 35654771
Balachandran 2014	Balachandran A, Krawczyk SN, Potiaumpai M, Signorile JF. High-speed circuit training vs hypertrophy training to improve physical function in sarcopenic obese adults: a randomized controlled trial. Exp Gerontol. 2014 Dec;60:64-71. doi: 10.1016/j.exger.2014.09.016. Epub 2014 Oct 1. PMID: 25281504.

Beavers 2015	Beavers KM, Gordon MM, Easter L, Beavers DP, Hairston KG, Nicklas BJ, Vitolins MZ. Effect of protein source during weight loss on body composition, cardiometabolic risk and physical performance in abdominally obese, older adults: a pilot feeding study. J Nutr Health Aging. 2015 Jan;19(1):87-95. doi: 10.1007/s12603-015-0438-7. PMID: 25560821.
Beavers 2019	Beavers KM, Nesbit BA, Kiel JR, Sheedy JL, Arterburn LM, Collins AE, Ford SA, Henderson RM, Coleman CD, Beavers DP. Effect of an Energy-Restricted, Nutritionally Complete, Higher Protein Meal Plan on Body Composition and Mobility in Older Adults With Obesity: A Randomized Controlled Trial. J Gerontol A Biol Sci Med Sci. 2019 May 16;74(6):929-935. doi: 10.1093/gerona/gly146. PMID: 30629126.
	Serra MC, Beavers DP, Henderson RM, Kelleher JL, Kiel JR, Beavers KM. Effects of a Hypocaloric, Nutritionally Complete, Higher Protein Meal Plan on Regional Body Fat and Cardiometabolic Biomarkers in Older Adults with Obesity. Ann Nutr Metab. 2019;74(2):149-155. doi: 10.1159/000497066. Epub 2019 Feb 12. PMID: 30754039.
	Weaver AA, Houston DK, Shapses SA, Lyles MF, Henderson RM, Beavers DP, Baker AC, Beavers KM. Effect of a hypocaloric, nutritionally complete, higher-protein meal plan on bone density and quality in older adults with obesity: a randomized trial. Am J Clin Nutr. 2019 Feb 1;109(2):478-486. doi: 10.1093/ajcn/nqy237. PMID: 30624598.
	Quillen EE, Beavers DP, O'Brien Cox A, Furdui CM, Lee J, Miller RM, Wu H, Beavers KM. Use of Metabolomic Profiling to Understand Variability in Adiposity Changes Following an Intentional Weight Loss Intervention in Older Adults. Nutrients. 2020 Oct 19;12(10):3188. doi: 10.3390/nu12103188. PMID: 33086512.
	Shaver LN, Beavers DP, Kiel J, Kritchevsky SB, Beavers KM. Effect of Intentional Weight Loss on Mortality Biomarkers in Older Adults With Obesity. J Gerontol A Biol Sci Med Sci. 2019 Jul 12;74(8):1303-1309. doi: 10.1093/gerona/gly192. PMID: 30137218.
Bowen 2022	Bowen PG, Affuso O, Opoku-Agyeman W, Mixon VR, Clay OJ. Texting Older Sisters to Step to Manage Obesity in Older Black Women: A Feasibility Study. Am J Prev Med. 2022 Jul;63(1 Suppl 1):S56-S66. doi: 10.1016/j.amepre.2022.03.014. PMID: 35725141.
Brennan 2020	Brennan AM, Standley RA, Yi F, Carnero EA, Sparks LM, Goodpaster BH. Individual Response Variation in the Effects of Weight Loss and Exercise on Insulin Sensitivity and Cardiometabolic Risk in Older Adults. Front Endocrinol (Lausanne). 2020 Sep 10;11:632. doi: 10.3389/fendo.2020.00632. PMID: 33013705.
	Brennan AM, Standley RA, Anthony SJ, Grench KE, Helbling NL, DeLany JP, Cornnell HH, Yi F, Stefanovic-Racic M, Toledo FGS, Coen PM, Carnero EA, Goodpaster BH. Weight loss and exercise differentially affect insulin sensitivity, body composition, cardiorespiratory fitness and muscle strength in older adults with obesity; a randomized controlled trial. J Gerontol A Biol Sci Med Sci. 2021 Aug 18:glab240. doi: 10.1093/gerona/glab240. Epub ahead of print. PMID: 34406407.
Brubaker 2023	Brubaker PH, Nicklas BJ, Houston DK, Hundley WG, Chen H, Molina AJA, Lyles WM, Nelson B, Upadhya B, Newland R, Kitzman DW. A Randomized, Controlled Trial of Resistance Training Added to Caloric Restriction Plus Aerobic Exercise Training in Obese Heart Failure With Preserved Ejection Fraction. Circ Heart Fail. 2023 Feb;16(2):e010161. doi:

	10.1161/CIRCHEARTFAILURE.122.010161. Epub 2022 Oct 31. PMID: 36314122.
Cai 2019	Cai R, Chao J, Li D, Zhang M, Kong L, Wang Y. Effect of community-based lifestyle interventions on weight loss and cardiometabolic risk factors in obese elderly in China: A randomized controlled trial. Exp Gerontol. 2019 Dec;128:110749. doi: 10.1016/j.exger.2019.110749. Epub 2019 Oct 20. PMID: 31644921.
Coppock 2023	Coppock JA, McNulty AL, Porter Starr KN, Holt AG, Borack MS, Kosinski AS, Collins AT, Bales CW, DeFrate LE. The effects of a 6-month weight loss intervention on physical function and serum biomarkers in older adults with and without osteoarthritis. Osteoarthr Cartil Open. 2023 May 26;5(3):100376. doi: 10.1016/j.ocarto.2023.100376. PMID: 37719442.
Davidson 2009	Davidson LE, Hudson R, Kilpatrick K, Kuk JL, McMillan K, Janiszewski PM, Lee S, Lam M, Ross R. Effects of exercise modality on insulin resistance and functional limitation in older adults: a randomized controlled trial. Arch Intern Med. 2009 Jan 26;169(2):122-31. doi: 10.1001/archinternmed.2008.558. PMID: 19171808.
	Ko G, Davidson LE, Brennan AM, Lam M, Ross R. Abdominal Adiposity, Not Cardiorespiratory Fitness, Mediates the Exercise-Induced Change in Insulin Sensitivity in Older Adults. PLoS One. 2016 Dec 9;11(12):e0167734. doi: 10.1371/journal.pone.0167734. PMID: 27936206.
Elsayed 2022	Elsayed MM, Rabiee A, El Refaye GE, Elsisi HF. Aerobic Exercise with Mediterranean-DASH Intervention for Neurodegenerative Delay Diet Promotes Brain Cells' Longevity despite Sex Hormone Deficiency in Postmenopausal Women: A Randomized Controlled Trial. Oxid Med Cell Longev. 2022 Apr 4;2022:4146742. doi: 10.1155/2022/4146742. PMID: 35419166.
Fanning 2020	Fanning J, Brooks AK, Ip E, Nicklas BJ, Rejeski WJ, Nesbit B, Ford S. A Mobile Health Behavior Intervention to Reduce Pain and Improve Health in Older Adults With Obesity and Chronic Pain: The MORPH Pilot Trial. Front Digit Health. 2020 Dec;2:598456. doi: 10.3389/fdgth.2020.598456. Epub 2020 Dec 18. PMID: 33817686.
	Fanning J, Brooks AK, Hsieh KL, Kershner K, Furlipa J, Nicklas BJ, Rejeski WJ. The Effects of a Pain Management-Focused Mobile Health Behavior Intervention on Older Adults' Self-efficacy, Satisfaction with Functioning, and Quality of Life: a Randomized Pilot Trial. Int J Behav Med. 2022 Apr;29(2):240-246. doi: 10.1007/s12529-021-10003-3. Epub 2021 May 20. PMID: 34018138.
Fanning 2021	Fanning J, Rejeski WJ, Leng I, Barnett C, Lovato JF, Lyles MF, Nicklas BJ. Intervening on exercise and daylong movement for weight loss maintenance in older adults: A randomized, clinical trial. Obesity (Silver Spring). 2022 Jan;30(1):85-95. doi: 10.1002/oby.23318. PMID: 34932885; PMCID: PMC8711609.
	Fanning J, Nicklas B, Furlipa J, Rejeski WJ. The impact of Dietary Weight loss, Aerobic Exercise, and Daylong Movement on Social Cognitive Mediators of Long-term Weight loss. J Behav Med. 2023 Jun;46(3):499-508. doi: 10.1007/s10865-022-00359-6. Epub 2022 Oct 10. PMID: 36215000.
	Bahrami M, Burdette JH, Laurienti PJ, Nicklas BJ, Rejeski WJ, Fanning J. Neural Determinants of Sedentary Lifestyle in Older Adults: A Brain Network Analysis. Brain Behav. 2025 Jan;15(1):e70085. doi: 10.1002/brb3.70085.

Ferhi 2023	Ferhi H, Gaied Chortane S, Durand S, Beaune B, Boyas S, Maktouf W. Effects of Physical Activity Program on Body Composition, Physical Performance, and Neuromuscular Strategies during Walking in Older Adults with Sarcopenic Obesity: Randomized Controlled Trial. Healthcare (Basel). 2023 Aug 14;11(16):2294. doi: 10.3390/healthcare11162294. PMID: 37628492.
	Magtouf E, Chortane SG, Chortane OG, Boyas S, Beaune B, Durand S, Maktouf W. Influence of Concurrent Exercise Training on Ankle Muscle Activation during Static and Proactive Postural Control on Older Adults with Sarcopenic Obesity: A Multicenter, Randomized, and Controlled Trial. Eur J Investig Health Psychol Educ. 2023 Nov 27;13(12):2779-2794. doi: 10.3390/ejihpe13120192. PMID: 38131891.
Furlano 2023	Furlano JA, Horst BR, Petrella RJ, Shoemaker JK, Nagamatsu LS. Changes in Cognition and Brain Function After 26 Weeks of Progressive Resistance Training in Older Adults at Risk for Diabetes: A Pilot Randomized Controlled Trial. Can J Diabetes. 2023 Apr;47(3):250-256. doi: 10.1016/j.jcjd.2023.01.004. Epub 2023 Jan 13. PMID: 36858923.
Gregori 2025	Gregori G, Mediwala S, Liebschner M, Kim D, Bryant MS, Klonis N, Armamento-Villareal R, Qualls C, Villareal DT. Bone quality response to lifestyle intervention in older adults with obesity (LIMB-Q trial): a randomised controlled trial. Lancet Healthy Longev. 2025 Sep;6(9):100761. doi: 10.1016/j.lanhl.2025.100761. Epub 2025 Sep 18.
Haywood 2017	Haywood CJ, Prendergast LA, Purcell K, Le Fevre L, Lim WK, Galea M, Proietto J. Very Low Calorie Diets for Weight Loss in Obese Older Adults-A Randomized Trial. J Gerontol A Biol Sci Med Sci. 2017 Dec 12;73(1):59-65. doi: 10.1093/gerona/glx012. PMID: 28329121.
	Haywood CJ, Prendergast LA, Lim R, Lappas M, Lim WK, Proietto J. Obesity in older adults: Effect of degree of weight loss on cardiovascular markers and medications. Clin Obes. 2019 Aug;9(4):e12316. doi: 10.1111/cob.12316. Epub 2019 Jun 17. PMID: 31207126.
Horie 2016	Horie NC, Serrao VT, Simon SS, Gascon MR, Dos Santos AX, Zambone MA, Del Bigio de Freitas MM, Cunha-Neto E, Marques EL, Halpern A, de Melo ME, Mancini MC, Cercato C. Cognitive Effects of Intentional Weight Loss in Elderly Obese Individuals With Mild Cognitive Impairment. J Clin Endocrinol Metab. 2016 Mar;101(3):1104-12. doi: 10.1210/jc.2015-2315. Epub 2015 Dec 29. PMID: 26713821.
Ismail 2023	Ismail AMA, El-Azeim ASA, Saif HFAEA. Effect of aerobic exercise alone or combined with Mediterranean diet on dry eye in obese hypertensive elderly. Ir J Med Sci. 2023 Dec;192(6):3151-3161. doi: 10.1007/s11845-023-03387-6. Epub 2023 May 9. PMID: 37160570.
Kallings 2009	Kallings LV, Sierra Johnson J, Fisher RM, Faire Ud, Ståhle A, Hemmingsson E, Hellénius ML. Beneficial effects of individualized physical activity on prescription on body composition and cardiometabolic risk factors: results from a randomized controlled trial. Eur J Cardiovasc Prev Rehabil. 2009 Feb;16(1):80-4. doi: 10.1097/HJR.0b013e32831e953a. PMID: 19237997.
	Sjögren P, Sierra-Johnson J, Kallings LV, Cederholm T, Kolak M, Halldin M, Brismar K, de Faire U, Hellénius ML, Fisher RM. Functional changes in adipose tissue in a randomised controlled trial of physical activity. Lipids Health Dis. 2012 Jun 21;11:80. doi: 10.1186/1476-511X-11-80. PMID: 22721353; PMCID: PMC3475078.
	Olsson SJ, Börjesson M, Ekblom-Bak E, Hemmingsson E, Hellénius ML, Kallings LV. Effects of the Swedish physical activity on prescription model

	on health-related quality of life in overweight older adults: a randomised controlled trial. BMC Public Health. 2015 Jul 21;15:687. doi: 10.1186/s12889-015-2036-3. PMID: 26193882; PMCID: PMC4509721.
Kemmler 2016	Kemmler W, Teschler M, Weissenfels A, Bebenek M, von Stengel S, Kohl M, Freiberger E, Goisser S, Jakob F, Sieber C, Engelke K. Whole-body electromyostimulation to fight sarcopenic obesity in community-dwelling older women at risk. Resultsof the randomized controlled FORMOsA-sarcopenic obesity study. Osteoporos Int. 2016 Nov;27(11):3261-3270. doi: 10.1007/s00198-016-3662-z. Epub 2016 Jun 12. PMID: 27289534.
	Kemmler, W.; Von Stengel, S.; Teschler, M.; Weissenfels, A.; Bebenek, M.; Kohl, M.; Freiberger, E.; Bollheimer, C.; Goisser, S.; Sieber, C.; et al. Whole-body Electromyostimulation and Sarcopenic Obesity Results of the randomized controlled FORMOsA-Sarcopenic Obesity Study. Osteologie 2016; 25
	Wittmann K, Sieber C, von Stengel S, Kohl M, Freiberger E, Jakob F, Lell M, Engelke K, Kemmler W. Impact of whole body electromyostimulation on cardiometabolic risk factors in older women with sarcopenic obesity: the randomized controlled FORMOsA-sarcopenic obesity study. Clin Interv Aging. 2016 Nov 18;11:1697-1706. doi: 10.2147/CIA.S116430. PMID: 27920508; PMCID: PMC5123721.
Kemmler 2017	Kemmler W, Weissenfels A, Teschler M, Willert S, Bebenek M, Shojaa M, Kohl M, Freiberger E, Sieber C, von Stengel S. Whole-body electromyostimulation and protein supplementation favorably affect sarcopenic obesity in community-dwelling older men at risk: the randomized controlled FranSO study. Clin Interv Aging. 2017 Sep 21;12:1503-1513. doi: 10.2147/CIA.S137987. PMID: 28989278; PMCID: PMC5624743.
	Kemmler W, Kohl M, Freiberger E, Sieber C, von Stengel S. Effect of whole-body electromyostimulation and / or protein supplementation on obesity and cardiometabolic risk in older men with sarcopenic obesity: the randomized controlled FranSO trial. BMC Geriatr. 2018 Mar 9;18(1):70. doi: 10.1186/s12877-018-0759-6. PMID: 29523089; PMCID: PMC5845205.
	Kemmler W, Grimm A, Bebenek M, Kohl M, von Stengel S. Effects of Combined Whole-Body Electromyostimulation and Protein Supplementation on Local and Overall Muscle/Fat Distribution in Older Men with Sarcopenic Obesity: The Randomized Controlled Franconia Sarcopenic Obesity (FranSO) Study. Calcif Tissue Int. 2018 Sep;103(3):266-277. doi: 10.1007/s00223-018-0424-2. Epub 2018 Apr 19. PMID: 29675640.
	Kemmler W, von Stengel S, Kohl M, Rohleder N, Bertsch T, Sieber CC, Freiberger E, Kob R. Safety of a Combined WB-EMS and High-Protein Diet Intervention in Sarcopenic Obese Elderly Men. Clin Interv Aging. 2020 Jun 24;15:953-967. doi: 10.2147/CIA.S248868. PMID: 32612355; PMCID: PMC7322975.
Kim 2018	Kim J, Park HY, Lim K. Effects of 12 Weeks of Combined Exercise on Heart Rate Variability and Dynamic Pulmonary Function in Obese and Elderly Korean Women. Iran J Public Health. 2018 Jul;47(Suppl 1):74-81. PMID: 30186815; PMCID: PMC6124150.
Kim 2019	Kim SW, Jung WS, Park W, Park HY. Twelve Weeks of Combined Resistance and Aerobic Exercise Improves Cardiometabolic Biomarkers and Enhances Red Blood Cell Hemorheological Function in Obese Older Men: A Randomized Controlled Trial. Int J Environ Res Public Health. 2019 Dec

	10;16(24):5020. doi: 10.3390/ijerph16245020. PMID: 31835508; PMCID: PMC6950327.
Kim 2020	Kim JS, Kim CJ. [Effect of a Physical Activity Promoting Program Based on the IMB Model on Obese-Metabolic Health Outcomes among Obese Older Adults with Knee Osteoarthritis]. J Korean Acad Nurs. 2020 Apr;50(2):271-285. Korean. doi: 10.4040/jkan.2020.50.2.271. PMID: 32376814.
Kitzman 2016	Kitzman DW, Brubaker P, Morgan T, Haykowsky M, Hundley G, Kraus WE, Eggebeen J, Nicklas BJ. Effect of Caloric Restriction or Aerobic Exercise Training on Peak Oxygen Consumption and Quality of Life in Obese Older Patients With Heart Failure With Preserved Ejection Fraction: A Randomized Clinical Trial. JAMA. 2016 Jan 5;315(1):36-46. doi: 10.1001/jama.2015.17346. PMID: 26746456; PMCID: PMC4787295.
	Justice JN, Pajewski NM, Espeland MA, Brubaker P, Houston DK, Marcovina S, Nicklas BJ, Kritchevsky SB, Kitzman DW. Evaluation of a blood-based geroscience biomarker index in a randomized trial of caloric restriction and exercise in older adults with heart failure with preserved ejection fraction. Geroscience. 2022 Apr;44(2):983-995. doi: 10.1007/s11357-021-00509-9. Epub 2022 Jan 10. PMID: 35013909; PMCID: PMC9135899.
	Upadhya B, Brubaker PH, Nicklas BJ, Houston DK, Haykowsky MJ, Kitzman DW. Long-term Changes in Body Composition and Exercise Capacity Following Calorie Restriction and Exercise Training in Older Patients with Obesity and Heart Failure With Preserved Ejection Fraction. J Card Fail. 2025 Mar;31(3):497-507. doi: 10.1016/j.cardfail.2024.06.007. Epub 2024 Jul 4.
Koh 2025	Koh S, Kim T, Son S, Kim M, Kim D. Progressive aerobic exercise in reducing inflammation and oxidative stress markers in older adult Korean women with obesity. Exp Gerontol. 2025 Oct 15;210:112868. doi: 10.1016/j.exger.2025.112868. Epub 2025 Aug 18. PMID: 40835078.
Kritchevsky 2017	Kritchevsky SB, Lovato L, Handing EP, Blair S, Botoseneanu A, Guralnik JM, Liu C, King A, Marsh AP, Pahor M, Rejeski WJ, Spring B, Manini T. Exercise's effect on mobility disability in older adults with and without obesity: The LIFE study randomized clinical trial. Obesity (Silver Spring). 2017 Jul;25(7):1199-1205. doi: 10.1002/oby.21860. PMID: 28653499; PMCID: PMC5567861.
Lambert 2008	Lambert CP, Wright NR, Finck BN, Villareal DT. Exercise but not diet-induced weight loss decreases skeletal muscle inflammatory gene expression in frail obese elderly persons. J Appl Physiol (1985). 2008 Aug;105(2):473-8. doi: 10.1152/japplphysiol.00006.2008. Epub 2008 Jun 5. PMID: 18535122; PMCID: PMC2519937.
Lee 2021	Lee YH, Lee PH, Lin LF, Liao CD, Liou TH, Huang SW. Effects of progressive elastic band resistance exercise for aged osteosarcopenic adiposity women. Exp Gerontol. 2021 May;147:111272. doi: 10.1016/j.exger.2021.111272. Epub 2021 Feb 4. PMID: 33549820.
Maillard 2016	Maillard F, Rousset S, Pereira B, Traore A, de Pradel Del Amaze P, Boirie Y, Duclos M, Boisseau N. High-intensity interval training reduces abdominal fat mass in postmenopausal women with type 2 diabetes. Diabetes Metab. 2016 Dec;42(6):433-441. doi: 10.1016/j.diabet.2016.07.031. Epub 2016 Aug 24. PMID: 27567125.
Manini 2010	Manini TM, Newman AB, Fielding R, Blair SN, Perri MG, Anton SD, Goodpaster BC, Katula JA, Rejeski WJ, Kritchevsky SB, Hsu FC, Pahor M, King AC; LIFE Research Group. Effects of exercise on mobility in obese and nonobese older adults. Obesity (Silver Spring). 2010 Jun;18(6):1168-75.

	doi: 10.1038/oby.2009.317. Epub 2009 Oct 15. Erratum in: Obesity (Silver Spring).2011 May;19(5):1106. King, Abby C [added]. PMID: 19834467; PMCID: PMC3114403.
Mesinovic 2025	Mesinovic J, Gandham A, Cervo MM, Jansons P, Glavas C, Braude M, Rodriguez JP, De Courten B, Zengin A, Beck BR, Ebeling PR, Scott D. Resistance and Impact Training During Weight Loss Improves Physical Function and Body Composition in Older Adults With Obesity. J Cachexia Sarcopenia Muscle. 2025 Apr;16(2):e13789. doi: 10.1002/jcsm.13789.
	Glavas C, Mesinovic J, Gandham A, Cervo MM, Ng CA, Ebeling PR, George ES, Daly RM, Beck BR, Jansons P, Scott D. Experiences and outcomes of older adults with obesity transitioning from gym- to home-based resistance training due to COVID-19 lockdowns: a mixed-methods analysis of a RCT. BMC Geriatr. 2025 Jul 29;25(1):556. doi: 10.1186/s12877-025-06247-3.
Miller 2006	Miller GD, Nicklas BJ, Davis C, Loeser RF, Lenchik L, Messier SP. Intensive weight loss program improves physical function in older obese adults with knee osteoarthritis. Obesity (Silver Spring). 2006 Jul;14(7):1219-30. doi: 10.1038/oby.2006.139. PMID: 16899803.
	Wang X, Miller GD, Messier SP, Nicklas BJ. Knee strength maintained despite loss of lean body mass during weight loss in older obese adults with knee osteoarthritis. J Gerontol A Biol Sci Med Sci. 2007 Aug;62(8):866-71. doi: 10.1093/gerona/62.8.866. PMID: 17702878.
	Miller GD, Nicklas BJ, Loeser RF. Inflammatory biomarkers and physical function in older, obese adults with knee pain and self-reported osteoarthritis after intensive weight-loss therapy. J Am Geriatr Soc. 2008 Apr;56(4):644-51. doi: 10.1111/j.1532-5415.2007.01636.x. Epub 2008 Feb 28. PMID: 18312558.
	Miller GD. Improved nutrient intake in older obese adults undergoing a structured diet and exercise intentional weight loss program. J Nutr Health Aging. 2010 Jun;14(6):461-6. doi: 10.1007/s12603-010-0100-3. PMID: 20617289.
	Miller GD, Jenks MZ, Vendela M, Norris JL, Muday GK. Influence of weight loss, body composition, and lifestyle behaviors on plasma adipokines: a randomized weight loss trial in older men and women with symptomatic knee osteoarthritis. J Obes. 2012;2012:708505. doi: 10.1155/2012/708505. Epub 2012 Dec 26. PMID: 23326650; PMCID: PMC3541640.
	Miller GD, Robinson SL. Impact of Body Composition on Physical Performance Tasks in Older Obese Women Undergoing a Moderate Weight Loss Program. J Frailty Aging. 2013;2(1):27-32. doi: 10.14283/jfa.2013.5. PMID: 27070455.
Muscariello 2016	Muscariello E, Nasti G, Siervo M, Di Maro M, Lapi D, D'Addio G, Colantuoni A. Dietary protein intake in sarcopenic obese older women. Clin Interv Aging. 2016 Feb 5;11:133-40. doi: 10.2147/CIA.S96017. PMID: 26917955; PMCID: PMC4751896.
Nabuco 2019	Nabuco HCG, Tomeleri CM, Fernandes RR, Sugihara Junior P, Cavalcante EF, Cunha PM, Antunes M, Nunes JP, Venturini D, Barbosa DS, Burini RC, Silva AM, Sardinha LB, Cyrino ES. Effect of whey protein supplementation combined with resistance training on body composition, muscular strength, functional capacity, and plasma-metabolism biomarkers in older women with sarcopenic obesity: A randomized, double-blind, placebo-controlled trial. Clin Nutr ESPEN. 2019 Aug;32:88-95. doi: 10.1016/j.clnesp.2019.04.007. Epub 2019 May 13. PMID: 31221297.

Nicklas 2014	Nicklas BJ, Gaukstern JE, Beavers KM, Newman JC, Leng X, Rejeski WJ. Self-monitoring of spontaneous physical activity and sedentary behavior to prevent weight regain in older adults. Obesity (Silver Spring). 2014 Jun;22(6):1406-12. doi: 10.1002/oby.20732. Epub 2014 Mar 17. PMID: 24585701; PMCID: PMC4037357.
Nicklas 2019	Nicklas BJ, Brinkley TE, Houston DK, Lyles MF, Hugenschmidt CE, Beavers KM, Leng X. Effects of Caloric Restriction on Cardiorespiratory Fitness, Fatigue, and Disability Responses to Aerobic Exercise in Older Adults With Obesity: A Randomized Controlled Trial. J Gerontol A Biol Sci Med Sci. 2019 Jun 18;74(7):1084-1090. doi: 10.1093/gerona/gly159. PMID: 29982294; PMCID: PMC6580693.
	Hugenschmidt CE, Leng X, Lyles M, Michael L, Dougherty A, Babcock P, Baker LD, Brinkley TE, Nicklas BJ. Cognitive Effects of Adding Caloric Restriction to Aerobic Exercise Training in Older Adults with Obesity. Obesity (Silver Spring). 2019 Aug;27(8):1266-1274. doi: 10.1002/oby.22525. Epub 2019 Jun 14. PMID: 31199592; PMCID: PMC6656607.
	Brinkley TE, Leng I, Bailey MJ, Houston DK, Hugenschmidt CE, Nicklas BJ, Hundley WG. Effects of Exercise and Weight Loss on Proximal Aortic Stiffness in Older Adults With Obesity. Circulation. 2021 Aug 31;144(9):684-693. doi: 10.1161/CIRCULATIONAHA.120.051943. Epub 2021 Aug 2. PMID: 34333991; PMCID: PMC8405553.
Normandin 2018	Normandin E, Yow D, Crotts C, Kiel J, Beavers KM, Nicklas BJ. Feasibility of Weighted Vest Use during a Dietary Weight Loss Intervention and Effects on Body Composition and Physical Function in Older Adults. J Frailty Aging. 2018;7(3):198-203. doi: 10.14283/jfa.2018.17. PMID: 30095153; PMCID: PMC6489119.
	Kelleher JL, Beavers DP, Henderson RM, Yow D, Crotts C, Kiel J, Nicklas BJ, Beavers KM. Weighted Vest Use during Dietary Weight Loss on Bone Health in Older Adults with Obesity. J Osteoporos Phys Act. 2017;5(4):210. doi: 10.4172/2329-9509.1000210. Epub 2017 Nov 28. PMID: 29388604; PMCID: PMC5788462.
O'Leary 2007	O'Leary VB, Jorett AE, Marchetti CM, Gonzalez F, Phillips SA, Ciaraldi TP, Kirwan JP. Enhanced adiponectin multimer ratio and skeletal muscle adiponectin receptor expression following exercise training and diet in older insulin-resistant adults. Am J Physiol Endocrinol Metab. 2007 Jul;293(1):E421-7. doi: 10.1152/ajpendo.00123.2007. Epub 2007 May 8. PMID: 17488807
	Kelly KR, Brooks LM, Solomon TP, Kashyap SR, O'Leary VB, Kirwan JP. The glucose-dependent insulinotropic polypeptide and glucose-stimulated insulin response to exercise training and diet in obesity. Am J Physiol Endocrinol Metab. 2009 Jun;296(6):E1269-74. doi: 10.1152/ajpendo.00112.2009. Epub 2009 Apr 7. PMID: 19351807; PMCID: PMC2692394
Park 2017	Park J, Kwon Y, Park H. Effects of 24-Week Aerobic and Resistance Training on Carotid Artery Intima-Media Thickness and Flow Velocity in Elderly Women with Sarcopenic Obesity. J Atheroscler Thromb. 2017 Nov 1;24(11):1117-1124. doi: 10.5551/jat.39065. Epub 2017 Jul 1. PMID: 28674320; PMCID: PMC5684477.
Park 2019	Park HY, Jung WS, Kim J, Lim K. Twelve weeks of exercise modality in hypoxia enhances health-related function in obese older Korean men: A

	randomized controlled trial. Geriatr Gerontol Int. 2019 Apr;19(4):311-316. doi: 10.1111/ggi.13625. Epub 2019 Feb 20. PMID: 30788892.
Park 2020	Park W, Jung WS, Hong K, Kim YY, Kim SW, Park HY. Effects of Moderate Combined Resistance- and Aerobic-Exercise for 12 Weeks on Body Composition, Cardiometabolic Risk Factors, Blood Pressure, Arterial Stiffness, and Physical Functions, among Obese Older Men: A Pilot Study. Int J Environ Res Public Health. 2020 Oct 3;17(19):7233. doi: 10.3390/ijerph17197233. PMID: 33022918; PMCID: PMC7579509.
Park 2024	Park W, Park HY, Kim SW. Effects of 12 Weeks of Combined Exercise Training in Normobaric Hypoxia on Arterial Stiffness, Inflammatory Biomarkers, and Red Blood Cell Hemorheological Function in Obese Older Women. Healthcare (Basel). 2024 Sep 20;12(18):1887. doi: 10.3390/healthcare12181887.
Polo-Ferrero 2025	Polo-Ferrero L, Martin MJ, Puente-González AS, Barbero-Iglesias FJ, González-Manzano S, Méndez-Sánchez R. Efficacy of Power Training on Sarcopenic Obesity in Community-Dwelling Older Women: A 32-Week Randomized Clinical Trial. Nutrients. 2025 May 27;17(11):1822. doi: 10.3390/nu17111822.
Porter Starr 2016	Porter Starr KN, Pieper CF, Orenduff MC, McDonald SR, McClure LB, Zhou R, Payne ME, Bales CW. Improved Function With Enhanced Protein Intake per Meal: A Pilot Study of Weight Reduction in Frail, Obese Older Adults. J Gerontol A Biol Sci Med Sci. 2016 Oct;71(10):1369-75. doi: 10.1093/gerona/glv210. Epub 2016 Jan 18. PMID: 26786203; PMCID: PMC5018561.
	Porter Starr KN, McDonald SR, Jarman A, Orenduff M, Sloane R, Pieper CF, Bales CW. Markers of Renal Function in Older Adults Completing a Higher Protein Obesity Intervention and One Year Later: Findings from the MEASUR-UP Trial. J Nutr Gerontol Geriatr. 2018 Apr-Jun;37(2):117-129. doi: 10.1080/21551197.2018.1478696. Epub 2018 Jun 20. PMID: 29924703; PMCID: PMC6444359.
	Payne ME, Porter Starr KN, Orenduff M, Mulder HS, McDonald SR, Spira AP, Pieper CF, Bales CW. Quality of Life and Mental Health in Older Adults with Obesity and Frailty: Associations with a Weight Loss Intervention. J Nutr Health Aging. 2018;22(10):1259-1265. doi: 10.1007/s12603-018-1127-0. PMID: 30498835; PMCID: PMC6444357.
	Porter Starr KN, Orenduff M, McDonald SR, Mulder H, Sloane R, Pieper CF, Bales CW. Influence of Weight Reduction and Enhanced Protein Intake on Biomarkers of Inflammation in Older Adults with Obesity. J Nutr Gerontol Geriatr. 2019 Jan-Mar;38(1):33-49. doi: 10.1080/21551197.2018.1564200. Epub 2019 Feb 27. PMID: 30810500; PMCID: PMC6447442.
Prieto 2014	Prieto JA, Del Valle M, Nistal P, Méndez D, Abelairas-Gómez C, Barcala-Furelos R. Repercusion del ejercicio fisico en la composicion corporal y la capacidad aerobica de adultos mayores con obesidad mediante tres modelos de intervencion [Impact of exercise on the body composition and aerobic capacity of elderly with obesity through three models of intervention]. Nutr Hosp. 2014 Dec 17;31(3):1217-24. Spanish. doi: 10.3305/nh.2015.31.3.8434. PMID: 25726215.
Prieto 2015	Prieto JA, Del Valle M, Nistal P, Méndez D, Barcala-Furelos R, Abelairas-Gómez C. RELEVANCIA DE UN PROGRAMA DE EQUILIBRIO EN LA CALIDAD DE VIDA RELACIONADA CON LA SALUD DE MUJERES ADULTAS MAYORES OBESAS [RELEVANCE OF A PROGRAM BALANCE IN HEALTH-RELATED QUALITY OF LIFE OF OBESE ELDERLY WOMEN]. Nutr Hosp. 2015 Dec

	1;32(6):2800-7. Spanish. doi: 10.3305/nh.2015.32.6.9713. PMID: 26667737.
Rezaeipour 2021	Rezaeipour M. Effects of two water-based exercise programs on body weight and blood lipid parameters in elderly obese males with a sedentary lifestyle. Diabetes Metab Syndr. 2021 Jul-Aug;15(4):102194. doi: 10.1016/j.dsx.2021.102194. Epub 2021 Jun 30. PMID: 34242940.
Rosenberg 2020	Rosenberg DE, Anderson ML, Renz A, Matson TE, Lee AK, Greenwood-Hickman MA, Arterburn DE, Gardiner PA, Kerr J, McClure JB. Reducing Sitting Time in Obese Older Adults: The I-STAND Randomized Controlled Trial. J Aging Phys Act. 2020 Jun 4:1-11. doi: 10.1123/japa.2019-0470. Epub ahead of print. PMID: 32498040.
	Matson TE, Anderson ML, Renz AD, Greenwood-Hickman MA, McClure JB, Rosenberg DE. Changes in Self-Reported Health and Psychosocial Outcomes in Older Adults Enrolled in Sedentary Behavior Intervention Study. Am J Health Promot. 2019 Sep;33(7):1053-1057. doi: 10.1177/0890117119841405. Epub 2019 Apr 7. PMID: 30957508.
Rosenberg 2024	Rosenberg DE, Zhu W, Greenwood-Hickman MA, Cook AJ, Florez Acevedo S, McClure JB, Arterburn DE, Cooper J, Owen N, Dunstan D, Perry SR, Yarborough L, Mettert KD, Green BB. Sitting Time Reduction and Blood Pressure in Older Adults: A Randomized Clinical Trial. JAMA Netw Open. 2024 Mar 4;7(3):e243234. doi: 10.1001/jamanetworkopen.2024.3234. PMID: 38536177.
Rosety 2015	Rosety MA, Pery MT, Rodriguez-Pareja MA, Diaz A, Rosety J, Garcia N, Brenes-Martin F, Rosety-Rodríguez M, Toro R, Ordoñez FJ, Rosety I. A SHORT-TERM CIRCUIT RESISTANCE PROGRAMME REDUCED EPICARDIAL FAT IN OBESE AGED WOMEN. Nutr Hosp. 2015 Nov 1;32(5):2193-7. doi: 10.3305/nh.2015.32.5.9696. PMID: 26545677.
	Rosety I, Pery MT, Rosety J, García N, Rodríguez-Pareja MA, Brenes-Martín F, Díaz A, Rosety-Rodríguez M, Ordoñez FJ, Rosety MÁ. [Circuit resistance training improved endothelial dysfunction in obese aged women]. Nutr Hosp. 2016 Feb 16;33(1):17. Spanish. doi: 10.20960/nh.v33i1.17. PMID: 27019244.
Serra-Prat 2021	Serra-Prat M, Terradellas M, Lorenzo I, Arús M, Burdoy E, Salietti A, Ramírez S, Palomera E, Papiol M, Pleguezuelos E. Effectiveness of a Weight-Loss Intervention in Preventing Frailty and Functional Decline in Community-Dwelling Obese Older People. A Randomized Controlled Trial. J Frailty Aging (2021). https://doi.org/10.14283/jfa.2021.38
Shah 2009	Shah K, Stufflebam A, Hilton TN, Sinacore DR, Klein S, Villareal DT. Diet and exercise interventions reduce intrahepatic fat content and improve insulin sensitivity in obese older adults. Obesity (Silver Spring). 2009 Dec;17(12):2162-8. doi: 10.1038/oby.2009.126. Epub 2009 Apr 23. PMID: 19390517; PMCID: PMC2793412.
Solomon 2009	Solomon TP, Haus JM, Marchetti CM, Stanley WC, Kirwan JP. Effects of exercise training and diet on lipid kinetics during free fatty acid-induced insulin resistance in older obese humans with impaired glucose tolerance. Am J Physiol Endocrinol Metab. 2009 Aug;297(2):E552-9. doi: 10.1152/ajpendo.00220.2009. Epub 2009 Jun 16. PMID: 19531640; PMCID: PMC2724112.
	Haus JM, Solomon TP, Marchetti CM, Edmison JM, González F, Kirwan JP. Free fatty acid-induced hepatic insulin resistance is attenuated following lifestyle intervention in obese individuals with impaired glucose tolerance.

	J Clin Endocrinol Metab. 2010 Jan;95(1):323-7. doi: 10.1210/jc.2009-1101. Epub 2009 Nov 11. PMID: 19906790; PMCID: PMC2805494.
Solomon 2010	Solomon TP, Haus JM, Kelly KR, Cook MD, Filion J, Rocco M, Kashyap SR, Watanabe RM, Barkoukis H, Kirwan JP. A low-glycemic index diet combined with exercise reduces insulin resistance, postprandial hyperinsulinemia, and glucose-dependent insulinotropic polypeptide responses in obese, prediabetic humans. Am J Clin Nutr. 2010 Dec;92(6):1359-68. doi: 10.3945/ajcn.2010.29771. Epub 2010 Oct 27. PMID: 20980494; PMCID: PMC2980961.
	Kelly KR, Haus JM, Solomon TP, Patrick-Melin AJ, Cook M, Rocco M, Barkoukis H, Kirwan JP. A low-glycemic index diet and exercise intervention reduces TNF(alpha) in isolated mononuclear cells of older, obese adults. J Nutr. 2011 Jun;141(6):1089-94. doi: 10.3945/jn.111.139964. Epub 2011 Apr 27. PMID: 21525252; PMCID: PMC3095140.
	Malin SK, Niemi N, Solomon TP, Haus JM, Kelly KR, Filion J, Rocco M, Kashyap SR, Barkoukis H, Kirwan JP. Exercise training with weight loss and either a high- or low-glycemic index diet reduces metabolic syndrome severity in older adults. Ann Nutr Metab. 2012;61(2):135-41. doi: 10.1159/000342084. PMID: 23036993; PMCID: PMC3586384.
	Solomon TP, Haus JM, Cook MA, Flask CA, Kirwan JP. A low-glycemic diet lifestyle intervention improves fat utilization during exercise in older obese humans. Obesity (Silver Spring). 2013 Nov;21(11):2272-8. doi: 10.1002/oby.20411. Epub 2013 Jun 11. PMID: 23512711; PMCID: PMC3696477.
	Kelly KR, Navaneethan SD, Solomon TP, Haus JM, Cook M, Barkoukis H, Kirwan JP. Lifestyle-induced decrease in fat mass improves adiponectin secretion in obese adults. Med Sci Sports Exerc. 2014;46(5):920-6. doi: 10.1249/MSS.0000000000000200. PMID: 24614337; PMCID: PMC3991752
	Hari A, Fealy C, Solomon TPJ, Haus JM, Kelly KR, Barkoukis H, Kirwan JP. Exercise-induced improvements in glucose effectiveness are blunted by a high glycemic diet in adults with prediabetes. Acta Diabetol. 2019 Feb;56(2):211-217. doi: 10.1007/s00592-018-1272-2. Epub 2019 Jan 5. PMID: 30612202; PMCID: PMC6530476.
Son 2024a	Son WH, Ha MS, Park TJ. Effect of physical activity on free fatty acids, insulin resistance, and blood pressure in obese older women. Phys Act Nutr. 2024 Jun;28(2):1-6. doi: 10.20463/pan.2024.0009. Epub 2024 Jun 30.
Son 2024b	Son WH, Jeong WM, Park IY, Ha MS. Enhancing Inflammatory Factors, Nitric Oxide, and Arterial Stiffness Through Aquatic Walking for Amelioration and Disease Prevention: Targeting in Obese Elderly Women. Mediators Inflamm. 2024 Dec 23;2024:5520987. doi: 10.1155/mi/5520987.
Stillman 2018	Stillman CM, Donahue PT, Williams MF, Callas M, Lwanga C, Brown C, Wollam ME, Jedrziewski MK, Kang C, Erickson KI. Weight-Loss Outcomes from a Pilot Study of African Dance in Older African Americans. Obesity (Silver Spring). 2018 Dec;26(12):1893-1897. doi: 10.1002/oby.22331. Epub 2018 Oct 25. PMID: 30358132; PMCID: PMC6249065.
Travieso 2025	Travieso SG, Ortiz GU, Abud GF, Villalba MM, Junqueira GP, Gomes MM, Marchini JS, Freitas EC. Effects of Multicomponent Training on Metabolic and Functional Health of Older Women With Obesity: A Randomized Clinical Trial. J Aging Phys Act. 2025 Aug 26:1-13. doi: 10.1123/japa.2024-0341. Epub ahead of print.

Villareal 2006	Villareal DT, Banks M, Sinacore DR, Siener C, Klein S. Effect of weight loss and exercise on frailty in obese older adults. Arch Intern Med. 2006 Apr 24;166(8):860-6. doi: 10.1001/archinte.166.8.860. PMID: 16636211.
	Villareal DT, Miller BV 3rd, Banks M, Fontana L, Sinacore DR, Klein S. Effect of lifestyle intervention on metabolic coronary heart disease risk factors in obese older adults. Am J Clin Nutr. 2006 Dec;84(6):1317-23. doi: 10.1093/ajcn/84.6.1317. PMID: 17158411.
	Villareal DT, Banks MR, Patterson BW, Polonsky KS, Klein S. Weight loss therapy improves pancreatic endocrine function in obese older adults. Obesity (Silver Spring). 2008 Jun;16(6):1349-54. doi: 10.1038/oby.2008.226. Epub 2008 Apr 3. PMID: 18388888; PMCID: PMC2799929.
	Villareal DT, Shah K, Banks MR, Sinacore DR, Klein S. Effect of weight loss and exercise therapy on bone metabolism and mass in obese older adults: a one-year randomized controlled trial. J Clin Endocrinol Metab. 2008 Jun;93(6):2181-7. doi: 10.1210/jc.2007-1473. Epub 2008 Mar 25. PMID: 18364384; PMCID: PMC2435639.
	Frimel TN, Sinacore DR, Villareal DT. Exercise attenuates the weight-loss-induced reduction in muscle mass in frail obese older adults. Med Sci Sports Exerc. 2008 Jul;40(7):1213-9. doi: 10.1249/MSS.0b013e31816a85ce. PMID: 18580399; PMCID: PMC2650077.
Villareal 2011	Villareal DT, Chode S, Parimi N, Sinacore DR, Hilton T, Armamento-Villareal R, Napoli N, Qualls C, Shah K. Weight loss, exercise, or both and physical function in obese older adults. N Engl J Med. 2011 Mar 31;364(13):1218-29. doi: 10.1056/NEJMoa1008234. PMID: 21449785; PMCID: PMC3114602.
	Shah K, Armamento-Villareal R, Parimi N, Chode S, Sinacore DR, Hilton TN, Napoli N, Qualls C, Villareal DT. Exercise training in obese older adults prevents increase in bone turnover and attenuates decrease in hip bone mineral density induced by weight loss despite decline in bone-active hormones. J Bone Miner Res. 2011 Dec;26(12):2851-9. doi: 10.1002/jbmr.475. PMID: 21786319; PMCID: PMC3206995.
	Armamento-Villareal R, Sadler C, Napoli N, Shah K, Chode S, Sinacore DR, Qualls C, Villareal DT. Weight loss in obese older adults increases serum sclerostin and impairs hip geometry but both are prevented by exercise training. J Bone Miner Res. 2012 May;27(5):1215-21. doi: 10.1002/jbmr.1560. PMID: 22392834; PMCID: PMC3361603.
	Waters DL, Vawter R, Qualls C, Chode S, Armamento-Villareal R, Villareal DT. Long-term maintenance of weight loss after lifestyle intervention in frail, obese older adults. J Nutr Health Aging. 2013 Jan;17(1):3-7. doi: 10.1007/s12603-012-0421-5. PMID: 23299370; PMCID: PMC3580336.
	Armamento-Villareal R, Aguirre L, Napoli N, Shah K, Hilton T, Sinacore DR, Qualls C, Villareal DT. Changes in thigh muscle volume predict bone mineral density response to lifestyle therapy in frail, obese older adults. Osteoporos Int. 2014 Feb;25(2):551-8. doi: 10.1007/s00198-013-2450-2. Epub 2013 Jul 27. PMID: 23892583; PMCID: PMC3903658.
	Bouchonville M, Armamento-Villareal R, Shah K, Napoli N, Sinacore DR, Qualls C, Villareal DT. Weight loss, exercise or both and cardiometabolic risk factors in obese older adults: results of a randomized controlled trial. Int J Obes (Lond). 2014 Mar;38(3):423-31. doi: 10.1038/ijo.2013.122. Epub 2013 Jul 4. PMID: 23823329; PMCID: PMC3835728.
	Napoli N, Shah K, Waters DL, Sinacore DR, Qualls C, Villareal DT. Effect of weight loss, exercise, or both on cognition and quality of life in obese older

	adults. Am J Clin Nutr. 2014 Jul;100(1):189-98. doi: 10.3945/ajcn.113.082883. Epub 2014 Apr 30. PMID: 24787497; PMCID: PMC4144098.
	Armamento-Villareal R, Aguirre LE, Qualls C, Villareal DT. Effect of Lifestyle Intervention on the Hormonal Profile of Frail, Obese Older Men. J Nutr Health Aging. 2016 Mar;20(3):334-40. doi: 10.1007/s12603-016-0698-x. PMID: 26892583; PMCID: PMC4811358.
	Colleluori G, Napoli N, Phadnis U, Armamento-Villareal R, Villareal DT. Effect of Weight Loss, Exercise, or Both on Undercarboxylated Osteocalcin and Insulin Secretion in Frail, Obese Older Adults. Oxid Med Cell Longev. 2017;2017:4807046. doi: 10.1155/2017/4807046. Epub 2017 Aug 23. PMID: 28951766; PMCID: PMC5603129.
	Ho E, Qualls C, Villareal DT. Effect of Diet, Exercise, or Both on Biological Age and Healthy Aging in Older Adults with Obesity: Secondary Analysis of a Randomized Controlled Trial. J Nutr Health Aging. 2022;26(6):552-557. doi: 10.1007/s12603-022-1812-x. PMID: 35718862.
Villareal 2017	Villareal DT, Aguirre L, Gurney AB, Waters DL, Sinacore DR, Colombo E, Armamento-Villareal R, Qualls C. Aerobic or Resistance Exercise, or Both, in Dieting Obese Older Adults. N Engl J Med. 2017 May 18;376(20):1943-1955. doi: 10.1056/NEJMoa1616338. PMID: 28514618; PMCID: PMC5552187.
	Colleluori G, Aguirre L, Phadnis U, Fowler K, Armamento-Villareal R, Sun Z, Brunetti L, Hyoung Park J, Kaipparettu BA, Putluri N, Auetumrongsawat V, Yarasheski K, Qualls C, Villareal DT. Aerobic Plus Resistance Exercise in Obese Older Adults Improves Muscle Protein Synthesis and Preserves Myocellular Quality Despite Weight Loss. Cell Metab. 2019 Aug 6;30(2):261-273.e6. doi: 10.1016/j.cmet.2019.06.008. Epub 2019 Jul 3. PMID: 31279675; PMCID: PMC6685749.
	Armamento-Villareal R, Aguirre L, Waters DL, Napoli N, Qualls C, Villareal DT. Effect of Aerobic or Resistance Exercise, or Both, on Bone Mineral Density and Bone Metabolism in Obese Older Adults While Dieting: A Randomized Controlled Trial. J Bone Miner Res. 2020 Mar;35(3):430-439. doi: 10.1002/jbmr.3905. Epub 2019 Dec 4. PMID: 31797417; PMCID: PMC7064383.
	Waters DL, Aguirre L, Gurney AB, Sinacore DR, Fowler K, Gregori G, Armamento-Villareal R, Qualls C, Villareal DT. Effect of Aerobic or Resistance Exercise, or Both, on Intermuscular and Visceral Fat and Physical and Metabolic Function in Older Adults with Obesity While Dieting. J Gerontol A Biol Sci Med Sci. 2021 Apr 11:glab111. doi: 10.1093/gerona/glab111. Epub ahead of print. PMID: 33839788.
	Colleluori G, Viola V, Bathina S, Armamento-Villareal R, Qualls C, Giordano A, Villareal DT. Effect of aerobic or resistance exercise, or both on insulin secretion, ciliary neurotrophic factor, and insulin-like growth factor-1 in dieting older adults with obesity. Clin Nutr. 2025 Aug;51:50-62. doi: 10.1016/j.clnu.2025.05.016. Epub 2025 May 28.
Vincent 2014	Vincent HK, George SZ, Seay AN, Vincent KR, Hurley RW. Resistance exercise, disability, and pain catastrophizing in obese adults with back pain. Med Sci Sports Exerc. 2014 Sep;46(9):1693-701. doi: 10.1249/MSS.0000000000000294. PMID: 25133997; PMCID: PMC4137474.
	Vincent HK, Vincent KR, Seay AN, Conrad BP, Hurley RW, George SZ. Back strength predicts walking improvement in obese, older adults with chronic low back pain. PM R. 2014 May;6(5):418-26. doi:

	10.1016/j.pmrj.2013.11.002. Epub 2013 Nov 7. PMID: 24211698; PMCID: PMC4013252.
Wang 2023	Wang Y, Liu L, Chen Q, Chen Y, Lam WK. Pilot testing of a simplified dance intervention for cardiorespiratory fitness and blood lipids in obese older women. Geriatr Nurs. 2023 May-Jun;51:40-48. doi: 10.1016/j.gerinurse.2023.02.011. Epub 2023 Mar 4. PMID: 36878130.
West 2011	West DS, Bursac Z, Cornell CE, Felix HC, Fausett JK, Krukowski RA, Lensing S, Love SJ, Prewitt TE, Beck C. Lay health educators translate a weight-loss intervention in senior centers: a randomized controlled trial. Am J Prev Med. 2011 Oct;41(4):385-91. doi: 10.1016/j.amepre.2011.06.041. PMID: 21961465; PMCID: PMC6737925.
	Beck C, Fausett JK, Krukowski RA, Cornell CE, Prewitt TE, Lensing S, Bursac Z, Felix HC, Love S, McDougall G, West DS. A randomized trial of a community-based cognitive intervention for obese senior adults. J Aging Health. 2013 Feb;25(1):97-118. doi: 10.1177/0898264312467374. Epub 2012 Dec 16. PMID: 23248351; PMCID: PMC3947810.
Yassine 2009	Yassine HN, Marchetti CM, Krishnan RK, Vrobel TR, Gonzalez F, Kirwan JP. Effects of exercise and caloric restriction on insulin resistance and cardiometabolic risk factors in older obese adults--a randomized clinical trial. J Gerontol A Biol Sci Med Sci. 2009 Jan;64(1):90-5. doi: 10.1093/gerona/gln032. Epub 2009 Jan 20. PMID: 19164269; PMCID: PMC2691195.
Yin 2023	Yin YH, Liu JYW, Välimäki M. Dietary behaviour change intervention for managing sarcopenic obesity among community-dwelling older people: a pilot randomised controlled trial. BMC Geriatr. 2023 Sep 26;23(1):597. doi: 10.1186/s12877-023-04327-w. PMID: 37752447.
	Yin YH, Liu JYW, Välimäki M. "How difficult it is to change dietary behaviour" experience of older people with sarcopenic obesity: a qualitative study. BMC Geriatr. 2024 Jul 1;24(1):568. doi: 10.1186/s12877-024-05157-0.
Zhou 2021	Zhou M, Zhang N, Zhang Y, Yan X, Li M, Guo W, Guo X, He H, Guo K, Ma G. Effect of Mobile-Based Lifestyle Intervention on Weight Loss among the Overweight and Obese Elderly Population in China: A Randomized Controlled Trial. Int J Environ Res Public Health. 2021 Aug 21;18(16):8825. doi: 10.3390/ijerph18168825. PMID: 34444573; PMCID: PMC8393964.

Table S6: Detailed Characteristics of included studies (design, participants, interventions, outcomes, funding/COI)

To view table S6, please download file Supporting information 2 on OSF: https://osf.io/ayhq3?view_only=6e987edd80924f35b689f2b50ee3749b

Table S7: Detailed Description of intervention characteristics

To view table S7, please download file Supporting information 2 on OSF: https://osf.io/ayhq3?view_only=6e987edd80924f35b689f2b50ee3749b

Tables S8: GRADE evaluation for network meta-analyses

Table S8A: GRADE evaluation for NMA (intervention types) on physical function measured by performance batteries

	Direct evidence			Indirect evidence		Network meta-analysis	
**Comparison**	**N RCTs**	**SMD (95%-CI)**	**Certainty of evidence**	**SMD (95%-CI)**	**Certainty of evidence**	**SMD (95%-CI)**	**Certainty of evidence**
Control vs BCI	2	-0.04 [-3.24; 3.15]	⊕⊕⊕⊕	-	-	-0.04 [-3.24; 3.15]	⊕⊕◯◯[4]
Control vs Ex	4	-1.24 [-1.76; -0.72]	⊕⊕◯◯[2]	0.45 [-3.02; 3.92]	⊕⊕⊕⊕	-1.20 [-1.71; -0.69]	⊕◯◯◯[3]
Control vs Nut	2	0.23 [-2.23; 2.70]	⊕⊕◯◯[2]	-0.71 [-3.13; 1.71]	⊕⊕◯◯	-0.25 [-1.97; 1.48]	⊕◯◯◯[4]
Control vs Nut + BCI	2	-1.21 [-3.83; 1.40]	⊕⊕⊕◯[1]	0.71 [-5.91; 7.33]	⊕⊕◯◯	-0.95 [-3.38; 1.47]	⊕◯◯◯[4]
Control vs Nut + Ex	1	0.20 [-2.31; 2.71]	⊕⊕◯◯[2]	-0.64 [-2.91; 1.64]	⊕⊕◯◯	-0.26 [-1.94; 1.42]	⊕◯◯◯[4]
Control vs Nut + Ex + BCI	4	-3.46 [-5.08; -1.84]	⊕⊕⊕⊕	1.97 [-9.90; 13.84]		-3.37 [-4.97; -1.76]	⊕⊕⊕⊕
Ex vs Nut + BCI	1	0.90 [-4.72; 6.52]	⊕⊕⊕⊕	0.09 [-2.66; 2.84]	⊕⊕◯◯	0.25 [-2.22; 2.72]	⊕◯◯◯[4]
Ex vs Nut + Ex	1	-0.05 [-3.04; 2.94]	⊕⊕⊕◯[1]	1.43 [-0.66; 3.52]	⊕⊕◯◯	0.94 [-0.77; 2.66]	⊕◯◯◯[4]
Ex vs Nut + Ex + BCI	1	-1.40 [-8.19; 5.39]	⊕⊕⊕⊕	-2.21 [-3.95; -0.48]	⊕⊕◯◯	-2.16 [-3.85; -0.48]	⊕◯◯◯[4]
Ex + BCI vs Nut + Ex + BCI	1	-0.20 [-1.06; 0.67]	⊕⊕⊕⊕	-	-	-0.20 [-1.06; 0.67]	⊕⊕◯◯[4]
Nut vs Nut + Ex	3	0.01 [-0.69; 0.71]	⊕⊕◯◯[2]	-1.46 [-7.16; 4.25]	⊕⊕◯◯	-0.01 [-0.71; 0.68]	⊕◯◯◯[4]
Nut + BCI vs Nut + Ex + BCI	1	-2.30 [-7.75; 3.15]	⊕⊕⊕⊕	-2.45 [-5.72; 0.82]	⊕⊕⊕◯	-2.41 [-5.22; 0.39]	⊕◯◯◯[4]
BCI vs Ex	0	-	-	1.16 [-2.08; 4.40]	⊕⊕◯◯	1.16 [-2.08; 4.40]	⊕◯◯◯[4]
BCI vs Ex + BCI	0	-	-	3.12 [-0.56; 6.80]	⊕⊕⊕⊕	3.12 [-0.56; 6.80]	⊕⊕◯◯[4]
BCI vs Nut	0	-	-	0.20 [-3.43; 3.84]	⊕⊕◯◯	0.20 [-3.43; 3.84]	⊕◯◯◯[4]
BCI vs Nut + BCI	0	-	-	0.91 [-3.11; 4.93]	⊕⊕⊕◯	0.91 [-3.11; 4.93]	⊕◯◯◯[4]
BCI vs Nut + Ex	0	-	-	0.22 [-3.40; 3.83]	⊕⊕◯◯	0.22 [-3.40; 3.83]	⊕◯◯◯[4]
BCI vs Nut + Ex + BCI	0	-	-	3.32 [-0.26; 6.90]	⊕⊕⊕⊕	3.32 [-0.26; 6.90]	⊕⊕◯◯[4]
Control vs Ex + BCI	0	-	-	-3.17 [-4.99; -1.34]	⊕⊕⊕⊕	-3.17 [-4.99; -1.34]	⊕⊕⊕⊕
Ex vs Ex + BCI	0	-	-	-1.97 [-3.86; -0.07]	⊕⊕⊕⊕	-1.97 [-3.86; -0.07]	⊕⊕◯◯[4]
Ex vs Nut	0	-	-	0.96 [-0.80; 2.71]	⊕⊕◯◯	0.96 [-0.80; 2.71]	⊕◯◯◯[4]
Ex + BCI vs Nut	0	-	-	2.92 [0.41; 5.43]	⊕⊕◯◯	2.92 [0.41; 5.43]	⊕◯◯◯[4]
Ex + BCI vs Nut + BCI	0	-	-	2.21 [-0.72; 5.15]	⊕⊕⊕⊕	2.21 [-0.72; 5.15]	⊕⊕◯◯[4]
Ex + BCI vs Nut + Ex	0	-	-	2.91 [0.43; 5.39]	⊕⊕◯◯	2.91 [0.43; 5.39]	⊕◯◯◯[4]
Nut vs Nut + BCI	0	-	-	-0.71 [-3.69; 2.27]	⊕⊕◯◯	-0.71 [-3.69; 2.27]	⊕◯◯◯[4]
Nut vs Nut + Ex + BCI	0	-	-	-3.12 [-5.48; -0.76]	⊕⊕◯◯	-3.12 [-5.48; -0.76]	⊕◯◯◯[4]
Nut + BCI vs Nut + Ex	0	-	-	0.70 [-2.26; 3.65]	⊕⊕◯◯	0.70 [-2.26; 3.65]	⊕◯◯◯[4]
Nut + Ex vs Nut + Ex + BCI	0	-	-	-3.11 [-5.43; -0.78]	⊕⊕◯◯	-3.11 [-5.43; -0.78]	⊕◯◯◯[4]

⊕⊕⊕⊕ High; ⊕⊕⊕◯ Moderate; ⊕⊕◯◯ Low; ⊕◯◯◯ Very low

[1]once downgraded for RoB; [2]twice downgraded for RoB; [3]once downgraded for imprecision; [4]twice downgraded for imprecision

BCI Behavior Change Intervention, CI Confidence Interval, Ex Exercise, Nut Nutrition, RCT Randomized Controlled Trial, SMD Standardized Mean Difference

Table S8B: GRADE evaluation for NMA (intervention types) on physical function measured by VO_2max

	Direct evidence			Indirect evidence		Network meta-analysis	
**Comparison**	**N RCTs**	**MD (95%-CI)**	**Certainty of evidence**	**MD (95%-CI)**	**Certainty of evidence**	**MD (95%-CI)**	**Certainty of evidence**
Ex vs Con	8	2.10 [-0.04; 4.25]	⊕⊕◯◯[1,2]	-0.09 [-6.95; 6.77]	⊕⊕⊕◯	1.91 [-0.14; 3.96]	⊕◯◯◯[3]
Nut vs Con	1	1.40 [-2.18; 4.98]	⊕⊕⊕⊕	2.56 [-2.54; 7.67]	⊕⊕◯◯	1.78 [-1.15; 4.72]	⊕⊕⊕◯[3]
Nut + BCI vs Con	1	2.10 [-3.51; 7.71]	⊕⊕⊕⊕	1.63 [-4.27; 7.52]	⊕⊕◯◯	1.88 [-2.19; 5.94]	⊕⊕◯◯[4]
Nut + Ex vs Con	1	2.50 [-1.74; 6.74]	⊕⊕⊕⊕	3.96 [-1.95; 9.87]	⊕⊕◯◯	3.00 [-0.45; 6.44]	⊕⊕⊕◯[3]
Nut + Ex + BCI vs Con	4	2.18 [-0.91; 5.27]	⊕⊕⊕◯[2]	3.71 [-5.16; 12.57]	⊕⊕◯◯	2.35 [-0.57; 5.27]	⊕⊕◯◯[3]
Ex vs Nut	1	0.00 [-2.83; 2.83]	⊕⊕⊕⊕	0.94 [-6.28; 8.15]	⊕⊕◯◯	0.12 [-2.51; 2.76]	⊕⊕◯◯[4]
Ex vs Nut + BCI	1	-0.10 [-3.97; 3.77]	⊕⊕⊕⊕	2.87 [-14.98; 20.72]	⊕⊕⊕⊕	0.03 [-3.74; 3.81]	⊕⊕◯◯[4]
Ex vs Nut + Ex	3	-1.23 [-4.45; 1.99]	⊕⊕◯◯[1,2]	1.35 [-12.10; 14.80]	⊕⊕◯◯	-1.09 [-4.22; 2.05]	⊕◯◯◯[4]
Ex vs Nut + Ex + BCI	1	-1.50 [-6.42; 3.42]	⊕⊕⊕⊕	0.35 [-3.88; 4.58]	⊕⊕◯◯	-0.44 [-3.64; 2.77]	⊕◯◯◯[4]
Nut vs Nut + Ex	1	-1.10 [-4.68; 2.48]	⊕⊕⊕⊕	-2.21 [-12.81; 8.40]	⊕⊕◯◯	-1.21 [-4.61; 2.18]	⊕⊕◯◯[4]
Nut + BCI vs Nut + Ex + BCI	1	-1.40 [-7.01; 4.21]	⊕⊕⊕⊕	1.38 [-6.53; 9.29]	⊕⊕⊕⊕	-0.47 [-5.04; 4.10]	⊕⊕◯◯[4]
Nut vs Nut + BCI	-	-	-	-0.09 [-4.63; 4.45]	⊕⊕⊕⊕	-0.09 [-4.63; 4.45]	⊕⊕◯◯[4]
Nut vs Nut + Ex + BCI	-	-	-	-0.56 [-4.49; 3.36]	⊕⊕⊕⊕	-0.56 [-4.49; 3.36]	⊕⊕◯◯[4]
Nut + BCI vs Nut + Ex	-	-	-	-1.12 [-5.98; 3.74]	⊕⊕◯◯	-1.12 [-5.98; 3.74]	⊕◯◯◯[4]
Nut + Ex vs Nut + Ex + BCI	-	-	-	0.65 [-3.66; 4.96]	⊕⊕◯◯	0.65 [-3.66; 4.96]	⊕◯◯◯[4]

⊕⊕⊕⊕ High; ⊕⊕⊕◯ Moderate; ⊕⊕◯◯ Low; ⊕◯◯◯ Very low

[1]once downgraded for RoB; [2]once downgraded for indirectness; [3]once downgraded for imprecision; [4]twice downgraded for imprecision

BCI Behavior Change Intervention, CI Confidence Interval, Ex Exercise, MD Mean Difference, Nut Nutrition, RCT Randomized Controlled Trial

Table S8C: GRADE evaluation for NMA (intervention types) on fat free mass (kg)

	Direct evidence			Indirect evidence		Network meta-analysis	
**Comparison**	**N RCTs**	**MD (95%-CI)**	**Certainty of evidence**	**MD (95%-CI)**	**Certainty of evidence**	**MD (95%-CI)**	**Certainty of evidence**
Ex vs Con	6	0.64 [-1.41; 2.70]	⊕⊕◯◯[1,2]	0.80 [-3.55; 5.15]	⊕⊕⊕◯	0.67 [-1.19; 2.53]	⊕◯◯◯[4]
Nut vs Con	3	-2.55 [-5.48; 0.39]	⊕⊕⊕◯[1]	-0.64 [-6.20; 4.91]	⊕⊕◯◯	-2.13 [-4.72; 0.46]	⊕◯◯◯[4]
Nut + BCI vs Con	2	-1.42 [-5.69; 2.85]	⊕⊕◯◯[1,3]	-3.29 [-7.83; 1.24]	⊕⊕◯◯	-2.30 [-5.41; 0.81]	⊕◯◯◯[4]
Nut + Ex vs Con	2	-1.32 [-5.91; 3.28]	⊕⊕◯◯[1,3]	-1.62 [-5.20; 1.95]	⊕⊕◯◯	-1.51 [-4.33; 1.31]	⊕◯◯◯[4]
Nut + Ex + BCI vs Con	6	-1.28 [-3.64; 1.08]	⊕⊕⊕◯[1]	-1.87 [-6.95; 3.21]	⊕⊕◯◯	-1.38 [-3.52; 0.76]	⊕◯◯◯[4]
Ex vs Nut	1	2.00 [-8.49; 12.49]	⊕⊕⊕◯[3]	2.88 [-0.21; 5.96]	⊕⊕◯◯	2.81 [-0.15; 5.76]	⊕◯◯◯[4]
Ex vs Nut + BCI	2	4.38 [-0.57; 9.33]	⊕⊕⊕⊕	1.80 [-2.73; 6.32]	⊕⊕◯◯	2.97 [-0.37; 6.31]	⊕◯◯◯[4]
Ex vs Nut + Ex	6	1.34 [-3.33; 6.00]	⊕⊕⊕◯[1]	2.79 [-1.17; 6.75]	⊕⊕◯◯	2.18 [-0.84; 5.20]	⊕◯◯◯[4]
Ex vs Nut + Ex + BCI	1	3.10 [-1.48; 7.68]	⊕⊕⊕⊕	1.56 [-1.60; 4.72]	⊕⊕◯◯	2.06 [-0.54; 4.65]	⊕◯◯◯[4]
Ex + BCI vs Nut + Ex + BCI	1	0.56 [-0.32; 1.43]	⊕⊕⊕⊕	-	-	0.56 [-0.32; 1.43]	⊕⊕⊕◯[5]
Nut vs Nut + Ex	5	-0.29 [-2.86; 2.29]	⊕⊕◯◯[1,3]	-2.71 [-9.10; 3.68]	⊕⊕◯◯	-0.62 [-3.02; 1.77]	⊕◯◯◯[4]
Nut + BCI vs Nut + Ex + BCI	3	-1.29 [-4.91; 2.34]	⊕⊕⊕◯[1]	0.07 [-5.87; 6.01]	⊕⊕◯◯	-0.92 [-4.01; 2.18]	⊕◯◯◯[4]
Ex + BCI vs Con	-	-	-	-0.82 [-3.14; 1.49]	⊕⊕⊕◯	-0.82 [-3.14; 1.49]	⊕◯◯◯[4]
Ex vs Ex + BCI	-	-	-	1.50 [-1.24; 4.24]	⊕⊕⊕⊕	1.50 [-1.24; 4.24]	⊕⊕◯◯[5]
Ex + BCI vs Nut	-	-	-	1.31 [-2.13; 4.74]	⊕⊕⊕◯	1.31 [-2.13; 4.74]	⊕◯◯◯[4]
Ex + BCI vs Nut + BCI	-	-	-	1.48 [-1.74; 4.69]	⊕⊕⊕◯	1.48 [-1.74; 4.69]	⊕◯◯◯[4]
Ex + BCI vs Nut + Ex	-	-	-	0.68 [-2.90; 4.27]	⊕⊕◯◯	0.68 [-2.90; 4.27]	⊕◯◯◯[4]
Nut vs Nut + BCI	-	-	-	0.17 [-3.83; 4.17]	⊕⊕◯◯	0.17 [-3.83; 4.17]	⊕◯◯◯[4]
Nut vs Nut + Ex + BCI	-	-	-	-0.75 [-4.07; 2.57]	⊕⊕⊕◯	-0.75 [-4.07; 2.57]	⊕◯◯◯[4]
Nut + BCI vs Nut + Ex	-	-	-	-0.79 [-4.91; 3.33]	⊕⊕◯◯	-0.79 [-4.91; 3.33]	⊕◯◯◯[4]
Nut + Ex vs Nut + Ex + BCI	-	-	-	-0.12 [-3.61; 3.36]	⊕⊕◯◯	-0.12 [-3.61; 3.36]	⊕◯◯◯[4]

⊕⊕⊕⊕ High; ⊕⊕⊕◯ Moderate; ⊕⊕◯◯ Low; ⊕◯◯◯ Very low

[1]once downgraded for RoB; [2]once downgraded for inconsistency; [3]once downgraded for indirectness; [4]twice downgraded for imprecision, [5]once downgraded for imprecision;

BCI Behavior Change Intervention, CI Confidence Interval, Ex Exercise, MD Mean Difference, Nut Nutrition, RCT Randomized Controlled Trial

Table S8D: GRADE evaluation for NMA (intervention types) on fat mass (kg)

	Direct evidence			Indirect evidence		Network meta-analysis	
**Comparison**	**N RCTs**	**MD (95%-CI)**	**Certainty of evidence**	**MD (95%-CI)**	**Certainty of evidence**	**MD (95%-CI)**	**Certainty of evidence**
Con vs BCI	1	1.10 [0.12; 2.08]	⊕⊕⊕◯[1]	-	-	1.10 [0.12; 2.08]	⊕⊕◯◯[3]
Con vs Ex	6	2.43 [-0.97; 5.83]	⊕⊕⊕◯[1]	-0.60 [-6.09; 4.89]	⊕⊕◯◯	1.59 [-1.30; 4.48]	⊕◯◯◯[4]
Con vs Nut	4	1.92 [-1.56; 5.41]	⊕⊕◯◯[1,2]	4.10 [-2.88; 11.07]	⊕⊕◯◯	2.36 [-0.76; 5.47]	⊕◯◯◯[4]
Con vs Nut + BCI	3	6.00 [-0.68; 12.69]	⊕⊕◯◯[1,2]	6.39 [-2.60; 15.37]	⊕⊕⊕◯	6.14 [0.78; 11.50]	⊕◯◯◯[4]
Con vs Nut + Ex	3	3.15 [-0.33; 6.64]	⊕⊕◯◯[1]	3.59 [-1.85; 9.03]	⊕⊕⊕◯	3.28 [0.35; 6.22]	⊕◯◯◯[4]
Con vs Nut + Ex + BCI	6	6.97 [2.53; 11.42]	⊕⊕⊕◯[1]	5.40 [-2.34; 13.14]	⊕⊕⊕◯	6.58 [2.73; 10.44]	⊕◯◯◯[4]
Ex vs Nut	1	3.00 [-11.14; 17.14]	⊕⊕⊕⊕	0.56 [-3.69; 4.82]	⊕⊕◯◯	0.77 [-3.31; 4.84]	⊕◯◯◯[4]
Ex vs Nut + BCI	2	5.31 [-2.93; 13.55]	⊕⊕⊕⊕	3.90 [-3.71; 11.51]	⊕⊕◯◯	4.55 [-1.04; 10.14]	⊕◯◯◯[4]
Ex vs Nut + Ex	6	4.66 [-1.82; 11.15]	⊕⊕⊕◯[1]	0.23 [-4.32; 4.78]	⊕⊕◯◯	1.69 [-2.03; 5.42]	⊕◯◯◯[4]
Ex vs Nut + Ex + BCI	1	4.50 [-2.13; 11.13]	⊕⊕⊕⊕	5.35 [-0.26; 10.95]	⊕⊕⊕◯	4.99 [0.71; 9.27]	⊕◯◯◯[4]
Nut vs Nut + Ex	6	0.62 [-2.96; 4.19]	⊕⊕◯◯[1,2]	7.17 [-8.91; 23.26]	⊕⊕◯◯	0.93 [-2.56; 4.42]	⊕◯◯◯[4]
Nut + BCI vs Nut + Ex + BCI	3	-0.44 [-8.16; 7.28]	⊕⊕⊕◯[1]	1.66 [-7.40; 10.71]	⊕⊕◯◯	0.44 [-5.43; 6.32]	⊕◯◯◯[4]
Nut + Ex vs Nut + Ex + BCI	0	-	-	3.30 [-1.45; 8.05]	⊕⊕◯◯	3.30 [-1.45; 8.05]	⊕◯◯◯[4]
Ex vs BCI	0	-	-	-0.49 [-3.54; 2.56]	⊕⊕⊕◯	-0.49 [-3.54; 2.56]	⊕◯◯◯[4]
Nut vs BCI	0	-	-	-1.26 [-4.52; 2.01]	⊕⊕◯◯	-1.26 [-4.52; 2.01]	⊕◯◯◯[4]
Nut + BCI vs BCI	0	-	-	-5.04 [-10.49; 0.41]	⊕⊕◯◯	-5.04 [-10.49; 0.41]	⊕◯◯◯[4]
Nut + Ex vs BCI	0	-	-	-2.18 [-5.28; 0.91]	⊕⊕◯◯	-2.18 [-5.28; 0.91]	⊕◯◯◯[4]
Nut + Ex + BCI vs BCI	0	-	-	-5.48 [-9.46; -1.51]	⊕⊕⊕◯	-5.48 [-9.46; -1.51]	⊕◯◯◯[4]
Nut vs Nut + BCI	0	-	-	3.78 [-2.38; 9.95]	⊕⊕◯◯	3.78 [-2.38; 9.95]	⊕◯◯◯[4]
Nut vs Nut + Ex + BCI	0	-	-	4.23 [-0.69; 9.14]	⊕⊕◯◯	4.23 [-0.69; 9.14]	⊕◯◯◯[4]
Nut + BCI vs Nut + Ex	0	-	-	-2.86 [-8.88; 3.17]	⊕⊕◯◯	-2.86 [-8.88; 3.17]	⊕◯◯◯[4]

⊕⊕⊕⊕ High; ⊕⊕⊕◯ Moderate; ⊕⊕◯◯ Low; ⊕◯◯◯ Very low
[1]once downgraded for RoB; [2]once downgraded for indirectness, [3]once downgraded for imprecision; [4]twice downgraded for imprecision
BCI Behavior Change Intervention, CI Confidence Interval, Ex Exercise, MD Mean Difference, Nut Nutrition, RCT Randomized Controlled Trial

Table S8E: GRADE evaluation for NMA (intervention types) on body weight (kg)

	Direct evidence			Indirect evidence		Network meta-analysis	
**Comparison**	**N RCTs**	**MD (95%-CI)**	**Certainty of evidence**	**MD (95%-CI)**	**Certainty of evidence**	**MD (95%-CI)**	**Certainty of evidence**
Con vs BCI	4	1.30 [0.16; 2.44]	⊕⊕⊕◯[1]	-	-	1.30 [0.16; 2.44]	⊕⊕◯◯[4]
Con vs Ex	15	1.77 [-1.11; 4.66]	⊕⊕⊕◯[1]	-1.56 [-10.69; 7.57]	⊕⊕◯◯	1.47 [-1.28; 4.22]	⊕◯◯◯[5]
Con vs Nut	4	3.33 [-1.19; 7.85]	⊕⊕◯◯[2]	6.46 [-3.43; 16.36]	⊕⊕⊕◯	3.87 [-0.24; 7.99]	⊕◯◯◯[5]
Con vs Nut + BCI	4	6.36 [5.12; 7.59]	⊕⊕⊕◯[1]	8.60 [-9.45; 26.65]	⊕⊕⊕◯	6.37 [5.14; 7.60]	⊕⊕⊕◯
Con vs Nut + Ex	2	8.77 [-1.50; 19.05]	⊕⊕◯◯[1,3]	4.75 [-0.91; 10.41]	⊕⊕⊕◯	5.69 [0.73; 10.64]	⊕◯◯◯[5]
Con vs Nut + Ex + BCI	6	8.59 [3.50; 13.68]	⊕⊕⊕◯[1]	9.01 [-0.07; 18.10]	⊕⊕⊕◯	8.69 [4.25; 13.14]	⊕◯◯◯[5]
Ex vs Nut	1	3.00 [-11.14; 17.14]	⊕⊕⊕◯[3]	2.33 [-2.66; 7.32]	⊕⊕◯◯	2.40 [-2.30; 7.11]	⊕◯◯◯[5]
Ex vs Nut + BCI	2	8.79 [-3.44; 21.02]	⊕⊕⊕⊕	4.65 [1.56; 7.74]	⊕⊕◯◯	4.90 [1.90; 7.90]	⊕◯◯◯[4]
Ex vs Nut + Ex	6	5.81 [-1.88; 13.50]	⊕⊕⊕◯[1]	2.93 [-3.98; 9.84]	⊕⊕◯◯	4.22 [-0.92; 9.35]	⊕◯◯◯[5]
Ex vs Nut + Ex + BCI	1	8.10 [-2.16; 18.36]	⊕⊕⊕⊕	6.96 [1.27; 12.64]	⊕⊕⊕◯	7.22 [2.25; 12.20]	⊕◯◯◯[5]
Ex + BCI vs Nut + Ex + BCI	1	2.80 [0.83; 4.78]	⊕⊕⊕⊕	-	-	2.80 [0.83; 4.78]	⊕⊕⊕◯[4]
Nut vs Nut + Ex	5	1.04 [-5.17; 7.25]	⊕⊕⊕◯[1]	3.40 [-5.52; 12.33]	⊕⊕◯◯	1.81 [-3.29; 6.91]	⊕◯◯◯[5]
Nut + BCI vs Nut + Ex + BCI	3	-0.33 [-11.92; 11.25]	⊕⊕⊕◯[1]	2.82 [-2.17; 7.81]	⊕⊕◯◯	2.33 [-2.26; 6.91]	⊕◯◯◯[5]
Nut + Ex vs Nut + Ex + BCI	1	3.60 [-6.44; 13.64]	⊕⊕⊕◯[1]	2.70 [-4.56; 9.96]	⊕⊕◯◯	3.01 [-2.87; 8.89]	⊕◯◯◯[5]
Ex vs BCI	0	-	-	-0.17 [-3.14; 2.81]	⊕⊕⊕◯	-0.17 [-3.14; 2.81]	⊕◯◯◯[5]
Ex + BCI vs BCI	0	-	-	-4.59 [-9.58; 0.40]	⊕⊕⊕◯	-4.59 [-9.58; 0.40]	⊕◯◯◯[5]
Nut vs BCI	0	-	-	-2.57 [-6.84; 1.70]	⊕⊕◯◯	-2.57 [-6.84; 1.70]	⊕◯◯◯[4]
Nut + BCI vs BCI	0	-	-	-5.07 [-6.74; -3.39]	⊕⊕◯◯	-5.07 [-6.74; -3.39]	⊕◯◯◯[5]
Nut + Ex vs BCI	0	-	-	-4.38 [-9.47; 0.70]	⊕⊕◯◯	-4.38 [-9.47; 0.70]	⊕◯◯◯[5]
Nut + Ex + BCI vs BCI	0	-	-	-7.39 [-11.98; -2.81]	⊕⊕⊕◯	-7.39 [-11.98; -2.81]	⊕◯◯◯[5]
Con vs Ex + BCI	0	-	-	5.89 [1.03; 10.75]	⊕⊕⊕◯	5.89 [1.03; 10.75]	⊕◯◯◯[5]
Ex vs Ex + BCI	0	-	-	4.42 [-0.93; 9.77]	⊕⊕⊕⊕	4.42 [-0.93; 9.77]	⊕⊕◯◯[5]
Ex + BCI vs Nut	0	-	-	-2.02 [-8.14; 4.10]	⊕⊕◯◯	-2.02 [-8.14; 4.10]	⊕◯◯◯[5]
Ex + BCI vs Nut + BCI	0	-	-	0.48 [-4.51; 5.47]	⊕⊕⊕◯	0.48 [-4.51; 5.47]	⊕◯◯◯[5]
Ex + BCI vs Nut + Ex	0	-	-	-0.21 [-6.41; 6.00]	⊕⊕⊕◯	-0.21 [-6.41; 6.00]	⊕◯◯◯[5]
Nut vs Nut + BCI	0	-	-	2.49 [-1.79; 6.78]	⊕⊕◯◯	2.49 [-1.79; 6.78]	⊕◯◯◯[5]
Nut vs Nut + Ex + BCI	0	-	-	4.82 [-0.97; 10.61]	⊕⊕◯◯	4.82 [-0.97; 10.61]	⊕◯◯◯[5]
Nut + BCI vs Nut + Ex	0	-	-	-0.68 [-5.78; 4.42]	⊕⊕◯◯	-0.68 [-5.78; 4.42]	⊕◯◯◯[5]

⊕⊕⊕⊕ High; ⊕⊕⊕◯ Moderate; ⊕⊕◯◯ Low; ⊕◯◯◯ Very low

[1]once downgraded for RoB; [2]twice downgraded for RoB; [3]once downgraded for indirectness, [4]once downgraded for imprecision; [5]twice downgraded for imprecision

BCI Behavior Change Intervention, CI Confidence Interval, Ex Exercise, MD Mean Difference, Nut Nutrition, RCT Randomized Controlled Trial

Table S8F: GRADE evaluation for NMA (intervention types) on bone mineral density (total hip, g/cm²)

	Direct evidence			Indirect evidence		Network meta-analysis	
**Comparison**	**N RCTs**	**MD (95%-CI)**	**Certainty of evidence**	**MD (95%-CI)**	**Certainty of evidence**	**MD (95%-CI)**	**Certainty of evidence**
Ex vs Con	1	0.02 [-0.03; 0.07]	⊕⊕⊕⊕	-0.00 [-0.12; 0.11]	-	0.02 [-0.03; 0.06]	⊕⊕◯◯[2]
Nut + BCI vs Con	1	-0.02 [-0.08; 0.04]	⊕⊕⊕⊕	-0.05 [-0.22; 0.11]	-	-0.02 [-0.08; 0.03]	⊕⊕◯◯[2]
Ex vs Nut + Ex + BCI	1	0.02 [-0.03; 0.08]	⊕⊕⊕⊕	0.04 [-0.04; 0.13]	-	0.03 [-0.02; 0.08]	⊕⊕◯◯[2]
Nut + BCI vs Nut + Ex + BCI	1	-0.02 [-0.08; 0.05]	⊕⊕⊕⊕	0.01 [-0.11; 0.12]	-	-0.01 [-0.07; 0.05]	⊕⊕◯◯[2]
Nut + Ex + BCI vs Con	3	-0.01 [-0.05; 0.02]	⊕⊕⊕◯[1]	-	-	-0.01 [-0.05; 0.02]	⊕◯◯◯[2]
Ex vs Nut + BCI	1	0.04 [-0.01; 0.09]	⊕⊕⊕⊕	-	-	0.04 [-0.01; 0.09]	⊕⊕◯◯[2]

⊕⊕⊕⊕ High; ⊕⊕⊕◯ Moderate; ⊕⊕◯◯ Low; ⊕◯◯◯ Very low

[1]once downgraded for RoB; [2]twice downgraded for imprecision,

BCI Behavior Change Intervention, CI Confidence Interval, Ex Exercise, MD Mean Difference, Nut Nutrition, RCT Randomized Controlled Trial

Tables S9: League tables - results of pairwise and network meta-analyses of different intervention types (mean or standardized mean differences with 95% confidence intervals)

Abbreviations: BCI Behavior Change Intervention, Ex Exercise, Nut Nutrition,

Table S9A: League table - results of pairwise and network meta-analyses of different intervention types on performance batteries

Pairwise meta-analysis								
	BCI	0.04 [-3.15; 3.24]	.	.	.	.	.	.
	0.04 [-3.15; 3.24]	Con	**-1.24 [-1.76; -0.72]**	.	0.23 [-2.23; 2.70]	-1.21 [-3.83; 1.40]	0.20 [-2.31; 2.71]	**-3.46 [-5.08; -1.84]**
	-1.16 [-4.40; 2.08]	**-1.20 [-1.71; -0.69]**	Ex	.	.	0.90 [-4.72; 6.52]	-0.05 [-3.04; 2.94]	-1.40 [-8.19; 5.39]
	-3.12 [-6.80; 0.56]	**-3.17 [-4.99; -1.34]**	**-1.97 [-3.86; -0.07]**	Ex + BCI	.	.	.	-0.20 [-1.06; 0.67]
	-0.20 [-3.84; 3.43]	-0.25 [-1.97; 1.48]	0.96 [-0.80; 2.71]	**2.92 [0.41; 5.43]**	Nut	.	0.01 [-0.69; 0.71]	.
	-0.91 [-4.93; 3.11]	-0.95 [-3.38; 1.47]	0.25 [-2.22; 2.72]	2.21 [-0.72; 5.15]	-0.71 [-3.69; 2.27]	Nut + BCI	.	-2.30 [-7.75; 3.15]
	-0.22 [-3.83; 3.40]	-0.26 [-1.94; 1.42]	0.94 [-0.77; 2.66]	**2.91 [0.43; 5.39]**	-0.01 [-0.71; 0.68]	0.70 [-2.26; 3.65]	Nut + Ex	.
	-3.32 [-6.90; 0.26]	**-3.37 [-4.97; -1.76]**	**-2.16 [-3.85; -0.48]**	-0.20 [-1.06; 0.67]	**-3.12 [-5.48; -0.76]**	-2.41 [-5.22; 0.39]	**-3.11 [-5.43; -0.78]**	Nut + Ex + BCI
Network meta-analysis								

Table S9B: League table - results of pairwise and network meta-analyses of different intervention types on gait speed

Pairwise meta-analysis					
**Con**	-0.01 [-0.04; 0.01]	-0.02 [-0.40; 0.36]	-0.03 [-0.24; 0.18]	-0.04 [-0.15; 0.08]	-0.12 [-0.37; 0.14]
-0.02 [-0.04; 0.01]	**Ex**	.	0.06 [-0.48; 0.59]	0.01 [-0.16; 0.18]	-0.14 [-1.62; 1.33]
0.01 [-0.18; 0.20]	0.02 [-0.17; 0.22]	**Nut**	.	-0.04 [-0.23; 0.16]	.
-0.02 [-0.22; 0.18]	-0.00 [-0.20; 0.20]	-0.03 [-0.30; 0.25]	**Nut + BCI**	.	-0.20 [-1.60; 1.19]
-0.03 [-0.12; 0.06]	-0.01 [-0.11; 0.08]	-0.04 [-0.22; 0.14]	-0.01 [-0.23; 0.21]	**Nut + Ex**	.
-0.12 [-0.37; 0.14]	-0.10 [-0.36; 0.16]	-0.12 [-0.44; 0.19]	-0.10 [-0.42; 0.22]	-0.09 [-0.36; 0.18]	**Nut + Ex + BCI**
**Network meta-analysis**					

Table S9C: League table - results of pairwise and network meta-analyses of different intervention types on chair rise performance rate

Pairwise meta-analysis				
**BCI**	1.00 [0.73; 1.38]	.	.	.
1.00 [0.73; 1.38]	**Con**	**0.84 [0.74; 0.95]**	.	.
0.84 [0.59; 1.18]	**0.84 [0.74; 0.95]**	**Ex**	.	0.96 [0.77; 1.20]
0.74 [0.43; 1.28]	0.74 [0.47; 1.16]	0.88 [0.57; 1.36]	**Nut**	1.09 [0.75; 1.58]
0.80 [0.54; 1.21]	0.80 [0.63; 1.04]	0.96 [0.77; 1.20]	1.09 [0.75; 1.58]	**Nut + Ex**
**Network meta-analysis**				

Table S9D: League table - results of pairwise and network meta-analyses of different intervention types on handgrip strength

Pairwise meta-analysis						
**Con**	-1.92<br>[-5.25; 1.41]	.	-1.25<br>[-8.76; 6.26]	-1.21<br>[-10.37; 7.96]	-1.53<br>[-5.98; 2.92]	0.51<br>[-13.25; 14.27]
-1.97<br>[-5.24; 1.30]	**Ex**	.	.	.	-0.16<br>[-5.66; 5.34]	.
-0.59<br>[-14.62; 13.45]	1.39<br>[-13.02; 15.79]	**Ex + BCI**	.	.	.	1.10<br>[-1.67; 3.86]
-0.88<br>[-7.10; 5.34]	1.09<br>[-5.59; 7.78]	-0.29<br>[-15.64; 15.06]	**Nut**	.	-1.15<br>[-7.41; 5.11]	.
-1.21<br>[-10.37; 7.96]	0.77<br>[-8.96; 10.50]	-0.62<br>[-17.38; 16.14]	-0.33<br>[-11.41; 10.75]	**Nut + BCI**	.	.
-1.86<br>[-6.02; 2.31]	0.12<br>[-4.42; 4.65]	-1.27<br>[-15.91; 13.37]	-0.98<br>[-6.84; 4.89]	-0.65<br>[-10.72; 9.42]	**Nut + Ex**	.
0.51<br>[-13.25; 14.27]	2.48<br>[-11.66; 16.62]	1.10<br>[-1.67; 3.86]	1.39<br>[-13.71; 16.49]	1.72<br>[-14.81; 18.25]	2.37<br>[-12.01; 16.74]	**Nut + Ex + BCI**
Network meta-analysis						

Table S9E: League table - results of pairwise and network meta-analyses of different intervention types on VO2max

		**Pairwise meta-analysis**			
**Con**	-2.10 [-4.25; 0.04]	-1.40 [-4.98; 2.18]	-2.10 [-7.71; 3.51]	-2.50 [-6.74; 1.74]	-2.18 [-5.27; 0.91]
-1.91 [-3.96; 0.14]	**Ex**	0.00 [-2.83; 2.83]	-0.10 [-3.97; 3.77]	-1.23 [-4.45; 1.99]	-1.50 [-6.42; 3.42]
-1.78 [-4.72; 1.15]	0.12 [-2.51; 2.76]	**Nut**	.	-1.10 [-4.68; 2.48]	.
-1.88 [-5.94; 2.19]	0.03 [-3.74; 3.81]	-0.09 [-4.63; 4.45]	**Nut + BCI**	.	-1.40 [-7.01; 4.21]
-3.00 [-6.44; 0.45]	-1.09 [-4.22; 2.05]	-1.21 [-4.61; 2.18]	-1.12 [-5.98; 3.74]	**Nut + Ex**	.
-2.35 [-5.27; 0.57]	-0.44 [-3.64; 2.77]	-0.56 [-4.49; 3.36]	-0.47 [-5.04; 4.10]	0.65 [-3.66; 4.96]	**Nut + Ex + BCI**
		**Network meta-analysis**			

Table S9F: League table - results of pairwise and network meta-analyses of different intervention types on fat-free mass (kg)

Pairwise meta-analysis						
**Con**	-0.64<br>[-2.70; 1.41]	.	2.55<br>[-0.39; 5.48]	1.42<br>[-2.85; 5.69]	1.32<br>[-3.28; 5.91]	1.28<br>[-1.08; 3.64]
-0.67<br>[-2.53; 1.19]	**Ex**	.	2.00<br>[-8.49; 12.49]	4.38<br>[-0.57; 9.33]	1.34<br>[-3.33; 6.00]	3.10<br>[-1.48; 7.68]
0.82<br>[-1.49; 3.14]	1.50<br>[-1.24; 4.24]	**Ex + BCI**	.	.	.	0.56<br>[-0.32; 1.43]
2.13<br>[-0.46; 4.72]	2.81<br>[-0.15; 5.76]	1.31<br>[-2.13; 4.74]	**Nut**	.	-0.29<br>[-2.86; 2.29]	.
2.30<br>[-0.81; 5.41]	2.97<br>[-0.37; 6.31]	1.48<br>[-1.74; 4.69]	0.17<br>[-3.83; 4.17]	**Nut + BCI**	.	-1.29<br>[-4.91; 2.34]
1.51<br>[-1.31; 4.33]	2.18<br>[-0.84; 5.20]	0.68<br>[-2.90; 4.27]	-0.62<br>[-3.02; 1.77]	-0.79<br>[-4.91; 3.33]	**Nut + Ex**	.
1.38<br>[-0.76; 3.52]	2.06<br>[-0.54; 4.65]	0.56<br>[-0.32; 1.43]	-0.75<br>[-4.07; 2.57]	-0.92<br>[-4.01; 2.18]	-0.12<br>[-3.61; 3.36]	**Nut + Ex + BCI**
**Network meta-analysis**						

Table S9G: League table - results of pairwise and network meta-analyses of different intervention types on fat mass (kg)

Pairwise meta-analysis						
**BCI**	**-1.10 [-2.08; -0.12]**	.	.	.	.	.
**-1.10 [-2.08; -0.12]**	**Con**	2.43 [-0.97; 5.83]	1.92 [-1.56; 5.41]	6.00 [-0.68; 12.69]	3.15 [-0.33; 6.64]	**6.97 [2.53; 11.42]**
0.49 [-2.56; 3.54]	1.59 [-1.30; 4.48]	**Ex**	3.00 [-11.14; 17.14]	5.31 [-2.93; 13.55]	4.66 [-1.82; 11.15]	4.50 [-2.13; 11.13]
1.26 [-2.01; 4.52]	2.36 [-0.76; 5.47]	0.77 [-3.31; 4.84]	**Nut**	.	0.62 [-2.96; 4.19]	.
5.04 [-0.41; 10.49]	**6.14 [0.78; 11.50]**	4.55 [-1.04; 10.14]	3.78 [-2.38; 9.95]	**Nut + BCI**	.	-0.44 [-8.16; 7.28]
2.18 [-0.91; 5.28]	**3.28 [0.35; 6.22]**	1.69 [-2.03; 5.42]	0.93 [-2.56; 4.42]	-2.86 [-8.88; 3.17]	**Nut + Ex**	.
**5.48 [1.51; 9.46]**	**6.58 [2.73; 10.44]**	4.99 [0.71; 9.27]	4.23 [-0.69; 9.14]	0.44 [-5.43; 6.32]	3.30 [-1.45; 8.05]	**Nut + Ex + BCI**
Network meta-analysis						

Table S9H: League table - results of pairwise and network meta-analyses of different intervention types on waist circumference (cm)

Pairwise meta-analysis							
**BCI**	-0.88<br>[-2.28; 0.51]	.	.	.	.	.	.
-0.88<br>[-2.28; 0.51]	**Con**	2.83<br>[-2.05; 7.70]	.	2.53<br>[-9.29; 14.35]	5.62<br>[-9.05; 20.28]	5.90<br>[-10.20; 22.00]	8.72<br>[-0.93; 18.36]
1.67<br>[-3.17; 6.50]	2.55<br>[-2.08; 7.18]	**Ex**	.	.	4.40<br>[-20.14; 28.94]	3.66<br>[-4.46; 11.79]	4.20<br>[-22.74; 31.14]
6.40<br>[-3.36; 16.16]	7.28<br>[-2.38; 16.94]	4.73<br>[-5.88; 15.34]	**Ex + BCI**	.	.	.	1.15<br>[-0.24; 2.54]
3.41<br>[-6.61; 13.42]	4.29<br>[-5.63; 14.21]	1.74<br>[-8.52; 12.00]	-2.99<br>[-16.81; 10.83]	**Nut**	.	0.17<br>[-11.99; 12.32]	.
4.63<br>[-9.55; 18.81]	5.51<br>[-8.60; 19.62]	2.96<br>[-11.69; 17.61]	-1.77<br>[-18.29; 14.74]	1.22<br>[-15.97; 18.41]	**Nut + BCI**	.	-0.20<br>[-26.50; 26.10]
4.70<br>[-3.07; 12.48]	5.59<br>[-2.06; 13.23]	3.04<br>[-3.97; 10.05]	-1.69<br>[-13.95; 10.56]	1.30<br>[-8.76; 11.35]	0.08<br>[-15.84; 15.99]	**Nut + Ex**	.
7.55<br>[-2.11; 17.21]	8.43<br>[-1.13; 17.99]	5.88<br>[-4.64; 16.40]	1.15<br>[-0.24; 2.54]	4.14<br>[-9.61; 17.89]	2.92<br>[-13.54; 19.38]	2.84<br>[-9.33; 15.02]	**Nut + Ex + BCI**
**Network meta-analysis**							

Table S9I: League table - results of pairwise and network meta-analyses of different intervention types on body mass (kg)

Pairwise meta-analysis							
**BCI**	**-1.30 [-2.44; -0.16]**	.	.	.	.	.	.
**-1.30 [-2.44; -0.16]**	**Con**	1.77 [-1.11; 4.66]	.	3.33 [-1.19; 7.85]	**6.36 [5.12; 7.59]**	8.77 [-1.50; 19.05]	**8.59 [3.50; 13.68]**
0.17 [-2.81; 3.14]	1.47 [-1.28; 4.22]	**Ex**	.	3.00 [-11.14; 17.14]	8.79 [-3.44; 21.02]	5.81 [-1.88; 13.50]	8.10 [-2.16; 18.36]
4.59 [-0.40; 9.58]	**5.89 [1.03; 10.75]**	4.42 [-0.93; 9.77]	**Ex + BCI**	.	.	.	**2.80 [0.83; 4.78]**
2.57 [-1.70; 6.84]	3.87 [-0.24; 7.99]	2.40 [-2.30; 7.11]	-2.02 [-8.14; 4.10]	**Nut**	.	1.04 [-5.17; 7.25]	.
**5.07 [3.39; 6.74]**	**6.37 [5.14; 7.60]**	**4.90 [1.90; 7.90]**	0.48 [-4.51; 5.47]	2.49 [-1.79; 6.78]	**Nut + BCI**	.	-0.33 [-11.92; 11.25]
4.38 [-0.70; 9.47]	**5.69 [0.73; 10.64]**	4.22 [-0.92; 9.35]	-0.21 [-6.41; 6.00]	1.81 [-3.29; 6.91]	-0.68 [-5.78; 4.42]	**Nut + Ex**	3.60 [-6.44; 13.64]
**7.39 [2.81; 11.98]**	**8.69 [4.25; 13.14]**	**7.22 [2.25; 12.20]**	**2.80 [0.83; 4.78]**	4.82 [-0.97; 10.61]	2.33 [-2.26; 6.91]	3.01 [-2.87; 8.89]	**Nut + Ex + BCI**
Network meta-analysis							

Table S9J: League table - results of pairwise and network meta-analyses of different intervention types on bone mineral density of the total hip (BMD TH)

Pairwise meta-analysis			
**Con**	-0.02 [-0.07; 0.03]	0.02 [-0.04; 0.08]	0.01 [-0.02; 0.05]
-0.02 [-0.06; 0.03]	**Ex**	0.04 [-0.01; 0.09]	0.02 [-0.03; 0.08]
0.02 [-0.03; 0.08]	0.04 [-0.01; 0.09]	**Nut + BCI**	-0.02 [-0.08; 0.05]
0.01 [-0.02; 0.05]	0.03 [-0.02; 0.08]	-0.01 [-0.07; 0.05]	**Nut + Ex + BCI**
**Network meta-analysis**			

Tables S9: League tables - results of pairwise and network meta-analyses of different intervention modalities (mean or standardized mean differences with 95% confidence intervals)

Abbreviations: AE aerobic exercise; BCI behavior change intervention; Con control; CI confidence interval; CR calorie restriction; HP high protein; MD mean difference; RE resistance exercise; SMD standardized mean difference

Table S10A: League table - results of pairwise and network meta-analyses of different intervention modalities on performance batteries

Pairwise meta-analysis														
**AE**	**-0.53 [-0.81; -0.25]**	.	**1.00 [0.69; 1.31]**	.	-0.05 [-3.04; 2.94]	.	.	.	.	.	.	.	-0.18 [-0.49; 0.13]	.
**-0.53 [-0.81; -0.25]**	**AE + RE**	.	**1.52 [1.22; 1.83]**	.	.	.	.	-1.40 [-8.19; 5.39]	0.90 [-4.72; 6.52]	.	.	.	0.34 [0.04; 0.65]	.
0.95 [-2.26; 4.16]	1.48 [-1.74; 4.69]	**BCI**	0.04 [-3.15; 3.24]	.	.	.	.	.	.	.	.	.	.	.
**0.99 [0.69; 1.30]**	**1.52 [1.22; 1.82]**	0.04 [-3.15; 3.24]	**Con**	0.23 [-2.23; 2.70]	.	**-2.90 [-4.01; -1.79]**	0.20 [-2.31; 2.71]	**-4.41 [-5.46; -3.36]**	-2.90 [-7.37; 1.57]	-0.34 [-3.56; 2.88]	.	-2.90 [-4.01; -1.79]	**-1.19 [-1.52; -0.86]**	.
0.71 [-1.23; 2.64]	1.23 [-0.71; 3.17]	-0.24 [-3.98; 3.49]	-0.29 [-2.21; 1.64]	**CR**	0.05 [-0.69; 0.79]	.	-0.20 [-3.26; 2.86]	.	.	.	-0.50 [-3.54; 2.54]	.	.	.
0.71 [-1.25; 2.67]	1.24 [-0.73; 3.20]	-0.24 [-3.99; 3.51]	-0.28 [-2.24; 1.67]	0.00 [-0.72; 0.73]	**CR + AE**	.	.	.	.	.	.	.	.	.
**-1.84 [-2.97; -0.70]**	**-1.31 [-2.45; -0.18]**	-2.79 [-6.17; 0.59]	**-2.83 [-3.93; -1.74]**	**-2.54 [-4.76; -0.33]**	**-2.55 [-4.79; -0.31]**	**CR + AE + BCI**	.	**-1.60 [-2.71; -0.49]**	.	.	.	0.00 [-1.11; 1.11]	.	.
0.98 [-1.46; 3.42]	1.51 [-0.93; 3.95]	0.03 [-3.98; 4.05]	-0.01 [-2.44; 2.42]	0.28 [-2.43; 2.98]	0.27 [-2.48; 3.02]	**2.82 [0.16; 5.48]**	**CR + AE + RE**	.	.	.	.	.	.	.
**-3.37 [-4.46; -2.28]**	**-2.84 [-3.93; -1.75]**	**-4.32 [-7.68; -0.95]**	**-4.36 [-5.41; -3.31]**	**-4.08 [-6.27; -1.88]**	**-4.08 [-6.30; -1.86]**	**-1.53 [-2.63; -0.44]**	**-4.35 [-7.00; -1.71]**	**CR + AE + RE + BCI**	2.30 [-3.15; 7.75]	.	.	**1.60 [0.49; 2.71]**	.	.
-0.16 [-3.26; 2.95]	0.37 [-2.73; 3.48]	-1.10 [-5.56; 3.35]	-1.15 [-4.25; 1.95]	-0.86 [-4.51; 2.78]	-0.87 [-4.53; 2.80]	1.68 [-1.57; 4.94]	-1.14 [-5.07; 2.80]	**3.21 [0.01; 6.42]**	**CR + BCI**	-1.50 [-6.21; 3.21]	.	.	.	.
-0.08 [-2.93; 2.77]	0.45 [-2.40; 3.30]	-1.03 [-5.30; 3.24]	-1.07 [-3.91; 1.76]	-0.79 [-4.21; 2.64]	-0.79 [-4.23; 2.65]	1.76 [-1.27; 4.78]	-1.06 [-4.79; 2.67]	**3.29 [0.29; 6.29]**	0.07 [-3.32; 3.47]	**CR + HP + BCI**	.	.	.	.
0.21 [-3.40; 3.81]	0.73 [-2.88; 4.34]	-0.74 [-5.56; 4.08]	-0.79 [-4.39; 2.82]	-0.50 [-3.54; 2.54]	-0.50 [-3.63; 2.63]	2.04 [-1.72; 5.81]	-0.78 [-4.85; 3.30]	3.58 [-0.18; 7.33]	0.36 [-4.39; 5.11]	0.29 [-4.30; 4.87]	**CR + RE**	.	.	.
**-1.84 [-2.97; -0.70]**	**-1.31 [-2.45; -0.18]**	-2.79 [-6.17; 0.59]	**-2.83 [-3.93; -1.74]**	**-2.54 [-4.76; -0.33]**	**-2.55 [-4.79; -0.31]**	-0.00 [-1.11; 1.11]	**-2.82 [-5.48; -0.16]**	**1.53 [0.44; 2.63]**	-1.68 [-4.94; 1.57]	-1.76 [-4.78; 1.27]	-2.04 [-5.81; 1.72]	**CR + RE + BCI**	.	0.20 [-0.67; 1.06]
-0.19 [-0.50; 0.12]	**0.34 [0.03; 0.64]**	-1.14 [-4.35; 2.08]	**-1.18 [-1.51; -0.85]**	-0.90 [-2.84; 1.05]	-0.90 [-2.87; 1.07]	**1.65 [0.51; 2.79]**	-1.17 [-3.62; 1.28]	**3.18 [2.08; 4.28]**	-0.03 [-3.15; 3.08]	-0.11 [-2.96; 2.74]	-0.40 [-4.01; 3.22]	**1.65 [0.51; 2.79]**	**RE**	.
**-1.64 [-3.07; -0.21]**	-1.11 [-2.54; 0.32]	-2.59 [-6.08; 0.90]	**-2.63 [-4.03; -1.24]**	-2.35 [-4.73; 0.03]	-2.35 [-4.75; 0.05]	0.20 [-1.21; 1.60]	-2.62 [-5.42; 0.18]	**1.73 [0.33; 3.12]**	-1.48 [-4.85; 1.88]	-1.56 [-4.71; 1.59]	-1.85 [-5.71; 2.02]	0.20 [-0.67; 1.06]	**-1.45 [-2.88; -0.02]**	**RE + BCI**
Network meta-analysis														

Table S10B: League table - results of pairwise and network meta-analyses of different intervention modalities on gait speed

Pairwise meta-analysis														
**AE**	.	.	.	0.00 [-0.50; 0.50]	.	.	.	.	.	.	.	.	.	.
-0.01 [-0.78; 0.77]	**AE + RE**	0.01 [-0.01; 0.04]	.	.	.	.	-0.14 [-1.62; 1.33]	0.06 [-0.48; 0.59]	.	.	.	.	.	-0.10 [-0.57; 0.37]
0.01 [-0.76; 0.78]	0.01 [-0.01; 0.04]	**Con**	0.02 [-0.50; 0.54]	.	-0.14 [-0.52; 0.24]	0.05 [-0.37; 0.47]	-0.13 [-0.39; 0.12]	-0.06 [-0.46; 0.34]	-0.06 [-0.61; 0.49]	-0.02 [-0.26; 0.23]	.	-0.16 [-0.54; 0.22]	-0.04 [-0.16; 0.07]	-0.14 [-0.36; 0.08]
0.03 [-0.54; 0.60]	0.04 [-0.48; 0.56]	0.02 [-0.50; 0.54]	**CR**	-0.03 [-0.31; 0.25]	.	0.07 [-0.56; 0.71]	.	.	.	.	-0.07 [-0.37; 0.23]	.	.	.
0.00 [-0.50; 0.50]	0.01 [-0.58; 0.59]	-0.01 [-0.60; 0.58]	-0.03 [-0.31; 0.25]	**CR + AE**	.	.	.	.	.	.	.	.	.	.
-0.10 [-0.94; 0.75]	-0.09 [-0.44; 0.26]	-0.10 [-0.46; 0.25]	-0.13 [-0.75; 0.50]	-0.10 [-0.78; 0.59]	**CR + AE + BCI**	.	-0.07 [-0.45; 0.31]	.	.	.	.	-0.02 [-0.40; 0.36]	.	.
0.07 [-0.76; 0.89]	0.07 [-0.35; 0.49]	0.06 [-0.36; 0.48]	0.04 [-0.56; 0.63]	0.07 [-0.59; 0.72]	0.16 [-0.38; 0.71]	**CR + AE + RE**	.	.	.	.	.	.	.	.
-0.12 [-0.94; 0.69]	-0.12 [-0.37; 0.14]	-0.13 [-0.39; 0.12]	-0.15 [-0.73; 0.42]	-0.12 [-0.76; 0.52]	-0.03 [-0.38; 0.32]	-0.19 [-0.68; 0.30]	**CR + AE + RE + BCI**	0.20 [-1.19; 1.60]	.	.	.	0.05 [-0.33; 0.43]	.	.
-0.01 [-0.85; 0.83]	-0.01 [-0.34; 0.33]	-0.02 [-0.35; 0.31]	-0.04 [-0.66; 0.58]	-0.01 [-0.69; 0.66]	0.08 [-0.40; 0.57]	-0.08 [-0.61; 0.46]	0.11 [-0.30; 0.52]	**CR + BCI**	.	.	.	.	.	.
-0.05 [-1.00; 0.90]	-0.05 [-0.60; 0.51]	-0.06 [-0.61; 0.49]	-0.08 [-0.84; 0.68]	-0.05 [-0.86; 0.76]	0.04 [-0.61; 0.70]	-0.12 [-0.81; 0.58]	0.07 [-0.54; 0.68]	-0.04 [-0.69; 0.61]	**CR + HP**	.	.	.	.	.
-0.01 [-0.82; 0.80]	-0.00 [-0.25; 0.25]	-0.02 [-0.26; 0.23]	-0.04 [-0.61; 0.54]	-0.01 [-0.64; 0.63]	0.09 [-0.34; 0.52]	-0.07 [-0.56; 0.41]	0.12 [-0.24; 0.47]	0.01 [-0.41; 0.42]	0.04 [-0.56; 0.65]	**CR + HP + BCI**	.	.	.	.
-0.04 [-0.68; 0.60]	-0.03 [-0.63; 0.56]	-0.05 [-0.65; 0.55]	-0.07 [-0.37; 0.23]	-0.04 [-0.45; 0.37]	0.06 [-0.64; 0.75]	-0.11 [-0.77; 0.56]	0.08 [-0.57; 0.73]	-0.03 [-0.71; 0.66]	0.01 [-0.80; 0.83]	-0.03 [-0.68; 0.61]	**CR + RE**	.	.	.
-0.12 [-0.96; 0.73]	-0.11 [-0.46; 0.24]	-0.12 [-0.48; 0.23]	-0.15 [-0.77; 0.48]	-0.12 [-0.80; 0.57]	-0.02 [-0.40; 0.36]	-0.18 [-0.73; 0.36]	0.01 [-0.34; 0.36]	-0.10 [-0.59; 0.38]	-0.06 [-0.72; 0.59]	-0.11 [-0.54; 0.32]	-0.08 [-0.77; 0.62]	**CR + RE + BCI**	.	.
-0.04 [-0.82; 0.73]	-0.04 [-0.15; 0.07]	-0.05 [-0.16; 0.05]	-0.07 [-0.60; 0.46]	-0.04 [-0.64; 0.55]	0.05 [-0.32; 0.42]	-0.11 [-0.54; 0.32]	0.08 [-0.20; 0.36]	-0.03 [-0.38; 0.32]	0.01 [-0.56; 0.57]	-0.04 [-0.31; 0.23]	-0.00 [-0.61; 0.60]	0.07 [-0.30; 0.44]	**HP + RE**	-0.02 [-0.20; 0.17]
-0.09 [-0.87; 0.70]	-0.08 [-0.24; 0.07]	-0.10 [-0.25; 0.06]	-0.12 [-0.66; 0.42]	-0.09 [-0.70; 0.52]	0.01 [-0.38; 0.39]	-0.16 [-0.60; 0.29]	0.04 [-0.26; 0.33]	-0.08 [-0.44; 0.29]	-0.04 [-0.61; 0.54]	-0.08 [-0.37; 0.21]	-0.05 [-0.67; 0.57]	0.03 [-0.36; 0.41]	-0.04 [-0.19; 0.10]	**RE**
Network meta-analysis														

Table S10C: League table - results of pairwise and network meta-analyses of different intervention modalities on chair rise performance rate

**Pairwise meta-analysis**				
**AE + RE**	.	1.11 [0.88; 1.39]	.	.
1.11 [0.73; 1.69]	**BCI**	1.00 [0.70; 1.43]	.	.
1.11 [0.88; 1.39]	1.00 [0.70; 1.43]	**Con**	.	**0.80 [0.67; 0.96]**
0.89 [0.52; 1.50]	0.80 [0.44; 1.44]	0.80 [0.50; 1.28]	**HP + RE**	1.00 [0.65; 1.55]
0.89 [0.66; 1.19]	0.80 [0.53; 1.19]	**0.80 [0.67; 0.96]**	1.00 [0.65; 1.55]	**RE**
**Network meta-analysis**				

Table S10D: League table - results of pairwise and network meta-analyses of different intervention modalities on handgrip strength

Pairwise meta-analysis							
**AE + RE**	2.58 [-1.83; 6.99]	.	.	.	.	.	-0.10 [-11.35; 11.15]
2.48 [-1.82; 6.77]	**Con**	0.51 [-13.25; 14.27]	.	-1.21 [-10.37; 7.96]	-1.25 [-8.76; 6.26]	-1.53 [-5.98; 2.92]	-1.17 [-5.86; 3.52]
2.99 [-11.42; 17.40]	0.51 [-13.25; 14.27]	**CR + AE + RE + BCI**	.	.	.	.	.
1.07 [-12.57; 14.71]	-1.41 [-14.36; 11.54]	-1.92 [-20.81; 16.97]	**CR + BCI**	0.20 [-8.95; 9.35]	.	.	.
1.27 [-8.85; 11.39]	-1.21 [-10.37; 7.96]	-1.72 [-18.25; 14.81]	0.20 [-8.95; 9.35]	**CR + HP + BCI**	.	.	.
1.54 [-6.51; 9.60]	-0.93 [-7.80; 5.93]	-1.45 [-16.82; 13.93]	0.47 [-14.18; 15.13]	0.27 [-11.18; 11.73]	**HP**	-1.00 [-8.49; 6.49]	.
0.86 [-5.14; 6.85]	-1.62 [-5.97; 2.73]	-2.13 [-16.56; 12.30]	-0.21 [-13.87; 13.45]	-0.41 [-10.56; 9.73]	-0.69 [-7.55; 6.18]	**HP + RE**	0.16 [-5.34; 5.66]
1.07 [-4.75; 6.88]	-1.41 [-5.83; 3.00]	-1.92 [-16.37; 12.52]	-0.01 [-13.69; 13.68]	-0.21 [-10.38; 9.97]	-0.48 [-8.20; 7.25]	0.21 [-4.74; 5.15]	**RE**
Network meta-analysis							

Table S10E: League table - results of pairwise and network meta-analyses of different intervention modalities on VO2max

Pairwise meta-analysis

**AE**	0.20 [-10.24; 10.64]	2.41 [-0.27; 5.10]	-1.71 [-8.73; 5.31]	.	.	.	0.00 [-2.83; 2.83]	-1.10 [-4.73; 2.53]	.	.	2.80 [-5.94; 11.54]
0.38 [-3.33; 4.09]	**AE + RE**	2.32 [-0.51; 5.15]	.	.	-1.70 [-6.80; 3.40]	-0.30 [-5.22; 4.62]	.	.	.	.	2.60 [-8.18; 13.38]
2.36 [-0.29; 5.02]	1.99 [-0.69; 4.66]	**Con**	.	-3.20 [-8.46; 2.06]	-2.59 [-5.52; 0.35]	-2.60 [-7.98; 2.78]	-1.40 [-4.98; 2.18]	-2.50 [-6.74; 1.74]	.	-1.20 [-6.46; 4.06]	-2.03 [-9.33; 5.26]
-1.71 [-8.73; 5.31]	-2.09 [-10.03; 5.85]	-4.07 [-11.58; 3.43]	**CR + AE**	.	.	.	.	.	.	.	.
-0.64 [-6.10; 4.83]	-1.02 [-6.35; 4.31]	-3.00 [-7.78; 1.78]	1.07 [-7.82; 9.97]	**CR + AE + BCI**	0.20 [-5.06; 5.46]	.	.	.	.	2.00 [-3.26; 7.26]	.
-0.24 [-4.16; 3.68]	-0.62 [-4.14; 2.90]	-2.60 [-5.51; 0.30]	1.47 [-6.57; 9.51]	0.40 [-4.38; 5.18]	**CR + AE + RE + BCI**	1.40 [-5.12; 7.92]	.	.	.	1.80 [-3.46; 7.06]	.
0.11 [-5.59; 5.81]	-0.27 [-5.14; 4.60]	-2.26 [-7.33; 2.82]	1.82 [-7.22; 10.86]	0.75 [-6.09; 7.58]	0.35 [-5.16; 5.86]	**CR + BCI**	.	.	.	.	.
0.31 [-2.41; 3.02]	-0.07 [-4.18; 4.03]	-2.06 [-5.22; 1.10]	2.01 [-5.51; 9.54]	0.94 [-4.79; 6.67]	0.54 [-3.74; 4.83]	0.20 [-5.76; 6.15]	**CR + HP**	-1.10 [-4.68; 2.48]	.	.	.
-0.79 [-4.34; 2.75]	-1.17 [-5.86; 3.51]	-3.16 [-7.05; 0.73]	0.91 [-6.95; 8.78]	-0.16 [-6.32; 6.00]	-0.56 [-5.41; 4.29]	-0.90 [-7.28; 5.47]	-1.10 [-4.68; 2.48]	**CR + HP + AE**	-0.20 [-4.59; 4.19]	.	.
-0.99 [-6.63; 4.64]	-1.37 [-7.80; 5.05]	-3.36 [-9.22; 2.51]	0.71 [-8.29; 9.72]	-0.36 [-7.92; 7.21]	-0.76 [-7.30; 5.78]	-1.10 [-8.85; 6.64]	-1.30 [-6.96; 4.36]	-0.20 [-4.59; 4.19]	**CR + HP + AE + RE**	.	.
1.36 [-4.10; 6.83]	0.98 [-4.35; 6.31]	-1.00 [-5.78; 3.78]	3.07 [-5.82; 11.97]	2.00 [-3.26; 7.26]	1.60 [-3.18; 6.38]	1.25 [-5.58; 8.09]	1.06 [-4.67; 6.79]	2.16 [-4.00; 8.32]	2.36 [-5.21; 9.92]	**CR + RE + BCI**	.
1.50 [-5.31; 8.31]	1.12 [-5.86; 8.10]	-0.86 [-7.47; 5.75]	3.21 [-6.57; 12.99]	2.14 [-6.00; 10.28]	1.74 [-5.45; 8.93]	1.39 [-6.86; 9.64]	1.20 [-5.92; 8.32]	2.30 [-5.18; 9.77]	2.50 [-6.17; 11.16]	0.14 [-8.00; 8.28]	**RE**

Network meta-analysis

Table S10F: League table - results of pairwise and network meta-analyses of different intervention modalities on fat-free mass (kg)

Pairwise meta-analysis																	
**AE**	.	-1.00 [-11.50; 9.50]	.	1.74 [-4.58; 8.06]	.	.	.	.	2.00 [-8.49; 12.49]	2.00 [-12.28; 16.28]	.	.	.	.	.	.	.
-1.17 [-8.56; 6.21]	**AE + RE**	0.48 [-1.66; 2.63]	.	.	.	.	2.80 [-2.37; 7.97]	4.38 [-2.16; 10.92]	.	.	.	.	.	.	.	.	.
-0.64 [-7.73; 6.45]	0.53 [-1.54; 2.61]	**Con**	1.48 [-3.89; 6.85]	.	2.70 [-1.77; 7.17]	0.90 [-4.23; 6.03]	1.26 [-1.05; 3.56]	2.80 [-4.15; 9.75]	3.00 [-0.50; 6.50]	3.00 [-7.31; 13.31]	0.57 [-5.26; 6.40]	.	.	1.00 [-3.47; 5.47]	.	-6.30 [-18.50; 5.90]	.
0.93 [-5.67; 7.54]	2.11 [-3.28; 7.49]	1.57 [-3.40; 6.54]	**CR**	0.60 [-4.27; 5.47]	.	-0.60 [-6.15; 4.95]	.	.	.	.	.	.	-0.20 [-5.62; 5.22]	.	.	.	.
1.61 [-4.05; 7.27]	2.78 [-3.79; 9.36]	2.25 [-3.99; 8.49]	0.68 [-3.90; 5.25]	**CR + AE**	.	.	.	.	.	.	.	.	.	.	.	.	.
1.91 [-6.18; 9.99]	3.08 [-1.25; 7.42]	2.55 [-1.34; 6.44]	0.98 [-5.34; 7.29]	0.30 [-7.05; 7.65]	**CR + AE + BCI**	.	-1.00 [-6.26; 4.26]	0.39 [-7.26; 8.04]	.	.	.	.	.	-1.70 [-6.96; 3.56]	.	.	.
0.29 [-7.61; 8.19]	1.46 [-3.99; 6.92]	0.93 [-4.12; 5.98]	-0.64 [-6.05; 4.77]	-1.32 [-8.12; 5.48]	-1.62 [-7.99; 4.75]	**CR + AE + RE**	.	.	.	.	.	.	.	.	.	.	.
0.69 [-6.72; 8.11]	1.87 [-0.93; 4.67]	1.33 [-0.83; 3.50]	-0.24 [-5.66; 5.18]	-0.92 [-7.52; 5.69]	-1.21 [-5.27; 2.84]	0.40 [-5.09; 5.90]	**CR + AE + RE + BCI**	1.80 [-2.78; 6.38]	.	.	.	.	.	-0.70 [-5.96; 4.56]	.	.	.
2.17 [-5.84; 10.19]	3.35 [-0.73; 7.42]	2.82 [-0.92; 6.55]	1.24 [-4.98; 7.46]	0.56 [-6.70; 7.83]	0.27 [-4.35; 4.88]	1.88 [-4.39; 8.16]	1.48 [-2.23; 5.19]	**CR + BCI**	.	.	-0.70 [-7.10; 5.70]	.	.	.	.	.	.
2.34 [-5.19; 9.86]	3.51 [-0.53; 7.56]	2.98 [-0.49; 6.45]	1.41 [-4.51; 7.32]	0.73 [-6.18; 7.64]	0.43 [-4.78; 5.64]	2.05 [-4.02; 8.11]	1.65 [-2.45; 5.74]	0.16 [-4.93; 5.26]	**CR + HP**	0.00 [-10.30; 10.30]	.	-1.30 [-6.87; 4.27]	.	.	.	.	.
2.34 [-9.93; 14.61]	3.51 [-6.99; 14.02]	2.98 [-7.32; 13.28]	1.41 [-9.95; 12.76]	0.73 [-11.18; 12.63]	0.43 [-10.58; 11.44]	2.05 [-9.39; 13.48]	1.65 [-8.88; 12.17]	0.16 [-10.79; 11.12]	0.00 [-10.30; 10.30]	**CR + HP + AE**	.	.	.	.	.	.	.
0.63 [-7.84; 9.10]	1.80 [-3.20; 6.81]	1.27 [-3.36; 5.90]	-0.30 [-7.10; 6.49]	-0.98 [-8.75; 6.79]	-1.28 [-7.03; 4.47]	0.34 [-6.51; 7.19]	-0.06 [-4.96; 4.83]	-1.54 [-6.31; 3.22]	-1.71 [-7.50; 4.08]	-1.71 [-13.00; 9.58]	**CR + HP + BCI**	.	.	.	.	.	.
1.04 [-8.32; 10.40]	2.21 [-4.67; 9.10]	1.68 [-4.89; 8.24]	0.11 [-8.02; 8.23]	-0.57 [-9.45; 8.30]	-0.87 [-8.50; 6.76]	0.75 [-7.49; 8.98]	0.35 [-6.57; 7.26]	-1.14 [-8.69; 6.42]	-1.30 [-6.87; 4.27]	-1.30 [-13.01; 10.41]	0.41 [-7.63; 8.44]	**CR + HP + RE**	.	.	.	.	.
0.73 [-7.81; 9.28]	1.91 [-5.74; 9.55]	1.37 [-5.98; 8.73]	-0.20 [-5.62; 5.22]	-0.88 [-7.97; 6.22]	-1.18 [-9.50; 7.15]	0.44 [-7.22; 8.10]	0.04 [-7.63; 7.71]	-1.44 [-9.69; 6.81]	-1.61 [-9.63; 6.42]	-1.61 [-14.19; 10.98]	0.10 [-8.59; 8.80]	-0.31 [-10.08; 9.46]	**CR + RE**	.	.	.	.
0.24 [-8.05; 8.52]	1.41 [-3.30; 6.13]	0.88 [-3.41; 5.16]	-0.70 [-7.26; 5.87]	-1.37 [-8.94; 6.19]	-1.67 [-6.73; 3.39]	-0.05 [-6.67; 6.57]	-0.46 [-4.94; 4.02]	-1.94 [-7.35; 3.48]	-2.10 [-7.62; 3.41]	-2.10 [-13.26; 9.05]	-0.39 [-6.60; 5.81]	-0.80 [-8.64; 7.04]	-0.50 [-9.01; 8.02]	**CR + RE + BCI**	.	.	-0.45 [-1.08; 0.18]
-6.44 [-22.61; 9.73]	-5.27 [-19.95; 9.41]	-5.80 [-20.33; 8.73]	-7.37 [-22.73; 7.98]	-8.05 [-23.86; 7.76]	-8.35 [-23.39; 6.69]	-6.73 [-22.11; 8.65]	-7.13 [-21.83; 7.56]	-8.62 [-23.62; 6.39]	-8.78 [-23.72; 6.16]	-8.78 [-26.59; 9.03]	-7.07 [-22.32; 8.18]	-7.48 [-23.43; 8.47]	-7.17 [-23.46; 9.11]	-6.68 [-21.83; 8.47]	**HP + RE**	-0.50 [-8.40; 7.40]	.
-6.94 [-21.05; 7.17]	-5.77 [-18.14; 6.61]	-6.30 [-18.50; 5.90]	-7.87 [-21.04; 5.30]	-8.55 [-22.25; 5.15]	-8.85 [-21.65; 3.95]	-7.23 [-20.43; 5.97]	-7.63 [-20.02; 4.75]	-9.12 [-21.87; 3.64]	-9.28 [-21.96; 3.40]	-9.28 [-25.24; 6.68]	-7.57 [-20.62; 5.47]	-7.98 [-21.83; 5.87]	-7.67 [-21.92; 6.57]	-7.18 [-20.11; 5.75]	-0.50 [-8.40; 7.40]	**RE**	.
-0.21 [-8.52; 8.10]	0.96 [-3.80; 5.72]	0.43 [-3.90; 4.76]	-1.14 [-7.74; 5.45]	-1.82 [-9.41; 5.77]	-2.12 [-7.22; 2.98]	-0.50 [-7.15; 6.15]	-0.90 [-5.43; 3.62]	-2.39 [-7.84; 3.07]	-2.55 [-8.10; 3.00]	-2.55 [-13.72; 8.62]	-0.84 [-7.08; 5.39]	-1.25 [-9.11; 6.61]	-0.94 [-9.48; 7.59]	-0.45 [-1.08; 0.18]	6.23 [-8.93; 21.39]	6.73 [-6.21; 19.67]	**RE + BCI**
Network meta-analysis																	

Table S10G: League table - results of pairwise and network meta-analyses of different intervention modalities on fat mass (kg)

Pairwise meta-analysis																			
**AE**	0.35<br>[-5.75; 6.45]	.	-1.43<br>[-5.25; 2.39]	.	5.55<br>[-2.60; 13.69]	.	.	.	.	3.00<br>[-11.14; 17.14]	5.00<br>[-9.28; 19.28]	.	.	.	.	.	.	.	-1.47<br>[-7.76; 4.82]
1.23<br>[-3.55; 6.01]	**AE + RE**	.	-2.89<br>[-7.83; 2.05]	.	.	.	.	4.50<br>[-2.13; 11.13]	5.31<br>[-2.93; 13.55]	.	.	.	.	.	.	.	.	.	-1.82<br>[-7.60; 3.96]
-0.47<br>[-4.22; 3.29]	-1.70<br>[-5.85; 2.45]	**BCI**	**-1.10<br>[-2.08; -0.12]**	.	.	.	.	.	.	.	.	.	.	.	.	.	.	.	.
-1.57<br>[-5.19; 2.06]	-2.80<br>[-6.83; 1.23]	**-1.10<br>[-2.08; -0.12]**	**Con**	3.52<br>[-5.32; 12.35]	.	5.40<br>[-2.54; 13.34]	7.20<br>[-2.65; 17.05]	**7.08<br>[2.77; 11.39]**	8.30<br>[-4.28; 20.88]	5.00<br>[-9.00; 19.00]	7.00<br>[-7.14; 21.14]	.	5.10<br>[-2.79; 12.99]	.	.	6.10<br>[-1.84; 14.04]	1.43<br>[-2.45; 5.31]	2.35<br>[-1.47; 6.17]	3.28<br>[-1.84; 8.39]
3.11<br>[-4.71; 10.93]	1.88<br>[-6.50; 10.26]	3.58<br>[-3.96; 11.12]	4.68<br>[-2.80; 12.16]	**CR**	-0.30<br>[-11.12; 10.52]	.	3.60<br>[-7.65; 14.85]	.	.	.	.	.	.	.	0.30<br>[-9.32; 9.92]	.	.	.	.
4.56<br>[-2.54; 11.65]	3.33<br>[-4.93; 11.59]	5.03<br>[-2.57; 12.63]	6.13<br>[-1.41; 13.66]	1.45<br>[-6.76; 9.65]	**CR + AE**	.	.	.	.	.	.	.	.	.	.	.	.	.	.
4.22<br>[-3.49; 11.93]	2.99<br>[-4.56; 10.55]	4.69<br>[-2.29; 11.66]	5.79<br>[-1.12; 12.70]	1.11<br>[-9.05; 11.27]	-0.34<br>[-10.51; 9.84]	**CR + AE + BCI**	.	1.00<br>[-6.94; 8.94]	1.15<br>[-15.41; 17.71]	.	.	.	.	.	.	0.70<br>[-8.07; 9.47]	.	.	.
5.98<br>[-4.27; 16.23]	4.75<br>[-5.74; 15.24]	6.44<br>[-3.32; 16.21]	7.54<br>[-2.17; 17.26]	2.86<br>[-7.84; 13.57]	1.42<br>[-10.27; 13.11]	1.75<br>[-10.16; 13.67]	**CR + AE + RE**	.	.	.	.	.	.	.	.	.	.	.	.
**5.57<br>[0.40; 10.74]**	4.34<br>[-0.35; 9.03]	**6.04<br>[1.98; 10.10]**	**7.14<br>[3.20; 11.08]**	2.46<br>[-5.96; 10.88]	1.01<br>[-7.41; 9.43]	1.35<br>[-5.57; 8.27]	-0.41<br>[-10.88; 10.07]	**CR + AE + RE + BCI**	0.24<br>[-8.49; 8.96]	.	.	.	.	.	.	-0.30<br>[-8.24; 7.64]	.	.	.
5.49<br>[-2.14; 13.12]	4.26<br>[-2.64; 11.15]	5.96<br>[-1.08; 13.00]	**7.06<br>[0.09; 14.03]**	2.38<br>[-7.79; 12.55]	0.93<br>[-9.21; 11.07]	1.27<br>[-7.40; 9.93]	-0.49<br>[-12.43; 11.46]	-0.08<br>[-7.13; 6.97]	**CR + BCI**	.	.	.	1.60<br>[-15.51; 18.71]	.	.	.	.	.	.
3.22<br>[-9.04; 15.48]	1.99<br>[-10.78; 14.76]	3.69<br>[-8.60; 15.98]	4.79<br>[-7.46; 17.04]	0.11<br>[-14.11; 14.33]	-1.34<br>[-15.39; 12.71]	-1.00<br>[-15.04; 13.04]	-2.76<br>[-18.35; 12.84]	-2.35<br>[-15.18; 10.48]	-2.27<br>[-16.31; 11.77]	**CR + HP**	2.00<br>[-12.00; 16.00]	.	.	-2.60<br>[-12.89; 7.69]	.	.	.	.	.
5.22<br>[-7.20; 17.65]	3.99<br>[-8.94; 16.92]	5.69<br>[-6.77; 18.14]	6.79<br>[-5.63; 19.20]	2.11<br>[-12.25; 16.47]	0.66<br>[-13.53; 14.85]	1.00<br>[-13.18; 15.18]	-0.76<br>[-16.48; 14.97]	-0.35<br>[-13.34; 12.64]	-0.27<br>[-14.45; 13.91]	2.00<br>[-12.00; 16.00]	**CR + HP + AE**	-0.90<br>[-7.04; 5.24]	.	.	.	.	.	.	.
4.32<br>[-9.54; 18.18]	3.09<br>[-11.22; 17.40]	4.79<br>[-9.10; 18.67]	5.89<br>[-7.96; 19.74]	1.21<br>[-14.41; 16.83]	-0.24<br>[-15.70; 15.22]	0.10<br>[-15.36; 15.55]	-1.66<br>[-18.54; 15.23]	-1.25<br>[-15.62; 13.12]	-1.17<br>[-16.62; 14.28]	1.10<br>[-14.19; 16.39]	-0.90<br>[-7.04; 5.24]	**CR + HP + AE + RE**	.	.	.	.	.	.	.
4.16<br>[-3.93; 12.24]	2.93<br>[-5.20; 11.05]	4.62<br>[-2.71; 11.96]	5.72<br>[-1.55; 12.99]	1.04<br>[-9.38; 11.46]	-0.40<br>[-10.85; 10.05]	-0.07<br>[-9.91; 9.77]	-1.82<br>[-13.95; 10.31]	-1.41<br>[-9.53; 6.70]	-1.33<br>[-10.52; 7.85]	0.94<br>[-13.30; 15.17]	-1.06<br>[-15.44; 13.31]	-0.16<br>[-15.80; 15.47]	**CR + HP + BCI**	.	.	.	.	.	.
0.62<br>[-15.39; 16.63]	-0.61<br>[-17.01; 15.79]	1.09<br>[-14.94; 17.12]	2.19<br>[-13.81; 18.19]	-2.49<br>[-20.04; 15.06]	-3.94<br>[-21.35; 13.47]	-3.60<br>[-21.01; 13.80]	-5.36<br>[-24.04; 13.33]	-4.95<br>[-21.40; 11.50]	-4.87<br>[-22.27; 12.53]	-2.60<br>[-12.89; 7.69]	-4.60<br>[-21.97; 12.77]	-3.70<br>[-22.13; 14.73]	-3.54<br>[-21.10; 14.03]	**CR + HP + RE**	.	.	.	.	.
3.41<br>[-8.99; 15.81]	2.18<br>[-10.58; 14.94]	3.88<br>[-8.35; 16.10]	4.98<br>[-7.21; 17.17]	0.30<br>[-9.32; 9.92]	-1.15<br>[-13.79; 11.50]	-0.81<br>[-14.80; 13.18]	-2.56<br>[-16.96; 11.83]	-2.16<br>[-14.94; 10.63]	-2.08<br>[-16.08; 11.92]	0.19<br>[-16.98; 17.36]	-1.81<br>[-19.09; 15.48]	-0.91<br>[-19.25; 17.44]	-0.74<br>[-14.93; 13.44]	2.79<br>[-17.22; 22.81]	**CR + RE**	.	.	.	.
4.91<br>[-3.23; 13.04]	3.68<br>[-4.39; 11.74]	5.37<br>[-2.05; 12.80]	6.47<br>[-0.89; 13.83]	1.79<br>[-8.68; 12.27]	0.35<br>[-10.15; 10.84]	0.68<br>[-7.86; 9.22]	-1.07<br>[-13.26; 11.11]	-0.66<br>[-8.03; 6.70]	-0.58<br>[-10.18; 9.02]	1.69<br>[-12.59; 15.96]	-0.31<br>[-14.73; 14.10]	0.59<br>[-15.08; 16.25]	0.75<br>[-9.51; 11.00]	4.29<br>[-13.31; 21.88]	1.49<br>[-12.73; 15.72]	**CR + RE + BCI**	.	.	.
-0.13<br>[-5.44; 5.17]	-1.36<br>[-6.95; 4.23]	0.33<br>[-3.67; 4.33]	1.43<br>[-2.44; 5.31]	-3.25<br>[-11.67; 5.18]	-4.69<br>[-13.17; 3.78]	-4.36<br>[-12.28; 3.56]	-6.11<br>[-16.57; 4.35]	**-5.70<br>[-11.23; -0.18]**	-5.62<br>[-13.60; 2.35]	-3.35<br>[-16.21; 9.50]	-5.35<br>[-18.36; 7.65]	-4.45<br>[-18.84; 9.93]	-4.29<br>[-12.53; 3.95]	-0.75<br>[-17.22; 15.71]	-3.55<br>[-16.33; 9.24]	-5.04<br>[-13.36; 3.28]	**HP**	0.92<br>[-4.32; 6.16]	.
0.82<br>[-4.34; 5.99]	-0.41<br>[-5.85; 5.03]	1.29<br>[-2.56; 5.15]	2.39<br>[-1.34; 6.12]	-2.29<br>[-10.64; 6.06]	-3.73<br>[-12.13; 4.66]	-3.40<br>[-11.24; 4.44]	-5.15<br>[-15.56; 5.25]	-4.75<br>[-10.16; 0.67]	-4.67<br>[-12.55; 3.22]	-2.40<br>[-15.20; 10.40]	-4.40<br>[-17.35; 8.56]	-3.50<br>[-17.83; 10.84]	-3.33<br>[-11.50; 4.84]	0.20<br>[-16.22; 16.62]	-2.59<br>[-15.33; 10.15]	-4.08<br>[-12.33; 4.16]	0.96<br>[-4.22; 6.14]	**HP + RE**	-0.70<br>[-16.99; 15.59]
0.88<br>[-4.28; 6.03]	-0.35<br>[-5.48; 4.78]	1.34<br>[-3.31; 6.00]	2.44<br>[-2.11; 7.00]	-2.23<br>[-10.86; 6.39]	-3.68<br>[-12.17; 4.81]	-3.35<br>[-11.48; 4.79]	-5.10<br>[-15.80; 5.60]	-4.69<br>[-10.44; 1.06]	-4.61<br>[-12.59; 3.36]	-2.34<br>[-15.28; 10.59]	-4.34<br>[-17.43; 8.74]	-3.44<br>[-17.90; 11.01]	-3.28<br>[-11.80; 5.24]	0.26<br>[-16.27; 16.78]	-2.53<br>[-15.46; 10.39]	-4.03<br>[-12.58; 4.52]	1.01<br>[-4.96; 6.98]	0.05<br>[-5.65; 5.75]	**RE**
Network meta-analysis																			

Table S10H: League table - results of pairwise and network meta-analyses of different intervention modalities on waist circumference (cm)

**Pairwise meta-analysis**											
**AE**	-0.47<br>[-8.20; 7.26]	.	-4.80<br>[-12.56; 2.96]	.	4.02<br>[-4.59; 12.63]	.	.	.	.	.	-1.90<br>[-9.85; 6.05]
-1.18<br>[-8.18; 5.82]	**AE + RE**	.	-2.60<br>[-7.74; 2.54]	.	.	.	4.20<br>[-22.74; 31.14]	4.40<br>[-20.14; 28.94]	.	.	-1.12<br>[-8.89; 6.65]
-2.94<br>[-9.85; 3.98]	-1.76<br>[-6.98; 3.46]	**BCI**	-0.88<br>[-2.28; 0.51]	.	.	.	.	.	.	.	.
-3.82<br>[-10.60; 2.96]	-2.64<br>[-7.67; 2.39]	-0.88<br>[-2.28; 0.51]	**Con**	2.53<br>[-9.29; 14.35]	.	5.90<br>[-10.20; 22.00]	8.72<br>[-0.93; 18.36]	9.40<br>[-11.27; 30.07]	1.78<br>[-19.03; 22.59]	.	3.67<br>[-3.08; 10.43]
-0.05<br>[-11.68; 11.57]	1.13<br>[-10.30; 12.55]	2.88<br>[-7.71; 13.47]	3.77<br>[-6.73; 14.27]	**CR**	-0.10<br>[-20.06; 19.86]	3.70<br>[-15.55; 22.95]	.	.	.	.	.
3.36<br>[-4.75; 11.48]	4.54<br>[-5.77; 14.86]	6.30<br>[-3.83; 16.43]	7.18<br>[-2.85; 17.21]	3.42<br>[-9.18; 16.01]	**CR + AE**	.	.	.	.	.	.
2.61<br>[-13.86; 19.08]	3.79<br>[-12.21; 19.79]	5.55<br>[-9.76; 20.86]	6.43<br>[-8.82; 21.68]	2.66<br>[-13.72; 19.04]	-0.75<br>[-18.51; 17.01]	**CR + AE + RE**	.	.	.	.	.
4.64<br>[-7.03; 16.32]	5.82<br>[-4.87; 16.51]	7.58<br>[-2.09; 17.25]	8.46<br>[-1.11; 18.03]	4.70<br>[-9.50; 18.89]	1.28<br>[-12.55; 15.11]	2.03<br>[-15.97; 20.03]	**CR + AE + RE + BCI**	0.20<br>[-26.10; 26.50]	.	.	.
2.26<br>[-15.53; 20.06]	3.44<br>[-13.60; 20.49]	5.20<br>[-11.45; 21.85]	6.08<br>[-10.50; 22.67]	2.32<br>[-17.29; 21.92]	-1.10<br>[-20.39; 18.19]	-0.35<br>[-22.87; 22.18]	-2.38<br>[-20.80; 16.05]	**CR + BCI**	3.40<br>[-22.11; 28.91]	.	.
1.04<br>[-17.62; 19.70]	2.22<br>[-15.82; 20.26]	3.98<br>[-13.52; 21.47]	4.86<br>[-12.58; 22.30]	1.09<br>[-19.25; 21.44]	-2.32<br>[-22.40; 17.75]	-1.57<br>[-24.73; 21.59]	-3.60<br>[-23.22; 16.01]	-1.22<br>[-20.18; 17.73]	**CR + HP + BCI**	.	.
-0.24<br>[-25.88; 25.41]	0.94<br>[-24.51; 26.39]	2.70<br>[-22.68; 28.07]	3.58<br>[-21.75; 28.92]	-0.18<br>[-27.48; 27.11]	-3.60<br>[-30.36; 23.16]	-2.85<br>[-32.38; 26.68]	-4.88<br>[-31.94; 22.18]	-2.50<br>[-32.72; 27.72]	-1.28<br>[-32.01; 29.45]	**HP + RE**	-0.80<br>[-25.32; 23.72]
-1.04<br>[-8.55; 6.48]	0.14<br>[-6.67; 6.95]	1.90<br>[-4.63; 8.43]	2.78<br>[-3.60; 9.16]	-0.98<br>[-12.99; 11.02]	-4.40<br>[-15.12; 6.32]	-3.65<br>[-20.11; 12.81]	-5.68<br>[-17.14; 5.78]	-3.30<br>[-20.97; 14.36]	-2.08<br>[-20.60; 16.45]	-0.80<br>[-25.32; 23.72]	**RE**
**Network meta-analysis**											

Table S10I: League table - results of pairwise and network meta-analyses of different intervention modalities on body mass (kg)

Pairwise meta-analysis

**AE**	-0.46 [-5.95; 5.03]	.	-2.33 [-6.59; 1.93]	.	5.33 [-3.80; 14.45]	.	.	.	.	3.00 [-11.14; 17.14]	7.00 [-7.28; 21.28]	.	.	.	.	.	-2.13 [-8.00; 3.74]	.
-0.47 [-4.79; 3.84]	**AE + RE**	.	-1.86 [-5.06; 1.33]	.	.	.	.	8.10 [-2.16; 18.36]	8.79 [-3.44; 21.02]	.	.	.	.	.	.	.	-1.27 [-6.82; 4.28]	.
-0.87 [-4.90; 3.16]	-0.39 [-3.67; 2.88]	**BCI**	**-1.30 [-2.44; -0.16]**	.	.	.	.	.	.	.	.	.	.	.	.	.	.	.
-2.17 [-6.04; 1.69]	-1.70 [-4.77; 1.37]	**-1.30 [-2.44; -0.16]**	**Con**	3.02 [-1.75; 7.80]	.	8.10 [-1.58; 17.78]	7.40 [-7.56; 22.36]	**8.52 [3.48; 13.56]**	5.38 [-1.75; 12.51]	6.00 [-8.00; 20.00]	10.00 [-4.14; 24.14]	.	**6.39 [5.14; 7.64]**	.	.	7.60 [-1.17; 16.37]	1.72 [-3.38; 6.82]	.
0.77 [-5.26; 6.80]	1.24 [-4.36; 6.85]	1.64 [-3.20; 6.47]	2.94 [-1.76; 7.64]	**CR**	6.16 [-19.08; 31.40]	.	4.00 [-11.28; 19.28]	.	.	.	.	.	.	.	0.20 [-11.17; 11.57]	.	.	.
4.63 [-2.72; 11.99]	5.11 [-2.85; 13.07]	5.50 [-2.20; 13.20]	6.80 [-0.81; 14.42]	3.86 [-4.86; 12.58]	**CR + AE**	3.60 [-6.44; 13.64]	.	.	.	.	.	.	.	.	.	.	.	.
7.03 [-0.74; 14.79]	7.50 [-0.28; 15.29]	**7.90 [0.48; 15.31]**	**9.20 [1.88; 16.52]**	6.26 [-2.35; 14.86]	2.40 [-5.48; 10.27]	**CR + AE + BCI**	.	-0.50 [-10.18; 9.18]	1.48 [-31.04; 34.00]	.	.	.	.	.	.	-0.50 [-10.18; 9.18]	.	.
5.02 [-9.64; 19.67]	5.49 [-8.98; 19.96]	5.88 [-8.30; 20.07]	7.19 [-6.95; 21.33]	4.25 [-9.95; 18.44]	0.38 [-15.62; 16.39]	-2.01 [-17.91; 13.89]	**CR + AE + RE**	.	.	.	.	.	.	.	.	.	.	.
**6.50 [0.51; 12.49]**	**6.97 [1.53; 12.41]**	**7.37 [2.45; 12.28]**	**8.67 [3.89; 13.45]**	5.73 [-0.96; 12.41]	1.87 [-6.68; 10.41]	-0.53 [-8.31; 7.25]	1.48 [-13.44; 16.41]	**CR + AE + RE + BCI**	0.17 [-12.23; 12.56]	.	.	.	.	.	.	0.00 [-8.77; 8.77]	.	.
3.89 [-3.39; 11.16]	4.36 [-2.42; 11.14]	4.75 [-1.61; 11.12]	6.06 [-0.20; 12.32]	3.12 [-4.71; 10.94]	-0.75 [-10.44; 8.95]	-3.14 [-12.46; 6.18]	-1.13 [-16.59; 14.33]	-2.61 [-10.10; 4.87]	**CR + BCI**	.	.	.	-1.20 [-20.12; 17.72]	.	.	.	.	.
3.42 [-8.86; 15.70]	3.90 [-8.64; 16.43]	4.29 [-8.03; 16.61]	5.59 [-6.68; 17.86]	2.65 [-10.48; 15.78]	-1.21 [-15.33; 12.91]	-3.61 [-17.75; 10.54]	-1.59 [-20.31; 17.13]	-3.08 [-16.21; 10.06]	-0.46 [-14.22; 13.29]	**CR + HP**	4.00 [-10.00; 18.00]	.	.	3.00 [-8.74; 14.74]	.	.	.	.
7.42 [-5.02; 19.87]	7.90 [-4.80; 20.59]	8.29 [-4.19; 20.77]	9.59 [-2.84; 22.03]	6.65 [-6.63; 19.93]	2.79 [-11.47; 17.05]	0.39 [-13.89; 14.68]	2.41 [-16.42; 21.23]	0.92 [-12.36; 14.21]	3.54 [-10.36; 17.43]	4.00 [-10.00; 18.00]	**CR + HP + AE**	-1.00 [-12.16; 10.16]	.	.	.	.	.	.
6.42 [-10.29; 23.14]	6.90 [-10.01; 23.80]	7.29 [-9.45; 24.03]	8.59 [-8.11; 25.30]	5.65 [-11.69; 23.00]	1.79 [-16.31; 19.90]	-0.61 [-18.73; 17.52]	1.41 [-20.48; 23.29]	-0.08 [-17.42; 17.27]	2.54 [-15.29; 20.36]	3.00 [-14.90; 20.90]	-1.00 [-12.16; 10.16]	**CR + HP + AE+ RE**	.	.	.	.	.	.
**4.21 [0.15; 8.27]**	**4.68 [1.37; 8.00]**	**5.08 [3.39; 6.77]**	**6.38 [5.13; 7.63]**	3.44 [-1.42; 8.30]	-0.42 [-8.14; 7.29]	-2.82 [-10.25; 4.61]	-0.81 [-15.00; 13.39]	-2.29 [-7.23; 2.65]	0.32 [-6.03; 6.68]	0.79 [-11.55; 13.12]	-3.21 [-15.71; 9.28]	-2.21 [-18.96; 14.54]	**CR + HP + BCI**	.	.	.	.	.
6.42 [-10.57; 23.41]	6.90 [-10.28; 24.07]	7.29 [-9.73; 24.31]	8.59 [-8.39; 25.58]	5.65 [-11.96; 23.26]	1.79 [-16.57; 20.15]	-0.61 [-18.99; 17.78]	1.41 [-20.69; 23.50]	-0.08 [-17.69; 17.54]	2.54 [-15.55; 20.62]	3.00 [-8.74; 14.74]	-1.00 [-19.27; 17.27]	0.00 [-21.41; 21.41]	2.21 [-14.82; 19.24]	**CR + HP + RE**	.	.	.	.
0.97 [-11.90; 13.84]	1.44 [-11.23; 14.12]	1.84 [-10.52; 14.20]	3.14 [-9.16; 15.45]	0.20 [-11.17; 11.57]	-3.66 [-17.99; 10.67]	-6.06 [-20.32; 8.20]	-4.05 [-22.24; 14.15]	-5.53 [-18.72; 7.66]	-2.92 [-16.72; 10.89]	-2.45 [-19.82; 14.92]	-6.45 [-23.94; 11.03]	-5.45 [-26.19; 15.29]	-3.24 [-15.61; 9.13]	-5.45 [-26.42; 15.51]	**CR + RE**	.	.	.
6.11 [-2.44; 14.65]	6.58 [-1.71; 14.87]	6.98 [-0.91; 14.86]	**8.28 [0.48; 16.09]**	5.34 [-3.74; 14.42]	1.48 [-8.68; 11.63]	-0.92 [-9.92; 8.08]	1.09 [-15.05; 17.24]	-0.39 [-8.31; 7.53]	2.22 [-7.59; 12.04]	2.69 [-11.81; 17.18]	-1.31 [-15.95; 13.32]	-0.31 [-18.71; 18.09]	1.90 [-6.01; 9.80]	-0.31 [-18.97; 18.34]	5.14 [-9.42; 19.69]	**CR + RE + BCI**	.	**-2.80 [-4.78; -0.83]**
-1.10 [-6.33; 4.13]	-0.62 [-5.55; 4.30]	-0.23 [-5.06; 4.60]	1.07 [-3.62; 5.76]	-1.87 [-8.49; 4.76]	-5.73 [-14.32; 2.86]	-8.13 [-16.66; 0.40]	-6.11 [-21.01; 8.78]	**-7.60 [-14.20; -1.00]**	-4.98 [-12.74; 2.77]	-4.52 [-17.48; 8.43]	-8.52 [-21.63; 4.59]	-7.52 [-24.74; 9.69]	**-5.31 [-10.16; -0.45]**	-7.52 [-25.01; 9.96]	-2.07 [-15.23; 11.09]	-7.21 [-16.25; 1.83]	**RE**	.
3.31 [-5.46; 12.08]	3.78 [-4.74; 12.30]	4.17 [-3.96; 12.31]	5.48 [-2.57; 13.53]	2.54 [-6.76; 11.83]	-1.33 [-11.67; 9.02]	-3.72 [-12.93; 5.49]	-1.71 [-17.97; 14.56]	-3.19 [-11.35; 4.97]	-0.58 [-10.59; 9.43]	-0.12 [-14.74; 14.51]	-4.12 [-18.88; 10.65]	-3.12 [-21.62; 15.39]	-0.90 [-9.05; 7.24]	-3.12 [-21.87; 15.64]	2.34 [-12.35; 17.02]	**-2.80 [-4.78; -0.83]**	4.41 [-4.85; 13.66]	**RE + BCI**

Network meta-analysis

Table S10J: League table - results of pairwise and network meta-analyses of different intervention modalities on bone mineral density of the total hip (BMD TH)

Pairwise meta-analysis					
**AE + RE**	0.02 [-0.03; 0.07]	.	0.02 [-0.03; 0.08]	0.04 [-0.01; 0.09]	.
0.02 [-0.03; 0.06]	**Con**	0.03 [-0.04; 0.10]	0.01 [-0.02; 0.05]	0.02 [-0.04; 0.08]	0.01 [-0.06; 0.08]
0.05 [-0.03; 0.12]	0.03 [-0.04; 0.09]	**CR + AE + BCI**	-0.01 [-0.08; 0.06]	.	-0.02 [-0.09; 0.05]
0.03 [-0.02; 0.08]	0.01 [-0.02; 0.05]	-0.02 [-0.08; 0.05]	**CR + AE + RE + BCI**	0.02 [-0.05; 0.08]	-0.01 [-0.08; 0.06]
0.04 [-0.01; 0.09]	0.02 [-0.03; 0.08]	-0.01 [-0.08; 0.07]	0.01 [-0.05; 0.07]	**CR + BCI**	.
0.02 [-0.05; 0.10]	0.01 [-0.06; 0.07]	-0.02 [-0.09; 0.05]	-0.01 [-0.07; 0.06]	-0.02 [-0.10; 0.06]	**CR + RE + BCI**
**Network meta-analysis**					

Table S11 A-H: Descriptive results on the effects of lifestyle interventions on physical function (A), body composition and anthropometry measures related to muscle mass (B), body fat (C), and bone (D), quality of life (E), psychological outcomes (F), health events (G), and social participation (H)

To view table S11, please download file Supporting information 2 on OSF: https://osf.io/ayhq3?view_only=6e987edd80924f35b689f2b50ee3749b

Table S12: Adverse events reported in included randomized controlled trials (n=54)

Author, Year	Adverse Event
Abdelbasset 2020	Not reported
Amamou 2017	Not reported
Andonian 2024	No serious adverse events for study completers; other adverse events reported, but unrelated to intervention
Ard 2017	Related to intervention: definitely 3.9% (mostly musculoskeletal injuries), possibly 25.0%; 71.1%
Balachandran 2014	Events such as pain
Beavers 2015	Adverse Events not related to study protocol
Beavers 2019	Adverse Events not related to study protocol
Bowen 2022	Not reported
Brennan 2020	Not reported
Brubaker 2023	No study-related serious adverse events
Cai 2019	Not reported
Coppock 2023	Not reported
Davidson 2009	Not reported
Elsayed 2022	No adverse events related to the intervention in either group during the study.
Fanning 2020	Not reported
Fanning 2021	Not reported
Ferhi 2023	Not reported
Furlano 2023	Not reported
Gregori 2025	Exercise-related adverse events in the intensive lifestyle group included falls, neck pain, back pain, foot pain, shoulder pain, and leg pain
Haywood 2017	One participant fell during exercise class and one had chest pain. Constipation, dizziness, gout annd hzpoglzcaemia were reported as diet related adverse events.
Horie 2016	Not reported
Ismail 2023	Not reported
Kallings 2009	Not reported
Kemmler 2016	Adverse events reported but none related to intervention.
Kemmler 2017	No adverse effects of WB-EMS or protein supplementation were reported.
Kim 2018	Not reported
Kim 2019	Not reported
Kim 2020	Not reported
Kitzman 2016	Five adverse events possibly related to the intervention: hypoglycemia, ankle pain and swelling later diagnosed as partial tendon tear, stress foot fracture, an episode of unusual shortness of breath during exercise. No study-related serious adverse events.
Kritchevsky 2017	Not reported
Koh 2025	Not reported
Lambert 2008	Not reported
Lee 2021	No adverse event was observed after the intervention in any of the study participants.
Maillard 2016	Not reported
Manini 2010	Not reported

Mesinovic 2025	There were no exercise-related injuries reported in the AT group. In the HiRIT group, there was one exercise-related injury (lower back strain), and this participant returned to the gym after a 10-day break and completed the study without any ongoing issues. Two other injuries reported in the HiRIT group were possibly exercise-related, specifically a minor muscle tear a participant noticed during recreational swimming and lower back strain noticed by another participant when they were moving houses; both participants continued exercising with one achieving 100% adherence despite the muscle tear. Both groups had participants who had lesions (kidney and pancreas), or previously unknown biochemical abnormalities (iron deficiency), detected during baseline appointments. In the AT group, one participant reported supplement-related GI discomfort (unable to tolerate calcium and vitamin D tablets; the participant switched to a different brand with the same dose and did not report any issues), another participant had gastroenteritis, and one participant had a coronary event 1 month after completing the study, which was deemed to be a SAE unlikely to be related to the intervention.
Miller 2010	Not reported
Muscariello 2016	Not reported
Nabuco 2019	Not reported
Nicklas 2014	Not reported
Nicklas 2019	There were eight minor intervention-related adverse events (all musculoskeletal complaints).
Normandin 2019	Five participants reported adverse events from wearing the vest (all back pain or soreness).
O'Leary 2007	Not reported
Park 2017	Not reported
Park 2019	Not reported
Park 2020	Therefore, participants did not have any overt events associated with the exercise intervention, but the most adverse events reported were musculoskeletal problems such as the delayed onset of muscle soreness (DOMS) and joint pain in the inital period against the exercise intervention.
Park 2024	Not reported
Polo-Ferrero 2025	Notably, no serious adverse events were reported
Porter Starr 2016	No adverse events related to the protocol and no serious adverse events occurred. One Protein participant was disqualified due to GFR drop down and one control participant who had remained qualified throughout the trial had a GFR of 42 at 6 months.
Prieto 2014	Not reported
Prieto 2015	Not reported
Rezaeipour 2021	No events such as dropouts and adverse reactions.
Rosenberg 2020	There were no serious adverse events. Mild skin irritation was the only adverse event (two related to activPAL and three from the Jawbone UP band).
Rosenberg 2024	87 and 57 adverse events in intervention and control group. 35 and 19 musculoskeletal events, and 9 and 1 skin disorders in intervention and control group. None of the six serious adverse events in each study arm was study related.
Rosety 2015	No sport-related injuries were reported in the experimental group.

Serra-Prat 2021	Not reported
Shah 2009	None of the subjects experienced any clinical adverse effects related to the diet-induced weight loss or exercise intervention.
Solomon 2009	Not reported
Solomon 2010	No adverse events were associated with our testing procedures or the intvervention.
Son 2024a	Not reported
Son 2024b	Not reported
Stillman 2018	Not reported
Travieso 2025	Not reported
Villareal 2006	None of the participants experienced any adverse effects in serum electrolyte concentrations or in renal or liver function test results. One participant fell during an exercise session, which resulted in a small forehead laceration.
Villareal 2011	One participant fell during testing of physical function, and the fall resulted in an ankle fracture. Also, adverse events included a small number of exercise-associated musculoskeletal injuries.
Villareal 2017	Exercise-related adverse events included falling, shoulder pain, back pain, atrial fibrillation, shoulder pain, knee pain, shoulder injury, knee pain, hip pain in all intervention groups.
Vincent 2014	The number of patients experiencing any adverse event was 8/22 enrolled in the TOTRX and 3/20 in the LEXT compared with 0 in the con group. The proportion of patients experiencing a severe AE, as judged by the investigators, was similar between the training groups (9% in TOTRX and 10% in LEXT), and these were anticipated AE and not related to the study (worsening of back pain that required medical intervention).
Wang 2023	Not reported
West 2011	Not reported
Yassine 2009	Not reported
Yin 2023	No adverse events
Zhou 2021	Not reported

Table S13: References of reports for which requested information was requested
To view table S13, please download file Supporting information 2 on OSF:
https://osf.io/ayhq3?view_only=6e987edd80924f35b689f2b50ee3749b

Excluded References

1 A low-carbohydrate diet reduces obesity and may improve dyslipidaemia compared with a low-fat diet. *Evidence-based healthcare and public health*. 2004; 8: 370-72.

2 Effects of dietary intervention and aquatic physical exercises for obese older adult women with knee osteoarthritis on body weight and food consumption: a controlled clinical trial. *Clinical Nutrition ESPEN*. 2020; 40: 684-.

3 Abed H, Cheng Y, Baumert M, Antic N, Abhayaratna W, Wittert G*, et al.* Non-invasive characterisation of electrical remodeling in an interventional weight loss program: implications for obesity and atrial arrhythmias. *Heart lung and circulation*. 2011; 20: S103-.

4 Abou-Raya A, Abou-Raya S, Helmii M. Effect of exercise and dietary weight loss on symptoms and systemic inflammation in obese adults with psoriatic arthritis: randomized controlled trial. *Annals of the Rheumatic Diseases*. 2014; 73.

5 Abou-Raya S, Abou-Raya A, Abd Kareem A. Effects of physical activity on inflammation, skeletal muscle strength/function (sarcopenia) and fat infiltration (sarcopenic obesity) in older adults with knee osteoarthritis: a randomized controlled trial. *Annals of the Rheumatic Diseases*. 2013; 72.

6 Ang D, Beavers D, Messier SP. Does reduction in mechanical knee joint loading explain the beneficial effects of weight loss in overweight or obese patients with knee osteoarthritis? *Arthritis and rheumatology*. 2018; 70: 466-67.

7 Arija Val V, Martinez FV, Pedret Llaberia R, Vinuesa Fernandez A, Jovani D, Reche A*, et al.* Effectiveness of a physical activity program on cardiovascular disease risk in adult with overweight and obesity: the “Pas-a-Pas” community intervention trial. *Obesity Facts*. 2018; 11: 38-.

8 Armamento-Villareal R, Napoli N, Shah K, Aguirre L, Hilton T, Sinacore D*, et al.* Changes in thigh muscle volume predict changes in femoral BMD in sarcopenic elderly obese adults undergoing lifestyle therapy. *Journal of bone and mineral research*. 2012; 27.

9 Armamento-Villareal R, Sadler C, Chode S, Napoli N, Qualls C, Shah K*, et al.* Sclerostin mediates the skeletal effects of weight loss and exercise therapy in obese older adults. *Journal of bone and mineral research*. 2011; 26.

10 Atar-Greenfield H. The effects of type and frequency of exercise on maintenance of body composition and weight loss outcomes in obese women. 2005; 65: 6086-86.

11 Aubertin-Leheudre M. Preserving body & muscle composition in obese-osteopenic older women: highintensity interval training a potential intervention? *Osteoporosis international*. 2020; 31: S106-.

12 Balducci S, D'Errico V, Haxhi J, Sacchetti M, Orlando G, Cardelli P*, et al.* Effect of a behavioral intervention for adoption and maintenance of a physically active lifestyle: the Italian diabetes and exercise study 2 (IDES-2). *Diabetes*. 2016; 65: A188-.

13 Barone BB. The effect of exercise on endothelial function in adults with and without type 2 diabetes. 2011; 71: 6023-23.

14 Bartlett SJ. The psychological effects of exercise on dieting obese women. 1996; 57: 2929-29.

15 Beavers D, Martin S, Marsh A, Lyles M, Lenchik L, Nicklas B. Effect of exercise modality during weight loss on bone mineral density in overweight and obese, older adults. *Journal of bone and mineral research*. 2015; 30.

16 Beavers K, Walkup M, Ambrosius W, Kritchevsky S, Lenchik L, Shapses S*, et al.* Effect of exercise modality during weight loss on hip and spine bone mineral density in older adults with obesity. *Journal of bone and mineral research*. 2017; 32: S101-.

17 Blüher M, Rudich A, Klöting N, Golan R, Rubin E, Schwarzfuchs D*, et al.* Two major patterns of dynamics in adipokine serum concentrations over 2 years of dietary weight-loss intervention. *Diabetologia*. 2010; 53: S367-.
18 Bonardi JMT, Lima LG, Bertani RF, Moriguti JC, Ferriolli E, Lima NKC. The blood pressure fall after aerobic exercise is associated to the loss of fat mass in hypertensive older individuals with overweight. *Journal of Clinical Hypertension*. 2012; 14.
19 Bray G, Wagenknecht L, Walkup M, Heshka S, Hill J, Hubbard V*, et al.* Effect of age on loss and regain of lean body mass in type 2 diabetes: four-year results from the look ahead dxa substudy. *Obesity Facts*. 2012; 5: 48-.
20 Burke L, Jancey J, Howat P, Lee A, Kerr D, Shilton T*, et al.* Physical activity results of a home-based physical activity and nutrition program for seniors (PANS). *Journal of science and medicine in sport*. 2012; 15: S47-S48.
21 Celli A, Blevin D, Barnouin Y, Colleluori G, Villareal RC, Villareal DT. Intensive lifestyle intervention in older adults with diabetes improves glycemic control, body composition, physical function, and bone quality. *Endocrine reviews*. 2016; 37.
22 Chintapalli S, Jay M. Comparison of acceptance-based versus standard behavioral treatment for obesity in adults. *Journal of clinical outcomes management*. 2013; 20: 491-93.
23 Cubb T, Acevedo-calado MJ, Celli A, Colleluori G, Phadnis UD, Pietropaolo M*, et al.* Lifestyle intervention in senior diabetics (LISD) subanalysis- autoimmune profile in the overweight/obese elderly. *Diabetes*. 2018; 67: LB76-.
24 Dash C, Hicks J, Lu J, Mills M, Adams-Campbell L. Sarcopenic obesity and metabolic syndrome in the fierce aerobic exercise trial in black women. *Obesity (Silver Spring, Md)*. 2020; 28: 155-.
25 Distefano G, Standley R, Carnero EA, Coen PM, Vega RB, Goodpaster BH. Exercise is required to improve mitochondrial respiratory capacity in skeletal muscle of older obese subjects during calorie restriction-induced weight loss to improve insulin sensitivity. *Diabetes*. 2018; 67: A505-.
26 Dubyak PJ. The effect of group size on weight loss outcomes in a lifestyle intervention for obesity. 2017; 77.
27 Evans EM, Larson RD, Johnson CL, Guest DD, Das BM, Ward CL*, et al.* Effects of weight loss or exercise on muscle quality in obese older women. *FASEB Journal*. 2012; 26.
28 Fanning J, Brooks AK, Hsieh KL, Kershner K, Furlipa J, Nicklas BJ*, et al.* Building on Lessons Learned in a Mobile Intervention to Reduce Pain and Improve Health (MORPH): Protocol for the MORPH-II Trial. *JMIR Research Protocols*. 2021; 10: e29013.
29 Fanning J, Brooks AK, Irby MB, N'Dah KW, Rejeski WJ. Associations Between Patterns of Daily Stepping Behavior, Health-Related Quality of Life, and Pain Symptoms Among Older Adults with Chronic Pain: A Secondary Analysis of Two Randomized Controlled Trials. *Clinical Interventions In Aging*. 2024; 19: 459-70.
30 Fanning J, Brooks AK, Robison JT, Irby MB, Ford S, N'Dah K*, et al.* Associations between patterns of physical activity, pain intensity, and interference among older adults with chronic pain: a secondary analysis of two randomized controlled trials. *Frontiers in Aging*. 2023; 4: 1216942.
31 Fougere B. Physical performance in obeseosteopenic elderly women: highintensity interval training more efficient than continuous aerobic training? *Osteoporosis international*. 2020; 31: S106-.
32 Freitas-Simoes TM, Cofan M, Serra-Mir M, Roth I, Calvo C, Valls-Pedret C*, et al.* Walnut supplementation for 2 years in older individuals reduces ldl-cholesterol without

adverse effects on adiposity. a sub-study of the walnuts and healthy aging (WAHA) study. *Annals of Nutrition & Metabolism*. 2017; 71: 988-89.
33 Ganguli R, Brar JS. Behavioural intervention for weight loss in schizophrenia: an RCT with active controls. *Indian journal of psychiatry*. 2011; 53: S44.
34 Gibbs JC, Carvalho LP, Marcangeli V, El Hajj Boutros G, Dulac MC, Aubertin-Leheudre M. Effect of high-intensity interval training on peripheral quantitative computed tomography measures of quadriceps muscle and adipose tissue properties in obese, osteopenic older women. *Journal of bone and mineral research*. 2019; 34: 156-.
35 Gorin AA. A controlled trial of cognitive-behavioral therapy with and without spousal involvement for binge eating disorder. 2001; 61: 4983-83.
36 Haji M, Memelink RG, Brandt BW, Buijs MJ, Pasman WJ, De Vogel-Van den Bosch J*, et al.* Increased species richness in the oral microbiome by a 13-week protein and combined lifestyle intervention in older adults with obesity and type 2 diabetes. *Clinical Nutrition ESPEN*. 2021; 46: S667-.
37 Han L, Zhang T, Sun D, Ma W, Wang T, Heianza Y*, et al.* Temporal relationship between changes in body weight and insulin resistance and its impact on blood pressure in response to 2-year weight-loss diet interventions: the pounds. *Circulation*. 2017; 135.
38 Harris LM, Mounsey A, Nashelsky J. Clinical Inquiries: Can mobile technology improve weight loss in overweight and obese patients? *Journal of Family Practice*. 2017; 66: 111-13.
39 Jakubowicz D, Froy O, Tsameret S, Wainstein J, Raz I, Menaged M*, et al.* High energy breakfast diet is an effective strategy for weight loss and reduction of the total daily insulin dose in type 2 diabetes. *Endocrine reviews*. 2018; 39.
40 Jakubowicz D, Froy O, Tsameret S, Wainstein J, Raz I, Menaged M*, et al.* Three meals diet with high energy breakfast is an effective strategy for weight loss, reduction of glucose variability and of total daily insulin dose in type 2 diabetes. *Diabetologia*. 2018; 61: S99-.
41 Jefferson ME. The effects of resistance training and weight loss on arterial stiffness in older adults. *Arteriosclerosis, thrombosis, and vascular biology*. 2014; 34.
42 Jepma P, Jorstad HT, Snaterse M, Ter Riet G, Kragten HJ, Lachman S*, et al.* Successful lifestyle modification in older successful lifestyle modification in older patients with coronary artery disease: results from the RESPONSE-2 Trial. *European geriatric medicine*. 2019; 10: S157-.
43 Kahleova H, Belinova L, Malinska H, Kazdova L, Dezortova M, Hajek M*, et al.* Meal frequency and type 2 diabetes. *Obesity facts*. 2015; 8: 12.
44 Krinick GB. Evaluation of weight resistance training as a component of exercise in the behavioral treatment of obesity. 2000; 61: 1066-66.
45 Kritchevsky S, Manini T, Blair S, Newman A, Botoseneanu A, Lovato L*, et al.* The effect of physical activity on mobility disability in obese, abdominally obese and non-obese older adults: the lifestyle interventions and independence for elders (life) study. *Journal of the american geriatrics society*. 2015; 63: S1.
46 Lin TY, Hung SC. Effects of oral nutritional supplement in hemodialysis patients with normal weight obesity: a pilot feasibility randomized controlled trial. *Nephrology Dialysis Transplantation*. 2019; 34: a488-.
47 Loeser R, Beavers D, Bay-Jensen AC, Karsdal M, Nicklas B, Hunter D*, et al.* Effects of exercise and weight loss on interstitial matrix turnover and tissue inflammation biomarkers in adults with knee osteoarthritis: the intensive diet and exercise for arthritis trial (idea). *Osteoarthritis and cartilage*. 2016; 24: S32-S33.
48 Martin CK, Bray GA, Ryan DH, Carey VJ, Champagne CM, Williamson DA*, et al.* A high carbohydrate/average protein diet is less efficacious at promoting weight loss among

women compared to men: subgroup analysis from POUNDS lost. *Obesity (Silver Spring, Md)*. 2010; 18: S94-.
49 McDonald SR, Porter Starr KN, Mauceri L, Orenduff M, Granville E, Ocampo C*, et al.* Meal-based enhancement of protein quality and quantity during weight loss in obese older adults with mobility limitations: rationale and design for the MEASUR-UP trial. *Contemporary Clinical Trials*. 2015; 40: 112-23.
50 Memelink R, Verreijen A, Engberink M, Weijs P. Higher than 1.2 G/KG/D protein intake is associated with increased fat mass loss during weight loss in obese older adults. *Clinical nutrition*. 2014; 33: S110.
51 Memelink RG, Bongers A, Pasman WJ, van Rijmenam MJ, Wopereis S, Verlaan G*, et al.* Preservation of Lean Mass Upon Combined Lifestyle Intervention in Older Adults with Obesity and Type 2 Diabetes during 6-Months Follow-up after Rct (Probe Study). *Clinical nutrition (Edinburgh, Scotland)*. 2019; 38: S16-S17.
52 Miketinas D, Bray G, Sacks F, Champagne C. Fiber intake, dietary energy density, and adherence to diet assignment are positively associated with weight-loss in free-living adults consuming calorie-restricted diets at 6-month follow-up: the POUNDS LOST study. *FASEB Journal*. 2017; 31.
53 Miranda ER, Varady K, Haus JM. Weight loss via alternate day fasting increases circulating endogenous secretory RAGE and is associated with markers of adipocyte health. *FASEB Journal*. 2017; 31.
54 Mooney K, Kirk B, Khaiyat O, Amirabdollahian F. Effects of protein supplementation alone and in combination with exercise on cardiometabolic health markers in older adults. *Proceedings of the Nutrition Society*. 2019; 78.
55 Munro JF. Second-order change through brief therapy among obese clients of a university-based weight management program. 2002; 63: 1267-67.
56 Noirez P, Dulac M, Carvalho LP, El Haj Boutros G, Marcangeli V, Gaudreau P*, et al.* Effect of high-intensity interval or moderateintensity continuous training on body compostion and physical performance in obeseosteopenic elderly women. *Osteoporosis International*. 2018; 29: S150-.
57 Palle H. Implementation of educational and motivational text messaging of diet and lifestyle modifications to reduce body mass index (BMI) in African American adults with BMI>30 in 3 months. 2021; 82.
58 Perri MG, Wadden TA, VanItallie TB. Improving maintenance of weight loss following treatment by diet and lifestyle modification. *Treatment of the seriously obese patient*. 1992: 456-77.
59 Potts SA. Putting weight in context: Acceptance and Commitment Therapy (ACT) guided self-help for weight self-stigma. 2018; 79.
60 Pruss C, Ross R, Adams M. Long term exercise significantly decreases von Willebrand factor antigen levels in abdominally obese adults. *Journal of thrombosis and haemostasis*. 2015; 13: 745.
61 Ribot I, Lecha M, Penalva A, Cots I, Sansano A, Martinez J*, et al.* Signing a therapeutic commitment contract supports weight loss. *Clinical nutrition*. 2013; 32: S203.
62 Rosenberg DE, Greenwood-Hickman MA, Zhou J, Cook AJ, Mettert KD, Cooper J*, et al.* Protocol for a randomized controlled trial of sitting reduction to improve cardiometabolic health in older adults. *Contemporary Clinical Trials*. 2021; 111: 106593.
63 Rozati M, Barnett J, Wu D, Handelman G, Dallal G, Saltzman E*, et al.* Cardiometabolic and immunological impacts of extra virgin olive oil consumption in overweight and obese older adults: a randomized controlled trial. *FASEB Journal*. 2015; 29.

64 Salas-Salvado J. Weight loss using a healthy diet and exercise promotion to prevent cardiovascular disease: the predimed-plus trial. *Annals of Nutrition & Metabolism*. 2017; 71: 24-.
65 Sayer RD, Speaker KJ, Pan Z, Peters JC, Wyatt HJ, Hill JO. Body fat changes in the beef WISE study: beef's role in weight improvement, satisfaction, and energy. *FASEB Journal*. 2017; 31.
66 Serra-Prat M, Terradellas M, Arus M, Salietti A, Sanchez L, Ramirez S*, et al.* Effect of a nutritional and physical exercise intervention on changes in body composition and risk of frailty in elderly obese subjects. *European geriatric medicine*. 2019; 10: S21-.
67 Shah K, Parimi N, Chode S, Sinacore D, Napoli N, Armamento-Villarea R*, et al.* Effects of weight loss, exercise, or combined on bone mineral density and markers of bone turnover in frail obese older adults. *Journal of bone and mineral research*. 2010; 25: S76-.
68 Shai I. The effect of low-carb, mediterranean and low-fat diets on renal function; a 2-year dietary intervention randomized controlled trial (direct). *Obesity Facts*. 2012; 5: 19-.
69 Starr KNP, Orenduff MC, McDonald SR, Pieper CF, Mulder HC, Maloney K*, et al.* Influence of daily and per meal protein intakes on function and body composition in frail, obese older adults undergoing weight reduction. *FASEB Journal*. 2016; 30.
70 Stensvold DSEAWUSSA. Aerobic interval training versus strength training as a treatment for the metabolic syndrome. *European heart journal Conference: european society of cardiology, ESC congress 2009 Barcelona spain*. 2009; 30: 466.
71 Sulaj A, Zemva J, Zech U, Woehning A, Brune M, Rudofsky G*, et al.* The effect of lifestyle intervention in severely obese subjects on the soluble form of Activated Leukocyte Cell Adhesion Molecule (ALCAM). *Diabetologie und stoffwechsel*. 2016; 11.
72 Swift DL, Sarzynski MA, McGee J, Barefoot S, Brophy PM, Nevels T*, et al.* Effects of exercise training and increasing non-exercise physical activity on lipoprotein subclass and size: results from the i-can study. *Circulation*. 2018; 137.
73 Talavera GA, Castaneda S, Lopez-Gurrola M, Alvarez-Malo AR, Hernandez J, Sears D*, et al.* Arriba Por la Vida Estudio: a Randomized Trial Promoting Standing Behavior to Reduce Sitting Time among Postmenopausal Latinas. *Circulation*. 2020; 142.
74 Uusitupa M. Healthy nordic diet and metabolic syndrome-the sysdiet study. *Annals of nutrition and metabolism Conference: 12th european nutrition conference, FENS 2015 Berlin germany Conference start: 20151020 Conference end: 20151023 Conference publication: (varpagings)*. 2015; 67: 52.
75 Villalobos Martinez F, Pedret R, Vinuesa A, Jovani D, Reche A, Peralta L*, et al.* Physical activity, anthropometric measurements and selfesteem in adult obese women primary health-care users: the “pas-a-Pas” community intervention trial. *Obesity Facts*. 2019; 12: 228-.
76 Villareal R. A randomized, open-label, intervention study to evaluate the effect of diet composition on weight gain in obese, type 2 diabetes patients receiving intensive insulin therapy. *College of health sciences*. 2008; Ph.D.: 364.
77 Vincent HK, Conrad B, George SZ, Hurley RW, Montero C, Seay A*, et al.* Resistance exercise in obese older adults with chronic back pain: preliminary results. *PM and r*. 2012; 4: S251-.
78 Vincent HK, Seay A, Hurley RW, Vincent KR. Multifidus muscle cross-sectional area after resistance exercise is related to pain reduction but not improvement in ambulatory activity in obese older adults with chronic back pain. *PM and r*. 2014; 6: S183-S84.
79 Walton NR, Loeser RF, Beavers D, Nicklas BJ, Lyles M, Messier SP. The effects of intensive diet and exercise on bone density in older adults with knee osteoarthritis: the

intensive diet and exercise for arthritis (IDEA) trial. *Arthritis and rheumatism*. 2012; 64: S1070-S71.
80 Waters D. Lifestyle interventions for frail obese older adults. *Aging medicine and healthcare*. 2019; 10: 3-.
81 Whitehouse CR. From Hospital to Home: Aiding in Patient Transitions through Education for Obese Older Adults with Type 2 Diabetes Mellitus. *From Hospital to Home: Aiding in Patient Transitions Through Education for Obese Older Adults with Type 2 Diabetes Mellitus*. 2016: 1-1.
82 Willmer T, Oosthuizen A, Dias S, Goedecke J, Pheiffer C. Impact of Exercise Intervention on DNA Methylation of FKBP5 in Obese, Insulin Resistant South African Women. *Metabolism: Clinical and Experimental*. 2021; 116.
83 A diet based on multiple functional concepts improves cardiometabolic risk parameters in healthy subjects. *Nutrition & Metabolism*. 2012; 9: 29-39.
84 Impact of a motivational resistance-training programme on adherence and body composition in the elderly. *Scientific Reports*. 2018; 8: 1370-.
85 DXA-assessed changes in body composition in obese women following two different weight loss programs. *Nutrition (Burbank, Los Angeles County, Calif)*. 2018; 46.
86 Weight Loss Experiences of African American, Hispanic, and Non-Hispanic White Men and Women with Type 2 Diabetes: the Look AHEAD Trial. *Obesity (Silver Spring, Md)*. 2019; 27: 1275-84.
87 Effects of exercise intervention on serum leptin, cellular immune function, body mass index and exercise ability in patients with obesity. *Acta Medica Mediterranea*. 2020; 36: 2257-61.
88 Abadi FH, Sankaravel M, Zainuddin FF, Elumalai G, Razli AI. The effect of aquatic exercise program on low-back pain disability in obese women. *Journal of Exercise Rehabilitation*. 2019; 15: 855-60.
89 Abdelaal AAM, Mohamad MA. Obesity indices and haemodynamic response to exercise in obese diabetic hypertensive patients: Randomized controlled trial. *Obesity Research & Clinical Practice*. 2015; 9: 475-86.
90 Abed HS, Nelson AJ, Richardson JD, Worthley SG, Vincent A, Wittert GA*, et al.* Impact of weight reduction on pericardial adipose tissue and cardiac structure in patients with atrial fibrillation. *American Heart Journal*. 2015; 169: 655-62.e52.
91 Abed HS, Wittert GA, Leong DP, Shirazi MG, Bahrami B, Middeldorp ME*, et al.* Effect of weight reduction and cardiometabolic risk factor management on symptom burden and severity in patients with atrial fibrillation: a randomized clinical trial. *Jama*. 2013; 310: 2050-60.
92 Abraham N, Lyden K, Boucher R, Wei G, Gonce V, Carle J*, et al.* An intervention to decrease sedentary behavior in older adults: A secondary analysis of a randomized controlled trial. *Obesity Science & Practice*. 2023; 9: 529-37.
93 Abramson EE. Dismantling the behavioral treatment of obesity. *International Journal of Eating Disorders*. 1985; 4: 107-11.
94 Ackermann RT, Sandy LG, Beauregard T, Coblitz M, Norton KL, Vojta D. A randomized comparative effectiveness trial of using cable television to deliver diabetes prevention programming. *Obesity*. 2014; 22: 1601-07.
95 Adachi Y. The effect of behavioral treatment of obesity and correlates of weight loss in treatment and at 2-year follow-up. *Japanese Journal of Behavior Therapy*. 1989; 15: 36-55.

96 Adachi Y, Sato C, Yamatsu K, Ito S, Adachi K, Yamagami T. A randomized controlled trial on the long-term effects of a 1-month behavioral weight control program assisted by computer tailored advice. *Behaviour research and therapy*. 2007; 45: 459-70.
97 Adams BD, Arem H, Hubal MJ, Cartmel B, Li F, Harrigan M*, et al.* Exercise and weight loss interventions and miRNA expression in women with breast cancer. *Breast Cancer Research & Treatment*. 2018; 170: 55-67.
98 Adamsson V, Reumark A, Marklund M, Larsson A, Riserus U. Role of a prudent breakfast in improving cardiometabolic risk factors in subjects with hypercholesterolemia: a randomized controlled trial. *Clinical Nutrition*. 2015; 34: 20-26.
99 Ades PA, Savage PD, Lischke S, Toth MJ, Harvey-Berino J, Bunn JY*, et al.* The effect of weight loss and exercise training on flow-mediated dilatation in coronary heart disease: a randomized trial. *Chest*. 2011; 140: 1420-27.
100 Ades PA, Savage PD, Toth MJ, Harvey-Berino J, Schneider DJ, Bunn JY*, et al.* High-calorie-expenditure exercise: a new approach to cardiac rehabilitation for overweight coronary patients. *Circulation*. 2009; 119: 2671-78.
101 Adler E, Dhruva A, Moran PJ, Daubenmier J, Acree M, Epel ES*, et al.* Impact of a mindfulness-based weight-loss intervention on sleep quality among adults with obesity: Data from the SHINE randomized controlled trial. *The Journal of Alternative and Complementary Medicine*. 2017; 23: 188-95.
102 Agras WS, Berkowitz RI, Arnow BA, Telch CF, Marnell M, Henderson J*, et al.* Maintenance following a very-low-calorie diet. *Journal of consulting and clinical psychology*. 1996; 64: 610-13.
103 Agras WS, Telch CF, Arnow B, Eldredge K, Detzer MJ, Henderson J*, et al.* Does interpersonal therapy help patients with binge eating disorder who fail to respond to cognitive-behavioral therapy? *Journal of Consulting & Clinical Psychology*. 1995; 63: 356-60.
104 Ahn A, Choi J. A one-way text messaging intervention for obesity. *Journal of Telemedicine & Telecare*. 2016; 22: 148-52.
105 Ahrens RA, Hower M, Best AM. Effects of weight reduction interventions by community pharmacists. *Journal of the American Pharmacists Association : JAPhA*. 2003; 43: 583-89.
106 Aktas MF, Mahler A, Hamm M, Perger G, Simon F, Westenhofer J*, et al.* Lifestyle interventions in Muslim patients with metabolic syndrome-a feasibility study. *European Journal of Clinical Nutrition*. 2019; 73: 805-08.
107 Al Abdrabalnabi A, Rajaram S, Bitok E, Oda K, Beeson WL, Kaur A*, et al.* Effects of Supplementing the Usual Diet with a Daily Dose of Walnuts for Two Years on Metabolic Syndrome and Its Components in an Elderly Cohort. *Nutrients*. 2020; 12: 451.
108 Alam S, Stolinski M, Pentecost C, Boroujerdi MA, Jones RH, Sonksen PH*, et al.* The effect of a six-month exercise program on very low-density lipoprotein apolipoprotein B secretion in type 2 diabetes. *Journal of Clinical Endocrinology & Metabolism*. 2004; 89: 688-94.
109 Albu JB, Heilbronn LK, Kelley DE, Smith SR, Azuma K, Berk ES*, et al.* Metabolic changes following a 1-year diet and exercise intervention in patients with type 2 diabetes. *Diabetes*. 2010; 59: 627-33.
110 Alcántara-Aragón V, Rodrigo-Cano S, Lupianez-Barbero A, Martinez MJ, Martinez C, Tapia J*, et al.* Web Support for Weight-Loss Interventions: pREDIRCAM2 Clinical Trial Baseline Characteristics and Preliminary Results. *Diabetes Technology & Therapeutics*. 2018; 20: 380-85.

111 Alencar M, Johnson K, Gray V, Mullur R, Gutierrez E, Dionico P. Telehealth-based health coaching increases m-health device adherence and rate of weight loss in obese participants. *Telemedicine and e-Health*. 2020; 26: 365-68.
112 Alencar MK, Johnson K, Mullur R, Gray V, Gutierrez E, Korosteleva O. The efficacy of a telemedicine-based weight loss program with video conference health coaching support. *Journal of telemedicine and telecare*. 2019; 25: 151-57.
113 Alfaris N, Wadden TA, Sarwer DB, Diwald L, Volger S, Hong P*, et al.* Effects of a 2-year behavioral weight loss intervention on sleep and mood in obese individuals treated in primary care practice. *Obesity*. 2015; 23: 558-64.
114 Alfawaz HA, Wani K, Alnaami AM, Al-Saleh Y, Aljohani NJ, Al-Attas OS*, et al.* Effects of Different Dietary and Lifestyle Modification Therapies on Metabolic Syndrome in Prediabetic Arab Patients: a 12-Month Longitudinal Study. *Nutrients*. 2018; 10.
115 Alfonsson S, Parling T, Ghaderi A. Group behavioral activation for patients with severe obesity and binge eating disorder: a randomized controlled trial. *Behavior modification*. 2015; 39: 270-94.
116 Alghamdi RQ. A randomized controlled trial of a 12-week intensive lifestyle intervention program at a primary care obesity clinic for adults in western Saudi Arabia. *Saudi Medical Journal*. 2017; 38: 837-45.
117 Alharbi M, Gallagher R, Kirkness A, Sibbritt D, Tofler G. Long-term outcomes from Healthy Eating and Exercise Lifestyle Program for overweight people with heart disease and diabetes. *European Journal of Cardiovascular Nursing*. 2016; 15: 91-99.
118 Aller R, de Luis DA, Izaola O, de la Fuente B, Bachiller R. Effect of a high monounsaturated vs high polyunsaturated fat hypocaloric diets in nonalcoholic fatty liver disease. *European Review for Medical & Pharmacological Sciences*. 2014; 18: 1041-47.
119 Aller R, Izaola O, Primo D, de Luis DA. The effect of single-nucleotide polymorphisms at the ADIPOQ gene locus rs1501299 on metabolic parameters after 9 mo of a high-protein/low-carbohydrate versus a standard hypocaloric diet. *Nutrition*. 2019; 65: 44-49.
120 Aller R, Primo D, Izaola O, de Luis DA. Common polymorphism in the cannabinoid receptor gene type 2 (CB2R) rs3123554 are associated with metabolic changes after two different hypocaloric diets with different dietary fatty profiles. *Clinical Nutrition*. 2019; 38: 2632-38.
121 Allet L, Müller-Pinget S, Punt I, Edelsten C, Ballif A, Golay A*, et al.* Dance therapy combined with patient education improves quality of life of persons with obesity: a pilot feasibility study for a randomised controlled trial. *Obesity Research & Clinical Practice*. 2017; 11: 79-87.
122 Allicock M, Ko L, van der Sterren E, Valle CG, Campbell MK, Carr C. Pilot weight control intervention among US veterans to promote diets high in fruits and vegetables. *Preventive Medicine*. 2010; 51: 279-81.
123 Allison K, Jones S, Hinman RS, Pardo J, Li P, DeSilva A*, et al.* Alternative models to support weight loss in chronic musculoskeletal conditions: effectiveness of a physiotherapist-delivered intensive diet programme for knee osteoarthritis, the POWER randomised controlled trial. *British Journal of Sports Medicine*. 2024; 58: 538-47.
124 Alpaugh M, Pope L, Trubek A, Skelly J, Harvey J. Cooking as a health behavior: examining the role of cooking classes in a weight loss intervention. *Nutrients*. 2020; 12: 1-13.
125 Alvarez C, Ramírez R, Flores M, Zúñiga C, Celis-Morales CA. Effect of sprint interval training and resistance exercise on metabolic markers in overweight women. *Revista Medica de Chile*. 2012; 140: 1289-96.

126 Alvarez-Perez J, Sanchez-Villegas A, Diaz-Benitez EM, Ruano-Rodriguez C, Corella D, Martinez-Gonzalez MA*, et al.* Influence of a Mediterranean Dietary Pattern on Body Fat Distribution: Results of the PREDIMED-Canarias Intervention Randomized Trial. *Journal of the American College of Nutrition*. 2016; 35: 568-80.
127 Alves JG, Gale CR, Mutrie N, Correia JB, Batty GD. A 6-month exercise intervention among inactive and overweight favela-residing women in Brazil: the Caranguejo Exercise Trial. *American Journal of Public Health*. 2009; 99: 76-80.
128 Amati F, Dube JJ, Shay C, Goodpaster BH. Separate and combined effects of exercise training and weight loss on exercise efficiency and substrate oxidation. *Journal of Applied Physiology*. 2008; 105: 825-31.
129 Amato S, Colajanni E, Averna MR, Barbagallo CM, Lo Cascio ML, Traina G*, et al.* Diet and psychological therapy in a group of severely obese patients. *Minerva Endocrinologica*. 1990; 15: 219-21.
130 Ambeba EJ, Lei Y, Sereika SM, Styn MA, Acharya SD, Sevick MA*, et al.* The Use of mHealth to Deliver Tailored Messages Reduces Reported Energy and Fat Intake. *Journal of Cardiovascular Nursing*. 2015; 30: 35-43.
131 Ambrosini GL, Solis-Trapala I, Ahern AL, Fuller NR, Holzapfel C, Hauner H*, et al.* Greater improvements in diet quality among overweight participants following a group-based commercial weight loss programme than those receiving support to lose weight in primary care. *Nutrition Journal*. 2018; 17: 64.
132 Amer B, Clausen MR, Bertram HC, Bohl M, Nebel C, Zheng H*, et al.* Consumption of Whey in Combination with Dairy Medium-Chain Fatty Acids (MCFAs) may Reduce Lipid Storage due to Urinary Loss of Tricarboxylic Acid Cycle Intermediates and Increased Rates of MCFAs Oxidation. *Molecular Nutrition & Food Research*. 2017; 61.
133 Andersen SV, Sandby K, Hjorth MF, Korndal SK, Ritz C, Sjodin A*, et al.* No effects on appetite or body weight in weight-reduced individuals of foods containing components previously shown to reduce appetite - Results from the SATIN (Satiety Innovation) study. *Obesity medicine*. 2020; 17.
134 Anderson JW, Fuller J, Patterson K, Blair R, Tabor A. Soy compared to casein meal replacement shakes with energy-restricted diets for obese women: randomized controlled trial. *Metabolism: Clinical & Experimental*. 2007; 56: 280-88.
135 Anderson JW, Hoie LH. Weight loss and lipid changes with low-energy diets: comparator study of milk-based versus soy-based liquid meal replacement interventions. *Journal of the American College of Nutrition*. 2005; 24: 210-16.
136 Annesi JJ. Relations of changes in self-regulatory efficacy and physical self-concept with improvements in body satisfaction in obese women initiating exercise with cognitive-behavioral support. *Body Image*. 2010; 7: 356-59.
137 Annesi JJ. Moderation of fatigue and stress in the carry-over of self-regulation and self-efficacy for exercise to self-regulation and self-efficacy for managed eating. *Psychology*. 2011; 2: 694-99.
138 Annesi JJ. Effects of exercise self-regulation on subsequent eating self-regulation: Implications for depletion vs improvement based on behavioral treatment foci. *European Review of Applied Psychology / Revue Européenne de Psychologie Appliquée*. 2019; 69: 1-7.
139 Annesi JJ, Gorjala S. Association of reduction in waist circumference with normalization of mood in obese women initiating exercise supported by the Coach Approach protocol. *Southern Medical Journal*. 2010; 103: 517-21.

140 Annesi JJ, Johnson PH. Theory-based psychosocial factors that discriminate between weight-loss success and failure over 6 months in women with morbid obesity receiving behavioral treatments. *Eating and weight disorders*. 2015; 20: 223-32.
141 Annesi JJ, Johnson PH, Tennant GA, Porter KJ, McEwen KL. Weight Loss and the Prevention of Weight Regain: evaluation of a Treatment Model of Exercise Self-Regulation Generalizing to Controlled Eating. *Permanente Journal*. 2016; 20: 15-146.
142 Annesi JJ, Mareno N. Temporal aspects of psychosocial predictors of increased fruit and vegetable intake in adults with severe obesity: mediation by physical activity. *Journal of Community Health*. 2014; 39: 454-63.
143 Annesi JJ, Mareno N. Improvement in emotional eating associated with an enhanced body image in obese women: Mediation by weight-management treatments' effects on self-efficacy to resist emotional cues to eating. *Journal of Advanced Nursing*. 2015; 71: 2923-35.
144 Annesi JJ, Tennant GA. Exercise program-induced mood improvement and improved eating in severely obese adults. *International Quarterly of Community Health Education*. 2012; 33: 391-402.
145 Anton SD, Gallagher J, Carey VJ, Laranjo N, Cheng J, Champagne CM*, et al.* Diet type and changes in food cravings following weight loss: findings from the POUNDS LOST Trial. *Eating & Weight Disorders: EWD*. 2012; 17: e101-08.
146 Anton SD, LeBlanc E, Allen HR, Karabetian C, Sacks F, Bray G*, et al.* Use of a computerized tracking system to monitor and provide feedback on dietary goals for calorie-restricted diets: the POUNDS LOST study. *Journal of Diabetes Science & Technology*. 2012; 6: 1216-25.
147 Aoike DT, Baria F, Kamimura MA, Ammirati A, Cuppari L. Home-based versus center-based aerobic exercise on cardiopulmonary performance, physical function, quality of life and quality of sleep of overweight patients with chronic kidney disease. *Clinical & Experimental Nephrology*. 2018; 22: 87-98.
148 Arciero PJ, Baur D, Connelly S, Ormsbee MJ. Timed-daily ingestion of whey protein and exercise training reduces visceral adipose tissue mass and improves insulin resistance: the PRISE study. *Journal of applied physiology (Bethesda, Md : 1985)*. 2014; 117: 1-10.
149 Arciero PJ, Edmonds R, He F, Ward E, Gumpricht E, Mohr A*, et al.* Protein-Pacing Caloric-Restriction Enhances Body Composition Similarly in Obese Men and Women during Weight Loss and Sustains Efficacy during Long-Term Weight Maintenance. *Nutrients*. 2016; 8: 30.
150 Arciero PJ, Edmonds RC, Bunsawat K, Gentile CL, Ketcham C, Darin C*, et al.* Protein-Pacing from Food or Supplementation Improves Physical Performance in Overweight Men and Women: the PRISE 2 Study. *Nutrients*. 2016; 8.
151 Arciero PJ, Gentile CL, Pressman R, Everett M, Ormsbee MJ, Martin J*, et al.* Moderate protein intake improves total and regional body composition and insulin sensitivity in overweight adults. *Metabolism: clinical and experimental*. 2008; 57: 757-65.
152 Arciero PJ, Ives SJ, Norton C, Escudero D, Minicucci O, O'Brien G*, et al.* Protein-Pacing and Multi-Component Exercise Training Improves Physical Performance Outcomes in Exercise-Trained Women: the PRISE 3 Study. *Nutrients*. 2016; 8.
153 Aree-Ue S, Saraboon Y, Belza B. Long-Term Adherence and Effectiveness of a Multicomponent Intervention for Community-Dwelling Overweight Thai Older Adults with Knee Osteoarthritis. *Journal of Gerontological Nursing*. 2017; 43: 40-48.
154 Armendáriz-Anguiano AL, Jiménez-Cruz A, Bacardí-Gascón M, Hurtado-Ayala L. Effect of a low glycemic load on body composition and Homeostasis Model Assessment (HOMA) in overweight and obese subjects. *Nutricion Hospitalaria*. 2011; 26: 170-75.

155 Armenta Guirado BI, Díaz Zavala RG, Valencia Juillerat ME, Quizán Plata T. Obesity Management in the Primary Care Setting by an Intensive Lifestyle Intervention. *Nutricion Hospitalaria*. 2015; 32: 1526-34.
156 Armitage CJ, Alganem S, Norman P. Randomized controlled trial of a volitional help sheet to encourage weight loss in the Middle East. *Prevention science*. 2017; 18: 976-83.
157 Ashby WA, Wilson GT. Behavior therapy for obesity: booster sessions and long-term maintenance of weight loss. *Behaviour Research & Therapy*. 1977; 15: 451-63.
158 Astbury NM, Aveyard P, Nickless A, Hood K, Corfield K, Lowe R*, et al.* Doctor Referral of Overweight People to Low Energy total diet replacement Treatment (DROPLET): pragmatic randomised controlled trial. *BMJ (Clinical research ed)*. 2018; 362: k3760.
159 Astorga Verdugo S, Borges Silva F, González Silva S, Martínez Araya A, Rojas Cabezas G, Romero Bello E. Efectividad de la movilidad cervicodorsal con característica socializadora y lúdica con relación al riesgo de caídas en adultos mayores con anteposición de cabeza y cuello: un ensayo clínico aleatorizado. *Fisioterapia*. 2022; 44: 15-21.
160 Astrup AV, Nielsen CM, Greve T, Kreutzer M, Pedersen U, Soensen LB*, et al.* A randomized comparison of a course in gourmet cookery versus behavior modification in the treatment of severe obesity. *Ugeskrift for Laeger*. 2003; 165: 4958-61.
161 Attux C, Martini LC, Elkis H, Tamai S, Freirias A, Camargo M*, et al.* A 6-month randomized controlled trial to test the efficacy of a lifestyle intervention for weight gain management in schizophrenia. *BMC Psychiatry*. 2013; 13: 60.
162 Azadbakht L, Haghighatdoost F, Karimi G, Esmaillzadeh A. Effect of consuming salad and yogurt as preload on body weight management and cardiovascular risk factors: a randomized clinical trial. *International journal of food sciences and nutrition*. 2013; 64: 392-99.
163 Azadbakht L, Mirmiran P, Esmaillzadeh A, Azizi F. Better dietary adherence and weight maintenance achieved by a long-term moderate-fat diet. *British Journal of Nutrition*. 2007; 97: 399-404.
164 Azamian Jazi A, Moradi Sarteshnizi E, Fathi M, Azamian Jazi Z. Elastic band resistance training increases adropin and ameliorates some cardiometabolic risk factors in elderly women: A quasi-experimental study. *BMC Sports Science, Medicine and Rehabilitation*. 2022; 14: 178.
165 Azar KMJ, Aurora M, Wang EJ, Muzaffar A, Pressman A, Palaniappan LP. Virtual small groups for weight management: An innovative delivery mechanism for evidence-based lifestyle interventions among obese men. *Translational Behavioral Medicine*. 2015; 5: 37-44.
166 Azhar G, Raza S, Pangle A, Coleman K, Dawson A, Schrader A*, et al.* Potential Beneficial Effects of Dietary Protein Supplementation and Exercise on Functional Capacity in a Pilot Study of Individuals with Heart Failure with Preserved Ejection Fraction. *Gerontology & Geriatric Medicine*. 2020; 6: 2333721420982808.
167 Babatunde OA, Arp Adams S, Truman S, Sercy EM, Murphy AE, Khan SM*, et al.* The impact of a randomized dietary and physical activity intervention on chronic inflammation among obese African-American women. *Women & Health*. 2020; 60: 792-805.
168 Bacchi E, Negri C, Zanolin ME, Milanese C, Faccioli N, Trombetta M*, et al.* Metabolic effects of aerobic training and resistance training in type 2 diabetic subjects: a randomized controlled trial (the RAED2 study). *Diabetes Care*. 2012; 35: 676-82.
169 Baireddy S, Saharan AK, Kathju V, Yatheendrakumar G, Saharan M, Sivajyothi N. The effect of body mass index on functional outcome of patients with knee arthroplasty with and without hip abductor muscle strengthening: six months follow-up of a randomized pilot study. *International Journal of Research in Pharmaceutical Sciences*. 2020; 11: 2365-70.

170 Bajpeyi S, Tanner CJ, Slentz CA, Duscha BD, McCartney JS, Hickner RC*, et al.* Effect of exercise intensity and volume on persistence of insulin sensitivity during training cessation. *Journal of applied physiology (Bethesda, Md : 1985)*. 2009; 106: 1079-85.
171 Bakhtiari A, Yassin Z, Hanachi P, Rahmat A, Ahmad Z, Sajadi P*, et al.* Effects of Soy on Body Composition: A 12-Week Randomized Controlled Trial among Iranian Elderly Women with Metabolic Syndrome. *Iranian Journal of Public Health*. 2012; 41: 9-18.
172 Balducci S, Zanuso S, Cardelli P, Salerno G, Fallucca S, Nicolucci A*, et al.* Supervised exercise training counterbalances the adverse effects of insulin therapy in overweight/obese subjects with type 2 diabetes. *Diabetes Care*. 2012; 35: 39-41.
173 Bales CW, Hawk VH, Granville EO, Rose SB, Shields T, Bateman L*, et al.* Aerobic and resistance training effects on energy intake: the STRRIDE-AT/RT study. *Medicine and science in sports and exercise*. 2012; 44: 2033-39.
174 Balkestein EJ, van Aggel-Leijssen DP, van Baak MA, Struijker-Boudier HA, Van Bortel LM. The effect of weight loss with or without exercise training on large artery compliance in healthy obese men. *Journal of Hypertension*. 1999; 17: 1831-35.
175 Ballesteros-Pomar MD, Calleja-Fernandez AR, Vidal-Casariego A, Urioste-Fondo AM, Cano-Rodriguez I. Effectiveness of energy-restricted diets with different protein:carbohydrate ratios: the relationship to insulin sensitivity. *Public Health Nutrition*. 2010; 13: 2119-26.
176 Bancks MP, Lovato J, Balasubramanyam A, Coday M, Johnson KC, Munshi M*, et al.* Association of Type 2 Diabetes Subgroups With Cognitive Status Without Modification From Lifestyle Intervention. *Journal of Clinical Endocrinology & Metabolism*. 2023; 108: e334-e42.
177 Banitalebi E, Banitalebi E, Ghahfarokhi MM, Rahimi M, Laher I, Davison K. Resistance Band Exercise: An Effective Strategy to Reverse Cardiometabolic Disorders in Women With Osteosarcopenic Obesity. *Journal of Aging & Physical Activity*. 2023; 31: 633-41.
178 Banitalebi E, Faramarzi M, Ghahfarokhi MM, SavariNikoo F, Soltani N, Bahramzadeh A. Osteosarcopenic obesity markers following elastic band resistance training: A randomized controlled trial. *Experimental Gerontology*. 2020; 135: 110884.
179 Banitalebi E, Ghahfarrokhi MM, Dehghan M. Effect of 12-weeks elastic band resistance training on MyomiRs and osteoporosis markers in elderly women with Osteosarcopenic obesity: a randomized controlled trial. *BMC Geriatrics*. 2021; 21: 433.
180 Bao DQ, Mori TA, Burke V, Puddey IB, Beilin LJ. Effects of dietary fish and weight reduction on ambulatory blood pressure in overweight hypertensives. *Hypertension*. 1998; 32: 710-17.
181 Barazzoni R, Gortan Cappellari G, Zanetti M, Klaus KA, Semolic A, Johnson ML*, et al.* Higher unacylated ghrelin and insulin sensitivity following dietary restriction and weight loss in obese humans. *Clinical Nutrition*. 2021; 40: 638-44.
182 Barbour JA, Howe PR, Buckley JD, Bryan J, Coates AM. Effect of 12 Weeks High Oleic Peanut Consumption on Cardio-Metabolic Risk Factors and Body Composition. *Nutrients*. 2015; 7: 7381-98.
183 Barnes RD, Barber JA. Preliminary examination of metabolic syndrome response to motivational interviewing for weight loss as compared to an attentional control and usual care in primary care for individuals with and without binge-eating disorder. *Eating Behaviors*. 2017; 26: 108-13.
184 Barnes RD, Ivezaj V, Martino S, Pittman BP, Grilo CM. Back to Basics? No Weight Loss from Motivational Interviewing Compared to Nutrition Psychoeducation at One-Year Follow-Up. *Obesity*. 2017; 25: 2074-78.

185 Barnes RD, Ivezaj V, Martino S, Pittman BP, Paris M, Grilo CM. Examining motivational interviewing plus nutrition psychoeducation for weight loss in primary care. *Journal of Psychosomatic Research*. 2018; 104: 101-07.

186 Barnes RD, Ivezaj V, Pittman BP, Grilo CM. Early weight loss predicts weight loss treatment response regardless of binge-eating disorder status and pretreatment weight change. *International Journal of Eating Disorders*. 2018; 51: 558-64.

187 Barnes RD, White MA, Martino S, Grilo CM. A randomized controlled trial comparing scalable weight loss treatments in primary care. *Obesity (Silver Spring, Md)*. 2014; 22: 2508-16.

188 Barone BB, Wang NY, Bacher AC, Stewart KJ. Decreased exercise blood pressure in older adults after exercise training: contributions of increased fitness and decreased fatness. *British Journal of Sports Medicine*. 2009; 43: 52-56.

189 Barratt R, Frost G, Millward DJ, Truby H. A randomised controlled trial investigating the effect of an intensive lifestyle intervention v. standard care in adults with type 2 diabetes immediately after initiating insulin therapy. *British Journal of Nutrition*. 2008; 99: 1025-31.

190 Bartels SJ, Pratt SI, Aschbrenner KA, Barre LK, Jue K, Wolfe RS*, et al.* Clinically significant improved fitness and weight loss among overweight persons with serious mental illness. *Psychiatric Services*. 2013; 64: 729-36.

191 Bartfield JK, Stevens VJ, Jerome GJ, Batch BC, Kennedy BM, Vollmer WM*, et al.* Behavioral transitions and weight change patterns within the PREMIER trial. *Obesity*. 2011; 19: 1609-15.

192 Basler H-D, Brinkmeier U, Buser K, Haehn K-D, Mölders-Kober R. Psychological group treatment of obese essential hypertensives by lay therapists in rural general practice settings. *Journal of Psychosomatic Research*. 1985; 29: 383-91.

193 Baum JG, Clark HB, Sandler J. Preventing relapse in obesity through posttreatment maintenance systems: comparing the relative efficacy of two levels of therapist support. *Journal of Behavioral Medicine*. 1991; 14: 287-302.

194 Bazzano LA, Hu T, Reynolds K, Yao L, Bunol C, Liu Y*, et al.* Effects of low-carbohydrate and low-fat diets: a randomized trial. *Annals of Internal Medicine*. 2014; 161: 309-18.

195 Beavers DP, Beavers KM, Loeser RF, Walton NR, Lyles MF, Nicklas BJ*, et al.* The independent and combined effects of intensive weight loss and exercise training on bone mineral density in overweight and obese older adults with osteoarthritis. *Osteoarthritis & Cartilage*. 2014; 22: 726-33.

196 Beavers KM, Ambrosius WT, Nicklas BJ, Rejeski WJ. Independent and combined effects of physical activity and weight loss on inflammatory biomarkers in overweight and obese older adults. *Journal of the American Geriatrics Society*. 2013; 61: 1089-94.

197 Beavers KM, Ambrosius WT, Rejeski WJ, Burdette JH, Walkup MP, Sheedy JL*, et al.* Effect of Exercise Type During Intentional Weight Loss on Body Composition in Older Adults with Obesity. *Obesity*. 2017; 25: 1823-29.

198 Beavers KM, Avery AE, Shankaran M, Evans WJ, Lynch SD, Dwyer C*, et al.* Application of the D₃ -creatine muscle mass assessment tool to a geriatric weight loss trial: A pilot study. *Journal of Cachexia, Sarcopenia and Muscle*. 2023; 14: 2350-58.

199 Beavers KM, Beavers DP, Martin SB, Marsh AP, Lyles MF, Lenchik L*, et al.* Change in Bone Mineral Density During Weight Loss with Resistance Versus Aerobic Exercise Training in Older Adults. *Journals of Gerontology Series A-Biological Sciences & Medical Sciences*. 2017; 72: 1582-85.

200 Beavers KM, Beavers DP, Nesbit BA, Ambrosius WT, Marsh AP, Nicklas BJ*, et al.* Effect of an 18-month physical activity and weight loss intervention on body composition in overweight and obese older adults. *Obesity*. 2014; 22: 325-31.
201 Beavers KM, Beavers DP, Newman JJ, Anderson AM, Loeser RF, Jr., Nicklas BJ*, et al.* Effects of total and regional fat loss on plasma CRP and IL-6 in overweight and obese, older adults with knee osteoarthritis. *Osteoarthritis & Cartilage*. 2015; 23: 249-56.
202 Beavers KM, Miller ME, Rejeski WJ, Nicklas BJ, Kritchevsky SB. Fat mass loss predicts gain in physical function with intentional weight loss in older adults. *Journals of Gerontology Series A-Biological Sciences & Medical Sciences*. 2013; 68: 80-86.
203 Beavers KM, Walkup MP, Weaver AA, Lenchik L, Kritchevsky SB, Nicklas BJ*, et al.* Effect of Exercise Modality During Weight Loss on Bone Health in Older Adults With Obesity and Cardiovascular Disease or Metabolic Syndrome: A Randomized Controlled Trial. *Journal of Bone & Mineral Research*. 2018; 33: 2140-49.
204 Beebe Nowen E. Effects of the addition of Tai Chi to diet education on the atherogenicity of lipoproteins in obese older women. 2012: 134.
205 Beeken RJ, Leurent B, Vickerstaff V, Wilson R, Croker H, Morris S*, et al.* A brief intervention for weight control based on habit-formation theory delivered through primary care: results from a randomised controlled trial. *International Journal of Obesity*. 2017; 41: 246-54.
206 Befort CA, Donnelly JE, Sullivan DK, Ellerbeck EF, Perri MG. Group versus individual phone-based obesity treatment for rural women. *Eating Behaviors*. 2010; 11: 11-17.
207 Befort CA, Kimler BF, Bantis LE, Phillips TA, Fabian CJ. Effects of Weight Loss and Weight Regain on Circulating Biomarkers in Overweight/Obese Breast Cancer Survivors Enrolled in a Weight Loss Trial in the Rural Midwest. *Cancer Epidemiology, Biomarkers & Prevention*. 2020; 29: 1321-28.
208 Befort CA, Nollen N, Ellerbeck EF, Sullivan DK, Thomas JL, Ahluwalia JS. Motivational interviewing fails to improve outcomes of a behavioral weight loss program for obese African American women: a pilot randomized trial. *Journal of Behavioral Medicine*. 2008; 31: 367-77.
209 Behm-Morawitz E, Lewallen J, Choi G. A second chance at health: How a 3D virtual world can improve health self-efficacy for weight loss management among adults. *Cyberpsychology, Behavior, and Social Networking*. 2016; 19: 74-79.
210 Belalcazar LM, Reboussin DM, Haffner SM, Hoogeveen RC, Kriska AM, Schwenke DC*, et al.* A 1-year lifestyle intervention for weight loss in individuals with type 2 diabetes reduces high C-reactive protein levels and identifies metabolic predictors of change: from the Look AHEAD (Action for Health in Diabetes) study. *Diabetes Care*. 2010; 33: 2297-303.
211 Belanger-Gravel A, Godin G, Bilodeau A, Poirier P. The effect of implementation intentions on physical activity among obese older adults: a randomised control study. *Psychology & Health*. 2013; 28: 217-33.
212 Belski R, Mori TA, Puddey IB, Sipsas S, Woodman RJ, Ackland TR*, et al.* Effects of lupin-enriched foods on body composition and cardiovascular disease risk factors: a 12-month randomized controlled weight loss trial. *International Journal of Obesity*. 2011; 35: 810-19.
213 Bennett GG, Herring SJ, Puleo E, Stein EK, Emmons KM, Gillman MW. Web-based weight loss in primary care: a randomized controlled trial. *Obesity (Silver Spring, Md)*. 2010; 18: 308-13.

214 Bennett GG, Warner ET, Glasgow RE, Askew S, Goldman J, Ritzwoller DP*, et al.* Obesity treatment for socioeconomically disadvantaged patients in primary care practice. *Archives of Internal Medicine*. 2012; 172: 565-74.
215 Berkley-Patton J, Bowe Thompson C, Bauer AG, Berman M, Bradley-Ewing A, Goggin K*, et al.* A Multilevel Diabetes and CVD Risk Reduction Intervention in African American Churches: Project Faith Influencing Transformation (FIT) Feasibility and Outcomes. *Journal of Racial & Ethnic Health Disparities*. 2020; 7: 1160-71.
216 Bevilaqua CA, Pelloso SM, Marcon SS. Stages of change of behavior in women on a multi-professional program for treatment of obesity. *Revista latino-americana de enfermagem*. 2016; 24: e2809.
217 Bhoite R, Chandrasekaran A, Pratti VL, Satyavrat V, Aacharya S, Mane A*, et al.* Effect of a High-Protein High-Fibre Nutritional Supplement on Lipid Profile in Overweight/Obese Adults with Type 2 Diabetes Mellitus: A 24-Week Randomized Controlled Trial. *Journal of Nutrition and Metabolism*. 2021; 2021 (no pagination).
218 Bhutani S, Klempel MC, Kroeger CM, Trepanowski JF, Phillips SA, Norkeviciute E*, et al.* Alternate day fasting with or without exercise: effects on endothelial function and adipokines in obese humans. *E-spen journal*. 2013; 8: e205-e09.
219 Bhutani S, Klempel MC, Kroeger CM, Trepanowski JF, Varady KA. Alternate day fasting and endurance exercise combine to reduce body weight and favorably alter plasma lipids in obese humans. *Obesity*. 2013; 21: 1370-79.
220 Biteli P, Barbalho SM, Detregiachi CRP, dos Santos Haber JF, Chagas EFB. Dyslipidemia influences the effect of physical exercise on inflammatory markers on obese women in post-menopause: A randomized clinical trial. *Experimental Gerontology*. 2021; 150 (no pagination).
221 Blackford K, Jancey J, Lee AH, James AP, Waddell T, Howat P. Home-based lifestyle intervention for rural adults improves metabolic syndrome parameters and cardiovascular risk factors: a randomised controlled trial. *Preventive Medicine*. 2016; 89: 15-22.
222 Bliss ES, Wong RHX, Howe PRC, Mills DE. The Effects of Aerobic Exercise Training on Cerebrovascular and Cognitive Function in Sedentary, Obese, Older Adults. *Frontiers in aging neuroscience*. 2022; 14: 892343.
223 Blos AP, Drehmer E, Ceca D, Garcia-Esteve A, Lopez-Hernandez L, Fargueta M*, et al.* Effects of a lifestyle intervention program for treating obesity in lower socioeconomic status adults: a randomized controlled trial. *Gazzetta medica italiana archivio per LE scienze mediche*. 2017; 176: 467-77.
224 Blumenthal JA, Babyak MA, Hinderliter A, Watkins LL, Craighead L, Lin PH*, et al.* Effects of the DASH diet alone and in combination with exercise and weight loss on blood pressure and cardiovascular biomarkers in men and women with high blood pressure: the ENCORE study. *Archives of Internal Medicine*. 2010; 170: 126-35.
225 Blumenthal JA, Babyak MA, Sherwood A, Craighead L, Lin PH, Johnson J*, et al.* Effects of the dietary approaches to stop hypertension diet alone and in combination with exercise and caloric restriction on insulin sensitivity and lipids. *Hypertension*. 2010; 55: 1199-205.
226 Blumenthal JA, Sherwood A, Gullette EC, Babyak M, Waugh R, Georgiades A*, et al.* Exercise and weight loss reduce blood pressure in men and women with mild hypertension: effects on cardiovascular, metabolic, and hemodynamic functioning. *Archives of Internal Medicine*. 2000; 160: 1947-58.
227 Bo S, Rahimi F, Goitre I, Properzi B, Ponzo V, Regaldo G*, et al.* Effects of Self-Conditioning Techniques (Self-Hypnosis) in Promoting Weight Loss in Patients with Severe Obesity: A Randomized Controlled Trial. *Obesity*. 2018; 26: 1422-29.

228 Bocalini DS, Lima LS, de Andrade S, Madureira A, Rica RL, Dos Santos RN*, et al.* Effects of circuit-based exercise programs on the body composition of elderly obese women. *Clinical Interventions In Aging*. 2012; 7: 551-56.
229 Bohl M, Bjornshave A, Gregersen S, Hermansen K. Whey and Casein Proteins and Medium-Chain Saturated Fatty Acids from Milk Do Not Increase Low-Grade Inflammation in Abdominally Obese Adults. *The Review of Diabetic Studies*. 2016; 13: 148-57.
230 Bohl M, Bjornshave A, Larsen MK, Gregersen S, Hermansen K. The effects of proteins and medium-chain fatty acids from milk on body composition, insulin sensitivity and blood pressure in abdominally obese adults. *European Journal of Clinical Nutrition*. 2017; 71: 76-82.
231 Bohl M, Bjornshave A, Rasmussen KV, Schioldan AG, Amer B, Larsen MK*, et al.* Dairy proteins, dairy lipids, and postprandial lipemia in persons with abdominal obesity (DairyHealth): a 12-wk, randomized, parallel-controlled, double-blinded, diet intervention study. *American Journal of Clinical Nutrition*. 2015; 101: 870-78.
232 Bolognesi M, Nigg CR, Massarini M, Lippke S. Reducing obesity indicators through brief physical activity counseling (PACE) in Italian primary care settings. *Annals of Behavioral Medicine*. 2006; 31: 179-85.
233 Boraxbekk CJ, Stomby A, Ryberg M, Lindahl B, Larsson C, Nyberg L*, et al.* Diet-Induced Weight Loss Alters Functional Brain Responses during an Episodic Memory Task. *Obesity Facts*. 2015; 8: 261-72.
234 Borradaile KE, Halpern SD, Wyatt HR, Klein S, Hill JO, Bailer B*, et al.* Relationship between treatment preference and weight loss in the context of a randomized controlled trial. *Obesity*. 2012; 20: 1218-22.
235 Botoseneanu A, Chen H, Ambrosius WT, Allore HG, Anton S, Folta SC*, et al.* Effect of Metabolic Syndrome on the Mobility Benefit of a Structured Physical Activity Intervention-The Lifestyle Interventions and Independence for Elders Randomized Clinical Trial. *Journal of the American Geriatrics Society*. 2017; 65: 1244-50.
236 Botton CE, Umpierre D, Rech A, Pfeifer LO, Machado CLF, Teodoro JL*, et al.* Effects of resistance training on neuromuscular parameters in elderly with type 2 diabetes mellitus: A randomized clinical trial. *Experimental Gerontology*. 2018; 113: 141-49.
237 Bowen J, Noakes M, Clifton PM. A high dairy protein, high-calcium diet minimizes bone turnover in overweight adults during weight loss. *Journal of Nutrition*. 2004; 134: 568-73.
238 Braden A, Flatt SW, Boutelle KN, Strong D, Sherwood NE, Rock CL. Emotional eating is associated with weight loss success among adults enrolled in a weight loss program. *Journal of Behavioral Medicine*. 2016; 39: 727-32.
239 Brar JS, Ganguli R, Pandina G, Turkoz I, Berry S, Mahmoud R. Effects of behavioral therapy on weight loss in overweight and obese patients with schizophrenia or schizoaffective disorder. *Journal of Clinical Psychiatry*. 2005; 66: 205-12.
240 Brawley L, Rejeski WJ, Gaukstern JE, Ambrosius WT. Social cognitive changes following weight loss and physical activity interventions in obese, older adults in poor cardiovascular health. *Annals of Behavioral Medicine*. 2012; 44: 353-64.
241 Bray GA, Smith SR, DeJonge L, de Souza R, Rood J, Champagne CM*, et al.* Effect of diet composition on energy expenditure during weight loss: the POUNDS LOST Study. *International Journal of Obesity*. 2012; 36: 448-55.
242 Brehm BJ, Seeley RJ, Daniels SR, D'Alessio DA. A randomized trial comparing a very low carbohydrate diet and a calorie-restricted low fat diet on body weight and cardiovascular risk factors in healthy women. *Journal of clinical endocrinology and metabolism*. 2003; 88: 1617-23.

243 Brehm BJ, Spang SE, Lattin BL, Seeley RJ, Daniels SR, D'Alessio DA. The role of energy expenditure in the differential weight loss in obese women on low-fat and low-carbohydrate diets. *Journal of clinical endocrinology and metabolism*. 2005; 90: 1475-82.
244 Breyer BN, Creasman JM, Richter HE, Myers D, Burgio KL, Wing RR*, et al.* A Behavioral Weight Loss Program and Nonurinary Incontinence Lower Urinary Tract Symptoms in Overweight and Obese Women with Urinary Incontinence: a Secondary Data Analysis of PRIDE. *Journal of Urology*. 2018; 199: 215-22.
245 Brindal E, Freyne J, Saunders I, Berkovsky S, Smith G, Noakes M. Features predicting weight loss in overweight or obese participants in a web-based intervention: randomized trial. *Journal of Medical Internet Research*. 2012; 14: e173.
246 Brinkworth GD, Noakes M, Buckley JD, Keogh JB, Clifton PM. Long-term effects of a very-low-carbohydrate weight loss diet compared with an isocaloric low-fat diet after 12 mo. *American Journal of Clinical Nutrition*. 2009; 90: 23-32.
247 Brinkworth GD, Noakes M, Parker B, Foster P, Clifton PM. Long-term effects of advice to consume a high-protein, low-fat diet, rather than a conventional weight-loss diet, in obese adults with type 2 diabetes: one-year follow-up of a randomised trial. *Diabetologia*. 2004; 47: 1677-86.
248 Brochu M, Malita MF, Messier V, Doucet E, Strychar I, Lavoie JM*, et al.* Resistance training does not contribute to improving the metabolic profile after a 6-month weight loss program in overweight and obese postmenopausal women. *Journal of Clinical Endocrinology & Metabolism*. 2009; 94: 3226-33.
249 Brodie DA, Slade PD. Dietary versus healthy life style interventions in the treatment of obesity: Resultant changes and the prediction of outcome. *Psychology & Health*. 1990; 4: 319-31.
250 Brown C, Goetz J, Hamera E. Weight loss intervention for people with serious mental illness: A randomized controlled trial of the RENEW program. *Psychiatric Services*. 2011; 62: 800-02.
251 Brown C, Goetz J, Hamera E, Gajewski B. Treatment response to the RENEW weight loss intervention in schizophrenia: impact of intervention setting. *Schizophrenia Research*. 2014; 159: 421-25.
252 Brown JC, Sarwer DB, Troxel AB, Sturgeon K, DeMichele AM, Denlinger CS*, et al.* A randomized trial of exercise and diet on body composition in survivors of breast cancer with overweight or obesity. *Breast Cancer Research and Treatment*. 2021; 189(1): 145-54.
253 Brown JC, Yung RL, Gobbie-Hurder A, Shockro L, O'Connor K, Campbell N*, et al.* Randomized trial of a clinic-based weight loss intervention in cancer survivors. *Journal of Cancer Survivorship*. 2018; 12: 186-95.
254 Buckinx F, Gouspillou G, Carvalho LP, Marcangeli V, Boutros GE, Dulac M*, et al.* Effect of High-Intensity Interval Training Combined with L-Citrulline Supplementation on Functional Capacities and Muscle Function in Dynapenic-Obese Older Adults. *Journal of Clinical Medicine*. 2018; 7: 14.
255 Burguera B, Jesus Tur J, Escudero AJ, Alos M, Pagan A, Cortes B*, et al.* An Intensive Lifestyle Intervention Is an Effective Treatment of Morbid Obesity: the TRAMOMTANA Study-A Two-Year Randomized Controlled Clinical Trial. *International journal of endocrinology*. 2015; 2015.
256 Burke L, Lee AH, Pasalich M, Jancey J, Kerr D, Howat P. Effects of a physical activity and nutrition program for seniors on body mass index and waist-to-hip ratio: a randomised controlled trial. *Preventive Medicine*. 2012; 54: 397-401.

257 Burke LE, Conroy MB, Sereika SM, Elci OU, Styn MA, Acharya SD*, et al.* The effect of electronic self-monitoring on weight loss and dietary intake: A randomized behavioral weight loss trial. *Obesity*. 2011; 19: 338-44.
258 Burke LE, Ewing LJ, Ye L, Styn M, Zheng Y, Music E*, et al.* The SELF trial: A self-efficacy-based behavioral intervention trial for weight loss maintenance. *Obesity*. 2015; 23: 2175-82.
259 Burke LE, Hudson AG, Warziski MT, Styn MA, Music E, Elci OU*, et al.* Effects of a vegetarian diet and treatment preference on biochemical and dietary variables in overweight and obese adults: a randomized clinical trial. *American Journal of Clinical Nutrition*. 2007; 86: 588-96.
260 Burke LE, Styn MA, Sereika SM, Conroy MB, Ye L, Glanz K*, et al.* Using mHealth technology to enhance self-monitoring for weight loss: a randomized trial. *American Journal of Preventive Medicine*. 2012; 43: 20-26.
261 Burke LE, Styn MA, Steenkiste AR, Music E, Warziski M, Choo J. A Randomized Clinical Trial Testing Treatment Preference and Two Dietary Options in Behavioral Weight Management: Preliminary Results of the Impact of Diet at 6 Months--PREFER Study. *Obesity*. 2006; 14: 2007-17.
262 Burke LE, Warziski M, Styn MA, Music E, Hudson AG, Sereika SM. A randomized clinical trial of a standard versus vegetarian diet for weight loss: The impact of treatment preference. *International Journal of Obesity*. 2008; 32: 166-76.
263 Burke V, Beilin LJ, Cutt HE, Mansour J, Williams A, Mori TA. A lifestyle program for treated hypertensives improved health-related behaviors and cardiovascular risk factors, a randomized controlled trial. *Journal of Clinical Epidemiology*. 2007; 60: 133-41.
264 Burnette JL, Finkel EJ. Buffering against weight gain following dieting setbacks: An implicit theory intervention. *Journal of Experimental Social Psychology*. 2012; 48: 721-25.
265 Busch AM, Whited MC, Appelhans BM, Schneider KL, Waring ME, DeBiasse MA*, et al.* Reliable change in depression during behavioral weight loss treatment among women with major depression. *Obesity*. 2013; 21: E211-18.
266 Butryn ML, Forman EM, Lowe MR, Gorin AA, Zhang F, Schaumberg K. Efficacy of environmental and acceptance-based enhancements to behavioral weight loss treatment: The ENACT trial. *Obesity*. 2017; 25: 866-72.
267 Butryn ML, Martinelli MK, Crane NT, Godfrey K, Roberts SR, Zhang F*, et al.* Counselor Surveillance of Digital Self-Monitoring Data: A Pilot Randomized Controlled Trial. *Obesity (19307381)*. 2020; 28: 2339-46.
268 Cabrera-Pivaral CE, González-Pérez G, Vega-López MG, Arias-Merino ED. Impact of participatory education on body mass index and blood glucose in obese type-2 diabetics. *Cadernos de Saude Publica*. 2004; 20: 275-81.
269 Cadmus-Bertram L, Nelson SH, Hartman S, Patterson RE, Parker BA, Pierce JP. Randomized trial of a phone- and web-based weight loss program for women at elevated breast cancer risk: the HELP study. *Journal of Behavioral Medicine*. 2016; 39: 551-59.
270 Calleja Fernandez A, Vidal Casariego A, Cano Rodriguez I, Ballesteros Pomar MD. One-year effectiveness of two hypocaloric diets with different protein/carbohydrate ratios in weight loss and insulin resistance. *Nutricion Hospitalaria*. 2012; 27: 2093-101.
271 Camacho-Cardenosa A, Camacho-Cardenosa M, Brazo-Sayavera J, Burtscher M, Timón R, Olcina G. Effects of High-Intensity Interval Training Under Normobaric Hypoxia on Cardiometabolic Risk Markers in Overweight/Obese Women. *High altitude medicine & biology*. 2018; 19: 356-66.

272 Camacho-Cardenosa A, Camacho-Cardenosa M, Olcina G, Timón R, Brazo-Sayavera J. Detraining effect on overweight/obese women after high-intensity interval training in hypoxia. *Scandinavian Journal of Medicine & Science in Sports*. 2019; 29: 535-43.
273 Camargo A, Meneses ME, Rangel-Zuñiga OA, Perez-Martinez P, Marin C, Delgado-Lista J*, et al.* Endoplasmic reticulum stress in adipose tissue determines postprandial lipoprotein metabolism in metabolic syndrome patients. *Molecular Nutrition & Food Research*. 2013; 57: 2166-76.
274 Camolas J, Santos O, Moreira P, do Carmo I. INDIVIDUO: results from a patient-centered lifestyle intervention for obesity surgery candidates. *Obesity Research & Clinical Practice*. 2017; 11: 475-88.
275 Campanati A, Molinelli E, Ganzetti G, Giuliodori K, Minetti I, Taus M*, et al.* The effect of low-carbohydrates calorie-restricted diet on visceral adipose tissue and metabolic status in psoriasis patients receiving TNF-alpha inhibitors: results of an open label controlled, prospective, clinical study. *Journal of Dermatological Treatment*. 2017; 28: 206-12.
276 Campbell DD, Meckling KA. Effect of the protein: carbohydrate ratio in hypoenergetic diets on metabolic syndrome risk factors in exercising overweight and obese women. *British Journal of Nutrition*. 2012; 108: 1658-71.
277 Campbell L, Wallman K, Green D. The effects of intermittent exercise on physiological outcomes in an obese population: Continuous versus interval walking. *Journal of Sports Science and Medicine*. 2010; 9: 24-30.
278 Campbell MK, Carr C, Devellis B, Switzer B, Biddle A, Amamoo MA*, et al.* A randomized trial of tailoring and motivational interviewing to promote fruit and vegetable consumption for cancer prevention and control. *Annals of Behavioral Medicine*. 2009; 38: 71-85.
279 Campbell WW, Haub MD, Wolfe RR, Ferrando AA, Sullivan DH, Apolzan JW*, et al.* Resistance training preserves fat-free mass without impacting changes in protein metabolism after weight loss in older women. *Obesity*. 2009; 17: 1332-39.
280 Cardillo S, Seshadri P, Iqbal N. The effects of low-carbohydrate versus low-fat diet on asdipocytokines in severely obese adult: three-year follow-up of a randomized trial. *European review for medical and pharmacological sciences*. 2006; 10: 99-106.
281 Carels RA, Borushok J, Taylor M, Rossi J, Hoffmann D, Burmeister J*, et al.* A randomized trial comparing two approaches to weight loss. *Journal of Health Psychology*. 2017; 22: 943-50.
282 Carels RA, Burmeister JM, Koball AM, Oehlhof MW, Hinman N, LeRoy M*, et al.* A randomized trial comparing two approaches to weight loss: differences in weight loss maintenance. *Journal of Health Psychology*. 2014; 19: 296-311.
283 Carels RA, Darby L, Cacciapaglia HM, Douglass OM, Harper J, Kaplar ME*, et al.* Applying a stepped-care approach to the treatment of obesity. *Journal of Psychosomatic Research*. 2005; 59: 375-83.
284 Carels RA, Darby L, Cacciapaglia HM, Konrad K, Coit C, Harper J*, et al.* Using motivational interviewing as a supplement to obesity treatment: A stepped-care approach. *Health Psychology*. 2007; 26: 369-74.
285 Carels RA, Darby LA, Douglass OM, Cacciapaglia HM, Rydin S. Education on the glycemic index of foods fails to improve treatment outcomes in a behavioral weight loss program. *Eating Behaviors*. 2005; 6: 145-50.
286 Carels RA, Miller JC, Shonrock AT, Byrd JR, Haley E. Exploring the addition of self-compassion skills training to a behavioral weight loss program delivered using video conferencing software. *Journal of Contextual Behavioral Science*. 2021; 21: 196-202.

287 Carels RA, Young KM, Coit CB, Darby LA, Clayton AM, Spencer AC*, et al.* The failure of therapist assistance and stepped-care to improve weight loss outcomes. *Obesity*. 2008; 16: 1460-62.
288 Carels RA, Young KM, Koball A, Gumble A, Darby LA, Oehlhof MW*, et al.* Transforming your life: An environmental modification approach to weight loss. *Journal of Health Psychology*. 2011; 16: 430-38.
289 Carr LJ, Bartee RT, Dorozynski CM, Broomfield JF, Smith ML, Smith DT. Eight-month follow-up of physical activity and central adiposity: results from an Internet-delivered randomized control trial intervention. *Journal of Physical Activity & Health*. 2009; 6: 444-55.
290 Carter MC, Burley VJ, Nykjaer C, Cade JE. Adherence to a smartphone application for weight loss compared to website and paper diary: pilot randomized controlled trial. *Journal of Medical Internet Research*. 2013; 15: e32.
291 Carter S, Clifton PM, Keogh JB. The effects of intermittent compared to continuous energy restriction on glycaemic control in type 2 diabetes; a pragmatic pilot trial. *Diabetes research and clinical practice*. 2016; 122: 106-12.
292 Casas R, Ribó-Coll M, Ros E, Fitó M, Lamuela-Raventos R-M, Salas-Salvadó J*, et al.* Change to a healthy diet in people over 70 years old: the PREDIMED experience. *European Journal of Nutrition*. 2022; 61: 1429-44.
293 Casas R, Sacanella E, Urpi-Sarda M, Chiva-Blanch G, Ros E, Martinez-Gonzalez MA*, et al.* The effects of the Mediterranean diet on biomarkers of vascular wall inflammation and plaque vulnerability in subjects with high risk for cardiovascular disease. A randomized trial. *PloS one*. 2014; 9.
294 Casas R, Sacanella E, Urpi-Sarda M, Corella D, Castanr O, Lamuela-Raventos RM*, et al.* Long-term immunomodulatory effects of a mediterranean diet in adults at high risk of cardiovascular disease in the prevencion con dieta mediterranea (predimed) randomized controlled trial. *Journal of Nutrition*. 2016; 146: 1684-93.
295 Casas-Agustench P, López-Uriarte P, Bulló M, Ros E, Cabré-Vila JJ, Salas-Salvadó J. Effects of one serving of mixed nuts on serum lipids, insulin resistance and inflammatory markers in patients with the metabolic syndrome. *Nutrition, metabolism, and cardiovascular diseases : NMCD*. 2011; 21: 126-35.
296 Castelnuovo G, Manzoni GM, Cuzziol P, Cesa GL, Corti S, Tuzzi C*, et al.* TECNOB study: Ad interim results of a randomized controlled trial of a multidisciplinary telecare intervention for obese patients with type-2 diabetes. *Clinical practice and epidemiology in mental health*. 2011; 7.
297 Castelnuovo G, Manzoni GM, Villa V, Cesa GL, Molinari E. Brief strategic therapy vs cognitive behavioral therapy for the inpatient and telephone-based outpatient treatment of binge eating disorder: the STRATOB randomized controlled clinical trial. *Clinical practice and epidemiology in mental health*. 2011; 7: 29-37.
298 Cavalcante EF, Ribeiro AS, do Nascimento MA, Silva AM, Tomeleri CM, Nabuco HCG*, et al.* Effects of Different Resistance Training Frequencies on Fat in Overweight/Obese Older Women. *International Journal of Sports Medicine*. 2018; 39: 527-34.
299 Cayir Y, Aslan SM, Akturk Z. The effect of pedometer use on physical activity and body weight in obese women. *European journal of sport science*. 2015; 15: 351-56.
300 Celis-Morales C, Livingstone KM, Petermann-Rocha F, Navas-Carretero S, San-Cristobal R, O'Donovan CB*, et al.* Frequent nutritional feedback, personalized advice, and behavioral changes: Findings from the European Food4Me internet-based RCT. *American Journal of Preventive Medicine*. 2019; 57: 209-19.

301 Chang SH, Chen MC, Chien NH, Lin HF. Effectiveness of community-based exercise intervention programme in obese adults with metabolic syndrome. *Journal of Clinical Nursing*. 2016; 25: 2579-89.
302 Chang SH, Chien NH, Yu CY. Long-Term Lifestyle Intervention in Elderly With Metabolic Syndrome. *Clinical Nursing Research*. 2019; 28: 658-75.
303 Chao AM, Wadden TA, Gorin AA, Shaw Tronieri J, Pearl RL, Bakizada ZM*, et al.* Binge Eating and Weight Loss Outcomes in Individuals with Type 2 Diabetes: 4-Year Results from the Look AHEAD Study. *Obesity*. 2017; 25: 1830-37.
304 Chao D, Espeland MA, Farmer D, Register TC, Lenchik L, Applegate WB*, et al.* Effect of voluntary weight loss on bone mineral density in older overweight women. *Journal of the American Geriatrics Society*. 2000; 48: 753-59.
305 Chapman MJ, Craven MJ, Chadwick DD. Fighting fit? An evaluation of health practitioner input to improve healthy living and reduce obesity for adults with learning disabilities. *Journal of Intellectual Disabilities*. 2005; 9: 131-44.
306 Chapman MJ, Craven MJ, Chadwick DD. Following up Fighting Fit: The long-term impact of health practitioner input on obesity and BMI amongst adults with intellectual disabilities. *Journal of Intellectual Disabilities*. 2008; 12: 309-23.
307 Chen L, Appel LJ, Loria C, Lin PH, Champagne CM, Elmer PJ*, et al.* Reduction in consumption of sugar-sweetened beverages is associated with weight loss: the PREMIER trial. *American Journal of Clinical Nutrition*. 2009; 89: 1299-306.
308 Chen TC, Chung-Chan H, Kuo-Wei T, Chih-Chiao H, Kazunori N. Effects of Descending Stair Walking on Health and Fitness of Elderly Obese Women. *Medicine & Science in Sports & Exercise*. 2017; 49: 1614-22.
309 Cheskin LJ, Mitchell AM, Jhaveri AD, Mitola AH, Davis LM, Lewis RA*, et al.* Efficacy of meal replacements versus a standard food-based diet for weight loss in type 2 diabetes: a controlled clinical trial. *Diabetes Educator*. 2008; 34: 118-27.
310 Cheung KL, Schwabe I, Walthouwer MJL, Oenema A, Lechner L, de Vries H. Effectiveness of a Video-Versus Text-Based Computer-Tailored Intervention for Obesity Prevention after One Year: a Randomized Controlled Trial. *International journal of environmental research and public health*. 2017; 14.
311 Cheyette C. Weight no more: a randomised controlled trial for people with type 2 diabetes on insulin therapy. *Practical diabetes international*. 2007; 24: 450-56.
312 Chhajer B, Singh V, Kumari G, Lohmor M. Effect of yoga based lifestyle intervention on coronary artery disease patients. *Biomedical and pharmacology journal*. 2018; 11: 1275-89.
313 Chmelo EA, Beavers DP, Lyles MF, Marsh AP, Nicklas BJ, Beavers KM. Legacy effects of short-term intentional weight loss on total body and thigh composition in overweight and obese older adults. *Nutrition & Diabetes*. 2016; 6: e203.
314 Chmelo EA, Crotts CI, Newman JC, Brinkley TE, Lyles MF, Leng X*, et al.* Heterogeneity of physical function responses to exercise training in older adults. *Journal of the American Geriatrics Society*. 2015; 63: 462-69.
315 Choi EJ, Kim YJ, Lee SY. Effects of Electrical Muscle Stimulation on Waist Circumstance in Adults with Adbominal Obesity: a Randomized, Double-blind, Sham-Controlled Trial. *JNMA; journal of the Nepal Medical Association*. 2018; 56: 904-11.
316 Choo J, Lee J, Cho JH, Burke LE, Sekikawa A, Jae SY. Effects of weight management by exercise modes on markers of subclinical atherosclerosis and cardiometabolic profile among women with abdominal obesity: a randomized controlled trial. *BMC Cardiovascular Disorders*. 2014; 14: 82.

317 Christian JG, Bessesen DH, Byers TE, Christian KK, Goldstein MG, Bock BC. Clinic-based support to help overweight patients with type 2 diabetes increase physical activity and lose weight. *Archives of Internal Medicine*. 2008; 168: 141-46.
318 Chua SD, Jr., Messier SP, Legault C, Lenz ME, Thonar EJ, Loeser RF. Effect of an exercise and dietary intervention on serum biomarkers in overweight and obese adults with osteoarthritis of the knee. *Osteoarthritis & Cartilage*. 2008; 16: 1047-53.
319 Chun-De L, Jau-Yih T, Li-Fong L, Shih-Wei H, Jan-Wen K, Lin-Chuan C*, et al.* Effects of elastic resistance exercise on body composition and physical capacity in older women with sarcopenic obesity: A CONSORT-compliant prospective randomized controlled trial. *Medicine*. 2017; 96: 1-8.
320 Church TS, Earnest CP, Skinner JS, Blair SN. Effects of different doses of physical activity on cardiorespiratory fitness among sedentary, overweight or obese postmenopausal women with elevated blood pressure: a randomized controlled trial. *Jama*. 2007; 297: 2081-91.
321 Claessens M, van Baak MA, Monsheimer S, Saris WH. The effect of a low-fat, high-protein or high-carbohydrate ad libitum diet on weight loss maintenance and metabolic risk factors. *International journal of obesity (2005)*. 2009; 33: 296-304.
322 Clark JS, Dyer KA, Davis CR, Shivappa N, Hebert JR, Woodman R*, et al.* Adherence to a Mediterranean Diet for 6 Months Improves the Dietary Inflammatory Index in a Western Population: Results from the MedLey Study. *Nutrients*. 2023; 15: 11.
323 Clifton PM, Keogh JB, Foster PR, Noakes M. Effect of weight loss on inflammatory and endothelial markers and FMD using two low-fat diets. *International journal of obesity (2005)*. 2005; 29: 1445-51.
324 Cohen MD, D'Amico FJ, Merenstein JH. Weight reduction in obese hypertensive patients. *Family Medicine*. 1991; 23: 25-28.
325 Coker RH, Hays NP, Williams RH, Brown AD, Freeling SA, Kortebein PM*, et al.* Exercise-induced changes in insulin action and glycogen metabolism in elderly adults. *Medicine & Science in Sports & Exercise*. 2006; 38: 433-38.
326 Coker RH, Williams RH, Kortebein PM, Sullivan DH, Evans WJ. Influence of exercise intensity on abdominal fat and adiponectin in elderly adults. *Metabolic Syndrome & Related Disorders*. 2009; 7: 363-68.
327 Coker RH, Williams RH, Yeo SE, Kortebein PM, Bodenner DL, Kern PA*, et al.* The impact of exercise training compared to caloric restriction on hepatic and peripheral insulin resistance in obesity. *Journal of Clinical Endocrinology & Metabolism*. 2009; 94: 4258-66.
328 Coles LT, Fletcher EA, Galbraith CE, Clifton PM. Patient freedom to choose a weight loss diet in the treatment of overweight and obesity: a randomized dietary intervention in type 2 diabetes and pre-diabetes. *International Journal of Behavioral Nutrition & Physical Activity*. 2014; 11: 64.
329 Collins CE, Morgan PJ, Jones P, Fletcher K, Martin J, Aguiar EJ*, et al.* A 12-week commercial web-based weight-loss program for overweight and obese adults: Randomized controlled trial comparing basic versus enhanced features. *Journal of Medical Internet Research*. 2012; 14: 128-43.
330 Collins T, Geana M, Overton K, Benton M, Lu L, Khan F*, et al.* Use of a Smartphone App Versus Motivational Interviewing to Increase Walking Distance and Weight Loss in Overweight/Obese Adults With Peripheral Artery Disease: Pilot Randomized Trial. *JMIR Formative Research*. 2022; 6: e30295.

331 Conley M, Le Fevre L, Haywood C, Proietto J. Is two days of intermittent energy restriction per week a feasible weight loss approach in obese males? A randomised pilot study. *Nutrition & Dietetics*. 2018; 75: 65-72.
332 Cooper Z, Doll HA, Hawker DM, Byrne S, Bonner G, Eeley E*, et al.* Testing a new cognitive behavioural treatment for obesity: a randomized controlled trial with three-year follow-up. *Behaviour research and therapy*. 2010; 48: 706-13.
333 Cornelius T, Gettens K, Gorin AA. Dyadic Dynamics in a Randomized Weight Loss Intervention. *Annals of Behavioral Medicine*. 2016; 50: 506-15.
334 Cornelius T, Gettens K, Lenz E, Wojtanowski AC, Foster GD, Gorin AA. How prescriptive support affects weight loss in weight-loss intervention participants and their untreated spouses. *Health Psychology*. 2018; 37: 775-81.
335 Coughlin JW, Brantley PJ, Champagne CM, Vollmer WM, Stevens VJ, Funk K*, et al.* The impact of continued intervention on weight: five-year results from the weight loss maintenance trial. *Obesity (Silver Spring, Md)*. 2016; 24: 1046-53.
336 Courteix D, Valente-dos-Santos J, Ferry B, Lac G, Lesourd B, Chapier R*, et al.* Multilevel Approach of a 1-Year Program of Dietary and Exercise Interventions on Bone Mineral Content and Density in Metabolic Syndrome - the RESOLVE Randomized Controlled Trial. *PloS one*. 2015; 10: 20.
337 Cox TL, Krukowski R, Love SJ, Eddings K, DiCarlo M, Chang JY*, et al.* Stress management–augmented behavioral weight loss intervention for African American women: A pilot, randomized controlled trial. *Health Education & Behavior*. 2013; 40: 78-87.
338 Crane MM, Gavin K, Wolfson J, Linde JA. How Accurate are Recalls of Self-Weighing Frequency? Data from a 24-Month Randomized Trial. *Obesity (Silver Spring, Md)*. 2018; 26: 1296-302.
339 Crane MM, Ward DS, Lutes LD, Bowling JM, Tate DF. Theoretical and Behavioral Mediators of a Weight Loss Intervention for Men. *Annals of Behavioral Medicine*. 2016; 50: 460-70.
340 Creasy SA, Wayland L, Panter SL, Purcell SA, Rosenberg R, Willis EA*, et al.* Effect of Morning and Evening Exercise on Energy Balance: A Pilot Study. *Nutrients*. 2022; 14: 816.
341 Cunha PM, Ribeiro AS, Tomeleri CM, Schoenfeld BJ, Silva AM, Souza MF*, et al.* The effects of resistance training volume on osteosarcopenic obesity in older women. *Journal of Sports Sciences*. 2018; 36: 1564-71.
342 D'Eramo-Melkus GA, Wylie-Rosett J, Hagan JA. Metabolic impact of education in NIDDM. *Diabetes Care*. 1992; 15: 864-69.
343 Dale KS, McAuley KA, Taylor RW, Williams SM, Farmer VL, Hansen P*, et al.* Determining optimal approaches for weight maintenance: a randomized controlled trial. *CMAJ Canadian Medical Association Journal*. 2009; 180: E39-46.
344 Dalle Grave R, Todesco T, Banderali A, Guardini S. Cognitive-behavioural guided self-help for obesity: A preliminary research. *Eating and weight disorders*. 2004; 9: 69-76.
345 Dallow CB, Anderson J. Using self-efficacy and a transtheoretical model to develop a physical activity intervention for obese women. *American Journal of Health Promotion*. 2003; 17: 373-81.
346 Daly ME, Paisey R, Paisey R, Millward BA, Eccles C, Williams K*, et al.* Short-term effects of severe dietary carbohydrate-restriction advice in Type 2 diabetes--a randomized controlled trial. *Diabetic Medicine*. 2006; 23: 15-20.
347 Daly RM, Dunstan DW, Owen N, Jolley D, Shaw JE, Zimmet PZ. Does high-intensity resistance training maintain bone mass during moderate weight loss in older overweight adults with type 2 diabetes? *Osteoporosis International*. 2005; 16: 1703-12.

348 Damschroder LJ, Lutes LD, Kirsh S, Kim HM, Gillon L, Holleman RG*, et al.* Small-changes obesity treatment among veterans: 12-month outcomes. *American Journal of Preventive Medicine*. 2014; 47: 541-53.
349 Dassen FCM, Houben K, Van Breukelen GJP, Jansen A. Gamified working memory training in overweight individuals reduces food intake but not body weight. *Appetite*. 2018; 124: 89-98.
350 Dastan B, Afshar Zanjani S, Froueddin Adl A, Habibi M. The effectiveness of dialectical behaviour therapy for treating women with obesity suffering from BED: A feasibility and pilot study. *Clinical Psychologist*. 2020; 24: 133-42.
351 Daubenmier J, Lin J, Blackburn E, Hecht FM, Kristeller J, Maninger N*, et al.* Changes in stress, eating, and metabolic factors are related to changes in telomerase activity in a randomized mindfulness intervention pilot study. *Psychoneuroendocrinology*. 2012; 37: 917-28.
352 Daubenmier J, Moran PJ, Kristeller J, Acree M, Bacchetti P, Kemeny ME*, et al.* Effects of a mindfulness-based weight loss intervention in adults with obesity: A randomized clinical trial. *Obesity*. 2016; 24: 794-804.
353 Daumit GL, Dickerson FB, Wang N-Y, Dalcin A, Jerome GJ, Anderson CAM*, et al.* A behavioral weight-loss intervention in persons with serious mental illness. *The New England Journal of Medicine*. 2013; 368: 1594-602.
354 Davis LM, Coleman C, Kiel J, Rampolla J, Hutchisen T, Ford L*, et al.* Efficacy of a meal replacement diet plan compared to a food-based diet plan after a period of weight loss and weight maintenance: a randomized controlled trial. *Nutrition Journal*. 2010; 9: 11.
355 Davis Martin P, Rhode PC, Dutton GR, Redmann SM, Ryan DH, Brantley PJ. A primary care weight management intervention for low-income African-American women. *Obesity*. 2006; 14: 1412-20.
356 Davis NJ, Tomuta N, Isasi CR, Leung V, Wylie-Rosett J. Diabetes-specific quality of life after a low-carbohydrate and low-fat dietary intervention. *Diabetes Educator*. 2012; 38: 250-55.
357 Davis NJ, Tomuta N, Schechter C, Isasi CR, Segal-Isaacson CJ, Stein D*, et al.* Comparative study of the effects of a 1-year dietary intervention of a low-carbohydrate diet versus a low-fat diet on weight and glycemic control in type 2 diabetes. *Diabetes Care*. 2009; 32: 1147-52.
358 de Bont AJ, Baker IA, St Leger AS, Sweetnam PM, Wragg KG, Stephens SM*, et al.* A randomised controlled trial of the effect of low fat diet advice on dietary response in insulin independent diabetic women. *Diabetologia*. 1981; 21: 529-33.
359 de Luis D, Izaola O, Primo D, Aller R. Role of rs670 variant of APOA1 gene on metabolic response after a high fat vs. a low fat hypocaloric diets in obese human subjects. *Journal of Diabetes & its Complications*. 2019; 33: 249-54.
360 de Luis DA, Aller R, Izaola O, Conde R, Eiros Bouza JM. Genetic variation in the beta 3-adrenoreceptor gene (Trp64Arg polymorphism) and its influence on anthropometric parameters and insulin resistance under a high monounsaturated versus a high polyunsaturated fat hypocaloric diet. *Annals of Nutrition & Metabolism*. 2013; 62: 303-09.
361 de Luis DA, Aller R, Izaola O, de la Fuente B, Conde R, Sagrado MG*, et al.* Evaluation of weight loss and adipocytokines levels after two hypocaloric diets with different macronutrient distribution in obese subjects with rs9939609 gene variant. *Diabetes/metabolism research and reviews*. 2012; 28: 663-68.

362 de Luis DA, Aller R, Izaola O, Gonzalez Sagrado M, Bellioo D, Conde R. Effects of a low-fat versus a low-carbohydrate diet on adipocytokines in obese adults. *Hormone research*. 2007; 67: 296-300.
363 de Luis DA, Aller R, Izaola O, Gonzalez Sagrado M, Conde R. Effect of two different hypocaloric diets in transaminases and insulin resistance in nonalcoholic fatty liver disease and obese patients. *Nutricion Hospitalaria*. 2010; 25: 730-35.
364 de Luis DA, Aller R, Izaola O, Gonzalez Sagrado M, Conde R. Role of G308 promoter variant of tumor necrosis factor alpha gene on weight loss and metabolic parameters after a high monounsaturated versus a high polyunsaturated fat hypocaloric diets. *Medicina Clinica*. 2013; 141: 189-93.
365 De Luis DA, Aller R, Izaola O, Pacheco D. Role of Rs9939609 Fto Gene Variant in Weight Loss, Insulin Resistance and Metabolic Parameters after a High Monounsaturated Vs a High Polyunsaturated Fat Hypocaloric Diets. *Nutricion Hospitalaria*. 2015; 32: 175-81.
366 de Luis DA, Aller R, Izaola O, Romero E. Effects of a high-protein/low-carbohydrate versus a standard hypocaloric diet on adipocytokine levels and cardiovascular risk factors during 9 months, role of rs6923761 gene variant of glucagon-like peptide 1 receptor. *Journal of Endocrinological Investigation*. 2015; 38: 1183-89.
367 de Luis DA, Aller R, Izaola O, Romero E. Effect of -55CT Polymorphism of UCP3 on Insulin Resistance and Cardiovascular Risk Factors after a High Protein/Low Carbohydrate versus a Standard Hypocaloric Diet. *Annals of Nutrition & Metabolism*. 2016; 68: 157-63.
368 de Luis DA, Izaola O, de la Fuente B, Primo D, Romero E. Role of Fatty Acid-Binding Protein 2 Ala54Thr Genotype on Weight Loss and Cardiovascular Risk Factors after a High-Protein/Low-Carbohydrate versus a Standard Hypocaloric Diet during 9 Months. *Annals of Nutrition & Metabolism*. 2015; 67: 81-86.
369 De Luis DA, Izaola O, García Alonso M, González Sagrado M, Conde R, Aller R*, et al.* Randomized clinical trial between nutritional counselling and commercial hypocaloric diet in weight loss in obese patients with chronic arthropathy. *Medicina Clinica*. 2009; 132: 735-39.
370 de Luis DA, Izaola O, Primo D, Aller R. Polymorphism rs16147 of the Neuropeptide Y Gene Modifies the Response of Cardiovascular Risk Biomarkers and Adipokines to Two Hypocaloric Diets. *Journal of nutrigenetics and nutrigenomics*. 2017; 10: 63-72.
371 de Luis DA, Izaola O, Primo D, Aller R. Impact of 2 Different Hypocaloric Diets on Serum Omentin Levels in Obese Subjects. *Annals of Nutrition & Metabolism*. 2018; 73: 138-44.
372 de Luis DA, Izaola O, Primo D, Aller R. Dietary-fat effect of the rs10830963 polymorphism in MTNR1B on insulin resistance in response to 3 months weight-loss diets. *Endocrinologia Diabetes y Nutricion*. 2020; 67: 43-52.
373 de Luis DA, Izaola O, Primo D, Aller R, de Luis Román DA, Izaola Jáuregui O*, et al.* Influencia de la variante rs670 del gen APOA1 en la respuesta HDL sérica a una dieta hipocalórica enriquecida con grasas poliinsaturadas frente a una enriquecida con grasas monoinsaturadas. *Nutricion Hospitalaria*. 2019; 36: 1288-96.
374 de Luis DA, Izaola O, Primo D, Aller R, Ortola A, Gomez E*, et al.* The association of SNP276G>T at adiponectin gene with insulin resistance and circulating adiponectin in response to two different hypocaloric diets. *Diabetes Research & Clinical Practice*. 2018; 137: 93-99.
375 de Luis DA, Mulero I, Primo D, Izaola O, Aller R. Effects of polymorphism rs3123554 in the cannabinoid receptor gene type 2 (CB2R) on metabolic and adiposity parameters after weight loss with two hypocaloric diets. *Diabetes Research & Clinical Practice*. 2018; 139: 339-47.

376 De Luis DA, Primo D, Izaola O, Aller R. Adiponectin Gene Variant rs266729 Interacts with Different Macronutrient Distribution of Two Different Hypocaloric Diets. *Lifestyle Genomics*. 2020; 13: 20-27.
377 de Luis DA, Romero E, Izaola O, Primo D, Aller R. Cardiovascular Risk Factors and Insulin Resistance after Two Hypocaloric Diets with Different Fat Distribution in Obese Subjects: Effect of the rs10767664 Gene Variant in Brain-Derived Neurotrophic Factor. *Journal of Nutrigenetics & Nutrigenomics*. 2017; 10: 163-71.
378 de Luis DA, Sagrado MG, Conde R, Aller R, Izaola O. The effects of two different hypocaloric diets on glucagon-like peptide 1 in obese adults, relation with insulin response after weight loss. *Journal of diabetes and its complications*. 2009; 23: 239-43.
379 de Luis Roman DA, Izaola Jauregui O, Primo D, Aller R. [Influence of rs670 variant of APOA1 gene on serum HDL response to an enriched-polyunsaturated vs. an enriched-monounsaturated fat hypocaloric diet]. *Nutricion Hospitalaria*. 2019; 36: 1288-95.
380 de Mello VD, Kolehmainen M, Pulkkinen L, Schwab U, Mager U, Laaksonen DE*, et al.* Downregulation of genes involved in NFkappaB activation in peripheral blood mononuclear cells after weight loss is associated with the improvement of insulin sensitivity in individuals with the metabolic syndrome: the GENOBIN study. *Diabetologia*. 2008; 51: 2060-67.
381 de Mello VD, Kolehmainen M, Schwab U, Mager U, Laaksonen DE, Pulkkinen L*, et al.* Effect of weight loss on cytokine messenger RNA expression in peripheral blood mononuclear cells of obese subjects with the metabolic syndrome. *Metabolism: Clinical & Experimental*. 2008; 57: 192-99.
382 de Oliveira PA, Kovacs C, Moreira P, Magnoni D, Saleh MH, Faintuch J. Unsaturated Fatty Acids Improve Atherosclerosis Markers in Obese and Overweight Non-diabetic Elderly Patients. *Obesity Surgery*. 2017; 27: 2663-71.
383 de Souza RJ, Bray GA, Carey VJ, Hall KD, LeBoff MS, Loria CM*, et al.* Effects of 4 weight-loss diets differing in fat, protein, and carbohydrate on fat mass, lean mass, visceral adipose tissue, and hepatic fat: results from the POUNDS LOST trial. *American Journal of Clinical Nutrition*. 2012; 95: 614-25.
384 Del Giglio M, Gisondi P, Tessari G, Girolomoni G. Weight reduction alone may not be sufficient to maintain disease remission in obese patients with psoriasis: a randomized, investigator-blinded study. *Dermatology*. 2012; 224: 31-37.
385 Delahanty LM, Otto A, Vitolins MZ, Brancati FL, Swartz L, Cheskin L*, et al.* Four-year weight losses in the look AHEAD study: Factors associated with long-term success. *Obesity*. 2011; 19: 1987-98.
386 DeLany JP, Kelley DE, Hames KC, Jakicic JM, Goodpaster BH. Effect of physical activity on weight loss, energy expenditure, and energy intake during diet induced weight loss. *Obesity*. 2014; 22: 363-70.
387 Delbridge EA, Prendergast LA, Pritchard JE, Proietto J. One-year weight maintenance after significant weight loss in healthy overweight and obese subjects: does diet composition matter? *American Journal of Clinical Nutrition*. 2009; 90: 1203-14.
388 DeLucia JL, Kalodner CR. An individualized cognitive intervention: Does it increase the efficacy of behavioral interventions for obesity? *Addictive Behaviors*. 1990; 15: 473-79.
389 Delucia JL, Kalodner CR, Horan JJ. The effect of two nutritional software programs used as adjuncts to the behavioral treatment of obesity. *Journal of Substance Abuse*. 1988; 1: 203-08.
390 Demark-Wahnefried W, Colditz GA, Rock CL, Sedjo RL, Liu J, Wolin KY*, et al.* Quality of life outcomes from the Exercise and Nutrition Enhance Recovery and Good Health for You

(ENERGY)-randomized weight loss trial among breast cancer survivors. *Breast Cancer Research & Treatment*. 2015; 154: 329-37.
391 Demark-Wahnefried W, Jones LW, Snyder DC, Sloane RJ, Kimmick GG, Hughes DC*, et al.* Daughters and Mothers Against Breast Cancer (DAMES): main outcomes of a randomized controlled trial of weight loss in overweight mothers with breast cancer and their overweight daughters. *Cancer*. 2014; 120: 2522-34.
392 Demark-Wahnefried W, Morey MC, Sloane R, Snyder DC, Miller PE, Hartman TJ*, et al.* Reach out to enhance wellness home-based diet-exercise intervention promotes reproducible and sustainable long-term improvements in health behaviors, body weight, and physical functioning in older, overweight/obese cancer survivors. *Journal of Clinical Oncology*. 2012; 30: 2354-61.
393 Dengel DR, Pratley RE, Hagberg JM, Rogus EM, Goldberg AP. Distinct effects of aerobic exercise training and weight loss on glucose homeostasis in obese sedentary men. *Journal of applied physiology (Bethesda, Md : 1985)*. 1996; 81: 318-25.
394 Desouza CV, Padala PR, Haynatzki G, Anzures P, Demasi C, Shivaswamy V. Role of apathy in the effectiveness of weight management programmes. *Diabetes, Obesity & Metabolism*. 2012; 14: 419-23.
395 Di Mauro S, Spallina G, Leotta C, Giardina M, Di Fazio I, Malaguarnera M*, et al.* The effects of caloric restriction and controlled physical exercise on hypertension in the elderly. *Archives of gerontology and geriatrics*. 1998; 27: 1-8.
396 Di Rosa C, Lattanzi G, Spiezia C, Imperia E, Piccirilli S, Beato I*, et al.* Mediterranean Diet versus Very Low-Calorie Ketogenic Diet: Effects of Reaching 5% Body Weight Loss on Body Composition in Subjects with Overweight and with Obesity-A Cohort Study. *International Journal of Environmental Research & Public Health [Electronic Resource]*. 2022; 19: 11.
397 Dias-Júnior SA, Reis M, de Carvalho-Pinto RM, Stelmach R, Halpern A, Cukier A. Effects of weight loss on asthma control in obese patients with severe asthma. *The european respiratory journal*. 2014; 43: 1368-77.
398 Diedrich A, Munroe DJ, Romano M. Promoting physical activity for persons with diabetes. *Diabetes Educator*. 2010; 36: 132-40.
399 Dieli-Conwright CM, Courneya KS, Demark-Wahnefried W, Sami N, Lee K, Sweeney FC*, et al.* Aerobic and resistance exercise improves physical fitness, bone health, and quality of life in overweight and obese breast cancer survivors: a randomized controlled trial. *Breast Cancer Research*. 2018; 20: 124.
400 Dieli-Conwright CM, Sweeney FC, Courneya KS, Tripathy D, Sami N, Lee K*, et al.* Hispanic ethnicity as a moderator of the effects of aerobic and resistance exercise in survivors of breast cancer. *Cancer*. 2019; 125: 910-20.
401 DiMarco ID, Klein DA, Clark VL, Wilson GT. The use of motivational interviewing techniques to enhance the efficacy of guided self-help behavioral weight loss treatment. *Eating Behaviors*. 2009; 10: 134-36.
402 Dimitrov Ulian M, Pinto AJ, de Morais Sato P, F BB, Lopes de Campos-Ferraz P, Coelho D*, et al.* Effects of a new intervention based on the Health at Every Size approach for the management of obesity: the "Health and Wellness in Obesity" study. *PloS one*. 2018; 13: e0198401.
403 DiPietro L, Seeman TE, Stachenfeld NS, Katz LD, Nadel ER. Moderate-intensity aerobic training improves glucose tolerance in aging independent of abdominal adiposity. *Journal of the American Geriatrics Society*. 1998; 46: 875-79.

404 Ditschuneit HH, Flechtner-Mors M. Value of structured meals for weight management: risk factors and long-term weight maintenance. *Obesity Research*. 2001; 9 Suppl 4: 284S-89S.
405 Ditschuneit HH, Flechtner-Mors M, Johnson TD, Adler G. Metabolic and weight-loss effects of a long-term dietary intervention in obese patients. *American Journal of Clinical Nutrition*. 1999; 69: 198-204.
406 Ditschuneit HH, Frier HI, Flechtner-Mors M. Lipoprotein responses to weight loss and weight maintenance in high-risk obese subjects. *European Journal of Clinical Nutrition*. 2002; 56: 264-70.
407 Djuric Z, DiLaura NM, Jenkins I, Darga L, Jen CK, Mood D*, et al.* Combining weight-loss counseling with the weight watchers plan for obese breast cancer survivors. *Obesity Research*. 2002; 10: 657-65.
408 Djuric Z, Mirasolo J, Kimbrough L, Brown DR, Heilbrun LK, Canar L*, et al.* A pilot trial of spirituality counseling for weight loss maintenance in African American breast cancer survivors. *Journal of the National Medical Association*. 2009; 101: 552-64.
409 Domenech M, Serra-Mir M, Roth I, Freitas-Simoes T, Valls-Pedret C, Cofan M*, et al.* Effect of a walnut diet on office and 24-hour ambulatory blood pressure in elderly individuals: Findings from the WAHA randomized trial. *Hypertension*. 2019; 73: 1049-57.
410 Donnelly JE, Jacobsen DJ, Snyder Heelan K, Seip R, Smith S. The effects of 18 months of intermittent vs continuous exercise on aerobic capacity, body weight and composition, and metabolic fitness in previously sedentary, moderately obese females. *International Journal of Obesity*. 2000; 24: 566-72.
411 Dornelas EA, Wylie-Rosett J, Swencionis C. The DIET study: long-term outcomes of a cognitive-behavioral weight-control intervention in independent-living elders. Dietary Intervention: Evaluation of Technology. *Journal of the American Dietetic Association*. 1998; 98: 1276-81.
412 Drew RJ, Morgan PJ, Young MD. Mechanisms of an eHealth program targeting depression in men with overweight or obesity: A randomised trial. *Journal of Affective Disorders*. 2022; 299: 309-17.
413 Drummen M, Dorenbos E, Vreugdenhil ACE, Stratton G, Raben A, Westerterp-Plantenga MS*, et al.* Associations of Brain Reactivity to Food Cues with Weight Loss, Protein Intake and Dietary Restraint during the PREVIEW Intervention. *Nutrients*. 2018; 10: 15.
414 Dubbert PM, Wilson GT. Goal-setting and spouse involvement in the treatment of obesity. *Behaviour research and therapy*. 1984; 22: 227-42.
415 Dube JJ, Amati F, Toledo FG, Stefanovic-Racic M, Rossi A, Coen P*, et al.* Effects of weight loss and exercise on insulin resistance, and intramyocellular triacylglycerol, diacylglycerol and ceramide. *Diabetologia*. 2011; 54: 1147-56.
416 Duggan C, Tapsoba JD, Shivappa N, Harris HR, Hebert JR, Wang CY*, et al.* Changes in Dietary Inflammatory Index Patterns with Weight Loss in Women: A Randomized Controlled Trial. *Cancer Prevention Research*. 2021; 14: 85-94.
417 Dunn C, MacKenzie Whetstone L, Kolasa KM, Jayaratne KSU, Thomas C, Aggarwal S*, et al.* Using Synchronous Distance-Education Technology to Deliver a Weight Management Intervention. *Journal of Nutrition Education & Behavior*. 2014; 46: 602-09.
418 Dunn C, Olabode-Dada O, Whetstone L, Thomas C, Aggarwal S, Nordby K*, et al.* Using synchronous distance education to deliver a weight loss intervention: A randomized trial. *Obesity*. 2016; 24: 44-50.

419 Dunstan DW, Daly RM, Owen N, Jolley D, De Courten M, Shaw J, *et al.* High-intensity resistance training improves glycemic control in older patients with type 2 diabetes. *Diabetes Care*. 2002; 25: 1729-36.
420 Dunstan DW, Daly RM, Owen N, Jolley D, Vulikh E, Shaw J, *et al.* Home-based resistance training is not sufficient to maintain improved glycemic control following supervised training in older individuals with type 2 diabetes. *Diabetes Care*. 2005; 28: 3-9.
421 Dupuit M, Rance M, Morel C, Bouillon P, Boscaro A, Martin V, *et al.* Effect of Concurrent Training on Body Composition and Gut Microbiota in Postmenopausal Women with Overweight or Obesity. *Medicine & Science in Sports & Exercise*. 2022; 54: 517-29.
422 Dupuit M, Rance M, Morel C, Bouillon P, Pereira B, Bonnet A, *et al.* Moderate-Intensity Continuous Training or High-Intensity Interval Training with or without Resistance Training for Altering Body Composition in Postmenopausal Women. *Medicine & Science in Sports & Exercise*. 2020; 52: 736-45.
423 Eakin EG, Reeves MM, Winkler E, Healy GN, Dunstan DW, Owen N, *et al.* Six-month outcomes from living well with diabetes: A randomized trial of a telephone-delivered weight loss and physical activity intervention to improve glycemic control. *Annals of Behavioral Medicine*. 2013; 46: 193-203.
424 Eakin EG, Winkler EA, Dunstan DW, Healy GN, Owen N, Marshall AM, *et al.* Living well with diabetes: 24-month outcomes from a randomized trial of telephone-delivered weight loss and physical activity intervention to improve glycemic control. *Diabetes Care*. 2014; 37: 2177-85.
425 Egert S, Baxheinrich A, Lee-Barkey YH, Tschoepe D, Stehle P, Stratmann B, *et al.* Effects of a hypoenergetic diet rich in alpha-linolenic acid on fatty acid composition of serum phospholipids in overweight and obese patients with metabolic syndrome. *Nutrition*. 2018; 49: 74-80.
426 Egert S, Baxheinrich A, Lee-Barkey YH, Tschoepe D, Stehle P, Stratmann B, *et al.* Effects of a hypoenergetic diet rich in α-linolenic acid on fatty acid composition of serum phospholipids in overweight and obese patients with metabolic syndrome. *Nutrition (Burbank, Los Angeles County, Calif)*. 2018; 49: 74-80.
427 Eguaras S, Toledo E, Buil-Cosiales P, Salas-Salvado J, Corella D, Gutierrez-Bedmar M, *et al.* Does the Mediterranean diet counteract the adverse effects of abdominal adiposity? *Nutrition Metabolism & Cardiovascular Diseases*. 2015; 25: 569-74.
428 Elder C, Ritenbaugh C, Mist S, Aickin M, Schneider J, Zwickey H, *et al.* Randomized trial of two mind-body interventions for weight-loss maintenance. *The Journal of Alternative and Complementary Medicine*. 2007; 13: 67-78.
429 Ello-Martin JA, Roe LS, Ledikwe JH, Beach AM, Rolls BJ. Dietary energy density in the treatment of obesity: a year-long trial comparing 2 weight-loss diets. *American Journal of Clinical Nutrition*. 2007; 85: 1465-77.
430 Ely AC, Banitt A, Befort C, Hou Q, Rhode PC, Grund C, *et al.* Kansas primary care weighs in: a pilot randomized trial of a chronic care model program for obesity in 3 rural Kansas primary care practices. *Journal of Rural Health*. 2008; 24: 125-32.
431 Emerenziani GP, Gallotta MC, Meucci M, Di Luigi L, Migliaccio S, Donini LM, *et al.* Effects of Aerobic Exercise Based upon Heart Rate at Aerobic Threshold in Obese Elderly Subjects with Type 2 Diabetes. *International Journal of Endocrinology Print*. 2015; 2015: 695297.
432 Epstein DE, Sherwood A, Smith PJ, Craighead L, Caccia C, Lin PH, *et al.* Determinants and consequences of adherence to the dietary approaches to stop hypertension diet in

African-American and white adults with high blood pressure: results from the ENCORE trial. *Journal of the academy of nutrition and dietetics*. 2012; 112: 1763-73.
433 Erez G, Tirosh A, Rudich A, Meiner V, Schwarzfuchs D, Sharon N*, et al.* Phenotypic and genetic variation in leptin as determinants of weight regain. *International Journal of Obesity*. 2011; 35: 785-92.
434 Erickson ZD, Kwan CL, Gelberg HA, Arnold IY, Chamberlin V, Rosen JA*, et al.* A Randomized, Controlled Multisite Study of Behavioral Interventions for Veterans with Mental Illness and Antipsychotic Medication-Associated Obesity. *Journal of General Internal Medicine*. 2017; 32: 32-39.
435 Espeland MA, Dutton GR, Neiberg RH, Carmichael O, Hayden KM, Johnson KC*, et al.* Impact of a multidomain intensive lifestyle intervention on complaints about memory, problem-solving, and decision-making abilities: The Action for Health in Diabetes randomized controlled clinical trial. *The Journals of Gerontology: Series A: Biological Sciences and Medical Sciences*. 2018; 73: 1560-67.
436 Espeland MA, Glick HA, Bertoni A, Brancati FL, Bray GA, Clark JM*, et al.* Impact of an intensive lifestyle intervention on use and cost of medical services among overweight and obese adults with type 2 diabetes: the action for health in diabetes. *Diabetes Care*. 2014; 37: 2548-56.
437 Espeland MA, Lewis CE, Bahnson J, Knowler WC, Regensteiner JG, Gaussoin SA*, et al.* Impact of weight loss on ankle-brachial index and interartery blood pressures. *Obesity*. 2014; 22: 1032-41.
438 Espeland MA, Rejeski WJ, West DS, Bray GA, Clark JM, Peters AL*, et al.* Intensive weight loss intervention in older individuals: results from the Action for Health in Diabetes Type 2 diabetes mellitus trial. *Journal of the American Geriatrics Society*. 2013; 61: 912-22.
439 Esposito K, Di Palo C, Maiorino MI, Petrizzo M, Bellastella G, Siniscalchi I*, et al.* Long-term effect of mediterranean-style diet and calorie restriction on biomarkers of longevity and oxidative stress in overweight men. *Cardiology Research & Practice*. 2010; 2011: 293916.
440 Evans EM, Straight CR, Reed RA, Berg AC, Rowe DA, Johnson MA. Exercise and Protein Effects on Strength and Function with Weight Loss in Older Women. *Medicine & Science in Sports & Exercise*. 2021; 53: 183-91.
441 Evans EM, Van Pelt RE, Binder EF, Williams DB, Ehsani AA, Kohrt WM. Effects of HRT and exercise training on insulin action, glucose tolerance, and body composition in older women. *Journal of Applied Physiology*. 2001; 90: 2033-40.
442 Evans WE, Raynor HA, Howie W, Lipton RB, Thomas GJ, Wing RR*, et al.* Associations between lifestyle intervention-related changes in dietary targets and migraine headaches among women in the Women's Health and Migraine (WHAM) randomized controlled trial. *Obesity science and practice*. 2019.
443 Ewert CP, Campos S, Medina M, Bustamante C, Urrutia M, Sánchez H*, et al.* Evaluación del Modelo multicomponente de telecuidado de apoyo a personas con prediabetes y obesidad en Chile = Evaluation of a multicomponent tele-care model for supporting people with prediabetes and obesity in Chile. *Actualidades en Psicología*. 2016; 30: 103-17.
444 Fabricatore AN, Wadden TA, Ebbeling CB, Thomas JG, Stallings VA, Schwartz S*, et al.* Targeting dietary fat or glycemic load in the treatment of obesity and type 2 diabetes: a randomized controlled trial. *Diabetes Research & Clinical Practice*. 2011; 92: 37-45.
445 Fanning J, Walkup MP, Ambrosius WT, Brawley LR, Ip EH, Marsh AP*, et al.* Change in health-related quality of life and social cognitive outcomes in obese, older adults in a

randomized controlled weight loss trial: Does physical activity behavior matter? *Journal of Behavioral Medicine*. 2018; 41: 299-308.
446 Farnsworth E, Luscombe ND, Noakes M, Wittert G, Argyiou E, Clifton PM. Effect of a high-protein, energy-restricted diet on body composition, glycemic control, and lipid concentrations in overweight and obese hyperinsulinemic men and women. *American Journal of Clinical Nutrition*. 2003; 78: 31-39.
447 Farsijani S, Cauley JA, Santanasto AJ, Glynn NW, Boudreau RM, Newman AB. Transition to a More even Distribution of Daily Protein intake Is Associated with Enhanced Fat Loss during a Hypocaloric and Hhysical Activity Intervention in Obese Older Adults. *Journal of Nutrition, Health & Aging*. 2020; 24: 210-17.
448 Fatouros IG, Chatzinikolaou A, Tournis S, Nikolaidis MG, Jamurtas AZ, Douroudos, II*, et al.* Intensity of resistance exercise determines adipokine and resting energy expenditure responses in overweight elderly individuals. *Diabetes Care*. 2009; 32: 2161-67.
449 Fatouros IG, Tournis S, Leontsini D, Jamurtas AZ, Sxina M, Thomakos P*, et al.* Leptin and adiponectin responses in overweight inactive elderly following resistance training and detraining are intensity related. *Journal of Clinical Endocrinology & Metabolism*. 2005; 90: 5970-77.
450 Faulconbridge LF, Driscoll CFB, Hopkins CM, Bailer Benforado B, Bishop-Gilyard C, Carvajal R*, et al.* Combined Treatment for Obesity and Depression: A Pilot Study. *Obesity*. 2018; 26: 1144-52.
451 Faulconbridge LF, Wadden TA, Rubin RR, Wing RR, Walkup MP, Fabricatore AN*, et al.* One-year changes in symptoms of depression and weight in overweight/obese individuals with type 2 diabetes in the Look AHEAD study. *Obesity*. 2012; 20: 783-93.
452 Fava F, Gitau R, Griffin BA, Gibson GR, Tuohy KM, Lovegrove JA. The type and quantity of dietary fat and carbohydrate alter faecal microbiome and short-chain fatty acid excretion in a metabolic syndrome 'at-risk' population. *International Journal of Obesity*. 2013; 37: 216-23.
453 Fernandez-Garcia JC, Martinez-Sanchez MA, Bernal-Lopez MR, Munoz-Garach A, Martinez-Gonzalez MA, Fito M*, et al.* Effect of a lifestyle intervention program with energy-restricted Mediterranean diet and exercise on the serum polyamine metabolome in individuals at high cardiovascular disease risk: a randomized clinical trial. *American Journal of Clinical Nutrition*. 2020; 111: 975-82.
454 Fernández-Ruiz VE, Armero-Barranco D, Paniagua-Urbano JA, Sole-Agusti M, Ruiz-Sánchez A, Gómez-Marín J. Short-medium-long-term efficacy of interdisciplinary intervention against overweight and obesity: randomized controlled clinical trial. *International journal of nursing practice*. 2018; 24: e12690.
455 Fernandez-Ruiz VE, Paniagua-Urbano JA, Sole-Agusti M, Ruiz-Sanchez A, Gomez-Marin J, Armero-Barranco D. Impact of the I2AO2 interdisciplinary program led by nursing on psychological comorbidity and quality of life: randomized controlled clinical trial. *Archives of psychiatric nursing*. 2018; 32: 268-77.
456 Fernandez-Ruiz VE, Paniagua-Urbano JA, Sole-Agusti M, Ruiz-Sanchez A, Gomez-Marin J, Armero-Barranco D. Effectiveness of the I²AO² interdisciplinary programme led by nurses on metabolic syndrome and cardiovascular risk: a randomized, controlled trial. *Journal of International Medical Research*. 2018; 46: 2202-18.
457 Ferrara CM, McCrone SH, Brendle D, Ryan AS, Goldberg AP. Metabolic effects of the addition of resistive to aerobic exercise in older men. *International Journal of Sport Nutrition & Exercise Metabolism*. 2004; 14: 73-80.

458 Ferre R, Plana N, Merino J, Aragones G, Girona J, Heras M*, et al.* Effects of therapeutic lifestyle changes on peripheral artery tonometry in patients with abdominal obesity. *Nutrition Metabolism & Cardiovascular Diseases*. 2012; 22: 95-102.
459 Feuerstein M, Papciak A, Shapiro S, Tannenbaum S. The Weight Loss Profile: A biopsychosocial approach to weight loss. *International Journal of Psychiatry in Medicine*. 1989; 19: 181-92.
460 Finch EA, Linde JA, Jeffery RW, Rothman AJ, King CM, Levy RL. The effects of outcome expectations and satisfaction on weight loss and maintenance: correlational and experimental analyses--a randomized trial. *Health Psychology*. 2005; 24: 608-16.
461 Fisher A, Craigie AM, Macleod M, Steele RJC, Anderson AS. The impact of social deprivation on the response to a randomised controlled trial of a weight management intervention (BeWEL) for people at increased risk of colorectal cancer. *Journal of Human Nutrition & Dietetics*. 2018; 31: 306-13.
462 Fitzgibbon ML, Stolley MR, Ganschow P, Schiffer L, Wells A, Simon N*, et al.* Results of a faith-based weight loss intervention for black women. *Journal of the National Medical Association*. 2005; 97: 1393-402.
463 Fitzgibbon ML, Stolley MR, Schiffer L, Sharp LK, Singh V, Dyer A. Obesity Reduction Black Intervention Trial (ORBIT): 18-month results. *Obesity*. 2010; 18: 2317-25.
464 Fitzpatrick SL, Bandeen-Roche K, Stevens VJ, Coughlin JW, Rubin RR, Brantley PJ*, et al.* Examining behavioral processes through which lifestyle interventions promote weight loss: Results from PREMIER. *Obesity*. 2014; 22: 1002-07.
465 Flack KD, Hays HM, Moreland J. The consequences of exercise-induced weight loss on food reinforcement. A randomized controlled trial. *PLoS ONE*. 2020; 15: 17.
466 Flechtner-Mors M, Boehm BO, Wittmann R, Thoma U, Ditschuneit HH. Enhanced weight loss with protein-enriched meal replacements in subjects with the metabolic syndrome. *Diabetes/Metabolism Research Reviews*. 2010; 26: 393-405.
467 Fleming RM. The effect of high-, moderate-, and low-fat diets on weight loss and cardiovascular disease risk factors. *Preventive cardiology*. 2002; 5: 110-18.
468 Flood A, Mitchell N, Jaeb M, Finch EA, Laqua PS, Welsh EM*, et al.* Energy density and weight change in a long-term weight-loss trial. *The International Journal of Behavioral Nutrition and Physical Activity*. 2009; 6.
469 Focht BC, Rejeski WJ, Ambrosius WT, Katula JA, Messier SP. Exercise, self-efficacy, and mobility performance in overweight and obese older adults with knee osteoarthritis. *Arthritis and rheumatism*. 2005; 53: 659-65.
470 Fordyce-Baum MK, Langer LM, Mantero-Atienza E, Crass R, Beach RS. Use of an expanded-whole-wheat product in the reduction of body weight and serum lipids in obese females. *American Journal of Clinical Nutrition*. 1989; 50: 30-36.
471 Foreyt JP, Goodrick GK, Reeves RS, Raynaud AS, Darnell L, Brown AH*, et al.* Response of free-living adults to behavioral treatment of obesity: Attrition and compliance to exercise. *Behavior Therapy*. 1993; 24: 659-69.
472 Forman EM, Butryn ML, Juarascio AS, Bradley LE, Lowe MR, Herbert JD*, et al.* The mind your health project: a randomized controlled trial of an innovative behavioral treatment for obesity. *Obesity*. 2013; 21: 1119-26.
473 Forman EM, Butryn ML, Manasse SM, Crosby RD, Goldstein SP, Wyckoff EP*, et al.* Acceptance-based versus standard behavioral treatment for obesity: Results from the mind your health randomized controlled trial. *Obesity*. 2016; 24: 2050-56.
474 Forman EM, Kerrigan SG, Butryn ML, Juarascio AS, Manasse SM, Ontañón S*, et al.* Can the artificial intelligence technique of reinforcement learning use continuously-monitored

digital data to optimize treatment for weight loss? *Journal of Behavioral Medicine*. 2019; 42: 276-90.
475 Forman EM, Manasse SM, Butryn ML, Crosby RD, Dallal DH, Crochiere RJ. Long-Term Follow-up of the Mind Your Health Project: Acceptance-Based versus Standard Behavioral Treatment for Obesity. *Obesity*. 2019; 27: 565-71.
476 Forrest LN, Grilo CM. Change in eating-disorder psychopathology network structure in patients with binge-eating disorder: Findings from treatment trial with 12-month follow-up. *Journal of Consulting and Clinical Psychology*. 2022.
477 Fossati M, Amati F, Painot D, Reiner M, Haenni C, Golay A. Cognitive-behavioral therapy with simultaneous nutritional and physical activity education in obese patients with binge eating disorder. *Eating and weight disorders*. 2004; 9: 134-38.
478 Foster GD, Wyatt HR, Hill JO, McGuckin BG, Brill C, Mohammed BS*, et al.* A randomized trial of a low-carbohydrate diet for obesity. *New England Journal of Medicine*. 2003; 348: 2082-90.
479 Fox AA, Thompson JL, Butterfield GE, Gylfadottir U, Moynihan S, Spiller G. Effects of diet and exercise on common cardiovascular disease risk factors in moderately obese older women. *American Journal of Clinical Nutrition*. 1996; 63: 225-33.
480 Foy CG, Lewis CE, Hairston KG, Miller GD, Lang W, Jakicic JM*, et al.* Intensive lifestyle intervention improves physical function among obese adults with knee pain: findings from the Look AHEAD trial. *Obesity*. 2011; 19: 83-93.
481 Francis SL, Simmering JE, Polgreen LA, Evans NJ, Hosteng KR, Carr LJ*, et al.* Gamifying accelerometer use increases physical activity levels of individuals pre-disposed to type II diabetes. *Preventive medicine reports*. 2021; 23.
482 Frank E, Wallace ML, Hall M, Hasler B, Levenson JC, Janney CA*, et al.* An integrated risk reduction intervention can reduce body mass index in individuals being treated for bipolar I disorder: Results from a randomized trial. *Bipolar Disorders*. 2015; 17: 424-37.
483 Frankwich KA, Egnatios J, Kenyon ML, Rutledge TR, Liao PS, Gupta S*, et al.* Differences in Weight Loss Between Persons on Standard Balanced vs Nutrigenetic Diets in a Randomized Controlled Trial. *Clinical gastroenterology and hepatology*. 2015; 13: 1625-32.
484 Fraser A, Abel R, Lawlor DA, Fraser D, Elhayany A. A modified Mediterranean diet is associated with the greatest reduction in alanine aminotransferase levels in obese type 2 diabetes patients: results of a quasi-randomised controlled trial. *Diabetologia*. 2008; 51: 1616-22.
485 Freer CL, George ES, Tan SY, Abbott G, Dunstan DW, Daly RM. Effect of progressive resistance training with weight loss compared with weight loss alone on the fatty liver index in older adults with type 2 diabetes: secondary analysis of a 12-month randomized controlled trial. *BMJ Open Diabetes Research & Care*. 2022; 10: 10.
486 Freitas PD, Silva AG, Ferreira PG, Da Silva A, Salge JM, Carvalho-Pinto RM*, et al.* Exercise Improves Physical Activity and Comorbidities in Obese Adults with Asthma. *Medicine and science in sports and exercise*. 2018; 50: 1367-76.
487 Freitas-Simoes T-M, Cofán M, Blasco MA, Soberón N, Foronda M, Serra-Mir M*, et al.* Walnut Consumption for Two Years and Leukocyte Telomere Attrition in Mediterranean Elders: Results of a Randomized Controlled Trial. *Nutrients*. 2018; 10: 1907.
488 Friedman AN, Ogden LG, Foster GD, Klein S, Stein R, Miller B*, et al.* Comparative effects of low-carbohydrate high-protein versus low-fat diets on the kidney. *Clinical journal of the American Society of Nephrology*. 2012; 7: 1103-11.
489 Frisch S, Zittermann A, Berthold HK, Gotting C, Kuhn J, Kleesiek K*, et al.* A randomized controlled trial on the efficacy of carbohydrate-reduced or fat-reduced diets in patients

attending a telemedically guided weight loss program. *Cardiovascular Diabetology*. 2009; 8 (no pagination).
490 Frost G, Masters K, King C, Kelly M, Hasan U, Heavens P*, et al.* A new method of energy prescription to improve weight loss...Reprinted from Journal of Human Nutrition and Dietetics (1991),4;369-373. *Journal of Human Nutrition & Dietetics*. 2007; 20: 152-56.
491 Fuglsang-Nielsen R, Rakvaag E, Langdahl B, Knudsen KEB, Hartmann B, Holst JJ*, et al.* Effects of whey protein and dietary fiber intake on insulin sensitivity, body composition, energy expenditure, blood pressure, and appetite in subjects with abdominal obesity. *European Journal of Clinical Nutrition*. 2021; 75: 611-19.
492 Fuller NR, Caterson ID, Sainsbury A, Denyer G, Fong M, Gerofi J*, et al.* The effect of a high-egg diet on cardiovascular risk factors in people with type 2 diabetes: the Diabetes and Egg (DIABEGG) study-a 3-mo randomized controlled trial. *American Journal of Clinical Nutrition*. 2015; 101: 705-13.
493 Fuller NR, Lau NS, Denyer G, Simpson AE, Gerofi J, Wu M*, et al.* A 12-week, randomised, controlled trial to examine the acceptability of the Korean diet and its effectiveness on weight and metabolic parameters in an Australian overweight and obese population. *Obesity Research & Clinical Practice*. 2012; 6: e71-e83.
494 Fuller NR, Sainsbury A, Caterson ID, Denyer G, Fong M, Gerofi J*, et al.* Effect of a high-egg diet on cardiometabolic risk factors in people with type 2 diabetes: the Diabetes and Egg (DIABEGG) Study-randomized weight-loss and follow-up phase. *American Journal of Clinical Nutrition*. 2018; 107: 921-31.
495 Fuller NR, Williams K, Shrestha R, Ahern AL, Holzapfel C, Hauner H*, et al.* Changes in physical activity during a weight loss intervention and follow-up: a randomized controlled trial. *Clinical Obesity*. 2014; 4: 127-35.
496 Gabel K, Kroeger CM, Trepanowski JF, Hoddy KK, Cienfuegos S, Kalam F*, et al.* Differential Effects of Alternate-Day Fasting Versus Daily Calorie Restriction on Insulin Resistance. *Obesity (19307381)*. 2019; 27: 1443-50.
497 Gabriele JM, Carpenter BD, Tate DF, Fisher EB. Directive and nondirective e-coach support for weight loss in overweight adults. *Annals of Behavioral Medicine*. 2011; 41: 252-63.
498 Gadelha AB, Paiva FM, Gauche R, de Oliveira RJ, Lima RM. Effects of resistance training on sarcopenic obesity index in older women: A randomized controlled trial. *Archives of Gerontology & Geriatrics*. 2016; 65: 168-73.
499 Gagnon C, Brown C, Couture C, Kamga-Ngande CN, Hivert MF, Baillargeon JP*, et al.* A cost-effective moderate-intensity interdisciplinary weight-management programme for individuals with prediabetes. *Diabetes & Metabolism*. 2011; 37: 410-18.
500 Gajewska D, Kucharska A, Kozak M, Wunderlich S, Niegowska J. Effectiveness of individual nutrition education compared to group education, in improving anthropometric and biochemical indices among hypertensive adults with excessive body weight: a randomized controlled trial. *Nutrients*. 2019; 11.
501 Galbreath M, Campbell B, La Bounty P, Bunn J, Dove J, Harvey T*, et al.* Effects of Adherence to a Higher Protein Diet on Weight Loss, Markers of Health, and Functional Capacity in Older Women Participating in a Resistance-Based Exercise Program. *Nutrients*. 2018; 10: 1070.
502 Galindo Munoz JS, Gomez Gallego M, Diaz Soler I, Barbera Ortega MC, Martinez Caceres CM, Hernandez Morante JJ. Effect of a chronotype-adjusted diet on weight loss effectiveness: A randomized clinical trial. *Clinical Nutrition*. 2020; 39: 1041-48.

503 Gallagher R, Zelestis E, Hollams D, Denney-Wilson E, Kirkness A. Impact of the healthy eating and exercise lifestyle programme on depressive symptoms in overweight people with heart disease and diabetes. *European Journal of Preventive Cardiology*. 2014; 21: 1117-24.
504 Gamiochipi M, Cruz M, Kumate J, Wacher NH. Effect of an intensive metabolic control lifestyle intervention in type-2 diabetes patients. *Patient education and counseling*. 2016; 99: 1184-89.
505 Gandler N, Simmance N, Keenan J, Choong PF, Dowsey MM. A pilot study investigating dietetic weight loss interventions and 12 month functional outcomes of patients undergoing total joint replacement. *Obesity Research & Clinical Practice*. 2016; 10: 220-23.
506 Garavelo JJ, Altimari LR, Gabardo JM, Ferreira-Junior A, Freitas LAG, Buzzachera CF*, et al.* Effects of walk training with self-selected intensity on biochemical markers and anthropometric variables in women with obesity. *Obesity Research & Clinical Practice*. 2019; 13: 211-13.
507 Garcia-Arellano A, Ramallal R, Ruiz-Canela M, Salas-Salvadó J, Corella D, Shivappa N*, et al.* Dietary Inflammatory Index and Incidence of Cardiovascular Disease in the PREDIMED Study. *Nutrients*. 2015; 7: 4124-38.
508 Gardner CD, Offringa LC, Hartle JC, Kapphahn K, Cherin R. Weight loss on low-fat vs low-carbohydrate diets by insulin resistance status among overweight adults and adults with obesity: A randomized pilot trial. *Obesity*. 2016; 24: 79-86.
509 Garvin JT, Williams LB, Joshua TV, Looney SW, Marion LN. Percent weight reduction required to achieve minimal clinically important improvements in health-related quality of life among African Americans: A secondary analysis of the fit body and soul study. *Applied Nursing Research*. 2017; 36: 100-05.
510 Gelo OC, Zips A, Ponocny-Seliger E, Neumann K, Balugani R, Gold C. Hypnobehavioral and hypnoenergetic therapy in the treatment of obese women: a pragmatic randomized clinical trial. *International Journal of Clinical & Experimental Hypnosis*. 2014; 62: 260-91.
511 Georgiades A, Sherwood A, Gullette EC, Babyak MA, Hinderliter A, Waugh R*, et al.* Effects of exercise and weight loss on mental stress-induced cardiovascular responses in individuals with high blood pressure. *Hypertension (dallas, tex : 1979)*. 2000; 36: 171-76.
512 Gepner Y, Shelef I, Komy O, Cohen N, Schwarzfuchs D, Bril N*, et al.* The beneficial effects of Mediterranean diet over low-fat diet may be mediated by decreasing hepatic fat content. *Journal of Hepatology*. 2019; 71: 379-88.
513 Gerber BS, Schiffer L, Brown AA, Berbaum ML, Rimmer JH, Braunschweig CL*, et al.* Video telehealth for weight maintenance of African-American women. *Journal of Telemedicine & Telecare*. 2013; 19: 266-72.
514 Ghasemi E, Cheraghbirjandi K. Investigating the Effect of a Multicomponent Exercise Program on Adropin, Endothelial Function, Insulin Resistance, and Sleep Quality in Overweight Older Adults (a Link With Physiological Indexes and Sleep Quality): Results of a Randomized Controlled Study. *Journal of Aging & Physical Activity*. 2024: 1-7.
515 Ghroubi S, Elleuch H, Kaffel N, Echikh T, Abid M, Elleuch MH. Contribution of exercise and diet in the management of knee osteoarthritis in the obese. *Annales de readaptation et de medecine physique*. 2008; 51: 663-70.
516 Giaccaglia V, Nicklas B, Kritchevsky S, Mychalecky J, Messier S, Bleecker E*, et al.* Interaction between angiotensin converting enzyme insertion/deletion genotype and exercise training on knee extensor strength in older individuals. *International Journal of Sports Medicine*. 2008; 29: 40-44.

517 Giardina S, Sala-Vila A, Hernández-Alonso P, Calvo C, Salas-Salvadó J, Bulló M. Carbohydrate quality and quantity affects the composition of the red blood cell fatty acid membrane in overweight and obese individuals. *Clinical nutrition (Edinburgh, Scotland)*. 2018; 37: 481-87.
518 Gibbons C, Blundell JE, Caudwell P, Webb DL, Hellström PM, Näslund E*, et al.* The Role of Episodic Postprandial Peptides in Exercise-Induced Compensatory Eating. *Journal of clinical endocrinology and metabolism*. 2017; 102: 4051-59.
519 Gilbert S, Garrow JS. A prospective controlled trial of outpatient treatment for obesity. *Human nutrition Clinical nutrition*. 1983; 37: 21-29.
520 Gillison F, Stathi A, Reddy P, Perry R, Taylor G, Bennett P*, et al.* Processes of behavior change and weight loss in a theory-based weight loss intervention program: a test of the process model for lifestyle behavior change. *International Journal of Behavioral Nutrition & Physical Activity*. 2015; 12: 2.
521 Gocer E, Ardic F, Akkaya N, Herek D. Efficacy of moderate-intensity walking provided feedback by ECE PEDO on abdominal fat in overweight and obese women: a randomized, exercise study. *Turkiye fiziksel tip ve rehabilitasyon dergisi*. 2017; 63: 340-47.
522 Godfrey KM, Schumacher LM, Butryn ML, Forman EM. Physical activity intentions and behavior mediate treatment response in an acceptance-based weight loss intervention. *Annals of Behavioral Medicine*. 2019; 53: 1009-19.
523 Godziuk K, Prado CM, Forhan M. Protocol for the POMELO (Prevention Of MusclE Loss in Osteoarthritis) randomized pilot feasibility trial. *Osteoarthritis and Cartilage Open*. 2022; 4: 100312.
524 Göhner W, Schlatterer M, Seelig H, Frey I, Berg A, Fuchs R. Two-year follow-up of an interdisciplinary cognitive-behavioral intervention program for obese adults. *The Journal of Psychology: Interdisciplinary and Applied*. 2012; 146: 371-91.
525 Gokulakrishnan K, Ranjani H, Weber MB, Pandey GK, Anjana RM, Balasubramanyam M*, et al.* Effect of lifestyle improvement program on the biomarkers of adiposity, inflammation and gut hormones in overweight/obese Asian Indians with prediabetes. *Acta Diabetologica*. 2017; 54: 843-52.
526 Goldbacher E, La Grotte C, Komaroff E, Veur SV, Foster GD. An initial evaluation of a weight loss intervention for individuals who engage in emotional eating. *Journal of Behavioral Medicine*. 2016; 39: 139-50.
527 Goldberg RW, Reeves G, Tapscott S, Medoff D, Dickerson F, Goldberg AP*, et al.* 'MOVE!': Outcomes of a weight loss program modified for veterans with serious mental illness. *Psychiatric Services*. 2013; 64: 737-44.
528 Gomez-Marcos MA, Patino-Alonso MC, Recio-Rodriguez JI, Agudo-Conde C, Romaguera-Bosch M, Magdalena-Gonzalez O*, et al.* Short- and long-term effectiveness of a smartphone application for improving measures of adiposity: a randomised clinical trial - EVIDENT II study. *European Journal of Cardiovascular Nursing*. 2018; 17: 552-62.
529 Goni L, Riezu-Boj JI, Milagro FI, Corrales FJ, Ortiz L, Cuervo M*, et al.* Interaction between an ADCY3 Genetic Variant and Two Weight-Lowering Diets Affecting Body Fatness and Body Composition Outcomes Depending on Macronutrient Distribution: a Randomized Trial. *Nutrients*. 2018; 10.
530 Gonzalo-Encabo P, McNeil J, Perez-Lopez A, Valades D, Courneya KS, Friedenreich CM. Dose-response effects of aerobic exercise on adiposity markers in postmenopausal women: pooled analyses from two randomized controlled trials. *International Journal of Obesity*. 2021; 45: 1298-309.

531 Goodpaster BH, DeLany JP, Otto AD, Kuller L, Vockley J, South-Paul JE*, et al.* Effects of diet and physical activity interventions on weight loss and cardiometabolic risk factors in severely obese adults: A randomized trial. *JAMA: Journal of the American Medical Association*. 2010; 304: 1795-18020.
532 Goodrick GK, Poston WSC, II, Kimball KT, Reeves RS, Foreyt JP. Nondieting versus dieting treatment for overweight binge-eating women. *Journal of consulting and clinical psychology*. 1998; 66: 363-68.
533 Goodwin PJ, Segal RJ, Vallis M, Ligibel JA, Pond GR, Robidoux A*, et al.* The LISA randomized trial of a weight loss intervention in postmenopausal breast cancer. *NPJ Breast Cancer*. 2020; 6.
534 Gordon EL, Merlo LJ, Durning PE, Perri MG. Longitudinal Changes in Food Addiction Symptoms and Body Weight among Adults in a Behavioral Weight-Loss Program. *Nutrients*. 2020; 12: 29.
535 Gordon NF, Scott CB, Levine BD. Comparison of single versus multiple lifestyle interventions: are the antihypertensive effects of exercise training and diet-induced weight loss additive? *American Journal of Cardiology*. 1997; 79: 763-67.
536 Gorgey AS, Mather KJ, Cupp HR, Gater DR. Effects of resistance training on adiposity and metabolism after spinal cord injury. *Medicine and science in sports and exercise*. 2012; 44: 165-74.
537 Gorin AA, Le Grange D, Stone AA. Effectiveness of spouse involvement in cognitive behavioral therapy for binge eating disorder. *International Journal of Eating Disorders*. 2003; 33: 421-33.
538 Gorin AA, Powers TA, Gettens K, Cornelius T, Koestner R, Mobley AR*, et al.* A randomized controlled trial of a theory-based weight-loss program for couples. *Health Psychology*. 2020; 39: 137-46.
539 Goyenechea E, Holst C, van Baak MA, Saris WH, Jebb S, Kafatos A*, et al.* Effects of different protein content and glycaemic index of ad libitum diets on diabetes risk factors in overweight adults: the DIOGenes multicentre, randomized, dietary intervention trial. *Diabetes/metabolism research and reviews*. 2011; 27: 705-16.
540 Gozukara YM, Akalan G, Tok EC, Aytan H, Ertunc D. The improvement in pelvic floor symptoms with weight loss in obese women does not correlate with the changes in pelvic anatomy. *International Urogynecology Journal*. 2014; 25: 1219-25.
541 Grajek M, Gdanska A, Krupa-Kotara K, Glogowska-Ligus J, Kobza J. Global Self-Esteem, Physical Activity, and Body Composition Changes Following a 12-Week Dietary and Physical Activity Intervention in Older Women. *International Journal of Environmental Research & Public Health [Electronic Resource]*. 2022; 19: 14.
542 Green CA, Janoff SL, Yarborough BJ, Yarborough MT. A 12-week weight reduction intervention for overweight individuals taking antipsychotic medications. *Community mental health journal*. 2014; 50: 974-80.
543 Green CA, Yarborough BJH, Leo MC, Stumbo SP, Perrin NA, Nichols GA*, et al.* Weight maintenance following the STRIDE lifestyle intervention for individuals taking antipsychotic medications. *Obesity*. 2015; 23: 1995-2001.
544 Gregg EW, Chen H, Wagenknecht LE, Clark JM, Delahanty LM, Bantle J*, et al.* Association of an intensive lifestyle intervention with remission of type 2 diabetes. *Jama*. 2012; 308: 2489-96.
545 Gretebeck KA, Blaum CS, Moore T, Brown R, Galecki A, Strasburg D*, et al.* Functional Exercise Improves Mobility Performance in Older Adults With Type 2 Diabetes: A Randomized Controlled Trial. *Journal of Physical Activity & Health*. 2019; 16: 461-69.

546 Grilo CM, Gueorguieva R, Pittman B. Examining depression scores as predictors and moderators of treatment outcomes in patients with binge-eating disorder. *International Journal of Eating Disorders*. 2021; 54: 1555-59.
547 Grilo CM, Masheb RM. A randomized controlled comparison of guided self-help cognitive behavioral therapy and behavioral weight loss for binge eating disorder. *Behaviour research and therapy*. 2005; 43: 1509-25.
548 Grilo CM, Masheb RM, Wilson GT, Gueorguieva R, White MA. Cognitive–behavioral therapy, behavioral weight loss, and sequential treatment for obese patients with binge-eating disorder: A randomized controlled trial. *Journal of consulting and clinical psychology*. 2011; 79: 675-85.
549 Grilo CM, White MA, Gueorguieva R, Barnes RD, Masheb RM. Self-help for binge eating disorder in primary care: a randomized controlled trial with ethnically and racially diverse obese patients. *Behaviour research and therapy*. 2013; 51: 855-61.
550 Guasch-Ferré M, Hu FB, Ruiz-Canela M, Bulló M, Toledo E, Wang DD*, et al.* Plasma Metabolites From Choline Pathway and Risk of Cardiovascular Disease in the PREDIMED (Prevention With Mediterranean Diet) Study. *Journal of the American Heart Association*. 2017; 6: 1-N.PAG.
551 Gulati S, Misra A, Tiwari R, Sharma M, Pandey RM, Yadav CP. Effect of high-protein meal replacement on weight and cardiometabolic profile in overweight/obese Asian Indians in North India. *British Journal of Nutrition*. 2017; 117: 1531-40.
552 Gurka MJ, Wolf AM, Conaway MR, Crowther JQ, Nadler JL, Bovbjerg VE. Lifestyle intervention in obese patients with type 2 diabetes: Impact of the patient's educational background. *Obesity*. 2006; 14: 1085-92.
553 Hageman PA, Pullen CH, Hertzog M, Pozehl B, Eisenhauer C, Boeckner LS. Web-Based Interventions Alone or Supplemented with Peer-Led Support or Professional Email Counseling for Weight Loss and Weight Maintenance in Women from Rural Communities: Results of a Clinical Trial. *Journal of Obesity*. 2017; 2017: 1602627.
554 Haggerty AF, Hagemann A, Barnett M, Thornquist M, Neuhouser ML, Horowitz N*, et al.* A randomized, controlled, multicenter study of technology-based weight loss interventions among endometrial cancer survivors. *Obesity*. 2017; 25: S102-S08.
555 Hales S, Turner-McGrievy GM, Wilcox S, Fahim A, Davis RE, Huhns M*, et al.* Social networks for improving healthy weight loss behaviors for overweight and obese adults: a randomized clinical trial of the social pounds off digitally (Social POD) mobile app. *International journal of medical informatics*. 2016; 94: 81-90.
556 Hall KS, Gregg J, Bosworth HB, Beckham JC, Hoerster KD, Sloane R*, et al.* Physical Activity Counseling Promotes Physical and Psychological Resilience in Older Veterans with Posttraumatic Stress Disorder. *Mental Health & Physical Activity*. 2016; 11: 53-59.
557 Hall M, Bennell KL, Beavers DP, Wrigley TV, DeVita P, Messier SP. Does frontal knee kinematics predict treatment outcomes? Exploratory analyses from the Intensive Diet and Exercise for Arthritis (IDEA) trial. *Gait & Posture*. 2018; 63: 139-44.
558 Hall SM, Hall RG, Borden BL, Hanson RW. Follow-up strategies in the behavioral treatment of overweight. *Behaviour research and therapy*. 1975; 13: 167-72.
559 Hämäläinen H, Rönnemaa T, Virtanen A, Lindström J, Eriksson JG, Valle TT*, et al.* Improved fibrinolysis by an intensive lifestyle intervention in subjects with impaired glucose tolerance. The Finnish Diabetes Prevention Study. *Diabetologia*. 2005; 48: 2248-53.
560 Hammami N, Mechraoui A, Hattabi S, Forte P, Sampaio T, Sortwell A*, et al.* Concentric Isokinetic Strengthening Program's Impact on Knee Biomechanical Parameters, Physical

Performance and Quality of Life in Overweight/Obese Women with Chronic Meniscal Lesions. *Healthcare*. 2023; 11: 20.
561 Han CJ, Korde LA, Reding S, Allott K, Van Doren M, Schwarz Y*, et al.* Investigation of a Lifestyle Intervention in Women at High Risk of Breast Cancer. *Western Journal of Nursing Research*. 2018; 40: 976-96.
562 Han Y, Kim AR, Lee JH, Kim M. Adiponectin and 8-epi-PGF2α as intermediate influencing factors in weight reduction after legume consumption: a 12-week randomised controlled trial. *British Journal of Nutrition*. 2022; 127: 257-65.
563 Hansen E, Landstad BJ, Gundersen KT, Torjesen PA, Svebak S. Insulin sensitivity after maximal and endurance resistance training. *Journal of Strength & Conditioning Research*. 2012; 26: 327-34.
564 Hardcastle S, Taylor A, Bailey M, Castle R. A randomized controlled trial on the effectiveness of a primary health care based counseling intervention on physical activity, diet and CHD risk. *Patient education and counseling*. 2008; 70: 31-39.
565 Harrington DM, Champagne CM, Broyles ST, Johnson WD, Tudor-Locke C, Katzmarzyk PT. Cardiometabolic risk factor response to a lifestyle intervention: a randomized trial. *Metabolic syndrome and related disorders*. 2015; 13: 125-31.
566 Harris Jackson K, West SG, Vanden Heuvel JP, Jonnalagadda SS, Ross AB, Hill AM*, et al.* Effects of whole and refined grains in a weight-loss diet on markers of metabolic syndrome in individuals with increased waist circumference: a randomized controlled-feeding trial. *American Journal of Clinical Nutrition*. 2014; 100: 577-86.
567 Harris L, Hankey C, Jones N, Murray H, Pert C, Tobin J*, et al.* Process evaluation of a cluster-randomised controlled trial of multi-component weight management programme in adults with intellectual disabilities and obesity. *Journal of Intellectual Disability Research*. 2019; 63: 49-63.
568 Harris L, Hankey C, Jones N, Pert C, Murray H, Tobin J*, et al.* A cluster randomised control trial of a multi-component weight management programme for adults with intellectual disabilities and obesity. *British Journal of Nutrition*. 2017; 118: 229-40.
569 Hartwich J, Malec MM, Partyka L, Perez-Martinez P, Marin C, Lopez-Miranda J*, et al.* The effect of the plasma n-3/n-6 polyunsaturated fatty acid ratio on the dietary LDL phenotype transformation - insights from the LIPGENE study. *Clinical Nutrition*. 2009; 28: 510-15.
570 Harvey-Berino J. Changing health behavior via telecommunications technology: Using interactive television to treat obesity. *Behavior Therapy*. 1998; 29: 505-19.
571 Harvey-Berino J. Calorie restriction is more effective for obesity treatment than dietary fat restriction. *Annals of Behavioral Medicine*. 1999; 21: 35-39.
572 Harvey-Berino J, Pintauro SJ, Gold EC. The feasibility of using Internet support for the maintenance of weight loss. *Behavior modification*. 2002; 26: 103-16.
573 Harvey-Berino J, West D, Krukowski R, Prewitt E, VanBiervliet A, Ashikaga T*, et al.* Internet delivered behavioral obesity treatment. *Preventive Medicine: An International Journal Devoted to Practice and Theory*. 2010; 51: 123-28.
574 Haufe S, Engeli S, Kaminski J, Witt H, Rein D, Kamlage B*, et al.* Branched-chain amino acid catabolism rather than amino acids plasma concentrations is associated with diet-induced changes in insulin resistance in overweight to obese individuals. *Nutrition, metabolism, and cardiovascular diseases : NMCD*. 2017; 27: 858-64.
575 Haufe S, Engeli S, Kast P, Böhnke J, Utz W, Haas V*, et al.* Randomized comparison of reduced fat and reduced carbohydrate hypocaloric diets on intrahepatic fat in overweight and obese human subjects. *Hepatology (Baltimore, Md)*. 2011; 53: 1504-14.

576 Haufe S, Utz W, Engeli S, Kast P, Böhnke J, Pofahl M*, et al.* Left ventricular mass and function with reduced-fat or reduced-carbohydrate hypocaloric diets in overweight and obese subjects. *Hypertension (dallas, tex : 1979)*. 2012; 59: 70-75.
577 Hays NP, Starling RD, Liu X, Sullivan DH, Trappe TA, Fluckey JD*, et al.* Effects of an ad libitum low-fat, high-carbohydrate diet on body weight, body composition, and fat distribution in older men and women: a randomized controlled trial. *Archives of Internal Medicine*. 2004; 164: 210-17.
578 Hays NP, Starling RD, Sullivan DH, Fluckey JD, Coker RH, Williams RH*, et al.* Effects of an ad libitum, high carbohydrate diet and aerobic exercise training on insulin action and muscle metabolism in older men and women. *Journals of Gerontology Series A-Biological Sciences & Medical Sciences*. 2006; 61: 299-304.
579 Hébert JR, Wirth M, Davis L, Davis B, Harmon BE, Hurley TG*, et al.* C-reactive protein levels in African Americans: A diet and lifestyle randomized community trial. *American Journal of Preventive Medicine*. 2013; 45: 430-40.
580 Heianza Y, Sun D, Ma W, Zheng Y, Champagne CM, Bray GA*, et al.* Gut-microbiome-related LCT genotype and 2-year changes in body composition and fat distribution: the POUNDS Lost Trial. *International journal of obesity (2005)*. 2018; 42: 1565-73.
581 Heianza Y, Sun D, Smith SR, Bray GA, Sacks FM, Qi L. Changes in Gut Microbiota-Related Metabolites and Long-term Successful Weight Loss in Response to Weight-Loss Diets: the POUNDS Lost Trial. *Diabetes Care*. 2018; 41: 413-19.
582 Heianza Y, Sun D, Wang T, Huang T, Bray GA, Sacks FM*, et al.* Starch Digestion-Related Amylase Genetic Variant Affects 2-Year Changes in Adiposity in Response to Weight-Loss Diets: the POUNDS Lost Trial. *Diabetes*. 2017; 66: 2416-23.
583 Heianza Y, Zhou T, Yuhang C, Huang T, Willett WC, Hu FB*, et al.* Starch digestion-related amylase genetic variants, diet, and changes in adiposity: Analyses in prospective cohort studies and a randomized dietary intervention. *Diabetes*. 2020; 69: 1917-26.
584 Heideman WH, de Wit M, Middelkoop BJ, Nierkens V, Stronks K, Verhoeff AP*, et al.* Diabetes risk reduction in overweight first degree relatives of type 2 diabetes patients: effects of a low-intensive lifestyle education program (DiAlert) A randomized controlled trial. *Patient Education & Counseling*. 2015; 98: 476-83.
585 Heilbronn LK, Noakes M, Clifton PM. Effect of energy restriction, weight loss, and diet composition on plasma lipids and glucose in patients with type 2 diabetes. *Diabetes Care*. 1999; 22: 889-95.
586 Heitzmann CA, Kaplan RM, Wilson DK, Sandler J. Sex differences in weight loss among adults with type II diabetes mellitus. *Journal of Behavioral Medicine*. 1987; 10: 197-211.
587 Heller SR, Clarke P, Daly H, Davis I, McCulloch DK, Allison SP*, et al.* Group education for obese patients with type 2 diabetes: greater success at less cost. *Diabetic Medicine*. 1988; 5: 552-56.
588 Henderson RM, Leng XI, Chmelo EA, Brinkley TE, Lyles MF, Marsh AP*, et al.* Gait speed response to aerobic versus resistance exercise training in older adults. *Aging clinical and experimental research*. 2017; 29: 969-76.
589 Henriksen M, Christensen R, Danneskiold-Samsoe B, Bliddal H. Changes in lower extremity muscle mass and muscle strength after weight loss in obese patients with knee osteoarthritis: a prospective cohort study. *Arthritis & Rheumatism*. 2012; 64: 438-42.
590 Henriksen M, Christensen R, Hunter DJ, Gudbergsen H, Boesen M, Lohmander LS*, et al.* Structural changes in the knee during weight loss maintenance after a significant weight loss in obese patients with osteoarthritis: a report of secondary outcome analyses from a randomized controlled trial. *Osteoarthritis & Cartilage*. 2014; 22: 639-46.

591 Hersey JC, Khavjou O, Strange LB, Atkinson RL, Blair SN, Campbell S*, et al.* The efficacy and cost-effectiveness of a community weight management intervention: A randomized controlled trial of the health weight management demonstration. *Preventive Medicine: An International Journal Devoted to Practice and Theory*. 2012; 54: 42-49.
592 Heshka S, Greenway F, Anderson JW, Atkinson RL, Hill JO, Phinney SD*, et al.* Self-help weight loss versus a structured commercial program after 26 weeks: a randomized controlled study. *American Journal of Medicine*. 2000; 109: 282-87.
593 Hill AM, Harris Jackson KA, Roussell MA, West SG, Kris-Etherton PM. Type and amount of dietary protein in the treatment of metabolic syndrome: a randomized controlled trial. *American Journal of Clinical Nutrition*. 2015; 102: 757-70.
594 Hinderliter AL, Sherwood A, Craighead LW, Lin PH, Watkins L, Babyak MA*, et al.* The long-term effects of lifestyle change on blood pressure: one-year follow-up of the ENCORE study. *American Journal of Hypertension*. 2014; 27: 734-41.
595 Hinkleman LL, Nieman DC. The effects of a walking program on body composition and serum lipids and lipoproteins in overweight women. *Journal of sports medicine and physical fitness*. 1993; 33: 49-58.
596 Hjorth MF, Astrup A, Zohar Y, Urban LE, Sayer RD, Patterson BW*, et al.* Personalized nutrition: pretreatment glucose metabolism determines individual long-term weight loss responsiveness in individuals with obesity on low-carbohydrate versus low-fat diet. *International Journal of Obesity*. 2019; 43: 2037-44.
597 Hjorth MF, Blædel T, Bendtsen LQ, Lorenzen JK, Holm JB, Kiilerich P*, et al.* Prevotella-to-Bacteroides ratio predicts body weight and fat loss success on 24-week diets varying in macronutrient composition and dietary fiber: results from a post-hoc analysis. *International journal of obesity (2005)*. 2019; 43: 149-57.
598 Hjorth MF, Due A, Larsen TM, Astrup A. Pretreatment fasting plasma glucose modifies dietary weight loss maintenance success: Results from a stratified RCT. *Obesity*. 2017; 25: 2045-48.
599 Hjorth MF, Roager HM, Larsen TM, Poulsen SK, Licht TR, Bahl MI*, et al.* Pre-treatment microbial Prevotella-to-Bacteroides ratio, determines body fat loss success during a 6-month randomized controlled diet intervention. *International journal of obesity (2005)*. 2018; 42: 580-83.
600 Ho SS, Dhaliwal SS, Hills AP, Pal S. The effect of 12 weeks of aerobic, resistance or combination exercise training on cardiovascular risk factors in the overweight and obese in a randomized trial. *BMC Public Health*. 2012; 12: 704.
601 Ho SS, Dhaliwal SS, Hills AP, Pal S. Effects of chronic exercise training on inflammatory markers in Australian overweight and obese individuals in a randomized controlled trial. *Inflammation*. 2013; 36: 625-32.
602 Ho SS, Radavelli-Bagatini S, Dhaliwal SS, Hills AP, Pal S. Resistance, aerobic, and combination training on vascular function in overweight and obese adults. *Journal of Clinical Hypertension*. 2012; 14: 848-54.
603 Höchsmann C, Dorling JL, Apolzan JW, Johannsen NM, Hsia DS, Church TS*, et al.* Effect of different doses of supervised aerobic exercise on heart rate recovery in inactive adults who are overweight or obese: results from E-MECHANIC. *European Journal of Applied Physiology*. 2019; 119: 2095-103.
604 Hodgson JM, Lee YP, Puddey IB, Sipsas S, Ackland TR, Beilin LJ*, et al.* Effects of increasing dietary protein and fibre intake with lupin on body weight and composition and blood lipids in overweight men and women. *International Journal of Obesity*. 2010; 34: 1086-94.

605 Holliday A, Burgin A, Fernandez EV, Fenton SAM, Thielecke F, Blannin AK. Points-based physical activity: a novel approach to facilitate changes in body composition in inactive women with overweight and obesity. *BMC Public Health*. 2018; 18: 261.
606 Holt RIG, Gossage-Worrall R, Hind D, Bradburn MJ, McCrone P, Morris T*, et al.* Structured lifestyle education for people with schizophrenia, schizoaffective disorder and first-episode psychosis (STEPWISE): Randomised controlled trial. *The British Journal of Psychiatry*. 2019; 214: 63-73.
607 Holtrop JS, Luo Z, Piatt G, Green LA, Chen Q, Piette J. Diabetic and Obese Patient Clinical Outcomes Improve During a Care Management Implementation in Primary Care. *Journal of Primary Care & Community Health*. 2017; 8: 312-18.
608 Holzapfel C, Cresswell L, Ahern AL, Fuller NR, Eberhard M, Stoll J*, et al.* The challenge of a 2-year follow-up after intervention for weight loss in primary care. *International Journal of Obesity*. 2014; 38: 806-11.
609 Hong J, Kim SW, Joo H, Kong HJ. Effects of smartphone mirroring-based telepresence exercise on body composition and physical function in obese older women. *Aging Clinical & Experimental Research*. 2022; 14: 14.
610 Honkola A, Forsen T, Eriksson J. Resistance training improves the metabolic profile in individuals with type 2 diabetes. *Acta Diabetologica*. 1997; 34: 245-48.
611 Hsu YI, Chen YC, Lee CL, Chang NJ. Effects of diet control and telemedicine-based resistance exercise intervention on patients with obesity and knee osteoarthritis: A randomized control trial. *International Journal of Environmental Research and Public Health*. 2021; 18(15) (no pagination).
612 Hu H, Yuan G, Wang X, Sun J, Gao Z, Zhou T*, et al.* Effects of a diet with or without physical activity on angiopoietin-like protein 8 concentrations in overweight/obese patients with newly diagnosed type 2 diabetes: a randomized controlled trial. *Endocrine Journal*. 2019; 66: 89-105.
613 Hu T, Yao L, Reynolds K, Niu T, Li S, Whelton P*, et al.* The effects of a low-carbohydrate diet on appetite: A randomized controlled trial. *Nutrition Metabolism & Cardiovascular Diseases*. 2016; 26: 476-88.
614 Hu T, Yao L, Reynolds K, Whelton PK, Niu T, Li S*, et al.* The Effects of a Low-Carbohydrate Diet vs. a Low-Fat Diet on Novel Cardiovascular Risk Factors: A Randomized Controlled Trial. *Nutrients*. 2015; 7: 7978-94.
615 Huang SW, Ku JW, Lin LF, Liao CD, Chou LC, Liou TH. Body composition influenced by progressive elastic band resistance exercise of sarcopenic obesity elderly women: a pilot randomized controlled trial. *European journal of physical & rehabilitation medicine*. 2017; 53: 556-63.
616 Huang T, Huang J, Qi Q, Li Y, Bray GA, Rood J*, et al.* PCSK7 genotype modifies effect of a weight-loss diet on 2-year changes of insulin resistance: the POUNDS LOST trial. *Diabetes Care*. 2015; 38: 439-44.
617 Huang T, Ley SH, Zheng Y, Wang T, Bray GA, Sacks FM*, et al.* Genetic susceptibility to diabetes and long-term improvement of insulin resistance and β cell function during weight loss: the Preventing Overweight Using Novel Dietary Strategies (POUNDS LOST) trial. *American Journal of Clinical Nutrition*. 2016; 104: 198-204.
618 Huang T, Wang T, Heianza Y, Sun D, Ivey K, Durst R*, et al.* HNF1A variant, energy-reduced diets and insulin resistance improvement during weight loss: The POUNDS Lost trial and DIRECT. *Diabetes, Obesity & Metabolism*. 2018; 20: 1445-52.

619 Huber JM, Shapiro JS, Wieland ML, Croghan IT, Vickers Douglas KS, Schroeder DR*, et al.* Telecoaching plus a portion control plate for weight care management: A randomized trial. *Trials*. 2015; 16.
620 Huffman KM, Hawk VH, Henes ST, Ocampo CI, Orenduff MC, Slentz CA*, et al.* Exercise effects on lipids in persons with varying dietary patterns-does diet matter if they exercise? Responses in Studies of a Targeted Risk Reduction Intervention through Defined Exercise I. *American Heart Journal*. 2012; 164: 117-24.
621 Huffman KM, Redman LM, Landerman LR, Pieper CF, Stevens RD, Muehlbauer MJ*, et al.* Caloric restriction alters the metabolic response to a mixed-meal: results from a randomized, controlled trial. *PloS one*. 2012; 7: e28190.
622 Huffman KM, Slentz CA, Bales CW, Houmard JA, Kraus WE. Relationships between adipose tissue and cytokine responses to a randomized controlled exercise training intervention. *Metabolism: Clinical & Experimental*. 2008; 57: 577-83.
623 Hughes SL, Tussing-Humphreys L, Schiffer L, Smith-Ray R, Marquez DX, DeMott AD*, et al.* Fit & Strong! Plus Trial Outcomes for Obese Older Adults with Osteoarthritis. *Gerontologist*. 2018.
624 Iglesias-García C, Toimil-Iglesias A, Alonso-Villa MJ. Pilot study of the efficacy of an educational programme to reduce weight, on overweight and obese patients with chronic stable schizophrenia. *Journal of psychiatric and mental health nursing*. 2010; 17: 849-51.
625 Ikenaga M, Yamada Y, Kose Y, Morimura K, Higaki Y, Kiyonaga A*, et al.* Effects of a 12-week, short-interval, intermittent, low-intensity, slow-jogging program on skeletal muscle, fat infiltration, and fitness in older adults: randomized controlled trial. *European Journal of Applied Physiology*. 2017; 117: 7-15.
626 Imes CC, Zheng Y, Mendez DD, Rockette-Wagner BJ, Mattos MK, Goode RW*, et al.* Group-based trajectory analysis of physical activity change in a US weight loss intervention. *Journal of Physical Activity & Health*. 2018; 15: 840-46.
627 Iqbal N, Seshadri P, Stern L, Loh J, Kundu S, Jafar T*, et al.* Serum resistin is not associated with obesity or insulin resistance in humans. *European Review for Medical & Pharmacological Sciences*. 2005; 9: 161-65.
628 Iqbal N, Vetter ML, Moore RH, Chittams JL, Dalton-Bakes CV, Dowd M*, et al.* Effects of a low-intensity intervention that prescribed a low-carbohydrate vs. a low-fat diet in obese, diabetic participants. *Obesity*. 2010; 18: 1733-38.
629 Irandoust K, Taheri M, Shavikloo J. The Effect of Water-Based Aerobic Training on the Dynamic Balance and Walking Speed of Obese Elderly Men with Low Back Pain. *Sleep & Hypnosis*. 2018; 20: 233-40.
630 Isabelle C, Christelle C, Patrick R, Tony L, Van der linden M, Alain G. Acceptance and efficacy of a guided internet self-help treatment program for obese patients with binge eating disorder. *Clinical practice and epidemiology in mental health*. 2011; 7.
631 Izaola O, Primo D, Gomez Hoyos E, Lopez Gomez JJ, Ortola A, de Luis D. Association of rs670 variant of APOA1 gene with lipid profile and insulin resistance after 9 months of a high protein/low carbohydrate vs a standard hypocaloric diet. *Clinical Nutrition*. 2020; 39: 988-93.
632 Jackson JB, Pietrabissa G, Rossi A, Manzoni GM, Castelnuovo G. Brief strategic therapy and cognitive behavioral therapy for women with binge eating disorder and comorbid obesity: A randomized clinical trial one-year follow-up. *Journal of Consulting & Clinical Psychology*. 2018; 86: 688-701.
633 Jacobsen DJ, Donnelly JE, Snyder-Heelan K, Livingston K. Adherence and attrition with intermittent and continuous exercise in overweight women. *International Journal of Sports Medicine*. 2003; 24: 459-64.

634 Jahangiry L, Montazeri A, Najafi M, Yaseri M, Farhangi MA. An interactive web-based intervention on nutritional status, physical activity and health-related quality of life in patient with metabolic syndrome: a randomized-controlled trial (The Red Ruby Study). *Nutrition & Diabetes*. 2017; 7: e240.
635 Jahn J, Spielau M, Brandsch C, Stangl GI, Delank K-S, Bähr I*, et al.* Decreased NK cell functions in obesity can be reactivated by fat mass reduction. *Obesity*. 2015; 23: 2233-41.
636 Jakicic JM, Davis KK, Rogers RJ, Sherman SA, Barr S, Marcin ML*, et al.* Feasibility of Integration of Yoga in a Behavioral Weight-Loss Intervention: A Randomized Trial. *Obesity (19307381)*. 2021; 29: 512-20.
637 Jakicic JM, Egan CM, Fabricatore AN, Gaussoin SA, Glasser SP, Hesson LA*, et al.* Four-year change in cardiorespiratory fitness and influence on glycemic control in adults with type 2 diabetes in a randomized trial: the Look AHEAD Trial. *Diabetes Care*. 2013; 36: 1297-303.
638 Jakubowicz D, Barnea M, Wainstein J, Froy O. High Caloric intake at breakfast vs. dinner differentially influences weight loss of overweight and obese women. *Obesity*. 2013; 21: 2504-12.
639 Jalkanen L. The effect of a weight reduction program on cardiovascular risk factors among overweight hypertensives in primary health care. *Scandinavian journal of social medicine*. 1991; 19: 66-71.
640 James AP, Watts GF, Barrett PH, Smith D, Pal S, Chan DC*, et al.* Effect of weight loss on postprandial lipemia and low-density lipoprotein receptor binding in overweight men. *Metabolism: clinical and experimental*. 2003; 52: 136-41.
641 Jane M, Hagger M, Foster J, Ho S, Kane R, Pal S. Effects of a weight management program delivered by social media on weight and metabolic syndrome risk factors in overweight and obese adults: a randomised controlled trial. *PloS one*. 2017; 12: e0178326.
642 Janney CA, Jakicic JM. The influence of exercise and BMI on injuries and illnesses in overweight and obese individuals: A randomized control trial. *The International Journal of Behavioral Nutrition and Physical Activity*. 2010; 7.
643 Jansson SP, Engfeldt P, Magnuson A, Pt GL, Liljegren G. Interventions for lifestyle changes to promote weight reduction, a randomized controlled trial in primary health care. *BMC Research Notes*. 2013; 6: 213.
644 Jazi AA, Sarteshnizi EM, Fathi M. Effect of selected elastic band resistance training on serum levels of fibroblast growth factor 23, tnf-alpha and hscrp in overweight elderly women. [Persian]. *Iranian Journal of Obstetrics, Gynecology and Infertility*. 2021; 24(1): 26-35.
645 Jean-Baptiste M, Tek C, Liskov E, Chakunta UR, Nicholls S, Hassan AQ*, et al.* A pilot study of a weight management program with food provision in schizophrenia. *Schizophrenia Research*. 2007; 96: 198-205.
646 Jebb SA, Ahern AL, Olson AD, Aston LM, Holzapfel C, Stoll J*, et al.* Primary care referral to a commercial provider for weight loss treatment versus standard care: a randomised controlled trial. *Lancet (london, england)*. 2011; 378: 1485-92.
647 Jefferson ME, Nicklas BJ, Chmelo EA, Crotts CI, Shaltout HA, Diz DI*, et al.* Effects of Resistance Training With and Without Caloric Restriction on Arterial Stiffness in Overweight and Obese Older Adults. *American Journal of Hypertension*. 2016; 29: 494-500.
648 Jeffery RW, Gillum R, Gerber WM, Jacobs D, Elmer PJ, Prineas RJ. Weight and sodium reduction for the prevention of hypertension: a comparison of group treatment and individual counseling. *American Journal of Public Health*. 1983; 73: 691-93.

649 Jeffery RW, Hellerstedt WL, French SA, Baxter JE. A randomized trial of counseling for fat restriction versus calorie restriction in the treatment of obesity. *International journal of obesity and related metabolic disorders*. 1995; 19: 132-37.
650 Jeffery RW, Linde JA, Finch EA, Rothman AJ, King CM. A Satisfaction Enhancement Intervention for Long-Term Weight Loss. *Obesity*. 2006; 14: 863-69.
651 Jeffery RW, Wing RR, Sherwood NE, Tate DF. Physical activity and weight loss: does prescribing higher physical activity goals improve outcome? *American Journal of Clinical Nutrition*. 2003; 78: 684-89.
652 Jeffery RW, Wing RR, Thorson C, Burton LR. Use of personal trainers and financial incentives to increase exercise in a behavioral weight-loss program. *Journal of consulting and clinical psychology*. 1998; 66: 777-83.
653 Jehn ML, Patt MR, Appel LJ, Miller ER, III. One year follow-up of overweight and obese hypertensive adults following intensive lifestyle therapy. *Journal of Human Nutrition & Dietetics*. 2006; 19: 349-54.
654 Jen KL, Djuric Z, DiLaura NM, Buison A, Redd JN, Maranci V*, et al.* Improvement of metabolism among obese breast cancer survivors in differing weight loss regimens. *Obesity Research*. 2004; 12: 306-12.
655 Jerome GJ, Young DR, Dalcin AT, Wang N-Y, Gennusa J, III, Goldsholl S*, et al.* Cardiorespiratory benefits of group exercise among adults with serious mental illness. *Psychiatry research*. 2017; 256: 85-87.
656 Jesudason DR, Pedersen E, Clifton PM. Weight-loss diets in people with type 2 diabetes and renal disease: a randomized controlled trial of the effect of different dietary protein amounts. *American Journal of Clinical Nutrition*. 2013; 98: 494-501.
657 Johnson DL, Brinker GD. A comparative study of multi-component weight loss treatments: A replication and exploratory look at female smokers, nonsmokers, and participant BMIs. *North American Journal of Psychology*. 2001; 3: 13-30.
658 Johnson KE, Alencar MK, Coakley KE, Swift DL, Cole NH, Mermier CM*, et al.* Telemedicine-based health coaching is effective for inducing weight loss and improving metabolic markers. *Telemedicine and e-Health*. 2019; 25: 85-92.
659 Johnson SS, Paiva AL, Cummins CO, Johnson JL, Dyment SJ, Wright JA*, et al.* Transtheoretical model-based multiple behavior intervention for weight management: effectiveness on a population basis. *Preventive Medicine*. 2008; 46: 238-46.
660 Jones DW, Miller ME, Wofford MR, Anderson DC, Cameron ME, Willoughby DL*, et al.* The effect of weight loss intervention on antihypertensive medication requirements in the hypertension Optimal Treatment (HOT) study. *American Journal of Hypertension*. 1999; 12: 1175-80.
661 Jones KW, Eller LK, Parnell JA, Doyle-Baker PK, Edwards AL, Reimer RA. Effect of a dairy- and calcium-rich diet on weight loss and appetite during energy restriction in overweight and obese adults: a randomized trial. *European Journal of Clinical Nutrition*. 2013; 67: 371-76.
662 Jones SE, Owens HM, Bennett GA. Does behaviour therapy work for dietitians? An experimental evaluation of the effects of three procedures in a weight reduction clinic. *Human nutrition Applied nutrition*. 1986; 40: 272-81.
663 Jospe MR, Roy M, Brown RC, Williams SM, Osborne HR, Meredith-Jones KA*, et al.* The Effect of Different Types of Monitoring Strategies on Weight Loss: a Randomized Controlled Trial. *Obesity (Silver Spring, Md)*. 2017; 25: 1490-98.
664 Juanola-Falgarona M, Salas-Salvadó J, Ibarrola-Jurado N, Rabassa-Soler A, Díaz-López A, Guasch-Ferré M*, et al.* Effect of the glycemic index of the diet on weight loss, modulation

of satiety, inflammation, and other metabolic risk factors: a randomized controlled trial. *American Journal of Clinical Nutrition*. 2014; 100: 27-35.
665 Kadoglou NP, Fotiadis G, Athanasiadou Z, Vitta I, Lampropoulos S, Vrabas IS. The effects of resistance training on ApoB/ApoA-I ratio, Lp(a) and inflammatory markers in patients with type 2 diabetes. *Endocrine*. 2012; 42: 561-69.
666 Kadoglou NP, Iliadis F, Angelopoulou N, Perrea D, Ampatzidis G, Liapis CD*, et al.* The anti-inflammatory effects of exercise training in patients with type 2 diabetes mellitus. *European journal of cardiovascular prevention and rehabilitation*. 2007; 14: 837-43.
667 Kadoglou NP, Iliadis F, Sailer N, Athanasiadou Z, Vitta I, Kapelouzou A*, et al.* Exercise training ameliorates the effects of rosiglitazone on traditional and novel cardiovascular risk factors in patients with type 2 diabetes mellitus. *Metabolism: Clinical & Experimental*. 2010; 59: 599-607.
668 Kadoglou NP, Vrabas IS, Kapelouzou A, Lampropoulos S, Sailer N, Kostakis A*, et al.* The impact of aerobic exercise training on novel adipokines, apelin and ghrelin, in patients with type 2 diabetes. *Medical Science Monitor*. 2012; 18: CR290-95.
669 Kahleova H, Dort S, Holubkov R, Barnard ND. A Plant-Based High-Carbohydrate, Low-Fat Diet in Overweight Individuals in a 16-Week Randomized Clinical Trial: The Role of Carbohydrates. *Nutrients*. 2018; 10: 14.
670 Kahleova H, Fleeman R, Hlozkova A, Holubkov R, Barnard ND. A plant-based diet in overweight individuals in a 16-week randomized clinical trial: metabolic benefits of plant protein. *Nutrition & Diabetes*. 2018; 8: 58.
671 Kahleova H, Hlozkova A, Fleeman R, Fletcher K, Holubkov R, Barnard ND. Fat Quantity and Quality, as Part of a Low-Fat, Vegan Diet, Are Associated with Changes in Body Composition, Insulin Resistance, and Insulin Secretion. A 16-Week Randomized Controlled Trial. *Nutrients*. 2019; 11: 13.
672 Kahleova H, Tura A, Hill M, Holubkov R, Barnard ND. A Plant-Based Dietary Intervention Improves Beta-Cell Function and Insulin Resistance in Overweight Adults: A 16-Week Randomized Clinical Trial. *Nutrients*. 2018; 10: 09.
673 Kahleova H, Znayenko-Miller T, Uribarri J, Holubkov R, Barnard ND. Dietary advanced glycation products and their associations with insulin sensitivity and body weight: A 16-week randomized clinical trial. *Obesity Science & Practice*. 2023; 9: 235-42.
674 Kaholokula JK, Townsend CK, Ige A, Sinclair K, Mau MK, Leake A*, et al.* Sociodemographic, behavioral, and biological variables related to weight loss in native Hawaiians and other Pacific Islanders. *Obesity (Silver Spring, Md)*. 2013; 21: E196-203.
675 Kaholokula JKa, Mau MK, Efird JT, Leake A, West M, Palakiko D-M*, et al.* A family and community focused lifestyle program prevents weight regain in Pacific Islanders: A pilot randomized controlled trial. *Health Education & Behavior*. 2012; 39: 386-95.
676 Kalam F, Kroeger CM, Trepanowski JF, Gabel K, Song JH, Cienfuegos S*, et al.* Beverage intake during alternate-day fasting: Relationship to energy intake and body weight. *Nutrition & Health*. 2019; 25: 167-71.
677 Kallio P, Kolehmainen M, Laaksonen DE, Pulkkinen L, Atalay M, Mykkänen H*, et al.* Inflammation markers are modulated by responses to diets differing in postprandial insulin responses in individuals with the metabolic syndrome. *American Journal of Clinical Nutrition*. 2008; 87: 1497-503.
678 Kalodner CR, DeLucia JL. The individual and combined effects of cognitive therapy and nutrition education as additions to a behavior modification program for weight loss. *Addictive Behaviors*. 1991; 16: 255-63.

679 Kamel DM, Thabet AA, Tantawy SA, Radwa MM. Abdominal versus pelvic floor muscles exercises in mild stress urinary incontinence in obese Egyptian women. *Indian journal of physiotherapy & occupational therapy*. 2012; 6: 36-40.
680 Kang M, Yoo HJ, Kim M, Kim M, Lee JH. Metabolomics identifies increases in the acylcarnitine profiles in the plasma of overweight subjects in response to mild weight loss: a randomized, controlled design study. *Lipids in health and disease*. 2018; 17: 237.
681 Kang SJ. Trekking exercise promotes cardiovascular health and fitness benefits in older obese women. *Journal of Exercise Rehabilitation*. 2014; 10: 225-29.
682 Karl JP, Cheatham RA, Das SK, Hyatt RR, Gilhooly CH, Pittas AG*, et al.* Effect of glycemic load on eating behavior self-efficacy during weight loss. *Appetite*. 2014; 80: 204-11.
683 Karl JP, Roberts SB, Schaefer EJ, Gleason JA, Fuss P, Rasmussen H*, et al.* Effects of carbohydrate quantity and glycemic index on resting metabolic rate and body composition during weight loss. *Obesity*. 2015; 23: 2190-98.
684 Karvetti RL, Knuts LR. Effects of comprehensive rehabilitation on weight reduction in myocardial infarction patients. *Scandinavian journal of rehabilitation medicine*. 1983; 15: 11-16.
685 Katcher HI, Legro RS, Kunselman AR, Gillies PJ, Demers LM, Bagshaw DM*, et al.* The effects of a whole grain-enriched hypocaloric diet on cardiovascular disease risk factors in men and women with metabolic syndrome. *American Journal of Clinical Nutrition*. 2008; 87: 79-90.
686 Katula JA, Vitolins MZ, Morgan TM, Lawlor MS, Blackwell CS, Isom SP*, et al.* The Healthy Living Partnerships to Prevent Diabetes study: 2-year outcomes of a randomized controlled trial. *American Journal of Preventive Medicine*. 2013; 44: S324-32.
687 Katz DL, Chan W, Gonzalez M, Larson D, Nawaz H, Abdulrahman M*, et al.* Technical skills for weight loss: preliminary data from a randomized trial. *Preventive Medicine*. 2002; 34: 608-15.
688 Katzmarzyk PT, Martin CK, Newton RL, Jr., Apolzan JW, Arnold CL, Davis TC*, et al.* *Patient Centered Outcomes Research Institute*. 2021; 06: 06.
689 Kaukua J, Pekkarinen T, Sane T, Mustajoki P. Health-related quality of life in WHO class II-III obese men losing weight with very-low-energy diet and behaviour modification: A randomised clinical trial. *International Journal of Obesity*. 2002; 26: 487-95.
690 Keating FK, Schneider DJ, Savage PD, Bunn JY, Harvey-Berino J, Ludlow M*, et al.* Effect of exercise training and weight loss on platelet reactivity in overweight patients with coronary artery disease. *Journal of Cardiopulmonary Rehabilitation & Prevention*. 2013; 33: 371-77.
691 Keating SE, Machan EA, O'Connor HT, Gerofi JA, Sainsbury A, Caterson ID*, et al.* Continuous exercise but not high intensity interval training improves fat distribution in overweight adults. *Journal of Obesity*. 2014; 2014: 834865.
692 Kelley DS, Daudu PA, Branch LB, Johnson HL, Taylor PC, Mackey B. Energy restriction decreases number of circulating natural killer cells and serum levels of immunoglobulins in overweight women. *European Journal of Clinical Nutrition*. 1994; 48: 9-18.
693 Kemmler W, Birlauf A, von Stengel S. Effects of Whole-Body-Electromyostimulation on Body Composition and Cardiac Risk Factors in Elderly Men with the Metabolic Syndrome. The TEST-II Study. *Deutsche Zeitschrift Fur Sportmedizin*. 2010; 61: 117-23.
694 Kemmler W, von Stengel S. Whole-body electromyostimulation as a means to impact muscle mass and abdominal body fat in lean, sedentary, older female adults: subanalysis of the TEST-III trial. *Clinical Interventions In Aging*. 2013; 8: 1353-64.

695 Kemmler W, von Stengel S, Engelke K, Haberle L, Mayhew JL, Kalender WA. Exercise, body composition, and functional ability: a randomized controlled trial. *American Journal of Preventive Medicine*. 2010; 38: 279-87.
696 Kempf K, Rohling M, Banzer W, Braumann KM, Halle M, McCarthy D*, et al.* High-Protein, Low-Glycaemic Meal Replacement Decreases Fasting Insulin and Inflammation Markers-A 12-Month Subanalysis of the ACOORH Trial. *Nutrients*. 2021; 13: 23.
697 Kemppainen T, Ruoppi P, Seppa J, Sahlman J, Peltonen M, Tukiainen H*, et al.* Effect of weight reduction on rhinometric measurements in overweight patients with obstructive sleep apnea. *American Journal of Rhinology*. 2008; 22: 410-15.
698 Kennedy BM, Paeratakul S, Champagne CM, Ryan DH, Harsha DW, McGee B*, et al.* A pilot church-based weight loss program for African-American adults using church members as health educators: a comparison of individual and group intervention. *Ethnicity & Disease*. 2005; 15: 373-78.
699 Keogh JB, Luscombe-Marsh ND, Noakes M, Wittert GA, Clifton PM. Long-term weight maintenance and cardiovascular risk factors are not different following weight loss on carbohydrate-restricted diets high in either monounsaturated fat or protein in obese hyperinsulinaemic men and women. *British Journal of Nutrition*. 2007; 97: 405-10.
700 Keogh JB, Pedersen E, Petersen KS, Clifton PM. Effects of intermittent compared to continuous energy restriction on short-term weight loss and long-term weight loss maintenance. *Clinical Obesity*. 2014; 4: 150-56.
701 Keränen A, Savolainen MJ, Reponen AH, Kujari M, Lindeman SM, Bloigu RS*, et al.* The effect of eating behavior on weight loss and maintenance during a lifestyle intervention. *Preventive Medicine*. 2009; 49: 32-38.
702 Kerr J, Patrick K, Norman G, Stein MB, Calfas K, Zabinski M*, et al.* Randomized control trial of a behavioral intervention for overweight women: Impact on depressive symptoms. *Depression and Anxiety*. 2008; 25: 555-58.
703 Kerrigan SG, Forman EM, Williams D, Patel M, Loyka C, Zhang F*, et al.* Project Step: A Randomized Controlled Trial Investigating the Effects of Frequent Feedback and Contingent Incentives on Physical Activity. *Journal of Physical Activity & Health*. 2021; 18: 247-53.
704 Khakimov B, Poulsen SK, Savorani F, Acar E, Gürdeniz G, Larsen TM*, et al.* New Nordic Diet versus Average Danish Diet: a Randomized Controlled Trial Revealed Healthy Long-Term Effects of the New Nordic Diet by GC-MS Blood Plasma Metabolomics. *Journal of Proteome Research*. 2016; 15: 1939-54.
705 Kiernan M, Brown SD, Schoffman DE, Lee K, King AC, Taylor CB*, et al.* Promoting healthy weight with "stability skills first": a randomized trial. *Journal of consulting and clinical psychology*. 2013; 81: 336-46.
706 Kikuchi Y, Nozaki S, Makita M, Yokozuka S, Fukudome SI, Yanagisawa T*, et al.* Effects of Whole Grain Wheat Bread on Visceral Fat Obesity in Japanese Subjects: a Randomized Double-Blind Study. *Plant foods for human nutrition (dordrecht, netherlands)*. 2018; 73: 161-65.
707 Kim G, Kim S, Lee YB, Jin SM, Hur KY, Kim JH. A randomized controlled trial of an app-based intervention on physical activity and glycemic control in people with type 2 diabetes. *BMC Medicine*. 2024; 22: 185.
708 Kim H, Kim M, Kojima N, Fujino K, Hosoi E, Kobayashi H*, et al.* Exercise and Nutritional Supplementation on Community-Dwelling Elderly Japanese Women With Sarcopenic Obesity: A Randomized Controlled Trial. *Journal of the American Medical Directors Association*. 2016; 17: 1011-19.

709 Kim HS. Effects of Web-based diabetic education in obese diabetic patients. *Taehan Kanho Hakhoe chi*. 2005; 35: 924-30.
710 Kim HS, Song MS. Technological intervention for obese patients with type 2 diabetes. *Applied Nursing Research*. 2008; 21: 84-89.
711 Kim SH, Lee SH, Ahn KY, Lee DH, Suh YJ, Cho SG*, et al.* Effect of lifestyle modification on serum chemerin concentration and its association with insulin sensitivity in overweight and obese adults with type 2 diabetes. *Clinical Endocrinology*. 2014; 80: 825-33.
712 Kim SI, Kim HS. Effectiveness of mobile and internet intervention in patients with obese type 2 diabetes. *International journal of medical informatics*. 2008; 77: 399-404.
713 Kim SW, Park HY, Jung WS, Lim K. Effects of Twenty-Four Weeks of Resistance Exercise Training on Body Composition, Bone Mineral Density, Functional Fitness and Isokinetic Muscle Strength in Obese Older Women: A Randomized Controlled Trial. *International Journal of Environmental Research & Public Health [Electronic Resource]*. 2022; 19: 06.
714 Kirk EP, Jacobsen DJ, Gibson C, Hill JO, Donnelly JE. Time course for changes in aerobic capacity and body composition in overweight men and women in response to long-term exercise: The midwest exercise trial (MET). *International Journal of Obesity*. 2003; 27: 912-19.
715 Kirkman MS, Weinberger M, Landsman PB, Samsa GP, Shortliffe EA, Simel DL*, et al.* A telephone-delivered intervention for patients with NIDDM. Effect on coronary risk factors. *Diabetes Care*. 1994; 17: 840-46.
716 Kittiskulnam P, Kanjanabuch T, Tangmanjitjaroen K, Chancharoenthana W, Praditpornsilpa K, Eiam-Ong S. The beneficial effects of weight reduction in overweight patients with chronic proteinuric immunoglobulin a nephropathy: a randomized controlled trial. *Journal of Renal Nutrition*. 2014; 24: 200-07.
717 Kjellin A, Ramel S, Rössner S, Thor K. Gastroesophageal reflux in obese patients is not reduced by weight reduction. *Scandinavian Journal of Gastroenterology*. 1996; 31: 1047-51.
718 Klem ML, Klesges RC. Competition in a minimal-contact weight-loss program. *Journal of consulting and clinical psychology*. 1988; 56: 142-44.
719 Kliemann N, Vickerstaff V, Croker H, Johnson F, Nazareth I, Beeken RJ. The role of self-regulatory skills and automaticity on the effectiveness of a brief weight loss habit-based intervention: secondary analysis of the 10 top tips randomised trial. *International Journal of Behavioral Nutrition & Physical Activity*. 2017; 14: 119.
720 Knauper B, Carriere K, Frayn M, Ivanova E, Xu Z, Ames-Bull A*, et al.* The Effects of If-Then Plans on Weight Loss: Results of the McGill CHIP Healthy Weight Program Randomized Controlled Trial. *Obesity*. 2018; 26: 1285-95.
721 Ko LK, Turner-McGrievy GM, Campbell MK. Information processing versus social cognitive mediators of weight loss in a podcast-delivered health intervention. *Health Education & Behavior*. 2014; 41: 197-206.
722 Kolehmainen M, Ulven SM, Paananen J, De Mello V, Schwab U, Carlberg C*, et al.* Healthy Nordic diet downregulates the expression of genes involved in inflammation in subcutaneous adipose tissue in individuals with features of the metabolic syndrome. *American Journal of Clinical Nutrition*. 2015; 101: 228-39.
723 Koo BK, Han KA, Ahn HJ, Jung JY, Kim HC, Min KW. The effects of total energy expenditure from all levels of physical activity vs. physical activity energy expenditure from moderate-to-vigorous activity on visceral fat and insulin sensitivity in obese Type 2 diabetic women. *Diabetic Medicine*. 2010; 27: 1088-92.

724 Kordi R, Dehghani S, Noormohammadpour P, Rostami M, Mansournia MA. Effect of abdominal resistance exercise on abdominal subcutaneous fat of obese women: a randomized controlled trial using ultrasound imaging assessments. *Journal of Manipulative & Physiological Therapeutics*. 2015; 38: 203-09.
725 Korhonen T, Uusitupa M, Aro A, Kumpulainen T, Siitonen O, Voutilainen E*, et al.* Efficacy of dietary instructions in newly diagnosed non-insulin-dependent diabetic patients. Comparison of two different patient education regimens. *Acta Medica Scandinavica*. 1987; 222: 323-31.
726 Koutoukidis DA, Jebb SA, Aveyard P, Astbury NM. The Effect of Moderate Weight Loss on a Non-Invasive Biomarker of Liver Fibrosis: A Randomised Controlled Trial. *Obesity Facts*. 2020; 13: 144-51.
727 Koyuncu H, Fidan V, Toktas H, Binay O, Celik H. Effect of ketogenic diet versus regular diet on voice quality of patients with Parkinson's disease. *Acta Neurologica Belgica*. 2021; 121: 1729-32.
728 Kratz M, Kuzma JN, Hagman DK, van Yserloo B, Matthys CC, Callahan HS*, et al.* n3 PUFAs do not affect adipose tissue inflammation in overweight to moderately obese men and women. *Journal of Nutrition*. 2013; 143: 1340-47.
729 Kratz M, Swarbrick MM, Callahan HS, Matthys CC, Havel PJ, Weigle DS. Effect of dietary n-3 polyunsaturated fatty acids on plasma total and high-molecular-weight adiponectin concentrations in overweight to moderately obese men and women. *American Journal of Clinical Nutrition*. 2008; 87: 347-53.
730 Kraus WE, Houmard JA, Duscha BD, Knetzger KJ, Wharton MB, McCartney JS*, et al.* Effects of the amount and intensity of exercise on plasma lipoproteins. *New England Journal of Medicine*. 2002; 347: 1483-92.
731 Kristeller JL, Jordan KD. Mindful eating: Connecting with the wise self, the spiritual self. *Frontiers in Psychology*. 2018; 9.
732 Kristensen M, Pelletier X, Ross AB, Thielecke F. A High Rate of Non-Compliance Confounds the Study of Whole Grains and Weight Maintenance in a Randomised Intervention Trial-The Case for Greater Use of Dietary Biomarkers in Nutrition Intervention Studies. *Nutrients*. 2017; 9.
733 Krotkiewski M, Toss L, Björntorp P, Holm G. The effect of a very-low-calorie diet with and without chronic exercise on thyroid and sex hormones, plasma proteins, oxygen uptake, insulin and c peptide concentrations in obese women. *International Journal of Obesity*. 1981; 5: 287-93.
734 Krukowski RA, Harvey-Berino J, Bursac Z, Ashikaga T, West DS. Patterns of success: online self-monitoring in a web-based behavioral weight control program. *Health Psychology*. 2013; 32: 164-70.
735 Krukowski RA, Lueders NK, Prewitt TE, Williams DK, West DS. Obesity treatment tailored for a Catholic faith community: a feasibility study. *Journal of Health Psychology*. 2010; 15: 382-90.
736 Krukowski RA, Tilford JM, Harvey-Berino J, West DS. Comparing behavioral weight loss modalities: Incremental cost-effectiveness of an Internet-based versus an in-person condition. *Obesity*. 2011; 19: 1629-35.
737 Kucharska A, Gajewska D, Kiedrowski M, Sinska B, Juszczyk G, Czerw A*, et al.* The impact of individualised nutritional therapy according to DASH diet on blood pressure, body mass, and selected biochemical parameters in overweight/obese patients with primary arterial hypertension: a prospective randomised study. *Kardiologia Polska*. 2018; 76: 158-65.

738 Kuller LH, Gabriel KKP, Kinzel LS, Underwood DA, Conroy MB, Chang Y*, et al.* The Women on the Move Through Activity and Nutrition (WOMAN) Study: Final 48-month results. *Obesity*. 2012; 20: 636-43.
739 Kumanyika SK, Espeland MA, Bahnson JL, Bottom JB, Charleston JB, Folmar S*, et al.* Ethnic comparison of weight loss in the Trial of Nonpharmacologic Interventions in the Elderly. *Obesity Research*. 2002; 10: 96-106.
740 Kumanyika SK, Fassbender JE, Sarwer DB, Phipps E, Allison KC, Localio R*, et al.* One-year results of the Think Health! study of weight management in primary care practices. *Obesity*. 2012; 20: 1249-57.
741 Kumanyika SK, Morales KH, Allison KC, Russell Localio A, Sarwer DB, Phipps E*, et al.* Two-Year Results of Think Health! ¡Vive Saludable!: a Primary Care Weight-Management Trial. *Obesity (Silver Spring, Md)*. 2018; 26: 1412-21.
742 Kuna ST, Reboussin DM, Borradaile KE, Sanders MH, Millman RP, Zammit G*, et al.* Long-term effect of weight loss on obstructive sleep apnea severity in obese patients with type 2 diabetes. *Sleep*. 2013; 36: 641-49A.
743 Kurtzman GW, Day SC, Small DS, Lynch M, Zhu J, Wang W*, et al.* Social Incentives and Gamification to Promote Weight Loss: the LOSE IT Randomized, Controlled Trial. *Journal of General Internal Medicine*. 2018; 33: 1669-75.
744 Kushner RF, Blatner DJ, Jewell DE, Rudloff K. The PPET Study: people and pets exercising together. *Obesity*. 2006; 14: 1762-70.
745 Laaksonen DE, Nuutinen J, Lahtinen T, Rissanen A, Niskanen LK. Changes in abdominal subcutaneous fat water content with rapid weight loss and long-term weight maintenance in abdominally obese men and women. *International journal of obesity and related metabolic disorders*. 2003; 27: 677-83.
746 Laaksonen DE, Toppinen LK, Juntunen KS, Autio K, Liukkonen KH, Poutanen KS*, et al.* Dietary carbohydrate modification enhances insulin secretion in persons with the metabolic syndrome. *American Journal of Clinical Nutrition*. 2005; 82: 1218-27.
747 Laing BY, Mangione CM, Tseng CH, Leng M, Vaisberg E, Mahida M*, et al.* Effectiveness of a smartphone application for weight loss compared with usual care in overweight primary care patients: a randomized, controlled trial. *Annals of Internal Medicine*. 2014; 161: S5-12.
748 Laitinen J, Uusitupa M, Ahola I, Siitonen O. Metabolic and dietary determinants of serum lipids in obese patients with recently diagnosed non-insulin-dependent diabetes. *Annals of Medicine*. 1994; 26: 119-24.
749 Lally P, Beeken RJ, Wilson R, Omar R, Hunter R, Fovargue S*, et al.* A manualised weight management programme for adults with mild–moderate intellectual disabilities affected by excess weight: A randomised controlled feasibility trial (Shape Up-LD). *Journal of Applied Research in Intellectual Disabilities*. 2022; 35: 112-22.
750 Lambers S, Van Laethem C, Van Acker K, Calders P. Influence of combined exercise training on indices of obesity, diabetes and cardiovascular risk in type 2 diabetes patients. *Clinical Rehabilitation*. 2008; 22: 483-92.
751 Lantz H, Peltonen M, Agren L, Torgerson JS. A dietary and behavioural programme for the treatment of obesity. A 4-year clinical trial and a long-term posttreatment follow-up. *Journal of Internal Medicine*. 2003; 254: 272-79.
752 Larsen LH, Angquist L, Vimaleswaran KS, Hager J, Viguerie N, Loos RJ*, et al.* Analyses of single nucleotide polymorphisms in selected nutrient-sensitive genes in weight-regain prevention: the DIOGENES study. *American Journal of Clinical Nutrition*. 2012; 95: 1254-60.

753 Larsen RN, Mann NJ, Maclean E, Shaw JE. The effect of high-protein, low-carbohydrate diets in the treatment of type 2 diabetes: a 12 month randomised controlled trial. *Diabetologia*. 2011; 54: 731-40.
754 Latka RN, Alvarez-Reeves M, Cadmus L, Irwin ML. Adherence to a randomized controlled trial of aerobic exercise in breast cancer survivors: the Yale exercise and survivorship study. *Journal of Cancer Survivorship*. 2009; 3: 148-57.
755 Laukkanen O, Pihlajamaki J, Lindstrom J, Eriksson J, Valle TT, Hamalainen H*, et al.* Common polymorphisms in the genes regulating the early insulin signalling pathway: Effects on weight and the conversion from impaired glucose tolerance to Type 2 diabetes. The finnish diabetes prevention study. *Diabetologia*. 2004; 47: 871-77.
756 Le T, Flatt SW, Natarajan L, Pakiz B, Quintana EL, Heath DD*, et al.* Effects of Diet Composition and Insulin Resistance Status on Plasma Lipid Levels in a Weight Loss Intervention in Women. *Journal of the American Heart Association*. 2016; 5: 25.
757 Leader NJ, Ryan L, Molyneaux L, Yue DK. How best to use partial meal replacement in managing overweight or obese patients with poorly controlled type 2 diabetes. *Obesity*. 2013; 21: 251-53.
758 Leahey TM, Huedo-Medina TB, Grenga A, Gay L, Fernandes D, Denmat Z*, et al.* Patient-provided e-support in reduced intensity obesity treatment: The INSPIRE randomized controlled trial. *Health Psychology*. 2020; 39: 1037-47.
759 Leahey TM, Subak LL, Fava J, Schembri M, Thomas G, Xu X*, et al.* Benefits of adding small financial incentives or optional group meetings to a web-based statewide obesity initiative. *Obesity (Silver Spring, Md)*. 2015; 23: 70-76.
760 Leahey TM, Thomas G, Fava JL, Subak LL, Schembri M, Krupel K*, et al.* Adding evidence-based behavioral weight loss strategies to a statewide wellness campaign: a randomized clinical trial. *American Journal of Public Health*. 2014; 104: 1300-06.
761 Leahey TM, Wing RR. A randomized controlled pilot study testing three types of health coaches for obesity treatment: Professional, peer, and mentor. *Obesity*. 2013; 21: 928-34.
762 Ledikwe JH, Rolls BJ, Smiciklas-Wright H, Mitchell DC, Ard JD, Champagne C*, et al.* Reductions in dietary energy density are associated with weight loss in overweight and obese participants in the PREMIER trial. *American Journal of Clinical Nutrition*. 2007; 85: 1212-21.
763 Lee CH, Cheung B, Yi GH, Oh B, Oh YH. Mobile health, physical activity, and obesity: Subanalysis of a randomized controlled trial. *Medicine*. 2018; 97: e12309.
764 Lee K, Lee J, Bae WK, Choi JK, Kim HJ, Cho B. Efficacy of low-calorie, partial meal replacement diet plans on weight and abdominal fat in obese subjects with metabolic syndrome: a double-blind, randomised controlled trial of two diet plans - one high in protein and one nutritionally balanced. *International Journal of Clinical Practice*. 2009; 63: 195-201.
765 Lee K, Sami N, Tripathy D, Demark-Wahnefried W, Norris MK, Courneya KS*, et al.* Aerobic and resistance exercise improves Reynolds risk score in overweight or obese breast cancer survivors. *Cardio-Oncology*. 2020; 6: 27.
766 Lee S, Schorr E, Chi C-L, Treat-Jacobson D, Mathiason MA, Lindquist R. Peer group and text message–based weight-loss and management intervention for African American women. *Western Journal of Nursing Research*. 2018; 40: 1203-19.
767 Lee YJ, Lee A, Yoo HJ, Kim M, Jee SH, Shin DY*, et al.* Effect of weight loss on circulating fatty acid profiles in overweight subjects with high visceral fat area: a 12-week randomized controlled trial. *Nutrition Journal*. 2018; 17.

768 Lee YK, Cho SY, Roh HT. Effects of 16 Weeks of Taekwondo Training on the Cerebral Blood Flow Velocity, Circulating Neurotransmitters, and Subjective Well-Being of Obese Postmenopausal Women. *International Journal of Environmental Research and Public Health*. 2021; 18.
769 Lee YP, Mori TA, Puddey IB, Sipsas S, Ackland TR, Beilin LJ*, et al.* Effects of lupin kernel flour-enriched bread on blood pressure: a controlled intervention study. *American Journal of Clinical Nutrition*. 2009; 89: 766-72.
770 Leehey DJ, Collins E, Kramer HJ, Cooper C, Butler J, McBurney C*, et al.* Structured exercise in obese diabetic patients with chronic kidney disease: a randomized controlled trial (NCT01036490). *American Journal of Kidney Diseases*. 2016; 67: A67-.
771 Leehey DJ, Moinuddin I, Bast JP, Qureshi S, Jelinek CS, Cooper C*, et al.* Aerobic exercise in obese diabetic patients with chronic kidney disease: a randomized and controlled pilot study. *Cardiovascular Diabetology*. 2009; 8: 62.
772 Leermakers EA, Perri MG, Shigaki CL, Fuller PR. Effects of exercise-focused versus weight-focused maintenance programs on the management of obesity. *Addictive Behaviors*. 1999; 24: 219-27.
773 Leichtle AB, Helmschrodt C, Ceglarek U, Shai I, Henkin Y, Schwarzfuchs D*, et al.* Effects of a 2-y dietary weight-loss intervention on cholesterol metabolism in moderately obese men. *American Journal of Clinical Nutrition*. 2011; 94: 1189-95.
774 Levy RL, Jeffery RW, Langer SL, Graham DJ, Welsh EM, Flood AP*, et al.* Maintenance-tailored therapy vs standard behavior therapy for 30-month maintenance of weight loss. *Preventive Medicine: An International Journal Devoted to Practice and Theory*. 2010; 51: 457-59.
775 Lewis E, Huang H-CC, Hassmén P, Welvaert M, Pumpa KL. Adding telephone and text support to an obesity management program improves behavioral adherence and clinical outcomes A randomized controlled crossover trial. *International Journal of Behavioral Medicine*. 2019; 26: 580-90.
776 Li B, Liu LB, Liu XY, Wang YP. Effect of moderate-intensity exercise on the insulin sensitivity and plasma level of adiponectin in obese patients with type 2 diabetes mellitus. [Chinese]. *Chinese journal of clinical rehabilitation*. 2005; 9: 22-24.
777 Li X, Cai X, Ma X, Jing L, Gu J, Bao L*, et al.* Short- and Long-Term Effects of Wholegrain Oat Intake on Weight Management and Glucolipid Metabolism in Overweight Type-2 Diabetics: a Randomized Control Trial. *Nutrients*. 2016; 8.
778 Li Z, Hong K, Saltsman P, DeShields S, Bellman M, Thames G*, et al.* Long-term efficacy of soy-based meal replacements vs an individualized diet plan in obese type II DM patients: relative effects on weight loss, metabolic parameters, and C-reactive protein. *European Journal of Clinical Nutrition*. 2005; 59: 411-18.
779 Li Z, Song R, Nguyen C, Zerlin A, Karp H, Naowamondhol K*, et al.* Pistachio nuts reduce triglycerides and body weight by comparison to refined carbohydrate snack in obese subjects on a 12-week weight loss program. *Journal of the American College of Nutrition*. 2010; 29: 198-203.
780 Liao CD, Chiu YS, Ku JW, Huang SW, Liou TH. Effects of Elastic Resistance Exercise on Postoperative Outcomes Linked to the ICF Core Sets for Osteoarthritis after Total Knee Replacement in Overweight and Obese Older Women with Sarcopenia Risk: A Randomized Controlled Trial. *Journal of Clinical Medicine*. 2020; 9: 15.
781 Liao CD, Tsauo JY, Huang SW, Ku JW, Hsiao DJ, Liou TH. Effects of elastic band exercise on lean mass and physical capacity in older women with sarcopenic obesity: A randomized controlled trial. *Scientific Reports*. 2018; 8: 2317.

782 Liao D, Asberry PJ, Shofer JB, Callahan H, Matthys C, Boyko EJ*, et al.* Improvement of BMI, body composition, and body fat distribution with lifestyle modification in Japanese Americans with impaired glucose tolerance. *Diabetes Care*. 2002; 25: 1504-10.
783 Ligibel JA, Campbell N, Partridge A, Chen WY, Salinardi T, Chen H*, et al.* Impact of a mixed strength and endurance exercise intervention on insulin levels in breast cancer survivors. *Journal of Clinical Oncology*. 2008; 26: 907-12.
784 Ligibel JA, Giobbie-Hurder A, Olenczuk D, Campbell N, Salinardi T, Winer EP*, et al.* Impact of a mixed strength and endurance exercise intervention on levels of adiponectin, high molecular weight adiponectin and leptin in breast cancer survivors. *Cancer Causes & Control*. 2009; 20: 1523-28.
785 Lillis J, Niemeier HM, Thomas JG, Unick J, Ross KM, Leahey TM*, et al.* A randomized trial of an acceptance-based behavioral intervention for weight loss in people with high internal disinhibition. *Obesity*. 2016; 24: 2509-14.
786 Lillis J, Thomas JG, Niemeier HM, Wing RR. Exploring process variables through which acceptance-based behavioral interventions may improve weight loss maintenance. *Journal of Contextual Behavioral Science*. 2017; 6: 398-403.
787 Lim SL, Loo WM, Ong KW, Ng ZL, Han C, Chan YH*, et al.* Lifestyle intervention enabled by mobile technologies leading to weight loss in patients with non-alcoholic fatty liver disease: a randomized controlled trial. *Journal of gastroenterology and hepatology*. 2018; 33: 94-.
788 Lin M, Mahmooth Z, Dedhia N, Frutchey R, Mercado CE, Epstein DH*, et al.* Tailored, interactive text messages for enhancing weight loss among African American adults: the TRIMM randomized controlled trial. *American Journal of Medicine*. 2015; 128: 896-904.
789 Lin PH, Wang Y, Levine E, Askew S, Lin S, Chang C*, et al.* A text messaging-assisted randomized lifestyle weight loss clinical trial among overweight adults in Beijing. *Obesity*. 2014; 22: E29-E37.
790 Lin X, Qi Q, Zheng Y, Huang T, Lathrop M, Zelenika D*, et al.* Neuropeptide Y genotype, central obesity, and abdominal fat distribution: the POUNDS LOST trial. *American Journal of Clinical Nutrition*. 2015; 102: 514-19.
791 Lindahl B, Nilsson TK, Jansson JH, Asplund K, Hallmans G. Improved fibrinolysis by intense lifestyle intervention. A randomized trial in subjects with impaired glucose tolerance. *Journal of Internal Medicine*. 1999; 246: 105-12.
792 Linde JA, Jeffery RW, Levy RL, Pronk NP, Boyle RG. Weight loss goals and treatment outcomes among overweight men and women enrolled in a weight loss trial. *International Journal of Obesity*. 2005; 29: 1002-05.
793 Lipkin EW, Schwartz AV, Anderson AM, Davis C, Johnson KC, Gregg EW*, et al.* The Look AHEAD Trial: bone loss at 4-year follow-up in type 2 diabetes. *Diabetes Care*. 2014; 37: 2822-29.
794 Lisevick A, Cartmel B, Harrigan M, Li F, Sanft T, Fogarasi M*, et al.* Effect of the Lifestyle, Exercise, and Nutrition (LEAN) Study on Long-Term Weight Loss Maintenance in Women with Breast Cancer. *Nutrients*. 2021; 13: 18.
795 Little P, Stuart B, Hobbs FR, Kelly J, Smith ER, Bradbury KJ*, et al.* Randomised controlled trial and economic analysis of an internet-based weight management programme: POWeR+ (Positive Online Weight Reduction). *Health Technology Assessment (Winchester, England)*. 2017; 21: 1-62.
796 Littman AJ, Bertram LC, Ceballos R, Ulrich CM, Ramaprasad J, McGregor B*, et al.* Randomized controlled pilot trial of yoga in overweight and obese breast cancer survivors:

effects on quality of life and anthropometric measures. *Supportive Care in Cancer*. 2012; 20: 267-77.
797 Liu X, Vitetta L, Kostner K, Crompton D, Williams G, Brown WJ*, et al.* The Effects of Tai Chi in Centrally Obese Adults with Depression Symptoms. *Evidence-based Complementary & Alternative Medicine (eCAM)*. 2015; 2015: 1-8.
798 Liu X, Williams G, Kostner K, Brown WJ. The Effect of t'ai chi on Quality of Life in Centrally Obese Adults with Depression. *Journal of Alternative & Complementary Medicine*. 2019; 25: 1005-08.
799 Livingstone KM, Celis-Morales C, Navas-Carretero S, San-Cristobal R, Forster H, Woolhead C*, et al.* Characteristics of participants who benefit most from personalised nutrition: findings from the pan-European Food4Me randomised controlled trial. *British Journal of Nutrition*. 2020: 1-10.
800 Löberbauer-Purer E, Meyer NL, Ring-Dimitriou S, Haudum J, Kässmann H, Müller E. Can alternating lower body negative and positive pressure during exercise alter regional body fat distribution or skin appearance? *European Journal of Applied Physiology*. 2012; 112: 1861-71.
801 Loeser RF, Beavers DP, Bay-Jensen AC, Karsdal MA, Nicklas BJ, Guermazi A*, et al.* Effects of dietary weight loss with and without exercise on interstitial matrix turnover and tissue inflammation biomarkers in adults with knee osteoarthritis: the Intensive Diet and Exercise for Arthritis trial (IDEA). *Osteoarthritis & Cartilage*. 2017; 25: 1822-28.
802 Logue E, Sutton K, Jarjoura D, Smucker W, Baughman K, Capers C. Transtheoretical model-chronic disease care for obesity in primary care: a randomized trial. *Obesity Research*. 2005; 13: 917-27.
803 Logue EE, Bourguet CC, Palmieri PA, Scott ED, Matthews BA, Dudley P*, et al.* The better weight-better sleep study: a pilot intervention in primary care. *American Journal of Health Behavior*. 2012; 36: 319-34.
804 Look ARG. Eight-year weight losses with an intensive lifestyle intervention: the look AHEAD study. *Obesity*. 2014; 22: 5-13.
805 Look ARG. Prospective Association of GLUL rs10911021 With Cardiovascular Morbidity and Mortality Among Individuals With Type 2 Diabetes: The Look AHEAD Study. *Diabetes*. 2016; 65: 297-302.
806 Look ARG, Greenway FL. Severe hypoglycemia in the Look AHEAD Trial. *Journal of Diabetes & its Complications*. 2016; 30: 935-43.
807 Lowe MR, Butryn ML, Thomas JG, Coletta M. Meal replacements, reduced energy density eating, and weight loss maintenance in primary care patients: a randomized controlled trial. *Obesity*. 2014; 22: 94-100.
808 Lowe MR, Butryn ML, Zhang F. Evaluation of meal replacements and a home food environment intervention for long-term weight loss: a randomized controlled trial. *American Journal of Clinical Nutrition*. 2018; 107: 12-19.
809 Lowe MR, Tappe KA, Annunziato RA, Riddell LJ, Coletta MC, Crerand CE*, et al.* The effect of training in reduced energy density eating and food self-monitoring accuracy on weight loss maintenance. *Obesity*. 2008; 16: 2016-23.
810 Lowndes J, Kawiecki D, Pardo S, Nguyen V, Melanson KJ, Yu Z*, et al.* The effects of four hypocaloric diets containing different levels of sucrose or high fructose corn syrup on weight loss and related parameters. *Nutrition Journal*. 2012; 11: 55.
811 Luley C, Blaik A, Götz A, Kicherer F, Kropf S, Isermann B*, et al.* Weight loss by telemonitoring of nutrition and physical activity in patients with metabolic syndrome for 1 year. *Journal of the American College of Nutrition*. 2014; 33: 363-74.

812 Lundgren JR, Janus C, Jensen SBK, Juhl CR, Olsen LM, Christensen RM*, et al.* Healthy weight loss maintenance with exercise, liraglutide, or both combined. *New England Journal of Medicine*. 2021; 384(18): 1719-30.
813 Lunt H, Draper N, Marshall HC, Logan FJ, Hamlin MJ, Shearman JP*, et al.* High intensity interval training in a real world setting: a randomized controlled feasibility study in overweight inactive adults, measuring change in maximal oxygen uptake. *PloS one*. 2014; 9: e83256.
814 Luscombe ND, Clifton PM, Noakes M, Parker B, Wittert G. Effects of energy-restricted diets containing increased protein on weight loss, resting energy expenditure, and the thermic effect of feeding in type 2 diabetes. *Diabetes Care*. 2002; 25: 652-57.
815 Luscombe-Marsh ND, Noakes M, Wittert GA, Keogh JB, Foster P, Clifton PM. Carbohydrate-restricted diets high in either monounsaturated fat or protein are equally effective at promoting fat loss and improving blood lipids. *American Journal of Clinical Nutrition*. 2005; 81: 762-72.
816 Lutes LD, Damschroder LJ, Masheb R, Kim HM, Gillon L, Holleman RG*, et al.* Behavioral Treatment for Veterans with Obesity: 24-Month Weight Outcomes from the ASPIRE-VA Small Changes Randomized Trial. *Journal of General Internal Medicine*. 2017; 32: 40-47.
817 Lutes LD, Winett RA, Barger SD, Wojcik JR, Herbert WG, Nickols-Richardson SM*, et al.* Small changes in nutrition and physical activity promote weight loss and maintenance: 3-month evidence from the ASPIRE randomized trial. *Annals of Behavioral Medicine*. 2008; 35: 351-57.
818 Lystrup R, Carlsen D, Sharon DJ, Crawford P. Wearable and interactive technology to share fitness goals results in weight loss but not improved diabetes outcomes. *Obesity Research and Clinical Practice*. 2020; 14: 443-48.
819 Ma J, King AC, Wilson SR, Xiao L, Stafford RS. Evaluation of lifestyle interventions to treat elevated cardiometabolic risk in primary care (E-LITE): a randomized controlled trial. *BMC Family Practice*. 2009; 10: 71.
820 Ma W, Huang T, Wang M, Zheng Y, Wang T, Heianza Y*, et al.* Two-year changes in circulating adiponectin, ectopic fat distribution and body composition in response to weight-loss diets: the POUNDS Lost Trial. *International journal of obesity (2005)*. 2016; 40: 1723-29.
821 Ma W, Huang T, Zheng Y, Wang M, Bray GA, Sacks FM*, et al.* Weight-Loss Diets, Adiponectin, and Changes in Cardiometabolic Risk in the 2-Year POUNDS Lost Trial. *Journal of clinical endocrinology and metabolism*. 2016; 101: 2415-22.
822 Madigan CD, Aveyard P, Jolly K, Denley J, Lewis A, Daley AJ. Regular self-weighing to promote weight maintenance after intentional weight loss: a quasi-randomized controlled trial. *Journal of Public Health*. 2014; 36: 259-67.
823 Madigan CD, Jolly K, Lewis AL, Aveyard P, Daley AJ. A randomised controlled trial of the effectiveness of self-weighing as a weight loss intervention. *International Journal of Behavioral Nutrition & Physical Activity*. 2014; 11: 125.
824 Madrid DA, Beavers KM, Walkup MP, Ambrosius WT, Rejeski WJ, Marsh AP*, et al.* Effect of exercise modality and weight loss on changes in muscle and bone quality in older adults with obesity. *Experimental Gerontology*. 2023; 174: 112126.
825 Madrona Marcos F, Panisello Royo JM, Tarraga Marcos ML, Rosich N, Carbayo Herencia JA, Alins J*, et al.* Effect of a motivational physical activity program on lipid parameters in patients with obesity and overweight. *Clinica e Investigacion en Arteriosclerosis*. 2019; 31: 245-50.

826 Maeda H, Yamamoto R, Hirao K, Tochikubo O. Effects of agar (kanten) diet on obese patients with impaired glucose tolerance and type 2 diabetes. *Diabetes, Obesity and Metabolism*. 2005; 7: 40-46.
827 Mager U, Kolehmainen M, de Mello VD, Schwab U, Laaksonen DE, Rauramaa R*, et al.* Expression of ghrelin gene in peripheral blood mononuclear cells and plasma ghrelin concentrations in patients with metabolic syndrome. *European Journal of Endocrinology*. 2008; 158: 499-510.
828 Maki KC, Rains TM, Kaden VN, Raneri KR, Davidson MH. Effects of a reduced-glycemic-load diet on body weight, body composition, and cardiovascular disease risk markers in overweight and obese adults. *American Journal of Clinical Nutrition*. 2007; 85: 724-34.
829 Maltais ML, Perreault K, Courchesne-Loyer A, Lagace JC, Barsalani R, Dionne IJ. Effect of Resistance Training and Various Sources of Protein Supplementation on Body Fat Mass and Metabolic Profile in Sarcopenic Overweight Older Adult Men: A Pilot Study. *International Journal of Sport Nutrition & Exercise Metabolism*. 2016; 26: 71-77.
830 Mangeri F, Montesi L, Forlani G, Grave RD, Marchesini G. A standard ballroom and Latin dance program to improve fitness and adherence to physical activity in individuals with type 2 diabetes and in obesity. *Diabetology and Metabolic Syndrome*. 2014; 6.
831 Mann S, Jimenez A, Steele J, Domone S, Wade M, Beedie C. Programming and supervision of resistance training leads to positive effects on strength and body composition: results from two randomised trials of community fitness programmes. *BMC Public Health*. 2018; 18: 420.
832 Manzoni GM, Cesa GL, Bacchetta M, Castelnuovo G, Conti S, Gaggioli A*, et al.* Virtual reality–enhanced cognitive–behavioral therapy for morbid obesity: A randomized controlled study with 1 year follow-up. *Cyberpsychology, Behavior, and Social Networking*. 2016; 19: 134-40.
833 Marcos-Pardo PJ, Gonzalez-Galvez N, Gea-Garcia GM, Lopez-Vivancos A, Espeso-Garcia A, Vale RGD. Sarcopenia as a Mediator of the Effect of a Gerontogymnastics Program on Cardiorespiratory Fitness of Overweight and Obese Older Women: A Randomized Controlled Trial. *International Journal of Environmental Research and Public Health*. 2020; 17: 15.
834 Marcos-Pardo PJ, Orquin-Castrillon FJ, Gea-Garcia GM, Menayo-Antunez R, Gonzalez-Galvez N, Vale RGD*, et al.* Effects of a moderate-to-high intensity resistance circuit training on fat mass, functional capacity, muscular strength, and quality of life in elderly: A randomized controlled trial. *Scientific Reports*. 2019; 9: 12.
835 Marks BL, Ward A, Morris DH, Castellani J, Rippe JM. Fat-free mass is maintained in women following a moderate diet and exercise program. *Medicine and science in sports and exercise*. 1995; 27: 1243-51.
836 Marniemi J, Seppänen A, Hakala P. Long-term effects on lipid metabolism of weight reduction on lactovegetarian and mixed diet. *International Journal of Obesity*. 1990; 14: 113-25.
837 Marrero DG, Palmer KN, Phillips EO, Miller-Kovach K, Foster GD, Saha CK. Comparison of Commercial and Self-Initiated Weight Loss Programs in People With Prediabetes: A Randomized Control Trial. *American Journal of Public Health*. 2016; 106: 949-56.
838 Martin CK, Anton SD, Han H, York-Crowe E, Redman LM, Ravussin E*, et al.* Examination of cognitive function during six months of calorie restriction: results of a randomized controlled trial. *Rejuvenation Research*. 2007; 10: 179-90.

839 Martin PD, Dutton GR, Brantley PJ. Self-Efficacy as a Predictor of Weight Change in African-American Women. *Obesity Research*. 2004; 12: 646-51.
840 Martin PD, Dutton GR, Rhode PC, Horswell RL, Ryan DH, Brantley PJ. Weight loss maintenance following a primary care intervention for low-income minority women. *Obesity*. 2008; 16: 2462-67.
841 Martin-Piedra L, Alcala-Diaz JF, Gutierrez-Mariscal FM, Arenas de Larriva AP, Romero-Cabrera JL, Torres-Pena JD*, et al.* Evolution of Metabolic Phenotypes of Obesity in Coronary Patients after 5 Years of Dietary Intervention: From the CORDIOPREV Study. *Nutrients*. 2021; 13: 12.
842 Martinez-Lapiscina E, Clavero P, Toledo E, San Julian B, Sanchez-Tainta A, Corella D*, et al.* Virgin olive oil supplementation and long-term cognition: the Predimed-Navarra randomized, trial. *Journal of Nutrition, Health & Aging*. 2013; 17: 544-52.
843 Martínez-Lapiscina EH, Clavero P, Toledo E, Estruch R, Salas-Salvadó J, Julián BS*, et al.* Mediterranean diet improves cognition: The PREDIMED-NAVARRA randomised trial. *Journal of Neurology, Neurosurgery & Psychiatry*. 2013; 84: 1318-25.
844 Mascola AJ, Yiaslas TA, Meir RL, McGee SM, Downing NL, Beaver KM*, et al.* Framing physical activity as a distinct and uniquely valuable behavior independent of weight management: A pilot randomized controlled trial from overweight and obese sedentary persons. *Eating and weight disorders*. 2009; 14: e148-e52.
845 Masheb RM, Grilo CM, Rolls BJ. A randomized controlled trial for obesity and binge eating disorder: low-energy-density dietary counseling and cognitive-behavioral therapy. *Behaviour research and therapy*. 2011; 49: 821-29.
846 Masheb RM, Lutes LD, Kim HM, Holleman RG, Goodrich DE, Janney CA*, et al.* Weight loss outcomes in patients with pain. *Obesity*. 2015; 23: 1778-84.
847 Mason AE, Epel ES, Aschbacher K, Lustig RH, Acree M, Kristeller J*, et al.* Reduced reward-driven eating accounts for the impact of a mindfulness-based diet and exercise intervention on weight loss: data from the SHINE randomized controlled trial. *Appetite*. 2016; 100: 86-93.
848 Mason AE, Hecht FM, Daubenmier JJ, Sbarra DA, Lin J, Moran PJ*, et al.* Weight Loss Maintenance and Cellular Aging in the Supporting Health Through Nutrition and Exercise Study. *Psychosomatic Medicine*. 2018; 80: 609-19.
849 Masuo K, Rakugi H, Ogihara T, Lambert GW. Different mechanisms in weight loss-induced blood pressure reduction between a calorie-restricted diet and exercise. *Hypertension research*. 2012; 35: 41-47.
850 Mateo KF. Evaluation of Intermittent Energy Restriction and Continuous Energy Restriction on Weight Loss and Blood Pressure Control in Overweight and Obese Patients With Hypertension. *Journal of Clinical Outcomes Management*. 2021; 28: 256-59.
851 Mateo-Gallego R, Lamiquiz-Moneo I, Perez-Calahorra S, Marco-Benedí V, Bea AM, Baila-Rueda L*, et al.* Different protein composition of low-calorie diet differently impacts adipokine profile irrespective of weight loss in overweight and obese women. *Nutrition, metabolism, and cardiovascular diseases : NMCD*. 2018; 28: 133-42.
852 Matsuse H, Segal NA, Rabe KG, Shiba N. The Effect of Neuromuscular Electrical Stimulation During Walking on Muscle Strength and Knee Pain in Obese Women With Knee Pain: A Randomized Controlled Trial. *American Journal of Physical Medicine & Rehabilitation*. 2020; 99: 56-64.
853 Mattei J, Qi Q, Hu FB, Sacks FM, Qi L. TCF7L2 genetic variants modulate the effect of dietary fat intake on changes in body composition during a weight-loss intervention. *American Journal of Clinical Nutrition*. 2012; 96: 1129-36.

854 Mavros Y, Kay S, Simpson KA, Baker MK, Wang Y, Zhao RR*, et al.* Reductions in C-reactive protein in older adults with type 2 diabetes are related to improvements in body composition following a randomized controlled trial of resistance training. *Journal of Cachexia, Sarcopenia and Muscle*. 2014; 5: 111-20.
855 Mayer VL, Vangeepuram N, Fei K, Hanlen-Rosado EA, Arniella G, Negron R*, et al.* Outcomes of a Weight Loss Intervention to Prevent Diabetes Among Low-Income Residents of East Harlem, New York. *Health Education & Behavior*. 2019; 46: 1073-82.
856 Mayer-Davis EJ, D'Antonio AM, Smith SM, Kirkner G, Levin Martin S, Parra-Medina D*, et al.* Pounds off with empowerment (POWER): a clinical trial of weight management strategies for black and white adults with diabetes who live in medically underserved rural communities. *American Journal of Public Health*. 2004; 94: 1736-42.
857 Mazini Filho ML, Macedo Vianna J, de Oliveira Venturini GR, de Matos DG, Caputo Ferreira ME. Efeitos de diferentes programas de exercícios físicos na pressão arterial e indicadores antropométricos de idosas. *Motricidade*. 2016; 12: 147-54.
858 McAuley KA, Hopkins CM, Smith KJ, McLay RT, Williams SM, Taylor RW*, et al.* Comparison of high-fat and high-protein diets with a high-carbohydrate diet in insulin-resistant obese women. *Diabetologia*. 2005; 48: 8-16.
859 McCaffery JM, Anderson A, Coday M, Espeland MA, Gorin AA, Johnson KC*, et al.* Loneliness Relates to Functional Mobility in Older Adults with Type 2 Diabetes: The Look AHEAD Study. *Journal of Aging Research*. 2020: 1-8.
860 McClernon FJ, Yancy WS, Jr., Eberstein JA, Atkins RC, Westman EC. The effects of a low-carbohydrate ketogenic diet and a low-fat diet on mood, hunger, and other self-reported symptoms. *Obesity*. 2007; 15: 182-87.
861 McConnon A, Horgan GW, Lawton C, Stubbs J, Shepherd R, Astrup A*, et al.* Experience and acceptability of diets of varying protein content and glycemic index in an obese cohort: results from the Diogenes trial. *European Journal of Clinical Nutrition*. 2013; 67: 990-95.
862 McConnon A, Kirk SF, Cockroft JE, Harvey EL, Greenwood DC, Thomas JD*, et al.* The Internet for weight control in an obese sample: results of a randomised controlled trial. *BMC Health Services Research*. 2007; 7: 206.
863 McEvoy CT, Wallace IR, Hamill LL, Hunter SJ, Neville CE, Patterson CC*, et al.* Increasing Fruit and Vegetable Intake Has No Dose-Response Effect on Conventional Cardiovascular Risk Factors in Overweight Adults at High Risk of Developing Cardiovascular Disease. *Journal of Nutrition*. 2015; 145: 1464-71.
864 McEvoy CT, Wallace IR, Hamill LL, Neville CE, Hunter SJ, Patterson CC*, et al.* Increasing fruit and vegetable intake has no effect on retinal vessel caliber in adults at high risk of developing cardiovascular disease. *Nutrition Metabolism & Cardiovascular Diseases*. 2016; 26: 318-25.
865 McLaughlin T, Carter S, Lamendola C, Abbasi F, Schaaf P, Basina M*, et al.* Clinical efficacy of two hypocaloric diets that vary in overweight patients with type 2 diabetes: comparison of moderate fat versus carbohydrate reductions. *Diabetes Care*. 2007; 30: 1877-79.
866 McLaughlin T, Carter S, Lamendola C, Abbasi F, Yee G, Schaaf P*, et al.* Effects of moderate variations in macronutrient composition on weight loss and reduction in cardiovascular disease risk in obese, insulin-resistant adults. *American Journal of Clinical Nutrition*. 2006; 84: 813-21.
867 McManus K, Antinoro L, Sacks F. A randomized controlled trial of a moderate-fat, low-energy diet compared with a low fat, low-energy diet for weight loss in overweight adults. *International Journal of Obesity*. 2001; 25: 1503-11.

868 McNabb W, Quinn M, Kerver J, Cook S, Karrison T. The PATHWAYS church-based weight loss program for urban African-American women at risk for diabetes. *Diabetes Care*. 1997; 20: 1518-23.
869 Meckling KA, Sherfey R. A randomized trial of a hypocaloric high-protein diet, with and without exercise, on weight loss, fitness, and markers of the Metabolic Syndrome in overweight and obese women. *Physiologie appliquee, nutrition ET metabolisme [applied physiology, nutrition, and metabolism]*. 2007; 32: 743-52.
870 Mediano MF, Barbosa JS, Sichieri R, Pereira RA. Effects of exercise on insulin sensitivity in obese women submitted to a weight loss program: a clinical trial. *Arquivos Brasileiros de Endocrinologia e Metabologia*. 2007; 51: 993-99.
871 Medina-Remon A, Tresserra-Rimbau A, Pons A, Tur JA, Martorell M, Ros E*, et al.* Effects of total dietary polyphenols on plasma nitric oxide and blood pressure in a high cardiovascular risk cohort. The PREDIMED randomized trial. *Nutrition Metabolism & Cardiovascular Diseases*. 2015; 25: 60-67.
872 Meekums B, Vaverniece I, Majore-Dusele I, Rasnacs O. Dance movement therapy for obese women with emotional eating: A controlled pilot study. *The Arts in Psychotherapy*. 2012; 39: 126-33.
873 Mefferd K, Nichols JF, Pakiz B, Rock CL. A cognitive behavioral therapy intervention to promote weight loss improves body composition and blood lipid profiles among overweight breast cancer survivors. *Breast Cancer Research & Treatment*. 2007; 104: 145-52.
874 Megakli T, Vlachopoulos SP, Theodorakis Y. Effects of an aerobic and resistance exercise intervention on health-related quality of life in women with obesity. *Journal of Applied Biobehavioral Research*. 2016; 21: 82-106.
875 Megakli T, Vlachopoulos SP, Thøgersen-Ntoumani C, Theodorakis Y. Impact of aerobic and resistance exercise combination on physical self-perceptions and self-esteem in women with obesity with one-year follow-up. *International Journal of Sport and Exercise Psychology*. 2017; 15: 236-57.
876 Mehrabani HH, Salehpour S, Amiri Z, Farahani SJ, Meyer BJ, Tahbaz F. Beneficial effects of a high-protein, low-glycemic-load hypocaloric diet in overweight and obese women with polycystic ovary syndrome: a randomized controlled intervention study. *Journal of the American College of Nutrition*. 2012; 31: 117-25.
877 Melchart D, Low P, Wuhr E, Kehl V, Weidenhammer W. Effects of a tailored lifestyle self-management intervention (TALENT) study on weight reduction: a randomized controlled trial. *Diabetes, metabolic syndrome and obesity: targets and therapy*. 2017; 10: 235-45.
878 Mellor DD, Whitham C, Goodwin S, Morris M, Reid M, Atkin SL. Weight loss in a UK commercial all meal provision study: a randomised controlled trial. *Journal of Human Nutrition & Dietetics*. 2014; 27: 377-83.
879 Memelink RG, Pasman WJ, Bongers A, Tump A, van Ginkel A, Tromp W*, et al.* Effect of an Enriched Protein Drink on Muscle Mass and Glycemic Control during Combined Lifestyle Intervention in Older Adults with Obesity and Type 2 Diabetes: A Double-Blind RCT. *Nutrients*. 2021; 13: 64-64.
880 Meneses ME, Camargo A, Perez-Martinez P, Delgado-Lista J, Cruz-Teno C, Jimenez-Gomez Y*, et al.* Postprandial inflammatory response in adipose tissue of patients with metabolic syndrome after the intake of different dietary models. *Molecular Nutrition & Food Research*. 2011; 55: 1759-70.
881 Mengham LH, Morris BF, Palmer CR, White AJS. Is intensive dietetic intervention effective for overweight patients with diabetes mellitus? A randomised controlled study in a general practice. *Practical diabetes international*. 1999; 16: 5-8.

882 Messier SP, Beavers DP, Mihalko SL, Miller GD, Lyles MF, Hunter DJ*, et al.* The effects of intensive dietary weight loss and exercise on gait in overweight and obese adults with knee osteoarthritis. The Intensive Diet and Exercise for Arthritis (IDEA) trial. *Journal of Biomechanics*. 2020; 98: 109477.
883 Messier SP, Loeser RF, Miller GD, Morgan TM, Rejeski WJ, Sevick MA*, et al.* Exercise and dietary weight loss in overweight and obese older adults with knee osteoarthritis: the Arthritis, Diet, and Activity Promotion Trial. *Arthritis & Rheumatism*. 2004; 50: 1501-10.
884 Messier SP, Loeser RF, Mitchell MN, Valle G, Morgan TP, Rejeski WJ*, et al.* Exercise and weight loss in obese older adults with knee osteoarthritis: a preliminary study. *Journal of the American Geriatrics Society*. 2000; 48: 1062-72.
885 Messier SP, Resnik AE, Beavers DP, Mihalko SL, Miller GD, Nicklas BJ*, et al.* Intentional Weight Loss in Overweight and Obese Patients With Knee Osteoarthritis: is More Better? *Arthritis Care & Research*. 2018; 70: 1569-75.
886 Metz JA, Stern JS, Kris-Etherton P, Reusser ME, Morris CD, Hatton DC*, et al.* A randomized trial of improved weight loss with a prepared meal plan in overweight and obese patients: impact on cardiovascular risk reduction. *Archives of Internal Medicine*. 2000; 160: 2150-58.
887 Mewes I, Stich A, Habermüller M-S, Revenstorf D. Gewichtsreduktion unter Hypnose und Verhaltenstherapie = Weight reduction by hypnosis and behavior therapy. *Verhaltenstherapie & Verhaltensmedizin*. 2003; 24: 499-512.
888 Micco N, Gold B, Buzzell P, Leonard H, Pintauro S, Harvey-Berino J. Minimal in-person support as an adjunct to internet obesity treatment. *Annals of Behavioral Medicine*. 2007; 33: 49-56.
889 Miedema B, Reading SA, Hamilton RA, Morrison KS, Thompson AE. Can certified health professionals treat obesity in a community-based programme? A quasi-experimental study. *BMJ Open*. 2015; 5.
890 Mihalko SL, Cox P, Beavers DP, Miller GD, Nicklas BJ, Lyles M*, et al.* Effect of intensive diet and exercise on self-efficacy in overweight and obese adults with knee osteoarthritis: The IDEA randomized clinical trial. *Translational Behavioral Medicine*. 2019; 9: 227-35.
891 Miklavcic JJ, Fraser KD, Ploeg J, Markle-Reid M, Fisher K, Gafni A*, et al.* Effectiveness of a community program for older adults with type 2 diabetes and multimorbidity: a pragmatic randomized controlled trial. *BMC Geriatrics*. 2020; 20: 14.
892 Miller EG, Sethi P, Nowson CA, Dunstan DW, Daly RM. Effects of progressive resistance training and weight loss versus weight loss alone on inflammatory and endothelial biomarkers in older adults with type 2 diabetes. *European Journal of Applied Physiology*. 2017; 117: 1669-78.
893 Miller GD, Beavers DP, Hamm D, Mihalko SL, Messier SP. Nutrient Intake During Diet-Induced Weight Loss and Exercise Interventions in a Randomized Trial in Older Overweight and Obese Adults. *Journal of Nutrition, Health & Aging*. 2017; 21: 1216-24.
894 Miller GD, Nicklas BJ, Davis CC, Ambrosius WT, Loeser RF, Messier SP. Is serum leptin related to physical function and is it modifiable through weight loss and exercise in older adults with knee osteoarthritis? *International Journal of Obesity & Related Metabolic Disorders: Journal of the International Association for the Study of Obesity*. 2004; 28: 1383-90.
895 Miller GD, Nicklas BJ, Davis CC, Legault C, Messier SP. Basal growth hormone concentration increased following a weight loss focused dietary intervention in older overweight and obese women. *Journal of Nutrition, Health & Aging*. 2012; 16: 169-74.

896 Millward DJ, Truby H, Fox KR, Livingstone MB, Macdonald IA, Tothill P. Sex differences in the composition of weight gain and loss in overweight and obese adults. *British Journal of Nutrition*. 2014; 111: 933-43.
897 Milsom VA, Malcolm RJ, Johnson GC, Pechon SM, Gray KM, Miller-Kovach K*, et al.* Changes in cardiovascular risk factors with participation in a 12-week weight loss trial using a commercial format. *Eating Behaviors*. 2014; 15: 68-71.
898 Mirkarimi K, Mostafavi F, Eshghinia S, Vakili MA, Ozouni-Davaji RB, Aryaie M. Effect of motivational interviewing on a weight loss program based on the protection motivation theory. *Iranian Red Crescent Medical Journal*. 2015; 17.
899 Mirzaei K, Xu M, Qi Q, de Jonge L, Bray GA, Sacks F*, et al.* Variants in glucose- and circadian rhythm-related genes affect the response of energy expenditure to weight-loss diets: the POUNDS LOST Trial. *American Journal of Clinical Nutrition*. 2014; 99: 392-99.
900 Mitchell NS, Manning BK, Staton EW, Emsermann CD, Dickinson LM, Pace WD. Outcomes of biomarker feedback on physical activity, eating habits, and emotional health: from the Americans in Motion-Healthy Intervention (AIM-HI) study. *Journal of the American Board of Family Medicine*. 2014; 27: 61-69.
901 Mitra SR, Tan PY. Effect of an individualised high-protein, energy-restricted diet on anthropometric and cardio-metabolic parameters in overweight and obese Malaysian adults: a 6-month randomised controlled study. *British Journal of Nutrition*. 2019; 121: 1002-17.
902 Mohamad H, Ntessalen M, Craig LCA, Clark J, Fielding S, N'Dow J*, et al.* A self-help diet and physical activity intervention with dietetic support for weight management in men treated for prostate cancer: Pilot study of the Prostate Cancer Weight Management (PRO-MAN) randomised controlled trial. *British Journal of Nutrition*. 2019; 122: 592-600.
903 Mohammad-Shahi M, Haidari F, Karandish M, Ebrahimi S, Haghighizadeh MH. The effect of nutritional education on inflammatory markers and the index of diet quality in obese women. [Persian]. *Journal of zanjan university of medical sciences and health services*. 2015; 23: 126-40.
904 Mohammadi M, Kargarfard M. The effect of 12 weeks of aerobic exercise with and without caloric restriction on visfatin plasma levels and metabolic indicators in elderly postmenopausal women. [Persian]. *Journal of isfahan medical school*. 2016; 34: 848-56.
905 Mohler ER, Sibley AA, Stein R, Davila-Roman V, Wyatt H, Badellino K*, et al.* Endothelial function and weight loss: comparison of low-carbohydrate and low-fat diets. *Obesity (Silver Spring, Md)*. 2013; 21: 504-09.
906 Mojtahedi MC, Thorpe MP, Karampinos DC, Johnson CL, Layman DK, Georgiadis JG*, et al.* The effects of a higher protein intake during energy restriction on changes in body composition and physical function in older women. *Journals of Gerontology Series A-Biological Sciences & Medical Sciences*. 2011; 66: 1218-25.
907 Molenaar EA, van Ameijden EJ, Vergouwe Y, Grobbee DE, Numans ME. Effect of nutritional counselling and nutritional plus exercise counselling in overweight adults: A randomized trial in multidisciplinary primary care practice. *Family Practice*. 2010; 27: 143-50.
908 Moncrieft AE, Llabre MM, McCalla JR, Gutt M, Mendez AJ, Gellman MD*, et al.* Effects of a Multicomponent Life-Style Intervention on Weight, Glycemic Control, Depressive Symptoms, and Renal Function in Low-Income, Minority Patients With Type 2 Diabetes: Results of the Community Approach to Lifestyle Modification for Diabetes Randomized Controlled Trial. *Psychosomatic Medicine*. 2016; 78: 851-60.
909 Moore CS, Bryant SP, Mishra GD, Krebs JD, Browning LM, Miller GJ*, et al.* Oily fish reduces plasma triacylglycerols: a primary prevention study in overweight men and women. *Nutrition*. 2006; 22: 1012-24.

910 Moore RH, Diewald LK, Wadden TA, Sarwer DB, Berkowitz RI, Chittams J*, et al.* The impact of a primary care-based weight loss intervention on the quality of life. *International Journal of Obesity*. 2013; 37: S25-S30.
911 Mora-Rodriguez R, Fernandez-Elias VE, Morales-Palomo F, Pallares JG, Ramirez-Jimenez M, Ortega JF. Aerobic interval training reduces vascular resistances during submaximal exercise in obese metabolic syndrome individuals. *European Journal of Applied Physiology*. 2017; 117: 2065-73.
912 Moradi Kelardeh B, Rahmati-Ahmadabad S, Farzanegi P, Helalizadeh M, Azarbayjani M-A. Effects of non-linear resistance training and curcumin supplementation on the liver biochemical markers levels and structure in older women with non-alcoholic fatty liver disease. *Journal of Bodywork & Movement Therapies*. 2020; 24: 154-60.
913 Morales E, Valero Ma A, Leon M, Hernandez E, Praga M. Beneficial effects of weight loss in overweight patients with chronic proteinuric nephropathies. *American Journal of Kidney Diseases*. 2003; 41: 319-27.
914 Morales-Palomo F, Ramirez-Jimenez M, Ortega JF, Mora-Rodriguez R. Exercise Periodization over the Year Improves Metabolic Syndrome and Medication Use. *Medicine and science in sports and exercise*. 2018; 50: 1983-91.
915 Moran LJ, Luscombe-Marsh ND, Noakes M, Wittert GA, Keogh JB, Clifton PM. The satiating effect of dietary protein is unrelated to postprandial ghrelin secretion. *Journal of clinical endocrinology and metabolism*. 2005; 90: 5205-11.
916 Morato-Martínez M, Santurino C, López-Plaza B, Arcos-Castellanos L, Clavero-Fraile M, Palma-Milla S*, et al.* Un programa integral estandarizado de intervención nutricional y actividad física reduce el peso corporal en mujeres recién diagnosticadas de cáncer de mama. *Nutricion Hospitalaria*. 2021; 38: 575-84.
917 Moreno B, Bellido D, Sajoux I, Goday A, Saavedra D, Crujeiras AB*, et al.* Comparison of a very low-calorie-ketogenic diet with a standard low-calorie diet in the treatment of obesity. *Endocrine*. 2014; 47: 793-805.
918 Moreno B, Crujeiras AB, Bellido D, Sajoux I, Casanueva FF. Obesity treatment by very low-calorie-ketogenic diet at two years: reduction in visceral fat and on the burden of disease. *Endocrine*. 2016; 54: 681-90.
919 Morey MC, Blair CK, Sloane R, Cohen HJ, Snyder DC, Demark-Wahnefried W. Group trajectory analysis helps to identify older cancer survivors who benefit from distance-based lifestyle interventions. *Cancer*. 2015; 121: 4433-40.
920 Morey MC, Pieper CF, Edelman DE, Yancy WS, Green JB, Lum H*, et al.* Enhanced fitness: a randomized controlled trial of the effects of home-based physical activity counseling on glycemic control in older adults with prediabetes mellitus. *Journal of the American Geriatrics Society*. 2012; 60: 1655-62.
921 Morey MC, Snyder DC, Sloane R, Cohen HJ, Peterson B, Hartman TJ*, et al.* Effects of home-based diet and exercise on functional outcomes among older, overweight long-term cancer survivors: RENEW: A randomized controlled trial. *JAMA - journal of the american medical association*. 2009; 301: 1883-89.
922 Morgan PJ, Callister R, Collins CE, Plotnikoff RC, Young MD, Berry N*, et al.* The SHED-IT community trial: a randomized controlled trial of internet- and paper-based weight loss programs tailored for overweight and obese men. *Annals of Behavioral Medicine*. 2013; 45: 139-52.
923 Morgan PJ, Scott HA, Young MD, Plotnikoff RC, Collins CE, Callister R. Associations between program outcomes and adherence to Social Cognitive theory tasks: process

evaluation of the SHED-IT community weight loss trial for men. *International journal of behavioral nutrition and physical activity*. 2014; 11: 89.
924 Mori TA, Bao DQ, Burke V, Puddey IB, Watts GF, Beilin LJ. Dietary fish as a major component of a weight-loss diet: effect on serum lipids, glucose, and insulin metabolism in overweight hypertensive subjects. *American Journal of Clinical Nutrition*. 1999; 70: 817-25.
925 Mori TA, Burke V, Puddey IB, Shaw JE, Beilin LJ. Effect of fish diets and weight loss on serum leptin concentration in overweight, treated-hypertensive subjects. *Journal of Hypertension*. 2004; 22: 1983-90.
926 Moss J, Tew GA, Copeland RJ, Stout M, Billings CG, Saxton JM*, et al.* Effects of a pragmatic lifestyle intervention for reducing body mass in obese adults with obstructive sleep apnoea: A randomised controlled trial. *BioMed Research International*. 2014; 2014 (no pagination).
927 Motie M, Evangelista LS, Lombardo D, Hoi J, Horwich TB, Hamilton M*, et al.* Effect of weight loss on renal function in overweight and obese patients with heart failure. *Diabetes & Metabolic Syndrome*. 2017; 11: 95-98.
928 Muller-Pinget S, Allet L, Golay A. Body as subject, body as object: How treatment of the obese patient can be improved by dance therapy. *Education Thérapeutique du Patient / Therapeutic Patient Education*. 2018; 10: 1-5.
929 Munsch S, Biedert E, Meyer A, Michael T, Schlup B, Tuch A*, et al.* A randomized comparison of cognitive behavioral therapy and behavioral weight loss treatment for overweight individuals with binge eating disorder. *International Journal of Eating Disorders*. 2007; 40: 102-13.
930 Munsch S, Meyer AH, Biedert E. Efficacy and predictors of long-term treatment success for Cognitive-Behavioral Treatment and Behavioral Weight-Loss-Treatment in overweight individuals with binge eating disorder. *Behaviour Research & Therapy*. 2012; 50: 775-85.
931 Munugoda IP, Beavers DP, Wirth W, Aitken DA, Loeser RF, Miller GD*, et al.* The effect of weight loss on the progression of meniscal extrusion and size in knee osteoarthritis: a post-hoc analysis of the Intensive Diet and Exercise for Arthritis (IDEA) trial. *Osteoarthritis & Cartilage*. 2020; 28: 410-17.
932 Muollo V, Rossi AP, Milanese C, Masciocchi E, Taylor M, Zamboni M*, et al.* The effects of exercise and diet program in overweight people - Nordic walking versus walking. *Clinical Interventions In Aging*. 2019; 14: 1555-65.
933 Muollo V, Rossi AP, Milanese C, Zamboni M, Rosa R, Schena F*, et al.* Prolonged unsupervised Nordic walking and walking exercise following six months of supervision in adults with overweight and obesity: A randomised clinical trial. *Nutrition, Metabolism and Cardiovascular Diseases*. 2021; 31(4): 1247-56.
934 Murakami T, Horigome H, Tanaka K, Nakata Y, Katayama Y, Matsui A. Effects of diet with or without exercise on leptin and anticoagulation proteins levels in obesity. *Blood Coagulation & Fibrinolysis*. 2007; 18: 389-94.
935 Muralidharan A, Brown CH, Zhang Y, Niv N, Cohen AN, Kreyenbuhl J*, et al.* Quality of life outcomes of web-based and in-person weight management for adults with serious mental illness. *Journal of Behavioral Medicine*. 2020; 43: 865-72.
936 Murphy CM, Rohsenow DJ, Johnson KC, Wing RR. Smoking and weight loss among smokers with overweight and obesity in Look AHEAD. *Health Psychology*. 2018; 37: 399-406.
937 Murphy KJ, Thomson RL, Coates AM, Buckley JD, Howe PR. Effects of eating fresh lean pork on cardiometabolic health parameters. *Nutrients*. 2012; 4: 711-23.

938 Muzio F, Mondazzi L, Harris WS, Sommariva D, Branchi A. Effects of moderate variations in the macronutrient content of the diet on cardiovascular disease risk factors in obese patients with the metabolic syndrome. *American Journal of Clinical Nutrition*. 2007; 86: 946-51.
939 Myers VH, McVay MA, Brashear MM, Johnson WD, Gupta AK, Brantley PJ*, et al.* Five-year medical and pharmacy costs after a medically supervised intensive treatment program for obesity. *American Journal of Health Promotion*. 2014; 28: 364-71.
940 Nabuco HCG, Tomeleri CM, Fernandes RR, Sugihara Junior P, Cavalcante EF, Venturini D*, et al.* Effects of Protein Intake Beyond Habitual Intakes Associated With Resistance Training on Metabolic Syndrome-Related Parameters, Isokinetic Strength, and Body Composition in Older Women. *Journal of Aging & Physical Activity*. 2019; 27: 545-52.
941 Nackers LM, Middleton KR, Dubyak PJ, Daniels MJ, Anton SD, Perri MG. Effects of prescribing 1,000 versus 1,500 kilocalories per day in the behavioral treatment of obesity: a randomized trial. *Obesity*. 2013; 21: 2481-87.
942 Nakade M, Aiba N, Suda N, Morita A, Miyachi M, Sasaki S*, et al.* Behavioral change during weight loss program and one-year follow-up: saku Control Obesity Program (SCOP) in Japan. *Asia Pacific Journal of Clinical Nutrition*. 2012; 21: 22-34.
943 Naldi L, Conti A, Cazzaniga S, Patrizi A, Pazzaglia M, Lanzoni A*, et al.* Diet and physical exercise in psoriasis: a randomized controlled trial. *British Journal of Dermatology*. 2014; 170: 634-42.
944 Navas-Carretero S, Holst C, Saris WH, van Baak MA, Jebb SA, Kafatos A*, et al.* The Impact of Gender and Protein Intake on the Success of Weight Maintenance and Associated Cardiovascular Risk Benefits, Independent of the Mode of Food Provision: the DiOGenes Randomized Trial. *Journal of the American College of Nutrition*. 2016; 35: 20-30.
945 Neiberg RH, Rejeski JJ, Applegate WB, Clark JM, Knowler WC, Bray GA*, et al.* Self-reported gastrointestinal symptoms in type 2 diabetes improve with an intensive lifestyle intervention: results from the action for health in diabetes (Look AHEAD) clinical trial. *Clinical diabetes*. 2015; 33: 181-88.
946 Neuendorf R, Corn J, Hanes D, Bradley R. Impact of Food Immunoglobulin G-Based Elimination Diet on Subsequent Food Immunoglobulin G and Quality of Life in Overweight/Obese Adults. *Journal of Alternative & Complementary Medicine*. 2019; 25: 241-48.
947 Neumark-Sztainer D, Kaufmann NA, Berry EM. Physical activity within a community-based weight control program: program evaluation and predictors of success. *Public Health Reviews*. 1995; 23: 237-51.
948 Ng CF, Yee CH, So WY, Yip SK, Wu E, Yau P. Effect of weight reduction on severity of lower urinary tract symptoms in obese men with benign prostatic hyperplasia. *Hong Kong Medical Journal*. 2017; 23 Suppl 2: 35-37.
949 Ng SSS, Chan RSM, Woo J, Chan TO, Cheung BHK, Sea MMM*, et al.* A Randomized Controlled Study to Examine the Effect of a Lifestyle Modification Program in OSA. *Chest*. 2015; 148: 1193-203.
950 Ng TW, Chan DC, Barrett PH, Watts GF. Effect of weight loss on HDL-apoA-II kinetics in the metabolic syndrome. *Clinical science (London, England : 1979)*. 2009; 118: 79-85.
951 Ngo Njembe MT, Pachikian B, Lobysheva I, Van Overstraeten N, Dejonghe L, Verstraelen E*, et al.* A Three-Month Consumption of Eggs Enriched with omega-3, omega-5 and omega-7 Polyunsaturated Fatty Acids Significantly Decreases the Waist Circumference of Subjects at Risk of Developing Metabolic Syndrome: A Double-Blind Randomized Controlled Trial. *Nutrients*. 2021; 13: 18.

952 Nicklas BJ, Ambrosius W, Messier SP, Miller GD, Penninx BW, Loeser RF*, et al.* Diet-induced weight loss, exercise, and chronic inflammation in older, obese adults: a randomized controlled clinical trial. *American Journal of Clinical Nutrition*. 2004; 79: 544-51.
953 Nicklas BJ, Chmelo E, Delbono O, Carr JJ, Lyles MF, Marsh AP. Effects of resistance training with and without caloric restriction on physical function and mobility in overweight and obese older adults: a randomized controlled trial. *American Journal of Clinical Nutrition*. 2015; 101: 991-99.
954 Nicklas BJ, Mychaleckyj J, Kritchevsky S, Palla S, Lange LA, Lange EM*, et al.* Physical function and its response to exercise: associations with cytokine gene variation in older adults with knee osteoarthritis. *Journals of Gerontology Series A-Biological Sciences & Medical Sciences*. 2005; 60: 1292-98.
955 Nicklas JM, Sacks FM, Smith SR, LeBoff MS, Rood JC, Bray GA*, et al.* Effect of dietary composition of weight loss diets on high-sensitivity c-reactive protein: the Randomized POUNDS LOST trial. *Obesity*. 2013; 21: 681-89.
956 Nicolaï SP, Kruidenier LM, Leffers P, Hardeman R, Hidding A, Teijink JA. Supervised exercise versus non-supervised exercise for reducing weight in obese adults. *Journal of sports medicine and physical fitness*. 2009; 49: 85-90.
957 Nielsen JV, Jonsson E, Nilsson AK. Lasting improvement of hyperglycaemia and bodyweight: low-carbohydrate diet in type 2 diabetes. A brief report. *Upsala Journal of Medical Sciences*. 2005; 110: 179-83.
958 Nikseresht M. Comparison of Serum Cytokine Levels in Men Who are Obese or Men Who are Lean: effects of Nonlinear Periodized Resistance Training and Obesity. *Journal of strength and conditioning research*. 2018; 32: 1787-95.
959 Niswender K, Piletic M, Andersen H, Conradsen Hiort L, Hollander P. Weight change upon once-daily initiation of insulin detemir with or without dietary intervention in overweight or obese insulin-naïve individuals with type 2 diabetes: results from the DIET trial. *Diabetes, Obesity & Metabolism*. 2014; 16: 186-92.
960 Niv N, Cohen AN, Hamilton A, Reist C, Young AS. Effectiveness of a psychosocial weight management program for individuals with schizophrenia. *The Journal of Behavioral Health Services & Research*. 2014; 41: 370-80.
961 Njerve IU, Byrkjeland R, Arnesen H, Akra S, Solheim S, Seljeflot I. Effects of long-term exercise training on adipose tissue expression of fractalkine and MCP-1 in patients with type 2 diabetes and stable coronary artery disease: a substudy of a randomized controlled trial. *Diabetes, metabolic syndrome and obesity: targets and therapy*. 2016; 9: 55-62.
962 Njike VY, Ayettey R, Petraro P, Treu JA, Katz DL. Walnut ingestion in adults at risk for diabetes: effects on body composition, diet quality, and cardiac risk measures. *BMJ open diabetes research and care*. 2015; 3.
963 Njike VY, Kavak Y, Treu JA, Doughty K, Katz DL. Snacking, Satiety, and Weight: A Randomized, Controlled Trial. *American Journal of Health Promotion*. 2017; 31: 296-301.
964 Noakes M, Foster PR, Keogh JB, Clifton PM. Meal replacements are as effective as structured weight-loss diets for treating obesity in adults with features of metabolic syndrome. *Journal of Nutrition*. 2004; 134: 1894-99.
965 Nordby P, Rosenkilde M, Ploug T, Westh K, Feigh M, Nielsen NB*, et al.* Independent effects of endurance training and weight loss on peak fat oxidation in moderately overweight men: a randomized controlled trial. *Journal of applied physiology (Bethesda, Md : 1985)*. 2015; 118: 803-10.
966 Norman GJ, Kolodziejczyk JK, Adams MA, Patrick K, Marshall SJ. Fruit and vegetable intake and eating behaviors mediate the effect of a randomized text-message based weight

loss program. *Preventive Medicine: An International Journal Devoted to Practice and Theory*. 2013; 56: 3-7.
967 Normandin E, Chmelo E, Lyles MF, Marsh AP, Nicklas BJ. Effect of Resistance Training and Caloric Restriction on the Metabolic Syndrome. *Medicine & Science in Sports & Exercise*. 2017; 49: 413-19.
968 Nosrani SE, Tartibian B, Eslami R, Farinha C, Serrano J, Ferreira JP*, et al.* The Effects of Combined Aquatic Exercise on Physical Performance and Metabolic Indices in Overweight Healthy Older Adults. *International Journal of Exercise Science*. 2023; 16: 1499-513.
969 Nurkkala M, Kaikkonen K, Vanhala ML, Karhunen L, Keranen AM, Korpelainen R. Lifestyle intervention has a beneficial effect on eating behavior and long-term weight loss in obese adults. *Eating Behaviors*. 2015; 18: 179-85.
970 O'Brien KM, Wiggers J, Williams A, Campbell E, Hodder RK, Wolfenden L*, et al.* Telephone-based weight loss support for patients with knee osteoarthritis: a pragmatic randomised controlled trial. *Osteoarthritis and cartilage*. 2018; 26: 485-94.
971 O'Neil PM, Miller-Kovach K, Tuerk PW, Becker LE, Wadden TA, Fujioka K*, et al.* Randomized controlled trial of a nationally available weight control program tailored for adults with type 2 diabetes. *Obesity*. 2016; 24: 2269-77.
972 Okura T, Nakata Y, Tanaka K. Effects of exercise intensity on physical fitness and risk factors for coronary heart disease. *Obesity Research*. 2003; 11: 1131-39.
973 Oláh M, Koncz Á, Fehér J, Kálmánczhey J, Oláh C, Nagy G*, et al.* The effect of balneotherapy on antioxidant, inflammatory, and metabolic indices in patients with cardiovascular risk factors (hypertension and obesity)--a randomised, controlled, follow-up study. *Contemporary Clinical Trials*. 2011; 32: 793-801.
974 Ortner Hadziabdic M, Vitali Cepo D, Rahelic D, Bozikov V. The Effect of the Mediterranean Diet on Serum Total Antioxidant Capacity in Obese Patients: A Randomized Controlled Trial. *Journal of the American College of Nutrition*. 2016; 35: 224-35.
975 Osama AJ, Shehab AK. Psychological wellbeing and biochemical modulation in response to weight loss in obese type 2 diabetes patients. *African historical studies*. 2015; 15: 503-12.
976 Oshakbayev K, Dukenbayeva B, Otarbayev N, Togizbayeva G, Tabynbayev N, Gazaliyeva M*, et al.* Weight loss therapy for clinical management of patients with some atherosclerotic diseases: a randomized clinical trial. *Nutrition Journal*. 2015; 14: 120.
977 Oshima Y, Matsuoka Y, Sakane N. Effect of weight-loss program using self-weighing twice a day and feedback in overweight and obese subject: a randomized controlled trial. *Obesity Research & Clinical Practice*. 2013; 7: e361-66.
978 Otten J, Andersson J, Ståhl J, Stomby A, Saleh A, Waling M*, et al.* Exercise Training Adds Cardiometabolic Benefits of a Paleolithic Diet in Type 2 Diabetes Mellitus. *Journal of the American Heart Association*. 2019; 8: e010634.
979 Özbey Ü, Balaban S, Sözener ZÇ, Uçar A, Mungan D, Mısırlıgil Z. The effects of diet-induced weight loss on asthma control and quality of life in obese adults with asthma: A randomized controlled trial. *Journal of Asthma*. 2020; 57: 618-26.
980 Pacanowski CR, Levitsky DA. Frequent Self-Weighing and Visual Feedback for Weight Loss in Overweight Adults. *Journal of Obesity*. 2015; 2015: 763680.
981 Pacanowski CR, Linde JA, Faulconbridge LF, Coday M, Safford MM, Chen H*, et al.* Psychological status and weight variability over eight years: Results from Look AHEAD. *Health Psychology*. 2018; 37: 238-46.

982 Pacanowski CR, Mason TB, Crosby RD, Mitchell JE, Crow SJ, Wonderlich SA*, et al.* Weight change over the course of binge eating disorder treatment: Relationship to binge episodes and psychological factors. *Obesity*. 2018; 26: 838-44.
983 Paisey RB, Frost J, Harvey P, Paisey A, Bower L, Paisey RM*, et al.* Five year results of a prospective very low calorie diet or conventional weight loss programme in type 2 diabetes. *Journal of Human Nutrition & Dietetics*. 2002; 15: 121-27.
984 Pal S, Khossousi A, Binns C, Dhaliwal S, Ellis V. The effect of a fibre supplement compared to a healthy diet on body composition, lipids, glucose, insulin and other metabolic syndrome risk factors in overweight and obese individuals. *British Journal of Nutrition*. 2011; 105: 90-100.
985 Palmeira CS, Mussi FC, Santos CASdT, Lima ML, Ladeia AMT, Silva LCdJ. Effect of remote nursing monitoring on overweight in women: clinical trial. *Revista latino-americana de enfermagem*. 2019; 27.
986 Papadaki A, Linardakis M, Plada M, Larsen TM, Damsgaard CT, van Baak MA*, et al.* Impact of weight loss and maintenance with ad libitum diets varying in protein and glycemic index content on metabolic syndrome. *Nutrition*. 2014; 30: 410-17.
987 Papadaki A, Martínez-González MÁ, Alonso-Gómez A, Rekondo J, Salas-Salvadó J, Corella D*, et al.* Mediterranean diet and risk of heart failure: results from the PREDIMED randomized controlled trial. *European Journal of Heart Failure*. 2017; 19: 1179-85.
988 Papakonstantinou E, Kontogianni MD, Mitrou P, Magriplis E, Vassiliadi D, Nomikos T*, et al.* Effects of 6 vs 3 eucaloric meal patterns on glycaemic control and satiety in people with impaired glucose tolerance or overt type 2 diabetes: a randomized trial. *Diabetes & Metabolism*. 2018; 44: 226-34.
989 Papalazarou A, Yannakoulia M, Kavouras SA, Komesidou V, Dimitriadis G, Papakonstantinou A*, et al.* Lifestyle intervention favorably affects weight loss and maintenance following obesity surgery. *Obesity*. 2010; 18: 1348-53.
990 Papandonatos GD, Pan Q, Pajewski NM, Delahanty LM, Peter I, Erar B*, et al.* Genetic Predisposition to Weight Loss and Regain With Lifestyle Intervention: analyses From the Diabetes Prevention Program and the Look AHEAD Randomized Controlled Trials. *Diabetes*. 2015; 64: 4312-21.
991 Papandreou C, Becerra-Tomas N, Bullo M, Martinez-Gonzalez MA, Corella D, Estruch R*, et al.* Legume consumption and risk of all-cause, cardiovascular, and cancer mortality in the PREDIMED study. *Clinical Nutrition*. 2019; 38: 348-56.
992 Papandreou C, Schiza SE, Bouloukaki I, Hatzis CM, Kafatos AG, Siafakas NM*, et al.* Effect of Mediterranean diet versus prudent diet combined with physical activity on OSAS: a randomised trial. *The european respiratory journal*. 2012; 39: 1398-404.
993 Parikh P, Simon EP, Fei K, Looker H, Goytia C, Horowitz CR. Results of a pilot diabetes prevention intervention in East Harlem, New York City: Project HEED. *American Journal of Public Health*. 2010; 100 Suppl 1: S232-39.
994 Park J, Park H. Effects of 6 months of aerobic and resistance exercise training on carotid artery intima media thickness in overweight and obese older women. *Geriatrics & gerontology international*. 2017; 17: 2304-10.
995 Park S, Park J, Ham JA, Jee Y. Effects of aerobic dance with electrical stimulant on body composition and radiological circumference of obese elderly women. *Gazzetta Medica Italiana Archivio per le Scienze Mediche*. 2021; 180(3): 87-95.
996 Parker AR, Byham-Gray L, Denmark R, Winkle PJ. The effect of medical nutrition therapy by a registered dietitian nutritionist in patients with prediabetes participating in a

randomized controlled clinical research trial. *Journal of the academy of nutrition and dietetics*. 2014; 114: 1739-48.
997 Parker B, Noakes M, Luscombe N, Clifton P. Effect of a high-protein, high-monounsaturated fat weight loss diet on glycemic control and lipid levels in type 2 diabetes. *Diabetes Care*. 2002; 25: 425-30.
998 Parretti HM, Aveyard P, Blannin A, Clifford SJ, Coleman SJ, Roalfe A*, et al.* Efficacy of water preloading before main meals as a strategy for weight loss in primary care patients with obesity: RCT. *Obesity*. 2015; 23: 1785-91.
999 Parsian H, Eizadi M, Khorshidi D, Khanali F. The effect of long-term aerobic exercise on serum adiponectin and insulin sensitivity in type 2 diabetic patients. *Journal of jahrom university of medical sciences*. 2013; 11: 36-43.
1000 Pascale RW, Wing RR, Butler BA, Mullen M, Bononi P. Effects of a behavioral weight loss program stressing calorie restriction versus calorie plus fat restriction in obese individuals with NIDDM or a family history of diabetes. *Diabetes Care*. 1995; 18: 1241-48.
1001 Paskett ED, Baltic RD, Young GS, Katz ML, Lesko SM, Webber KH*, et al.* A Group Randomized Trial to Reduce Obesity among Appalachian Church Members: the Walk by Faith Study. *Cancer Epidemiology, Biomarkers & Prevention*. 2018; 27: 1289-97.
1002 Pasman WJ, Memelink RG, de Vogel-Van den Bosch J, Begieneman MPV, van den Brink WJ, Weijs PJM*, et al.* Obese Older Type 2 Diabetes Mellitus Patients with Muscle Insulin Resistance Benefit from an Enriched Protein Drink during Combined Lifestyle Intervention: The PROBE Study. *Nutrients*. 2020; 12: 2979.
1003 Patrick K, Calfas KJ, Norman GJ, Rosenberg D, Zabinski MF, Sallis JF*, et al.* Outcomes of a 12-month web-based intervention for overweight and obese men. *Annals of Behavioral Medicine*. 2011; 42: 391-401.
1004 Patrick K, Raab F, Adams MA, Dillon L, Zabinski M, Rock CL*, et al.* A text message-based intervention for weight loss: randomized controlled trial. *Journal of Medical Internet Research*. 2009; 11.
1005 Pavlou KN, Krey S, Steffee WP. Exercise as an adjunct to weight loss and maintenance in moderately obese subjects. *American Journal of Clinical Nutrition*. 1989; 49: 1115-23.
1006 Pavlou KN, Whatley JE, Jannace PW, DiBartolomeo JJ, Burrows BA, Duthie EA*, et al.* Physical activity as a supplement to a weight-loss dietary regimen. *American Journal of Clinical Nutrition*. 1989; 49: 1110-14.
1007 Pearl RL, Wadden TA, Bach C, Tronieri JS, Berkowitz RI. Six-Month Follow-up from a Randomized Controlled Trial of the Weight BIAS Program. *Obesity*. 2020; 28: 1878-88.
1008 Pedersen E, Jesudason DR, Clifton PM. High protein weight loss diets in obese subjects with type 2 diabetes mellitus. *Nutrition Metabolism & Cardiovascular Diseases*. 2014; 24: 554-62.
1009 Pedersen SD, Kang J, Kline GA. Portion control plate for weight loss in obese patients with type 2 diabetes mellitus: a controlled clinical trial. *Archives of Internal Medicine*. 2007; 167: 1277-83.
1010 Pedley CF, Case LD, Blackwell CS, Katula JA, Vitolins MZ. The 24-month metabolic benefits of the healthy living partnerships to prevent diabetes: A community-based translational study. *Diabetes & Metabolic Syndrome*. 2018; 12: 215-20.
1011 Pekmezi DW, Neighbors CJ, Lee CS, Gans KM, Bock BC, Morrow KM*, et al.* A culturally adapted physical activity intervention for Latinas: a randomized controlled trial. *American Journal of Preventive Medicine*. 2009; 37: 495-500.

1012 Pellegrini CA, Chang RW, Dunlop DD, Conroy DE, Lee J, Van Horn L*, et al.* Comparison of a Patient-Centered Weight Loss Program starting before versus after knee replacement: A pilot study. *Obesity Research & Clinical Practice*. 2018; 12: 472-78.
1013 Peña-Orihuela P, Camargo A, Rangel-Zuñiga OA, Perez-Martinez P, Cruz-Teno C, Delgado-Lista J*, et al.* Antioxidant system response is modified by dietary fat in adipose tissue of metabolic syndrome patients. *Journal of Nutritional Biochemistry*. 2013; 24: 1717-23.
1014 Perez-Diaz-Del-Campo N, Riezu-Boj JI, Marin-Alejandre BA, Monreal JI, Elorz M, Herrero JI*, et al.* A nutrigenetic tool for precision dietary management of non-alcoholic fatty liver disease deeming insulin resistance markers. *Panminerva Medica*. 2022; 64: 485-96.
1015 Perez-Huerta BD, Diaz-Pulido B, Pecos-Martin D, Beckwee D, Lluch-Girbes E, Fernandez-Matias R*, et al.* Effectiveness of a Program Combining Strengthening, Stretching, and Aerobic Training Exercises in a Standing versus a Sitting Position in Overweight Subjects with Knee Osteoarthritis: A Randomized Controlled Trial. *Journal of Clinical Medicine*. 2020; 9: 13.
1016 Perri MG, Limacher MC, Durning PE, Janicke DM, Lutes LD, Bobroff LB*, et al.* Extended-care programs for weight management in rural communities: the treatment of obesity in underserved rural settings (TOURS) randomized trial. *Archives of Internal Medicine*. 2008; 168: 2347-54.
1017 Perri MG, Limacher MC, von Castel-Roberts K, Daniels MJ, Durning PE, Janicke DM*, et al.* Comparative effectiveness of three doses of weight-loss counseling: two-year findings from the rural LITE trial. *Obesity*. 2014; 22: 2293-300.
1018 Perri MG, Martin AD, Leermakers EA, Sears SF, Notelovitz M. Effects of group- versus home-based exercise in the treatment of obesity. *Journal of consulting and clinical psychology*. 1997; 65: 278-85.
1019 Perri MG, McAdoo WG, McAllister DA, Lauer JB, Jordan RC, Yancey DZ*, et al.* Effects of peer support and therapist contact on long-term weight loss. *Journal of consulting and clinical psychology*. 1987; 55: 615-17.
1020 Perri MG, McAllister DA, Gange JJ, Jordan RC, McAdoo WG, Nezu AM. Effects of four maintenance programs on the long-term management of obesity. *Journal of consulting and clinical psychology*. 1988; 56: 529-34.
1021 Perri MG, Nezu AM, McKelvey WF, Shermer RL, Renjilian DA, Viegener BJ. Relapse prevention training and problem-solving therapy in the long-term management of obesity. *Journal of consulting and clinical psychology*. 2001; 69: 722-26.
1022 Perri MG, Nezu AM, Patti ET, McCann KL. Effect of length of treatment on weight loss. *Journal of consulting and clinical psychology*. 1989; 57: 450-52.
1023 Peters JC, Beck J, Cardel M, Wyatt HR, Foster GD, Pan Z*, et al.* The effects of water and non-nutritive sweetened beverages on weight loss and weight maintenance: A randomized clinical trial. *Obesity*. 2016; 24: 297-304.
1024 Peters JC, Wyatt HR, Foster GD, Pan Z, Wojtanowski AC, Vander Veur SS*, et al.* The effects of water and non-nutritive sweetened beverages on weight loss during a 12-week weight loss treatment program. *Obesity*. 2014; 22: 1415-21.
1025 Pettman TL, Misan GMH, Owen K, Warren K, Coates AM, Buckley JD*, et al.* Self-management for obesity and cardio-metabolic fitness: Description and evaluation of the lifestyle modification program of a randomised controlled trial. *The International Journal of Behavioral Nutrition and Physical Activity*. 2008; 5.
1026 Philippou C, Andreou E, Menelaou N, Hajigeorgiou P, Papandreou D. Effects of diet and exercise in 337 overweight/obese adults. *Hippokratia*. 2012; 16: 46-50.

1027 Philippou E, Neary NM, Chaudhri O, Brynes AE, Dornhorst A, Leeds AR*, et al.* The effect of dietary glycemic index on weight maintenance in overweight subjects: a pilot study. *Obesity*. 2009; 17: 396-401.
1028 Phimarn W, Pianchana P, Limpikanchakovit P, Suranart K, Supapanichsakul S, Narkgoen A*, et al.* Thai community pharmacist involvement in weight management in primary care to improve patient's outcomes. *International Journal of Clinical Pharmacy*. 2013; 35: 1208-17.
1029 Piacenza F, Malavolta M, Basso A, Costarelli L, Giacconi R, Ravussin E*, et al.* Effect of 6-month caloric restriction on Cu bound to ceruloplasmin in adult overweight subjects. *Journal of Nutritional Biochemistry*. 2015; 26: 876-82.
1030 Pinto AM, Subak LL, Nakagawa S, Vittinghoff E, Wing RR, Kusek JW*, et al.* The effect of weight loss on changes in health-related quality of life among overweight and obese women with urinary incontinence. *Quality of Life Research: An International Journal of Quality of Life Aspects of Treatment, Care & Rehabilitation*. 2012; 21: 1685-94.
1031 Pinto-Gouveia J, Carvalho SA, Palmeira L, Castilho P, Duarte C, Ferreira C*, et al.* BEfree: A new psychological program for binge eating that integrates psychoeducation, mindfulness, and compassion. *Clinical Psychology & Psychotherapy*. 2017; 24: 1090-98.
1032 Pjanic I, Müller R, Laimer M, Hagenbuch N, Laederach K, Stanga Z. Evaluation of a multiprofessional, nonsurgical obesity treatment program: Which parameters indicated life style changes and weight loss? *Journal of Eating Disorders*. 2017; 5.
1033 Pleticosic-Ramirez Y, Mecias-Calvo M, Arufe-Giraldez V, Navarro-Paton R. Incidence of a Multicomponent Physical Exercise Program on Body Composition in Overweight or Obese People Aged 60 Years or Older from Chile. *Journal of Functional Morphology & Kinesiology*. 2024; 9: 24.
1034 Plotnikoff RC, Eves N, Jung M, Sigal RJ, Padwal R, Karunamuni N. Multicomponent, home-based resistance training for obese adults with type 2 diabetes: A randomized controlled trial. *International Journal of Obesity*. 2010; 34: 1733-41.
1035 Poddar KH, Ames M, Hsin-Jen C, Feeney MJ, Wang Y, Cheskin LJ. Positive effect of mushrooms substituted for meat on body weight, body composition, and health parameters. A 1-year randomized clinical trial. *Appetite*. 2013; 71: 379-87.
1036 Poelman MP, de Vet E, Velema E, de Boer MR, Seidell JC, Steenhuis IHM. PortionControl@HOME: Results of a randomized controlled trial evaluating the effect of a multi-component portion size intervention on portion control behavior and body mass index. *Annals of Behavioral Medicine*. 2015; 49: 18-28.
1037 Pogacnik Murillo AL, Eckstein F, Wirth W, Beavers D, Loeser RF, Nicklas BJ*, et al.* Impact of Diet and/or Exercise Intervention on Infrapatellar Fat Pad Morphology: Secondary Analysis from the Intensive Diet and Exercise for Arthritis (IDEA) Trial. *Cells Tissues Organs*. 2017; 203: 258-66.
1038 Poppitt SD, Keogh GF, Prentice AM, Williams DE, Sonnemans HM, Valk EE*, et al.* Long-term effects of ad libitum low-fat, high-carbohydrate diets on body weight and serum lipids in overweight subjects with metabolic syndrome. *American Journal of Clinical Nutrition*. 2002; 75: 11-20.
1039 Porzelius LK, Houston C, Smith M, Arfken C, Fisher E. Comparison of a standard behavioral weight loss treatment and a binge eating weight loss treatment. *Behavior Therapy*. 1995; 26: 119-34.
1040 Poston WS, Haddock CK, Pinkston MM, Pace P, Karakoc ND, Reeves RS*, et al.* Weight loss with meal replacement and meal replacement plus snacks: a randomized trial. *International journal of obesity (2005)*. 2005; 29: 1107-14.

1041 Powers TA, Koestner R, Denes A, Cornelius T, Gorin AA. Autonomy support in a couples weight loss trial: Helping yourself while helping others. *Families, Systems, & Health*. 2022; 40: 70-78.
1042 Pownall HJ, Bray GA, Wagenknecht LE, Walkup MP, Heshka S, Hubbard VS*, et al.* Changes in body composition over 8 years in a randomized trial of a lifestyle intervention: the look AHEAD study. *Obesity*. 2015; 23: 565-72.
1043 Prather AA, Blumenthal JA, Hinderliter AL, Sherwood A. Ethnic differences in the effects of the DASH diet on nocturnal blood pressure dipping in individuals with high blood pressure. *American Journal of Hypertension*. 2011; 24: 1338-44.
1044 Prehn K, Lesemann A, Krey G, Witte AV, Kobe T, Grittner U*, et al.* Using resting-state fMRI to assess the effect of aerobic exercise on functional connectivity of the DLPFC in older overweight adults. *Brain and Cognition*. 2019; 131: 34-44.
1045 Preuss H, Pinnow M, Schnicker K, Legenbauer T. Improving inhibitory control abilities (ImpulsE)—A promising approach to treat impulsive eating? *European Eating Disorders Review*. 2017; 25: 533-43.
1046 Promrat K, Kleiner DE, Niemeier HM, Jackvony E, Kearns M, Wands JR*, et al.* Randomized controlled trial testing the effects of weight loss on nonalcoholic steatohepatitis. *Hepatology (Baltimore, Md)*. 2010; 51: 121-29.
1047 Ptomey LT, Saunders RR, Saunders M, Washburn RA, Mayo MS, Sullivan DK*, et al.* Weight management in adults with intellectual and developmental disabilities: A randomized controlled trial of two dietary approaches. *Journal of Applied Research in Intellectual Disabilities*. 2018; 31: 82-96.
1048 Qi Q, Bray GA, Hu FB, Sacks FM, Qi L. Weight-loss diets modify glucose-dependent insulinotropic polypeptide receptor rs2287019 genotype effects on changes in body weight, fasting glucose, and insulin resistance: the Preventing Overweight Using Novel Dietary Strategies trial. *American Journal of Clinical Nutrition*. 2012; 95: 506-13.
1049 Qi Q, Bray GA, Smith SR, Hu FB, Sacks FM, Qi L. Insulin receptor substrate 1 gene variation modifies insulin resistance response to weight-loss diets in a 2-year randomized trial: the Preventing Overweight Using Novel Dietary Strategies (POUNDS LOST) trial. *Circulation*. 2011; 124: 563-71.
1050 Qi Q, Durst R, Schwarzfuchs D, Leitersdorf E, Shpitzen S, Li Y*, et al.* CETP genotype and changes in lipid levels in response to weight-loss diet intervention in the POUNDS LOST and DIRECT randomized trials. *Journal of Lipid Research*. 2015; 56: 713-21.
1051 Qi Q, Zheng Y, Huang T, Rood J, Bray GA, Sacks FM*, et al.* Vitamin D metabolism-related genetic variants, dietary protein intake and improvement of insulin resistance in a 2 year weight-loss trial: POUNDS Lost. *Diabetologia*. 2015; 58: 2791-99.
1052 Raatz SK, Torkelson CJ, Redmon JB, Reck KP, Kwong CA, Swanson JE*, et al.* Reduced glycemic index and glycemic load diets do not increase the effects of energy restriction on weight loss and insulin sensitivity in obese men and women. *Journal of Nutrition*. 2005; 135: 2387-91.
1053 Raben A, Astrup A, Vasilaras TH, Prentice AM, Zunft HJ, Formiguera X*, et al.* The CARMEN trial: increased intake of carbohydrates--simple or complex--and unchanged blood lipids in overweight subjects. *Ugeskrift for Laeger*. 2002; 164: 627-31.
1054 Rabinovitz HR, Boaz M, Ganz T, Jakubowicz D, Matas Z, Madar Z*, et al.* Big breakfast rich in protein and fat improves glycemic control in type 2 diabetics. *Obesity*. 2014; 22: E46-54.

1055 Racette SB, Schoeller DA, Kushner RF, Neil KM. Exercise enhances dietary compliance during moderate energy restriction in obese women. *American Journal of Clinical Nutrition*. 1995; 62: 345-49.
1056 Racette SB, Schoeller DA, Kushner RF, Neil KM, Herling-Iaffaldano K. Effects of aerobic exercise and dietary carbohydrate on energy expenditure and body composition during weight reduction in obese women. *American Journal of Clinical Nutrition*. 1995; 61: 486-94.
1057 Rader S, Dorner TE, Schoberberger R, Wolf H. Effects of a web-based follow-up intervention on self-efficacy in obesity treatment for women. *Wiener Klinische Wochenschrift*. 2017; 129: 472-81.
1058 Radin RM, Epel ES, Daubenmier J, Moran P, Schleicher S, Kristeller J*, et al.* Do stress eating or compulsive eating influence metabolic health in a mindfulness-based weight loss intervention? *Health Psychology*. 2020; 39: 147-58.
1059 Rafiq MT, Hamid MSA, Hafiz E. Effect of Progressive Resistance Strength Training on Body Mass Index, Quality of Life and Functional Capacity in Knee Osteoarthritis: A Randomized Controlled Trial. *Journal of Multidisciplinary Healthcare*. 2021; 14: 2161-68.
1060 Rakvaag E, Fuglsang-Nielsen R, Bach Knudsen KE, Landberg R, Johannesson Hjelholt A, Sondergaard E*, et al.* Whey Protein Combined with Low Dietary Fiber Improves Lipid Profile in Subjects with Abdominal Obesity: A Randomized, Controlled Trial. *Nutrients*. 2019; 11: 04.
1061 Rallidis LS, Lekakis J, Kolomvotsou A, Zampelas A, Vamvakou G, Efstathiou S*, et al.* Close adherence to a Mediterranean diet improves endothelial function in subjects with abdominal obesity. *American Journal of Clinical Nutrition*. 2009; 90: 263-68.
1062 Raman J, Hay P, Tchanturia K, Smith E. A randomised controlled trial of manualized cognitive remediation therapy in adult obesity. *Appetite*. 2018; 123: 269-79.
1063 Ramirez EM, Rosen JC. A comparison of weight control and weight control plus body image therapy for obese men and women. *Journal of consulting and clinical psychology*. 2001; 69: 440-46.
1064 Ramos RM, Coelho-Junior HJ, Asano RY, Prado RCR, Silveira R, Urtado CB*, et al.* Impact of Moderate Aerobic Training on Physical Capacities of Hypertensive Obese Elderly. *Gerontology & Geriatric Medicine*. 2019; 5: 2333721419859691.
1065 Ramos RM, Coelho-Junior HJ, do Prado RCR, da Silva RS, Asano RY, Prestes J*, et al.* Moderate Aerobic Training Decreases Blood Pressure but No Other Cardiovascular Risk Factors in Hypertensive Overweight/Obese Elderly Patients. *Gerontology & Geriatric Medicine*. 2018; 4: 2333721418808645.
1066 Ramos-Lopez O, Riezu-Boj JI, Milagro FI, Goni L, Cuervo M, Martinez JA. Differential lipid metabolism outcomes associated with ADRB2 gene polymorphisms in response to two dietary interventions in overweight/obese subjects. *Nutrition, metabolism, and cardiovascular diseases : NMCD*. 2018; 28: 165-72.
1067 Rana BK, Flatt SW, Pakiz B, Quintana EL, Natarajan L, Rock CL. The IL-6 Gene Promoter SNP and Plasma IL-6 in Response to Diet Intervention. *Nutrients*. 2017; 9.
1068 Randolph JM, Edirisinghe I, Masoni AM, Kappagoda T, Burton-Freeman B. Potatoes, glycemic index, and weight loss in free-living individuals: practical implications. *Journal of the American College of Nutrition*. 2014; 33: 375-84.
1069 Razquin C, Martinez JA, Martinez-Gonzalez MA, Mitjavila MT, Estruch R, Marti A. A 3 years follow-up of a Mediterranean diet rich in virgin olive oil is associated with high plasma antioxidant capacity and reduced body weight gain. *European Journal of Clinical Nutrition*. 2009; 63: 1387-93.

1070 Razquin C, Sanchez-Tainta A, Salas-Salvado J, Buil-Cosiales P, Corella D, Fito M*, et al.* Dietary energy density and body weight changes after 3 years in the PREDIMED study. *International Journal of Food Sciences & Nutrition*. 2017; 68: 865-72.
1071 Redman LM, Heilbronn LK, Martin CK, Alfonso A, Smith SR, Ravussin E. Effect of calorie restriction with or without exercise on body composition and fat distribution. *Journal of clinical endocrinology and metabolism*. 2007; 92: 865-72.
1072 Reichard A, Saunders MD, Saunders RR, Donnelly JE, Lauer E, Sullivan DK*, et al.* A comparison of two weight management programs for adults with mobility impairments. *Disability and health journal*. 2015; 8: 61-69.
1073 Reid CM, Dart AM, Dewar EM, Jennings GL. Interactions between the effects of exercise and weight loss on risk factors, cardiovascular haemodynamics and left ventricular structure in overweight subjects. *Journal of Hypertension*. 1994; 12: 291-301.
1074 Rejeski JJ, Fanning J, Nicklas BJ, Rejeski WJ. Six-month changes in ghrelin and glucagon-like peptide-1 with weight loss are unrelated to long-term weight regain in obese older adults. *International Journal of Obesity*. 2021; 45: 888-94.
1075 Rejeski WJ, Ambrosius WT, Burdette JH, Walkup MP, Marsh AP. Community Weight Loss to Combat Obesity and Disability in At-Risk Older Adults. *Journals of Gerontology Series A-Biological Sciences & Medical Sciences*. 2017; 72: 1547-53.
1076 Rejeski WJ, Brubaker PH, Goff DC, Jr., Bearon LB, McClelland JW, Perri MG*, et al.* Translating weight loss and physical activity programs into the community to preserve mobility in older, obese adults in poor cardiovascular health. *Archives of Internal Medicine*. 2011; 171: 880-86.
1077 Rejeski WJ, Focht BC, Messier SP, Morgan T, Pahor M, Penninx B. Obese, older adults with knee osteoarthritis: weight loss, exercise, and quality of life. *Health Psychology*. 2002; 21: 419-26.
1078 Rejeski WJ, Marsh AP, Fanning J, Ambrosius WT, Walkup MP, Nicklas BJ. Dietary Weight Loss, Exercise, and Inflammation in Older Adults with Overweight or Obesity and Cardiometabolic Disease. *Obesity*. 2019; 27: 1805-11.
1079 Rejeski WJ, Mihalko SL, Ambrosius WT, Bearon LB, McClelland JW. Weight loss and self-regulatory eating efficacy in older adults: the cooperative lifestyle intervention program. *Journals of Gerontology Series B-Psychological Sciences & Social Sciences*. 2011; 66: 279-86.
1080 Renjilian DA, Perri MG, Nezu AM, McKelvey WF, Shermer RL, Anton SD. Individual versus group therapy for obesity: effects of matching participants to their treatment preferences. *Journal of consulting and clinical psychology*. 2001; 69: 717-21.
1081 Reseland JE, Anderssen SA, Solvoll K, Hjermann I, Urdal P, Holme I*, et al.* Effect of long-term changes in diet and exercise on plasma leptin concentrations. *American Journal of Clinical Nutrition*. 2001; 73: 240-45.
1082 Rice B, Janssen I, Hudson R, Ross R. Effects of aerobic or resistance exercise and/or diet on glucose tolerance and plasma insulin levels in obese men. *Diabetes Care*. 1999; 22: 684-91.
1083 Rickel KA, Milsom VA, Ross KM, Hoover VJ, Peterson ND, Perri MG. Differential response of African American and Caucasian women to extended-care programs for obesity management. *Ethnicity & Disease*. 2011; 21: 170-75.
1084 Rieger E, Treasure J, Murray K, Caterson I. The use of support people to improve the weight-related and psychological outcomes of adults with obesity: a randomised controlled trial. *Behaviour research and therapy*. 2017; 94: 48-59.
1085 Rimmer JH, Rauworth A, Wang E, Heckerling PS, Gerber BS. A randomized controlled trial to increase physical activity and reduce obesity in a predominantly African American

group of women with mobility disabilities and severe obesity. *Preventive Medicine*. 2009; 48: 473-79.
1086 Risica PM, Gans KM, Kumanyika S, Kirtania U, Lasater TM. SisterTalk: final results of a culturally tailored cable television delivered weight control program for Black women. *International Journal of Behavioral Nutrition & Physical Activity*. 2013; 10: 141.
1087 Rissanen A, Pietinen P, Siljamäki-Ojansuu U, Piirainen H, Reissel P. Treatment of hypertension in obese patients: efficacy and feasibility of weight and salt reduction programs. *Acta Medica Scandinavica*. 1985; 218: 149-56.
1088 Riva G, Bacchetta M, Cesa G, Conti S, Molinari E. Six-Month Follow-Up of In-Patient Experiential Cognitive Therapy for Binge Eating Disorders. *CyberPsychology & Behavior*. 2003; 6: 251-58.
1089 Rock CL, Flatt SW, Barkai HS, Pakiz B, Heath DD. Walnut consumption in a weight reduction intervention: effects on body weight, biological measures, blood pressure and satiety. *Nutrition Journal*. 2017; 16: 76.
1090 Rock CL, Flatt SW, Pakiz B, Barkai HS, Heath DD, Krumhar KC. Randomized clinical trial of portion-controlled prepackaged foods to promote weight loss. *Obesity*. 2016; 24: 1230-37.
1091 Rock CL, Flatt SW, Pakiz B, Quintana EL, Heath DD, Rana BK*, et al.* Effects of diet composition on weight loss, metabolic factors and biomarkers in a 1-year weight loss intervention in obese women examined by baseline insulin resistance status. *Metabolism: clinical and experimental*. 2016; 65: 1605-13.
1092 Rock CL, Flatt SW, Pakiz B, Taylor KS, Leone AF, Brelje K*, et al.* Weight loss, glycemic control, and cardiovascular disease risk factors in response to differential diet composition in a weight loss program in type 2 diabetes: a randomized controlled trial. *Diabetes Care*. 2014; 37: 1573-80.
1093 Rodrigues APS, Rosa LPS, Silveira EA. PPARG2 Pro12Ala polymorphism influences body composition changes in severely obese patients consuming extra virgin olive oil: A randomized clinical trial. *Nutrition and Metabolism*. 2018; 15: 1-13.
1094 Rodriguez-Cristobal JJ, Alonso-Villaverde C, Panisello JM, Trave-Mercade P, Rodriguez-Cortes F, Marsal JR*, et al.* Effectiveness of a motivational intervention on overweight/obese patients in the primary healthcare: a cluster randomized trial. *BMC Family Practice*. 2017; 18: 74.
1095 Rohling M, Kempf K, Banzer W, Braumann KM, Fuhrer-sakel D, Halle M*, et al.* A High-Protein and Low-Glycemic Formula Diet Improves Blood Pressure and Other Hemodynamic Parameters in High-Risk Individuals. *Nutrients*. 2022; 14.
1096 Rolland C, Hession M, Murray S, Wise A, Broom I. Randomized clinical trial of standard dietary treatment versus a low-carbohydrate/high-protein diet or the LighterLife Programme in the management of obesity. *Journal Of Diabetes*. 2009; 1: 207-17.
1097 Rolls BJ, Roe LS, Beach AM, Kris-Etherton PM. Provision of foods differing in energy density affects long-term weight loss. *Obesity Research*. 2005; 13: 1052-60.
1098 Rolls BJ, Roe LS, James BL, Sanchez CE. Does the incorporation of portion-control strategies in a behavioral program improve weight loss in a 1-year randomized controlled trial? *International Journal of Obesity*. 2017; 41: 434-42.
1099 Romero Z, Kimbrough S. The impact of moderate physical activity on weight and blood pressure in an elderly Mexican-American female population. *TAHPERD journal*. 2019; 87: 8-12.
1100 Rosas LG, Azar KMJ, Lv N, Xiao L, Goldhaber-Fiebert JD, Snowden MB*, et al.* Effect of an intervention for obesity and depression on patient-centered outcomes: An RCT. *American Journal of Preventive Medicine*. 2020; 58: 496-505.

1101 Rosenthal B, Allen GJ, Winter C. Husband involvement in the behavioral treatment of overweight women: initial effects and long-term follow-up. *International Journal of Obesity*. 1980; 4: 165-73.
1102 Ross KM, Wing RR. Impact of newer self-monitoring technology and brief phone-based intervention on weight loss: A randomized pilot study. *Obesity*. 2016; 24: 1653-59.
1103 Ross R, Hudson R, Stotz PJ, Lam M. Effects of exercise amount and intensity on abdominal obesity and glucose tolerance in obese adults: a randomized trial. *Annals of Internal Medicine*. 2015; 162: 325-34.
1104 Ross R, Rissanen J. Mobilization of visceral and subcutaneous adipose tissue in response to energy restriction and exercise. *American Journal of Clinical Nutrition*. 1994; 60: 695-703.
1105 Rossello X, Ramallal R, Romaguera D, Alonso-Gomez AM, Alonso A, Tojal-Sierra L*, et al.* Effect of an intensive lifestyle intervention on the structural and functional substrate for atrial fibrillation in people with metabolic syndrome. *European Journal of Preventive Cardiology*. 2024; 31: 629-39.
1106 Rossi A, Garber CE, Ortiz M, Shankar V, Goldberg GL, Nevadunsky NS. Feasibility of a physical activity intervention for obese, socioculturally diverse endometrial cancer survivors. *Gynecologic Oncology*. 2016; 142: 304-10.
1107 Rossi AP, Muollo V, Fantin F, Masciocchi E, Urbani S, Taylor M*, et al.* Effects of diet combined with Nordic walking or walking programme on weight loss and arterial stiffness in postmenopausal overweight and obese women: The Walking and Aging Verona pilot study. *European Journal of Preventive Cardiology*. 2020; 27: 2208-11.
1108 Rothert K, Strecher VJ, Doyle LA, Caplan WM, Joyce JS, Jimison HB*, et al.* Web-based Weight Management Programs in an Integrated Health Care Setting: A Randomized, Controlled Trial. *Obesity*. 2006; 14: 266-72.
1109 Rozati M, Barnett J, Wu D, Handelman G, Saltzman E, Wilson T*, et al.* Cardio-metabolic and immunological impacts of extra virgin olive oil consumption in overweight and obese older adults: a randomized controlled trial. *Nutrition & Metabolism*. 2015; 12: 28.
1110 Rubin RR, Peyrot M, Wang NY, Coughlin JW, Jerome GJ, Fitzpatrick SL*, et al.* Patient-reported outcomes in the practice-based opportunities for weight reduction (POWER) trial. *Quality of Life Research: An International Journal of Quality of Life Aspects of Treatment, Care & Rehabilitation*. 2013; 22: 2389-98.
1111 Rubin RR, Wadden TA, Bahnson JL, Blackburn GL, Brancati FL, Bray GA*, et al.* Impact of intensive lifestyle intervention on depression and health-related quality of life in type 2 diabetes: the Look AHEAD Trial. *Diabetes Care*. 2014; 37: 1544-53.
1112 Rumbo-Rodriguez L, Zaragoza-Marti A, Sanchez-SanSegundo M, Ferrer-Cascales R, Laguna-Perez A, Hurtado-Sanchez JA. Effectiveness of a Two-Year Multicomponent Intervention for the Treatment of Overweight and Obesity in Older People. *Nutrients*. 2022; 14: 11.
1113 Runhaar J, Beavers DP, Miller GD, Nicklas BJ, Loeser RF, Bierma-Zeinstra S*, et al.* Inflammatory cytokines mediate the effects of diet and exercise on pain and function in knee osteoarthritis independent of BMI. *Osteoarthritis and cartilage*. 2019; 27: 1118-23.
1114 Rushing K, Stenhouse A, Evans M, Huffman L, Bowie V. A low-carbohydrate diet versus a low-calorie diet: Poor retention. *Topics in clinical nutrition*. 2006; 21: 251-59.
1115 Rustaden AM, Haakstad LAH, Paulsen G, Bø K. Effects of BodyPump and resistance training with and without a personal trainer on muscle strength and body composition in overweight and obese women-A randomised controlled trial. *Obesity Research & Clinical Practice*. 2017; 11: 728-39.

1116 Ryan MC, Abbasi F, Lamendola C, Carter S, McLaughlin TL. Serum alanine aminotransferase levels decrease further with carbohydrate than fat restriction in insulin-resistant adults. *Diabetes Care*. 2007; 30: 1075-80.
1117 Ryttig KR, Rossner S. Weight maintenance after a very low calorie diet (VLCD) weight reduction period and the effects of VLCD supplementation. A prospective, randomized, comparative, controlled long-term trial. *Journal of Internal Medicine*. 1995; 238: 299-306.
1118 Saad MF, Cheah WL, Hazmi H. The Effects of a 7000-Step Goal and Weekly Group Walking Program for Overweight and Obese Elderly People in Sarawak, Malaysia: A Quasi-experimental Study. *Journal of Preventive Medicine & Public Health*. 2021; 54: 199-207.
1119 Sacks FM, Bray GA, Carey VJ, Smith SR, Ryan DH, Anton SD*, et al.* Comparison of weight-loss diets with different compositions of fat, protein, and carbohydrates. *New England Journal of Medicine*. 2009; 360: 859-73.
1120 Saffari M, Pakpour AH, Mohammadi-Zeidi I, Samadi M, Chen H. Long-term effect of motivational interviewing on dietary intake and weight loss in Iranian obese/overweight women. *Health Promotion Perspectives*. 2014; 4: 206-13.
1121 Sahlman J, Seppa J, Herder C, Peltonen M, Peuhkurinen K, Gylling H*, et al.* Effect of weight loss on inflammation in patients with mild obstructive sleep apnea. *Nutrition Metabolism & Cardiovascular Diseases*. 2012; 22: 583-90.
1122 Said M, Lamya N, Olfa N, Hamda M. Effects of high-impact aerobics vs. low-impact aerobics and strength training in overweight and obese women. *Journal of sports medicine and physical fitness*. 2017; 57: 278-88.
1123 Saidi O, Colin E, Rance M, Doré E, Pereira B, Duché P. Effect of morning versus evening exercise training on sleep, physical activity, fitness, fatigue and quality of life in overweight and obese adults. *Chronobiology International*. 2021; 38: 1537-48.
1124 Salas-Salvado J, Fernandez-Ballart J, Ros E, Martinez-Gonzalez MA, Fito M, Estruch R*, et al.* Effect of a Mediterranean diet supplemented with nuts on metabolic syndrome status: one-year results of the PREDIMED randomized trial. *Archives of Internal Medicine*. 2008; 168: 2449-58.
1125 Saldivar P, Mira V, Duran P, Moldovan C, Ang G, Parikh N*, et al.* Implementing texting programs in the P.O.W.E.R. (preventing obesity with eating right) medical group visit for weight loss. *Obesity Science and Practice*. 2021; 7(5): 583-90.
1126 Salopuro T, Pulkkinen L, Lindström J, Eriksson JG, Valle TT, Hämäläinen H*, et al.* Genetic variation in leptin receptor gene is associated with type 2 diabetes and body weight: the Finnish Diabetes Prevention Study. *International journal of obesity (2005)*. 2005; 29: 1245-51.
1127 Samaha FF, Iqbal N, Seshadri P, Chicano KL, Daily DA, McGrory J*, et al.* A low-carbohydrate as compared with a low-fat diet in severe obesity. *New England Journal of Medicine*. 2003; 348: 2074-81.
1128 Sanal E, Ardic F, Kirac S. Effects of aerobic or combined aerobic resistance exercise on body composition in overweight and obese adults: gender differences. A randomized intervention study. *European journal of physical and rehabilitation medicine*. 2013; 49: 1-11.
1129 Santanasto AJ, Glynn NW, Newman MA, Taylor CA, Brooks MM, Goodpaster BH*, et al.* Impact of weight loss on physical function with changes in strength, muscle mass, and muscle fat infiltration in overweight to moderately obese older adults: a randomized clinical trial. *Journal of Obesity*. 2011.
1130 Santanasto AJ, Newman AB, Strotmeyer ES, Boudreau RM, Goodpaster BH, Glynn NW. Effects of Changes in Regional Body Composition on Physical Function in Older Adults: A Pilot Randomized Controlled Trial. *Journal of Nutrition, Health & Aging*. 2015; 19: 913-21.

1131 Sardeli AV, Castro A, Gadelha VB, Santos WMD, Lord JM, Cavaglieri CR*, et al.* Metabolomic Response throughout 16 Weeks of Combined Aerobic and Resistance Exercise Training in Older Women with Metabolic Syndrome. *Metabolites*. 2022; 12: 30.
1132 Saris WH, Astrup A, Prentice AM, Zunft HJ, Formiguera X, Verboeket-van de Venne WP*, et al.* Randomized controlled trial of changes in dietary carbohydrate/fat ratio and simple vs complex carbohydrates on body weight and blood lipids: the CARMEN study. The Carbohydrate Ratio Management in European National diets. *International journal of obesity and related metabolic disorders*. 2000; 24: 1310-18.
1133 Sarmento Tenório LH, Santos AC, Câmara Neto JB, Amaral FJ, Moraes Passos VM, Jaguaribe Lima AM*, et al.* The influence of inspiratory muscle training on diaphragmatic mobility, pulmonary function and maximum respiratory pressures in morbidly obese individuals: A pilot study. *Disability and Rehabilitation: An International, Multidisciplinary Journal*. 2013; 35: 1915-20.
1134 Sarsan A, Ardiç F, Ozgen M, Topuz O, Sermez Y. The effects of aerobic and resistance exercises in obese women. *Clinical Rehabilitation*. 2006; 20: 773-82.
1135 Sarzynski MA, Ruiz-Ramie JJ, Barber JL, Slentz CA, Apolzan JW, McGarrah RW*, et al.* Effects of Increasing Exercise Intensity and Dose on Multiple Measures of HDL (High-Density Lipoprotein) Function. *Arteriosclerosis, Thrombosis & Vascular Biology*. 2018; 38: 943-52.
1136 Saslow LR, Kim S, Daubenmier JJ, Moskowitz JT, Phinney SD, Goldman V*, et al.* A randomized pilot trial of a moderate carbohydrate diet compared to a very low carbohydrate diet in overweight or obese individuals with type 2 diabetes mellitus or prediabetes. *PloS one*. 2014; 9: e91027.
1137 Saslow LR, Mason AE, Kim S, Goldman V, Ploutz-Snyder R, Bayandorian H*, et al.* An Online Intervention Comparing a Very Low-Carbohydrate Ketogenic Diet and Lifestyle Recommendations Versus a Plate Method Diet in Overweight Individuals With Type 2 Diabetes: a Randomized Controlled Trial. *Journal of Medical Internet Research*. 2017; 19: e36.
1138 Savage PD, Lee M, Harvey-Berino J, Brochu M, Ades PA. Weight reduction in the cardiac rehabilitation setting. *Journal of Cardiopulmonary Rehabilitation*. 2002; 22: 154-60.
1139 Saxton JM, Scott EJ, Daley AJ, Woodroofe M, Mutrie N, Crank H*, et al.* Effects of an exercise and hypocaloric healthy eating intervention on indices of psychological health status, hypothalamic-pituitary-adrenal axis regulation and immune function after early-stage breast cancer: a randomised controlled trial. *Breast Cancer Research*. 2014; 16: R39.
1140 Sbierski-Kind J, Mai K, Kath J, Jurisch A, Streitz M, Kuchenbecker L*, et al.* Association between Subcutaneous Adipose Tissue Inflammation, Insulin Resistance, and Calorie Restriction in Obese Females. *Journal of Immunology*. 2020; 205: 45-55.
1141 Sbrocco T, Nedegaard RC, Stone JM, Lewis EL. Behavioral choice treatment promotes continuing weight loss: Preliminary results of a cognitive–behavioral decision-based treatment for obesity. *Journal of consulting and clinical psychology*. 1999; 67: 260-66.
1142 Schenk S, Harber MP, Shrivastava CR, Burant CF, Horowitz JF. Improved insulin sensitivity after weight loss and exercise training is mediated by a reduction in plasma fatty acid mobilization, not enhanced oxidative capacity. *The Journal of Physiology*. 2009; 587: 4949-61.
1143 Schjerve IE, Tyldum GA, Tjønna AE, Stølen T, Loennechen JP, Hansen HE*, et al.* Both aerobic endurance and strength training programmes improve cardiovascular health in obese adults. *Clinical science (London, England : 1979)*. 2008; 115: 283-93.

1144 Schlundt DG, Hill JO, Pope-Cordle J, Arnold D, Virts KL, Katahn M. Randomized evaluation of a low fat ad libitum carbohydrate diet for weight reduction. *International journal of obesity and related metabolic disorders*. 1993; 17: 623-29.
1145 Schmitz KH, Troxel AB, Dean LT, DeMichele A, Brown JC, Sturgeon K*, et al.* Effect of Home-Based Exercise and Weight Loss Programs on Breast Cancer–Related Lymphedema Outcomes Among Overweight Breast Cancer Survivors: The WISER Survivor Randomized Clinical Trial. *JAMA Oncology*. 2019; 5: 1605-13.
1146 Schooneman MG, Napolitano A, Houten SM, Ambler GK, Murgatroyd PR, Miller SR*, et al.* Assessment of plasma acylcarnitines before and after weight loss in obese subjects. *Archives of biochemistry and biophysics*. 2016; 606: 73-80.
1147 Schroder H, Cardenas-Fuentes G, Martinez-Gonzalez MA, Corella D, Vioque J, Romaguera D*, et al.* Effectiveness of the physical activity intervention program in the PREDIMED-Plus study: a randomized controlled trial. *International Journal of Behavioral Nutrition & Physical Activity*. 2018; 15: 110.
1148 Schulte EM, Tuerk PW, Wadden TA, Garvey WT, Weiss D, Hermayer KL*, et al.* Changes in weight control behaviors and hedonic hunger in a commercial weight management program adapted for individuals with type 2 diabetes. *International Journal of Obesity*. 2020; 44: 990-98.
1149 Schumacher LM, Godfrey KM, Forman EM, Butryn ML. Change in domain-specific but not general psychological flexibility relates to greater weight loss in acceptance-based behavioral treatment for obesity. *Journal of Contextual Behavioral Science*. 2019; 12: 59-65.
1150 Schwab U, Seppanen-Laakso T, Yetukuri L, Agren J, Kolehmainen M, Laaksonen DE*, et al.* Triacylglycerol fatty acid composition in diet-induced weight loss in subjects with abnormal glucose metabolism - The GENOBIN study. *PloS one*. 2008; 3.
1151 Sedjo RL, Flatt SW, Byers T, Colditz GA, Demark-Wahnefried W, Ganz PA*, et al.* Impact of a behavioral weight loss intervention on comorbidities in overweight and obese breast cancer survivors. *Supportive Care in Cancer*. 2016; 24: 3285-93.
1152 Seif-Barghi T, Akbari-Fakhrabadi M, Teimori MP, Tashk A, Alizadeh Z, Memari AH. Cognitive Behavior Therapy's Effect in a Weight Loss Program Among Obese Iranian Women. *Nutrition Today*. 2018; 53: 174-78.
1153 Sen A, Jen KL, Djuric Z. Baseline leptin levels predict change in leptin levels during weight loss in obese breast cancer survivors. *Breast Journal*. 2007; 13: 180-86.
1154 Senna MK, Sallam RA, Ashour HS, Elarman M. Effect of weight reduction on the quality of life in obese patients with fibromyalgia syndrome: a randomized controlled trial. *Clinical Rheumatology*. 2012; 31: 1591-97.
1155 Serra MC, Ryan AS. Bone Mineral Density Changes during Weight Regain following Weight Loss with and without Exercise. *Nutrients*. 2021; 13: 19.
1156 Seshadri P, Iqbal N, Stern L, Williams M, Chicano KL, Daily DA*, et al.* A randomized study comparing the effects of a low-carbohydrate diet and a conventional diet on lipoprotein subfractions and C-reactive protein levels in patients with severe obesity. *American Journal of Medicine*. 2004; 117: 398-405.
1157 Seshadri P, Samaha FF, Stern L, Chicano KL, Daily DA, Iqbal N. Free fatty acids, insulin resistance, and corrected qt intervals in morbid obesity: effect of weight loss during 6 months with differing dietary interventions. *Endocrine Practice*. 2005; 11: 234-39.
1158 Shabani A, Shabani R, Dalili S, Rad AH. The effect of concurrent endurance and resistance training on cardio-respiratory capacity and cardiovascular risk markers among sedentary overweight or obese post-menopausal women. *Journal of nursing & midwifery sciences*. 2018; 5: 123-29.

1159 Shamsoddini A, Sobhani V, Chehreh MEG, Alavian SM, Zaree A. Effect of Aerobic and Resistance Exercise Training on Liver Enzymes and Hepatic Fat in Iranian Men With Nonalcoholic Fatty Liver Disease. *Hepatitis monthly*. 2015; 15: 7.
1160 Shapiro JR, Koro T, Doran N, Thompson S, Sallis JF, Calfas K*, et al.* Text4Diet: a randomized controlled study using text messaging for weight loss behaviors. *Preventive Medicine*. 2012; 55: 412-17.
1161 Shaver LN, Beavers DP, Kiel J, Kritchevsky SB, Beavers KM. Effect of Intentional Weight Loss on Mortality Biomarkers in Older Adults With Obesity. *Journals of Gerontology Series A-Biological Sciences & Medical Sciences*. 2019; 74: 1303-09.
1162 Shaw C, Mortimer P, Judd PA. A randomized controlled trial of weight reduction as a treatment for breast cancer-related lymphedema. *Cancer*. 2007; 110: 1868-74.
1163 Shaw C, Mortimer P, Judd PA. Randomized controlled trial comparing a low-fat diet with a weight-reduction diet in breast cancer-related lymphedema. *Cancer*. 2007; 109: 1949-56.
1164 Shaw RJ, Bosworth HB, Silva SS, Lipkus IM, Davis LL, Sha RS*, et al.* Mobile health messages help sustain recent weight loss. *American Journal of Medicine*. 2013; 126: 1002-09.
1165 Shea MK, Houston DK, Nicklas BJ, Messier SP, Davis CC, Miller ME*, et al.* The effect of randomization to weight loss on total mortality in older overweight and obese adults: the ADAPT Study. *Journals of Gerontology Series A-Biological Sciences & Medical Sciences*. 2010; 65: 519-25.
1166 Shea MK, Nicklas BJ, Houston DK, Miller ME, Davis CC, Kitzman DW*, et al.* The effect of intentional weight loss on all-cause mortality in older adults: results of a randomized controlled weight-loss trial. *American Journal of Clinical Nutrition*. 2011; 94: 839-46.
1167 Shechter A, Foster GD, Lang W, Reboussin DM, St-Onge MP, Zammit G*, et al.* Effects of a lifestyle intervention on REM sleep-related OSA severity in obese individuals with type 2 diabetes. *Journal of Sleep Research*. 2017; 26: 747-55.
1168 Shechter A, St-Onge MP, Kuna ST, Zammit G, RoyChoudhury A, Newman AB*, et al.* Sleep architecture following a weight loss intervention in overweight and obese patients with obstructive sleep apnea and type 2 diabetes: relationship to apnea-hypopnea index. *Journal of Clinical Sleep Medicine*. 2014; 10: 1205-11.
1169 Shenoy SF, Poston WS, Reeves RS, Kazaks AG, Holt RR, Keen CL*, et al.* Weight loss in individuals with metabolic syndrome given DASH diet counseling when provided a low sodium vegetable juice: a randomized controlled trial. *Nutrition Journal*. 2010; 9: 8.
1170 Sheppard L, Kristal AR, Kushi LH. Weight loss in women participating in a randomized trial of low-fat diets. *American Journal of Clinical Nutrition*. 1991; 54: 821-28.
1171 Sheppard VB, Hicks J, Makambi K, Hurtado-de-Mendoza A, Demark-Wahnefried W, Adams-Campbell L. The feasibility and acceptability of a diet and exercise trial in overweight and obese black breast cancer survivors: the Stepping STONE study. *Contemporary Clinical Trials*. 2016; 46: 106-13.
1172 Sherwood NE, Crain AL, Martinson BC, Anderson CP, Hayes MG, Anderson JD*, et al.* Enhancing long-term weight loss maintenance: 2 year results from the Keep It Off randomized controlled trial. *Preventive Medicine*. 2013; 56: 171-77.
1173 Sherwood NE, Jeffery RW, Pronk NP, Boucher JL, Hanson A, Boyle R*, et al.* Mail and phone interventions for weight loss in a managed-care setting: Weigh-to-be 2-year outcomes. *International Journal of Obesity*. 2006; 30: 1565-73.
1174 Sherwood NE, Jeffery RW, Welsh EM, Van Wormer J, Hotop AM. The Drop It At Last study: Six-month results of a phone-based weight loss trial. *American Journal of Health Promotion*. 2010; 24: 378-83.

1175 Shi X, Yin H, Li J, Huang C, Chen Y, Chen Z*, et al.* Circulating branch chain amino acids and improvement in liver fat content in response to exercise interventions in NAFLD. *Scientific reports*. 2021; 11: 13415-.
1176 Shige H, Nestel P, Sviridov D, Noakes M, Clifton P. Effect of weight reduction on the distribution of apolipoprotein A-I in high-density lipoprotein subfractions in obese non-insulin-dependent diabetic subjects. *Metabolism: clinical and experimental*. 2000; 49: 1453-59.
1177 Shirai K, Saiki A, Oikawa S, Teramoto T, Yamada N, Ishibashi S*, et al.* The effects of partial use of formula diet on weight reduction and metabolic variables in obese type 2 diabetic patients--multicenter trial. *Obesity Research & Clinical Practice*. 2013; 7: e43-54.
1178 Sijie T, Ping D, Wanting Z, Jiaqi P, Jianxiong W. Exercise Training at Maximal Fat Oxidation Intensity for Older Women with Type 2 Diabetes. *International Journal of Sports Medicine*. 2018; 39: 374-81.
1179 Silva Pestana MD, Martins Netto E, Silva Pestana MC, Silva Pestana V, Schinoni MI. Pilates versus resistance exercise on the serum levels of hs-CRP, in the abdominal circumference and body mass index (BMI) in elderly individuals. *Motricidade*. 2016; 12: 128-40.
1180 Simpson SA, Matthews L, Pugmire J, McConnachie A, McIntosh E, Coulman E*, et al.* An app-, web- and social support-based weight loss intervention for adults with obesity: the 'HelpMeDoIt!' feasibility randomised controlled trial. *Pilot & Feasibility Studies*. 2020; 6: 133.
1181 Singh RB, Niaz MA, Bishnoi I, Singh U, Begum R, Rastogi SS. Effect of low energy diet and weight loss on major risk factors, central obesity and associated disturbances in patients with essential hypertension. *Journal of Human Hypertension*. 1995; 9: 355-62.
1182 Skender ML, Goodrick GK, Del Junco DJ, Reeves RS, Darnell L, Gotto AM*, et al.* Comparison of 2-year weight loss trends in behavioral treatments of obesity: diet, exercise, and combination interventions. *Journal of the American Dietetic Association*. 1996; 96: 342-46.
1183 Skov AR, Toubro S, Bulow J, Krabbe K, Parving HH, Astrup A. Changes in renal function during weight loss induced by high vs low-protein low-fat diets in overweight subjects. *International Journal of Obesity*. 1999; 23: 1170-77.
1184 Skrypnik D, Bogdański P, Mądry E, Karolkiewicz J, Ratajczak M, Kryściak J*, et al.* Effects of Endurance and Endurance Strength Training on Body Composition and Physical Capacity in Women with Abdominal Obesity. *Obesity Facts*. 2015; 8: 175-87.
1185 Skrypnik D, Ratajczak M, Karolkiewicz J, Madry E, Pupek-Musialik D, Hansdorfer-Korzon R*, et al.* Effects of endurance and endurance-strength exercise on biochemical parameters of liver function in women with abdominal obesity. *Biomedicine and Pharmacotherapy*. 2016; 80: 1-7.
1186 Slentz CA, Aiken LB, Houmard JA, Bales CW, Johnson JL, Tanner CJ*, et al.* Inactivity, exercise, and visceral fat. STRRIDE: a randomized, controlled study of exercise intensity and amount. *Journal of applied physiology (Bethesda, Md : 1985)*. 2005; 99: 1613-18.
1187 Slentz CA, Houmard JA, Johnson JL, Bateman LA, Tanner CJ, McCartney JS*, et al.* Inactivity, exercise training and detraining, and plasma lipoproteins. STRRIDE: a randomized, controlled study of exercise intensity and amount. *Journal of Applied Physiology*. 2007; 103: 432-42.
1188 Slentz CA, Tanner CJ, Bateman LA, Durheim MT, Huffman KM, Houmard JA*, et al.* Effects of exercise training intensity on pancreatic beta-cell function. *Diabetes Care*. 2009; 32: 1807-11.

1189 Smith BK, Van Walleghen EL, Cook-Wiens G, Martin RN, Curry CR, Sullivan DK*, et al.* Comparison of two self-directed weight loss interventions: Limited weekly support vs. no outside support. *Obesity Research and Clinical Practice*. 2009; 3: 149-57.
1190 Smith DE, Heckemeyer CM, Kratt PP, Mason DA, Smith DE, Heckemeyer CM*, et al.* Motivational interviewing to improve adherence to a behavioral weight-control program for older obese women with NIDDM. A pilot study. *Diabetes Care*. 1997; 20: 52-54.
1191 Smith PJ, Blumenthal JA, Babyak MA, Craighead L, Welsh-Bohmer KA, Browndyke JN*, et al.* Effects of the Dietary Approaches to Stop Hypertension Diet, Exercise, and Caloric Restriction on Neurocognition in Overweight Adults With High Blood Pressure. *Hypertension*. 2010; 55: 1331-U85.
1192 Smith WA, Zucker-Levin A, Mihalko WM, Williams M, Loftin M, Gurney JG. A Randomized Study of Exercise and Fitness Trackers in Obese Patients After Total Knee Arthroplasty. *Orthopedic clinics of North America*. 2019; 50: 35-45.
1193 Snel M, Sleddering MA, Vd Peijl ID, Romijn JA, Pijl H, Meinders AE*, et al.* Quality of life in type 2 diabetes mellitus after a very low calorie diet and exercise. *European journal of internal medicine*. 2012; 23: 143-49.
1194 Snow R, Ruane J, LaLonde M, Shaffer L, Kim B, Graffagnino C*, et al.* Randomized trial assessing the impact of a musculoskeletal intervention for pain before participating in a weight management program. *Journal of cardiopulmonary rehabilitation and prevention*. 2010; 30: 173-80.
1195 Soenen S, Martens EA, Hochstenbach-Waelen A, Lemmens SG, Westerterp-Plantenga MS. Normal protein intake is required for body weight loss and weight maintenance, and elevated protein intake for additional preservation of resting energy expenditure and fat free mass. *Journal of Nutrition*. 2013; 143: 591-96.
1196 Solbrig L, Whalley B, Kavanagh DJ, May J, Parkin T, Jones R*, et al.* Functional imagery training versus motivational interviewing for weight loss: a randomised controlled trial of brief individual interventions for overweight and obesity. *International Journal of Obesity*. 2019; 43: 883-94.
1197 Somers TJ, Blumenthal JA, Guilak F, Kraus VB, Schmitt DO, Babyak MA*, et al.* Pain coping skills training and lifestyle behavioral weight management in patients with knee osteoarthritis: a randomized controlled study. *Pain*. 2012; 153: 1199-209.
1198 Son WH, Park HT, Jeon BH, Ha MS. Moderate intensity walking exercises reduce the body mass index and vascular inflammatory factors in postmenopausal women with obesity: a randomized controlled trial. *Scientific Reports*. 2023; 13: 20172.
1199 Son WM, Park JJ. Resistance Band Exercise Training Prevents the Progression of Metabolic Syndrome in Obese Postmenopausal Women. *Journal of Sports Science & Medicine*. 2021; 20: 291-99.
1200 Sone H, Katagiri A, Ishibashi S, Abe R, Saito Y, Murase T*, et al.* Effects of lifestyle modifications on patients with type 2 diabetes: the Japan Diabetes Complications Study (JDCS) study design, baseline analysis and three year-interim report. *Hormone & Metabolic Research*. 2002; 34: 509-15.
1201 Sørensen LB, Greve T, Kreutzer M, Pedersen U, Nielsen CM, Toubro S*, et al.* Weight maintenance through behaviour modification with a cooking course or neurolinguistic programming. *Revue canadienne de la pratique et de la recherche en dietetique [Canadian journal of dietetic practice and research]*. 2011; 72: 181-85.
1202 Sorkin DH, Mavandadi S, Rook KS, Biegler KA, Kilgore D, Dow E*, et al.* Dyadic collaboration in shared health behavior change: the effects of a randomized trial to test a lifestyle intervention for high-risk Latinas. *Health Psychology*. 2014; 33: 566-75.

1203 Sousa N, Mendes R, Abrantes C, Sampaio J, Oliveira J. A randomized 9-month study of blood pressure and body fat responses to aerobic training versus combined aerobic and resistance training in older men. *Experimental Gerontology*. 2013; 48: 727-33.
1204 Sousa N, Mendes R, Abrantes C, Sampaio J, Oliveira J. A randomized study on lipids response to different exercise programs in overweight older men. *International Journal of Sports Medicine*. 2014; 35: 1106-11.
1205 Sperger T, Araujo ACF, Soares CFP. Effect of myofunctional therapy on snoring in obese patients: a randomized trial. *Sleep Science*. 2022; 15: 421-28.
1206 Spring B, Pellegrini CA, Pfammatter A, Duncan JM, Pictor A, McFadden HG*, et al.* Effects of an abbreviated obesity intervention supported by mobile technology: the ENGAGED randomized clinical trial. *Obesity (Silver Spring, Md)*. 2017; 25: 1191-98.
1207 St George A, Bauman A, Johnston A, Farrell G, Chey T, George J. Independent effects of physical activity in patients with nonalcoholic fatty liver disease. *Hepatology (Baltimore, Md)*. 2009; 50: 68-76.
1208 Stamler R, Stamler J, Grimm R, Gosch FC, Elmer P, Dyer A*, et al.* Nutritional therapy for high blood pressure. Final report of a four-year randomized controlled trial--the Hypertension Control Program. *Jama*. 1987; 257: 1484-91.
1209 Stancliffe RA, Thorpe T, Zemel MB. Dairy attentuates oxidative and inflammatory stress in metabolic syndrome. *American Journal of Clinical Nutrition*. 2011; 94: 422-30.
1210 Stefoska-Needham A, Beck EJ, Johnson SK, Batterham MJ, Grant R, Ashton J*, et al.* A Diet Enriched with Red Sorghum Flaked Biscuits, Compared to a Diet Containing White Wheat Flaked Biscuits, Does Not Enhance the Effectiveness of an Energy-Restricted Meal Plan in Overweight and Mildly Obese Adults. *Journal of the American College of Nutrition*. 2017; 36: 184-92.
1211 Steinberg DM, Askew S, Lanpher MG, Foley PB, Levine EL, Bennett GG. The effect of a 'maintain, don’t gain' approach to weight management on depression among Black women: Results from a randomized controlled trial. *American Journal of Public Health*. 2014; 104: 1766-73.
1212 Steinberg DM, Bennett GG, Askew S, Tate DF. Weighing every day matters: daily weighing improves weight loss and adoption of weight control behaviors. *Journal of the academy of nutrition and dietetics*. 2015; 115: 511-18.
1213 Steinberg DM, Levine EL, Askew S, Foley P, Bennett GG. Daily text messaging for weight control among racial and ethnic minority women: randomized controlled pilot study. *Journal of Medical Internet Research*. 2013; 15: e244.
1214 Steinberg DM, Levine EL, Lane I, Askew S, Foley PB, Puleo E*, et al.* Adherence to self-monitoring via interactive voice response technology in an eHealth intervention targeting weight gain prevention among Black women: randomized controlled trial. *Journal of Medical Internet Research*. 2014; 16: e114.
1215 Steinberg DM, Tate DF, Bennett GG, Ennett S, Samuel-Hodge C, Ward DS. The efficacy of a daily self-weighing weight loss intervention using smart scales and e-mail. *Obesity (Silver Spring, Md)*. 2013; 21: 1789-97.
1216 Steinberg DM, Tate DF, Bennett GG, Ennett S, Samuel-Hodge C, Ward DS. Daily self-weighing and adverse psychological outcomes: a randomized controlled trial. *American Journal of Preventive Medicine*. 2014; 46: 24-29.
1217 Stenius-Aarniala B, Poussa T, Kvarnström J, Grönlund EL, Ylikahri M, Mustajoki P. Immediate and long term effects of weight reduction in obese people with asthma: randomised controlled study. *BMJ (Clinical research ed)*. 2000; 320: 827-32.

1218 Stensvold D, Tjønna AE, Skaug EA, Aspenes S, Stølen T, Wisløff U*, et al.* Strength training versus aerobic interval training to modify risk factors of metabolic syndrome. *Journal of applied physiology (Bethesda, Md : 1985)*. 2010; 108: 804-10.
1219 Stern L, Iqbal N, Seshadri P, Chicano KL, Daily DA, McGrory J*, et al.* The effects of low-carbohydrate versus conventional weight loss diets in severely obese adults: one-year follow-up of a randomized trial. *Annals of Internal Medicine*. 2004; 140: 778-85.
1220 Stetic L, Belcic I, Sporis G, Stetic L, Starcevic N. Influence of Physical Activity on the Regulation of Disease of Elderly Persons with Metabolic Syndrome. *International Journal of Environmental Research and Public Health*. 2021; 18: 17.
1221 Stetson B, Schlundt DG, Sbrocco T, Hill JO, Sharp T, Pope-Cordel J. The effects of aerobic exercise on psychological adjustment: a randomized study of sedentary obese women attempting weight loss. *Women & Health*. 1992; 19: 1-14.
1222 Stewart TM, Bachand AR, Han H, Ryan DH, Bray GA, Williamson DA. Body image changes associated with participation in an intensive lifestyle weight loss intervention. *Obesity*. 2011; 19: 1290-95.
1223 Stone SA, Han CJ, Senn T, Korde LA, Allott K, Reding S*, et al.* Sex Hormones in Women With Elevated Breast Cancer Risk Undergoing Weight Loss. *Western Journal of Nursing Research*. 2019; 41: 1602-22.
1224 Stout JR, Fukuda DH, Kendall KL, Smith-Ryan AE, Moon JR, Hoffman JR. beta-Hydroxy-beta-methylbutyrate (HMB) supplementation and resistance exercise significantly reduce abdominal adiposity in healthy elderly men. *Experimental Gerontology*. 2015; 64: 33-34.
1225 Stumm G, Blaik A, Kropf S, Westphal S, Hantke TK, Luley C. Long-Term Follow-Up of the Telemonitoring Weight-Reduction Program "Active Body Control". *Journal of Diabetes Research*. 2016; 2016: 3798729.
1226 Sugawara N, Sagae T, Yasui-Furukori N, Yamazaki M, Shimoda K, Mori T*, et al.* Effects of nutritional education on weight change and metabolic abnormalities among patients with schizophrenia in Japan: A randomized controlled trial. *Journal of Psychiatric Research*. 2018; 97: 77-83.
1227 Sukala WR, Page R, Lonsdale C, Lys I, Rowlands D, Krebs J*, et al.* Exercise improves quality of life in indigenous Polynesian peoples with type 2 diabetes and visceral obesity. *Journal of Physical Activity & Health*. 2013; 10: 699-707.
1228 Sullivan S, Kirk EP, Mittendorfer B, Patterson BW, Klein S. Randomized trial of exercise effect on intrahepatic triglyceride content and lipid kinetics in nonalcoholic fatty liver disease. *Hepatology (Baltimore, Md)*. 2012; 55: 1738-45.
1229 Sun D, Heianza Y, Li X, Shang X, Smith SR, Bray GA*, et al.* Genetic, epigenetic and transcriptional variations at NFATC2IP locus with weight loss in response to diet interventions: the POUNDS Lost Trial. *Diabetes, Obesity & Metabolism*. 2018; 20: 2298-303.
1230 Sun J, Wang Y, Chen X, Chen Y, Feng Y, Zhang X*, et al.* An integrated intervention program to control diabetes in overweight Chinese women and men with type 2 diabetes. *Asia Pacific Journal of Clinical Nutrition*. 2008; 17: 514-24.
1231 Svendsen M, Blomhoff R, Holme I, Tonstad S. The effect of an increased intake of vegetables and fruit on weight loss, blood pressure and antioxidant defense in subjects with sleep related breathing disorders. *European Journal of Clinical Nutrition*. 2007; 61: 1301-11.
1232 Svensson S, Eek F, Christiansen L, Wisen A. The effect of different exercise intensities on health related quality of life in people classified as obese. *European Journal of Physiotherapy*. 2017; 19: 104-15.
1233 Svetkey LP, Clark JM, Funk K, Corsino L, Batch BC, Hollis JF*, et al.* Greater weight loss with increasing age in the weight loss maintenance trial. *Obesity*. 2014; 22: 39-44.

1234 Svetkey LP, Stevens VJ, Brantley PJ, Appel LJ, Hollis JF, Loria CM*, et al.* Comparison of strategies for sustaining weight loss: the weight loss maintenance randomized controlled trial. *Jama*. 2008; 299: 1139-48.
1235 Swencionis C, Wylie-Rosett J, Lent MR, Ginsberg M, Cimino C, Wassertheil-Smoller S*, et al.* Weight change, psychological well-being, and vitality in adults participating in a cognitive-behavioral weight loss program. *Health Psychology*. 2013; 32: 439-46.
1236 Swift DL, Houmard JA, Slentz CA, Kraus WE. Effects of aerobic training with and without weight loss on insulin sensitivity and lipids. *PloS one*. 2018; 13: e0196637.
1237 Swift DL, Johannsen NM, Lavie CJ, Earnest CP, Blair SN, Church TS. Effects of clinically significant weight loss with exercise training on insulin resistance and cardiometabolic adaptations. *Obesity (Silver Spring, Md)*. 2016; 24: 812-19.
1238 Swisher AK, Abraham J, Bonner D, Gilleland D, Hobbs G, Kurian S*, et al.* Exercise and dietary advice intervention for survivors of triple-negative breast cancer: effects on body fat, physical function, quality of life, and adipokine profile. *Supportive Care in Cancer*. 2015; 23: 2995-3003.
1239 Szulinska M, Skrypnik D, Ratajczak M, Karolkiewicz J, Madry E, Musialik K*, et al.* Effects of Endurance and Endurance-strength Exercise on Renal Function in Abdominally Obese Women with Renal Hyperfiltration: A Prospective Randomized Trial. *Biomedical & Environmental Sciences*. 2016; 29: 706-12.
1240 Taheri M, Irandoust K. The Exercise-Induced Weight Loss Improves Self-Reported Quality of Sleep in Obese Elderly Women with Sleep Disorders. *Sleep & Hypnosis*. 2018; 20: 54-59.
1241 Takada A, Nakamura R, Furukawa M, Takahashi Y, Nishimura S, Kosugi S. The relationship between weight loss and time and risk preference parameters: a randomized controlled trial. *Journal of Biosocial Science*. 2011; 43: 481-503.
1242 Takahira M, Noda K, Fukushima M, Zhang B, Mitsutake R, Uehara Y*, et al.* Randomized, double-blind, controlled, comparative trial of formula food containing soy protein vs. milk protein in visceral fat obesity. -FLAVO study. *Circulation Journal*. 2011; 75: 2235-43.
1243 Tamin TZ, Loekito N. Aquatic versus land-based exercise for cardiorespiratory endurance and quality of life in obese patients with knee osteoarthritis: a randomized controlled trial. *Medical journal of indonesia*. 2018; 27: 284-92.
1244 Tang F, Wang W, Wang Y, Lee Y, Lou Q. Moderate resistance training reduces intermuscular adipose tissue and risk factors of atherosclerotic cardiovascular disease for elderly patients with type 2 diabetes. *Diabetes, Obesity & Metabolism*. 2024; 09: 09.
1245 Tang M, Armstrong CL, Leidy HJ, Campbell WW. Normal vs. high-protein weight loss diets in men: effects on body composition and indices of metabolic syndrome. *Obesity*. 2013; 21: E204-10.
1246 Tang MH, Leidy HJ, Campbell WW. Regional, but not total, body composition changes in overweight and obese adults consuming a higher protein, energy-restricted diet are sex specific. *Nutrition Research*. 2013; 33: 629-35.
1247 Tapsell L, Batterham M, Huang XF, Tan SY, Teuss G, Charlton K*, et al.* Short term effects of energy restriction and dietary fat sub-type on weight loss and disease risk factors. *Nutrition, metabolism, and cardiovascular diseases : NMCD*. 2010; 20: 317-25.
1248 Tarraga Marcos ML, Panisello Royo JM, Carbayo Herencia JA, Rosich Domenech N, Alins Presas J, Castell Panisello E*, et al.* Analysis of clinical relevance applied to 3methods of reducing weight in overweight or obesity followed-up for one year. *Hipertension y Riesgo Vascular*. 2018; 35: 5-14.

1249 Tárraga Marcos ML, Panisello Royo JM, Carbayo Herencia JA, Rosich Domenech N, Alins Presas J, Tárraga López PJ. Effect on the lipid parameters of an intervention to reduce weight in overweight and obese patients. *Clinica e investigacion en arteriosclerosis : publicacion oficial de la sociedad espanola de arteriosclerosis*. 2017; 29: 103-10.
1250 Tarraga Marcos ML, Panisello Royo JM, Carbayo-Herencia JA, Rosich Domenech N, Alins Presas J, Castell Panisello E*, et al.* Application of telemedicine in obesity management. *European research in telemedicine*. 2017; 6: 3-12.
1251 Tarraga Marcos ML, Rosich N, Panisello Royo JM, Galvez Casas A, Serrano Selva JP, Rodriguez-Montes JA*, et al.* Efficacy of motivational interventions in the treatment of overweight and obesity. *Nutricion Hospitalaria*. 2014; 30: 741-48.
1252 Tate DF, Jeffery RW, Sherwood NE, Wing RR. Long-term weight losses associated with prescription of higher physical activity goals. Are higher levels of physical activity protective against weight regain? *American Journal of Clinical Nutrition*. 2007; 85: 954-59.
1253 Tay J, Brinkworth GD, Noakes M, Keogh J, Clifton PM. Metabolic effects of weight loss on a very-low-carbohydrate diet compared with an isocaloric high-carbohydrate diet in abdominally obese subjects. *Journal of the American College of Cardiology*. 2008; 51: 59-67.
1254 Taylor CB, Agras WS, Losch M, Plante TG, Burnett K. Improving the effectiveness of computer-assisted weight loss. *Behavior Therapy*. 1991; 22: 229-36.
1255 ter Bogt NC, Milder IE, Bemelmans WJ, Beltman FW, Broer J, Smit AJ*, et al.* Changes in lifestyle habits after counselling by nurse practitioners: 1-year results of the Groningen Overweight and Lifestyle study. *Public Health Nutrition*. 2011; 14: 995-1000.
1256 Thaler L, Wassertheil-Smoller S, Blaufox MD, Oberman A, Langford HG. Effect of withdrawal of antihypertensive drug on depressive mood. *American Journal of Hypertension*. 1993; 6: 1055-62.
1257 Thida T, Yupin A, Sunida P. Effect of Self-Management Exercise Program on Physical Fitness among People with Prehypertension and Obesity: a Quasi Experiment Study. *Pacific rim international journal of nursing research*. 2019; 23: 6-17.
1258 Thomas D, Vydelingum V, Lawrence J. E-mail contact as an effective strategy in the maintenance of weight loss in adults. *Journal of Human Nutrition & Dietetics*. 2011; 24: 32-38.
1259 Thomas JG, Raynor HA, Bond DS, Luke AK, Cardoso CC, Wojtanowski AC*, et al.* Weight loss and frequency of body-weight self-monitoring in an online commercial weight management program with and without a cellular-connected 'smart' scale: a randomized pilot study. *Obesity science and practice*. 2017; 3: 365-72.
1260 Thompson WG, Kuhle CL, Koepp GA, McCrady-Spitzer SK, Levine JA. "Go4Life" exercise counseling, accelerometer feedback, and activity levels in older people. *Archives of Gerontology & Geriatrics*. 2014; 58: 314-19.
1261 Thompson WG, Rostad Holdman N, Janzow DJ, Slezak JM, Morris KL, Zemel MB. Effect of energy-reduced diets high in dairy products and fiber on weight loss in obese adults. *Obesity Research*. 2005; 13: 1344-53.
1262 Thong FS, Hudson R, Ross R, Janssen I, Graham TE. Plasma leptin in moderately obese men: independent effects of weight loss and aerobic exercise. *American journal of physiology Endocrinology and metabolism*. 2000; 279: E307-13.
1263 Thorgeirsson T, Torfadottir JE, Egilsson E, Oddsson S, Gunnarsdottir T, Aspelund T*, et al.* Randomized Trial for Weight Loss Using a Digital Therapeutic Application. *Journal of Diabetes Science & Technology*. 2021: 19322968211000815.

1264 Tiedemann LJ, Meyhofer SM, Francke P, Beck J, Buchel C, Brassen S. Insulin sensitivity in mesolimbic pathways predicts and improves with weight loss in older dieters. *eLife*. 2022; 11: 27.
1265 Tierney AC, McMonagle J, Shaw DI, Gulseth HL, Helal O, Saris WHM*, et al.* Effects of dietary fat modification on insulin sensitivity and on other risk factors of the metabolic syndrome—LIPGENE: A European randomized dietary intervention study. *International Journal of Obesity*. 2011; 35: 800-09.
1266 Tijssen A, Snaterse M, Minneboo M, Lachman S, Reimer WSO, Peters RJ*, et al.* Weight management and determinants of weight change in patients with coronary artery disease. *Heart*. 2021; 107: 1552-59.
1267 Toledo E, Salas-Salvadó J, Donat-Vargas C, Buil-Cosiales P, Estruch R, Ros E*, et al.* Mediterranean Diet and Invasive Breast Cancer Risk Among Women at High Cardiovascular Risk in the PREDIMED Trial: A Randomized Clinical Trial. *JAMA Internal Medicine*. 2015; 175: 1752-60.
1268 Tometich DB, Mosher CE, Winger JG, Badr HJ, Snyder DC, Sloane RJ*, et al.* Effects of diet and exercise on weight-related outcomes for breast cancer survivors and their adult daughters: an analysis of the DAMES trial. *Supportive Care in Cancer*. 2017; 25: 2559-68.
1269 Tonstad S, Malik N, Haddad E. A high-fibre bean-rich diet versus a low-carbohydrate diet for obesity. *Journal of Human Nutrition & Dietetics*. 2014; 27 Suppl 2: 109-16.
1270 Toplak H, Wascher TC. Influence of weight reduction on platelet volume: different effects of a hypocaloric diet and a very low calorie diet. *European Journal of Clinical Investigation*. 1994; 24: 778-80.
1271 Torres MR, Sanjuliani AF. Effects of weight loss from a high-calcium energy-reduced diet on biomarkers of inflammatory stress, fibrinolysis, and endothelial function in obese subjects. *Nutrition (Burbank, Los Angeles County, Calif)*. 2013; 29: 143-51.
1272 Torres SJ, Nowson CA. Effect of a weight-loss program on mental stress-induced cardiovascular responses and recovery. *Nutrition*. 2007; 23: 521-28.
1273 Torres V, Castro Sánchez AM, Matarán Peñarocha GA, Lara Palomo I, Aguilar Ferrándiz ME, Moreno Lorenzo C. Benefits of cognitive behavior therapy and acupressure therapy in obese patients: a randomized clinical trial. *Nutricion Hospitalaria*. 2011; 26: 1018-24.
1274 Tozetto WR, Leonel LD, Turnes T, Del Duca GF. Effects of linear periodization of combined training on quality of life of adults with obesity: a blind randomized controlled trial. *Scientific Reports*. 2022; 12: 10.
1275 Tremblay A, Dutheil F, Drapeau V, Metz L, Lesour B, Chapier R*, et al.* Long-term effects of high-intensity resistance and endurance exercise on plasma leptin and ghrelin in overweight individuals: the RESOLVE Study. *Applied Physiology, Nutrition, & Metabolism = Physiologie Appliquee, Nutrition et Metabolisme*. 2019; 44: 1172-79.
1276 Trepanowski JF, Kroeger CM, Barnosky A, Klempel M, Bhutani S, Hoddy KK*, et al.* Effects of alternate-day fasting or daily calorie restriction on body composition, fat distribution, and circulating adipokines: Secondary analysis of a randomized controlled trial. *Clinical Nutrition*. 2018; Part A. 37: 1871-78.
1277 Treyzon L, Chen S, Hong K, Yan E, Carpenter CL, Thames G*, et al.* A controlled trial of protein enrichment of meal replacements for weight reduction with retention of lean body mass. *Nutrition Journal*. 2008; 7: 23.
1278 Trico D, Masoni MC, Baldi S, Cimbalo N, Sacchetta L, Scozzaro MT*, et al.* Early time-restricted carbohydrate consumption vs conventional dieting in type 2 diabetes: a randomised controlled trial. *Diabetologia*. 2024; 67: 263-74.

1279 Trief PM, Cibula D, Delahanty LM, Weinstock RS. Depression, stress, and weight loss in individuals with metabolic syndrome in SHINE, a DPP translation study. *Obesity*. 2014; 22: 2532-38.
1280 Trovato GM, Pirri C, Martines GF, Tonzuso A, Trovato F, Catalano D. Lifestyle interventions, insulin resistance, and renal artery stiffness in essential hypertension. *Clinical and experimental hypertension (New York, NY : 1993)*. 2010; 32: 262-69.
1281 Truby H, Baic S, deLooy A, Fox KR, Livingstone MB, Logan CM*, et al.* Randomised controlled trial of four commercial weight loss programmes in the UK: initial findings from the BBC "diet trials". *Bmj*. 2006; 332: 1309-14.
1282 Tsai AG, Felton S, Hill JO, Atherly AJ. A randomized pilot trial of a full subsidy vs a partial subsidy for obesity treatment. *Obesity*. 2012; 20: 1838-43.
1283 Tsai AG, Felton S, Wadden TA, Hosokawa PW, Hill JO. A randomized clinical trial of a weight loss maintenance intervention in a primary care population. *Obesity*. 2015; 23: 2015-21.
1284 Tsai AG, Wadden TA, Rogers MA, Day SC, Moore RH, Islam BJ. A primary care intervention for weight loss: Results of a randomized controlled pilot study. *Obesity*. 2010; 18: 1614-18.
1285 Tuomilehto HP, Seppä JM, Partinen MM, Peltonen M, Gylling H, Tuomilehto JO*, et al.* Lifestyle intervention with weight reduction: first-line treatment in mild obstructive sleep apnea. *American journal of respiratory and critical care medicine*. 2009; 179: 320-27.
1286 Turner-McGrievy G, Tate D. Tweets, Apps, and Pods: results of the 6-month Mobile Pounds Off Digitally (Mobile POD) randomized weight-loss intervention among adults. *Journal of Medical Internet Research*. 2011; 13: e120.
1287 Turner-McGrievy GM, Campbell MK, Tate DF, Truesdale KP, Bowling JM, Crosby L. Pounds off digitally study: A randomized podcasting weight-loss intervention. *American Journal of Preventive Medicine*. 2009; 37: 263-69.
1288 Turner-McGrievy GM, Wilcox S, Boutte A, Hutto BE, Singletary C, Muth ER*, et al.* The Dietary Intervention to Enhance Tracking with Mobile Devices (DIET Mobile) Study: A 6-Month Randomized Weight Loss Trial. *Obesity*. 2017; 25: 1336-42.
1289 Tussing-Humphreys L, Thomson J, Onufrak S. A Church-Based Pilot Study Designed to Improve Dietary Quality for Rural, Lower Mississippi Delta, African American Adults. *Journal of Religion & Health*. 2015; 54: 455-69.
1290 Tuthill A, Quinn A, McColgan D, McKenna M, O'Shea D, McKenna TJ. A prospective randomized controlled trial of lifestyle intervention on quality of life and cardiovascular risk score in patients with obesity and type 2 diabetes. *Diabetes, Obesity & Metabolism*. 2007; 9: 917-19.
1291 Tzvetanov I, West-Thielke P, D'Amico G, Johnsen M, Ladik A, Hachaj G*, et al.* A novel and personalized rehabilitation program for obese kidney transplant recipients. *Transplantation proceedings*. 2014; 46: 3431-37.
1292 Ueki K, Sakurai N, Tochikubo O. Weight loss and blood pressure reduction in obese subjects in response to nutritional guidance using information communication technology. *Clinical & Experimental Hypertension (New York)*. 2009; 31: 231-40.
1293 Ueno T, Sugawara H, Sujaku K, Hashimoto O, Tsuji R, Tamaki S*, et al.* Therapeutic effects of restricted diet and exercise in obese patients with fatty liver. *Journal of Hepatology*. 1997; 27: 103-07.
1294 Unick J, Dorfman L, Leahey T, Wing R. A preliminary investigation into whether early intervention can improve weight loss among those initially non-responsive to an internet-based behavioral program. *Journal of Behavioral Medicine*. 2016; 39: 254-61.

1295 Usher K, Park T, Foster K, Buettner P. A randomized controlled trial undertaken to test a nurse-led weight management and exercise intervention designed for people with serious mental illness who take second generation antipsychotics. *Journal of Advanced Nursing*. 2013; 69: 1539-48.
1296 Utter AC, Nieman DC, Shannonhouse EM, Butterworth DE, Nieman CN. Influence of diet and/or exercise on body composition and cardiorespiratory fitness in obese women. *International Journal of Sport Nutrition*. 1998; 8: 213-22.
1297 Utz W, Engeli S, Haufe S, Kast P, Böhnke J, Haas V*, et al.* Moderate dietary weight loss reduces myocardial steatosis in obese and overweight women. *International Journal of Cardiology*. 2013; 167: 905-09.
1298 Uusitupa M, Louheranta A, Lindström J, Valle T, Sundvall J, Eriksson J*, et al.* The Finnish Diabetes Prevention Study. *British Journal of Nutrition*. 2000; 83 Suppl 1: S137-42.
1299 van Aggel-Leijssen DP, Saris WH, Homan M, van Baak MA. The effect of exercise training on beta-adrenergic stimulation of fat metabolism in obese men. *International journal of obesity and related metabolic disorders*. 2001; 25: 16-23.
1300 van Aggel-Leijssen DP, Saris WH, Wagenmakers AJ, Senden JM, van Baak MA. Effect of exercise training at different intensities on fat metabolism of obese men. *Journal of applied physiology (Bethesda, Md : 1985)*. 2002; 92: 1300-09.
1301 Van Bussel IPG, Backx EMP, De Groot C, Tieland M, Muller M, Afman LA. The impact of protein quantity during energy restriction on genome-wide gene expression in adipose tissue of obese humans. *International Journal of Obesity*. 2017; 41: 1114-20.
1302 van Dale D, Schoffelen PF, ten Hoor F, Saris WH. Effects of addition of exercise to energy restriction on 24-hour energy expenditure, sleeping metabolic rate and daily physical activity. *European Journal of Clinical Nutrition*. 1989; 43: 441-51.
1303 van den Akker K, Schyns G, Jansen A. Individual differences in extinction learning predict weight loss after treatment: A pilot study. *European Eating Disorders Review*. 2020; 28: 782-88.
1304 Van den Borne HW, Geertsma F, Westmaas-Jes MM. Effecten van een nieuwe groepsbehandeling voor dikke vrouwen = Effectiveness of a new group treatment for obese women. *Gedrag & Gezondheid: Tijdschrift voor Psychologie en Gezondheid*. 1988; 16: 108-16.
1305 van der Zalm IJB, van der Valk ES, Wester VL, Nagtzaam NMA, van Rossum EFC, Leenen PJM*, et al.* Obesity-associated T-cell and macrophage activation improve partly after a lifestyle intervention. *International Journal of Obesity*. 2020; 44: 1838-50.
1306 van Gool CH, Penninx BW, Kempen GI, Rejeski WJ, Miller GD, van Eijk JT*, et al.* Effects of exercise adherence on physical function among overweight older adults with knee osteoarthritis. *Arthritis & Rheumatism*. 2005; 53: 24-32.
1307 Vander Wal JS, McBurney MI, Moellering N, Marth J, Dhurandhar NV. Moderate-carbohydrate low-fat versus low-carbohydrate high-fat meal replacements for weight loss. *International journal of food sciences and nutrition*. 2007; 58: 321-29.
1308 Varady KA, Bhutani S, Klempel MC, Kroeger CM. Comparison of effects of diet versus exercise weight loss regimens on LDL and HDL particle size in obese adults. *Lipids in health and disease*. 2011; 10: 119.
1309 Vázquez C, Montagna C, Alcaraz F, Balsa JA, Zamarrón I, Arrieta F*, et al.* Meal replacement with a low-calorie diet formula in weight loss maintenance after weight loss induction with diet alone. *European Journal of Clinical Nutrition*. 2009; 63: 1226-32.

1310 Veleba J, Matoulek M, Hill M, Pelikanova T, Kahleova H. "A Vegetarian vs. Conventional Hypocaloric Diet: the Effect on Physical Fitness in Response to Aerobic Exercise in Patients with Type 2 Diabetes." A Parallel Randomized Study. *Nutrients*. 2016; 8.
1311 Velluzzi F, Cossu G, Fosci M, Montisci R, Zaccheddu R, Minerba L*, et al.* Effect of a Low-Moderate Exercise Program on Dysmetabolism in Older Adults: Results of a Randomized Controlled Trial. *Nutrients*. 2022; 14: 15.
1312 Venn BJ, Perry T, Green TJ, Skeaff CM, Aitken W, Moore NJ*, et al.* The effect of increasing consumption of pulses and wholegrains in obese people: a randomized controlled trial. *Journal of the American College of Nutrition*. 2010; 29: 365-72.
1313 Verberne LD, Hendriks MR, Rutten GM, Spronk I, Savelberg HH, Veenhof C*, et al.* Evaluation of a combined lifestyle intervention for overweight and obese patients in primary health care: a quasi-experimental design. *Family Practice*. 2016; 33: 671-77.
1314 Vetter ML, Wadden TA, Chittams J, Diewald LK, Panigrahi E, Volger S*, et al.* Effect of lifestyle intervention on cardiometabolic risk factors: Results of the POWER-UP trial. *International Journal of Obesity*. 2013; 37: S19-S24.
1315 Vila Córcoles A, Llor Vilá C, Pellejá Pellejá J, Gisbert Aguilar A, Jordana Ferrando P, Casacuberta Monge JM. Evaluation of the effectiveness of personalized and frequent dietetic counseling in the treatment of obesity. *Atencion primaria / Sociedad Espanola de Medicina de Familia y Comunitaria*. 1993; 11: 298-300.
1316 Vílchez Barboza V, Paravic Klijn T, Salazar Molina A, Sáez Carrillo KL. Effectiveness of personalized face-to-face and telephone nursing counseling interventions for cardiovascular risk factors: A controlled clinical trial. *Revista latino-americana de enfermagem*. 2016; 24.
1317 Vincent D, McEwen MM, Hepworth JT, Stump CS. The effects of a community-based, culturally tailored diabetes prevention intervention for high-risk adults of Mexican descent. *Diabetes Educator*. 2014; 40: 202-13.
1318 Vinkers CDW, Adriaanse MA, Kroese FM, Ridder DTD. Efficacy of a self-management intervention for weight control in overweight and obese adults: A randomized controlled trial. *Journal of Behavioral Medicine*. 2014; 37: 781-92.
1319 Visek J, Lacigova S, Cechurova D, Rusavy Z. Comparison of a low-glycemic index vs standard diabetic diet. *Biomedical papers of the medical faculty of the university palacky, olomouc, czechoslovakia*. 2014; 158: 112-16.
1320 Vitiello MV, Wilkinson CW, Merriam GR, Moe KE, Prinz PN, Ralph DD*, et al.* Successful 6-month endurance training does not alter insulin-like growth factor-I in healthy older men and women. *Journals of Gerontology Series A-Biological Sciences & Medical Sciences*. 1997; 52: M149-54.
1321 Vitolins MZ, Blackwell CS, Katula JA, Isom SP, Case LD. Long-term weight loss maintenance in the continuation of a randomized diabetes prevention translational study: The healthy living partnerships to prevent diabetes (HELP PD) continuation trial. *Diabetes Care*. 2019; 42: 1653-60.
1322 von Gruenigen VE, Courneya KS, Gibbons HE, Kavanagh MB, Waggoner SE, Lerner E. Feasibility and effectiveness of a lifestyle intervention program in obese endometrial cancer patients: a randomized trial. *Gynecologic Oncology*. 2008; 109: 19-26.
1323 Vreeland B, Minsky S, Menza M, Radler Rigassio D, Roemheld-Hamm B, Stern R. A Program for Managing Weight Gain Associated With Atypical Antipsychotics. *Psychiatric Services*. 2003; 54: 1155-57.
1324 Wachsberg KN, Creden A, Workman M, Lichten A, Basil A, Lee J*, et al.* Inpatient obesity intervention with postdischarge telephone follow-up: A randomized trial. *Journal of Hospital Medicine (Online)*. 2014; 9: 515-20.

1325 Wadden TA, Foster GD, Sarwer DB, Anderson DA, Gladis M, Sanderson RS*, et al.* Dieting and the development of eating disorders in obese women: results of a randomized controlled trial. *American Journal of Clinical Nutrition*. 2004; 80: 560-68.
1326 Wadden TA, Stunkard AJ, Liebschutz J. Three-year follow-up of the treatment of obesity by very low calorie diet, behavior therapy, and their combination. *Journal of consulting and clinical psychology*. 1988; 56: 925-28.
1327 Wadden TA, Vogt RA, Andersen RE, Bartlett SJ, Foster GD, Kuehnel RH*, et al.* Exercise in the treatment of obesity: Effects of four interventions on body composition, resting energy expenditure, appetite, and mood. *Journal of consulting and clinical psychology*. 1997; 65: 269-77.
1328 Wadden TA, Vogt RA, Foster GD, Anderson DA. Exercise and the maintenance of weight loss: 1-year follow-up of a controlled clinical trial. *Journal of consulting and clinical psychology*. 1998; 66: 429-33.
1329 Wadden TA, Volger S, Sarwer DB, Vetter ML, Tsai AG, Berkowitz RI*, et al.* A two- year randomized trial of obesity treatment in primary care practice. *The New England Journal of Medicine*. 2011; 365: 1969-79.
1330 Wagner B, Nagl M, Dölemeyer R, Klinitzke G, Steinig J, Hilbert A*, et al.* Randomized controlled trial of an internet-based cognitive-behavioral treatment program for binge-eating disorder. *Behavior Therapy*. 2016; 47: 500-14.
1331 Waib PH, Gonçalves MI, Barrile SR. Improvements in insulin sensitivity and muscle blood flow in aerobic-trained overweight-obese hypertensive patients are not associated with ambulatory blood pressure. *Journal of clinical hypertension (Greenwich, Conn)*. 2011; 13: 89-96.
1332 Walker KZ, O'Dea K, Johnson L, Sinclair AJ, Piers LS, Nicholson GC*, et al.* Body fat distribution and non-insulin-dependent diabetes: comparison of a fiber-rich, high-carbohydrate, low-fat (23%) diet and a 35% fat diet high in monounsaturated fat. *American Journal of Clinical Nutrition*. 1996; 63: 254-60.
1333 Wanderley FA, Moreira A, Sokhatska O, Palmares C, Moreira P, Sandercock G*, et al.* Differential responses of adiposity, inflammation and autonomic function to aerobic versus resistance training in older adults. *Experimental Gerontology*. 2013; 48: 326-33.
1334 Wang DD, Toledo E, Hruby A, Rosner BA, Willett WC, Qi S*, et al.* Plasma Ceramides, Mediterranean Diet, and Incident Cardiovascular Disease in the PREDIMED Trial. *Circulation*. 2017; 135: 2028-40.
1335 Wang J, Wang S, Henning SM, Qin T, Pan Y, Yang J*, et al.* Mixed tree nut snacks compared to refined carbohydrate snacks resulted in weight loss and increased satiety during both weight loss and weight maintenance: a 24-week randomized controlled trial. *Nutrients*. 2021; 13(5) (no pagination).
1336 Wang T, Huang T, Zheng Y, Rood J, Bray GA, Sacks FM*, et al.* Genetic variation of fasting glucose and changes in glycemia in response to 2-year weight-loss diet intervention: the POUNDS LOST trial. *International Journal of Obesity*. 2016; 40: 1164-69.
1337 Wang Y, Guan Q, Hou X, Zhang X, Zhang H, Xu C*, et al.* Multifactorial Intervention on Type 2 Diabetes (MIDiab) Study: A multicenter, open-label, randomized, parallel controlled, community trial. *Journal of Diabetes*. 2020; 12: 862-64.
1338 Wang ZM, Leng X, Messi ML, Choi SJ, Marsh AP, Nicklas B*, et al.* Relationship of Physical Function to Single Muscle Fiber Contractility in Older Adults: Effects of Resistance Training With and Without Caloric Restriction. *Journals of Gerontology Series A-Biological Sciences & Medical Sciences*. 2019; 74: 412-19.

1339 Wani K, Alfawaz H, Alnaami AM, Sabico S, Khattak MNK, Al-Attas O*, et al.* Effects of a 12-month intensive lifestyle monitoring program in predominantly overweight/obese arab adults with prediabetes. *Nutrients*. 2020; 12.
1340 Watson S, Woodside JV, Ware LJ, Hunter SJ, McGrath A, Cardwell CR*, et al.* Effect of a Web-Based Behavior Change Program on Weight Loss and Cardiovascular Risk Factors in Overweight and Obese Adults at High Risk of Developing Cardiovascular Disease: Randomized Controlled Trial. *Journal of Medical Internet Research*. 2015; 17: e177.
1341 Wattanakorn K, Deenan A, Puapan S, Kraenzle Schneider J. Effects of an Eating Behaviour Modification Program on Thai People with Diabetes and Obesity: a Randomised Clinical Trial. *Pacific rim international journal of nursing research*. 2013; 17: 356-70.
1342 Wauters JP, Busset R, Dayer A, Favre H. Effects of various weight-reducing regimens on body composition in simple obesity. Isotopic analysis. *Schweizerische Medizinische Wochenschrift Journal Suisse de Medecine*. 1970; 100: 1272-78.
1343 Webber KH, Casey EM, Mayes L, Katsumata Y, Mellin L. A comparison of a behavioral weight loss program to a stress management program: a pilot randomized controlled trial. *Nutrition (Burbank, Los Angeles County, Calif)*. 2016; 32: 904-09.
1344 Webber KH, Tate DF, Michael Bowling J. A randomized comparison of two motivationally enhanced Internet behavioral weight loss programs. *Behaviour research and therapy*. 2008; 46: 1090-95.
1345 Weber M, Wyne K. A cognitive/behavioral group intervention for weight loss in patients treated with atypical antipsychotics. *Schizophrenia Research*. 2006; 83: 95-101.
1346 Weber-Rajek M, Radziminska A, Straczynska A, Strojek K, Piekorz Z, Kozakiewicz M*, et al.* A Randomized-Controlled Trial Pilot Study Examining the Effect of Pelvic Floor Muscle Training on the Irisin Concentration in Overweight or Obese Elderly Women with Stress Urinary Incontinence. *BioMed Research International*. 2019; 2019: 7356187.
1347 Wei W, Jiang W, Gu W, Wu H, Jiang H, Li G*, et al.* The joint effect of energy reduction with calcium supplementation on the risk factors of type 2 diabetes in the overweight population: a two-year randomized controlled trial. *Aging*. 2021; 13: 5571-84.
1348 Weickert MO, Hattersley JG, Kyrou I, Arafat AM, Rudovich N, Roden M*, et al.* Effects of supplemented isoenergetic diets varying in cereal fiber and protein content on the bile acid metabolic signature and relation to insulin resistance. *Nutrition & Diabetes*. 2018; 8: 11.
1349 Weickert MO, Roden M, Isken F, Hoffmann D, Nowotny P, Osterhoff M*, et al.* Effects of supplemented isoenergetic diets differing in cereal fiber and protein content on insulin sensitivity in overweight humans. *American Journal of Clinical Nutrition*. 2011; 94: 459-71.
1350 Weijs PJM, Wolfe RR. Exploration of the protein requirement during weight loss in obese older adults. *Clinical Nutrition*. 2016; 35: 394-98.
1351 Weinstock RS, Dai H, Wadden TA. Diet and exercise in the treatment of obesity: effects of 3 interventions on insulin resistance. *Archives of Internal Medicine*. 1998; 158: 2477-83.
1352 Welsh P, Cezard G, Gill JM, Wallia S, Douglas A, Sheikh A*, et al.* Associations between weight change and biomarkers of cardiometabolic risk in South Asians: Secondary analyses of the PODOSA trial. *International Journal of Obesity*. 2016; 40: 1005-11.
1353 Werrij MQ, Jansen A, Mulkens S, Elgersma HJ, Ament AJ, Hospers HJ. Adding cognitive therapy to dietetic treatment is associated with less relapse in obesity. *Journal of Psychosomatic Research*. 2009; 67: 315-24.
1354 West DS, DiLillo V, Bursac Z, Gore SA, Greene PG. Motivational interviewing improves weight loss in women with type 2 diabetes. *Diabetes Care*. 2007; 30: 1081-87.

1355 West DS, Gorin AA, Subak LL, Foster G, Bragg C, Hecht J, *et al.* A motivation-focused weight loss maintenance program is an effective alternative to a skill-based approach. *International Journal of Obesity*. 2011; 35: 259-69.
1356 West DS, Harvey JR, Krukowski RA, Prewitt TE, Priest J, Ashikaga T. Do individual, online motivational interviewing chat sessions enhance weight loss in a group-based, online weight control program? *Obesity*. 2016; 24: 2334-40.
1357 West DS, Stansbury M, Krukowski RA, Harvey J. Enhancing group-based internet obesity treatment: A pilot RCT comparing video and text-based chat. *Obesity Science and Practice*. 2019; 5: 513-20.
1358 Westman EC, Yancy Jr WS, Mavropoulos JC, Marquart M, McDuffie JR. The effect of a low-carbohydrate, ketogenic diet versus a low-glycemic index diet on glycemic control in type 2 diabetes mellitus. *Nutrition & Metabolism*. 2008; 5: 36.
1359 Whelton PK, Appel LJ, Espeland MA, Applegate WB, Ettinger Jr WH, Kostis JB*, et al.* Sodium reduction and weight loss in the treatment of hypertension in older persons: A randomized controlled trial of nonpharmacologic interventions in the elderly (TONE). *Journal of the american medical association*. 1998; 279: 839-46.
1360 White DK, Neogi T, Rejeski WJ, Walkup MP, Lewis CE, Nevitt MC*, et al.* Can an intensive diet and exercise program prevent knee pain among overweight adults at high risk? *Arthritis Care & Research*. 2015; 67: 965-71.
1361 White N, Carnahan J, Nugent CA, Iwaoka T, Dodson MA. Management of obese patients with diabetes mellitus: comparison of advice education with group management. *Diabetes Care*. 1986; 9: 490-96.
1362 Whited MC, Olendzki E, Ma Y, Waring ME, Schneider KL, Appelhans BM*, et al.* Obstructive sleep apnea and weight loss treatment outcome among adults with metabolic syndrome. *Health Psychology*. 2016; 35: 1316-19.
1363 Wilfley DE, Welch RR, Stein RI, Spurrell EB, Cohen LR, Saelens BE*, et al.* A randomized comparison of group cognitive-behavioral therapy and group interpersonal psychotherapy for the treatment of overweight individuals with binge-eating disorder. *Archives of General Psychiatry*. 2002; 59: 713-21.
1364 Willaing I, Ladelund S, Jorgensen T, Simonsen T, Nielsen LM. Nutritional counselling in primary health care: a randomized comparison of an intervention by general practitioner or dietician. *European Journal of Cardiovascular Prevention & Rehabilitation*. 2004; 11: 513-20.
1365 Williams A, Wiggers J, O'Brien KM, Wolfenden L, Yoong SL, Hodder RK*, et al.* Effectiveness of a healthy lifestyle intervention for chronic low back pain: a randomised controlled trial. *Pain*. 2018; 159: 1137-46.
1366 Williams KV, Mullen ML, Kelley DE, Wing RR. The effect of short periods of caloric restriction on weight loss and glycemic control in type 2 diabetes. *Diabetes Care*. 1998; 21: 2-8.
1367 Williams PT, Krauss RM, Stefanick ML, Vranizan KM, Wood PD. Effects of low-fat diet, calorie restriction, and running on lipoprotein subfraction concentrations in moderately overweight men. *Metabolism: clinical and experimental*. 1994; 43: 655-63.
1368 Williams PT, Krauss RM, Vranizan KM, Albers JJ, Wood PD. Effects of weight-loss by exercise and by diet on apolipoproteins A-I and A-II and the particle-size distribution of high-density lipoproteins in men. *Metabolism: clinical and experimental*. 1992; 41: 441-49.
1369 Willmann C, Heni M, Linder K, Wagner R, Stefan N, Machann J*, et al.* Potential effects of reduced red meat compared with increased fiber intake on glucose metabolism and liver fat content: a randomized and controlled dietary intervention study. *American Journal of Clinical Nutrition*. 2019; 109: 288-96.

1370 Wing D, Eyler LT, Lenze EJ, Wetherell JL, Nichols JF, Meeusen R*, et al.* Fatness but Not Fitness Linked to BrainAge: Longitudinal Changes in Brain Aging during an Exercise Intervention. *Medicine & Science in Sports & Exercise*. 2024; 56: 655-62.
1371 Wing RR, Anglin K. Effectiveness of a behavioral weight control program for blacks and whites with NIDDM. *Diabetes Care*. 1996; 19: 409-13.
1372 Wing RR, Blair EH, Bononi P, Marcus MD, Watanabe R, Bergman RN. Caloric restriction per se is a significant factor in improvements in glycemic control and insulin sensitivity during weight loss in obese NIDDM patients. *Diabetes Care*. 1994; 17: 30-36.
1373 Wing RR, Bolin P, Brancati FL, Bray GA, Clark JM, Coday M*, et al.* Cardiovascular effects of intensive lifestyle intervention in type 2 diabetes. *New England Journal of Medicine*. 2013; 369: 145-54.
1374 Wing RR, Epstein LH, Marcus MD, Koeske R. Intermittent low-calorie regimen and booster sessions in the treatment of obesity. *Behaviour research and therapy*. 1984; 22: 445-49.
1375 Wing RR, Epstein LH, Nowalk MP, Scott N, Koeske R, Hagg S. Does self-monitoring of blood glucose levels improve dietary compliance for obese patients with type II diabetes? *American Journal of Medicine*. 1986; 81: 830-36.
1376 Wing RR, Jeffery RW, Burton LR, Thorson C, Nissinoff KS, Baxter JE. Food provision vs structured meal plans in the behavioral treatment of obesity. *International journal of obesity and related metabolic disorders*. 1996; 20: 56-62.
1377 Wing RR, Marcus MD, Epstein LH, Jawad A. A "family-based" approach to the treatment of obese type II diabetic patients. *Journal of Consulting & Clinical Psychology*. 1991; 59: 156-62.
1378 Wing RR, Venditti E, Jakicic JM, Polley BA, Lang W. Lifestyle intervention in overweight individuals with a family history of diabetes. *Diabetes Care*. 1998; 21: 350-59.
1379 Winger JG, Mosher CE, Rand KL, Morey MC, Snyder DC, Demark-Wahnefried W. Diet and exercise intervention adherence and health-related outcomes among older long-term breast, prostate, and colorectal cancer survivors. *Annals of Behavioral Medicine*. 2014; 48: 235-45.
1380 Wohlgemuth SE, Lees HA, Marzetti E, Manini TM, Aranda JM, Daniels MJ*, et al.* An exploratory analysis of the effects of a weight loss plus exercise program on cellular quality control mechanisms in older overweight women. *Rejuvenation Research*. 2011; 14: 315-24.
1381 Wolf AM, Conaway MR, Crowther JQ, Hazen KY, J LN, Oneida B*, et al.* Translating lifestyle intervention to practice in obese patients with type 2 diabetes: improving Control with Activity and Nutrition (ICAN) study. *Diabetes Care*. 2004; 27: 1570-76.
1382 Wolf S, Foley S, Budiman-Mak E, Moritz T, O'Connell S, Jelinek C*, et al.* Predictors of weight loss in overweight veterans with knee osteoarthritis who participated in a clinical trial. *Journal of Rehabilitation Research & Development*. 2010; 47: 171-81.
1383 Wood PD, Stefanick ML, Dreon DM, Frey-Hewitt B, Garay SC, Williams PT*, et al.* Changes in plasma lipids and lipoproteins in overweight men during weight loss through dieting as compared with exercise. *New England Journal of Medicine*. 1988; 319: 1173-79.
1384 Wright CS, Zhou J, Sayer RD, Kim JE, Campbell WW. Effects of a High-Protein Diet Including Whole Eggs on Muscle Composition and Indices of Cardiometabolic Health and Systemic Inflammation in Older Adults with Overweight or Obesity: A Randomized Controlled Trial. *Nutrients*. 2018; 10: 23.
1385 Wright J, Wood B, Hale G. Evaluation of group versus individual nutrition education in overweight patients with myocardial infarction. *Australian and New Zealand journal of medicine*. 1981; 11: 497-501.

1386 Wylie-Rosett J, Swencionis C, Ginsberg M, Cimino C, Wassertheil-Smoller S, Caban A, *et al.* Computerized weight loss intervention optimizes staff time: the clinical and cost results of a controlled clinical trial conducted in a managed care setting. *Journal of the American Dietetic Association*. 2001; 101: 1155-62; quiz 63-54.
1387 Wylie-Rosett J, Swencionis C, Peters MH, Dornelas EA, Edlen-Nezin L, Kelly LD, *et al.* A weight reduction intervention that optimizes use of practitioner's time, lowers glucose level, and raises HDL cholesterol level in older adults. *Journal of the American Dietetic Association*. 1994; 94: 37-42.
1388 Xu DF, Sun JQ, Chen M, Chen YQ, Xie H, Sun WJ, *et al.* Effects of lifestyle intervention and meal replacement on glycaemic and body-weight control in Chinese subjects with impaired glucose regulation: a 1-year randomised controlled trial. *British Journal of Nutrition*. 2013; 109: 487-92.
1389 Xu X, Leahey TM, Boguszewski K, Krupel K, Mailloux KA, Wing RR. Self-Expansion is Associated with Better Adherence and Obesity Treatment Outcomes in Adults. *Annals of Behavioral Medicine*. 2017; 51: 13-17.
1390 Xuelian J, Xiyun F, Rui W, Fengqin G, Chenping H, Jiang X, *et al.* The effect of care intervention for obese patients with type II diabetes. *Medicine*. 2017; 96: 1-5.
1391 Yamauchi K, Katayama T, Yamauchi T, Kotani K, Tsuzaki K, Takahashi K, *et al.* Efficacy of a 3-month lifestyle intervention program using a Japanese-style healthy plate on body weight in overweight and obese diabetic Japanese subjects: A randomized controlled trial. *Nutrition Journal*. 2014; 13.
1392 Yan J, Dai X, Feng J, Yuan X, Li J, Yang L, *et al.* Effect of 12-Month Resistance Training on Changes in Abdominal Adipose Tissue and Metabolic Variables in Patients with Prediabetes: a Randomized Controlled Trial. *Journal of Diabetes Research*. 2019; 2019.
1393 Yancy WS, Mayer SB, Coffman CJ, Smith VA, Kolotkin RL, Geiselman PJ, *et al.* Effect of Allowing Choice of Diet on Weight Loss: a Randomized Trial. *Annals of Internal Medicine*. 2015; 162: 805-14.
1394 Yancy WS, Jr., Olsen MK, Guyton JR, Bakst RP, Westman EC. A low-carbohydrate, ketogenic diet versus a low-fat diet to treat obesity and hyperlipidemia: a randomized, controlled trial. *Annals of Internal Medicine*. 2004; 140: 769-77.
1395 Yang Z, Yu Z, Jiang Y, Bai Y, Miller-Kovach K, Zhao W, *et al.* Evaluation of a community-based behavioral weight loss program in Chinese adults: A randomized controlled trial. *Obesity*. 2016; 24: 1464-70.
1396 Yanni AE, Kokkinos A, Psychogiou G, Binou P, Kartsioti K, Chatzigeorgiou A, *et al.* Daily consumption of fruit-flavored yoghurt enriched with vitamins B contributes to lower energy intake and body weight reduction, in type 2 diabetic patients: a randomized clinical trial. *Food & Function*. 2019; 10: 7435-43.
1397 Yarborough BJH, Leo MC, Yarborough MT, Stumbo S, Janoff SL, Perrin NA, *et al.* Improvement in body image, perceived health, and health-related self-efficacy among people with serious mental illness: The STRIDE study. *Psychiatric Services*. 2016; 67: 296-301.
1398 Yardley L, Ware LJ, Smith ER, Williams S, Bradbury KJ, Arden-Close EJ, *et al.* Randomised controlled feasibility trial of a web-based weight management intervention with nurse support for obese patients in primary care. *The International Journal of Behavioral Nutrition and Physical Activity*. 2014; 11.
1399 Yates T, Davies M, Gorely T, Bull F, Khunti K. Effectiveness of a pragmatic education program designed to promote walking activity in individuals with impaired glucose tolerance: a randomized controlled trial. *Diabetes Care*. 2009; 32: 1404-10.

1400 Yeh MC, Rodriguez E, Nawaz H, Gonzalez M, Nakamoto D, Katz DL. Technical skills for weight loss: 2-y follow-up results of a randomized trial. *International Journal of Obesity*. 2003; 27: 1500-06.
1401 Yokose C, McCormick N, Rai SK, Lu N, Curhan G, Schwarzfuchs D*, et al.* Effects of Low-Fat, Mediterranean, or Low-Carbohydrate Weight Loss Diets on Serum Urate and Cardiometabolic Risk Factors: A Secondary Analysis of the Dietary Intervention Randomized Controlled Trial (DIRECT). *Diabetes Care*. 2020; 43: 2812-20.
1402 Yoo S, Kim H, Cho HI. Improvements in the metabolic syndrome and stages of change for lifestyle behaviors in korean older adults. *Osong Public Health & Research Perspectives*. 2012; 3: 85-93.
1403 Yoshimura E, Kumahara H, Tobina T, Matsuda T, Ayabe M, Kiyonaga A*, et al.* Lifestyle intervention involving calorie restriction with or without aerobic exercise training improves liver fat in adults with visceral adiposity. *Journal of Obesity*. 2014; 2014: 197216.
1404 Yoshimura E, Kumahara H, Tobina T, Matsuda T, Watabe K, Matono S*, et al.* Aerobic exercise attenuates the loss of skeletal muscle during energy restriction in adults with visceral adiposity. *Obesity Facts*. 2014; 7: 26-35.
1405 Young AS, Cohen AN, Goldberg R, Hellemann G, Kreyenbuhl J, Niv N*, et al.* Improving Weight in People with Serious Mental Illness: The Effectiveness of Computerized Services with Peer Coaches. *Journal of General Internal Medicine*. 2017; 32: 48-55.
1406 Young MD, Callister R, Collins CE, Plotnikoff RC, Aguiar EJ, Morgan PJ. Efficacy of a gender-tailored intervention to prevent weight regain in men over 3 years: A weight loss maintenance RCT. *Obesity*. 2017; 25: 56-65.
1407 Young MD, Drew RJ, Kay-Lambkin F, Collins CE, Callister R, Kelly BJ*, et al.* Impact of a self-guided, eHealth program targeting weight loss and depression in men: A randomized trial. *Journal of Consulting and Clinical Psychology*. 2021; 89(8): 682-94.
1408 Yu CM, Li LS, Ho HH, Lau CP. Long-term changes in exercise capacity, quality of life, body anthropometry, and lipid profiles after a cardiac rehabilitation program in obese patients with coronary heart disease. *American Journal of Cardiology*. 2003; 91: 321-25.
1409 Yu R, Yan LL, Wang H, Ke L, Yang Z, Gong E*, et al.* Effectiveness of a community-based individualized lifestyle intervention among older adults with diabetes and hypertension, Tianjin, China, 2008-2009. *Preventing Chronic Disease*. 2014; 11: E84.
1410 Yubero-Serrano EM, Delgado-Lista J, Tierney AC, Perez-Martinez P, Garcia-Rios A, Alcala-Diaz JF*, et al.* Insulin resistance determines a differential response to changes in dietary fat modification on metabolic syndrome risk factors: the LIPGENE study. *American Journal of Clinical Nutrition*. 2015; 102: 1509-17.
1411 Zelber-Sagi S, Buch A, Yeshua H, Vaisman N, Webb M, Harari G*, et al.* Effect of resistance training on non-alcoholic fatty-liver disease a randomized-clinical trial. *World Journal of Gastroenterology*. 2014; 20: 4382-92.
1412 Zemel MB, Richards J, Milstead A, Campbell P. Effects of calcium and dairy on body composition and weight loss in African-American adults. *Obesity Research*. 2005; 13: 1218-25.
1413 Zhang H, Tong TK, Qiu W, Wang J, Nie J, He Y. Effect of high-intensity interval training protocol on abdominal fat reduction in overweight Chinese women: A randomized controlled trial. *Kinesiology*. 2015; 47: 57-66.
1414 Zhang HJ, Pan LL, Ma ZM, Chen Z, Huang ZF, Sun Q*, et al.* Long-term effect of exercise on improving fatty liver and cardiovascular risk factors in obese adults: a 1-year follow-up study. *Diabetes, Obesity & Metabolism*. 2017; 19: 284-89.

1415 Zhang P, Hire D, Espeland MA, Knowler WC, Thomas S, Tsai AG*, et al.* Impact of intensive lifestyle intervention on preference-based quality of life in type 2 diabetes: Results from the Look AHEAD Trial. *Obesity*. 2016; 24: 856-64.
1416 Zhang X, Qi Q, Bray GA, Hu FB, Sacks FM, Qi L. APOA5 genotype modulates 2-y changes in lipid profile in response to weight-loss diet intervention: the Pounds Lost Trial. *American Journal of Clinical Nutrition*. 2012; 96: 917-22.
1417 Zhang X, Qi Q, Liang J, Hu FB, Sacks FM, Qi L. Neuropeptide y promoter polymorphism modifies effects of a weight-loss diet on 2-year changes of blood pressure: The preventing overweight using novel dietary strategies trial. *Hypertension*. 2012; 60: 1169-75.
1418 Zhang X, Qi Q, Zhang C, Hu FB, Sacks FM, Qi L. FTO genotype and 2-year change in body composition and fat distribution in response to weight-loss diets: The POUNDS LOST trial. *Diabetes*. 2012; 61: 3005-11.
1419 Zheng H, Lorenzen JK, Astrup A, Larsen LH, Yde CC, Clausen MR*, et al.* Metabolic Effects of a 24-Week Energy-Restricted Intervention Combined with Low or High Dairy Intake in Overweight Women: an NMR-Based Metabolomics Investigation. *Nutrients*. 2016; 8: 108.
1420 Zwickert K, Rieger E, Swinbourne J, Manns C, McAulay C, Gibson AA*, et al.* High or low intensity text-messaging combined with group treatment equally promote weight loss maintenance in obese adults. *Obesity Research & Clinical Practice*. 2016; 10: 680-91.
1421 潘晓, 叶珊珊, 黄杏阁. KH 健康教育对社区中老年肥胖症病人的干预效果研究. *Chinese Nursing Research*. 2017; 31: 938-40.
1422 Ard JD, Cook M, Rushing J, Frain A, Beavers K, Miller G*, et al.* Impact on weight and physical function of intensive medical weight loss in older adults with stage II and III obesity. *Obesity*. 2016; 24: 1861-66.
1423 Avila JJ, Gutierres JA, Sheehy ME, Lofgren IE, Delmonico MJ. Effect of moderate intensity resistance training during weight loss on body composition and physical performance in overweight older adults. *European Journal of Applied Physiology*. 2010; 109: 517-25.
1424 Ballin M, Lundberg E, Sorlen N, Nordstrom P, Hult A, Nordstrom A. Effects of Interval Training on Visceral Adipose Tissue in Centrally Obese 70-Year-Old Individuals: A Randomized Controlled Trial. *Journal of the American Geriatrics Society*. 2019; 67: 1625-31.
1425 Ballin M, Lundberg E, Sorlen N, Nordstrom P, Hult A, Nordstrom A. Effects of interval training on quality of life and cardiometabolic risk markers in older adults: a randomized controlled trial. *Clinical Interventions In Aging*. 2019; 14: 1589-99.
1426 de Almeida SS, Teixeira EL, Merege-Filho CAA, Dozzi Brucki SM, de Salles Painelli V. Acute effects of resistance and functional-task exercises on executive function of obese older adults: Two counterbalanced, crossover, randomized exploratory studies. *Sport, Exercise, and Performance Psychology*. 2020.
1427 Kleinloog JPD, Mensink RP, Roodt JO, Thijssen DHJ, Hesselink MKC, Joris PJ. Aerobic exercise training improves not only brachial artery flow-mediated vasodilatation but also carotid artery reactivity: A randomized controlled, cross-over trial in older men. *Physiological Reports*. 2022; 10: e15395.
1428 McNamara RJ, McKeough ZJ, McKenzie DK, Alison JA. Obesity in COPD: the effect of water-based exercise. *European Respiratory Journal*. 2013; 42: 1737-39.
1429 Nijssen KM, Mensink RP, Plat J, Ivanov D, Preissl H, Joris PJ. Mixed nut consumption improves brain insulin sensitivity: a randomized, single-blinded, controlled, crossover trial in older adults with overweight or obesity. *American Journal of Clinical Nutrition*. 2024; 119: 314-23.

1430 Krukowski RA, Pope RA, Love S, Lensing S, Felix HC, Prewitt TE*, et al.* Examination of costs for a lay health educator-delivered translation of the Diabetes Prevention Program in senior centers. *Preventive Medicine*. 2013; 57: 400-02.
1431 Sjogren P, Fisher R, Kallings L, Svenson U, Roos G, Hellenius ML. Stand up for health--avoiding sedentary behaviour might lengthen your telomeres: secondary outcomes from a physical activity RCT in older people. *British Journal of Sports Medicine*. 2014; 48: 1407-09.
1432 Tuominen M, Suorsa K, Pentti J, Koski P, Stenholm S, Leskinen T. The Impact of a 12-Month Activity Tracker Intervention on Activity Behavior Across Body Mass Index Subgroups Among Recent Retirees: Post Hoc Analysis of a Randomized Controlled Trial. *Journal of Physical Activity & Health*. 2021; 18: 1563-69.
1433 Venditti EM, Zgibor JC, Vander Bilt J, Kieffer LA, Boudreau RM, Burke LE*, et al.* Mobility and Vitality Lifestyle Program (MOVE UP): A Community Health Worker Intervention for Older Adults With Obesity to Improve Weight, Health, and Physical Function. *Innovation in Aging*. 2018; 2: 17.
1434 Bester CL, Beukes RB, Grobler JJ, Schoeman CD. Die ontwikkeling en toepassing van n massaverminderingsprogram wat op gedragsmodifikasie berus = The development and application of a mass reduction programme based on behaviour modification. *South African Journal of Psychology*. 1985; 15: 42-45.
1435 Erickson ML, Malin SK, Wang Z, Brown JM, Hazen SL, Kirwan JP. Effects of Lifestyle Intervention on Plasma Trimethylamine N-Oxide in Obese Adults. *Nutrients*. 2019; 11: 16.
1436 Lynch DH, Rushing BR, Pathmasiri W, McRitchie S, Batchek DJ, Petersen CL*, et al.* Baseline Serum Biomarkers Predict Response to a Weight Loss Intervention in Older Adults with Obesity: A Pilot Study. *Metabolites*. 2023; 13: 17.
1437 Rica RL, Carneiro RMM, Serra AJ, Rodriguez D, Pontes FL, Bocalini DS. Effects of water-based exercise in obese older women: Impact of short-term follow-up study on anthropometric, functional fitness and quality of life parameters. *Geriatrics & gerontology international*. 2013; 13: 209-14.
1438 Tse KK, Neiberg RH, Beavers DP, Kritchevsky SB, Nicklas BJ, Kitzman DW*, et al.* Predictors of Clinically Meaningful Gait Speed Response to Caloric Restriction Among Older Adults Participating in Weight Loss Interventions. *Journals of Gerontology Series a-Biological Sciences and Medical Sciences*. 6.
1439 Xu F, Letendre J, Bekke J, Beebe N, Mahler L, Lofgren IE, et al. Impact of a program of Tai Chi plus behaviorally based dietary weight loss on physical functioning and coronary heart disease risk factors: a community-based study in obese older women. Journal of Nutrition in Gerontology & Geriatrics. 2015; 34: 50-65.
1440 In French AGIR Means to Get Into Action. This is the Generic Title of Our Study. In: Centre Hospitalier Universitaire V (ed.): 2010.
1441 Does Lifestyle Intervention Improve Bone Quality in Obese Older Adults? In: National Institute of D, Digestive, Kidney D, Michael EDVAMC, Biomedical Research Institute of New M (eds.): 2017.
1442 Weight Loss in Elderly Patients With Atrial Fibrillation (LOSE-AF): A Randomised Controlled Trial. 2018.
1443 Changes in Weight and Physical Function for Older African American Women in a National, Nonprofit, Community-Based, Peer-Led, Weight Loss Program. In: National Institute on A (ed.): 2019.
1444 Lifestyle Intervention Plus Metformin to Treat Frailty in Older Veterans With Obesity. In: Michael EDVAMC, Baylor College of M (eds.): 2020.

1445 Effects of Long-term Consumption of Two Plant-based Dietary Supplements on Cardiovascular Health and Low-grade Inflammation in the Elderly. In: Medical University of G (ed.): 2021.
1446 Beavers KM, Wolle BR, Ard JD, Beavers DP, Biehl O, Brubaker PH, et al. The Bone, Exercise, Alendronate, and Caloric Restriction (BEACON) trial design and methods. Contemporary Clinical Trials. 2024; 146: 107692.
1447 Fanning J, Brooks AK, Irby MB, N'Dah KW, Rejeski WJ. Associations Between Patterns of Daily Stepping Behavior, Health-Related Quality of Life, and Pain Symptoms Among Older Adults with Chronic Pain: A Secondary Analysis of Two Randomized Controlled Trials. Clinical Interventions In Aging. 2024; 19: 459–70.
1448 Fanning J, Brooks AK, Robison JT, Irby MB, Ford S, N'Dah K, et al. Associations between patterns of physical activity, pain intensity, and interference among older adults with chronic pain: a secondary analysis of two randomized controlled trials. Frontiers in Aging. 2023; 4: 1216942.
1449 Johansson J, Deraas TS, Hopstock LA, Henriksen A, Grimsgaard S. Improving and preserving cardiorespiratory fitness, muscle strength and adiposity through a complex lifestyle intervention in community-dwelling older adults with elevated cardiometabolic risk: study protocol for the RESTART randomised controlled trial. BMJ Open. 2025; 15: e095810.
1450 Lynch SD, Howard M, Beavers DP, Lenchik L, Barnard R, Stapleton JR, et al. Musculoskeletal characteristics in older adults with overweight or obesity: INVEST in Bone Health trial baseline analysis. Obesity. 2025; 06: 06.
1451 Stowe CL, Kennedy K, Emilson SS, Neiberg RH, Kritchevsky SB, Miller ME, et al. The Health, Aging, and Later-Life Outcomes Pilot Study: Design, recruitment, and participants' baseline characteristics. Contemporary Clinical Trials. 2025; 157: 108049.
1452 Bancks MP, Lovato J, Balasubramanyam A, Coday M, Johnson KC, Munshi M, et al. Association of Type 2 Diabetes Subgroups With Cognitive Status Without Modification From Lifestyle Intervention. Journal of Clinical Endocrinology & Metabolism. 2023; 108: e334–e42.
1453 Bliss ES, Wong RHX, Howe PRC, Mills DE. The Effects of Aerobic Exercise Training on Cerebrovascular and Cognitive Function in Sedentary, Obese, Older Adults. Frontiers in aging neuroscience. 2022; 14: 892343.
1454 Clark JS, Dyer KA, Davis CR, Shivappa N, Hebert JR, Woodman R, et al. Adherence to a Mediterranean Diet for 6 Months Improves the Dietary Inflammatory Index in a Western Population: Results from the MedLey Study. Nutrients. 2023; 15: 11.
1455 Di Rosa C, Lattanzi G, Spiezia C, Imperia E, Piccirilli S, Beato I, et al. Mediterranean Diet versus Very Low-Calorie Ketogenic Diet: Effects of Reaching 5% Body Weight Loss on Body Composition in Subjects with Overweight and with Obesity-A Cohort Study. International Journal of Environmental Research & Public Health [Electronic Resource]. 2022; 19: 11.
1456 Godziuk K, Prado CM, Forhan M. Protocol for the POMELO (Prevention Of MusclE Loss in Osteoarthritis) randomized pilot feasibility trial. Osteoarthritis and Cartilage Open. 2022; 4: 100312.
1457 Grajek M, Gdanska A, Krupa-Kotara K, Glogowska-Ligus J, Kobza J. Global Self-Esteem, Physical Activity, and Body Composition Changes Following a 12-Week Dietary and Physical Activity Intervention in Older Women. International Journal of Environmental Research & Public Health [Electronic Resource]. 2022; 19: 14.

1458 Hammami N, Mechraoui A, Hattabi S, Forte P, Sampaio T, Sortwell A, et al. Concentric Isokinetic Strengthening Program's Impact on Knee Biomechanical Parameters, Physical Performance and Quality of Life in Overweight/Obese Women with Chronic Meniscal Lesions. Healthcare. 2023; 11: 20.
1459 Katzmarzyk PT, Martin CK, Newton RL, Jr., Apolzan JW, Arnold CL, Davis TC, et al. Patient Centered Outcomes Research Institute. 2021; 06: 06.
1460 Kim G, Kim S, Lee YB, Jin SM, Hur KY, Kim JH. A randomized controlled trial of an app-based intervention on physical activity and glycemic control in people with type 2 diabetes. BMC Medicine. 2024; 22: 185.
1461 McLaren CW, Pearl RL, Smith GE, Anton SD. A Secondary Analysis of Caloric Restriction and Exercise Effects on Cognitive Function in Functionally Limited Postmenopausal Women with Overweight or Obesity. Nutrients. 2025; 17: 22.
1462 Messier SP, Monroe MG, Callahan LF, Mihalko SL, Beavers DP, Queen K, et al. Disparities Between Rural and Urban Communities: Response to 18 Months of Diet and Exercise Versus Control for Knee Osteoarthritis and Overweight or Obesity. Arthritis care & research. 2025; 77: 69–76.
1463 Nosrani SE, Tartibian B, Eslami R, Farinha C, Serrano J, Ferreira JP, et al. The Effects of Combined Aquatic Exercise on Physical Performance and Metabolic Indices in Overweight Healthy Older Adults. International Journal of Exercise Science. 2023; 16: 1499–513.
1464 Rossello X, Ramallal R, Romaguera D, Alonso-Gomez AM, Alonso A, Tojal-Sierra L, et al. Effect of an intensive lifestyle intervention on the structural and functional substrate for atrial fibrillation in people with metabolic syndrome. European Journal of Preventive Cardiology. 2024; 31: 629–39.
1465 Sardeli AV, Castro A, Gadelha VB, Santos WMD, Lord JM, Cavaglieri CR, et al. Metabolomic Response throughout 16 Weeks of Combined Aerobic and Resistance Exercise Training in Older Women with Metabolic Syndrome. Metabolites. 2022; 12: 30.
1466 Sperger T, Araujo ACF, Soares CFP. Effect of myofunctional therapy on snoring in obese patients: a randomized trial. Sleep Science. 2022; 15: 421–28.
1467 Tang F, Wang W, Wang Y, Lee Y, Lou Q. Moderate resistance training reduces intermuscular adipose tissue and risk factors of atherosclerotic cardiovascular disease for elderly patients with type 2 diabetes. Diabetes, Obesity & Metabolism. 2024; 09: 09.
1468 Tiedemann LJ, Meyhofer SM, Francke P, Beck J, Buchel C, Brassen S. Insulin sensitivity in mesolimbic pathways predicts and improves with weight loss in older dieters. eLife. 2022; 11: 27.
1469 Velluzzi F, Cossu G, Fosci M, Montisci R, Zaccheddu R, Minerba L, et al. Effect of a Low-Moderate Exercise Program on Dysmetabolism in Older Adults: Results of a Randomized Controlled Trial. Nutrients. 2022; 14: 15.
1470 Kahleova H, Znayenko-Miller T, Uribarri J, Holubkov R, Barnard ND. Dietary advanced glycation products and their associations with insulin sensitivity and body weight: A 16-week randomized clinical trial. Obesity Science & Practice. 2023; 9: 235–42.
1471 Ghasemi E, Cheraghbirjandi K. Investigating the Effect of a Multicomponent Exercise Program on Adropin, Endothelial Function, Insulin Resistance, and Sleep Quality in Overweight Older Adults (a Link With Physiological Indexes and Sleep Quality): Results of a Randomized Controlled Study. Journal of Aging & Physical Activity. 2024: 1–7.

1472 Kim SW, Park HY, Jung WS, Lim K. Effects of Twenty-Four Weeks of Resistance Exercise Training on Body Composition, Bone Mineral Density, Functional Fitness and Isokinetic Muscle Strength in Obese Older Women: A Randomized Controlled Trial. International Journal of Environmental Research & Public Health [Electronic Resource]. 2022; 19: 06.
1473 Beavers KM, Avery AE, Shankaran M, Evans WJ, Lynch SD, Dwyer C, et al. Application of the D₃ -creatine muscle mass assessment tool to a geriatric weight loss trial: A pilot study. Journal of Cachexia, Sarcopenia and Muscle. 2023; 14: 2350–58.
1474 Azamian Jazi A, Moradi Sarteshnizi E, Fathi M, Azamian Jazi Z. Elastic band resistance training increases adropin and ameliorates some cardiometabolic risk factors in elderly women: A quasi-experimental study. BMC Sports Science, Medicine and Rehabilitation. 2022; 14: 178.
1475 Pleticosic-Ramirez Y, Mecias-Calvo M, Arufe-Giraldez V, Navarro-Paton R. Incidence of a Multicomponent Physical Exercise Program on Body Composition in Overweight or Obese People Aged 60 Years or Older from Chile. Journal of Functional Morphology & Kinesiology. 2024; 9: 24.
1476 Rumbo-Rodriguez L, Zaragoza-Marti A, Sanchez-SanSegundo M, Ferrer-Cascales R, Laguna-Perez A, Hurtado-Sanchez JA. Effectiveness of a Two-Year Multicomponent Intervention for the Treatment of Overweight and Obesity in Older People. Nutrients. 2022; 14: 11.
1477 Sahafi L, Smith D, Jaarsma R, Battersby M. Preliminary Efficacy of a Self-Management Programme to Improve Quality-of-Life in Patients With Obesity and Osteoarthritis Awaiting Arthroplasty: A Randomised Trial. International Journal of Nursing Practice. 2025; 31: e70044.
1478 Hall M, Hinman RS, Knox G, Spiers L, McManus F, De Silva AP, et al. Efficacy of a Very-Low-Calorie Weight Loss Diet Plus Exercise Compared With Exercise Alone on Hip Osteoarthritis Pain : A Randomized Controlled Trial. Annals of Internal Medicine. 2025; 178: 1227–37.
1479 Hartman SJ, LaCroix AZ, Sears DD, Natarajan L, Zablocki RW, Chen R, et al. Impacts of Reducing Sitting Time or Increasing Sit-to-Stand Transitions on Blood Pressure and Glucose Regulation in Postmenopausal Women: Three-Arm Randomized Controlled Trial. Circulation. 2025; 152: 492–504.
1480 Biersteker EJM, van den Helder J, van der Spek N, Holwerda M, Kruizenga H, Weijs PJM, et al. Culture-sensitive lifestyle intervention tailored to non-Western migrant older adults improves physical performance: A randomized controlled trial. Journal of Nutrition, Health & Aging. 2025; 29: 100584.
1481 Jung WS, Ahn H, Kim SW, Park HY. Effects of 12-week Circuit Exercise Intervention on Blood Pressure, Vascular Function, and Inflammatory Cytokines in Obese Older Women with Sarcopenia. Reviews in Cardiovascular Medicine. 2024; 25: 185.
1482 Roemer FW, Kast S, Kemmler W, Collins JE, Engelke K, Guermazi A, et al. Structural effects of whole body electromyostimulation on knee osteoarthritis: the EMSOAT Study. Skeletal Radiology. 2025; 54: 2579–88.
1483 Jung WS, Kim YY, Kim JW, Park HY. Effects of Circuit Training Program on Cardiovascular Risk Factors, Vascular Inflammatory Markers, and Insulin-like Growth Factor-1 in Elderly Obese Women with Sarcopenia. Reviews in Cardiovascular Medicine. 2022; 23: 134.

1484 Perez-Diaz-Del-Campo N, Riezu-Boj JI, Marin-Alejandre BA, Monreal JI, Elorz M, Herrero JI, et al. A nutrigenetic tool for precision dietary management of non-alcoholic fatty liver disease deeming insulin resistance markers. Panminerva Medica. 2022; 64: 485–96.
1485 Wing D, Eyler LT, Lenze EJ, Wetherell JL, Nichols JF, Meeusen R, et al. Fatness but Not Fitness Linked to BrainAge: Longitudinal Changes in Brain Aging during an Exercise Intervention. Medicine & Science in Sports & Exercise. 2024; 56: 655–62.
1486 Freer CL, George ES, Tan SY, Abbott G, Dunstan DW, Daly RM. Effect of progressive resistance training with weight loss compared with weight loss alone on the fatty liver index in older adults with type 2 diabetes: secondary analysis of a 12-month randomized controlled trial. BMJ Open Diabetes Research & Care. 2022; 10: 10.
1487 Madrid DA, Beavers KM, Walkup MP, Ambrosius WT, Rejeski WJ, Marsh AP, et al. Effect of exercise modality and weight loss on changes in muscle and bone quality in older adults with obesity. Experimental Gerontology. 2023; 174: 112126.
1488 Son WH, Park HT, Jeon BH, Ha MS. Moderate intensity walking exercises reduce the body mass index and vascular inflammatory factors in postmenopausal women with obesity: a randomized controlled trial. Scientific Reports. 2023; 13: 20172.
1489 Trico D, Masoni MC, Baldi S, Cimbalo N, Sacchetta L, Scozzaro MT, et al. Early time-restricted carbohydrate consumption vs conventional dieting in type 2 diabetes: a randomised controlled trial. Diabetologia. 2024; 67: 263–74.
1490 Banitalebi E, Banitalebi E, Ghahfarokhi MM, Rahimi M, Laher I, Davison K. Resistance Band Exercise: An Effective Strategy to Reverse Cardiometabolic Disorders in Women With Osteosarcopenic Obesity. Journal of Aging & Physical Activity. 2023; 31: 633–41.
1491 Allison K, Jones S, Hinman RS, Pardo J, Li P, DeSilva A, et al. Alternative models to support weight loss in chronic musculoskeletal conditions: effectiveness of a physiotherapist-delivered intensive diet programme for knee osteoarthritis, the POWER randomised controlled trial. British Journal of Sports Medicine. 2024; 58: 538–47.
1492 Abraham N, Lyden K, Boucher R, Wei G, Gonce V, Carle J, et al. An intervention to decrease sedentary behavior in older adults: A secondary analysis of a randomized controlled trial. Obesity Science & Practice. 2023; 9: 529–37.
1493 Han T, Liang X, Liu H, Zhu M, Shen S, Song J, et al. Muscle-building interventions improve glucose metabolism in elderly type 2 diabetic patients with sarcopenic obesity. Nutrition & Metabolism. 2025; 22: 98.
1494 Pleticosic-Ramirez Y, Arufe-Giraldez V, Rodriguez-Negro J, Mecias-Calvo M, Navarro-Paton R. Is It Possible to Improve the Perceived Quality of Life of Overweight or Obese Older People through a Multicomponent Physical Exercise Program? Behavioral sciences. 2024; 14: 21.
1495 Beavers KM, Lynch SD, Fanning J, Howard M, Lawrence E, Lenchik L, et al. Weighted Vest Use or Resistance Exercise to Offset Weight Loss-Associated Bone Loss in Older Adults: A Randomized Clinical Trial. JAMA Network Open. 2025; 8: e2516772.
1496 Fanning J, Rejeski WJ, Nicklas BJ, Kritchevsky SB, Miller ME, Houston DK, et al. Intervening on Calorie Intake or Eating Timing in Older Adults: Lessons Learned in the Healthy Aging and Late-Life Outcomes Randomized Controlled Pilot Trial. Clinical Interventions In Aging. 2025; 20: 685–700.
1497 Dhana K, Aggarwal NT, Voigt RM, Ventrelle J, Rajan KB, Kaddurah-Daouk R, et al. Effect of weight loss through dietary interventions on cardiometabolic health in older adults. International Journal of Obesity. 2025; 03: 03.

1498 Halloway S, Aggarwal NT, Arfanakis K, Sacks FM, Barnes LL, Dhana K. Effect modifiers of the MIND diet for cognition in older adults: The MIND diet trial. Alzheimer's & Dementia. 2025; 21: e70731.
1499 Lien R, Furlano JA, Witt ST, Xian C, Nagamatsu LS. The effects of a six-month exercise intervention on white matter microstructure in older adults at risk for diabetes. Cerebral Circulation - Cognition and Behavior. 2024; 7: 100369.
1500 Guo X, Cao G, Tang Y, Liu X, Zhou Y, Chen M. 36-Week personalized resistance training improves muscle function and circulating myokines in older women with possible sarcopenic obesity: a randomized clinical trial. BMC Geriatrics. 2025; 25: 702.
1501 Kleinloog JPD, Mensink RP, Roodt JO, Thijssen DHJ, Hesselink MKC, Joris PJ. Aerobic exercise training improves not only brachial artery flow-mediated vasodilatation but also carotid artery reactivity: A randomized controlled, cross-over trial in older men. Physiological Reports. 2022; 10: e15395.
1502 Nijssen KMR, Chavez-Alfaro MA, Joris PJ, Plat J, Mensink RP. Effects of Longer-Term Mixed Nut Consumption on Lipoprotein Particle Concentrations in Older Adults with Overweight or Obesity. Nutrients. 2024; 17: 24.
1503 Nilsson MI, Xhuti D, de Maat NM, Hettinga BP, Tarnopolsky MA. Obesity and Metabolic Disease Impair the Anabolic Response to Protein Supplementation and Resistance Exercise: A Retrospective Analysis of a Randomized Clinical Trial with Implications for Aging, Sarcopenic Obesity, and Weight Management. Nutrients. 2024; 16: 23.
1504 Nijssen KM, Mensink RP, Plat J, Ivanov D, Preissl H, Joris PJ. Mixed nut consumption improves brain insulin sensitivity: a randomized, single-blinded, controlled, crossover trial in older adults with overweight or obesity. American Journal of Clinical Nutrition. 2024; 119: 314–23.
1505 Muollo V, Rossi AP, Milanese C, Cavedon V, Schena F, Giani A, et al. Effects of a Hypocaloric Diet Plus Resistance Training with and Without Amino Acids in Older Participants with Dynapenic Obesity: A Randomized Clinical Trial. Nutrients. 2025; 17: 23.
1506 Lynch DH, Rushing BR, Pathmasiri W, McRitchie S, Batchek DJ, Petersen CL, et al. Baseline Serum Biomarkers Predict Response to a Weight Loss Intervention in Older Adults with Obesity: A Pilot Study. Metabolites. 2023; 13: 17.
